# Catalytic enantioselective synthesis of fluoromethylated stereocenters by asymmetric hydrogenation†

**DOI:** 10.1039/d2sc02685f

**Published:** 2022-06-29

**Authors:** Jianping Yang, Sudipta Ponra, Xingzhen Li, Bram B. C. Peters, Luca Massaro, Taigang Zhou, Pher G. Andersson

**Affiliations:** Department of Organic Chemistry, Stockholm University, Arrhenius Laboratory 106 91 Stockholm Sweden pher.andersson@su.se; College of Chemistry and Chemical Engineering, Southwest Petroleum University Chengdu Sichuan 610500 China tgzhou@swpu.edu.cn; School of Chemistry and Physics, University of Kwazulu-Natal Private Bag X54001 Durban 4000 South Africa

## Abstract

Fluoromethyl groups possess specific steric and electronic properties and serve as a bioisostere of alcohol, thiol, nitro, and other functional groups, which are important in an assortment of molecular recognition processes. Herein we report a catalytic method for the asymmetric synthesis of a variety of enantioenriched products bearing fluoromethylated stereocenters with excellent yields and enantioselectivities. Various N,P-ligands were designed and applied in the hydrogenation of fluoromethylated olefins and vinyl fluorides.

## Abstract 2

Herein, a catalytic asymmetric hydrogenation to synthesize various products bearing fluoromethylated stereocenters has been developed.

## Graphical abstract

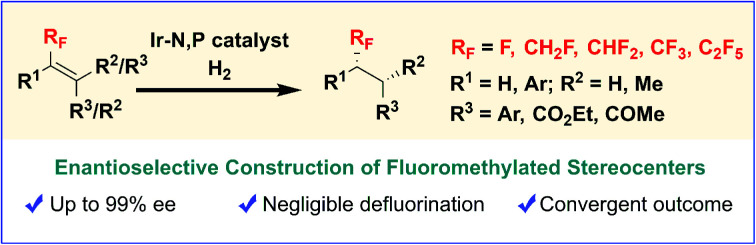

## Introduction

**Fig. 1:**
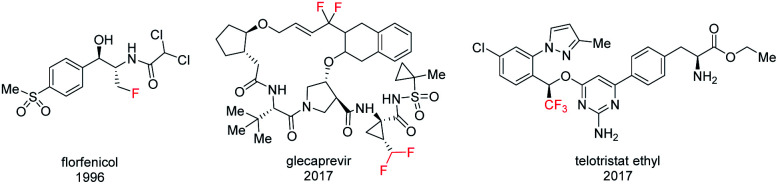
Fluoromethylated drugs.

Organofluorine compounds, on the basis of their special chemical and biological properties, are widely used in pharmaceuticals, agrochemistry, and materials science.^1^ In pharmaceuticals, the incorporation of a fluorine atom or fluorinated group into a biologically active compound usually modifies the biological and physicochemical properties by improving potency, lipophilicity, metabolic stability, binding affinity, and bioavailability.^2^ As a result, fluoromethylated analogues have become a potential class of drug candidates in isostere-based drug design.^3^ In terms of bioisosterism, monofluoromethyl (CH_2_F) and difluoromethyl (CHF_2_) groups are inert, isosteric and isopolar to an OH or SH group in biologically active compounds and pharmaceuticals.^4^ The trifluoromethyl (CF_3_) group could be considered to be bioisosteric with an ethyl group or a potential nitro bioisostere based on its topological, steric, and electronic effect.^3*c*,5^ In addition, mono/difluoromethylated analogues can also serve as a hydrogen donor in binding enzyme active sites. As a result, a variety of structurally diverse CH_2_F, CHF_2_, and CF_3_ containing drugs have been developed (Fig. 1).^1*f*^

**Scheme 1:**
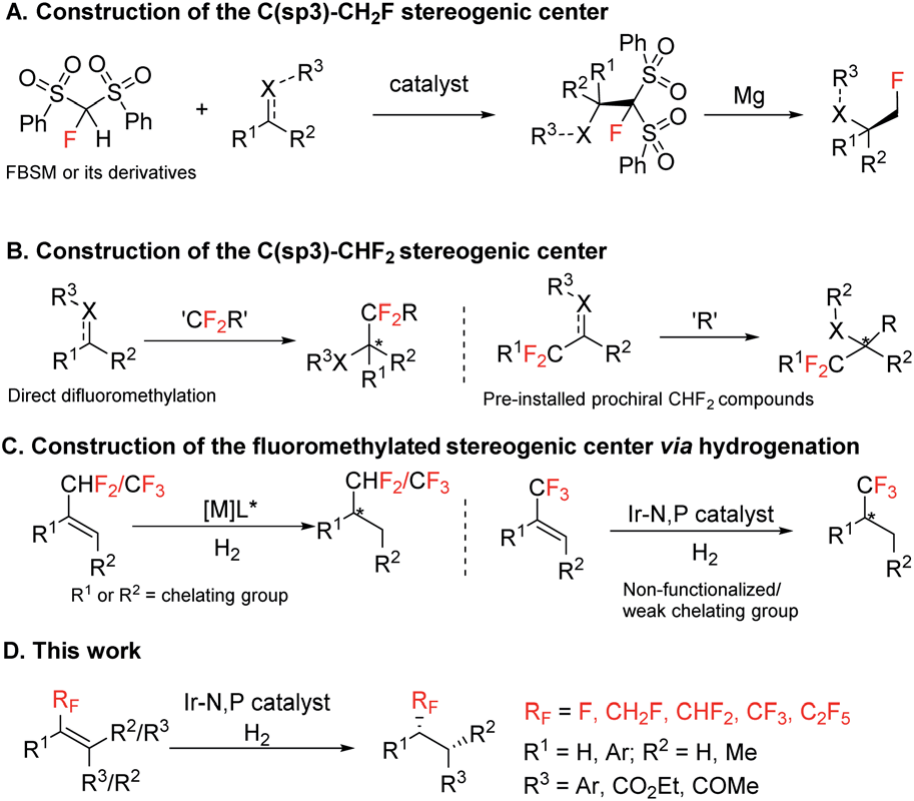
Strategies for the construction of fluoromethylated stereocenters. (A) Construction of the C(sp^3^)–CH_2_F stereogenic center; (B) construction of the C(sp^3^)–CHF_2_ stereogenic center; (C) construction of the fluoromethylated stereogenic center *via* hydrogenation; (D**)** This work.

Hence, in modern organic chemistry and in drug discovery the development of versatile fluoromethylated molecules in an efficient fashion (especially in enantioenriched version) are very active research areas. Although distinct approaches^6^ are available for the asymmetric construction of the C(sp^3^)–CF_3_ function, little attention has been devoted towards asymmetric construction of the C(sp^3^)–CH_2_F and C(sp^3^)–CHF_2_ functions. The most common used strategies for the construction of the C(sp^3^)–CH_2_F stereogenic center are monofluoromethylation using 1-fluorobis (phenylsulfonyl)methane (FBSM), fluoro-(phenylsulfonyl)methane (FSM), 2-fluoro-1,3-benzodithiole-1,1,3,3-tetraoxide (FBDT), or α-fluoro-α-nitro(phenylsulfonyl)-methane as the fluoromethide equivalent (Scheme 1A).^7^ Other strategies consist of diasteroselective monofluoromethylation of chiral *N*-(*tert*-butylsulfinyl) aldimines/ketimines using fluoromethyl phenyl sulfone.^8^ Enantioenriched difluoromethylated compounds are synthesized by reacting nucleophiles or electrophiles with difluoromethylation reagents, for example, PhSO_2_CF_2_H, TMSCF_2_SPh, Me_3_SiCF_2_H, Me_3_SiCF_2_SO_2_Ph, HCF_2_SO_2_Cl, *etc.*, or asymmetric addition of CF_2_H containing prochiral compounds such as imines, olefins, and carbonyl groups (Scheme 1B).^9^ However, the existing methods often require complex reaction conditions. Reduction of fluoromethylalkenes, on the other hand, remains unexplored but could be a broadly effective strategy for the construction of enantioenriched stereogenic centers bearing either CH_2_F, CHF_2_ or CF_3_ group by using a single general strategy.^6*r*,9*a*,10^

In asymmetric catalysis, enantioselective hydrogenation of alkenes using an appropriate transition metal catalyst and chiral ligand is one of the most fundamental and atom-economical processes. Rh and Ru catalysts are widely used for asymmetric hydrogenation of olefins having strong coordinating functional group such as amides or carboxylic acids in close proximity to the double bond.^11^ For olefins having weak coordinating groups or non-coordinating groups, Ir complexes are the most effective catalyst.^12^ Several Ru^II^,^13^ Rh^I^,^14^ and Pd^II^ (ref. 15) complexes were found effective for hydrogenation of some specific CF_3_ substituted olefins with a coordinating group near the substrate double bond (Scheme 1C, left). Fortunately, Ir complexes complement the substrate limitations of Rh/Ru catalyzed enantioselective hydrogenation and are efficient catalysts for enantioselective hydrogenation of CF_3_ substituted unfunctionalized olefins or CF_3_ substituted olefins with the weak chelating group.^16^

Herein, we report a direct catalytic, and highly enantioselective hydrogenation of fluoromethylated olefins for the efficient synthesis of a wide array of chiral building blocks containing fluoromethyl groups.

## Results and discussion

Difluoromethylated olefins were first chosen as the fluoromethylated olefin substrate for our study. We used (*E*)-ethyl 4,4-difluoro-3-phenylbut-2-enoate 1a as the model substrate and an iridium complex with a bicyclic backbone ligand as the catalyst for this asymmetric hydrogenation (Table 1). Hydrogenation of 1a using azabicyclo iridium oxazoline phosphine complex A (1 mol% catalyst, CH_2_Cl_2_, 10 bar H_2_) gave excellent conversion in 4 h but poor enantioselectivity (95% conversion, 21% ee) of the desired product 2a (entry 1). However, the thiazole N,P–iridium complex B dramatically increased the enantioselectivity (91% ee) with very good conversion (91%, entry 2). Based on our previous knowledge of iridium–N,P catalyzed asymmetric hydrogenation,^16*b*^ we investigated the effect of varying the substituents on phosphine. Replacing the aliphatic ^i^Pr group with aromatic group (Ph) resulted in a slight change of enantioselectivity to 92% ee with 72% conversion (entry 3). However, replacing the phenyl group with *ortho*-tolyl group on the bicyclic thiazole iridium–N,P catalyst (catalyst D) resulted in complete conversion (99%) to the desired product 2a with the same level of enantioselectivity (92% ee, entry 4).

**Table 1:** Optimization study^a^

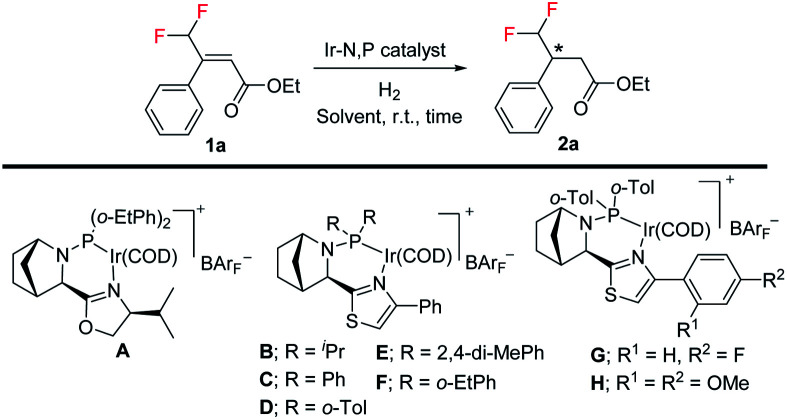						
Entry	Catalyst (mol%)	H_2_ (bar)	Solvent	Time (h)	Conversion (%)	ee (%)
1	A (1.0)	10	CH_2_Cl_3_	4	95	21
2	B (1.0)	10	CH_2_Cl_2_	4	91	91
3	C (1.0)	10	CH_2_Cl_2_	4	72	92
4	D (1.0)	10	CH_2_Cl_2_	4	99	92
5	D (0.5)	5	CH_2_Cl_2_	4	99	92
6	D (0.5)	5	Toluene	4	99	93
7	D (0.5)	5	PhCF_3_	4	99	94
8	E (0.5)	5	PhCF_3_	4	99	94
9	F (0.5)	5	PhCF_3_	4	99	95
**10**	G**(0.5)**	**5**	**PhCF**_**3**_	**4**	**99**	**96**
11	H(0.5)	5	PhCF_3_	4	17	90

a Reaction conditions: 0.05 mmol of 1a, 0.5 mL solvent. The conversion was determined by ^1^H-NMR. Enantiomeric excess was determined by GCMS using a chiral stationary phase.

Further optimization of the reaction conditions with catalyst D was carried out by lowering the catalyst loading from 1.0 mol% to 0.5 mol% as well as the H_2_ pressure from 10 bar to 5 bar, respectively (Table 1, entry 5, for details, see ESI†). Using PhCF_3_ as solvent (entry 7) provided slightly better enantioselectivity (94% ee). To further increase enantioselectivity, we prepared a few new catalysts by varying the electronic density and steric hindrance on phosphorus. Catalyst with 2,4-di-MePh substituent (catalyst E, entry 8) gave the same result as complex D. Changing the *ortho*-tolyl group to an *o*-Etphenyl group afforded new thiazole N,P–iridium complex F with slightly improved enantioselectivity (95% ee, entry 9). Gratifyingly, adding a small electron-withdrawing (F) substituent on the aromatic ring of thiazole moiety (catalyst G) provides the best result in terms of enantioselectivity (96% ee) and conversion (99%, entry 10). On the other hand, the electron-donating (OMe) substituent on aromatic ring of thiazole moiety (catalyst H) led to much lower conversion (17%) and slightly lower enantioselectivity of 90% ee (entry 11). Thus, among these effectively designed new catalyst, a phenyl ring with F atom at *para* position on thiazole moiety and *ortho*-tolyl group on phosphorus (catalyst G, 0.5 mol%) in PhCF_3_ under 5 bar H_2_ pressure for 4 h provided the superior result in enantioselectivity (96% ee) with excellent 99% conversion (entry 10).

With the optimized reaction conditions established, we evaluated the hydrogenation of various (*E*)-fluorinated olefins 1 having different substituents (Table 2). A variety of difluoromethylated olefins (*E*)-1a–1l having different ester groups and with either electron-donating or electron-withdrawing substituents on the phenyl rings were successfully hydrogenated to deliver the desired products 2a–2l in excellent yield (94–99%) and enantioselectivities (90–98% ee). When evaluating the *Z*-isomer (*Z*-1f, *Z*-1g and *Z*-1m), lower enantioselectivities but the same major enantiomers were observed (83% ee, 75% ee and 72% ee, respectively). Interestingly, substrates with electron-withdrawing substituents seem advantageous for higher enantioselectivity. Carbocyclic CHF_2_ olefins (1n–1o) were hydrogenated in excellent yield but with significant variations in enantioselectivities. Benzo-fused cyclohexyl ring substrate 1n gave 77% ee while substrate with five-member ring (1o) provided 99% ee. Aliphatic CHF_2_ olefins were also tested and they generally resulted in lower reactivity. Nevertheless, we managed to hydrogenate compound 1p with a moderate conversion and good ee (90%). When the H on CHF_2_ group was replaced by strong electron-withdrawing CF_3_ group, the olefin was also hydrogenated much sluggishly and provided 2q in only 43% yield with 81% ee. After successfully hydrogenating various trisubstituted (*E*)-CHF_2_ olefins, tetrasubstituted CHF_2_ olefin (1r) was efficiently hydrogenated (2r) in 99% yield, excellent diastereoselectivity (>99% d.r.) and enantioselectivity (91% ee). The effectiveness of this stereoselective hydrogenation process was further investigated by evaluating various CF_3_ containing olefins to produce chiral CF_3_ alkanes. Various CF_3_ containing trisubstituted β,β-unsaturated esters or ketone were successfully hydrogenated under the standard conditions with good yields (80–99%) and enantioselectivities (87–96% ee, 2s–2u). Similarly, the developed protocol was equally efficient for tetra-substituted CF_3_ containing aliphatic olefins (1v and 1w) which were efficiently hydrogenated in excellent yields (99%) and diastereoselectivities (>99% d.r.) with high enantioselectivity (88% and 82% ee respectively). In addition, CH_2_F containing olefin (1x) was also hydrogenated in an exceptionally good yield (99%) with good enantioselectivity (84% ee) and slight defluorination (9%). The successful examples in Table 2 emphasizes that this azabicyclo iridium thiazole phosphine catalyst is very general for various fluoromethylated olefins.

**Table 2:** Substrate scope^a^

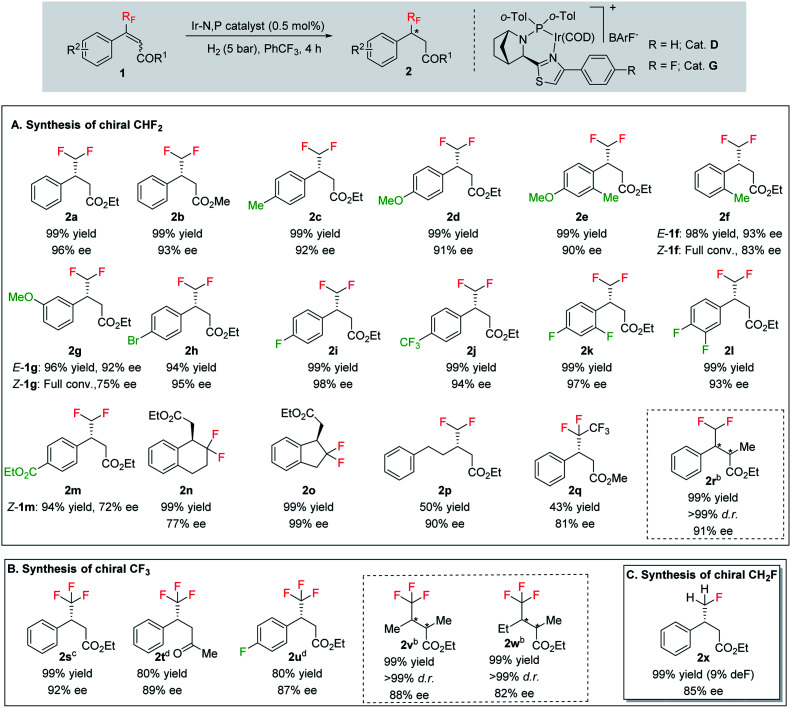

a Reaction conditions: 0.15 mmol of *E*-substrate, 0.5 mol% catalyst G, 5 bar H_2_, 1.5 mL PhCF_3_, 4 h.

b 1.0 mol% catalyst *ent*-D, 100 bar H_2_.

c 0.5 mol% catalyst D, 10 bar H_2_, 1.5 mL CH_2_Cl_2_.

d 2.0 mol% catalyst D, 10 bar H_2_, 1.5 mL CH_2_Cl_2_. Yields are isolated hydrogenated product. Enantiomeric excess was determined by SFC or GCMS using chiral stationary phases.

To further study the effectiveness of this developed method for the catalytic asymmetric synthesis of fluoromethylated stereogenic centers, a different class of olefins (vinyl fluoride), which affords the chiral monofluorinated molecule, was also evaluated. For these vinyl fluorides, catalyst B (1 mol%) was the most suitable catalyst using 20 bar H_2_ pressure for 24 h (see ESI† for optimization details). Employing the newly optimized reaction conditions, a variety of unfunctionalized naphthalene fused vinyl-fluoride substrates were efficiently hydrogenated in excellent enantioselectivity (90–98% ee, Table 3, 4a–h) although in some cases the conversions are low (3c, 73%; 3d, 40%; 3e, 40%; 3h, 70%). Notably, substrates having the bulky secondary (^i^Pr, Cy) substituent were hydrogenated in high levels of stereoselectivity (4d–e). Both substrates with electron-donating (Me, OMe) or electron-withdrawing (F) substituents were tolerated (3f–h), however; substrates bearing electron-donating substituents were slightly more favorable in terms of reactivity (3f–g). A small amount of de-F byproduct (3–11%) were detected in the hydrogenation, however considering the challenges generally associated with hydrogenation of vinyl-fluoride, this efficient hydrogenation still highlights this catalytic protocol as general for fluorine-containing olefins to synthesize enantioenriched fluoromethylated compounds.

**Table 3:** Hydrogenation of various vinyl-fluorides^a^

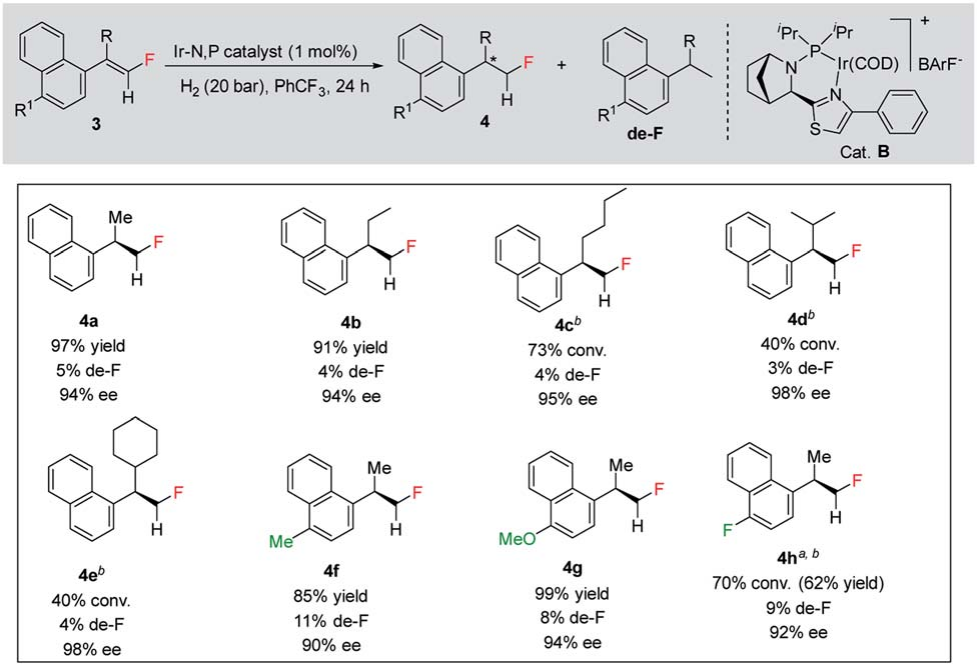

a Reaction conditions: 0.15 mmol of substrate, 1.0 mol% catalyst B, 3 mL PhCF_3_, 20 bar H_2_. Yields are isolated hydrogenated product.

b The conversion was determined by ^1^H-NMR. Enantiomeric excess was determined by HPLC or GCMS using chiral stationary phases.

Interestingly, in this work, an enantioconvergent outcome was observed, where the *E* and *Z* isomers of fluoromethylated olefins were successfully hydrogenated using catalyst *ent*-D. Both isomers produced the same enantiomer in favor. The three different types of fluoromethylated olefins, including CH_2_F, CHF_2_ and CF_3_ groups, underwent enantioconvergent hydrogenation (Table 4, entries 1–3). However, removal of fluorine from the substrate (Table 4, entry 4) provided an enantiodivergent hydrogenation outcome (Table 4, entry 4), which suggested fluorine played an important role in the enantio-discrimination step. Our recent work on an efficient convergent hydrogenation using Ir–N,P complexes with a weak chelating group on the double bond suggested that α-prochiral olefins underwent an enantioconvergent hydrogenation while β-prochiral olefins reacted in an enantiodivergent manner.^17^ In this case, conversely, β-prochiral fluoromethylated olefin react in an enantioconvergent manner. We speculate that this could be due to the chelation effect or the electronic effect of the fluorine atom. Further investigations are still in progress.

**Table 4:** Hydrogenation of both *E* and *Z* isomers of fluoromethylated and non-fluoromethylated olefins^a^

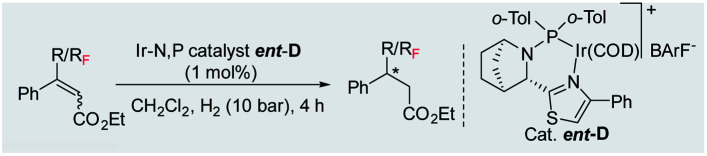					
Entry	Olefin	Isomer	Product	Conversion (%)	ee (%)
1	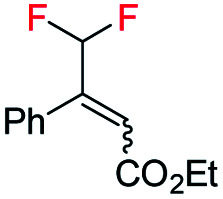	*E*-1a	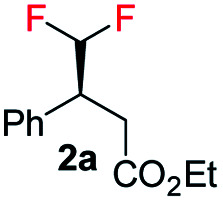	99	92 *(S)*
		*Z*-1a		99	55 *(S)*
		*E*/*Z* (1 : 1)		99	71 *(S)*
2	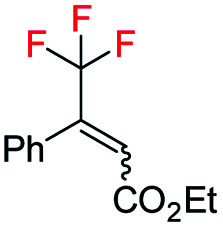	*E*-1s	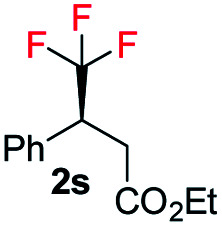	99	92 *(S)*
		*Z*-1s		99	26 *(S)*
		*E*/*Z* (1 : 1)		99	56 *(S)*
3	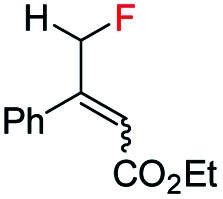	*E*-1x	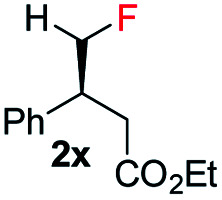	99 (9% de-F)	84 *(S)*
		*Z*-1x		69 (32% de-F)	56 *(S)*
		*E/Z (1:1)*		99 (30% de-F)	76 *(S)*
4	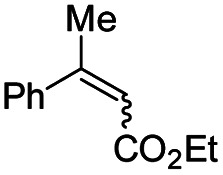	*E*-6	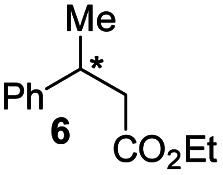	99	99 *(S)*
		*E*-6		99	62 *(R)*

a Reaction conditions: 0.05 mmol of substrate, 1.0 mol% catalyst *ent*-D, 0.5 mL PhCF_3_, 10 bar H_2_. Enantiomeric excess was determined by SFC or GCMS using chiral stationary phases.

**Scheme 2:**
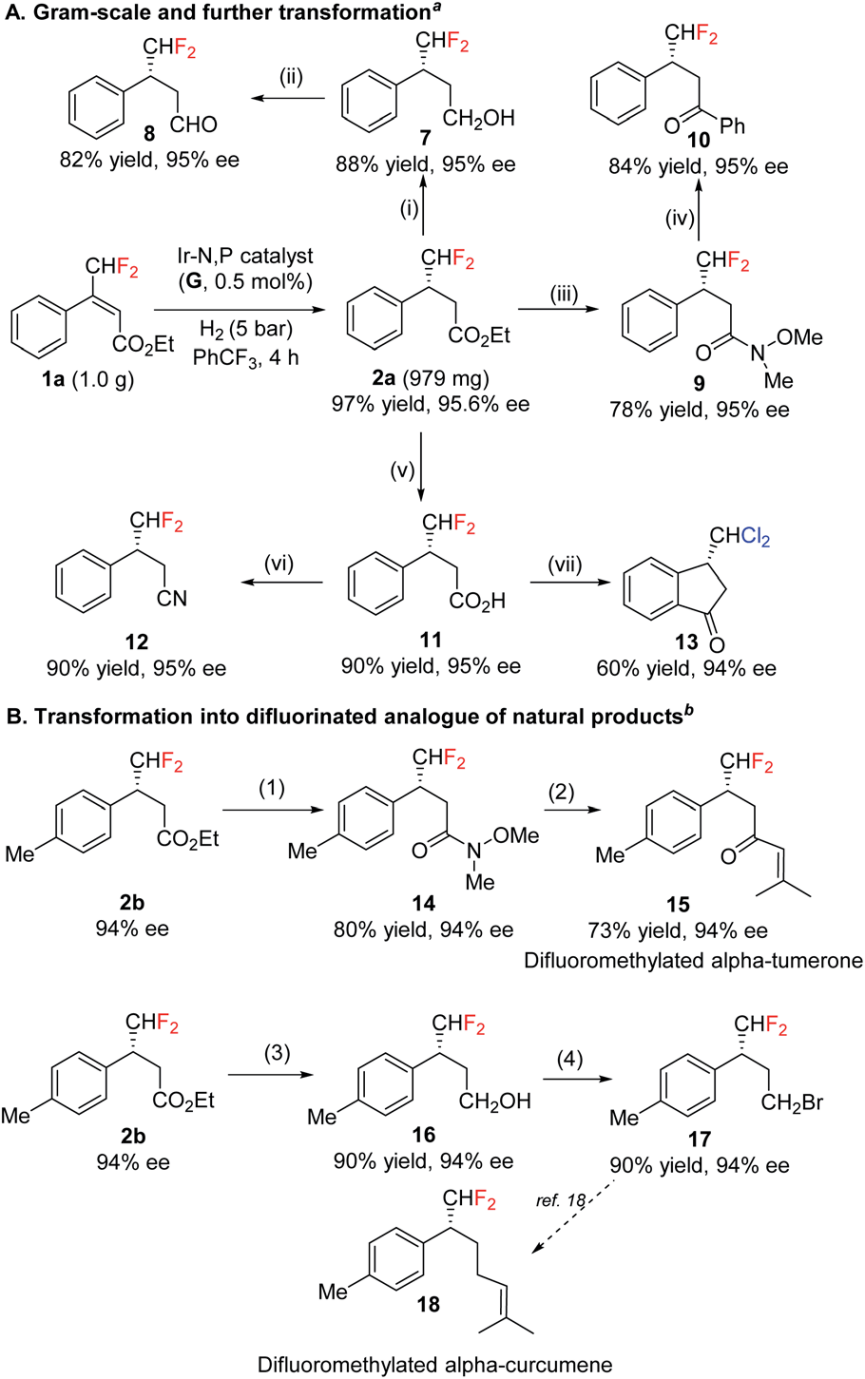
Synthesis of chiral difluoromethylated compounds having different functional group. (A) Gram-scale and further transformation; (B) transformation into difluorinated analogue of natural products. ^*a*^Reaction conditions: (i) LiAlH_4_, THF, 0 °C; (ii) DMP, DCM, r.t.; (iii) ^i^PrMgBr, NH(OMe)Me·HCl, THF, 0 °C; (iv) PhMgCl, THF, 0 °C; (v) 2 M NaOH (aq.), MeOH, reflux; (vi) NH_4_HCO_3_, Boc_2_O, dioxane, r.t.; NEt_3_, (COCF_3_)_2_O, DCM, 0 °C – r.t.; (vii) cyanuric trichloride, pyridine, AlCl_3_, DCM. ^*b*^Reaction conditions: (1) ^i^PrMgBr, NH(OMe)Me·HCl, THF, 0 °C; (2) 1-bromo-2-methylprop-1-ene, *^n^*BuLi, −78–0 °C; (3) LiAlH_4_, THF, 0 °C; (4) CBr_4_, PPh_3_, r.t.

The efficacy of the asymmetric synthesis of fluoromethylated compounds were investigated in gram-scale under standard reaction conditions. Product 2a was obtained in 97% yield with 96% ee (Scheme 2). This synthesized enantioenriched fluromethylated compound was transformed into a variety of many useful chiral fluorinated derivatives, such as alcohol, aldehyde, acid, Weinreb amide, ketone and nitrile (Scheme 2A) with almost perfect retention of enantiopurity. Interestingly, acid 11 provided (*S*)-3-(dichloromethyl)-2,3-dihydro-1*H*-inden-1-one 13 under Friedel–Crafts reaction condition. In the presence of AlCl_3_, difluoromethyl group underwent halogen exchange while preserving enantiomeric purity. Based on these successful transformations, some difluoromethylated natural products were accessed (Scheme 2B). Weinreb amide 14 was further transformed into difluorinated analogue of natural products 15. Synthetically versatile intermediate alcohol was transferred into bromide 17 which could be further transformed into the difluorinated analogue of alpha-curcumene 18.^18^

## Conclusions

In summary, we have developed a catalytic, asymmetric methodology to synthesize various products bearing fluoromethylated stereocenters, which are important bioisostere in drug discovery. Different types of fluoromethylated olefins and vinyl fluorides were hydrogenated successfully by effective new catalyst design. In addition, an interesting enantioconvergency was observed, which indicated that fluorine has the potential to control the enantioselectivity due to its special properties.

## Data availability

All experimental data associated with this work are available in the ESI.†

## Author contributions

P. G. Andersson and T. Zhou supervised the project and conceived experiments. J. Yang and S. Ponra designed the project, optimized the reaction, performed the major of experiments, and prepared the Supporting Information. X. Li, B. B. C. Peters, and L. Massaro prepared some of the starting materials and evaluated some hydrogenation reactions. P. G. Andersson, J. Yang, and S. Ponra wrote the paper. All authors discussed the results and commented on the manuscript.

## Conflicts of interest

There are no conflicts to declare.

## Supplementary Material

SC-013-D2SC02685F-s001
