# Supplementary material for: Catalytic enantioselective synthesis of fluoromethylated stereocenters by asymmetric hydrogenation
Source: Chem Sci. 2022 Jun 29;13(29):8590–6. doi: 10.1039/d2sc02685f (PMC9337738; doi:10.1039/d2sc02685f)
Supplement: SC-013-D2SC02685F-s001 [file SC-013-D2SC02685F-s001.pdf]

Electronic Supplementary Material (ESI) for Chemical Science.
This journal is © The Royal Society of Chemistry 2022

Catalytic Enantioselective Synthesis of Fluoromethylated Stereocenters by Hydrogenation

Jianping Yang†[a], Sudipta Ponra†[a], Xingzhen Li[b], Bram Peters[a], Luca Massaro[a], Taigang Zhou*[b] and Pher G Andersson*[a, c]

[a] Department of Organic Chemistry, Stockholm University, Svante Arrhenius väg 16C, SE-10691 Stockholm, Sweden. E-mail: pher.andersson@su.se

[b] College of Chemistry and Chemical Engineering, Southwest Petroleum University, Chengdu, Sichuan 610500, China. E-mail: tgzhou@swpu.edu.cn

[c] School of Chemistry and Physics, University of KwaZulu-Natal, Private Bag X54001, Durban, 4000, South Africa.

† Authors contributed equally to this work

Table of contents:

Table of contents: 1
1. General methods 1
2. Additional condition screenings 2
3. General procedure for synthesis of fluoromethylated alkenes 3
4. General procedure for synthesis of monofluoroalkenes 9
5. General procedure for asymmetric hydrogenation 11
6. General procedure for further transformation 21
7. Separation method data and specific rotation of chiral compounds 25
8. Absolute configuration determination 30
9. NMR Spectra 31
10. Chromatograms 139
11. References 162

1. General methods

All reactions were conducted under inert atmosphere using magnetic stirring. CH_2Cl_2, used in the hydrogenation, was freshly distilled from CaH_2 under nitrogen. THF was freshly distilled from sodium-benzophenone under nitrogen. All reagents were used as supplied commercially without further purification. Chromatographic separations were performed on Kiesel gel 60 H silica gel (particle size: 0.063-0.100 mm). Thin layer chromatography (TLC) was performed on aluminium plates coated with Kiesel gel 60 (0.20 mm, UV254) and visualized under ultraviolet light (ν = 254 nm). ^{1}H NMR spectra were recorded on a Bruker 400 or 500 at 400/500 MHz in $CDCl_3$ and referenced internally to the residual $CHCl_3$ peak (7.26 ppm). ^{13}C NMR spectra were recorded at 100/125 MHz in $CDCl_3$ and referenced to the central peak of $CDCl_3$ (77.16 ppm). Chemical shifts are reported in ppm (δ scale). Enantiomeric excesses were determined either using chiral HPLC, SFC or GCMS. Racemic compounds were used for comparison. HRMS data were obtained using a Bruker MicroTOF-Q II instrument operation at ambient temperature. Optical rotations were recorded on an Autopol IV polarimeter from Rudolp Research Analytical, equipped with a sodium lamp (589 nm) and a 10 cm cell.

2. Additional condition screenings

Table S1 Condition screenings for CHF_2 substrates

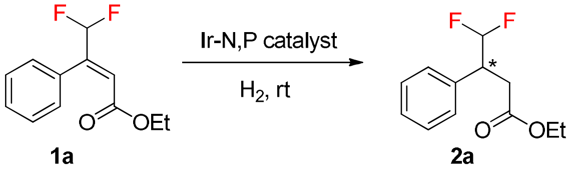

Entry	Catalyst (mol%)	H_2 (bar)	Solvent	Time (h)	Conversion (%)	ee (%)
1.	**A** (1.0)	10	CH_2Cl_2	4	95	21
2.	**B** (1.0)	10	CH_2Cl_2	4	91	91
3.	**C** (1.0)	10	CH_2Cl_2	4	72	92
4.	**D** (1.0)	10	CH_2Cl_2	4	99	92
5.	**D** (0.5)	10	CH_2Cl_2	4	99	92
6.	**D** (0.2)	10	CH_2Cl_2	4	68	89
7.	**D** (0.5)	5	CH_2Cl_2	4	99	92
8.	**D** (0.5)	2	CH_2Cl_2	4	60	92
9.	**D** (0.5)	5	Toluene	4	99	93
10.	**D** (0.5)	5	$PhCF_3$	4	99	94
11.	**D** (0.5)	5	$PhCF_3$	1	89	94
12.	**E** (0.5)	5	$PhCF_3$	4	99	94
13.	**F** (0.5)	5	$PhCF_3$	4	99	95
14.	**G** (0.5)	5	$PhCF_3$	4	99	96
15.	**H** (0.5)	5	$PhCF_3$	4	17	90

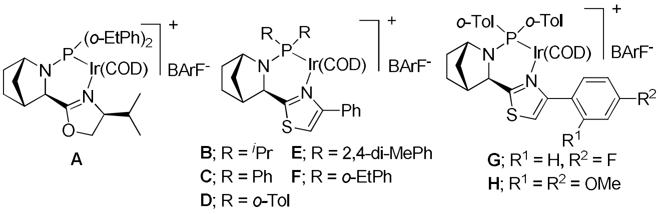

Reaction conditions: 0.05 mmol of **1a**, 0.5 mL solvent. The conversion was determined by ^1H-NMR. Enantiomeric excess was determined by GC/MS using chiral stationary phases.

Table S2 Condition screenings for vinyl fluorides

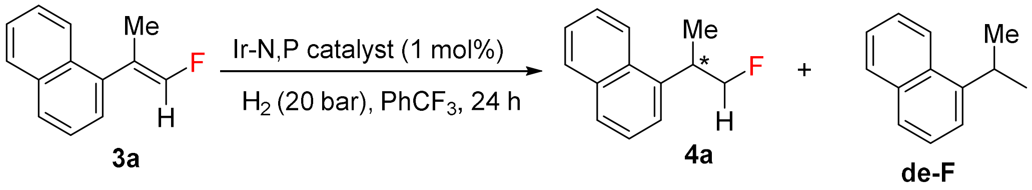

Entry	Catalyst (mol%)	H_2 (bar)	Solvent	Conversion (%)	de-F (%)	ee (%)
1.	**B** (1.0)	20	CH_2Cl_2 (0.1 M)	66	11	94
2.	**C** (1.0)	20	CH_2Cl_2 (0.1 M)	24	26	62
3.	**D** (1.0)	20	CH_2Cl_2 (0.1 M)	96	21	77
4.	**B** (1.0)	20	Toluene (0.1 M)	22	9	87
5.	**B** (1.0)	20	$PhCF_3$ (0.1 M)	99	5	93
6.	**B** (1.0)	20	$PhCF_3$ (0.05 M)	99	5	94
7.	**B** (1.0)	20	$PhCF_3$ (0.025 M)	68	5	94

Reaction conditions: 0.05 mmol of **3a**, 0.5 – 2 mL of solvent, 24 h. The conversion was determined by ^{1}H-NMR. Enantiomeric excess was determined by GC/MS using chiral stationary phases.

3. General procedure for synthesis of fluoromethylated alkenes

3.1 General procedure for the synthesis of difluoroketones

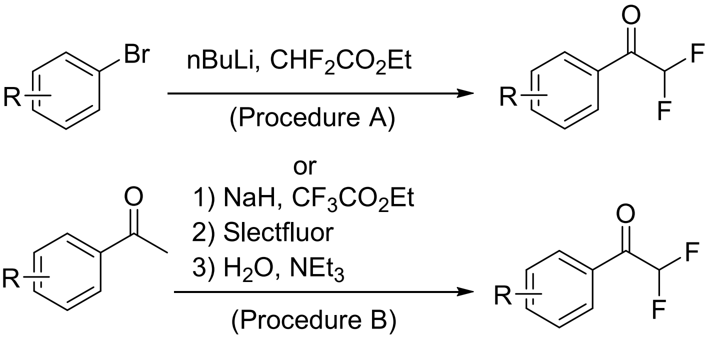

The corresponding difluoroketones were prepared following procedure A or B, which was carried out according to the literature.[1]

3.2 General procedure for the synthesis of di/tri-fluoromethylated olefins

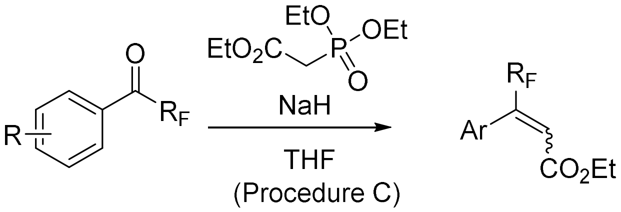

Di/tri-fluoromethylated olefins were prepared following procedure C, which was carried out according to the literature.[2]

New compounds:

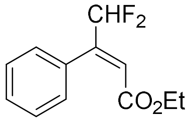

Ethyl (*E*)-4,4-difluoro-3-phenylbut-2-enoate (1a)

Colorless oil. ^{1}H NMR (400 MHz, Chloroform-*d*) δ 7.69 (t, *J* = 54.03 Hz, 1H), 7.59 – 7.50 (m, 2H), 7.47 – 7.35 (m, 3H), 6.29 (s, 1H), 4.27 (q, *J* = 7.14 Hz, 2H), 1.34 (t, *J* = 7.14 Hz, 3H). ^{13}C NMR (101 MHz, Chloroform-*d*) δ 164.7, 149.2 (t, *J* = 23.28 Hz), 134.1, 129.8, 128.6, 128.2, 124.9 (t, *J* = 7.88 Hz), 110.7 (t, *J* = 236.76 Hz), 61.4, 14.3. ^{19}F NMR (377 MHz, Chloroform-*d*) δ -116.1. HRMS-ESI: Found $[M+Na]^+$ = 249.0680; $C_{12}H_{12}F_2O_2Na$ requires 249.0698.

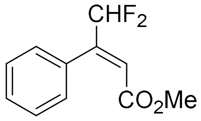

Methyl (*E*)-4,4-difluoro-3-phenylbut-2-enoate (1b)

Colorless oil. ^{1}H NMR (400 MHz, Chloroform-*d*) δ 7.71 (t, *J* = 54.71 Hz, 1H), 7.58 – 7.49 (m, 2H), 7.46 – 7.36 (m, 3H), 6.31 (s, 1H), 3.82 (s, 3H). ^{13}C NMR (101 MHz, Chloroform-*d*) δ 165.1, 149.5 (t, *J* = 23.37 Hz), 134.0, 129.9, 128.6, 128.2, 124.3 (t, *J* = 7.89 Hz), 110.7 (t, *J* = 236.79 Hz), 52.2. ^{19}F NMR (377 MHz, Chloroform-*d*) δ -116.1. HRMS-ESI: Found $[M+Na]^+$ = 235.0544; $C_{11}H_{10}F_2O_2Na$ requires 235.0541.

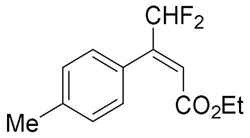

Ethyl (*E*)-4,4-difluoro-3-(p-tolyl)but-2-enoate (1c)

Colorless oil. ^{1}H NMR (400 MHz, Chloroform-*d*) δ 7.69 (t, *J* = 54.72 Hz, 1H), 7.45 (d, *J* = 8.12 Hz, 2H), 7.21 (d, *J* = 7.98 Hz, 2H), 6.28 (s, 1H), 4.26 (q, *J* = 7.14 Hz, 2H), 2.38 (s, 3H), 1.34 (t, *J* = 7.14 Hz, 3H). ^{13}C NMR (101 MHz, Chloroform-*d*) δ 164.9, 149.2 (t, *J* = 23.12 Hz), 140.1, 131.1, 129.4, 128.1, 124.0 (t, *J* = 7.88 Hz), 110.8 (t, *J* = 236.69 Hz), 61.3, 21.4, 14.3. ^{19}F NMR (377 MHz, Chloroform-*d*) δ -116.1. HRMS-ESI: Found $[M+Na]^+$ = 263.0862; $C_{13}H_{14}F_2O_2Na$ requires 263.0854.

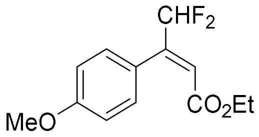

Ethyl (*E*)-4,4-difluoro-3-(4-methoxyphenyl)but-2-enoate (1d)

Colorless oil. ^{1}H NMR (400 MHz, Chloroform-*d*) δ 7.72 (t, *J* = 54.72 Hz, 1H), 7.56 – 7.48 (m, 2H), 6.96 – 6.88 (m, 2H), 6.26 (s, 1H), 4.26 (q, *J* = 7.13 Hz, 2H), 3.84 (s, 3H), 1.33 (t, *J* = 7.12 Hz, 3H). ^{13}C NMR (101 MHz, Chloroform-*d*) δ 165.0, 161.1, 148.7 (t, *J* = 23.18 Hz), 129.7, 126.2, 123.0 (t, *J* = 7.82 Hz), 114.1, 110.9 (t, *J* = 236.71 Hz), 61.2, 55.5, 14.3. ^{19}F NMR (377 MHz, Chloroform-*d*) δ -116.2. HRMS-ESI: Found $[M+Na]^+$ = 279.0812; $C_{13}H_{14}F_2O_3Na$ requires 279.0803.

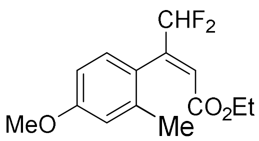

Ethyl (*E*)-4,4-difluoro-3-(4-methoxy-2-methylphenyl)but-2-enoate (1e)

Colorless oil. ^{1}H NMR (400 MHz, Chloroform-*d*) δ 7.71 (t, *J* = 54.78 Hz, 1H), 7.43 – 7.36 (m, 1H), 7.38 – 7.32 (m, 1H), 6.83 (d, *J* = 8.57 Hz, 1H), 6.25 (s, 1H), 4.25 (q, *J* = 7.14 Hz, 2H),

3.86 (s, 3H), 2.23 (s, 3H), 1.33 (t, J = 7.14 Hz, 3H). ^{13}C NMR (101 MHz, Chloroform-d) δ 165.0, 159.4, 148.9 (t, J = 22.57 Hz), 130.4, 127.3, 127.0, 125.7, 122.7 (t, J = 7.81 Hz), 111.0 (t, J = 236.67 Hz), 109.8, 61.2, 55.5, 16.4, 14.3. ^{19}F NMR (377 MHz, Chloroform-d) δ -116.1. HRMS-ESI: Found [M+Na]$^+$ = 293.0961; $C_{14}H_{16}F_2O_3Na$ requires 293.0960.

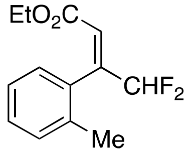

Ethyl (*E*)-4,4-difluoro-3-(*o*-tolyl)but-2-enoate (*E*-1f)

Colorless oil. ^{1}H NMR (400 MHz, Chloroform-d) δ 7.54 (t, J = 55.5 Hz, 1H), 7.32 – 7.16 (m, 4H), 6.04 (s, 1H), 4.28 (q, J = 7.1 Hz, 2H), 2.30 (s, 3H), 1.34 (t, J = 7.1 Hz, 3H). ^{13}C NMR (101 MHz, Chloroform-d) δ 164.5, 149.8 (t, J = 24.5 Hz), 136.4, 134.0, 130.4, 129.4, 128.9, 126.9 (t, J = 7.7 Hz), 125.5, 110.2 (t, J = 236.0 Hz), 61.4, 20.2, 14.3. ^{19}F NMR (377 MHz, Chloroform-d) δ -117.3 ppm. HRMS-ESI: Found [M+Na]$^+$ = 263.0849; $C_{13}H_{14}F_2O_2Na$ requires 263.0854.

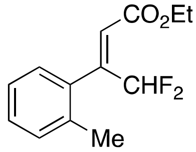

Ethyl (*Z*)-4,4-difluoro-3-(*o*-tolyl)but-2-enoate (*Z*-1f)

Colorless oil. ^{1}H NMR (400 MHz, Chloroform-d) δ 7.36 – 7.21 (m, 3H), 7.10 (d, J = 7.5 Hz, 1H), 6.47 (t, J = 2.0 Hz, 1H), 6.23 (t, J = 55.2 Hz, 1H), 4.06 (q, J = 7.1 Hz, 2H), 2.29 (s, 3H), 1.08 (t, J = 7.1 Hz, 3H). ^{13}C NMR (101 MHz, Chloroform-d) δ 164.4, 147.3 (t, J = 20.4 Hz), 136.3, 132.4, 129.9, 128.7, 128.1, 125.5, 123.7 (t, J = 8.7 Hz), 114.3 (t, J = 243.0 Hz), 60.7, 19.6, 13.8. ^{19}F NMR (377 MHz, Chloroform-d) δ -117.3. HRMS-ESI: Found [M+Na]$^+$ = 263.0874; $C_{13}H_{14}F_2O_2Na$ requires 263.0854.

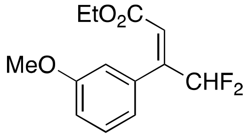

Ethyl (*E*)-4,4-difluoro-3-(3-methoxyphenyl)but-2-enoate (*E*-1g)

Yellow oil.^{1}H NMR (400 MHz, Chloroform-d) δ 7.66 (t, J = 54.8 Hz, 1H), 7.35 – 7.25 (m, 1H), 7.15 – 7.10 (m, 1H), 7.06 (s, 1H), 7.02 – 6.93 (m, 1H), 6.29 (s, 1H), 4.27 (q, J = 7.2 Hz, 2H), 3.83 (s, 3H), 1.34 (t, J = 7.1 Hz, 3H). ^{13}C NMR (101 MHz, Chloroform-d) δ 164.7, 159.6, 149.0 (t, J = 23.3 Hz), 135.4, 129.7, 125.0 (t, J = 7.9 Hz), 120.6, 115.4, 113.8, 110.6 (t, J = 236.9 Hz), 61.4, 55.4, 14.2. ^{19}F NMR (377 MHz, Chloroform-d) δ -116.2. HRMS-ESI: Found [M+Na]$^+$ = 279.0826; $C_{13}H_{14}F_2O_3Na$ requires 279.0803.

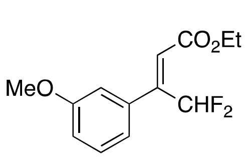

Ethyl (*Z*)-4,4-difluoro-3-(3-methoxyphenyl)but-2-enoate (*Z*-1g)

Yellow oil. ^{1}H NMR (400 MHz, Chloroform-d) δ 7.30 (t, J = 15.9 Hz, 1H), 6.93 (ddd, J = 8.4, 2.6, 1.0 Hz, 1H), 6.84 (d, J = 7.6 Hz, 1H), 6.81 (t, J = 2.1 Hz, 1H), 6.35 (s, 1H), 6.23 (t, J = 55.3 Hz, 1H), 4.06 (q, J = 7.1 Hz, 2H), 3.81 (s, 3H), 1.09 (t, J = 7.1 Hz, 3H). ^{13}C NMR (101 MHz, Chloroform-d) δ 164.9, 159.4, 146.6 (t, J = 20.2 Hz), 134.0, 129.4, 123.2 (t, J = 8.9 Hz), 120.9, 114.6, 114.3 (t, J = 242.9 Hz), 114.2, 61.0, 55.4, 14.0. ^{19}F NMR (377 MHz, Chloroform-d) δ -116.3. HRMS-ESI: Found [M+Na]$^+$ = 279.0800; $C_{13}H_{14}F_2O_3Na$ requires 279.0803.

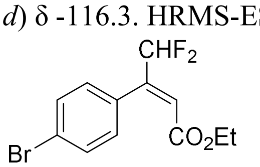

Ethyl (*E*)-3-(4-bromophenyl)-4,4-difluorobut-2-enoate (1h)

Colorless oil. ^{1}H NMR (400 MHz, Chloroform-*d*) δ 7.74 (t, *J* = 54.65 Hz, 1H), 7.53 (d, *J* = 8.50 Hz, 2H), 7.41 (d, *J* = 8.37 Hz, 2H), 6.28 (s, 1H), 4.26 (q, *J* = 7.14 Hz, 2H), 1.33 (t, *J* = 7.12 Hz, 3H). ^{13}C NMR (101 MHz, Chloroform-*d*) ^{13}C NMR (101 MHz, Chloroform-*d*) δ 164.4 (d, *J* = 1.99 Hz), 148.0 (t, *J* = 23.47 Hz), 132.9, 131.9, 129.8, 125.2 (t, *J* = 7.83 Hz), 110.5 (t, *J* = 236.87 Hz), 61.5, 14.2. ^{19}F NMR (376 MHz, Chloroform-*d*) δ -116.2. HRMS-ESI: Found $[M+Na]^+$ = 326.9813; $C_{12}H_{11}F_2BrO_2Na$ requires 326.9803 (^{79}Br).

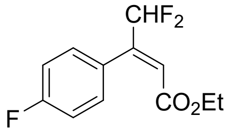

Ethyl (*E*)-4,4-difluoro-3-(4-fluorophenyl)but-2-enoate (1i)

Colorless oil. ^{1}H NMR (400 MHz, Chloroform-*d*) δ 7.81 (t, *J* = 56.02 Hz, 1H), 7.55 – 7.50 (m, 2H), 7.14 – 7.01 (m, 2H), 6.26 (s, 1H), 4.27 (q, *J* = 7.14 Hz, 2H), 1.34 (t, *J* = 7.14 Hz, 3H). ^{13}C NMR (101 MHz, Chloroform-*d*) δ 164.6, 163.8 (d, *J* = 250.23 Hz), 148.1 (t, *J* = 23.40 Hz), 130.3, 130.2, 124.8 (t, *J* = 7.34 Hz), 115.8 (d, *J* = 21.72 Hz), 110.6 (t, *J* = 236.72 Hz), 61.4, 14.3. ^{19}F NMR (377 MHz, Chloroform-*d*) δ -111.0, -116.3. HRMS-ESI: Found $[M+Na]^+$ = 267.0610; $C_{12}H_{11}F_3O_2Na$ requires 267.0603.

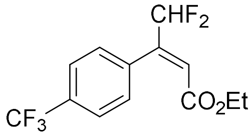

Ethyl (*E*)-4,4-difluoro-3-(4-(trifluoromethyl)phenyl)but-2-enoate (1j)

Colorless oil. ^{1}H NMR (400 MHz, Chloroform-*d*) δ 7.83 – 7.48 (m, 5H), 6.31 (td, *J* = 0.88, 0.70 Hz, 1H), 4.29 (q, *J* = 7.13 Hz, 2H), 1.35 (t, *J* = 7.15 Hz, 3H). ^{13}C NMR (101 MHz, Chloroform-*d*) δ 164.3 (t, *J* = 1.87 Hz), 147.9 (t, *J* = 23.66 Hz), 137.6, 131.8 (q, *J* = 32.67 Hz), 128.7, 126.4 (t, *J* = 7.80 Hz), 125.6 (q, *J* = 3.76 Hz), 124.0 (q, *J* = 274.50 Hz), 110.4 (t, *J* = 236.99 Hz), 61.7, 14.3. ^{19}F NMR (377 MHz, Chloroform-*d*) δ -62.9, -116.1. HRMS-ESI: Found $[M+Na]^+$ = 317.0566; $C_{13}H_{11}F_5O_2Na$ requires 317.0571.

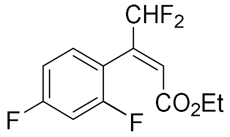

Ethyl (*E*)-3-(2,4-difluorophenyl)-4,4-difluorobut-2-enoate (1k)

Colorless oil. ^{1}H NMR (400 MHz, Chloroform-*d*) δ 7.55 (dt, *J* = 54.70, 1.09 Hz, 1H), 7.44 – 7.36 (m, 1H), 6.98 – 6.83 (m, 2H), 6.24 (s, 1H), 4.28 (q, *J* = 7.15 Hz, 2H), 1.34 (t, *J* = 7.15 Hz, 3H). ^{13}C NMR (101 MHz, Chloroform-*d*) δ 164.8 (d, *J* = 11.95 Hz), 164.2 (t, *J* = 1.72 Hz), 162.0 (dd, *J* = 60.43, 11.87 Hz), 159.2 (d, *J* = 11.96 Hz), 142.8 (t, *J* = 25.78 Hz), 131.9 (dd, *J* = 9.83, 3.84 Hz), 128.8 – 127.3 (m), 111.6 (dd, *J* = 21.44, 3.80 Hz), 110.0 (dd, *J* = 353.08, 237.82 Hz), 104.7 (t, *J* = 26.25 Hz), 61.6, 14.2. ^{19}F NMR (377 MHz, Chloroform-*d*) δ -107.6 (d, *J* = 8.80 Hz), -109.2 (dt, *J* = 8.76, 7.22 Hz), -116.8 (d, *J* = 7.25 Hz). HRMS-ESI: Found $[M+Na]^+$ = 285.0516; $C_{12}H_{10}F_4O_2Na$ requires 285.0509.

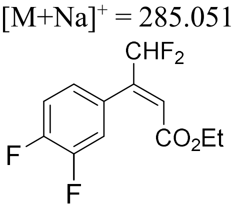

Ethyl (*E*)-3-(3,4-difluorophenyl)-4,4-difluorobut-2-enoate (1l)

Colorless oil. ^{1}H NMR (400 MHz, Chloroform-*d*) δ 7.66 (t, *J* = 55.00 Hz, 1H), 7.46 – 7.34 (m, 1H), 7.31 – 7.26 (m, 1H), 7.23 – 7.14 (m, 1H), 6.26 (s, 1H), 4.27 (q, *J* = 7.15 Hz, 2H), 1.34 (t, *J* = 7.14 Hz, 3H). ^{13}C NMR (101 MHz, Chloroform-*d*) δ 164.3, 152.1 (dd, *J* = 127.47, 12.48 Hz), 149.6 (dd, *J* = 124.19, 12.48 Hz), 147.0 (t, *J* = 23.72 Hz), 130.8, 125.7 (t, *J* = 7.39 Hz),

125.1 – 124.4 (m), 117.8, 117.6, 110.4 (t, J = 236.89 Hz), 61.6, 14.2.^{19}F NMR (377 MHz, Chloroform-d) δ -116.4, -135.4 (d, J = 21.22 Hz), -136.6 (d, J = 21.30 Hz). HRMS-ESI: Found [M+Na]$^+$ = 285.0511; $C_{12}H_{10}F_4O_2Na$ requires 285.0509.

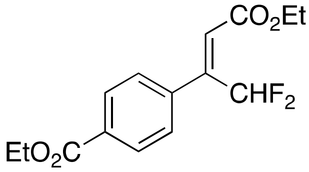

Ethyl (*Z*)-4-(4-ethoxy-1,1-difluoro-4-oxobut-2-en-2-yl)benzoate (*Z*-1m)

Colorless oil. ^{1}H NMR (400 MHz, Chloroform-d) δ 8.14 – 8.03 (m, 2H), 7.62 (d, J = 54.6 Hz, 1H), 7.60 (d, J = 8.2 Hz, 2H), 6.32 (s, 1H), 4.40 (q, J = 7.2 Hz, 2H), 4.28 (q, J = 7.2 Hz, 2H), 1.40 (t, J = 7.1 Hz, 3H), 1.35 (t, J = 7.2 Hz, 3H). ^{13}C NMR (101 MHz, Chloroform-d) δ 166.1, 164.4, 148.3 (t, J = 23.4 Hz), 138.3, 131.7, 129.8, 128.3, 126.0 (t, J = 7.8 Hz), 110.5 (t, J = 237.1 Hz), 61.6, 61.4, 14.5, 14.3. ^{19}F NMR (377 MHz, Chloroform-d) δ -116.1. HRMS-ESI: Found [M+Na]$^+$ = 321.0905; $C_{15}H_{16}F_2O_4Na$ requires 321.0909.

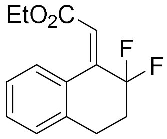

Ethyl (*E*)-2-(2,2-difluoro-3,4-dihydronaphthalen-1(2H)-ylidene)acetate (1n)

White solid. ^{1}H NMR (400 MHz, Chloroform-d) δ 7.56 (d, J = 7.96 Hz, 1H), 7.31 (td, J = 7.52, 1.30 Hz, 1H), 7.23 – 7.15 (m, 2H), 6.39 (t, J = 2.03 Hz, 1H), 4.24 (q, J = 7.14 Hz, 2H), 3.05 (t, J = 6.82 Hz, 2H), 2.39 (tt, J = 14.05, 6.83 Hz, 2H), 1.28 (t, J = 7.14 Hz, 3H). ^{13}C NMR (101 MHz, Chloroform-d) δ 167.1, 142.2 (t, J = 20.31 Hz), 136.8, 130.2, 129.9, 129.8 (t, J = 2.12 Hz), 128.4, 126.0, 119.8, 116.6 (t, J = 7.98 Hz), 61.1, 32.3 (t, J = 25.36 Hz), 26.4 (t, J = 5.33 Hz), 14.2. ^{19}F NMR (377 MHz, Chloroform-d) δ -99.0. HRMS-ESI: Found [M+Na]$^+$ = 275.0860; $C_{14}H_{14}F_2O_2Na$ requires 275.0854.

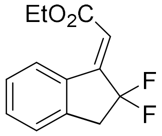

Ethyl (*E*)-2-(2,2-difluoro-2,3-dihydro-1H-inden-1-ylidene)acetate (1o)

Yellow oil. ^{1}H NMR (400 MHz, Chloroform-d) δ 8.73 (d, J = 7.45 Hz, 1H), 7.45 – 7.28 (m, 3H), 6.33 (t, J = 2.22 Hz, 1H), 4.29 (q, J = 7.11 Hz, 2H), 3.48 (t, J = 13.72 Hz, 2H), 1.36 (t, J = 7.12 Hz, 3H). ^{13}C NMR (101 MHz, Chloroform-d) δ 166.0 (t, J = 1.87 Hz), 149.7 (t, J = 21.62 Hz), 141.2 (t, J = 5.45 Hz), 133.9 (t, J = 2.78 Hz), 132.1, 129.2, 128.1, 126.1 (t, J = 250.07 Hz), 125.1 (t, J = 1.98 Hz), 115.9 (t, J = 3.27 Hz), 60.9, 40.6 (t, J = 26.27 Hz), 14.4. ^{19}F NMR (377 MHz, Chloroform-d) δ -94.6. HRMS-ESI: Found [M+Na]$^+$ = 261.0702; $C_{13}H_{12}F_2O_2Na$ requires 261.0698.

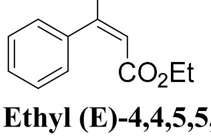

Ethyl (E)-4,4,5,5,5-pentafluoro-3-phenylpent-2-enoate (1q)

Colorless oil. ^{1}H NMR (400 MHz, Chloroform-d) δ 7.49 – 7.34 (m, 3H), 7.28 (d, J = 6.62 Hz, 2H), 6.68 (s, 1H), 4.04 (q, J = 7.14 Hz, 2H), 1.04 (t, J = 7.15 Hz, 3H). ^{13}C NMR (101 MHz, Chloroform-d) δ 164.0, 141.8 (t, J = 21.54 Hz), 131.4, 129.3, 129.0, 128.1, 127.8 (t, J = 8.36 Hz), 118.9 (qt, J = 287.00, 37.74 Hz), 115.6 – 108.9 (m), 61.1, 13.6. ^{19}F NMR (377 MHz, Chloroform-d) δ -82.0 (t, J = 2.02 Hz), -115.0 (q, J = 2.32 Hz). HRMS-ESI: Found [M+Na]$^+$ = 317.0566; $C_{13}H_{11}F_5O_2Na$ requires 317.0571.

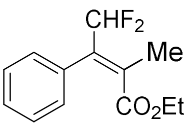

Ethyl (*E*)-4,4-difluoro-2-methyl-3-phenyl-4l3-but-2-enoate (1r)

Colorless oil. ^{1}H NMR (400 MHz, Chloroform-*d*) δ 7.44 – 7.36 (m, 3H), 7.24 – 7.19 (m, 2H), 6.62 (t, *J* = 55.2 Hz, 1H), 3.88 (q, *J* = 7.1 Hz, 2H), 2.18 (t, *J* = 2.9 Hz, 3H), 0.83 (t, *J* = 7.1 Hz, 3H). ^{13}C NMR (101 MHz, Chloroform-*d*) δ 169.2, 136.1 (d, *J* = 37.4 Hz), 136.6 – 135.7 (m), 134.7, 129.2, 128.3, 128.1, 112.5 (t, *J* = 237.7 Hz), 61.1, 15.4, 13.6. ^{19}F NMR (377 MHz, Chloroform-*d*) δ -114.1 (s). HRMS-ESI: Found $[M+Na]^+$ = 263.0860; $C_{13}H_{14}F_2O_2Na$ requires 263.0854.

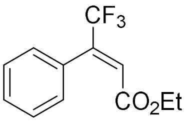

Ethyl (*E*)-4,4,4-trifluoro-3-phenylbut-2-enoate (1s)

Colorless oil. ^{1}H NMR (400 MHz, Chloroform-*d*) δ 7.46 – 7.35 (m, 3H), 7.32 – 7.26 (m, 2H), 6.61 (d, *J* = 1.36 Hz, 1H), 4.04 (q, *J* = 7.14 Hz, 2H), 1.06 (t, *J* = 7.14 Hz, 3H). ^{13}C NMR (101 MHz, Chloroform-*d*) δ 164.3, 142.5 (q, *J* = 30.58 Hz), 131.2, 129.4, 128.8, 128.3, 124.7 (q, *J* = 5.45 Hz), 124.0, 61.2, 13.9. ^{19}F NMR (377 MHz, Chloroform-*d*) δ -67.5. HRMS-ESI: Found $[M+Na]^+$ = 267.0612; $C_{12}H_{11}F_3O_2Na$ requires 267.0603.

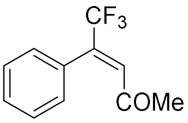

(*E*)-5,5,5-trifluoro-4-phenylpent-3-en-2-one (1t)

Colorless oil. ^{1}H NMR (400 MHz, Chloroform-*d*) δ 7.49 – 7.38 (m, 3H), 7.34 – 7.28 (m, 2H), 6.71 (q, *J* = 1.35 Hz, 1H), 1.90 (s, 3H). ^{13}C NMR (101 MHz, Chloroform-*d*) δ 199.4, 139.2 (q, *J* = 30.93 Hz), 132.6 (q, *J* = 4.90 Hz), 131.0, 130.0, 129.2, 128.9, 123.0 (q, *J* = 276.36 Hz), 30.6. ^{19}F NMR (376 MHz, Chloroform-*d*) δ -67.2. HRMS-ESI: Found $[M+Na]^+$ = 237.0486; $C_{11}H_9F_3ONa$ requires 237.0498.

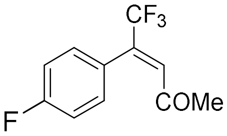

(*E*)-5,5,5-trifluoro-4-(4-fluorophenyl)pent-3-en-2-one (1u)

Colorless oil. ^{1}H NMR (400 MHz, Chloroform-*d*) δ 7.33 – 7.27 (m, 2H), 7.16 – 7.07 (m, 2H), 6.74 (q, *J* = 1.30 Hz, 1H), 1.97 (s, 3H). ^{13}C NMR (101 MHz, Chloroform-*d*) δ 198.9, 163.7 (d, *J* = 250.53 Hz), 138.1 (q, *J* = 31.06 Hz), 132.7 (q, *J* = 4.77 Hz), 126.8 (d, *J* = 3.62 Hz), 122.8 (d, *J* = 275.43 Hz), 116.2, 116.0, 30.8. ^{19}F NMR (376 MHz, Chloroform-*d*) δ -59.4, -111.3. HRMS-ESI: Found $[M+Na]^+$ = 255.0409; $C_{11}H_8F_4ONa$ requires 255.0403.

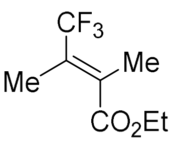

Ethyl (*E*)-4,4,4-trifluoro-2,3-dimethylbut-2-enoate (1v)

Colorless oil. ^{1}H NMR (400 MHz, Chloroform-*d*) δ 4.26 (q, *J* = 7.1 Hz, 2H), 2.05 (qd, *J* = 2.7, 1.4 Hz, 3H), 1.92 (q, *J* = 1.6 Hz, 3H), 1.33 (t, *J* = 7.1 Hz, 3H). ^{13}C NMR (Chloroform-*d*, 101 MHz) δ 169.1, 135.2 (t, *J* = 3.3 Hz), 128.5 (q, *J* = 29.7 Hz), 124.2 (d, *J* = 276.2 Hz), 61.5, 16.4 (q, *J* = 2.4 Hz), 15.3 (q, *J* = 3.0 Hz), 14.2.^{19}F NMR (Chloroform-*d*, 376 MHz) δ -61.6 (s). HRMS-ESI: Found $[M+Na]^+$ = 219.0606; $C_8H_{11}F_3O_2Na$ requires 219.0603.

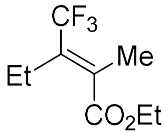

Ethyl (*E*)-2-methyl-3-(trifluoromethyl)pent-2-enoate (1w)

Colorless oil. ^{1}H NMR (400 MHz, Chloroform-*d*) δ 4.26 (q, *J* = 7.2 Hz, 2H), 2.37 – 2.20 (m, 2H), 2.04 (dtd, *J* = 3.5, 2.6, 0.9 Hz, 3H), 1.33 (t, *J* = 7.1 Hz, 3H), 1.16 – 1.02 (m, 3H). ^{13}C NMR (Chloroform-*d*, 101 MHz) δ 169.0, 135.6 (q, *J* = 3.5 Hz), 133.9 (q, *J* = 28.0 Hz), 124.4 (q, *J* = 277.0 Hz), 61.4 (d, *J* = 2.6 Hz), 23.5 (q, *J* = 2.6 Hz), 16.4 (d, *J* = 3.6 Hz), 14.1 (d, *J* = 3.7 Hz), 13.7 (d, *J* = 3.5 Hz). ^{19}F NMR (Chloroform-*d*, 376 MHz) δ -59.8 (s). HRMS-ESI: Found $[M+Na]^+$ = 233.0738; $C_9H_{13}F_3O_2Na$ requires 233.0760.

4. General procedure for synthesis of monofluoroalkenes

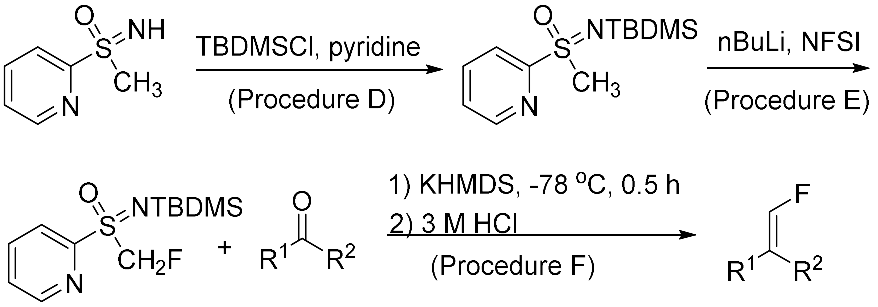

The corresponding monofluoroalkenes were prepared following procedure D-F, which was carried out according to the literature.[3]

New compounds:

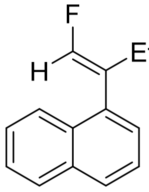

(E)-1-(1-fluorobut-1-en-2-yl)naphthalene (3b)

Colorless oil. ^{1}H NMR (400 MHz, Chloroform-*d*) δ 8.02 – 7.94 (m, 1H), 7.91 – 7.85 (m, 1H), 7.84 – 7.80 (m, *J* = 8.26, 1.10 Hz, 1H), 7.55 – 7.47 (m, 2H), 7.45 (dd, *J* = 8.25, 6.94 Hz, 1H), 7.30 (dd, *J* = 6.95, 1.31 Hz, 1H), 6.62 (d, *J* = 86.16 Hz, 1H), 2.65 (qdd, *J* = 7.47, 2.67, 0.97 Hz, 2H), 0.97 (t, *J* = 7.55 Hz, 3H). ^{13}C NMR (101 MHz, Chloroform-*d*) δ 146.2 (d, *J* = 261.20 Hz), 134.2 (d, *J* = 10.17 Hz), 133.9, 132.7 (d, *J* = 2.87 Hz), 128.5, 128.2, 127.5 (d, *J* = 2.94 Hz), 126.2, 126.0, 125.7, 125.3, 125.2 (d, *J* = 9.84 Hz), 22.5 (d, *J* = 3.64 Hz), 12.6 (d, *J* = 2.42 Hz). ^{19}F NMR (377 MHz, Chloroform-*d*) δ -129.9. HRMS-ESI: Found $[M+Na]^+$ = 223.0881; $C_{14}H_{13}FNa$ requires 223.0893.

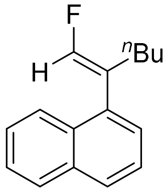

(*E*)-1-(1-fluorohex-1-en-2-yl)naphthalene (3c)

Colorless oil. ^{1}H NMR (400 MHz, Chloroform-*d*) δ 8.03 – 7.94 (m, 1H), 7.91 – 7.84 (m, 1H), 7.81 (dt, *J* = 8.23, 1.07 Hz, 1H), 7.56 – 7.47 (m, 2H), 7.44 (dd, *J* = 8.28, 7.00 Hz, 1H), 7.29 (dd, *J* = 7.01, 1.28 Hz, 1H), 6.64 (d, *J* = 86.36 Hz, 1H), 2.61 (dddd, *J* = 7.48, 5.60, 4.01, 1.81 Hz, 2H), 1.40 – 1.24 (m, 4H), 0.85 (t, *J* = 6.96 Hz, 3H). ^{13}C NMR (101 MHz, Chloroform-*d*) δ

146.6 (d, *J* = 261.19 Hz), 134.6 (d, *J* = 10.23 Hz), 133.9, 132.7 (d, *J* = 2.87 Hz), 128.5, 128.1, 127.4 (d, *J* = 2.97 Hz), 126.2, 126.0, 125.7, 125.3, 123.9 (d, *J* = 8.77 Hz), 30.0 (d, *J* = 2.30 Hz), 29.0 (d, *J* = 2.93 Hz), 22.6, 14.0. ^{19}F NMR (376 MHz, Chloroform-*d*) δ -129.4. HRMS-ESI: Found $[M+Na]^+$ = 251.1216; $C_{16}H_{17}FNa$ requires 251.1206.

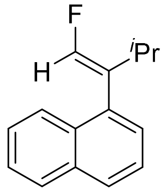

(*E*)-1-(1-fluoro-3-methylbut-1-en-2-yl)naphthalene (3d)

Colorless oil. ^{1}H NMR (400 MHz, Chloroform-*d*) δ 8.08 – 7.98 (m, 1H), 7.88 – 7.84 (m, 1H), 7.82 (dt, *J* = 8.26, 1.15 Hz, 1H), 7.54 – 7.46 (m, 2H), 7.44 (dd, *J* = 8.27, 7.03 Hz, 1H), 7.29 (dd, *J* = 7.05, 1.31 Hz, 1H), 6.49 (d, *J* = 85.98 Hz, 1H), 3.18 (hept, *J* = 6.98 Hz, 1H), 1.39 – 0.77 (m, 6H). ^{13}C NMR (101 MHz, Chloroform-*d*) δ 146.3 (d, *J* = 263.26 Hz), 133.8, 133.7 (d, *J* = 2.60 Hz), 133.4 (d, *J* = 11.00 Hz), 128.4 (d, *J* = 3.23 Hz), 128.3, 128.1, 128.1 (d, *J* = 6.05 Hz), 126.4, 126.0, 125.9, 125.0, 29.1 (d, *J* = 1.80 Hz), 21.4. ^{19}F NMR (377 MHz, Chloroform-*d*) δ -128.3. HRMS-ESI: Found $[M+Na]^+$ = 237.1064; $C_{15}H_{15}FNa$ requires 237.1050.

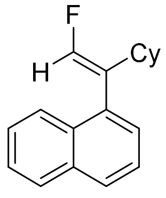

(*E*)-1-(1-cyclohexyl-2-fluorovinyl)naphthalene (3e)

Colorless oil. ^{1}H NMR (400 MHz, Chloroform-*d*) δ 8.09 – 7.99 (m, 1H), 7.90 – 7.78 (m, 2H), 7.55 – 7.40 (m, 3H), 7.32 – 7.27 (m, 1H), 6.49 (d, *J* = 86.33 Hz, 1H), 2.84 (tt, *J* = 11.99, 3.48 Hz, 1H), 2.02 – 1.56 (m, 6H), 1.40 – 1.22 (m, 3H), 1.12 – 1.00 (m, 1H). ^{13}C NMR (101 MHz, Chloroform-*d*) δ 146.3 (d, *J* = 263.14 Hz), 133.7 (d, *J* = 5.47 Hz), 133.7, 133.6, 128.3, 128.3, 128.0, 127.5 (d, *J* = 6.43 Hz), 126.5, 125.9 (d, *J* = 14.85 Hz), 125.3, 125.0, 39.3, 31.4, 26.7, 26.0. ^{19}F NMR (377 MHz, Chloroform-*d*) δ -128.5. HRMS-ESI: Found $[M+Na]^+$ = 277.1373, $C_{18}H_{19}FNa$ requires 277.1363.

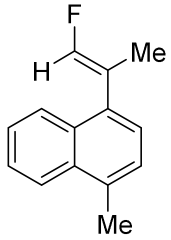

(*E*)-1-(1-fluoroprop-1-en-2-yl)-4-methylnaphthalene (3f)

Colorless oil. ^{1}H NMR (400 MHz, Chloroform-*d*) δ 8.08 – 7.91 (m, 2H), 7.59 – 7.46 (m, 2H), 7.28 (dd, *J* = 7.09, 1.03 Hz, 1H), 7.21 (d, *J* = 7.08 Hz, 1H), 6.67 (dq, *J* = 86.10, 1.59 Hz, 1H), 2.70 (d, *J* = 0.94 Hz, 3H), 2.12 (dd, *J* = 3.63, 1.59 Hz, 3H). ^{13}C NMR (101 MHz, Chloroform-*d*) δ 146.8 (d, *J* = 260.75 Hz), 134.5, 134.2 (d, *J* = 9.89 Hz), 133.0, 132.2 (d, *J* = 2.96 Hz), 126.4 (d, *J* = 2.99 Hz), 126.2, 126.1, 125.9, 125.9, 124.7, 119.4 (d, *J* = 9.25 Hz), 19.6, 15.7 (d, *J* = 4.17 Hz). ^{19}F NMR (377 MHz, Chloroform-*d*) δ -128.8. HRMS-ESI: Found $[M+Na]^+$ = 223.0876; $C_{14}H_{13}FNa$ requires 223.0893.

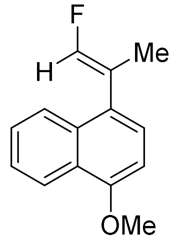

(*E*)-1-(1-fluoroprop-1-en-2-yl)-4-methoxynaphthalene (3g)

Colorless oil. ^{1}H NMR (400 MHz, Chloroform-*d*) δ 8.35 – 8.23 (m, 1H), 7.94 – 7.83 (m, 1H), 7.58 – 7.43 (m, 2H), 7.21 (d, *J* = 7.82 Hz, 1H), 6.76 (d, *J* = 7.78 Hz, 1H), 6.66 (dq, *J* = 85.91, 1.58 Hz, 1H), 4.01 (s, 3H), 2.09 (dd, *J* = 3.66, 1.58 Hz, 3H). ^{13}C NMR (101 MHz, Chloroform-*d*) δ 155.4, 146.8 (d, *J* = 260.80 Hz), 133.1 (d, *J* = 2.98 Hz), 128.1 (d, *J* = 10.27 Hz), 126.8, 126.7 (d, *J* = 2.99 Hz), 125.9, 125.3, 125.3, 122.5, 119.2 (d, *J* = 9.29 Hz), 103.3, 55.7, 15.7 (d, *J* = 4.09 Hz). ^{19}F NMR (377 MHz, Chloroform-*d*) δ -128.7. HRMS-ESI: Found $[M+Na]^+$ = 239.0855; $C_{14}H_{13}FONa$ requires 239.0843.

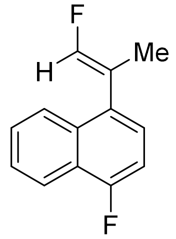

(*E*)-1-fluoro-4-(1-fluoroprop-1-en-2-yl)naphthalene (3h)

Colorless oil. ^{1}H NMR (400 MHz, Chloroform-*d*) δ 8.18 – 8.09 (m, 1H), 7.99 – 7.90 (m, 1H), 7.62 – 7.51 (m, 2H), 7.27 – 7.19 (m, 1H), 7.10 (dd, *J* = 10.32, 7.83 Hz, 1H), 6.66 (dq, *J* = 85.60, 1.55 Hz, 1H), 2.10 (dd, *J* = 3.65, 1.58 Hz, 3H). ^{13}C NMR (101 MHz, Chloroform-*d*) δ 158.6 (d, *J* = 252.24 Hz), 147.0 (d, *J* = 262.54 Hz), 133.5 (dd, *J* = 4.75, 3.02 Hz), 131.9 (dd, *J* = 10.15, 4.48 Hz), 127.2, 126.5 (dd, *J* = 8.28, 3.11 Hz), 126.3 (d, *J* = 1.89 Hz), 125.5 (d, *J* = 2.78 Hz), 124.1 (d, *J* = 16.40 Hz), 121.1 (d, *J* = 5.55 Hz), 118.8 (d, *J* = 9.83 Hz), 109.0 (d, *J* = 19.94 Hz), 15.6 (d, *J* = 4.01 Hz). ^{19}F NMR (377 MHz, Chloroform-*d*) δ -123.4, -127.8. HRMS-ESI: Found $[M+Na]^+$ = 227.0654; $C_{13}H_{10}F_2Na$ requires 227.0643.

5. General procedure for asymmetric hydrogenation

A vial was charged with substrate (0.15 mmol) and Ir-complex (1 mol%). Dry CH_2Cl_2 (1.5 ml) was added (so that the concentration of the substrate was 0.1 M) and the vial was placed in a high-pressure hydrogenation apparatus. The reactor was purged three times with Ar gas, then filled with H_2. The reaction was stirred at room temperature for 4-24 hours before the H_2 pressure was released and the solvent was removed *in vacuo*. The crude product was filtered through on a short plug of silica. Conversions were determined by ^{1}H NMR spectroscopy and *ee* values were determined by HPLC, SFC or GCMS using a chiral stationary phase.

The full characterizations of the new hydrogenated products are available below (in some specific cases the hydrogenated products are characterized in the mixture with de-fluorinated product).

The characterization of the hydrogenated compounds:

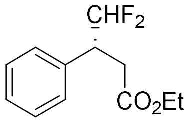

Ethyl *(R)*-4,4-difluoro-3-phenylbutanoate (2a)

Colorless oil. ^{1}H NMR (400 MHz, Chloroform-*d*) δ 7.38 – 7.26 (m, 5H), 5.94 (td, *J* = 56.34, 3.39 Hz, 1H), 4.07 (qd, *J* = 7.13, 3.92 Hz, 2H), 3.69 – 3.57 (m, 1H), 2.96 (dd, *J* = 16.21, 5.92 Hz, 1H), 2.78 (dd, *J* = 16.24, 8.95 Hz, 1H), 1.15 (t, *J* = 7.11 Hz, 3H). ^{13}C NMR (101 MHz, Chloroform-*d*) δ 171.1, 135.9 (dd, *J* = 5.26, 2.62 Hz), 128.9, 128.9, 128.1, 117.1 (t, *J* = 244.93 Hz), 60.9, 46.0 (t, *J* = 20.36 Hz), 33.7 (dd, *J* = 5.18, 3.68 Hz), 14.2. ^{19}F NMR (377 MHz, Chloroform-*d*) δ -119.5 (d, *J* = 278.09 Hz), -123.6 (d, *J* = 278.11 Hz). HRMS-ESI: Found $[M+Na]^+$ = 251.0858; $C_{12}H_{14}F_2O_2Na$ requires 251.0854.

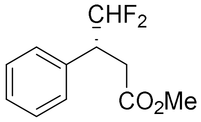

Methyl *(R)*-4,4-difluoro-3-phenylbutanoate (2b)

Colorless oil. ^{1}H NMR (400 MHz, Chloroform-*d*) δ 7.38 – 7.26 (m, 5H), 5.94 (td, *J* = 56.34, 3.37 Hz, 1H), 3.68 – 3.56 (m, 1H), 3.62 (s, 3H), 2.98 (dd, *J* = 16.37, 5.90 Hz, 1H), 2.79 (dd, *J* = 16.36, 8.87 Hz, 1H). ^{13}C NMR (101 MHz, Chloroform-*d*) δ 171.6, 135.9 (dd, *J* = 5.34, 2.36 Hz), 128.9, 128.8, 128.2, 117.1 (t, *J* = 244.93 Hz), 52.1, 46.0 (t, *J* = 20.18 Hz), 33.4 (dd, *J* = 5.18, 3.79 Hz). ^{19}F NMR (377 MHz, Chloroform-*d*) δ -119.4 (d, *J* = 278.11 Hz), -123.7 (d, *J* = 278.14 Hz). HRMS-ESI: Found $[M+Na]^+$ = 237.0704; $C_{11}H_{12}F_2O_2Na$ requires 237.0698.

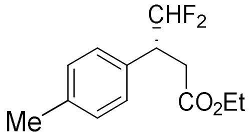

***(R)*-5,5-difluoro-4-(p-tolyl)pentan-2-one (2c)**

Colorless oil. ^{1}H NMR (400 MHz, Chloroform-*d*) δ 7.18-7.14 (m, 4H), 5.91 (td, *J* = 56.45, 3.42 Hz, 1H), 4.19 – 3.97 (m, 2H), 3.67 – 3.50 (m, 1H), 2.94 (dd, *J* = 16.20, 5.89 Hz, 1H), 2.75 (dd, *J* = 16.19, 9.01 Hz, 1H), 2.33 (s, 3H), 1.17 (t, *J* = 7.14 Hz, 3H). ^{13}C NMR (101 MHz, Chloroform-*d*) δ 171.2, 137.8, 132.8 (dd, *J* = 5.02, 2.84 Hz), 129.6, 128.7, 117.2 (t, *J* = 244.83 Hz), 60.9, 45.6 (t, *J* = 20.32 Hz), 33.7 (dd, *J* = 5.01, 3.74 Hz), 21.2, 14.2. ^{19}F NMR (377 MHz, Chloroform-*d*) δ -119.4 (d, *J* = 277.59 Hz), -123.6 (d, *J* = 277.63 Hz). HRMS-ESI: Found $[M+Na]^+$ = 265.1017; $C_{13}H_{16}F_2O_2Na$ requires 265.1011.

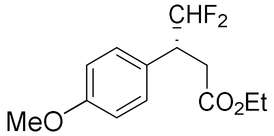

Ethyl (*S*)-4,4-difluoro-3-(4-methoxyphenyl)butanoate (2d)

Colorless oil. ^{1}H NMR (400 MHz, Chloroform-*d*) δ 7.24 – 7.16 (m, 2H), 6.92 – 6.83 (m, 2H), 5.89 (td, *J* = 56.49, 3.36 Hz, 1H), 4.15 – 3.98 (m, 2H), 3.79 (s, 3H), 3.66 – 3.49 (m, 1H), 2.92 (dd, *J* = 16.15, 5.84 Hz, 1H), 2.73 (dd, *J* = 16.10, 9.14 Hz, 1H), 1.16 (t, *J* = 7.13 Hz, 3H). ^{13}C NMR (101 MHz, Chloroform-*d*) δ 171.2, 159.4, 129.9, 127.8 (dd, *J* = 5.52, 2.45 Hz), 117.2 (t, *J* = 244.82 Hz), 114.3, 60.9, 55.4, 45.2 (t, *J* = 20.33 Hz), 33.8 (dd, *J* = 5.06, 3.60 Hz), 14.2. ^{19}F NMR (377 MHz, Chloroform-*d*) δ -119.6 (d, *J* = 277.27 Hz), -123.7 (d, *J* = 277.45 Hz). HRMS-ESI: Found $[M+Na]^+$ = 281.0975; $C_{13}H_{16}F_2O_3Na$ requires 281.0960.

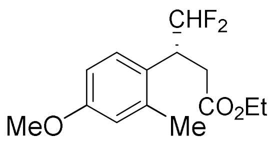

Ethyl *(R)*-4,4-difluoro-3-(4-methoxy-2-methylphenyl)butanoate (2e)

Colorless oil. ^{1}H NMR (400 MHz, Chloroform-*d*) δ 7.10 – 7.01 (m, 2H), 6.78 (d, *J* = 8.30 Hz, 1H), 5.89 (td, *J* = 56.55, 3.42 Hz, 1H), 4.15 – 3.99 (m, 2H), 3.81 (s, 3H), 3.58 – 3.48 (m, 1H), 2.91 (dd, *J* = 16.12, 5.87 Hz, 1H), 2.73 (dd, *J* = 16.11, 9.02 Hz, 1H), 2.20 (s, 3H), 1.17 (t, *J* = 7.14 Hz, 3H). ^{13}C NMR (101 MHz, Chloroform-*d*) δ 171.3, 157.6, 131.0, 127.4 (dd, *J* = 5.47, 2.72 Hz), 127.1, 127.0, 117.3 (t, *J* = 244.80 Hz), 110.1, 60.9, 55.4, 45.2 (t, *J* = 20.30 Hz), 33.8 (dd, *J* = 5.07, 3.59 Hz), 16.4, 14.2. ^{19}F NMR (377 MHz, Chloroform-*d*) δ -119.3 (d, *J* = 276.64 Hz), -123.7 (d, *J* = 276.68 Hz). HRMS-ESI: Found [M+Na]$^+$ = 295.1104; $C_{14}H_{18}F_2O_3Na$ requires 295.1116.

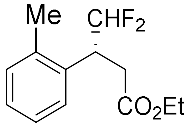

Ethyl (*R*)-4,4-difluoro-3-(*o*-tolyl)butanoate (2f)

Colorless oil. ^{1}H NMR (400 MHz, Chloroform-*d*) δ 7.24 – 7.16 (m, 4H), 5.88 (td, J = 56.5, 3.9 Hz, 1H), 4.10 – 3.91 (m, 3H), 2.96 (dd, J = 16.3, 5.9 Hz, 1H), 2.79 (dd, J = 16.3, 9.1 Hz, 1H), 2.42 (s, 3H), 1.14 (t, J = 7.1 Hz, 3H). ^{13}C NMR (101 MHz, Chloroform-*d*) δ 171.2, 137.5, 134.3 (dd, J = 4.8, 3.0 Hz), 130.8, 127.8, 127.2, 126.5, 117.3 (t, J = 244.9 Hz), 60.9, 40.8 (d, J = 20.5 Hz), 33.9 (t, J = 4.6 Hz), 20.1, 14.1. ^{19}F NMR (377 MHz, Chloroform-*d*) δ -119.3 (d, J = 277.2 Hz), -122.9 (d, J = 277.2 Hz). HRMS-ESI: Found [M+Na]$^+$ = 265.1012; $C_{13}H_{16}F_2O_2Na$ requires 265.1011.

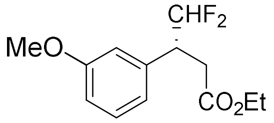

Ethyl (*R*)-4,4-difluoro-3-(3-methoxyphenyl)butanoate (2g)

Colorless oil. ^{1}H NMR (400 MHz, Chloroform-*d*) δ 7.30 – 7.22 (m, 1H), 6.90 – 6.80 (m, 3H), 5.93 (td, *J* = 56.4, 3.4 Hz, 1H), 4.08 (qd, *J* = 7.1, 3.9 Hz, 2H), 3.80 (s, 3H), 3.67 – 3.53 (m, 1H), 2.94 (dd, *J* = 16.3, 5.9 Hz, 1H), 2.76 (dd, *J* = 16.3, 8.9 Hz, 1H), 1.17 (t, *J* = 7.1 Hz, 3H). ^{13}C NMR (101 MHz, Chloroform-*d*) δ 171.1, 159.9, 137.4 (dd, *J* = 5.3, 2.6 Hz), 129.9, 121.1, 117.1 (t, *J* = 245.0 Hz), 114.8, 113.3, 61.0, 55.3, 46.0 (t, *J* = 20.4 Hz), 33.6 (dd, *J* = 4.9, 3.8 Hz), 14.2. ^{19}F NMR (377 MHz, Chloroform-*d*) δ -119.2 (d, *J* = 277.9 Hz), -123.5 (d, *J* = 277.8 Hz). HRMS-ESI: Found [M+Na]$^+$ = 281.0959; $C_{13}H_{16}F_2O_3Na$ requires 281.0960.

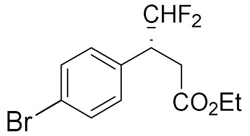

Ethyl (*R*)-3-(4-bromophenyl)-4,4-difluorobutanoate (2h)

Colorless oil. ^{1}H NMR (400 MHz, Chloroform-*d*) δ 7.38 – 7.27 (m, 4H), 5.94 (td, *J* = 56.3, 3.4 Hz, 1H), 4.07 (qd, *J* = 7.1, 4.0 Hz, 2H), 3.70 – 3.55 (m, 1H), 2.96 (dd, *J* = 16.2, 5.9 Hz, 1H), 2.78 (dd, *J* = 16.2, 9.0 Hz, 1H), 1.15 (t, *J* = 7.1 Hz, 3H). ^{13}C NMR (101 MHz, Chloroform-*d*) δ 171.1, 135.9 (dd, *J* = 5.4, 2.6 Hz), 128.9, 128.1, 117.1 (t, *J* = 245.3 Hz), 60.9, 46.0 (t, *J* = 20.4 Hz), 33.7 (dd, *J* = 5.1, 4.2 Hz), 14.2. ^{19}F NMR (377 MHz, Chloroform-*d*) δ -119.5 (d, *J* = 278.1 Hz), -123.5 (d, *J* = 278.4 Hz). HRMS-ESI: Found [M+Na]$^+$ = 328.9966; $C_{12}H_{13}F_2BrO_2Na$ requires 328.9959 (Br^{79}).

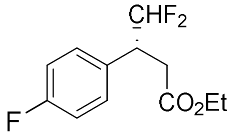

Ethyl (*R*)-4,4-difluoro-3-(4-fluorophenyl)butanoate (2i)

Colorless oil. ^{1}H NMR (400 MHz, Chloroform-*d*) δ 7.30 – 7.17 (m, 2H), 7.10 – 6.98 (m, 2H), 5.91 (td, *J* = 56.29, 3.25 Hz, 1H), 4.07 (qd, *J* = 7.15, 3.83 Hz, 2H), 3.67 – 3.55 (m, 1H), 2.94 (dd, *J* = 16.26, 5.77 Hz, 1H), 2.74 (dd, *J* = 16.24, 9.20 Hz, 1H), 1.16 (t, *J* = 7.14 Hz, 3H). ^{13}C NMR (101 MHz, Chloroform-*d*) δ 171.0, 162.6 (d, *J* = 246.69 Hz), 131.8 – 131.3 (m), 130.6 (d, *J* = 8.11 Hz), 116.8 (t, *J* = 244.90 Hz), 115.8 (d, *J* = 21.38 Hz), 61.0, 45.3 (t, *J* = 20.50 Hz), 33.8 (dd, *J* = 5.27, 3.57 Hz), 14.2. ^{19}F NMR (377 MHz, Chloroform-*d*) δ -114.3, -120.1 (d, *J* = 278.54 Hz), -123.4 (d, *J* = 278.79 Hz). HRMS-ESI: Found $[M+Na]^+$ = 269.0766; $C_{12}H_{13}F_3O_2Na$ requires 269.0760

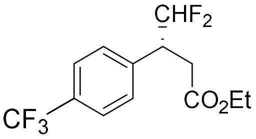

Ethyl (*R*)-4,4-difluoro-3-(4-(trifluoromethyl)phenyl)butanoate (2j)

Colorless oil. ^{1}H NMR (400 MHz, Chloroform-*d*) δ 7.61 (d, *J* = 8.15 Hz, 2H), 7.42 (d, *J* = 8.01 Hz, 2H), 5.96 (td, *J* = 56.12, 3.26 Hz, 1H), 4.08 (qd, *J* = 7.12, 4.37 Hz, 2H), 3.79 – 3.65 (m, 1H), 2.98 (dd, *J* = 16.48, 5.78 Hz, 1H), 2.79 (dd, *J* = 16.49, 9.10 Hz, 1H), 1.17 (t, *J* = 7.14 Hz, 3H). ^{13}C NMR (101 MHz, Chloroform-*d*) δ 170.7, 139.9, 130.5 (q, *J* = 32.62 Hz), 129.4, 125.8 (q, *J* = 3.78 Hz), 124.1 (q, *J* = 268.36 Hz), 116.5 (t, *J* = 245.05 Hz), 61.2, 45.8 (t, *J* = 20.62 Hz), 33.6 (dd, *J* = 5.24, 3.62 Hz), 14.1. ^{19}F NMR (377 MHz, Chloroform-*d*) δ -62.8, -120.5 (d, *J* = 280.50 Hz), -122.8 (d, *J* = 280.48 Hz). HRMS-ESI: Found $[M+Na]^+$ = 319.0734; $C_{13}H_{13}F_5O_2Na$ requires 319.0728

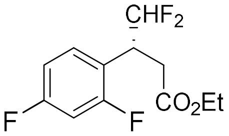

Ethyl *(R)*-3-(2,4-difluorophenyl)-4,4-difluorobutanoate (2k)

Colorless oil. ^{1}H NMR (400 MHz, Chloroform-*d*) δ 7.32 – 7.22 (m, 1H), 6.93 – 6.79 (m, 2H), 5.98 (tdd, *J* = 56.37, 3.90, 0.73 Hz, 1H), 4.08 (qd, *J* = 7.16, 4.32 Hz, 2H), 3.99 – 3.88 (m, 1H), 2.95 (dd, *J* = 16.37, 5.91 Hz, 1H), 2.78 (dd, *J* = 16.37, 9.20 Hz, 1H), 1.17 (t, *J* = 7.12 Hz, 3H). ^{13}C NMR (101 MHz, Chloroform-*d*) δ 170.7, 163.2 (dd, *J* = 145.91, 12.07 Hz), 160.7 (dd, *J* = 145.84, 12.22 Hz), 131.0 (dd, *J* = 9.70, 5.42 Hz), 119.2 – 118.6 (m), 116.0 (t, *J* = 244.64 Hz), 111.8 (dd, *J* = 21.26, 3.71 Hz), 104.3 (dd, *J* = 26.64, 25.19 Hz), 61.1, 39.1 (dd, *J* = 21.84, 0.98 Hz), 32.9 (d, *J* = 3.79 Hz), 14.2. ^{19}F NMR (377 MHz, Chloroform-*d*) δ -110.2 (d, *J* = 8.09 Hz), -112.5 (dt, *J* = 7.48, 3.52 Hz), -120.1 (dd, *J* = 279.82, 3.05 Hz), -123.0 (dd, *J* = 279.54, 3.70 Hz). HRMS-ESI: Found $[M+Na]^+$ = 287.0668; $C_{12}H_{12}F_4O_2Na$ requires 287.0666

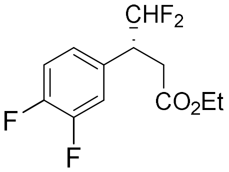

Ethyl *(R)*-3-(3,4-difluorophenyl)-4,4-difluorobutanoate (2l)

Colorless oil. ^{1}H NMR (400 MHz, Chloroform-*d*) δ 7.20 – 7.08 (m, 2H), 7.07 – 6.97 (m, 1H), 5.91 (td, *J* = 56.16, 3.12 Hz, 1H), 4.17 – 4.00 (m, 2H), 3.68 – 3.51 (m, 1H), 2.92 (dd, *J* = 16.44, 5.70 Hz, 1H), 2.72 (dd, *J* = 16.45, 9.22 Hz, 1H), 1.18 (t, *J* = 7.15 Hz, 3H). ^{13}C NMR (101 MHz, Chloroform-*d*) δ 170.7, 151.6 (dd, *J* = 21.17, 12.48 Hz), 149.1 (dd, *J* = 12.13, 8.57 Hz), 132.7 (dd, *J* = 9.51, 5.59 Hz), 126.2 – 122.6 (m), 118.0 (d, *J* = 17.64 Hz), 117.7 (d, *J* = 17.28 Hz), 116.4 (t, *J* = 245.14 Hz), 61.2 (d, *J* = 5.35 Hz), 45.2 (t, *J* = 20.42 Hz), 33.7 (dd, *J* = 5.16, 3.52 Hz), 14.2. ^{19}F NMR (377 MHz, Chloroform-*d*) δ -120.7 (d, *J* = 280.16 Hz), -123.1 (d, *J* = 280.17 Hz), -136.8 (d, *J* = 21.48 Hz), -138.6 (d, *J* = 21.19 Hz). HRMS-ESI: Found $[M+Na]^+$ = 287.0671; $C_{12}H_{12}F_4O_2Na$ requires 287.0666

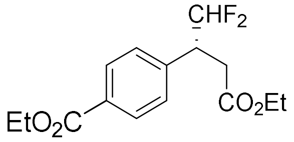

Ethyl (*R*)-4-(4-ethoxy-1,1-difluoro-4-oxobutan-2-yl)benzoate (2m)

Colorless oil. ^{1}H NMR (400 MHz, Chloroform-*d*) δ 8.02 (dd, *J* = 8.4, 2.0 Hz, 2H), 7.36 (d, *J* = 8.3 Hz, 2H), 5.95 (td, *J* = 56.2, 3.4 Hz, 1H), 4.37 (q, *J* = 7.1 Hz, 2H), 4.06 (qd, *J* = 7.1, 4.8 Hz, 2H), 3.77 – 3.63 (m, 1H), 2.97 (dd, *J* = 16.4, 5.8 Hz, 1H), 2.79 (dd, *J* = 16.4, 9.1 Hz, 1H), 1.39 (t, *J* = 7.1 Hz, 3H), 1.16 (t, *J* = 7.1 Hz, 3H). ^{13}C NMR (101 MHz, Chloroform-*d*) δ 170.8, 166.3, 140.8 (dd, *J* = 4.8, 2.5 Hz), 130.1, 130.1, 129.0, 116.6 (t, *J* = 245.1 Hz), 61.2, 61.1, 46.0 (t, *J* = 20.6 Hz), 34.0 (dd, *J* = 5.0, 3.9 Hz), 14.5, 14.2. ^{19}F NMR (377 MHz, Chloroform-*d*) δ -120.0 (d, *J* = 279.3 Hz), -122.9 (d, *J* = 279.7 Hz). HRMS-ESI: Found [M+Na]$^+$ = 323.1068; $C_{15}H_{18}F_2O_4Na$ requires 323.1065.

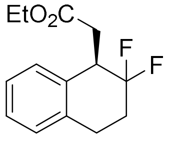

Ethyl (*R*)-2-(2,2-difluoro-1,2,3,4-tetrahydronaphthalen-1-yl)acetate (2n)

Colorless oil. ^{1}H NMR (400 MHz, Chloroform-*d*) δ 7.22 – 7.08 (m, 4H), 4.21 (q, *J* = 7.14 Hz, 2H), 3.85 (tt, *J* = 12.72, 6.41 Hz, 1H), 3.03 (t, *J* = 7.05 Hz, 2H), 2.88 (dd, *J* = 16.36, 6.11 Hz, 1H), 2.58 (dd, *J* = 16.32, 6.74 Hz, 1H), 2.36 – 2.16 (m, 2H), 1.29 (t, *J* = 7.14 Hz, 3H). ^{13}C NMR (101 MHz, Chloroform-*d*) δ 172.0, 136.0 (t, *J* = 4.38 Hz), 133.8, 128.7, 128.4, 127.2, 126.8, 124.1 (dd, *J* = 244.31, 241.81 Hz), 61.1, 42.7 (dd, *J* = 26.82, 22.80 Hz), 37.4 (t, *J* = 5.10 Hz), 29.0 (t, *J* = 24.34 Hz), 26.8 (dd, *J* = 6.55, 4.89 Hz), 14.3. ^{19}F NMR (377 MHz, Chloroform-*d*) δ -97.4 (d, *J* = 238.76 Hz), -104.0 (d, *J* = 238.76 Hz). HRMS-ESI: Found [M+Na]$^+$ = 277.1015; $C_{14}H_{16}F_2O_2Na$ requires 277.1011.

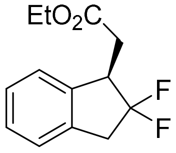

Ethyl (*R*)-2-(2,2-difluoro-2,3-dihydro-1H-inden-1-yl)acetate (2o)

Colorless oil. ^{1}H NMR (400 MHz, Chloroform-*d*) δ 7.24 – 6.91 (m, 4H), 4.23 (q, *J* = 7.13 Hz, 2H), 4.06 – 3.92 (m, 1H), 3.41 (dd, *J* = 15.57, 12.61 Hz, 2H), 2.86 (dd, *J* = 16.46, 6.36 Hz, 1H), 2.61 (ddd, *J* = 16.54, 7.83, 1.25 Hz, 1H), 1.30 (t, *J* = 7.12 Hz, 3H). ^{13}C NMR (101 MHz, Chloroform-*d*) δ 171.7, 140.8 (dd, *J* = 5.08, 1.82 Hz), 136.6 (t, *J* = 4.71 Hz), 133.5, 128.1, 127.9, 124.9, 124.2, 61.1, 48.0 (dd, *J* = 27.12, 22.35 Hz), 41.5 (t, *J* = 26.69 Hz), 33.8 (dd, *J* = 8.21, 2.23 Hz), 14.3. ^{19}F NMR (377 MHz, Chloroform-*d*) δ -97.3 (d, *J* = 229.32 Hz), -106.8 (d, *J* = 228.93 Hz). HRMS-ESI: Found [M+Na]$^+$ = 263.0864; $C_{13}H_{14}F_2O_2Na$ requires 263.0854.

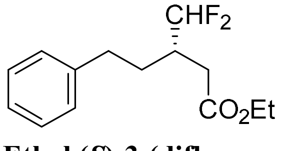

Ethyl (*S*)-3-(difluoromethyl)-5-phenylpentanoate (2p)

Colorless oil. ^{1}H NMR (400 MHz, Chloroform-*d*) δ 7.32-7.13 (m, 5H), 5.88 (td, *J* = 56.6, 3.02 Hz, 1H), 4.15 (q, *J* = 7.14 Hz, 2H), 2.70 (t, *J* = 7.97 Hz, 2H), 2.64-2.29 (m, 3H), 1.98-1.86 (m, 1H), 1.76 – 1.64 (m, 1H), 1.26 (t, *J* = 7.14 Hz). ^{13}C NMR (101 MHz, Chloroform-*d*) δ 171.4, 140.9, 128.3, 128.1, 125.9, 117.4 (t, *J* = 242.5 Hz), 60.7, 38.8 (t, *J* = 19.8 Hz), 32.9, 29.7 (t, *J* = 4.00 Hz), 32.6 (t, *J* = 4.87 Hz), 14.2. HRMS-ESI: Found [M+Na]$^+$ = 279.1169; $C_{14}H_{18}F_2O_2Na$ requires 279.1167.

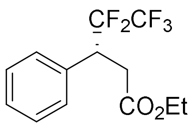

Ethyl (*R*)-4,4,5,5,5-pentafluoro-3-phenylpentanoate (2q)

Colorless oil. ^{1}H NMR (400 MHz, Chloroform-*d*) δ 7.40 – 7.27 (m, 5H), 4.14 – 3.79 (m, 3H), 3.09 (dd, *J* = 16.11, 4.64 Hz, 1H), 2.88 (dd, *J* = 16.13, 10.14 Hz, 1H), 1.10 (t, *J* = 7.13 Hz, 3H). ^{13}C NMR (101 MHz, Chloroform-*d*) δ 170.2, 133.6 (d, *J* = 5.85 Hz), 129.4, 128.8, 128.7, 122.5 – 109.6 (m, CF_2CF_3, 2C), 61.1, 44.1 (t, *J* = 20.75 Hz), 34.3, 14.1. ^{19}F NMR (377 MHz, Chloroform-*d*) δ -81.4, -115.2 (d, *J* = 269.84 Hz), -121.4 (d, *J* = 270.27 Hz). HRMS-ESI: Found $[M+Na]^+$ = 319.0722; $C_{13}H_{13}F_5O_2Na$ requires 319.0728.

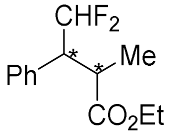

Ethyl-4,4-difluoro-2-methyl-3-phenyl-4l3-butanoate (2r)

Colorless oil. ^{1}H NMR (400 MHz, Chloroform-*d*) δ 7.38 – 7.29 (m, 3H), 7.25 – 7.21 (m, 2H), 5.99 (td, *J* = 56.21, 3.65 Hz, 1H), 4.21 (qd, *J* = 7.15, 1.05 Hz, 2H), 3.44 – 3.31 (m, 1H), 2.98 (dq, *J* = 10.54, 7.06 Hz, 1H), 1.30 (t, *J* = 7.14 Hz, 3H), 0.99 (d, *J* = 7.03 Hz, 3H). ^{13}C NMR (101 MHz, Chloroform-*d*) δ 175.3, 134.5 (t, *J* = 3.39 Hz), 129.7, 128.9, 128.1, 116.8 (t, *J* = 244.23 Hz), 61.0, 52.4 (t, *J* = 19.66 Hz), 39.4 (t, *J* = 3.74 Hz), 16.3, 14.3. ^{19}F NMR (Chloroform-*d*, 376 MHz) δ -120.3 (d, *J* = 34.0 Hz). HRMS-ESI: Found $[M+Na]^+$ = 265.1024; $C_{13}H_{16}F_2O_2Na$ requires 265.1011.

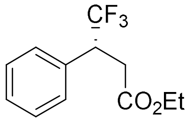

Ethyl (*R*)-4,4,4-trifluoro-3-phenylbutanoate (2s)

Colorless oil. ^{1}H NMR (400 MHz, Chloroform-*d*) δ 7.36 (d, *J* = 3.77 Hz, 5H), 4.16 – 4.00 (m, 2H), 4.01 – 3.88 (m, 1H), 3.05 (dd, *J* = 16.14, 5.13 Hz, 1H), 2.91 (dd, *J* = 16.16, 9.78 Hz, 1H), 1.16 (t, *J* = 7.12 Hz, 3H). ^{13}C NMR (101 MHz, Chloroform-*d*) δ 170.1, 133.9 (q, *J* = 1.66 Hz), 129.1, 128.8, 128.7, 126.5 (q, *J* = 279.74 Hz), 61.2, 46.3 (q, *J* = 27.76 Hz), 34.7 (d, *J* = 2.45 Hz), 14.1. ^{19}F NMR (377 MHz, Chloroform-*d*) δ -70.3. HRMS-ESI: Found $[M+Na]^+$ = 269.0772; $C_{12}H_{13}F_3O_2Na$ requires 269.0760.

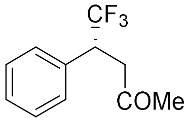

(*R*)-5,5,5-trifluoro-4-phenylpentan-2-one (2t)

Colorless oil. ^{1}H NMR (400 MHz, Chloroform-*d*) δ 7.38 – 7.29 (m, 5H), 4.01 (qdd, *J* = 9.66, 8.12, 5.33 Hz, 1H), 3.16 – 3.00 (m, 2H), 2.12 (s, 3H). ^{1}H NMR (400 MHz, Chloroform-*d*) δ 7.42 –7.28 (m, 5H), 4.15 –3.88 (m, 1H), 3.20 –2.96 (m, 2H), 2.12 (s, 3H). ^{13}C NMR (126 MHz, Chloroform-*d*) δ 203.8, 134.5, 129.1, 128.9, 128.5, 126.8 (q, *J*= 279.4 Hz), 44.7 (q, *J*= 27.6 Hz), 43.1, 30.5.^{19}F NMR (376 MHz, Chloroform-d) δ -70.0 (d, *J*= 9.6 Hz). HRMS-ESI: Found $[M+Na]^+$ = 239.0658; $C_{11}H_{11}F_3ONa$ requires 239.0654.

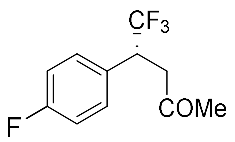

(*R*)-5,5,5-trifluoro-4-(4-fluorophenyl)pentan-2-one (2u)

Colorless oil. ^{1}H NMR (400 MHz, Chloroform-*d*) δ 7.34 – 7.22 (m, 2H), 7.14 – 6.98 (m, 2H), 4.00 (ddq, *J* = 9.42, 4.63 Hz, 1H), 3.15 – 2.97 (m, 2H), 2.12 (s, 3H). ^{13}C NMR (101 MHz,

Chloroform-*d*) δ 203.6, 162.8 (d, *J* = 247.33 Hz), 130.8 (d, *J* = 8.13 Hz), 130.3, 126.7 (q, *J* = 279.76 Hz), 115.9 (d, *J* = 21.59 Hz), 44.0 (q, *J* = 27.88 Hz), 43.2 (d, *J* = 1.98 Hz), 30.5. ^{19}F NMR (377 MHz, Chloroform-*d*) δ -70.2, -113.6. HRMS-ESI: Found $[M+Na]^+$ = 257.0565; $C_{11}H_{10}F_4ONa$ requires 257.0560.

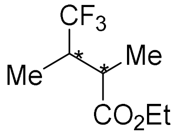

Ethyl-4,4,4-trifluoro-2,3-dimethylbutanoate (2v)

Colorless oil. ^{1}H NMR (400 MHz, Chloroform-*d*) δ 4.16 (qd, *J* = 7.1, 1.9 Hz, 2H), 2.85 – 2.69 (m, 2H), 1.26 (t, *J* = 7.1 Hz, 3H), 1.20 – 1.16 (m, 3H), 1.10 (d, *J* = 6.9 Hz, 3H). ^{13}C NMR (Chloroform-*d*, 101 MHz) δ 174.5, 133.0 – 122.6 (m), 61.1, 39.4 (q, *J* = 26.2 Hz), 38.4 (q, *J* = 2.1 Hz), 14.2, 12.4, 9.1 (q, *J* = 2.9 Hz).^{19}F NMR (Chloroform-*d*, 376 MHz) δ -71.2 (s). HRMS-ESI: Found $[M+Na]^+$ = 221.0746; $C_8H_{13}F_3O_2Na$ a requires 221.0760.

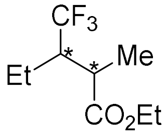

Ethyl-2-methyl-3-(trifluoromethyl)pentanoate (2w)

Colourless oil. ^{1}H NMR (400 MHz, Chloroform-*d*) δ 4.15 (qq, *J* = 7.3, 3.7 Hz, 2H), 2.84 (qd, *J* = 7.1, 4.4 Hz, 1H), 2.75 – 2.57 (m, 1H), 1.69 – 1.55 (m, 1H), 1.53 – 1.48 (m, 1H), 1.26 (d, *J* = 14.3 Hz, 3H), 1.18 (dd, *J* = 7.2, 1.0 Hz, 3H), 0.96 (dd, *J* = 7.5, 1.1 Hz, 3H). ^{13}C NMR (Chloroform-*d*, 101 MHz) δ 174.4, 128.1 (q, *J* = 281.0 Hz), 61.1, 45.7 (q, *J* = 24.6 Hz), 37.8 (q, *J* = 2.6 Hz), 18.0 (q, *J* = 2.3 Hz), 14.2, 12.0, 11.8.^{19}F NMR (Chloroform-*d*, 376 MHz) δ -67.8 (s). HRMS-ESI: Found $[M+Na]^+$ = 235.0928; $C_9H_{15}F_3O_2Na$ requires 235.0916.

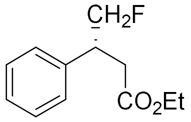

Ethyl (*R*)-4-fluoro-3-phenylbutanoate (2x)

Colorless oil. ^{1}H NMR (400 MHz, Chloroform-*d*) δ 7.37 – 7.30 (m, 2H), 7.29 – 7.19 (m, 3H), 4.69 – 4.42 (m, 2H), 4.08 (qd, *J* = 7.13, 1.78 Hz, 2H), 3.62 – 3.46 (m, 1H), 2.88 (dd, *J* = 15.85, 6.87 Hz, 1H), 2.70 (dd, *J* = 15.49, 8.49 Hz, 1H), 1.17 (t, *J* = 7.14 Hz, 3H). ^{13}C NMR (101 MHz, Chloroform-*d*) δ 171.8, 139.6 (d, *J* = 5.50 Hz), 128.8, 127.9, 127.5, 86.2 (d, *J* = 173.94 Hz), 60.7, 42.7 (d, *J* = 19.04 Hz), 36.7 (d, *J* = 4.97 Hz), 14.2.^{19}F NMR (377 MHz, Chloroform-*d*) δ 17.3. HRMS-ESI: Found $[M+Na]^+$ = 233.0933; $C_{12}H_{15}FO_2Na$ requires 233.0948.

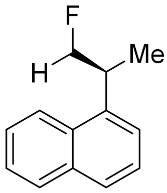

(*S*)-1-(1-fluoropropan-2-yl)naphthalene (4a)

Colorless oil. ^{1}H NMR (400 MHz, Chloroform-*d*) δ 8.16 – 8.09 (m, 1H), 7.92 – 7.86 (m, 1H), 7.80 – 7.73 (m, 1H), 7.61 – 7.39 (m, 4H), 4.72 (ddd, *J* = 47.35, 8.83, 5.20 Hz, 1H), 4.54 (ddd, *J* = 47.50, 8.83, 7.59 Hz, 1H), 4.63 – 4.44 (m, 1H), 1.53 (dd, *J* = 6.89, 1.12 Hz, 3H). ^{13}C NMR (101 MHz, Chloroform-*d*) δ 138.1 (d, *J* = 7.05 Hz), 134.1, 131.7, 129.2, 127.5, 126.3, 125.7, 125.7, 123.6 (d, *J* = 0.82 Hz), 122.9, 88.0 (d, *J* = 173.43 Hz), 34.9 (d, *J* = 19.54 Hz), 17.4 (d, *J* = 4.39 Hz). ^{19}F NMR (377 MHz, Chloroform-*d*) δ -216.4. HRMS-ESI: Found $[M+Na]^+$ = 211.0906; $C_{13}H_{13}FNa$ requires 211.0893.

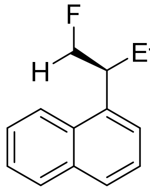

(*S*)-1-(1-fluorobutan-2-yl)naphthalene (4b)

Colorless oil. ^{1}H NMR (400 MHz, Chloroform-*d*) δ 8.16 – 8.10 (m, 1H), 7.92 – 7.85 (m, 1H), 7.83 – 7.74 (m, 1H), 7.59 – 7.38 (m, 4H), 4.71 (ddd, *J* = 15.32, 5.56, 5.08 Hz, 1H), 4.60 (ddd, *J* = 15.52, 5.29, 5.07 Hz, 1H), 3.90 – 3.74 (m, 1H), 2.16 – 2.03 (m, 1H), 1.98 – 1.82 (m, 1H), 0.94 (t, *J* = 7.43 Hz, 3H). ^{13}C NMR (101 MHz, Chloroform-*d*) δ 136.9 (d, *J* = 6.36 Hz), 134.2, 132.4, 129.2, 127.4, 126.2, 125.6, 125.6, 124.1, 123.0, 86.9 (d, *J* = 172.95 Hz), 41.9 (d, *J* = 18.84 Hz), 24.8 (d, *J* = 4.08 Hz), 12.0. ^{19}F NMR (377 MHz, Chloroform-*d*) δ -217.5. HRMS-ESI: Found [M+Na]$^+$ = 225.1063; $C_{14}H_{15}FNa$ requires 225.1050.

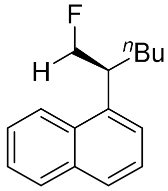

(*S*)-1-(1-fluorohexan-2-yl)naphthalene (4c)

Colorless oil. ^{1}H NMR (400 MHz, Chloroform-*d*) δ 8.18 – 8.11 (m, 1H), 7.93 – 7.88 (m, 1H), 7.81 – 7.77 (m, 1H), 7.60 – 7.41 (m, 4H), 4.71 (ddd, *J* = 28.70, 9.23, 5.53 Hz, 1H), 4.59 (ddd, *J* = 31.83, 9.42, 5.77 Hz, 1H), 3.99 – 3.83 (m, 1H), 2.66 – 2.59 (m, 1H), 2.13 – 2.00 (m, 1H), 1.95 – 1.82 (m, 1H), 1.41 – 1.28 (m, 2H), 0.87 (t, J = 7.03 Hz, 2H). ^{19}F NMR (377 MHz, Chloroform-*d*) δ -216.9. HRMS-ESI: Found [M+Na]$^+$ = 253.1380; $C_{16}H_{19}FNa$ requires 253.1363.

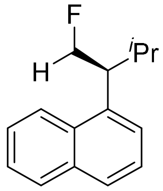

(*S*)-1-(1-fluoro-3-methylbutan-2-yl)naphthalene (4d)

Colorless oil. ^{1}H NMR (400 MHz, Chloroform-d) δ 8.10 (d, *J* = 8.26 Hz, 1H), 7.87 (dd, *J* = 7.67, 1.77 Hz, 1H), 7.76 (d, J = 8.02 Hz, 1H), 7.56 – 7.39 (m, 4H), 4.92 – 4.66 (m, 2H), 3.71 – 3.52 (m, 1H), 2.35 – 2.20 (m, 1H), 1.14 (d, *J* = 6.64 Hz, 3H), 0.85 (d, *J* = 6.74 Hz, 3H). ^{19}F NMR (377 MHz, Chloroform-*d*) δ -221.5. HRMS-ESI: Found [M+Na]$^+$ = 239.1215; $C_{15}H_{17}FNa$ requires 239.1206.

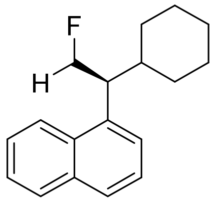

(*S*)-1-(1-cyclohexyl-2-fluoroethyl)naphthalene (4e)

Colorless oil. ^{1}H NMR (400 MHz, Chloroform-*d*) δ 8.12 (d, *J* = 8.28 Hz, 1H), 7.90 – 7.82 (m, 1H), 7.81 – 7.75 (m, 1H), 7.61 – 7.40 (m, 4H), 4.95 – 4.68 (m, 2H), 3.74 – 3.59 (m, 1H), 2.13 – 2.05 (m, 1H), 2.02 – 0.83 (m, 10H). HRMS-ESI: Found [M+Na]$^+$ = 279.1526; $C_{18}H_{21}FNa$ requires 279.1519.

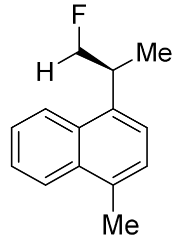

(*S*)-1-(1-fluoropropan-2-yl)-4-methylnaphthalene (4f)

Colorless oil. ^{1}H NMR (400 MHz, Chloroform-*d*) δ 8.22 – 8.12 (m, 1H), 8.12 – 8.01 (m, 1H), 7.63 – 7.49 (m, 2H), 7.33 (s, 2H), 4.72 (ddd, *J* = 47.44, 8.86, 5.20 Hz, 1H), 4.52 (ddd, *J* = 47.52, 8.84, 7.70 Hz, 1H), 4.11 – 3.94 (m, 1H), 2.70 (s, 3H), 1.53 (dd, *J* = 6.87, 1.11 Hz, 3H). ^{13}C NMR (101 MHz, Chloroform-*d*) δ 136.1 (d, *J* = 7.16 Hz), 133.5, 133.2, 131.7, 126.5, 126.0, 125.6, 125.2, 123.4, 123.3, 88.1 (d, *J* = 173.32 Hz), 34.7 (d, *J* = 19.40 Hz), 19.7, 17.4 (d, *J* = 4.26 Hz).^{19}F NMR (377 MHz, Chloroform-*d*) δ -216.1. HRMS-ESI: Found $[M+Na]^+$ = 225.1057; $C_{14}H_{15}FNa$ requires 225.1050.

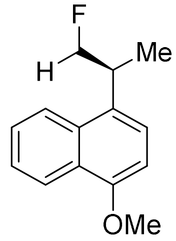

(*S*)-1-(1-fluoropropan-2-yl)-4-methoxynaphthalene (4g)

Colorless oil. ^{1}H NMR (400 MHz, Chloroform-*d*) δ 8.37 – 8.31 (m, 1H), 8.12 – 7.97 (m, 1H), 7.60 – 7.53 (m, 1H), 7.52 – 7.46 (m, 1H), 7.32 (d, *J* = 8.02 Hz, 1H), 6.80 (d, *J* = 8.05 Hz, 1H), 4.68 (ddd, *J* = 47.52, 8.81, 5.07 Hz, 1H), 4.47 (ddd, *J* = 47.62, 8.83, 7.75 Hz, 1H), 3.97 – 3.83 (m, 4H), 1.50 (dd, *J* = 6.85, 1.12 Hz, 3H). ^{13}C NMR (101 MHz, Chloroform-*d*) δ 154.6, 132.6, 129.7 (d, *J* = 7.30 Hz), 126.8, 126.1, 125.1, 123.6 (d, *J* = 1.18 Hz), 123.0, 122.7, 103.5, 88.1 (d, *J* = 173.37 Hz), 55.6, 34.5 (d, *J* = 19.45 Hz), 17.4 (d, *J* = 4.13 Hz). ^{19}F NMR (377 MHz, Chloroform-*d*) δ -215.9. HRMS-ESI: Found $[M+Na]^+$ = 241.1012; $C_{14}H_{15}FONa$ requires 241.0999.

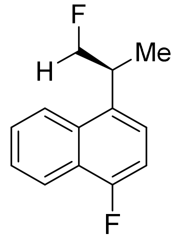

(*S*)-1-fluoro-4-(1-fluoropropan-2-yl)naphthalene (4h)

Colorless oil. ^{1}H NMR (400 MHz, Chloroform-*d*) δ 8.21 – 8.05 (m, 2H), 7.64 – 7.53 (m, 2H), 7.34 (dd, *J* = 8.08, 5.35 Hz, 1H), 7.13 (dd, *J* = 10.19, 8.06 Hz, 1H), 4.68 (ddd, *J* = 47.40, 8.89, 5.34 Hz, 1H), 4.51 (ddd, *J* = 47.44, 8.90, 7.34 Hz, 1H), 4.04 – 3.87 (m, 1H), 1.50 (dd, *J* = 6.93, 1.17 Hz, 3H). ^{13}C NMR (101 MHz, Chloroform-*d*) δ 159.3, 133.0 (d, *J* = 3.14 Hz), 127.3, 126.0 (d, *J* = 2.00 Hz), 124.3, 123.4 (d, *J* = 1.01 Hz), 123.4 – 123.2 (m), 123.0 (d, *J* = 2.81 Hz), 121.6 (d, *J* = 6.13 Hz), 109.1 (d, *J* = 19.75 Hz), 87.9 (d, *J* = 174.01 Hz), 34.7 (d, *J* = 19.61 Hz), 17.4 (d, *J* = 4.55 Hz). ^{19}F NMR (377 MHz, Chloroform-*d*) δ -124.7, -216.5. HRMS-ESI: Found $[M+Na]^+$ = 229.0787; $C_{13}H_{12}F_2Na$ requires 229.0799.

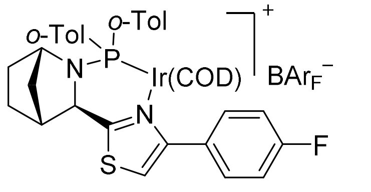

Catalyst G

Orange solid. ^{1}H NMR (400 MHz, Chloroform-*d*) δ 7.75 – 7.68 (m, 8H), 7.51 (s, 4H), 7.48 – 7.40 (m, 4H), 7.36 – 7.29 (m, 1H), 7.25 – 7.17 (m, 3H), 7.07 (t, J = 7.64 Hz, 1H), 6.96 – 6.88 (m, 2H), 6.88 – 6.81 (m, 2H), 6.61 (ddd, J = 9.34, 7.92, 1.32 Hz, 1H), 5.04 – 4.97 (m, 1H), 4.73 (d, J = 1.70 Hz, 1H), 4.75 – 4.63 (m, 1H), 3.64 – 3.60 (m, 1H), 3.58 – 3.51 (m, 1H), 3.23 (s, 3H), 3.11 – 3.05 (m, 1H), 2.75 (p, J = 7.34 Hz, 1H), 2.31 – 2.26 (m, 1H), 2.23 (s, 3H), 2.19 – 2.13 (m, 1H), 2.02 (d, J = 10.80 Hz, 1H), 1.92 – 1.85 (m, 2H), 1.82 – 1.77 (m, 2H), 1.73 – 1.67 (m, 1H), 1.48 – 1.38 (m, 1H), 1.09 – 1.02 (m, 1H), 1.02 – 0.93 (m, 1H), 0.87 – 0.80 (m, 2H). ^{13}C NMR (101 MHz, Chloroform-*d*) δ 172.2 (d, J = 1.74 Hz), 162.6, 162.1, 161.6, 161.1, 156.7, 141.8 (d, J = 13.63 Hz), 139.6 (d, J = 15.68 Hz), 135.0, 132.8 – 132.4 (m), 131.5, 129.6 – 128.4 (m), 127.7 (d, J = 7.38 Hz), 126.7 (d, J = 9.21 Hz), 126.0, 125.2, 124.6, 123.3, 120.6, 117.8 – 117.4 (m), 116.8, 116.2 (d, J = 21.78 Hz), 90.8 (d, J = 7.77 Hz), 78.5, 78.3, 75.7, 67.1 (d, J = 9.62 Hz), 65.0, 60.2, 42.5, 38.9, 36.8, 34.7, 29.9 (d, J = 12.85 Hz), 29.2, 28.0, 24.7 (d, J = 5.94 Hz), 24.5, 21.8 (d, J = 8.42 Hz). ^{31}P NMR (162 MHz, Chloroform-*d*) δ 49.40. ^{19}F NMR (377 MHz, Chloroform-*d*) δ -62.4, -108.6. HRMS-ESI: Found [M-BAr$_F$]$^+$ = 787.2280; $C_{37}H_{40}IrFN_2PS$ requires 787.2258. $[\alpha]_D^{25.0}$ = +21 (C= 0.10 in $CHCl_3$).

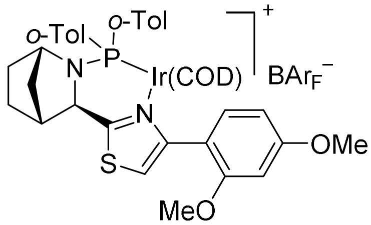

Catalyst H

Orange solid. ^{1}H NMR (400 MHz, Chloroform-*d*) δ 7.75 – 7.67 (m, 8H), 7.52 (s, 4H), 7.46 – 7.39 (m, 4H), 7.36 – 7.30 (m, 1H), 7.25 – 7.18 (m, 2H), 7.09 (t, J = 7.51 Hz, 1H), 6.71 – 6.61 (m, 1H), 6.48 (d, J = 2.27 Hz, 1H), 6.07 (dd, J = 8.46, 2.30 Hz, 1H), 5.96 (d, J = 8.37 Hz, 1H), 5.05 – 4.87 (m, 1H), 4.74 – 4.65 (m, 2H), 3.80 (s, 3H), 3.77 (s, 3H), 3.63 (s, 1H), 3.60 – 3.49 (m, 1H), 3.26 (s, 3H), 3.15 – 3.11 (m, 1H), 2.88 – 2.76 (m, 1H), 2.41 – 2.23 (m, 1H), 2.19 (s, 3H), 2.10 – 2.02 (m, 1H), 1.99 – 1.80 (m, 1H), 1.69 – 1.59 (m, 3H), 1.44 – 1.31 (m, 2H), 1.23 – 1.05 (m, 3H), 1.01 – 0.80 (m, 2H). ^{13}C NMR (101 MHz, Chloroform-*d*) δ 169.5, 162.7, 162.6, 162.1, 161.6, 161.1, 157.3, 152.9, 142.0, 139.7 (d, J = 15.61 Hz), 134.9, 132.9, 132.7 – 132.2 (m), 131.4 – 131.2 (m), 129.5, 129.3 – 129.1 (m), 129.0 – 128.7 (m), 128.7 – 128.4 (m), 127.6 (d, J = 7.38 Hz), 126.6 (d, J = 9.02 Hz), 126.0, 125.6, 125.0, 123.3, 120.6, 118.1, 117.8 – 117.4 (m), 114.8, 105.0, 98.4, 89.6 (d, J = 7.09 Hz), 75.7, 66.9 (d, J = 9.88 Hz), 64.6, 60.1 (d, J = 4.93 Hz), 55.8 (d, J = 4.29 Hz), 42.5 (d, J = 6.04 Hz), 39.0, 36.6 (d, J = 4.11 Hz), 34.9, 30.0, 29.3, 28.1, 26.8, 24.5 (d, J = 6.11 Hz), 21.8 (d, J = 8.50 Hz). ^{31}P NMR (162 MHz, Chloroform-*d*) δ 48.95. ^{19}F NMR (377 MHz, Chloroform-*d*) δ -62.4. HRMS-ESI: Found [M-BAr$_F$]$^+$ = 829.2586; $C_{39}H_{45}IrN_2O_2PS$ requires 829.2563. $[\alpha]_D^{25.0}$ = +19 (C= 0.10 in $CHCl_3$).

6. General procedure for further transformation

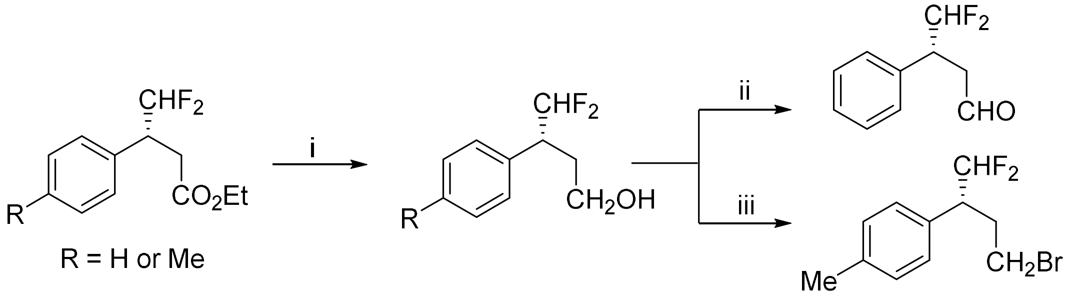

Procedure i: The corresponding ester (1.0 mmol) was dissolved in dry THF in dry glassware. $LiAlH_4$ (1.5 equiv.) was added slowly at 0 °C and stirred at room temperature for 1 hour. At 0 °C, x mL H_2O was added to the mixture, then x mL 10% NaOH, 3 x mL H_2O was added to quench the reaction (x is the mass of $LiAlH_4$, gram). The mixture was filtered through celite. The filtrate was concentrated under reduced pressure and the product was purified by column chromatography.

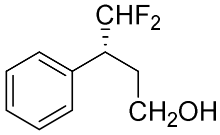

(*R*)-4,4-difluoro-3-phenylbutan-1-ol (7)

Colorless oil. ^{1}H NMR (400 MHz, Chloroform-*d*) δ 7.42 – 7.21 (m, 5H), 5.91 (td, *J* = 56.61, 3.72 Hz, 1H), 3.72 – 3.62 (m, 1H), 3.53 – 3.43 (m, 1H), 3.35 – 3.18 (m, 1H), 2.30 – 2.14 (m, 1H), 2.09 – 1.92 (m, 1H). ^{13}C NMR (101 MHz, Chloroform-*d*) δ 136.6 (dd, *J* = 5.29, 3.17 Hz), 129.0, 128.9, 127.9, 118.0 (t, *J* = 244.37 Hz), 60.0, 46.5 (t, *J* = 19.96 Hz), 32.5 – 29.8 (m). ^{19}F NMR (377 MHz, Chloroform-*d*) δ -118.5 (d, *J* = 276.71 Hz), -122.5 (d, *J* = 276.50 Hz). HRMS-ESI: Found $[M+Na]^+$ = 209.0756; $C_{10}H_{12}F_2ONa$ requires 209.0748.

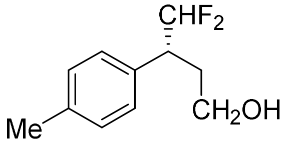

(*R*)-4,4-difluoro-3-(p-tolyl)butan-1-ol (16)

Colorless oil. ^{1}H NMR (400 MHz, Chloroform-*d*) δ 7.22 – 7.12 (m, 4H), 5.87 (td, *J* = 56.71, 3.76 Hz, 1H), 3.74 – 3.61 (m, 1H), 3.51 – 3.44 (m, 1H), 3.30 – 3.11 (m, 1H), 2.34 (s, 3H), 2.23 – 2.11 (m, 1H), 2.03 – 1.89 (m, 1H). ^{13}C NMR (101 MHz, Chloroform-*d*) δ 137.6, 133.4 (dd, *J* = 5.03, 3.39 Hz), 129.6, 128.9, 118.1 (t, *J* = 244.27 Hz), 60.1, 46.1 (t, *J* = 19.92 Hz), 31.3 (t, *J* = 3.91 Hz), 21.2. ^{19}F NMR (377 MHz, Chloroform-*d*) δ -118.5 (d, *J* = 276.39 Hz), -122.6 (d, *J* = 276.13 Hz). HRMS-ESI: Found $[M+Na]^+$ = 223.0908; $C_{11}H_{14}F_2ONa$ requires 223.0905.

Procedure ii: The corresponding alcohol (1 equiv.) was dissolved in the dry DCM, and DMP (1.2 equiv.) was added in the solution. After complete consumption of the starting material, the mixture was filtered and the filtrate was concentrated under vacuo. The crude was purified by column chromatography to obtain the aldehyde.

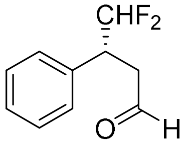

(*R*)-4,4-difluoro-3-phenylbutanal (8)

Colorless oil. ^{1}H NMR (400 MHz, Chloroform-*d*) δ 9.74 (p, *J* = 1.11 Hz, 1H), 7.39 – 7.27 (m, 5H), 5.93 (td, *J* = 56.38, 3.23 Hz, 1H), 3.81 – 3.62 (m, 1H), 3.10 (ddd, *J* = 18.07, 5.64, 1.10 Hz, 1H), 2.97 (ddd, *J* = 18.06, 8.38, 1.49 Hz, 1H). ^{13}C NMR (101 MHz, Chloroform-*d*) δ 199.1, 135.8 (dd, *J* = 5.83, 2.52 Hz), 129.1, 128.9, 128.3, 117.1 (t, *J* = 245.01 Hz), 43.7 (t, *J* = 20.48 Hz), 42.6 (dd, *J* = 4.41, 3.13 Hz). ^{19}F NMR (377 MHz, Chloroform-*d*) δ -118.6 (d, *J* = 277.92 Hz), -123.5 (d, *J* = 277.89 Hz). HRMS-ESI: Found $[M+Na]^+$ = 207.0599; $C_{10}H_{10}F_2ONa$ requires 207.0592.

Procedure iii: To a solution of CBr_4 (1.05 equiv.) in dry DCM was added corresponding alcohol (1 equiv.) and PPh_3(1.05 equiv.) at 0 °C. The solution was warmed to room temperature for 3h, the reaction mixture was evaporated. The crude was purified by column chromatography on silica gel to give the bromide.

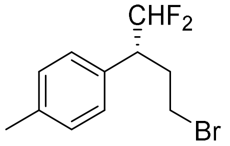

(*R*)-1-(4-bromo-1,1-difluorobutan-2-yl)-4-methylbenzene (17)

Colorless oil. ^{1}H NMR (400 MHz, Chloroform-*d*) δ 7.23 – 7.12 (m, 4H), 5.87 (td, *J* = 56.47, 3.60 Hz, 1H), 3.42 (ddd, *J* = 10.55, 6.41, 4.64 Hz, 1H), 3.36 – 3.23 (m, 1H), 3.11 (td, *J* = 9.85, 5.82 Hz, 1H), 2.48 – 2.21 (m, 5H). ^{13}C NMR (101 MHz, Chloroform-*d*) δ 138.0, 132.2 (dd, *J* = 5.65, 2.87 Hz), 129.8, 128.9, 117.7 (t, *J* = 244.86 Hz), 47.9 (t, *J* = 20.06 Hz), 31.3 (dd, *J* = 5.09, 3.48 Hz), 30.8, 21.2. ^{19}F NMR (377 MHz, Chloroform-*d*) δ -117.9 (d, *J* = 277.43 Hz), -122.9 (d, *J* = 277.43 Hz). HRMS-ESI: Found $[M+Na]^+$ = 285.0068; $C_{11}H_{13}F_2BrONa$ requires 285.0061 (Br^{79}).

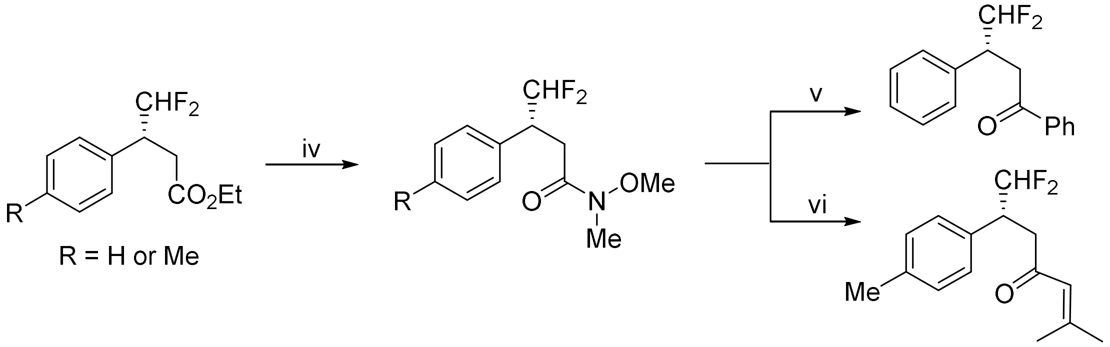

Procedure iv:
A solution of corresponding ester (1 equiv.) and HN(OMe)Me·HCl (2 equiv.) in THF was cooled to -5 °C. Isopropyl magnesium chloride (4 equiv.) was added dropwise over 30 minutes. The reaction mixture was stirred at room temperature for 1h, which monitored by TLC. Upon completion the reaction was quenched with saturated ammonium chloride solution, which was then extracted with ether. The organic layers were combined, washed with brine, dried over Na_2SO_4, filtered and concentrated to give the corresponding Weinreb amide, which was purified by column chromatography on silica gel.

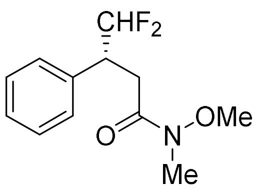

(*R*)-4,4-difluoro-N-methoxy-N-methyl-3-phenylbutanamide (9)

Colorless oil. ^{1}H NMR (400 MHz, Chloroform-*d*) δ 7.40 – 7.27 (m, 5H), 6.01 (td, *J* = 56.59, 3.08 Hz, 1H), 3.87 – 3.68 (m, 1H), 3.64 (s, 3H), 3.13 (s, 3H), 3.02 (td, *J* = 14.76, 12.84, 7.10 Hz, 2H). ^{13}C NMR (101 MHz, Chloroform-*d*) δ 171.5, 136.6, 129.1, 128.8, 127.9, 117.4 (t, *J* = 244.26 Hz), 61.4, 45.2 (t, *J* = 20.10 Hz), 32.3, 31.2. ^{19}F NMR (377 MHz, Chloroform-*d*) δ -120.5 (d, *J* = 277.73 Hz), -122.7 (d, *J* = 277.88 Hz). HRMS-ESI: Found $[M+Na]^+$ = 266.0970; $C_{12}H_{15}F_2NO_2Na$ requires 266.0963.

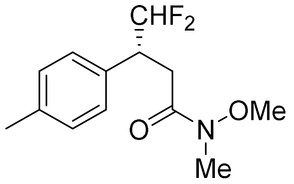

(*R*)-4,4-difluoro-N-methoxy-N-methyl-3-(p-tolyl)butanamide (14)

Colorless oil. ^{1}H NMR (400 MHz, Chloroform-*d*) δ 7.23 – 7.18 (m, 2H), 7.17 – 7.12 (m, 2H), 5.98 (td, *J* = 56.67, 3.11 Hz, 1H), 3.80 – 3.64 (m, 1H), 3.65 (s, 3H), 3.13 (s, 3H), 2.98 (qd, *J* = 16.69, 7.11 Hz, 2H), 2.33 (s, 3H). ^{13}C NMR (101 MHz, Chloroform-*d*) δ 171.7, 137.6, 133.6 (dd, *J* = 5.40, 2.46 Hz), 129.5, 128.9, 117.5 (t, *J* = 244.23 Hz), 61.4, 44.8 (t, *J* = 20.07 Hz), 32.3, 31.2, 21.2. ^{19}F NMR (377 MHz, Chloroform-*d*) δ -120.4 (d, *J* = 277.15 Hz), -122.7 (d, *J* = 277.32 Hz). HRMS-ESI: Found $[M+Na]^+$ = 280.1121; $C_{13}H_{17}F_2NO_2Na$ requires 280.1120.

Procedure v:

Grignard solution (1.5 equiv.) was slowly added to the stirring solution of the corresponding Weinreb amide (1 equiv.) in THF at -30 °C. The reaction mixture was then stirred at room temperature for 1h and then quenched with a saturated aqueous NH_4Cl solution. The aqueous phase was extracted with Et_2O and combined organic layer were washed with brine, dried over Na_2SO_4.The solvent was evaporated and the resulting crude was purified by a short column chromatography to yield pure of ketone.

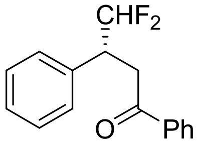

(*R*)-4,4-difluoro-1,3-diphenylbutan-1-one (10)

Colorless oil. ^{1}H NMR (400 MHz, Chloroform-*d*) δ 7.99 – 7.91 (m, 2H), 7.61 – 7.53 (m, 1H), 7.50 – 7.42 (m, 2H), 7.38 – 7.26 (m, 5H), 6.04 (td, *J* = 56.63, 2.95 Hz, 1H), 4.02 – 3.85 (m, 1H), 3.66 – 3.48 (m, 2H). ^{13}C NMR (101 MHz, Chloroform-*d*) δ 196.9, 136.7, 136.6 (dd, *J* = 5.61, 1.94 Hz), 133.5, 129.1, 128.9, 128.8, 128.2, 127.9, 117.4 (t, *J* = 244.60 Hz), 44.7 (t, *J* = 20.04 Hz), 37.5 (dd, *J* = 4.50, 2.89 Hz). ^{19}F NMR (377 MHz, Chloroform-*d*) δ -119.7 (d, *J* = 277.81 Hz), -122.8 (d, *J* = 277.29 Hz). HRMS-ESI: Found $[M+Na]^+$ = 283.0911; $C_{16}H_{14}F_2ONa$ requires 283.0905.

Procedure vi:

To a solution of 1-bromo-2-methylprop-1-ene (1 equiv.) in dry THF was added nBuLi (1 equiv.) slowly at -78 °C. The solution was stirring for 1h at the same temperature, then was slowly added to the stirring solution of the corresponding Weinreb amide. The solution was warmed to 0 °C and stirred until the reaction completed. quenched with a saturated aqueous NH_4Cl solution. The aqueous phase was extracted with Et_2O and combined organic layer were washed

with brine, dried over Na_2SO_4.The solvent was evaporated and the resulting crude was purified by column chromatography to yield pure of ketone.

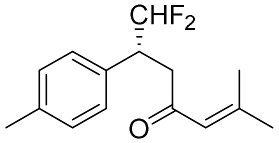

(*R*)-7,7-difluoro-2-methyl-6-(p-tolyl)hept-2-en-4-one (15)

Colorless oil. ^{1}H NMR (400 MHz, Chloroform-*d*) δ 7.21 – 7.06 (m, 4H), 6.11 – 5.72 (m, 2H), 3.78 – 3.61 (m, 1H), 3.04 (dd, *J* = 17.38, 5.73 Hz, 1H), 2.89 (dd, *J* = 17.35, 8.07 Hz, 1H), 2.32 (s, 3H), 2.09 (d, *J* = 1.22 Hz, 3H), 1.86 (d, *J* = 1.34 Hz, 3H). ^{13}C NMR (101 MHz, Chloroform-*d*) δ 197.2, 156.6, 137.4, 133.7 (dd, *J* = 5.20, 2.52 Hz), 129.5, 128.9, 123.7, 117.5 (t, *J* = 244.24 Hz), 44.3 (t, *J* = 20.10 Hz), 42.8 (dd, *J* = 4.37, 2.88 Hz), 27.8, 21.2, 21.0. ^{19}F NMR (377 MHz, Chloroform-*d*) δ -120.2 (d, *J* = 276.41 Hz), -122.5 (d, *J* = 276.52 Hz). HRMS-ESI: Found $[M+Na]^+$ = 275.1216; $C_{15}H_{18}F_2ONa$ requires 275.1218.

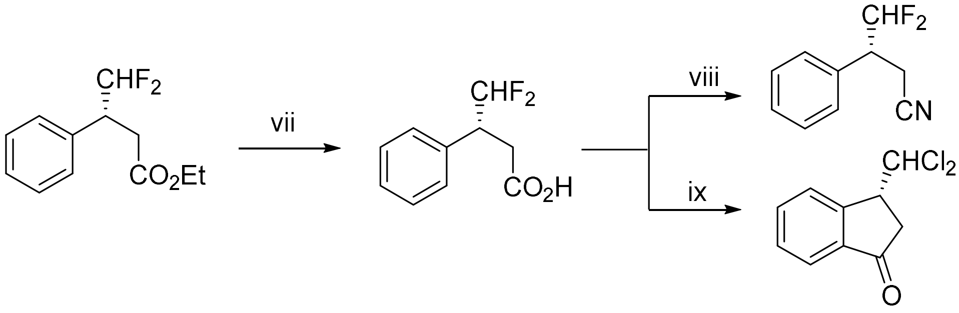

Procedure vii:

A solution of corresponding ester (1 equiv.) and 2M NaOH in MeOH was heated to reflux for 1 h. After cooling down to room temperature, the solution was extracted with DCM (3x). The aqueous phase was acidified to PH 2~3 and extracted with ethyl acetate (3x). The combined organic phase (ethyl acetate phase) was dried over Na_2SO_4, and evaporated to afford pure acid.

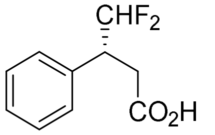

(*R*)-4,4-difluoro-3-phenylbutanoic acid (11)

Colorless oil. ^{1}H NMR (400 MHz, Chloroform-*d*) δ 7.39 – 7.26 (m, 5H), 5.92 (td, *J* = 56.25, 3.29 Hz, 1H), 3.69 – 3.52 (m, 1H), 3.02 (dd, *J* = 16.80, 5.68 Hz, 1H), 2.83 (dd, *J* = 16.73, 8.96 Hz, 1H). ^{13}C NMR (101 MHz, Chloroform-*d*) δ 175.7, 135.5 (dd, *J* = 5.45, 2.25 Hz), 129.0, 128.8, 128.3, 116.9 (t, *J* = 245.15 Hz), 45.7 (t, *J* = 20.45 Hz), 33.0 (dd, *J* = 5.31, 3.85 Hz). ^{19}F NMR (377 MHz, Chloroform-*d*) δ -119.2 (d, *J* = 278.32 Hz), -124.0 (d, *J* = 278.42 Hz). HRMS-ESI: Found $[M+Na]^+$ = 223.0544; $C_{10}H_{10}F_2O_2Na$ requires 223.0541.

Procedure viii:

To a solution of corresponding acid (1 equiv.) in dioxane were added Boc_2O (1.3 equiv.), $(NH_4)_2CO_3$ (1.3 equiv.) and pyridine (40 equiv.). After stirring for 18 h at 25 ºC, the solvent was evaporated. The residue was diluted with EtOAc and the combined organic layers were washed with a 20% aqueous solution of citric acid (3x), brine (1x) and dried over Na_2SO_4. The solvent was evaporated and the residue was dissolved in DCM. To the solution was added $(CF_3CO)_2O$ (1.3 equiv.) and Et_3N (2.7 equiv.). The mixture was stirred at 25 ºC for 1h and poured over H_2O. The mixture was extracted with DCM (3x) and dried over Na_2SO_4. The

solvent was evaporated and the residue was purified by column chromatography to afford the desired nitrile.

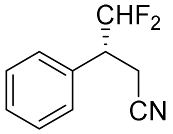

(*R*)-4,4-difluoro-3-phenylbutanenitrile (12)

Colorless oil. ^1H NMR (400 MHz, Chloroform-*d*) δ 7.45 – 7.36 (m, 3H), 7.33 – 7.28 (m, 2H), 5.99 (td, *J* = 55.58, 3.18 Hz, 1H), 3.51 – 3.35 (m, 1H), 2.96 (dd, *J* = 16.98, 5.56 Hz, 1H), 2.83 (dd, *J* = 16.98, 9.28 Hz, 1H). ^{13}C NMR (101 MHz, Chloroform-*d*) δ 133.7 (dd, *J* = 5.64, 2.09 Hz), 129.4, 129.1, 128.5, 117.3, 116.0 (t, *J* = 249.45 Hz), 46.3 (dd, *J* = 21.42, 19.97 Hz), 17.7 (dd, *J* = 6.97, 4.43 Hz). ^{19}F NMR (377 MHz, Chloroform-*d*) δ -118.1 (d, *J* = 281.30 Hz), -125.6 (d, *J* = 280.92 Hz). HRMS-ESI: Found $[M+Na]^+$ = 204.0601; $C_{10}H_9F_2NNa$ requires 204.0595.

Procedure ix:

The carboxylic acid (1 equiv) and cyanuric chloride (1.6 equiv) were dissolved in DCM. Pyridine (1 equiv) was drop-wise, and the mixture was stirred vigorously. After 15 min at room temperature, $AlCl_3$ (3 equiv) was added portion-wise. After the reaction was deemed complete, it was then filtered through Celite and the organic phase was washed with cooled water three times. The organic layer was dried over anhydrous Na_2SO_4, filtered and the solvent was evaporated. The crude product was purified by column chromatography.

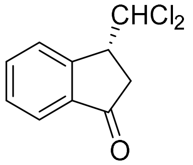

(*R*)-3-(dichloromethyl)-2,3-dihydro-1H-inden-1-one (13)

White solid. ^1H NMR (400 MHz, Chloroform-*d*) δ 7.81 (dt, *J* = 7.57, 1.07 Hz, 1H), 7.72 – 7.63 (m, 2H), 7.56 – 7.45 (m, 1H), 6.21 (d, *J* = 3.29 Hz, 1H), 4.19 (td, *J* = 5.33, 3.15 Hz, 1H), 2.93 (s, 1H), 2.92 (d, *J* = 0.71 Hz, 1H). ^{13}C NMR (101 MHz, Chloroform-*d*) δ 203.2, 151.4, 138.2, 135.1, 129.5, 126.1, 124.2, 74.9, 48.8, 39.3. HRMS-ESI: Found $[M+Na]^+$ = 236.9846; $C_{10}H_8Cl_2ONa$ requires 236.9844 (Cl^{35}).

7. Separation method data and specific rotation of chiral compounds

Entry	Product	Separation method	Optical rotation	*ee* (%)
1	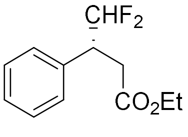	**SFC**, ASH column, 5% MeOH, 2 ml/min, t_R = 7.45 min (major)/8.07 (minor)	$[\alpha]_D^{25.0}$ = -33 (C= 0.10 in $CHCl_3$)	96
2	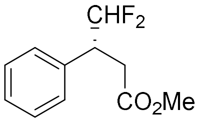	**SFC**, ADH column, 5% MeOH, 2 ml/min, t_R = 2.57 min (major)/2.80 (minor)	$[\alpha]_D^{25.0}$ = -44 (C= 0.10 in $CHCl_3$)	93
3	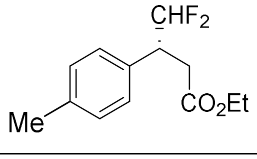	**SFC**, ADH column, 5% MeOH, 2 ml/min, t_R = 2.84 min (major)/3.25 (minor)	$[\alpha]_D^{25.0}$ = -28 (C= 0.10 in $CHCl_3$)	92

4	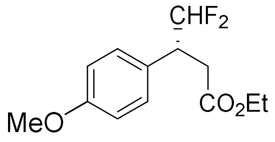	**SFC**, OJH column, 5% MeOH, 2 ml/min, t_R = 3.59 min (minor)/3.83 (major)	$[\alpha]_D^{25.0}$ = -36 (C= 0.10 in $CHCl_3$)	91
5	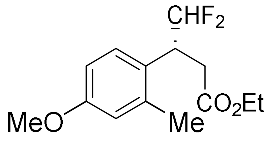	**SFC**, IF column, 5% MeOH, 2 ml/min, t_R = 3.66 min (major)/4.10 (minor)	$[\alpha]_D^{25.0}$ = -37 (C= 0.10 in $CHCl_3$)	90
6	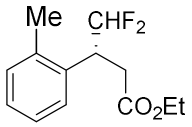	**SFC**, ADH column, 5% MeOH, 2 ml/min, from *E*-isomer: t_R = 2.21 min (major)/2.36 (minor)	$[\alpha]_D^{25.0}$ = -40 (C= 0.10 in $CHCl_3$)	93
7	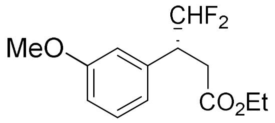	**GC-MS**, Chiraldex β-DM, 90 °C iso 200 min, 1.0 mL/min. t_R = 108.2 min (minor)/110.0 (major)	$[\alpha]_D^{25.0}$ = -19 (C= 0.10 in $CHCl_3$)	92
8	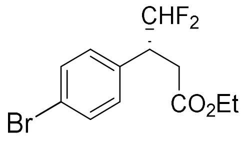	**GC-MS**, Chiraldex β-DM, 50 to 170 °C, 1 °C/min, 1.0 mL/min, t_R = 84.7 min (minor)/85.4 (major)	$[\alpha]_D^{25.0}$ = -16 (C= 0.10 in $CHCl_3$)	95
9	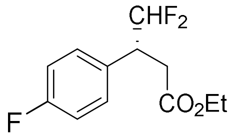	**SFC**, IF column, 5% MeOH, 2 ml/min, t_R = 2.52 min (major)/2.64 (minor)	$[\alpha]_D^{25.0}$ = -28 (C= 0.10 in $CHCl_3$)	98
10	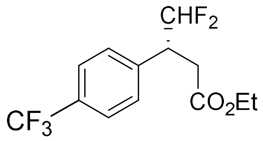	**SFC**, ADH column, 5% MeOH, 2 ml/min, t_R = 2.20 min (major)/2.28 (minor)	$[\alpha]_D^{25.0}$ = -22 (C= 0.1 in $CHCl_3$)	94
11	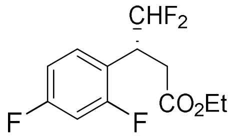	**SFC**, IF column, 5% MeOH, 2 ml/min, t_R = 4.28 min (minor)/4.44 (major)	$[\alpha]_D^{25.0}$ =-18 (C= 0.10 in $CHCl_3$)	97
12	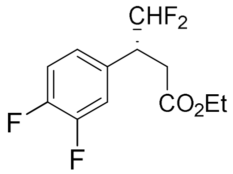	**SFC**, IF column, 5% MeOH, 2 ml/min, t_R = 4.95 min (major)/5.25 (minor)	$[\alpha]_D^{25.0}$ = -24 (C= 0.10 in $CHCl_3$)	93
13	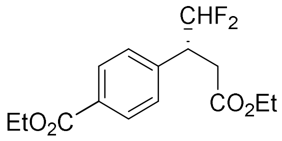	**SFC**, ADH column, 5% MeOH, 2 ml/min, t_R = 5.81 min (major)/7.79 (minor)	$[\alpha]_D^{25.0}$ = -17 (C= 0.10 in $CHCl_3$)	72
14	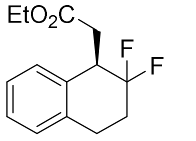	**SFC**, ASH column, 5% MeOH, 2 ml/min, t_R = 2.48 min (minor)/2.59 (major)	$[\alpha]_D^{25.0}$ = +38 (C= 0.10 in $CHCl_3$)	76

15	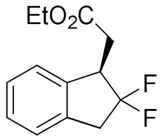	**SFC**, AYH column, 5% MeOH, 2 ml/min, t_R = 3.27 min (only)	$[\alpha]_D^{25.0}$ = + 41 (C= 0.77 in $CHCl_3$)	99
16	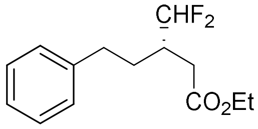	**SFC**, IF column, 5% MeOH, 2 ml/min, t_R = 10.31 min (minor)/11.10 min (major)	$[\alpha]_D^{25.0}$ = -9 (C= 0.73 in $CHCl_3$)	90
17	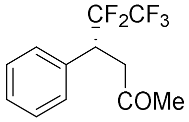	**GC-MS**, Hydrodex β-DM, 50 - 180 ºC, 1 degree/min, t_R = 42.7 min (minor) /43.4 (major)	$[\alpha]_D^{25.0}$ = -28 (C= 0.10 in $CHCl_3$)	81
18	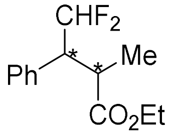	**GC-MS**, Hydrodex β-DM, 100 ºC iso 100 min, 1 degree/min, t_R = 36.4 min (minor) /38.3 (major)	$[\alpha]_D^{25.0}$ = -40 (C= 0.45in $CHCl_3$)	91
19	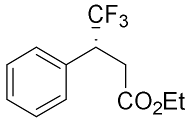	**GC-MS**, Hydrodex β-DM, 80 ºC iso 80 min, 1 degree/min, t_R = 62.9 min (minor) /65.2 (major)	$[\alpha]_D^{25.0}$ =-22 (C= 0.30 in $CHCl_3$)	92
20	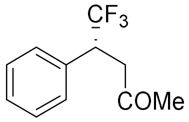	**SFC**, OJH column, 5% MeOH, 2 ml/min, t_R = 2.62 min (major)/2.75 min (minor)	$[\alpha]_D^{25.0}$ =-27 (C= 0.10 in $CHCl_3$)	89
21	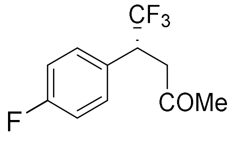	**GC-MS**, Hydrodex β-DM, 60 ºC iso 150 min, 1 degree/min, t_R = 56.3 min (minor) /58.1 (major)	$[\alpha]_D^{25.0}$ =-19 (C= 0.10 in $CHCl_3$)	87
22	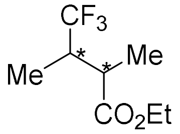	**GC-MS**, Hydrodex β-DM, 30 ºC iso, 1 degree/min, t_R = 19.9 min (major) /22.6 (minor)	$[\alpha]_D^{25.0}$ =-6 (C= 0.10 in $CHCl_3$)	88
23	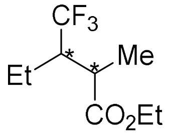	**GC-MS**, Hydrodex β-DM, 30 ºC iso, 1 degree/min, t_R = 42.3 min (major) /49.7 (minor)	$[\alpha]_D^{25.0}$ = -44 (C= 0.10 in $CHCl_3$)	82
24	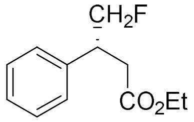	**SFC**, IF column, 5% MeOH, 2 ml/min, t_R = 3.42 min (major)/3.71 min (minor)	$[\alpha]_D^{25.0}$ = -15 (C= 0.10 in $CHCl_3$)	84

25	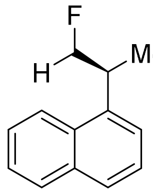	**GC-MS**, Hydrodex β-6TBDM, 120 ºC iso 70 min, 1 degree/min, t_R = 59.0 min (minor) /61.0 (major)	$[\alpha]_D^{25.0}$ = +23 (C= 0.10 in $CHCl_3$)	94
26	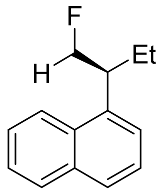	**HPLC**, ODH column, 2% iPrOH/Hexane, 0.5 ml/min, t_R = 11.46 min (major)/12.18 min (minor)	$[\alpha]_D^{25.0}$ = +14 (C= 0.10 in $CHCl_3$)	94
27	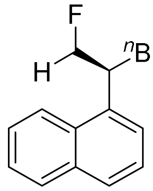	**HPLC**, ODH column, 2% iPrOH/Hexane, 0.5 ml/min, t_R = 10.79 min (major)/11.38 min (minor)	$[\alpha]_D^{25.0}$ = +3 (C= 0.10 in $CHCl_3$)	95
28	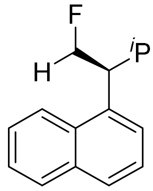	**HPLC**, ODH column, 2% iPrOH/Hexane, 0.5 ml/min, t_R = 11.05 min (major)/13.67 min (minor)	$[\alpha]_D^{25.0}$ = +12 (C= 0.10 in $CHCl_3$)	98
29	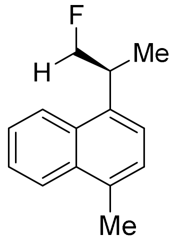	**GC-MS**, Hydrodex β-6TBDM, 120 ºC iso 300 min, 1 degree/min, t_R = 123.9 min (minor) /131.0 (major)	$[\alpha]_D^{25.0}$ = +21 (C= 0.10 in $CHCl_3$)	90
30	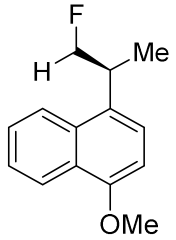	**GC-MS**, Hydrodex β-6TBDM, 120 ºC iso 300 min, 1 degree/min, t_R = 266.3 min (minor) /285.6 (major)	$[\alpha]_D^{25.0}$ = +14 (C= 0.10 in $CHCl_3$)	94
31	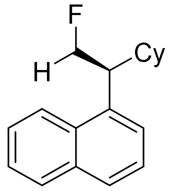	**HPLC**, ODH column, 2% iPrOH/Hexane, 0.5 ml/min, t_R = 12.35 min (major)/16.35 min (minor)	$[\alpha]_D^{25.0}$ = +17 (C= 0.10 in $CHCl_3$)	98
32	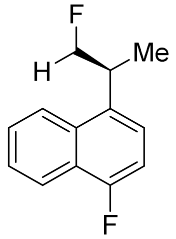	**GC-MS**, Hydrodex β-6TBDM, 90 ºC iso 120 min, 1 degree/min, t_R = 65.3 min (minor) /68.3 (major)	$[\alpha]_D^{25.0}$ = +15 (C= 0.10 in $CHCl_3$)	92

33	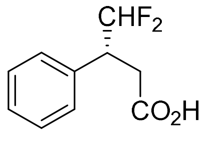	**SFC**, ADH column, 10% MeOH, 2 ml/min, t_R = 6.12 min (major)/6.65 (minor)	$[\alpha]_D^{25.0}$ = -20 (C= 0.10 in $CHCl_3$)	95
34	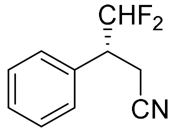	**SFC**, ADH column, 5% MeOH, 2 ml/min, t_R = 4.32 min (major)/4.71 (minor)	$[\alpha]_D^{25.0}$ = -24 (C= 0.10 in $CHCl_3$)	95
35	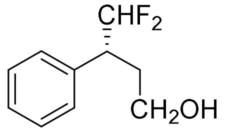	**SFC**, ASH column, 10% MeOH, 2 ml/min, t_R = 4.91 min (major)/5.35 (minor)	$[\alpha]_D^{25.0}$ = -16 (C= 0.10 in $CHCl_3$)	95
36	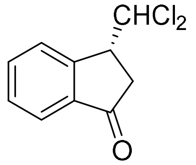	**SFC**, IA column, 5% MeOH, 2 ml/min, t_R = 17.42 min (major)/19.10 (minor)	$[\alpha]_D^{25.0}$ = +28 (C= 0.10 in $CHCl_3$)	95
37	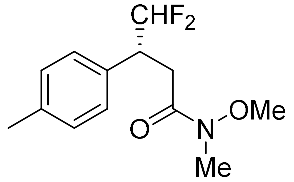	**SFC**, ASH column, 5% MeOH, 2 ml/min, t_R = 3.38 min (minor)/3.89 (major)	$[\alpha]_D^{25.0}$ = +25 (C= 0.10 in $CHCl_3$)	95
38	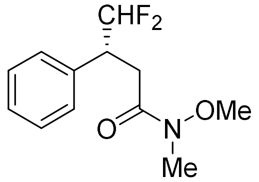	**SFC**, ASH column, 10% MeOH, 2 ml/min, t_R = 2.80 min (minor)/3.09 (major)	$[\alpha]_D^{25.0}$ = +18 (C= 0.10 in $CHCl_3$)	95
39	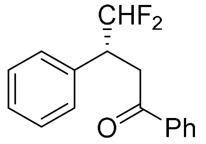	**SFC**, OJH column, 5% MeOH, 2 ml/min, t_R = 9.51 min (minor)/11.96 (major)	$[\alpha]_D^{25.0}$ = +31 (C= 0.10 in $CHCl_3$)	94
40	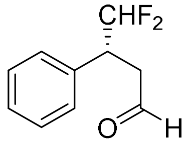	**GC-MS**, Chiraldex β-DM, 50 °C iso 200 min, 1 degree/min, t_R = 184.0 min (minor) /187.2 (major)	$[\alpha]_D^{25.0}$ = -24 (C= 0.10 in $CHCl_3$)	95
41	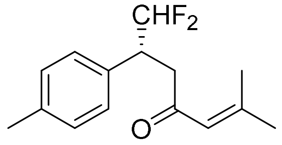	**SFC**, ASH column, 5% MeOH, 2 ml/min, t_R = 4.25 min (major)/4.87 (minor)	$[\alpha]_D^{25.0}$ = -28 (C= 0.10 in $CHCl_3$)	94
42	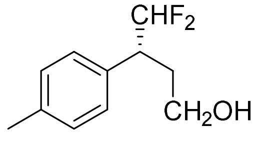	**SFC**, IF column, 5% MeOH, 2 ml/min, t_R = 19.54 min (minor)/20.18 (major)	$[\alpha]_D^{25.0}$ = -19 (C= 0.10 in $CHCl_3$)	94

8. Absolute configuration determination

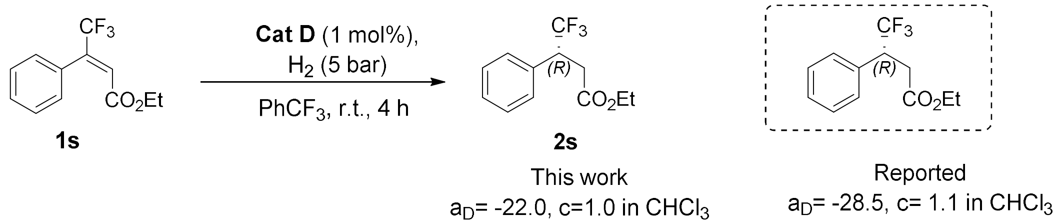

Substrate **1s** was hydrogenated to obtain **2s** (99% yield, 92% *ee*). The optical rotations were $[\alpha]^{25}_{D}$= -22.0 (c = 1, $CHCl_3$). This was confirmed to be the (*R*)-configuration for the product **2s** by comparison with the optical rotation data with literature.[4]

9. NMR Spectra

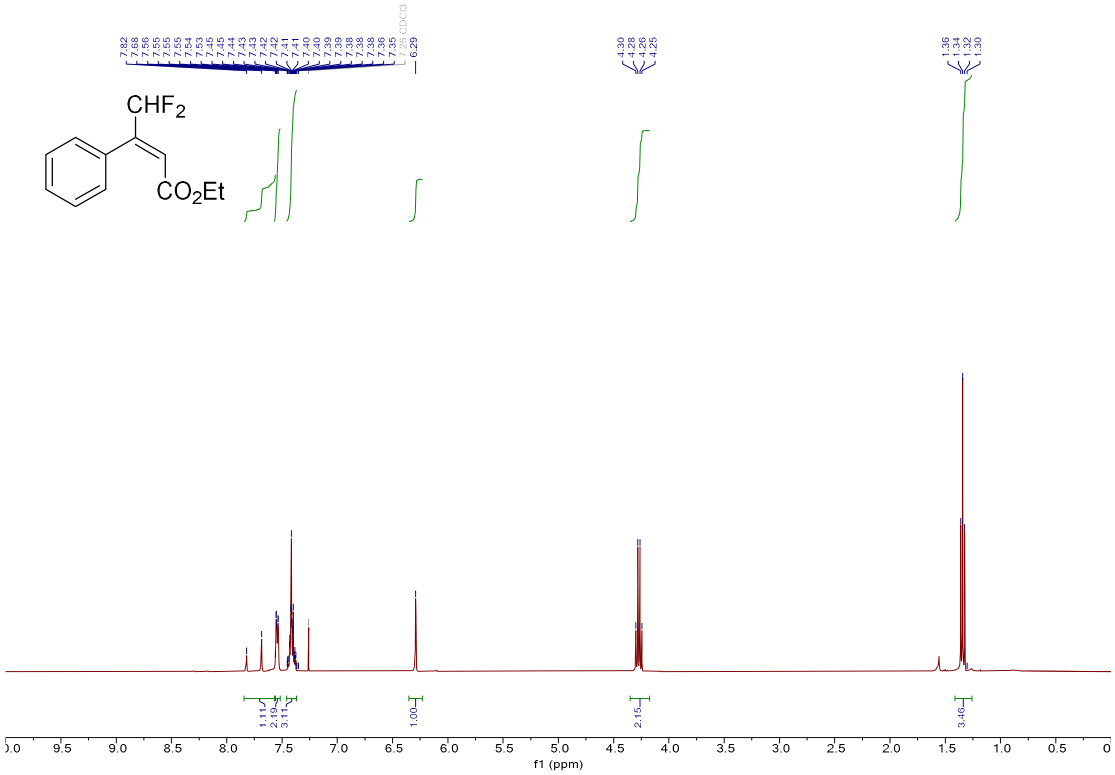

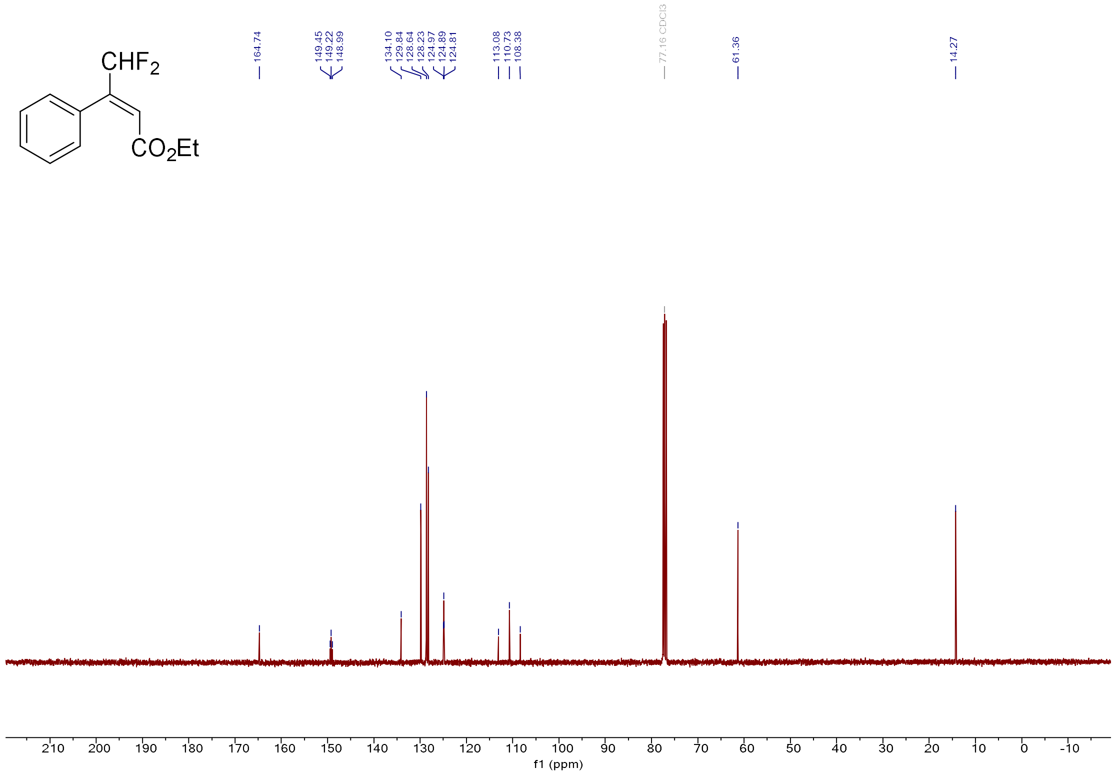

YJ-20201011-7-54-2-CF2.11.fid

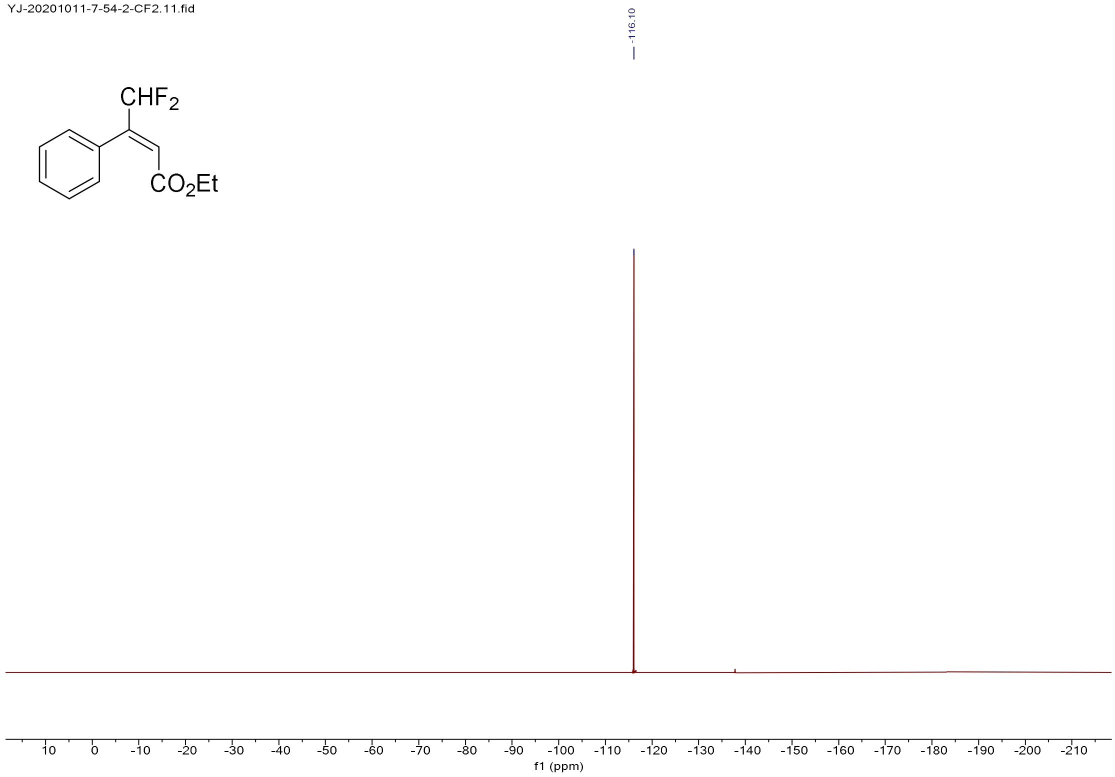

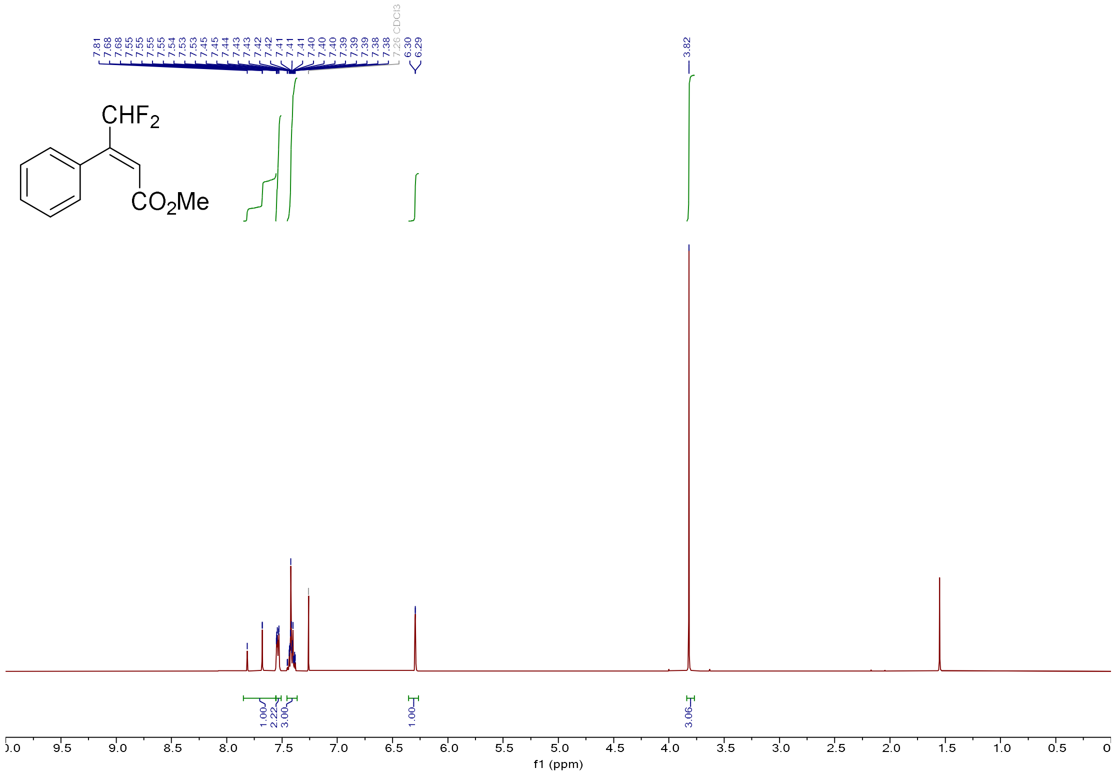

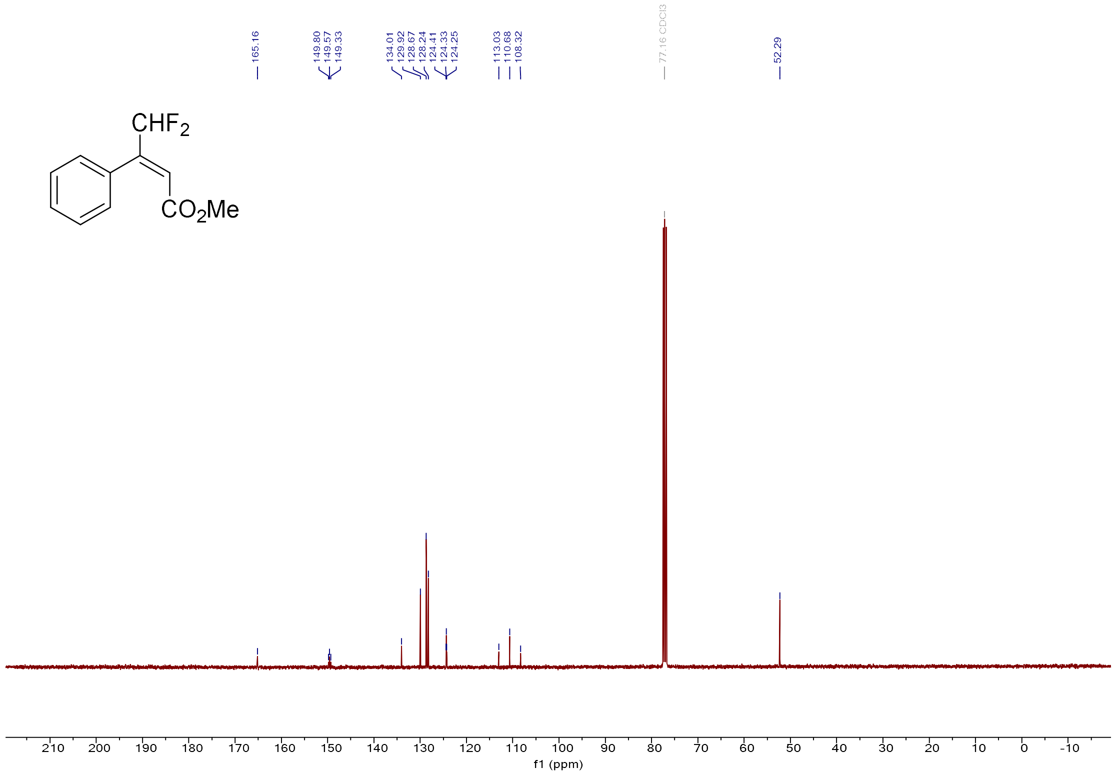
165.16
149.80
149.57
149.33
134.01
129.92
128.67
128.24
124.41
124.33
124.25
113.03
110.68
108.32
77.16 CDCl3
52.29
CHF2
CO2Me
f1 (ppm)

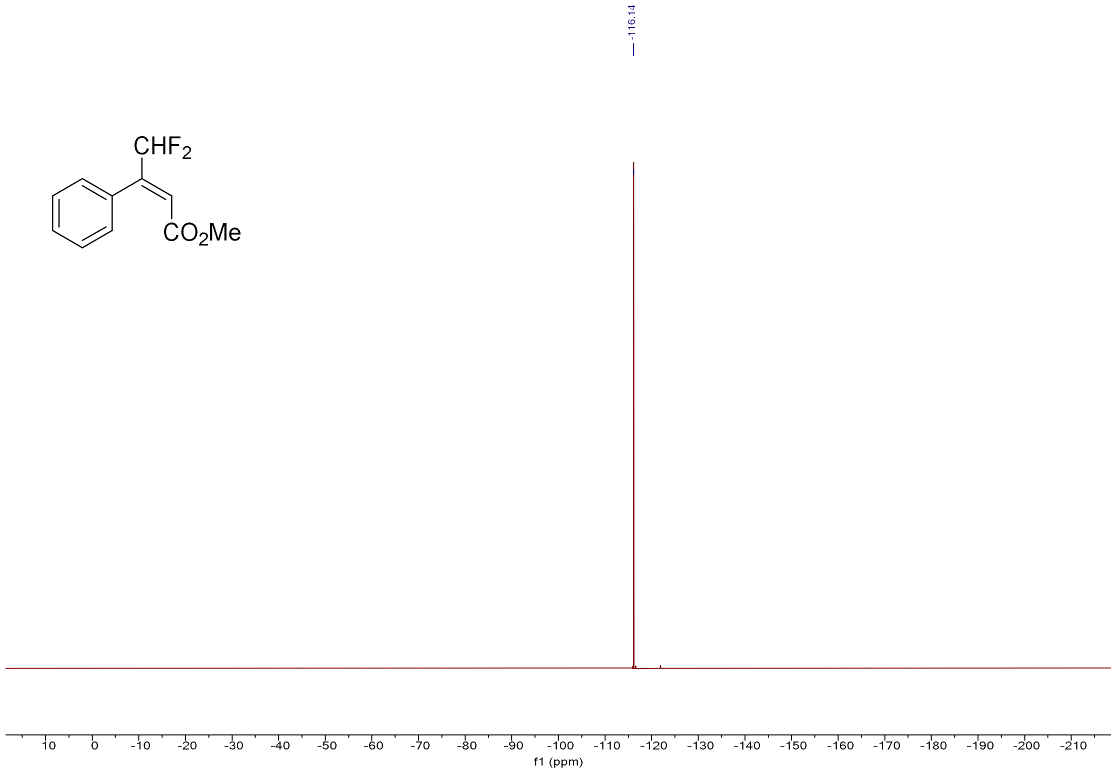
-116.14
CHF2
CO2Me
f1 (ppm)

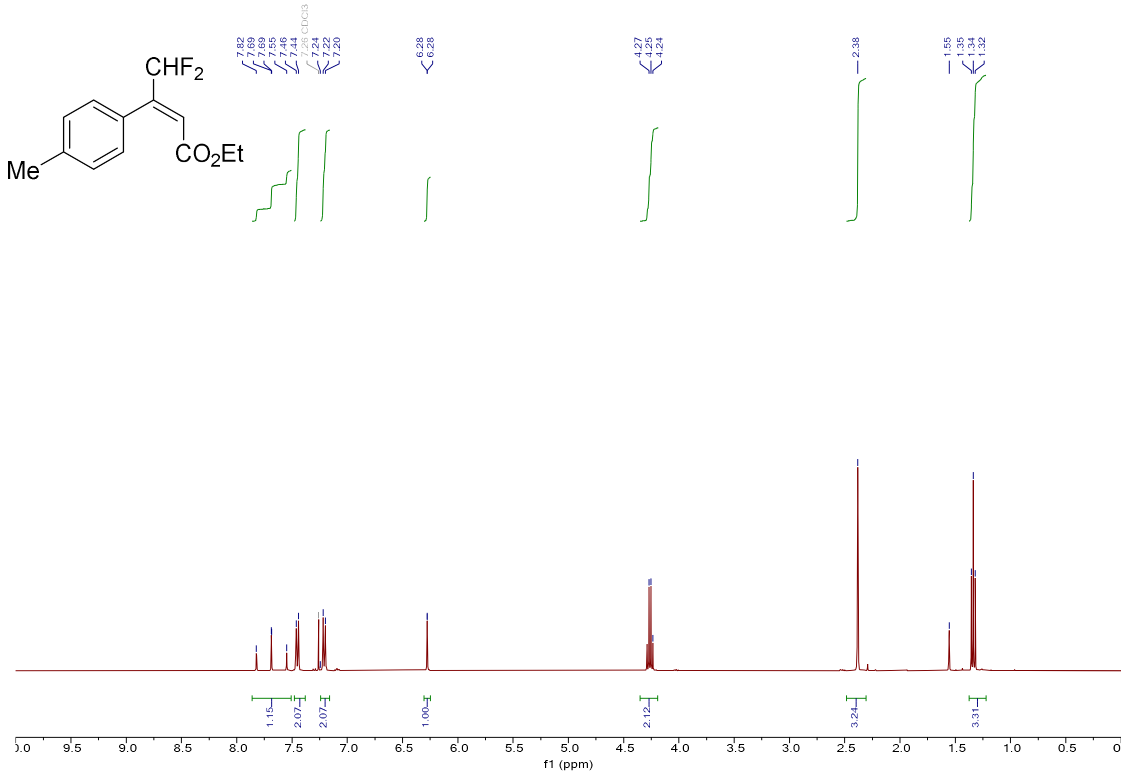
CHF2
Me
CO2Et
7.82
7.69
7.69
7.55
7.46
7.44
7.26 CDCl3
7.24
7.22
7.20
6.28
6.28
4.27
4.25
4.24
2.38
1.55
1.35
1.34
1.32
1.15
2.07
2.07
1.00
2.12
3.24
3.31
f1 (ppm)

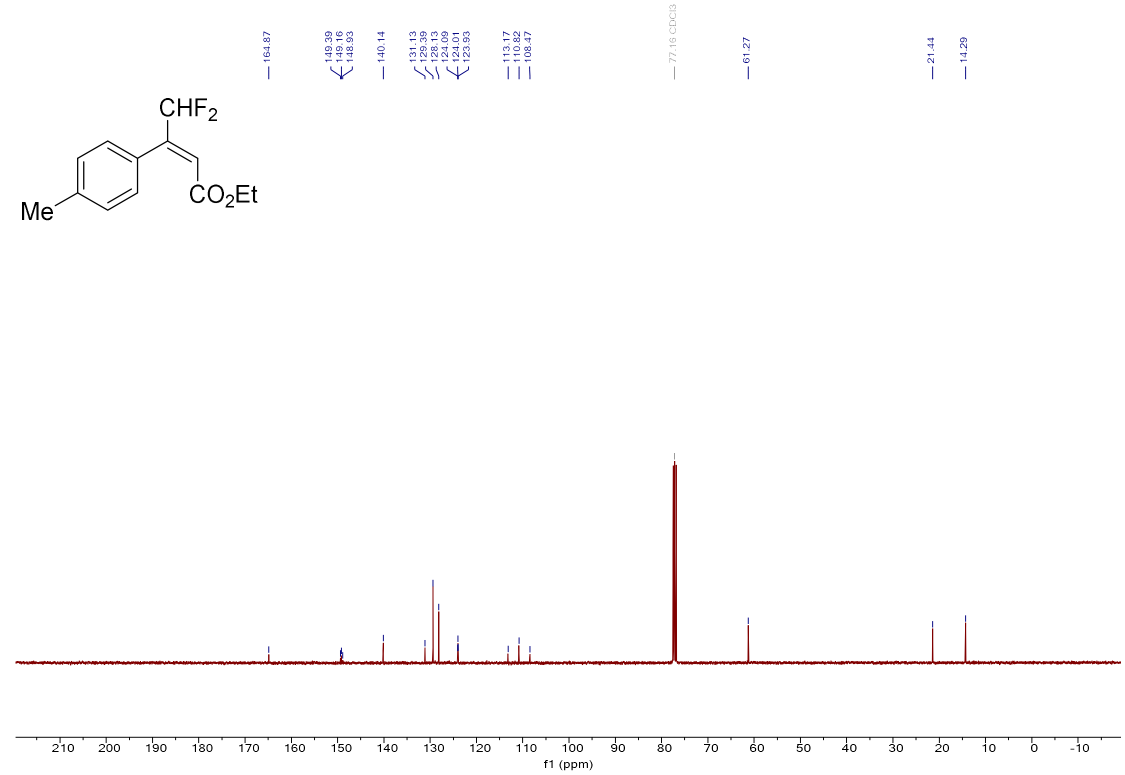
164.87
149.39
149.16
148.93
140.14
131.13
129.39
128.13
124.09
124.01
123.93
113.17
110.82
108.47
77.16 CDCl3
61.27
21.44
14.29
CHF2
Me
CO2Et
f1 (ppm)

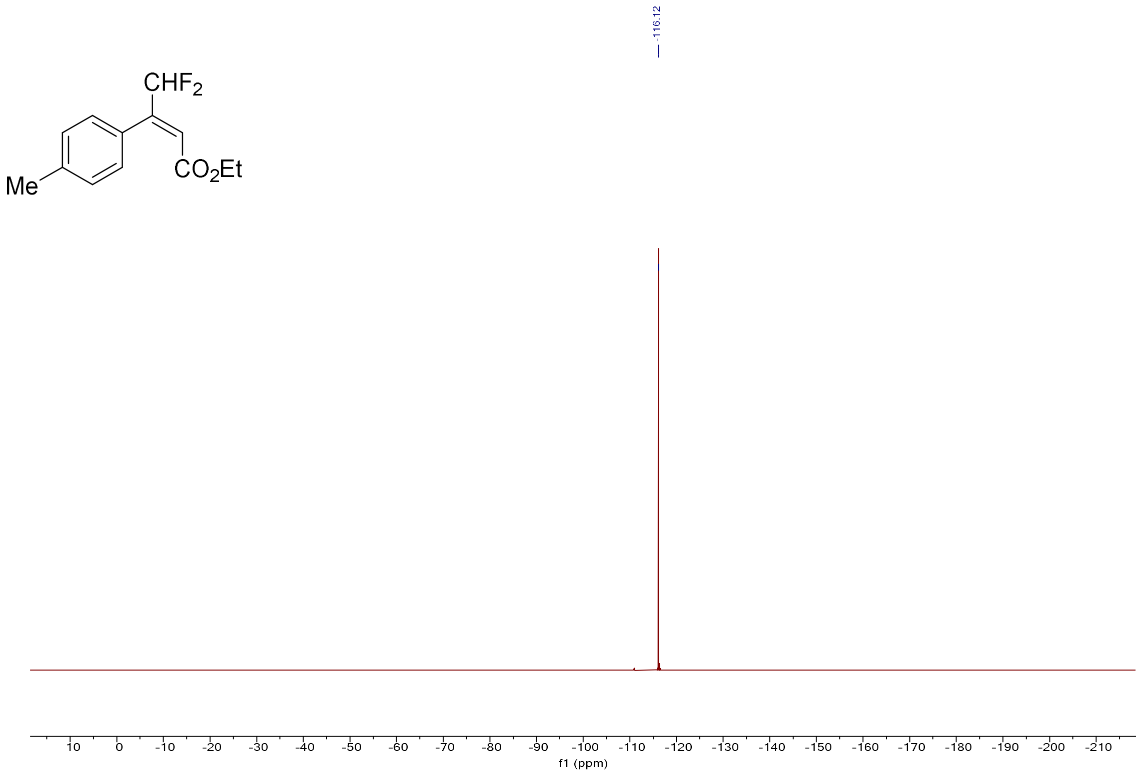

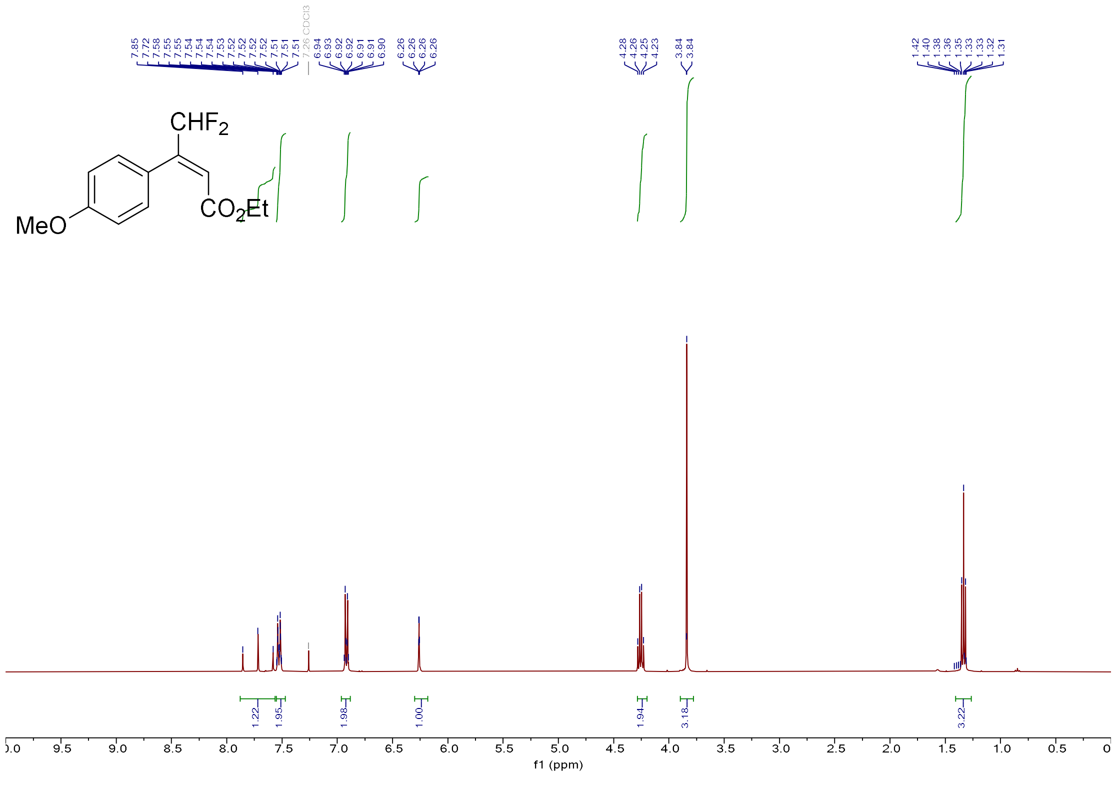

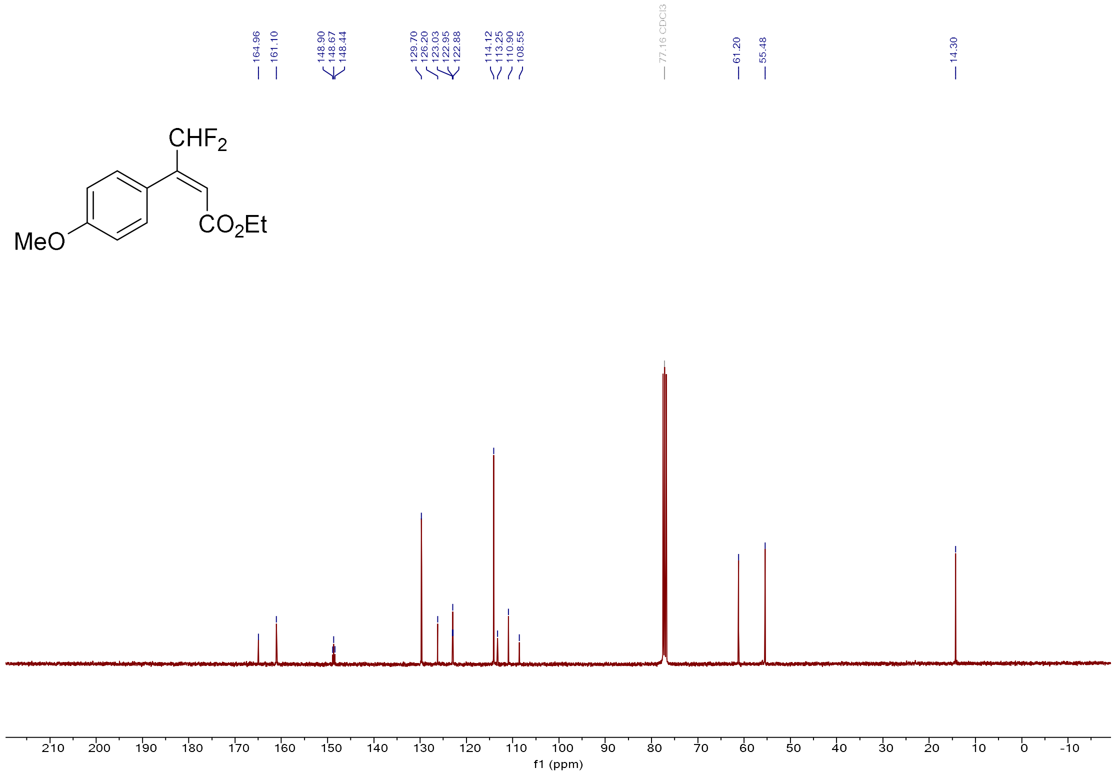
164.96
161.10
148.90
148.67
148.44
129.70
126.20
123.03
122.95
122.88
114.12
113.25
110.90
108.55
77.16 CDCl3
61.20
55.48
14.30
CHF2
MeO
CO2Et
f1 (ppm)

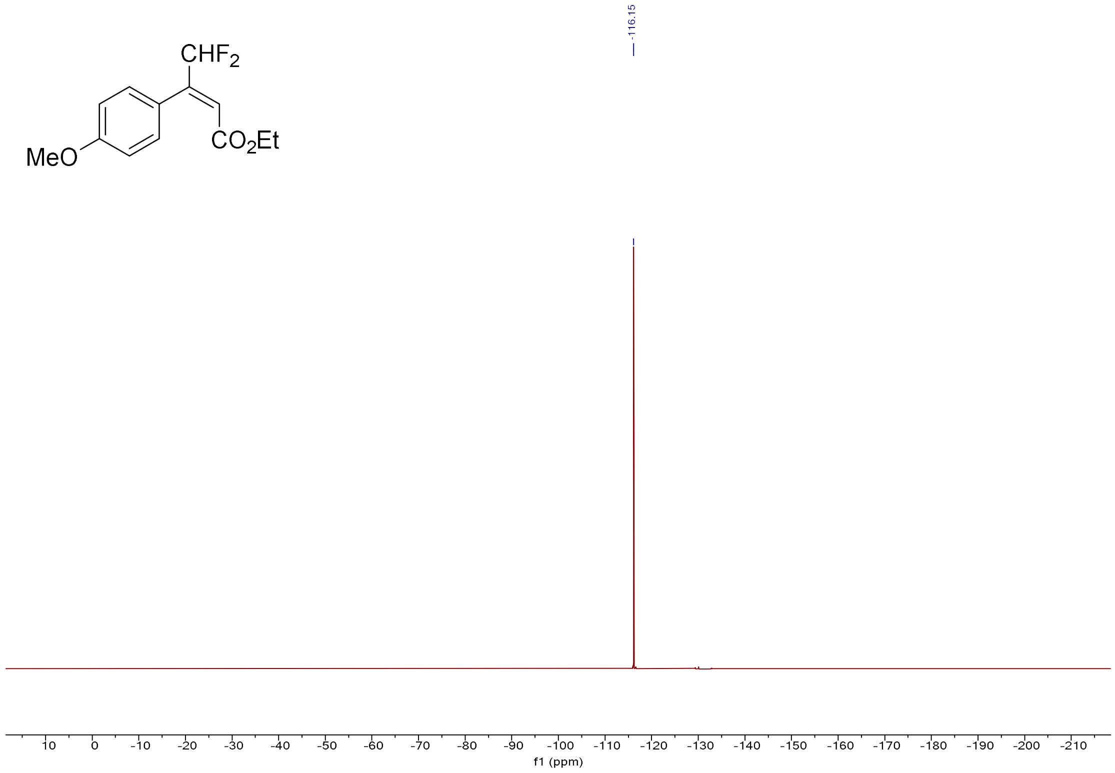
-116.15
CHF2
MeO
CO2Et
f1 (ppm)

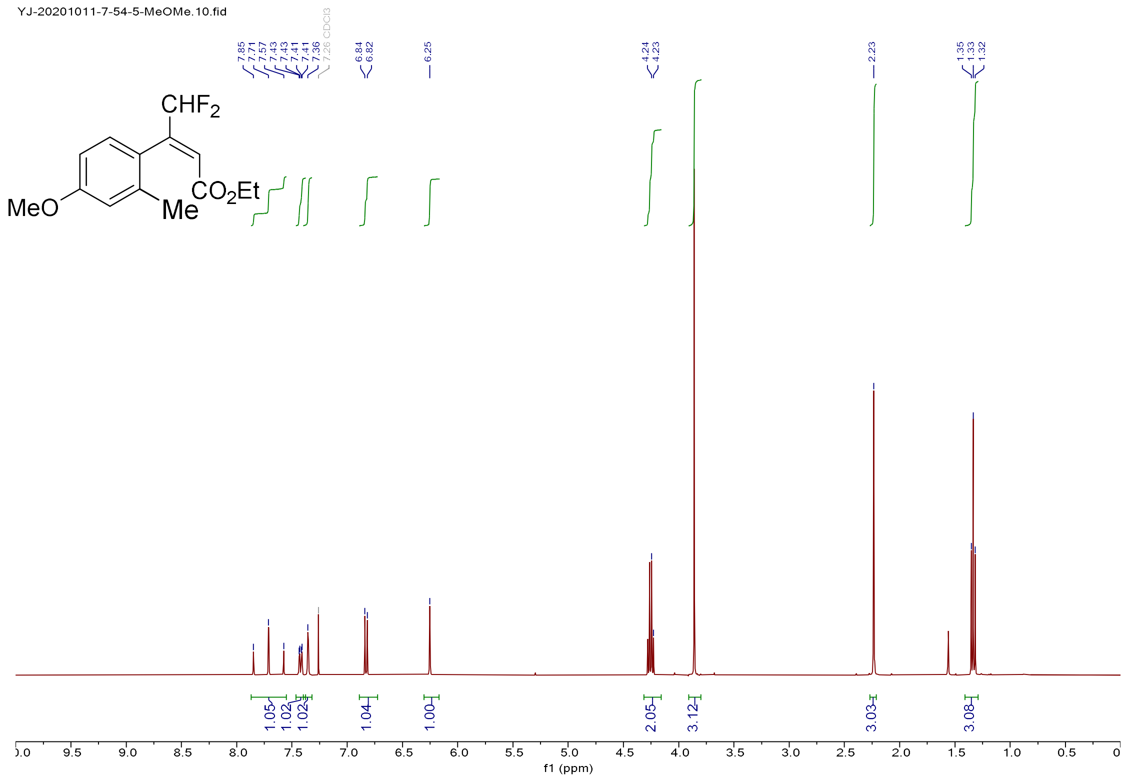
YJ-20201011-7-54-5-MeOMe.10.fid
CHF2
MeO
Me
CO2Et
f1 (ppm)

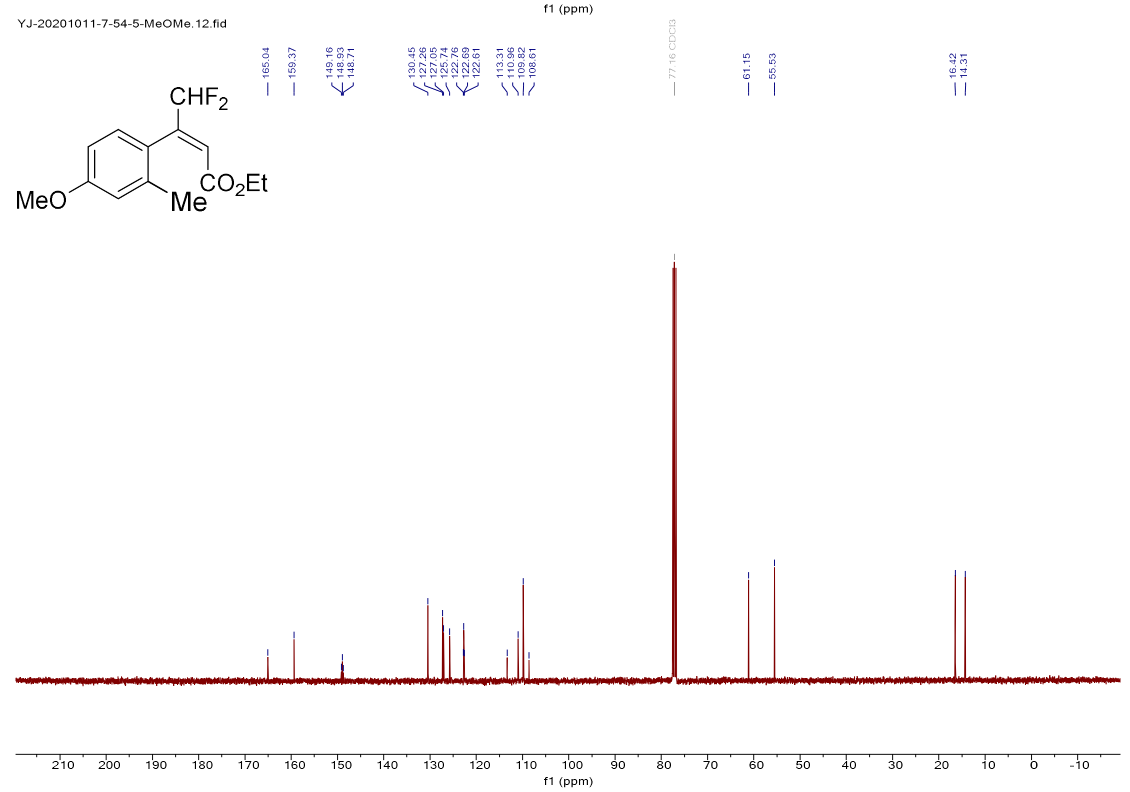
YJ-20201011-7-54-5-MeOMe.12.fid
CHF2
MeO
Me
CO2Et
f1 (ppm)

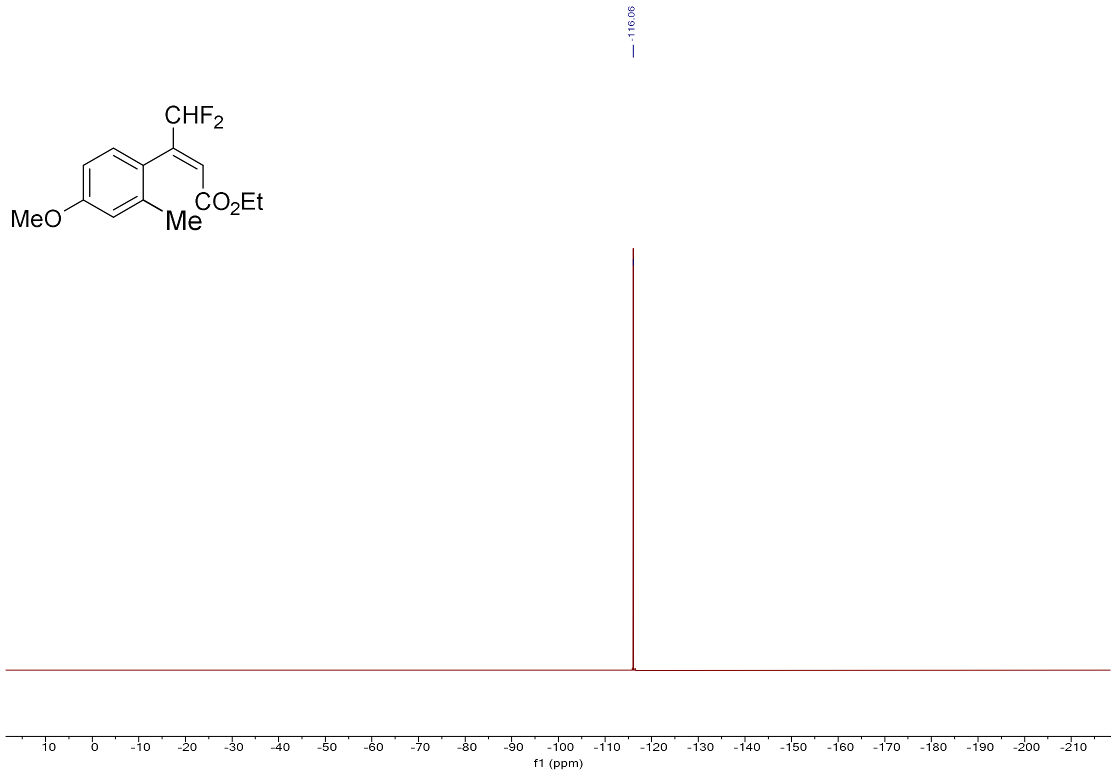
CHF2
CO2Et
MeO
Me
-116.06
10 0 -10 -20 -30 -40 -50 -60 -70 -80 -90 -100 -110 -120 -130 -140 -150 -160 -170 -180 -190 -200 -210
f1 (ppm)

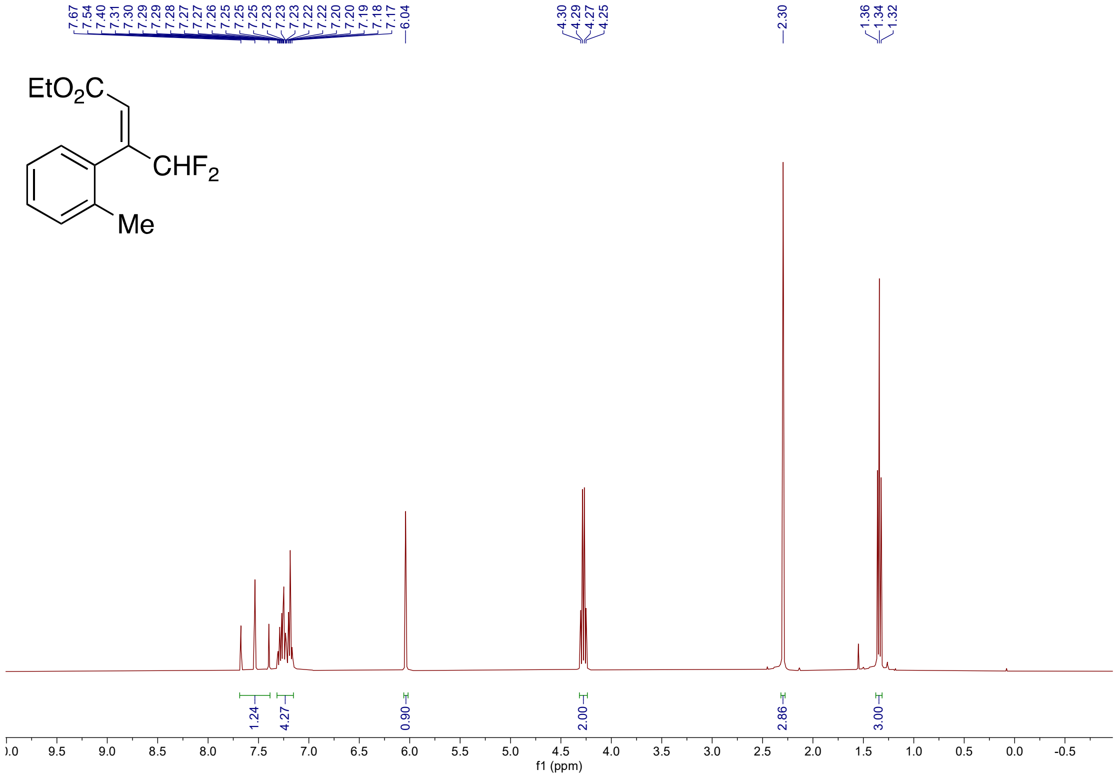
7.67 7.54 7.40 7.31 7.30 7.29 7.29 7.28 7.27 7.27 7.26 7.25 7.25 7.25 7.23 7.23 7.23 7.22 7.22 7.20 7.20 7.19 7.18 7.17
6.04
4.30 4.29 4.27 4.25
2.30
1.36 1.34 1.32
EtO2C
CHF2
Me
1.24
4.27
0.90
2.00
2.86
3.00
9.5 9.0 8.5 8.0 7.5 7.0 6.5 6.0 5.5 5.0 4.5 4.0 3.5 3.0 2.5 2.0 1.5 1.0 0.5 0.0 -0.5
f1 (ppm)

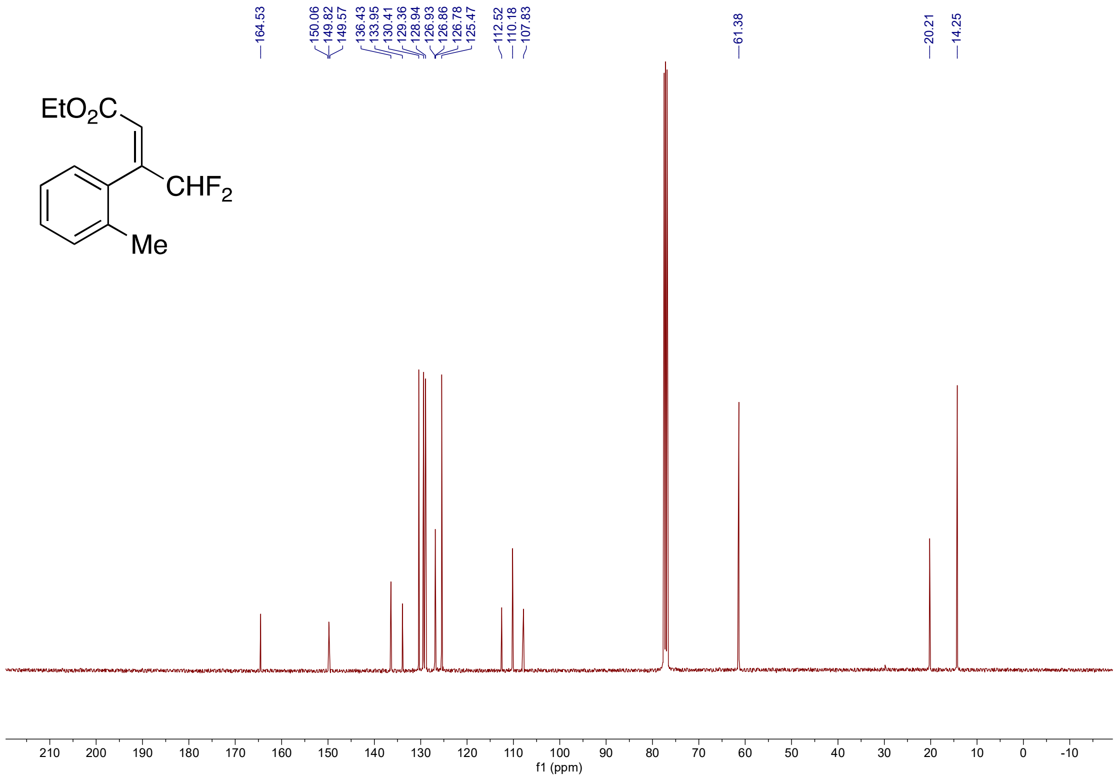
164.53
150.06
149.82
149.57
136.43
133.95
130.41
129.36
128.94
126.93
126.86
126.78
125.47
112.52
110.18
107.83
61.38
20.21
14.25
EtO2C
CHF2
Me
210 200 190 180 170 160 150 140 130 120 110 100 90 80 70 60 50 40 30 20 10 0 -10
f1 (ppm)

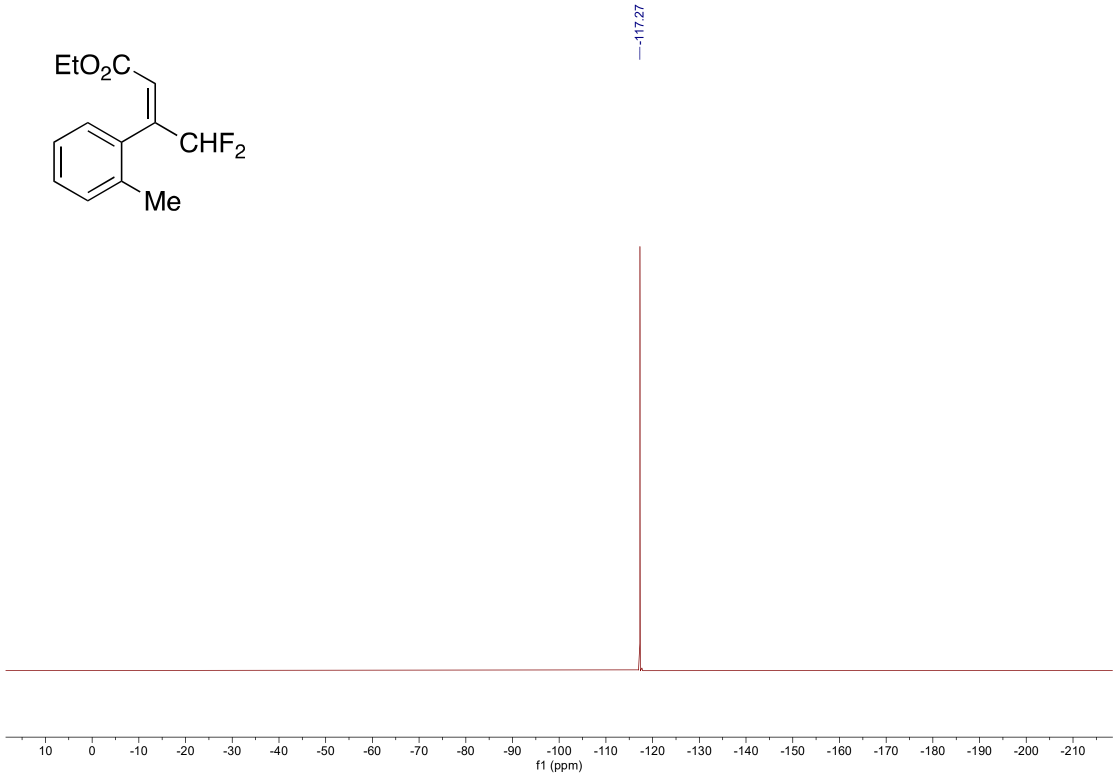
-117.27
EtO2C
CHF2
Me
10 0 -10 -20 -30 -40 -50 -60 -70 -80 -90 -100 -110 -120 -130 -140 -150 -160 -170 -180 -190 -200 -210
f1 (ppm)

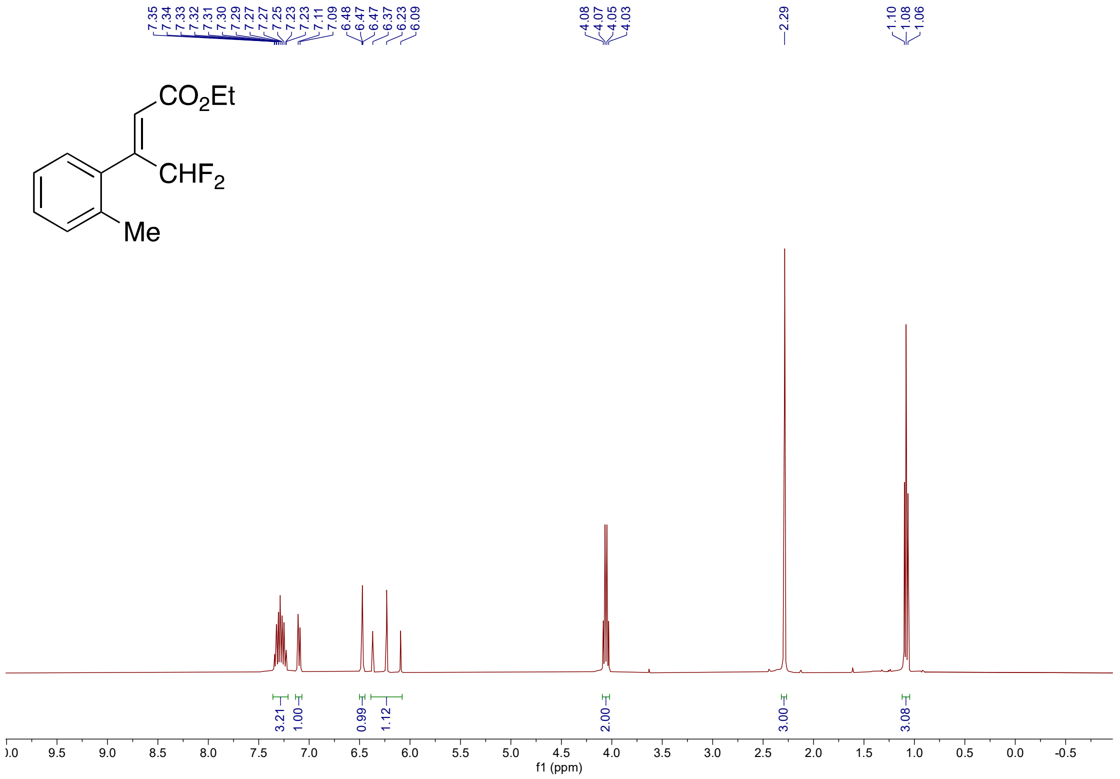
CO2Et
CHF2
Me
7.35
7.34
7.33
7.32
7.31
7.30
7.29
7.27
7.27
7.25
7.23
7.23
7.23
7.11
7.09
6.48
6.47
6.47
6.37
6.23
6.09
4.08
4.07
4.05
4.03
2.29
1.10
1.08
1.06
3.21
1.00
0.99
1.12
2.00
3.00
3.08
f1 (ppm)

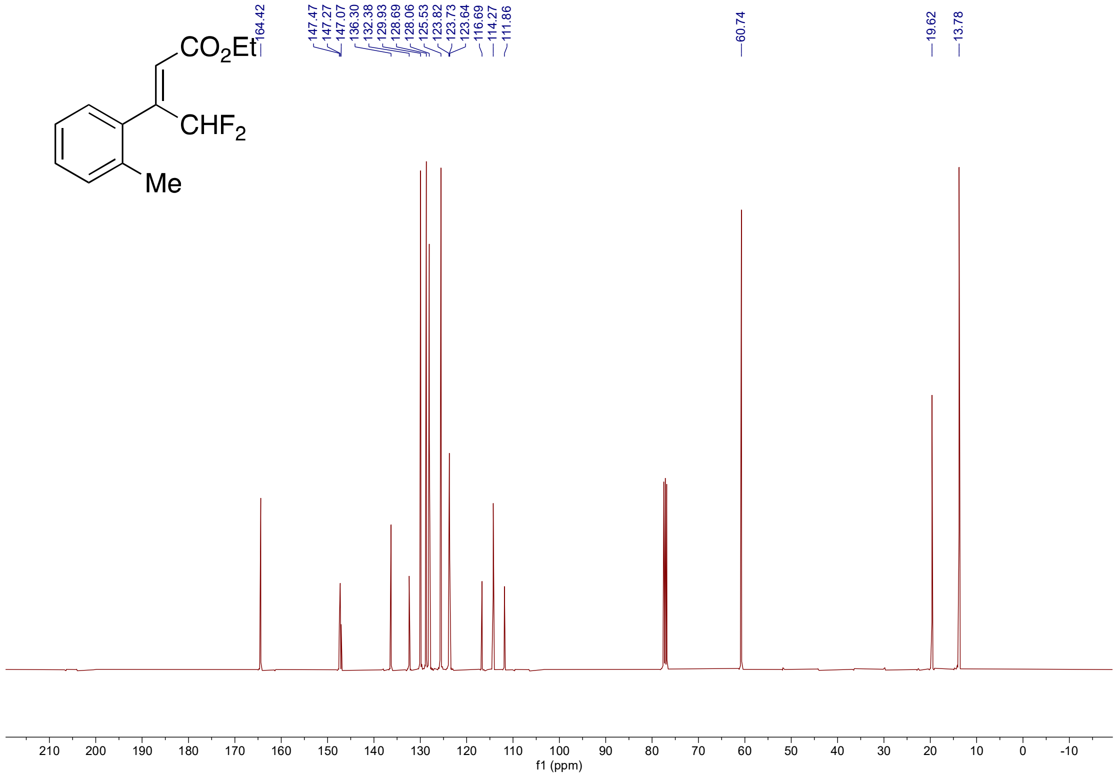
CO2Et
CHF2
Me
164.42
147.47
147.27
147.07
136.30
132.38
129.93
128.69
128.06
125.53
123.82
123.73
123.64
116.69
114.27
111.86
60.74
19.62
13.78
f1 (ppm)

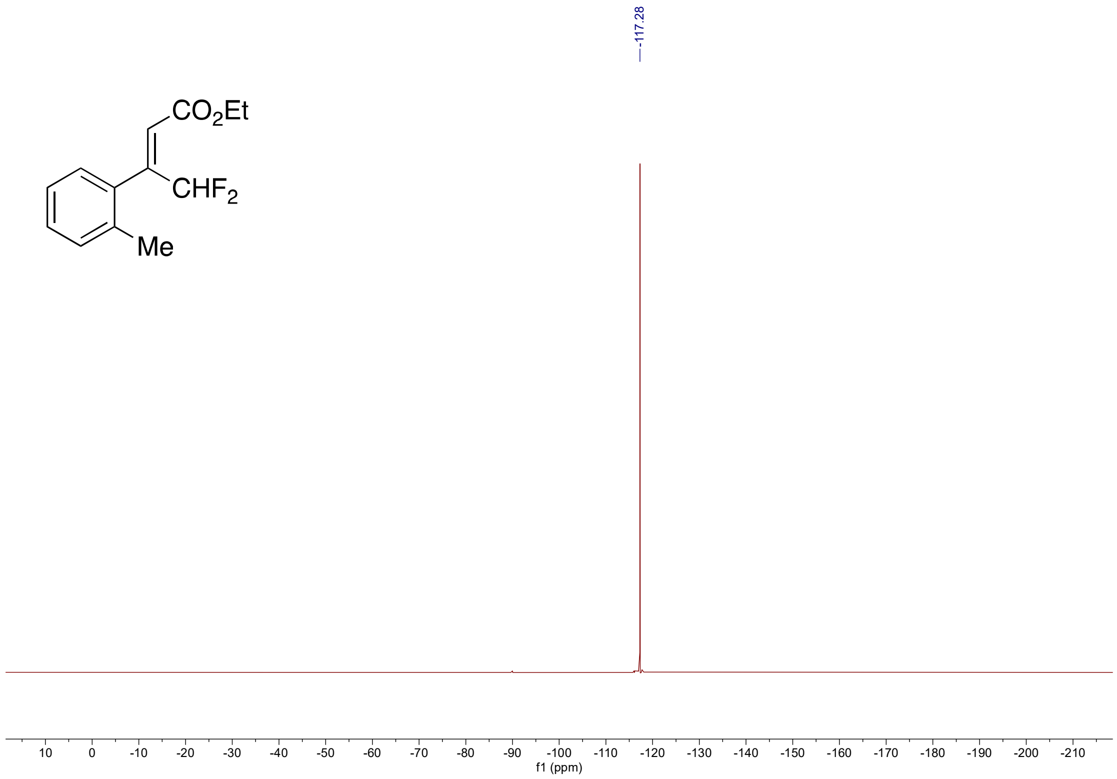
CO2Et
CHF2
Me
-117.28
f1 (ppm)

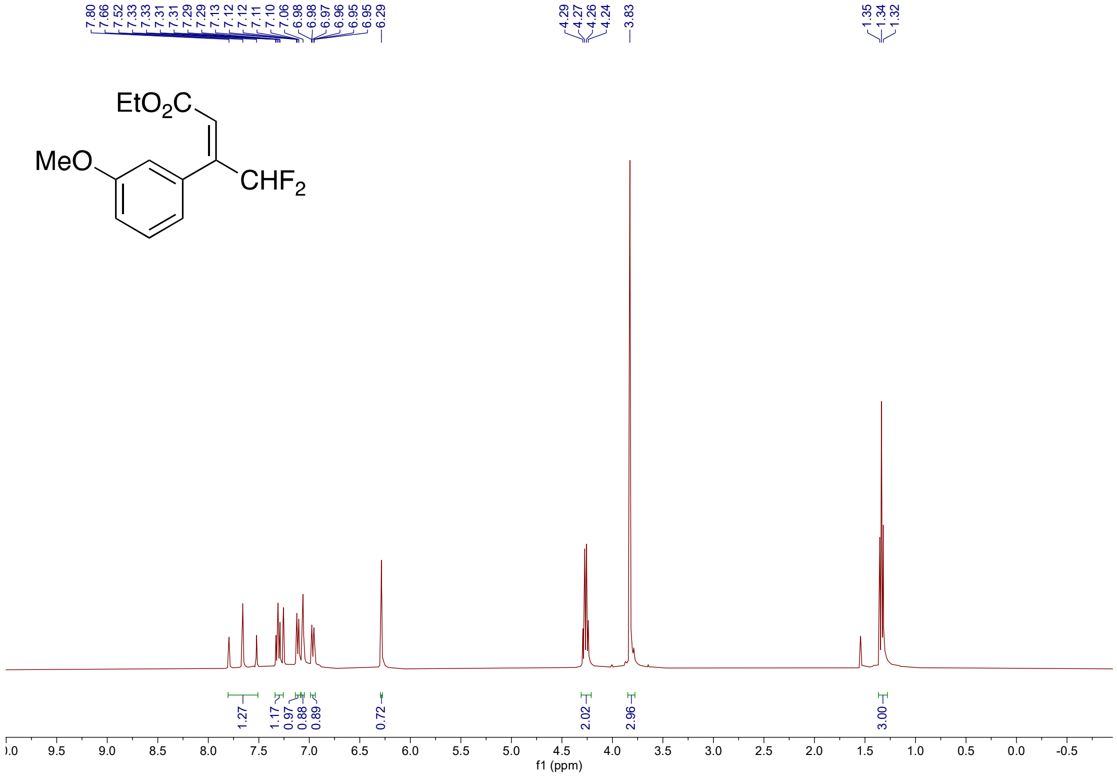
EtO2C
MeO
CHF2
7.80
7.66
7.52
7.33
7.33
7.31
7.31
7.29
7.29
7.13
7.12
7.12
7.11
7.10
7.06
6.98
6.98
6.97
6.96
6.95
6.95
6.29
4.29
4.27
4.26
4.24
3.83
1.35
1.34
1.32
1.27
1.17
0.97
0.88
0.89
0.72
2.02
2.96
3.00
f1 (ppm)

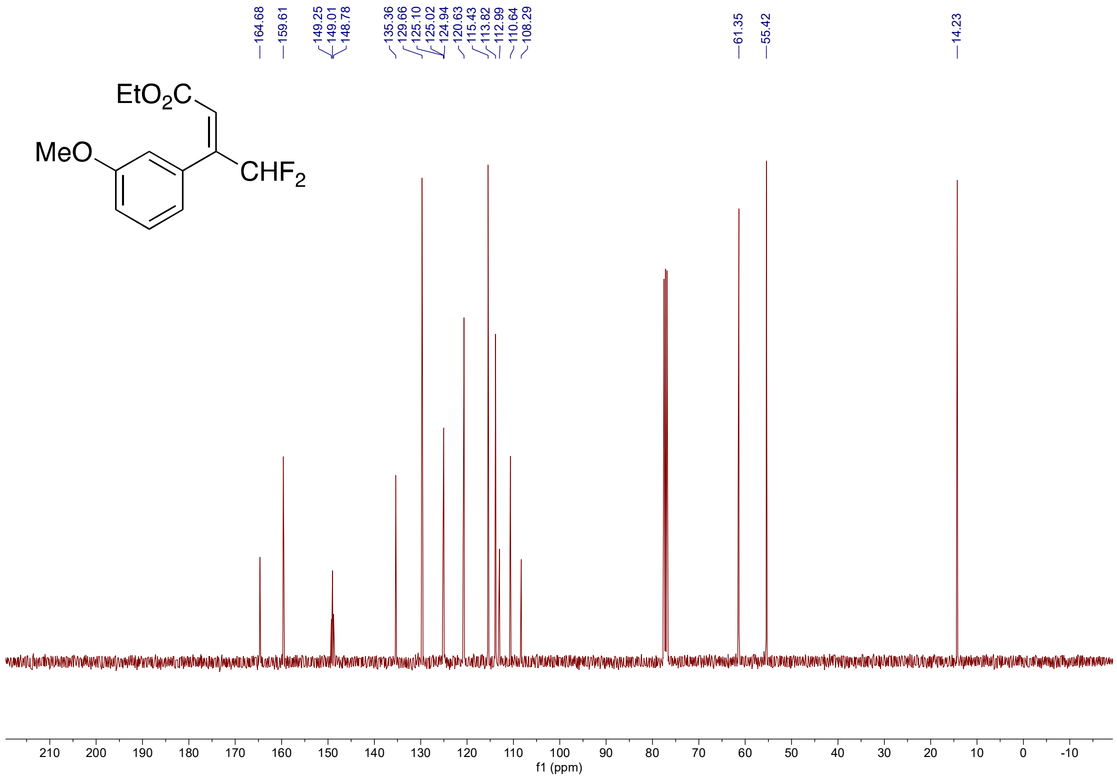
164.68
159.61
149.25
149.01
148.78
135.36
129.66
125.10
125.02
124.94
120.63
115.43
113.82
112.99
110.64
108.29
61.35
55.42
14.23
EtO_2C
MeO
CHF_2
f1 (ppm)

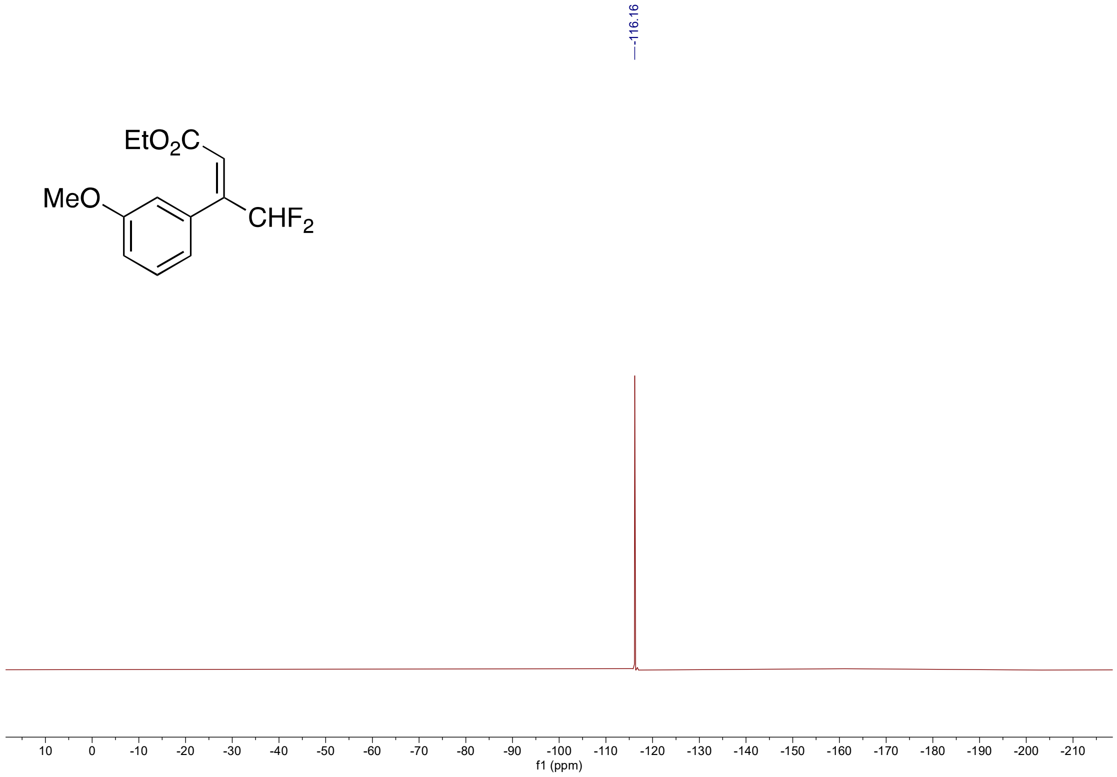
-116.16
EtO_2C
MeO
CHF_2
f1 (ppm)

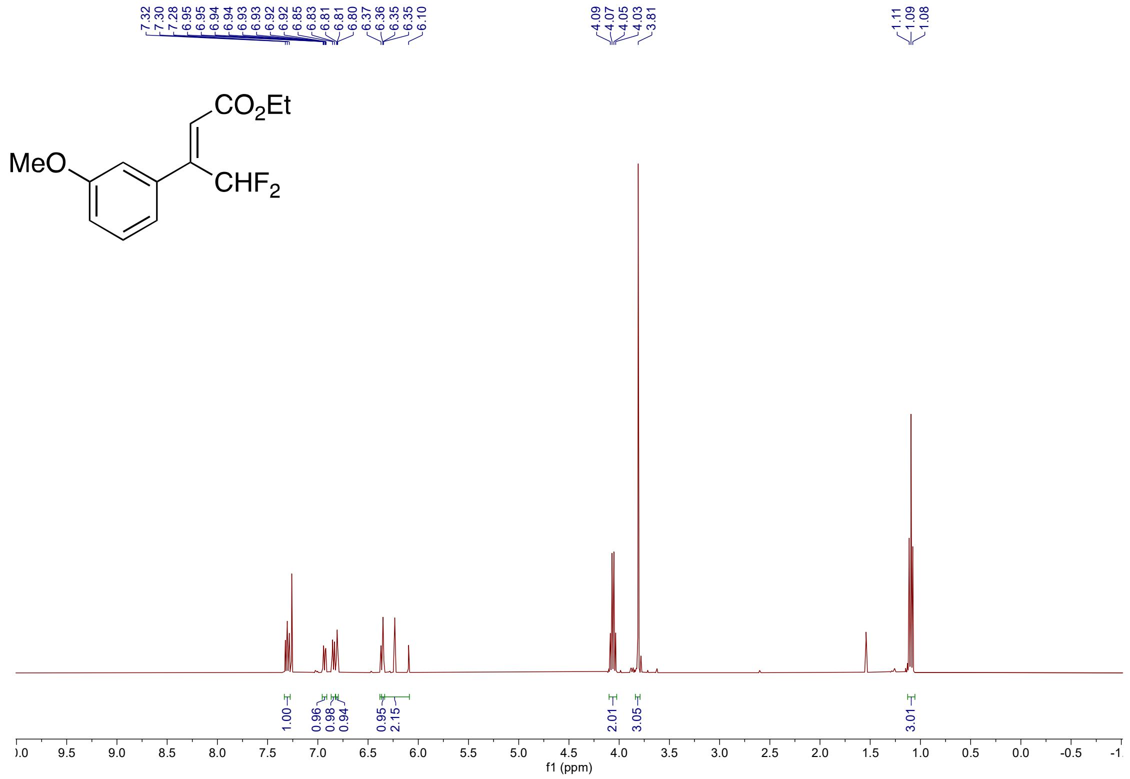
7.32
7.30
7.28
6.95
6.95
6.94
6.94
6.93
6.93
6.92
6.92
6.85
6.83
6.81
6.81
6.80
6.37
6.36
6.35
6.35
6.10
4.09
4.07
4.05
4.03
3.81
1.11
1.09
1.08
CO2Et
MeO
CHF2
1.00
0.96
0.98
0.94
0.95
2.15
2.01
3.05
3.01
f1 (ppm)

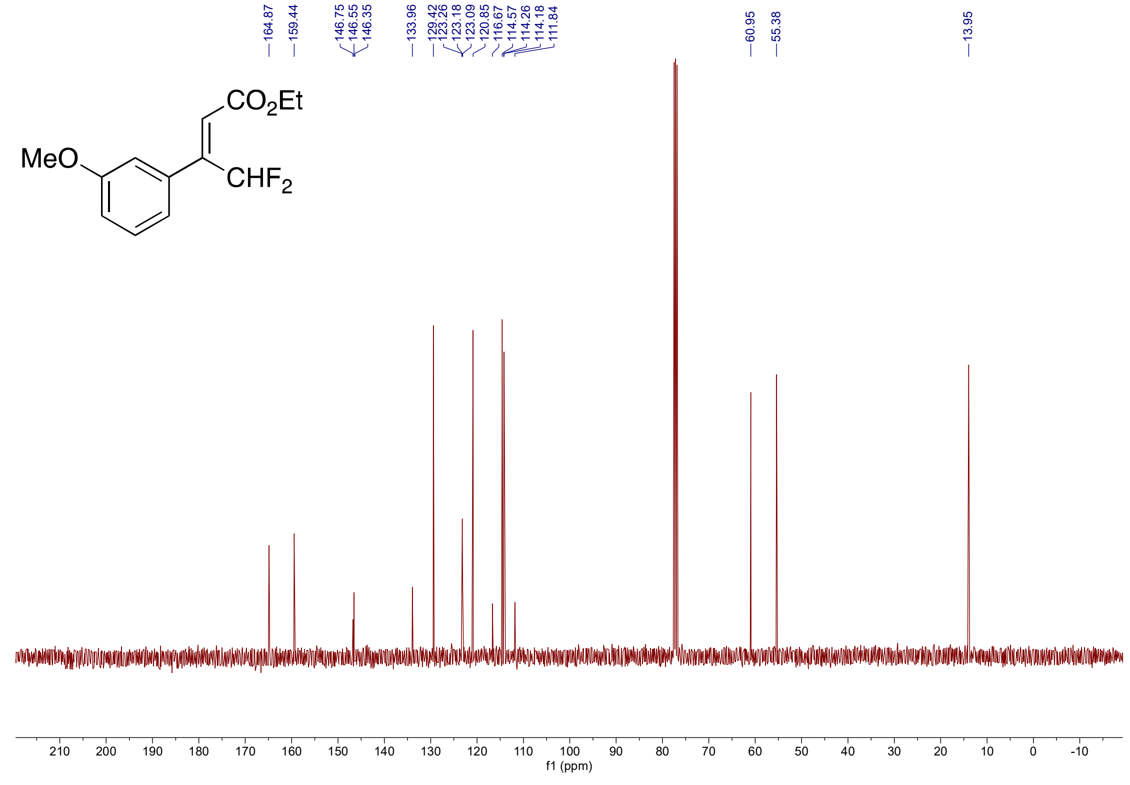
164.87
159.44
146.75
146.55
146.35
133.96
129.42
123.26
123.18
123.09
120.85
116.67
114.57
114.26
114.18
111.84
60.95
55.38
13.95
CO2Et
MeO
CHF2
f1 (ppm)

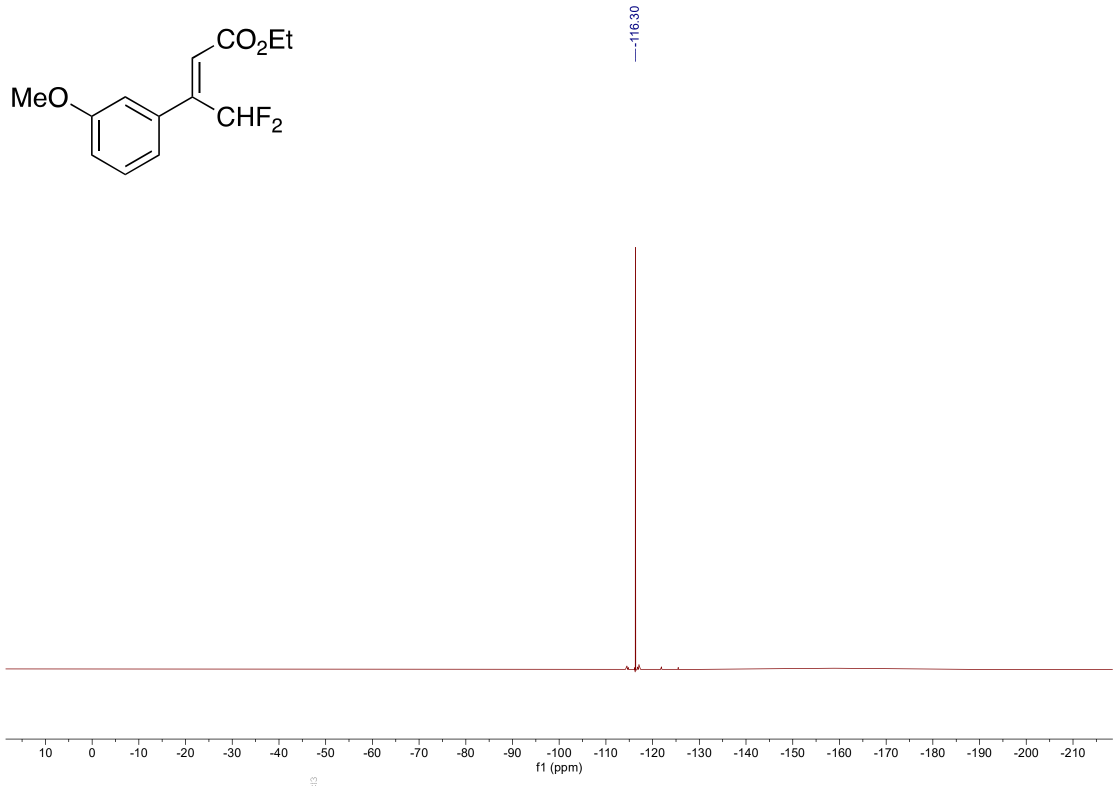

CO2Et
MeO
CHF2
-116.30
f1 (ppm)

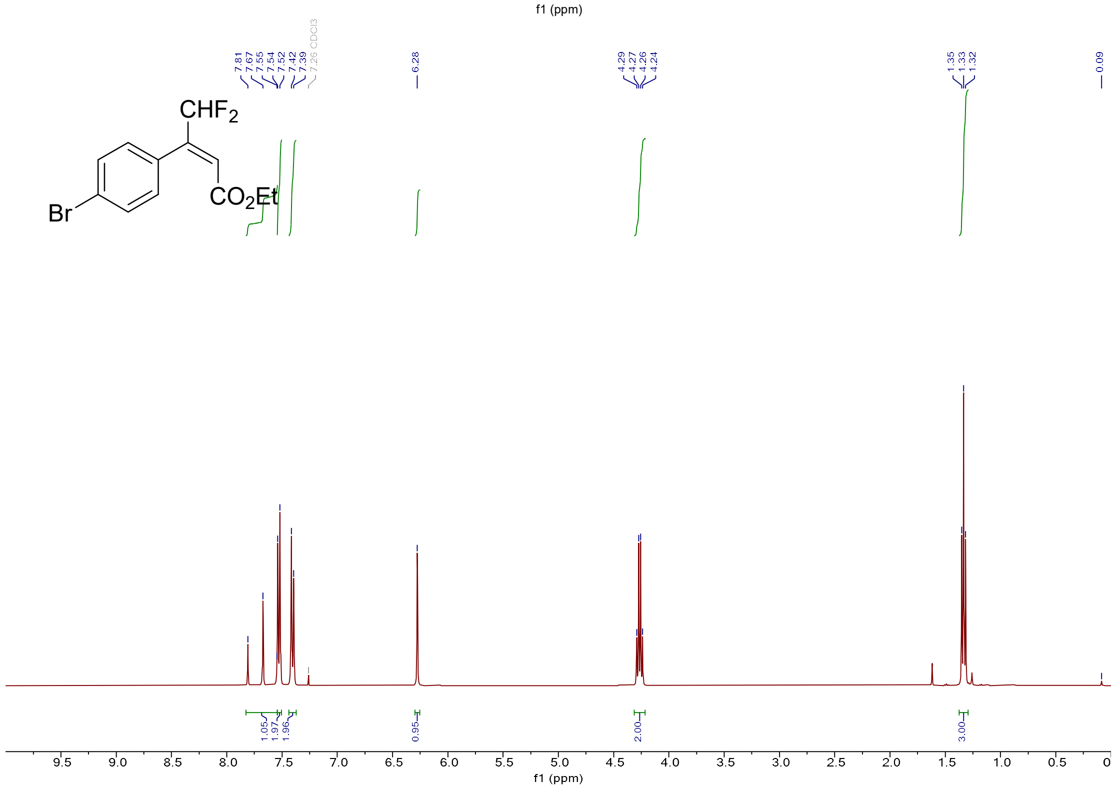

CHF2
Br
CO2Et
7.81
7.67
7.55
7.54
7.52
7.42
7.39
7.26 CDCl3
6.28
4.29
4.27
4.26
4.24
1.35
1.33
1.32
0.09
1.05
1.97
1.96
0.95
2.00
3.00
f1 (ppm)

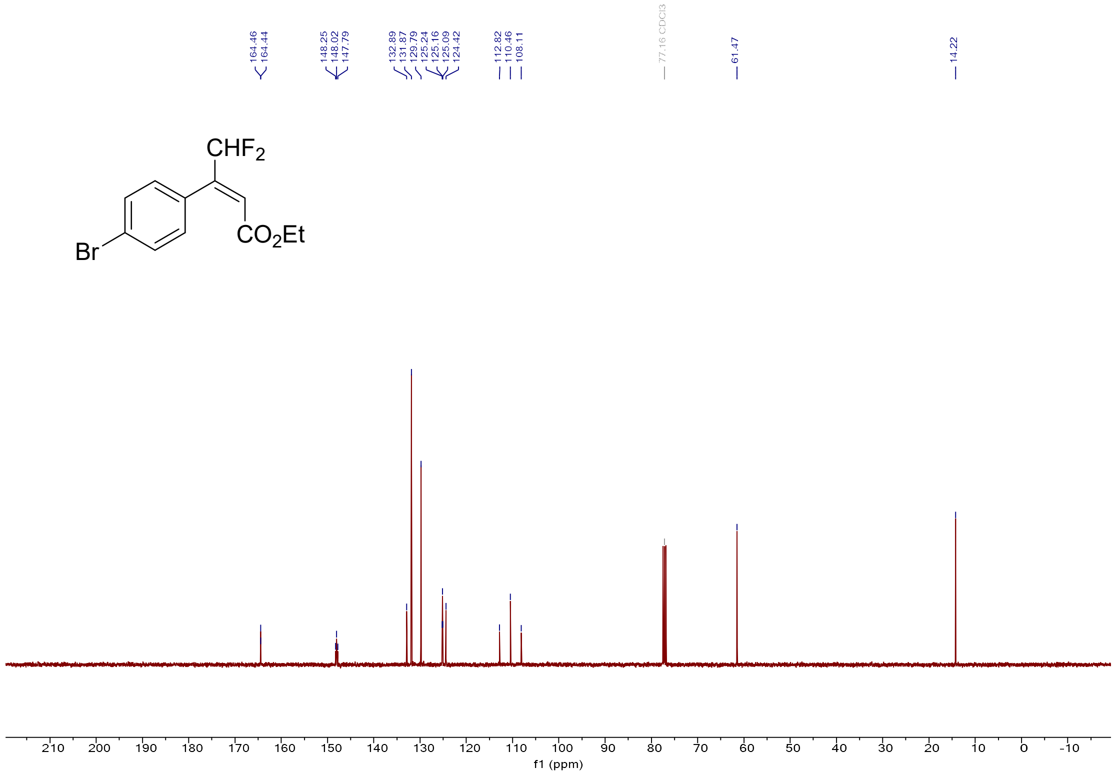
164.46
164.44
148.25
148.02
147.79
132.89
131.87
129.79
125.24
125.16
125.09
124.42
112.82
110.46
108.11
77.16 CDCl3
61.47
14.22
CHF2
CO2Et
Br
f1 (ppm)

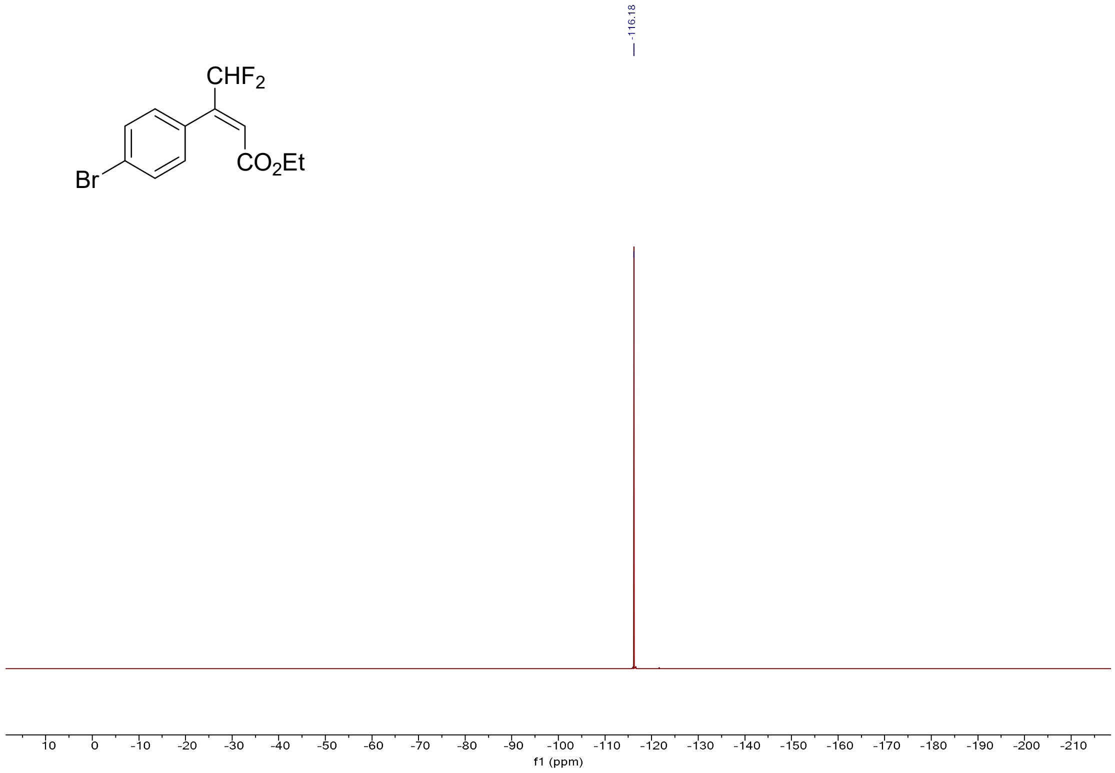
-116.18
CHF2
CO2Et
Br
f1 (ppm)

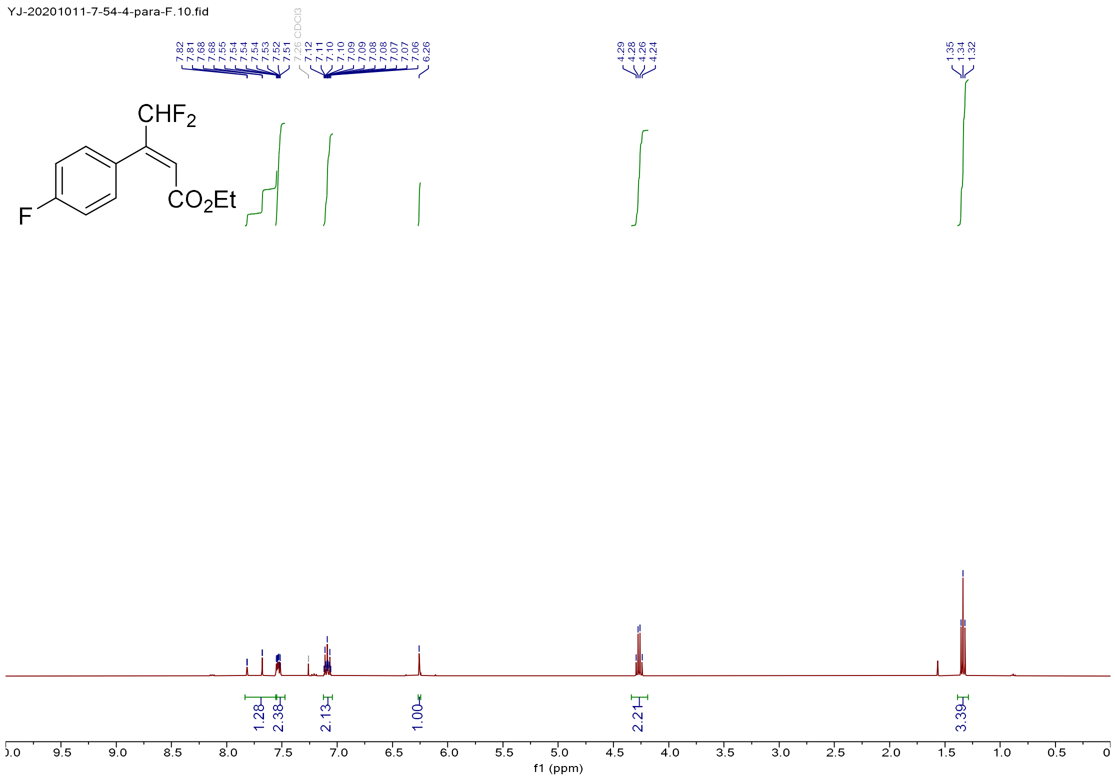
YJ-20201011-7-54-4-para-F.10.fid
7.82 7.81 7.68 7.68 7.55 7.54 7.54 7.53 7.52 7.51 7.26 CDCl3 7.12 7.11 7.10 7.10 7.09 7.09 7.08 7.08 7.07 7.07 7.06 6.26
4.29 4.28 4.26 4.24
1.35 1.34 1.32
CHF2
CO2Et
F
1.28 2.38 2.13 1.00 2.21 3.39
f1 (ppm)

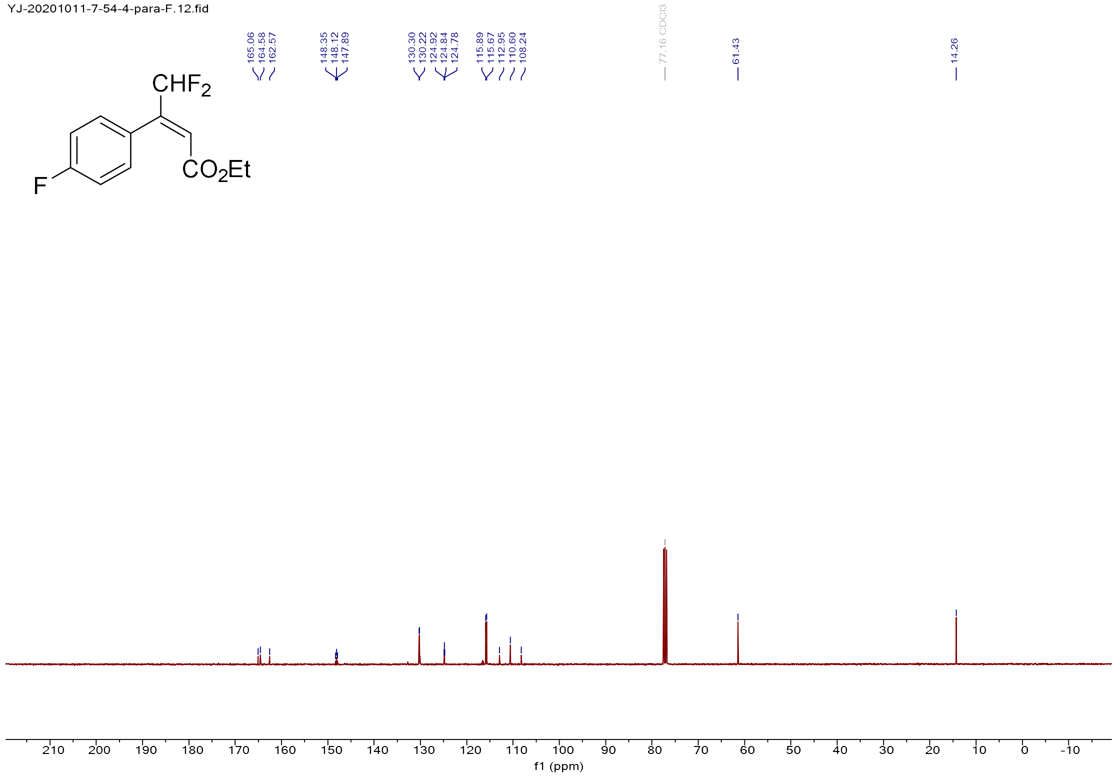
YJ-20201011-7-54-4-para-F.12.fid
165.06 164.58 162.57
148.35 148.12 147.89
130.30 130.22 124.92 124.84 124.78
115.89 115.67 112.95 110.60 108.24
77.16 CDCl3
61.43
14.26
CHF2
CO2Et
F
f1 (ppm)

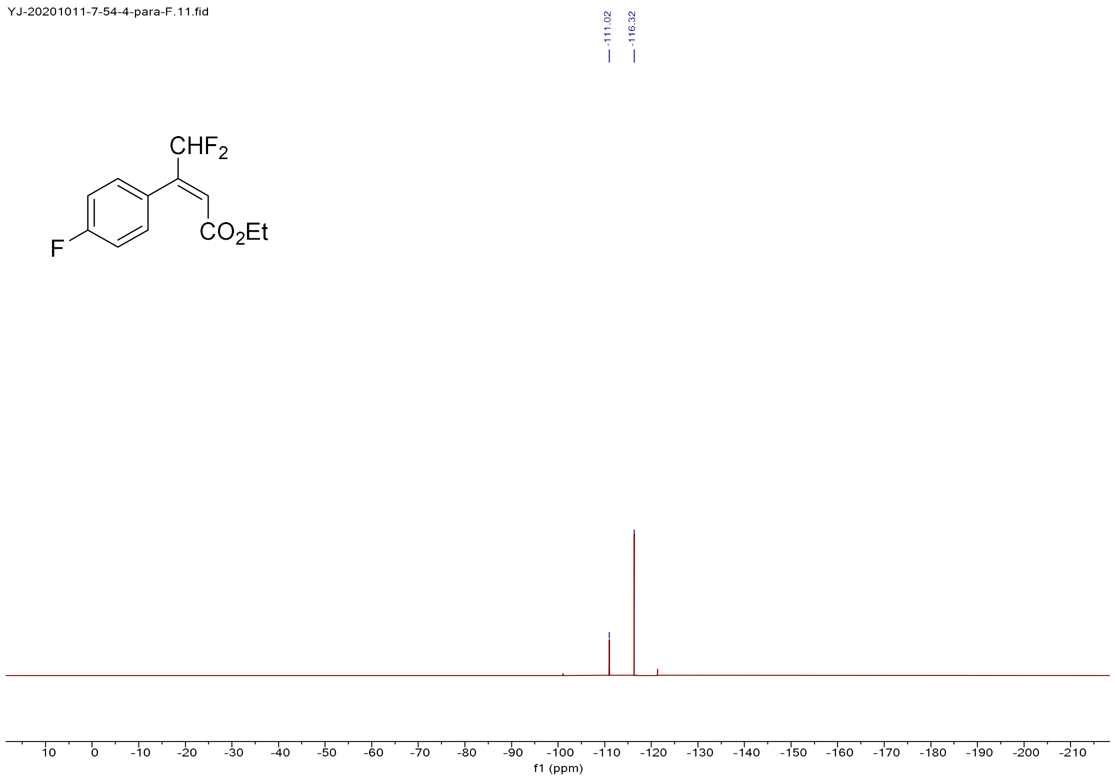
YJ-20201011-7-54-4-para-F.11.fid
-111.02
-116.32
CHF2
F
CO2Et
f1 (ppm)

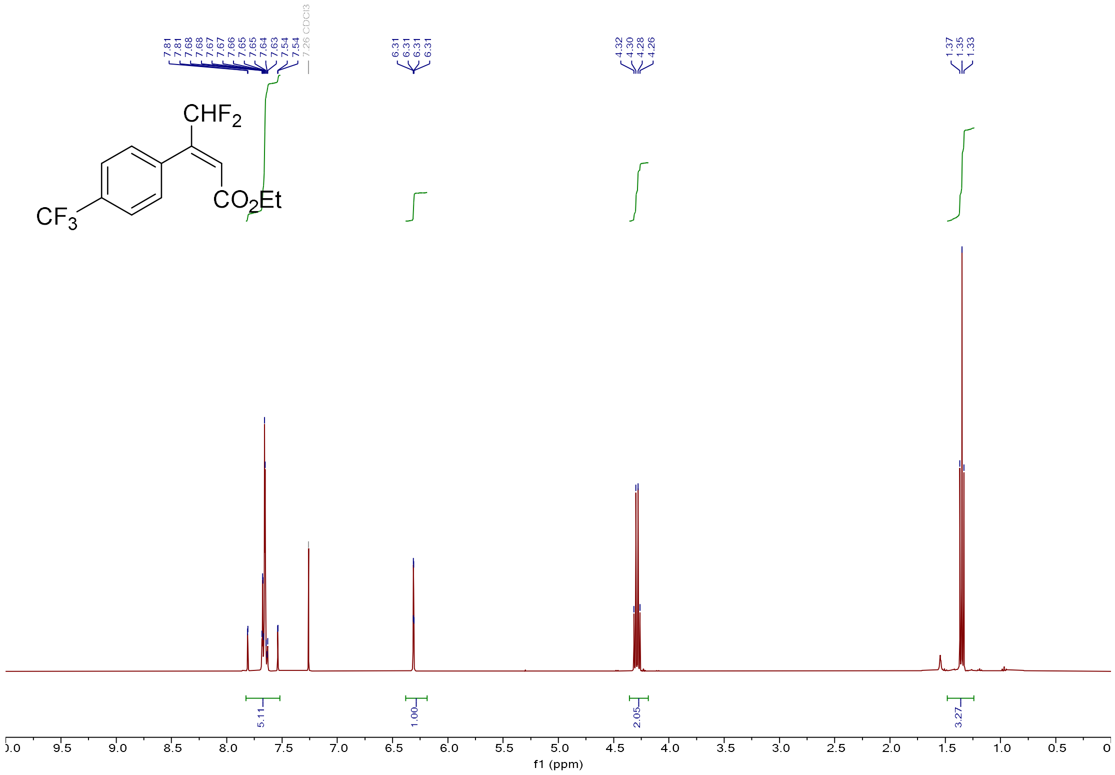
7.81
7.81
7.68
7.68
7.67
7.67
7.66
7.65
7.65
7.64
7.63
7.54
7.54
7.26 CDCl3
6.31
6.31
6.31
6.31
4.32
4.30
4.28
4.26
1.37
1.35
1.33
CHF2
CF3
CO2Et
5.11
1.00
2.05
3.27
f1 (ppm)

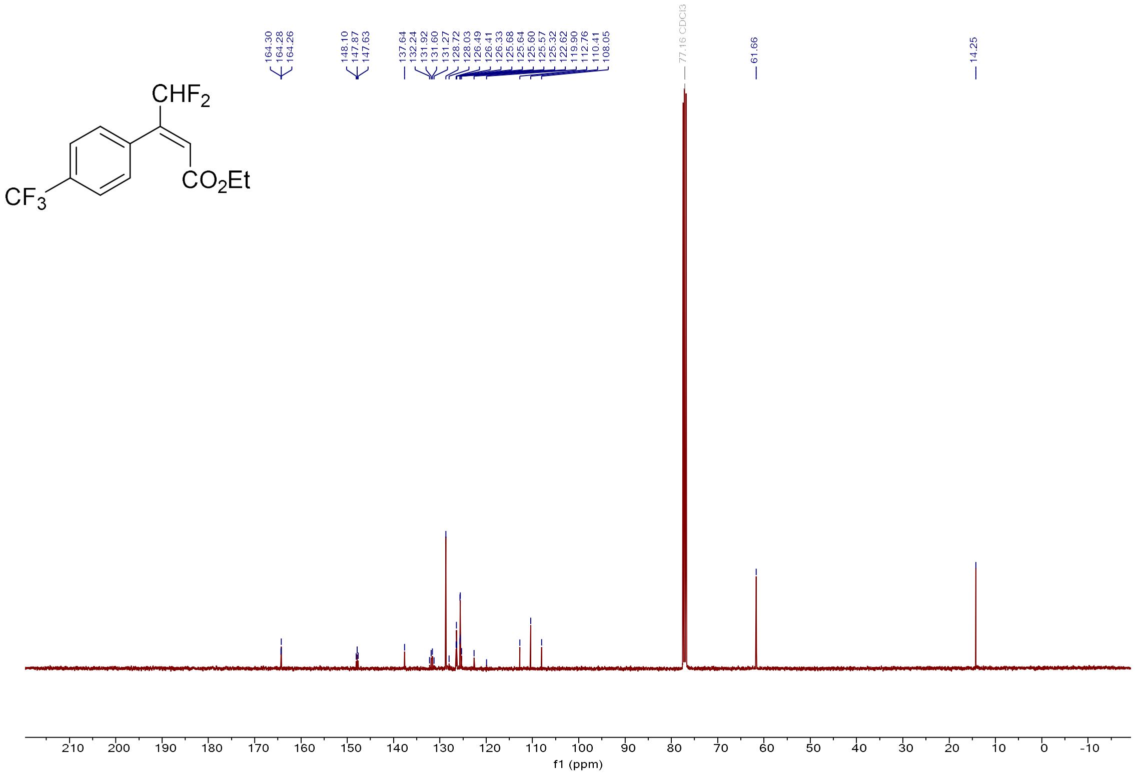
CHF2
CF3
CO2Et
164.30
164.28
164.26
148.10
147.87
147.63
137.64
132.24
131.92
131.60
131.27
128.72
128.03
126.49
126.41
126.33
125.68
125.64
125.60
125.57
125.32
122.62
119.90
112.76
110.41
108.05
77.16 CDCl3
61.66
14.25
f1 (ppm)

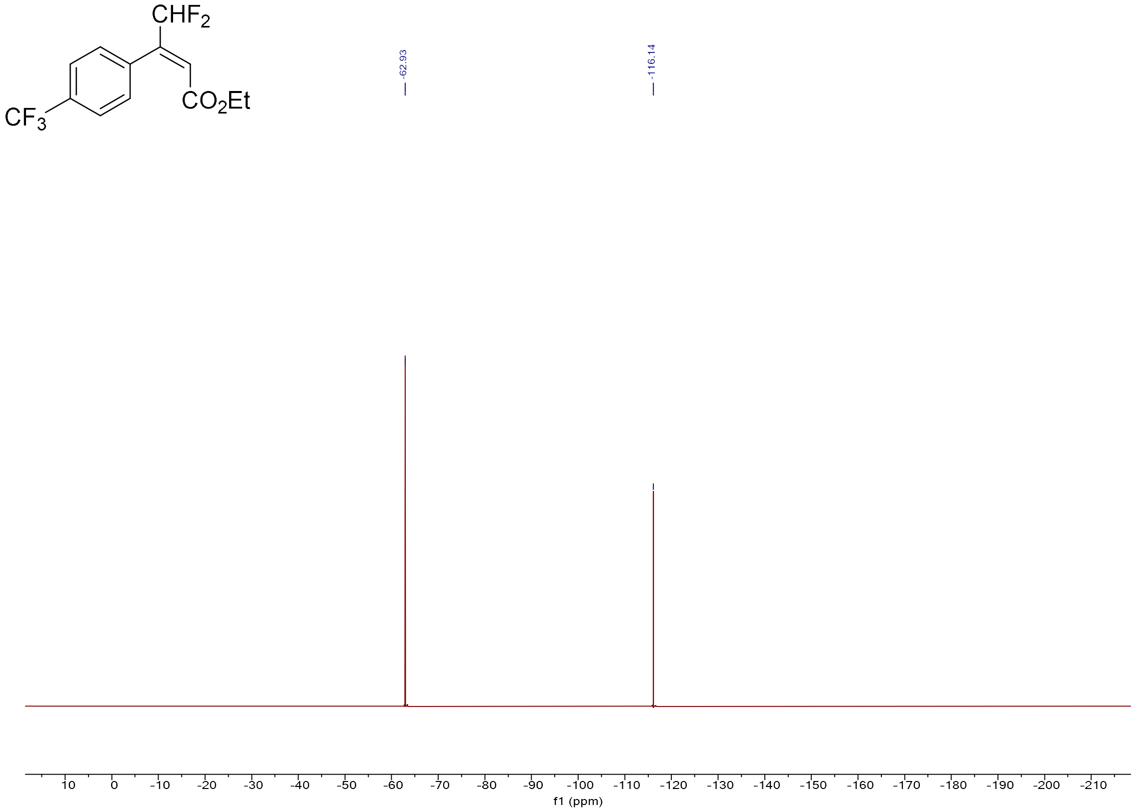
CHF2
CF3
CO2Et
-62.93
-116.14
f1 (ppm)

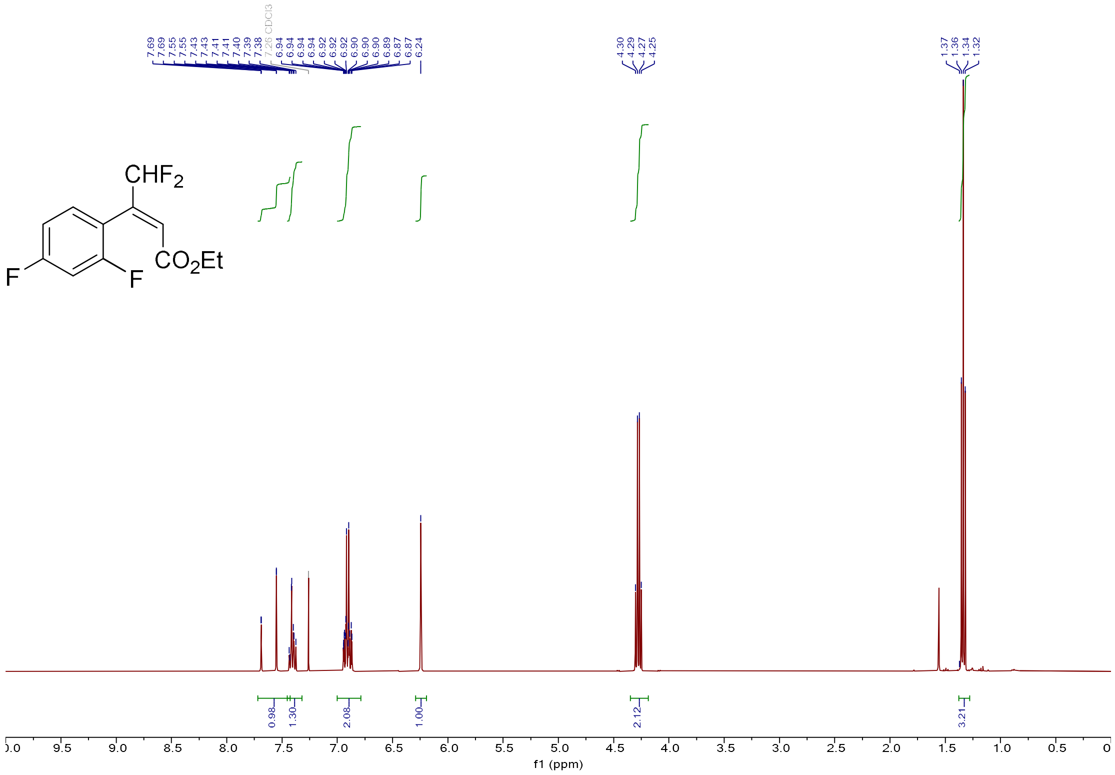

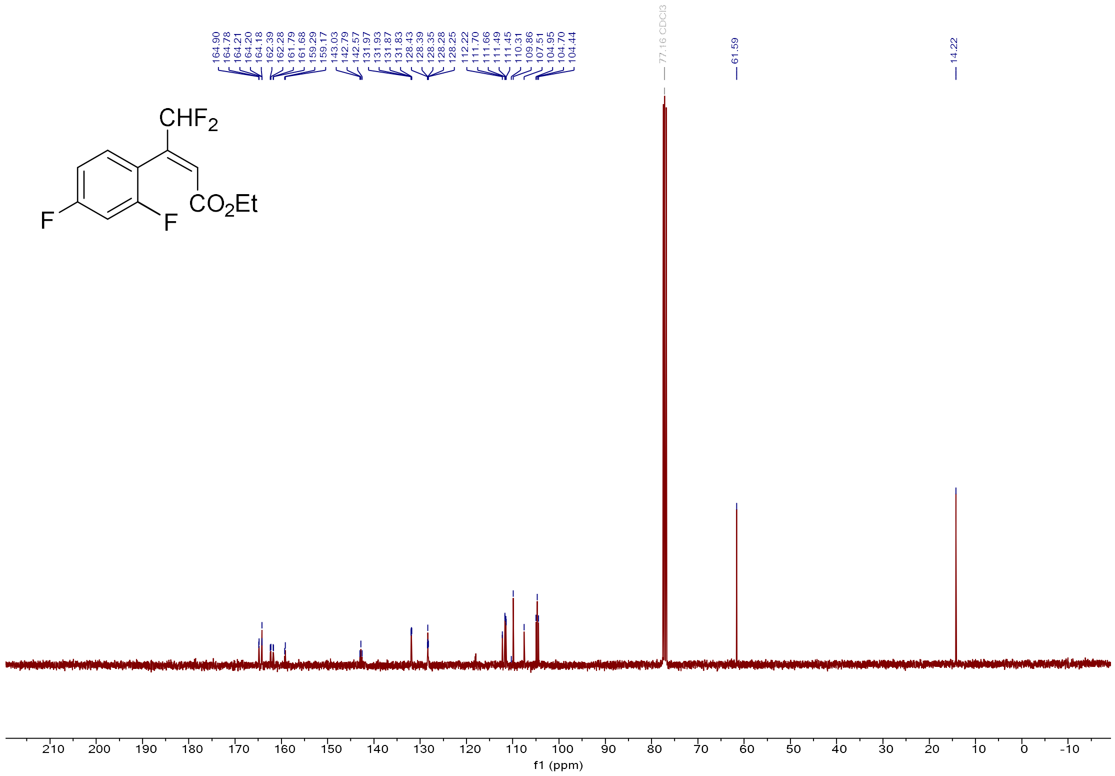

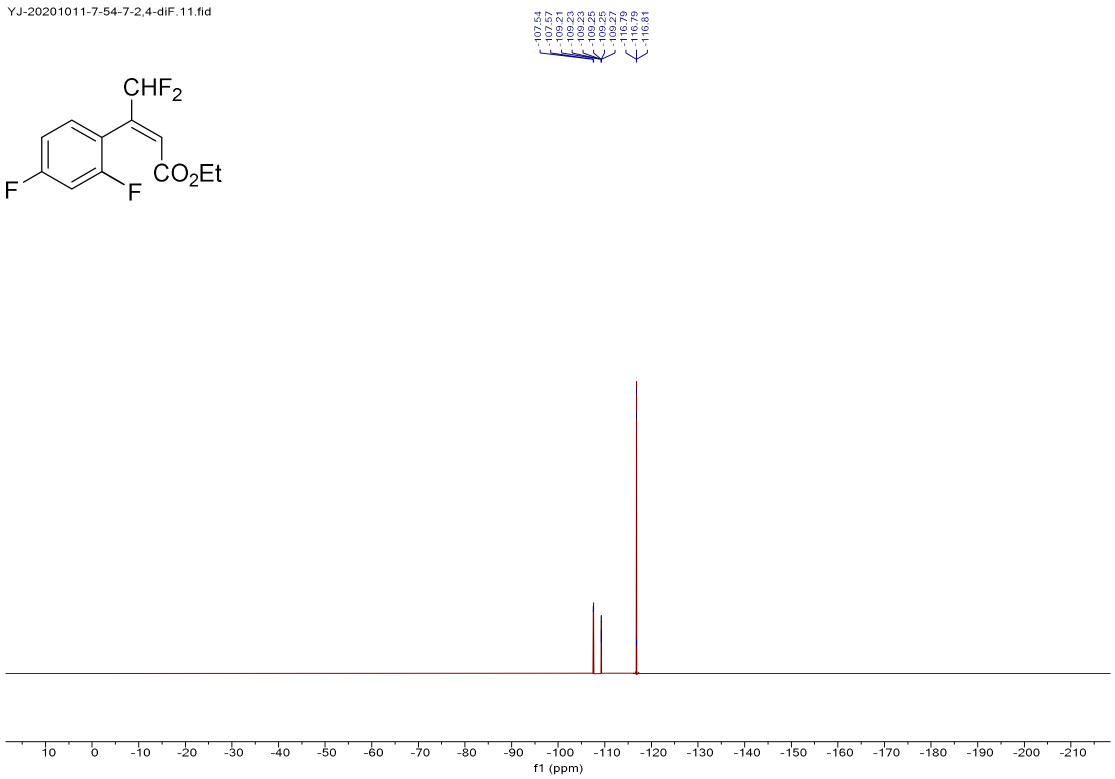
YJ-20201011-7-54-7-2,4-diF.11.fid
CHF2
CO2Et
F
F
-107.54
-107.57
-109.21
-109.23
-109.23
-109.25
-109.25
-109.27
-116.79
-116.79
-116.81
10 0 -10 -20 -30 -40 -50 -60 -70 -80 -90 -100 -110 -120 -130 -140 -150 -160 -170 -180 -190 -200 -210
f1 (ppm)

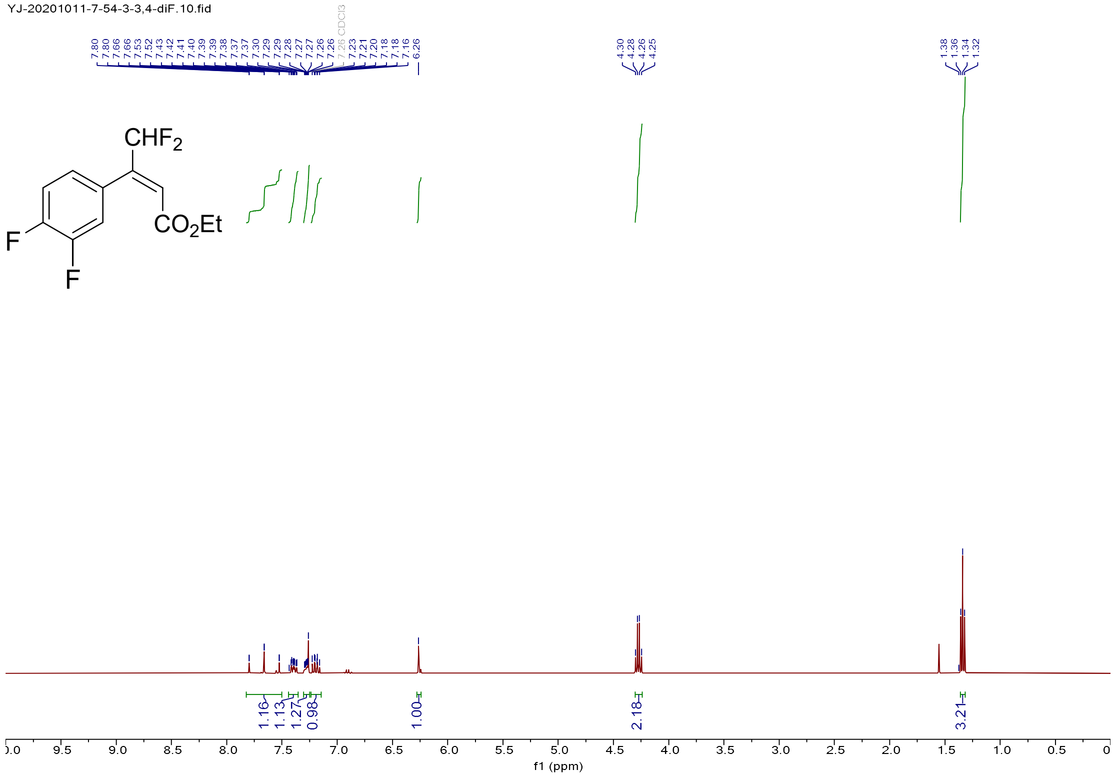
YJ-20201011-7-54-3-3,4-diF.10.fid
CHF2
CO2Et
F
F
7.80
7.80
7.66
7.53
7.52
7.43
7.42
7.41
7.40
7.39
7.39
7.38
7.37
7.30
7.29
7.29
7.28
7.27
7.27
7.26
7.26 CDCl3
7.23
7.21
7.20
7.18
7.16
6.26
4.30
4.28
4.26
4.25
1.38
1.36
1.34
1.32
1.16
1.13
1.27
0.98
1.00
2.18
3.21
9.5 9.0 8.5 8.0 7.5 7.0 6.5 6.0 5.5 5.0 4.5 4.0 3.5 3.0 2.5 2.0 1.5 1.0 0.5 0
f1 (ppm)

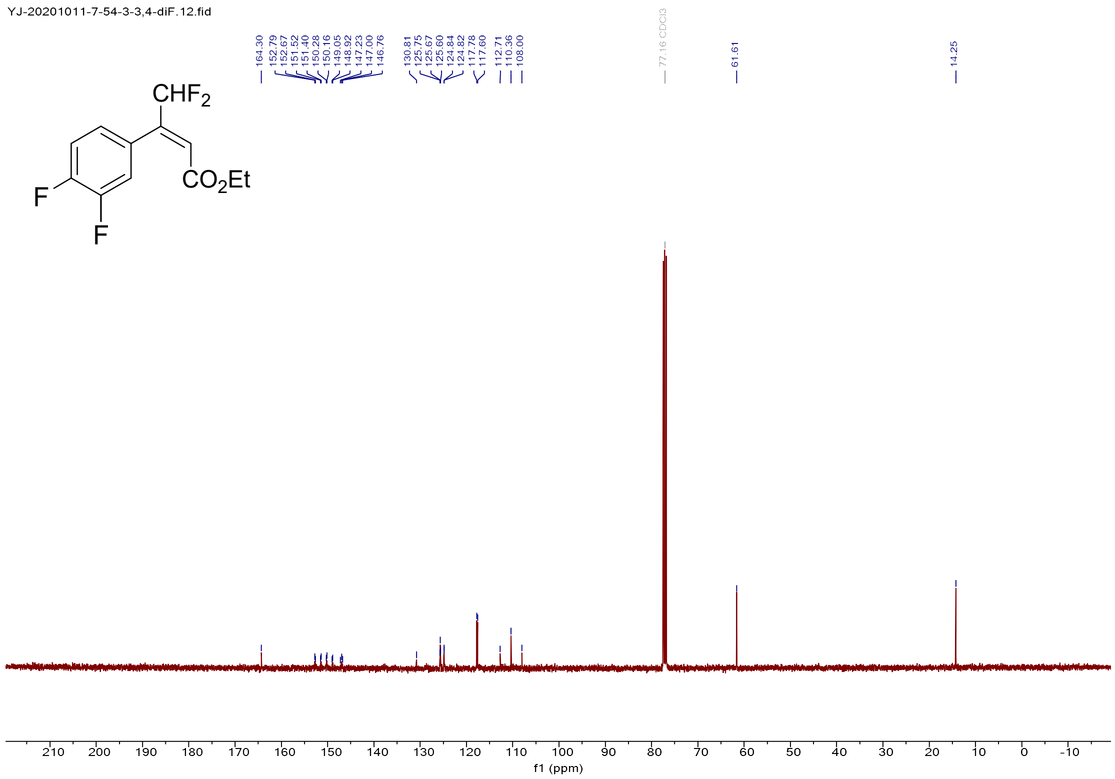
YJ-20201011-7-54-3-3,4-diF.12.fid
164.30
152.79
152.67
151.52
151.40
150.28
150.16
149.05
148.92
147.23
147.00
146.76
130.81
125.75
125.67
125.60
124.84
124.82
117.78
117.60
112.71
110.36
108.00
77.16 CDCl3
61.61
14.25
CHF2
CO2Et
F
F
f1 (ppm)

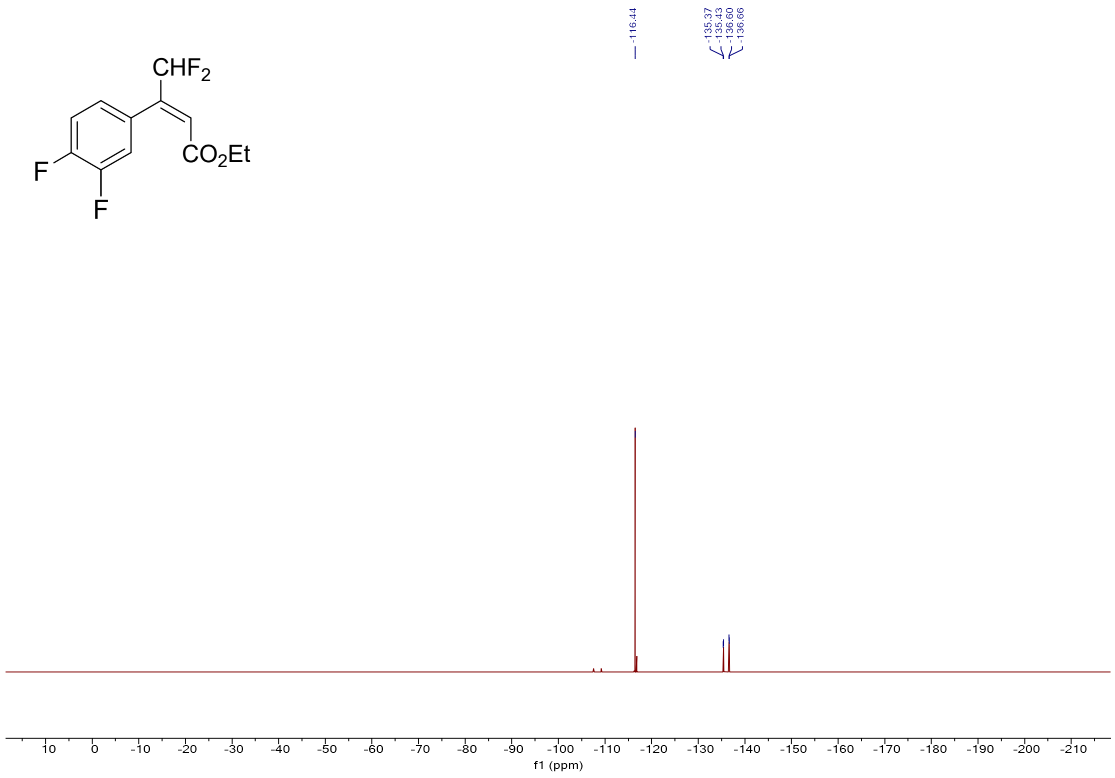
-116.44
-135.37
-135.43
-136.60
-136.66
CHF2
CO2Et
F
F
f1 (ppm)

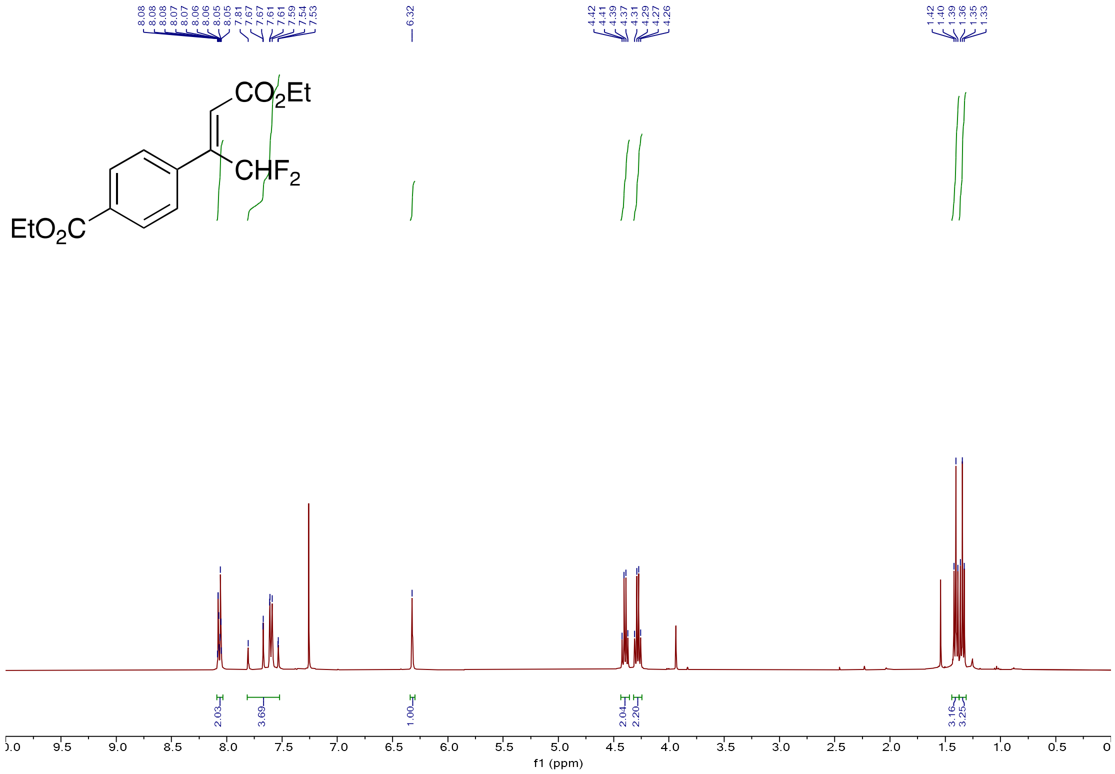
8.08
8.08
8.08
8.07
8.07
8.06
8.06
8.05
8.05
7.81
7.67
7.67
7.61
7.61
7.59
7.54
7.53
6.32
4.42
4.41
4.39
4.37
4.31
4.29
4.27
4.26
1.42
1.40
1.39
1.36
1.35
1.33
CO2Et
CHF2
EtO2C
2.03
3.69
1.00
2.04
2.20
3.16
3.25
f1 (ppm)

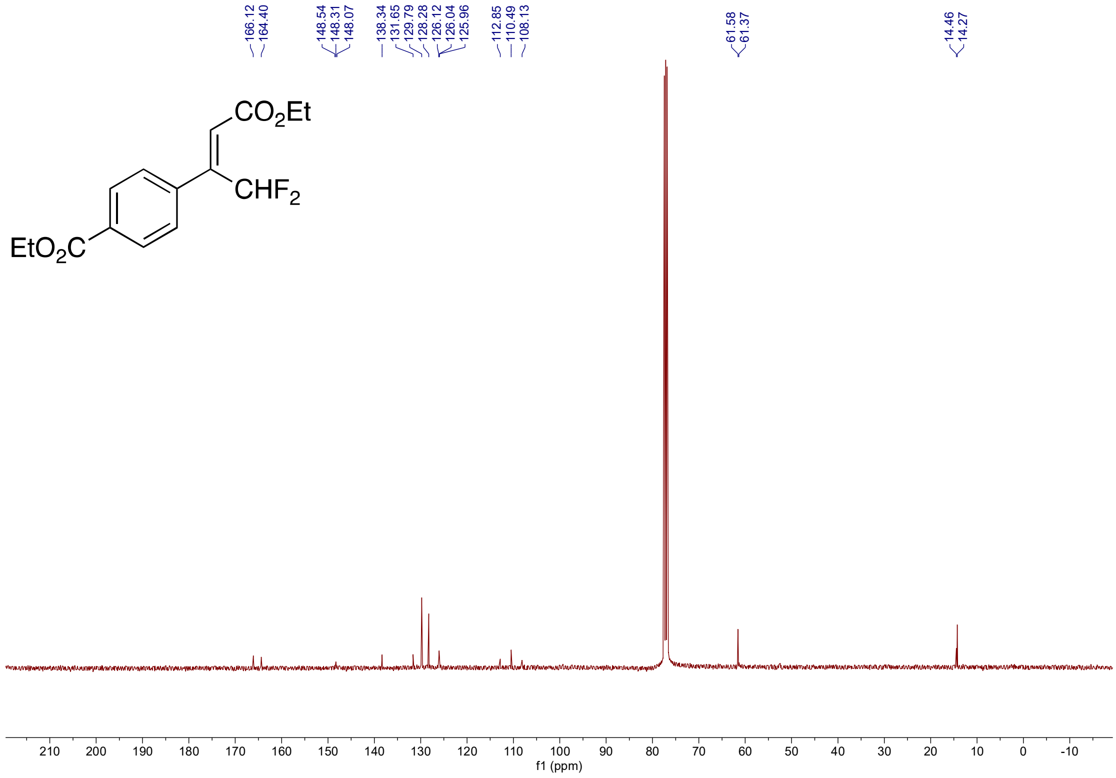
166.12
164.40
148.54
148.31
148.07
138.34
131.65
129.79
128.28
126.12
126.04
125.96
112.85
110.49
108.13
61.58
61.37
14.46
14.27
CO2Et
CHF2
EtO2C
f1 (ppm)

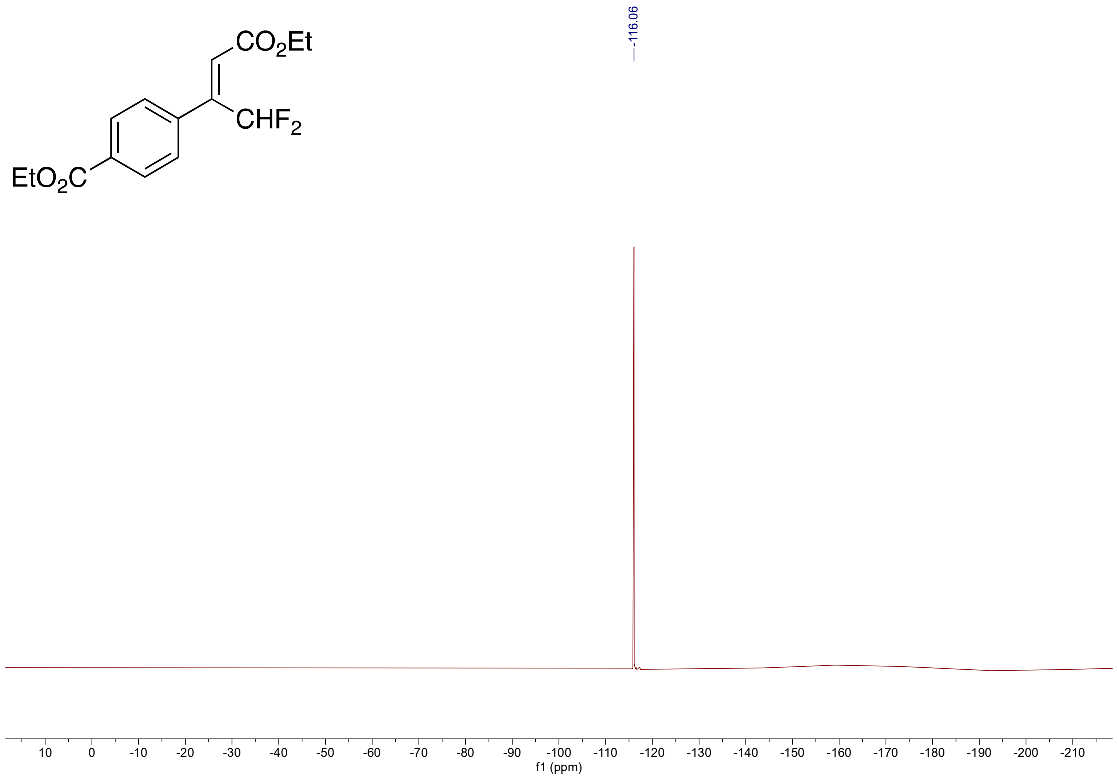
CO2Et
CHF2
EtO2C
-116.06
f1 (ppm)

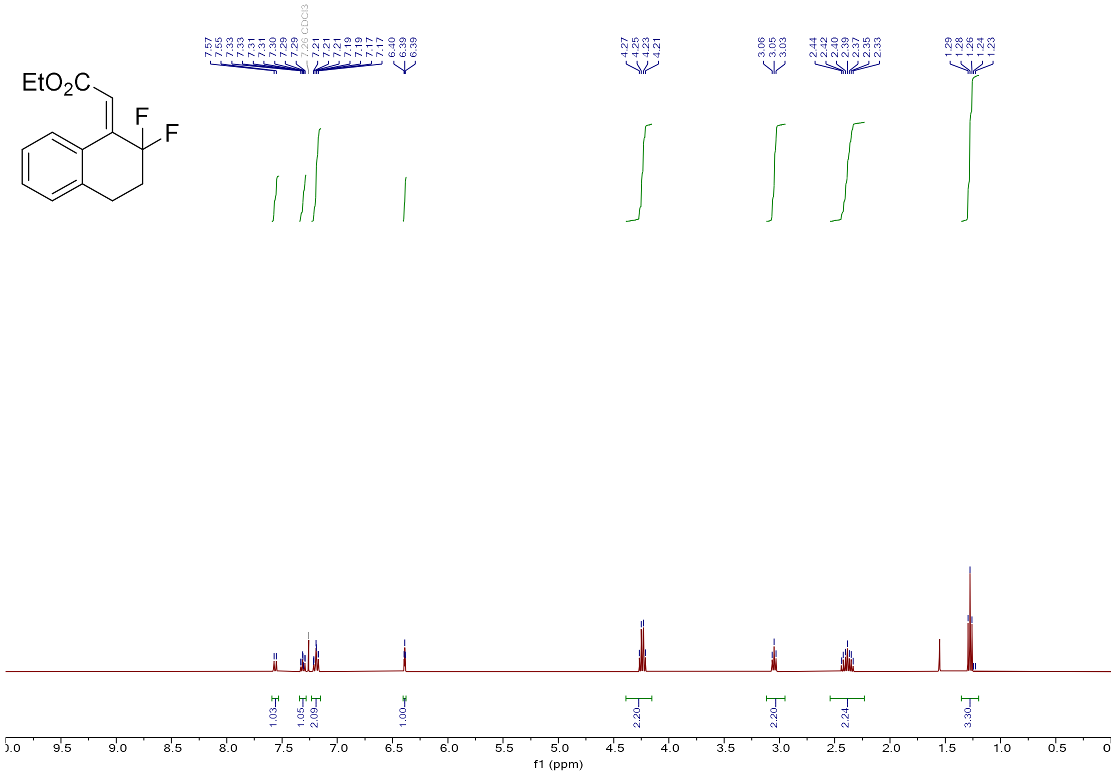
EtO2C
F
F
7.26 CDCl3
f1 (ppm)

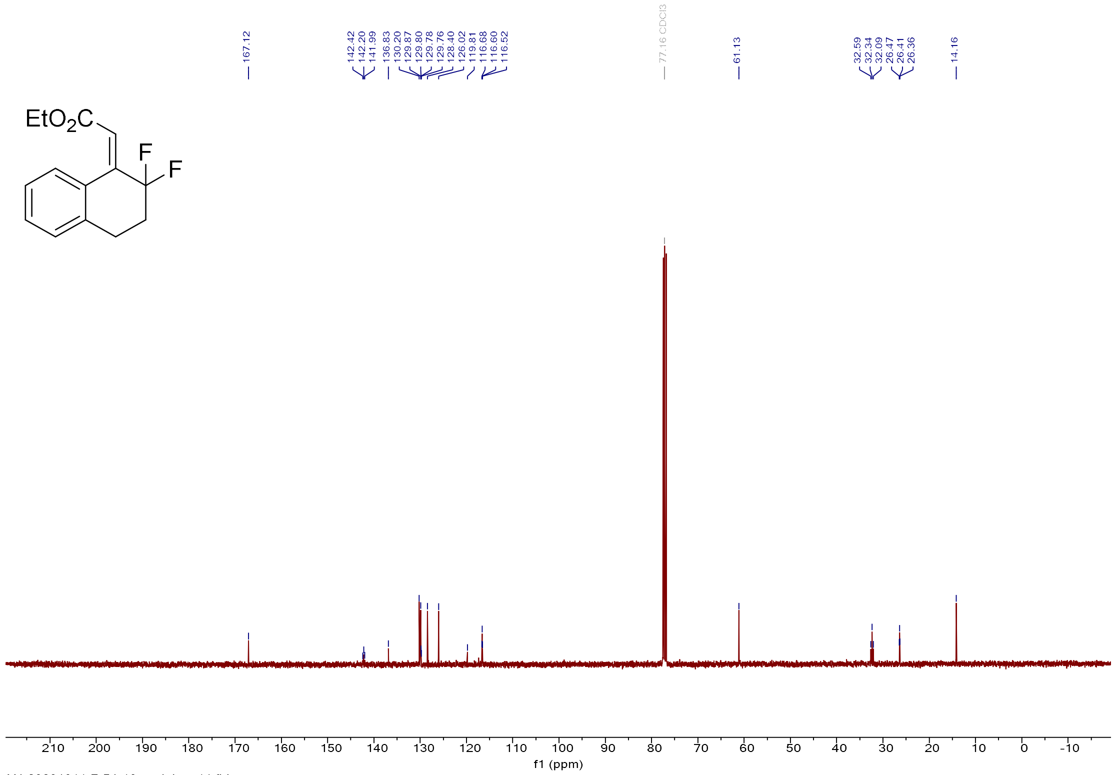

YJ-20201011-7-54-10-cyclohex.11.fid

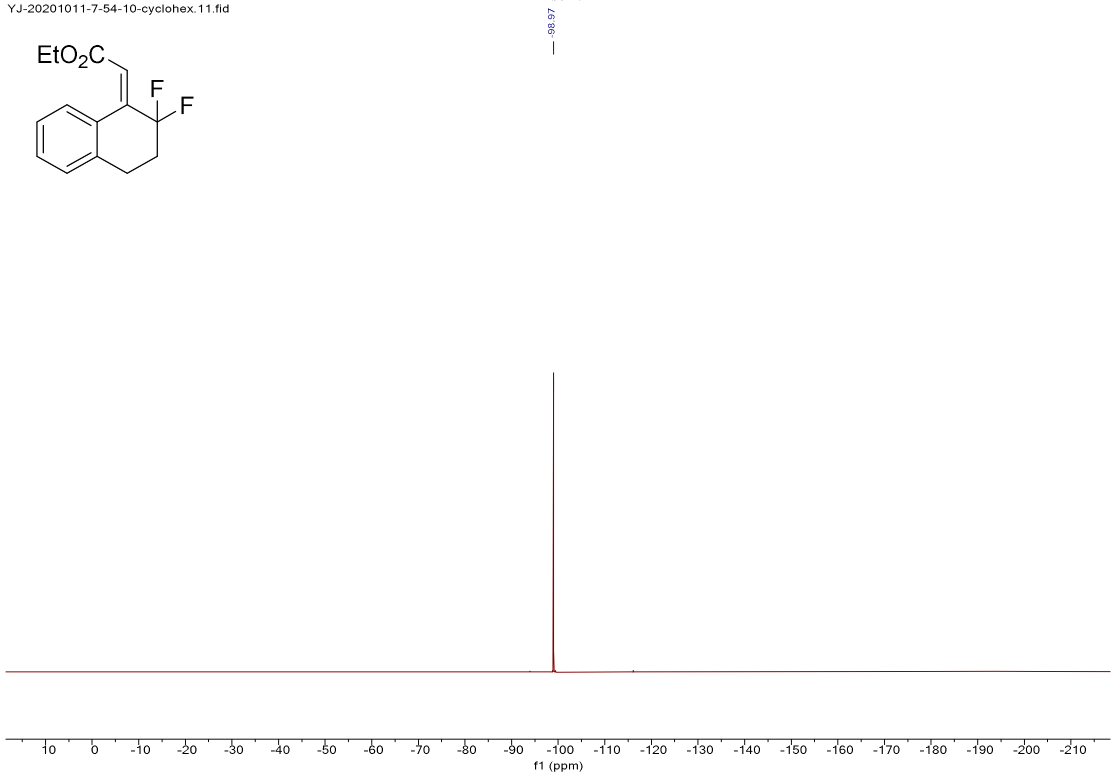

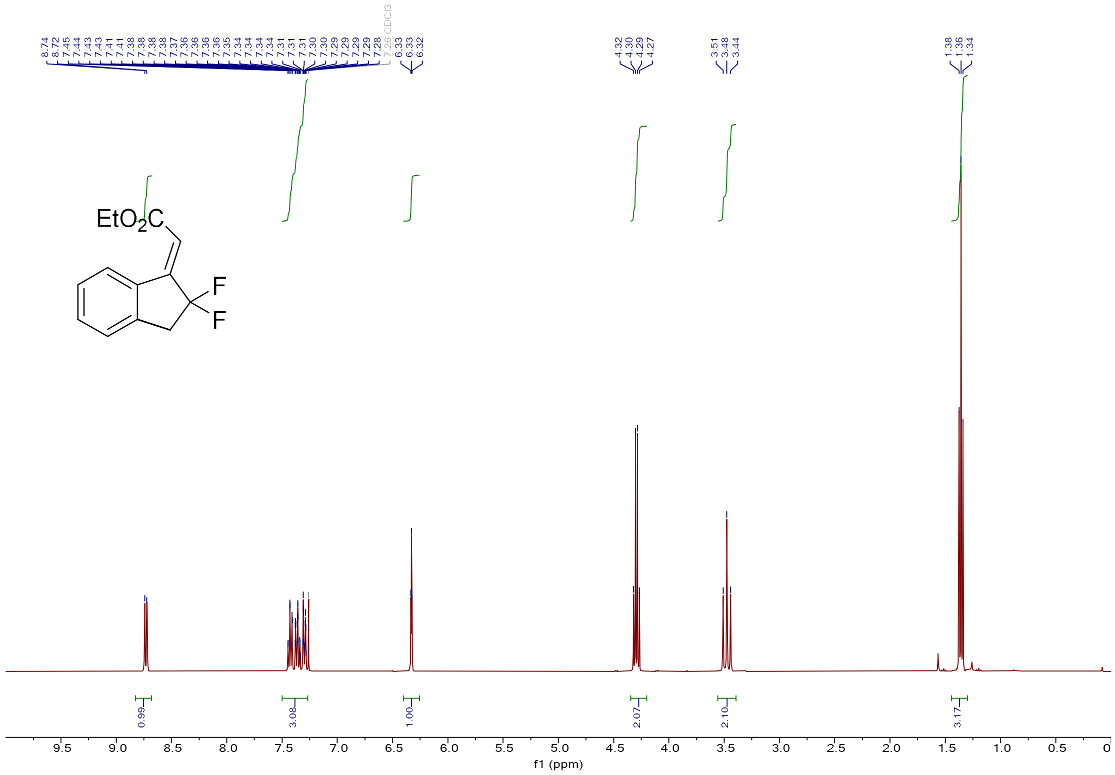

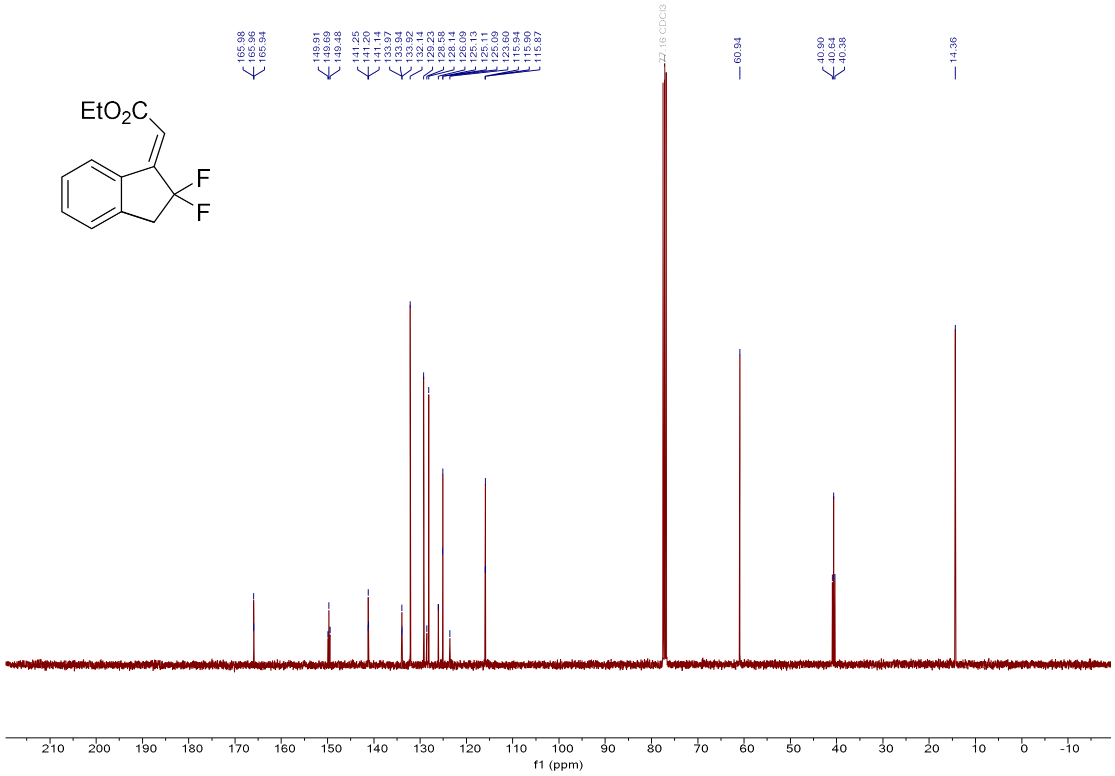

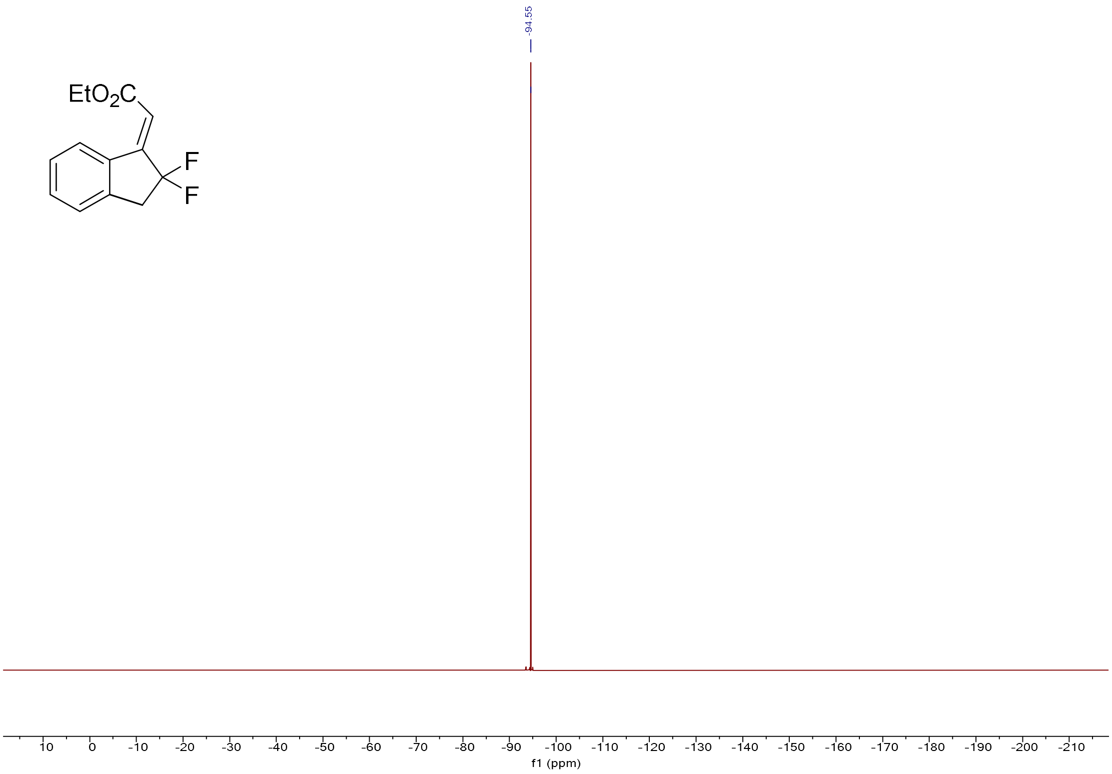

EtO2C
F
F
-94.55
10 0 -10 -20 -30 -40 -50 -60 -70 -80 -90 -100 -110 -120 -130 -140 -150 -160 -170 -180 -190 -200 -210
f1 (ppm)

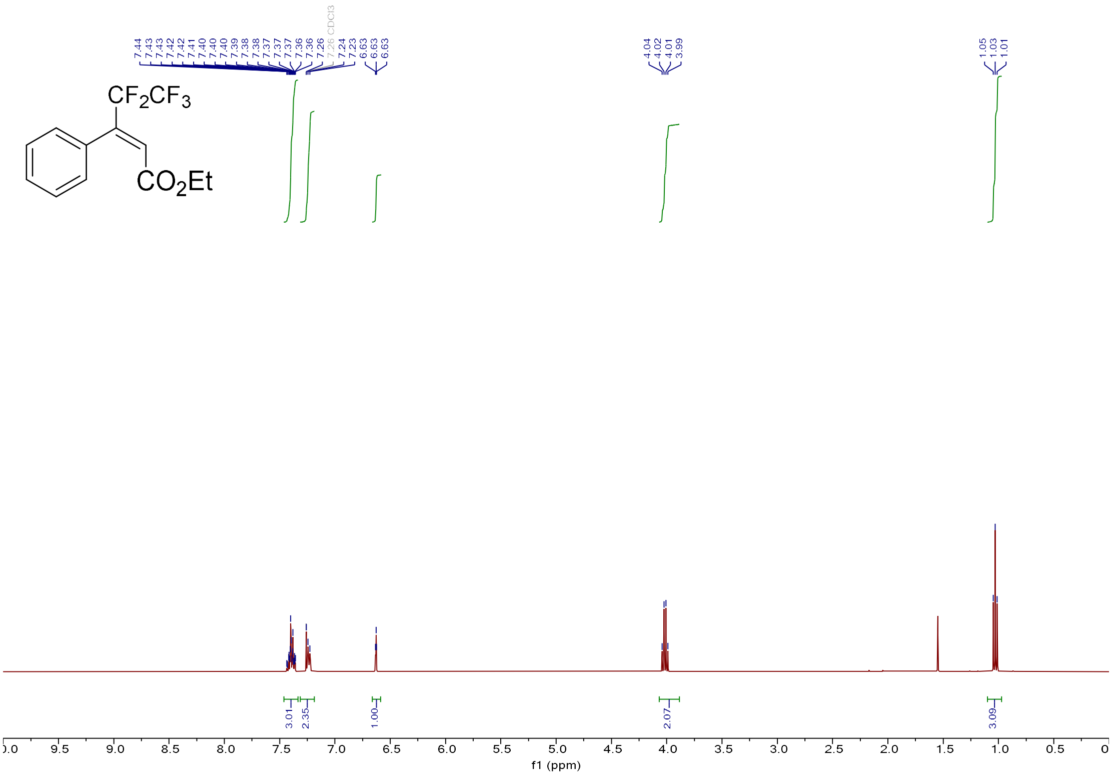

CF2CF3
CO2Et
7.44 7.43 7.43 7.42 7.42 7.41 7.40 7.40 7.39 7.38 7.38 7.37 7.37 7.36 7.36 7.26 7.26 CDCl3 7.24 7.23 6.63 6.63 6.63
4.04 4.02 4.01 3.99
1.05 1.03 1.01
3.01 2.35 1.00 2.07 3.09
9.5 9.0 8.5 8.0 7.5 7.0 6.5 6.0 5.5 5.0 4.5 4.0 3.5 3.0 2.5 2.0 1.5 1.0 0.5 0
f1 (ppm)

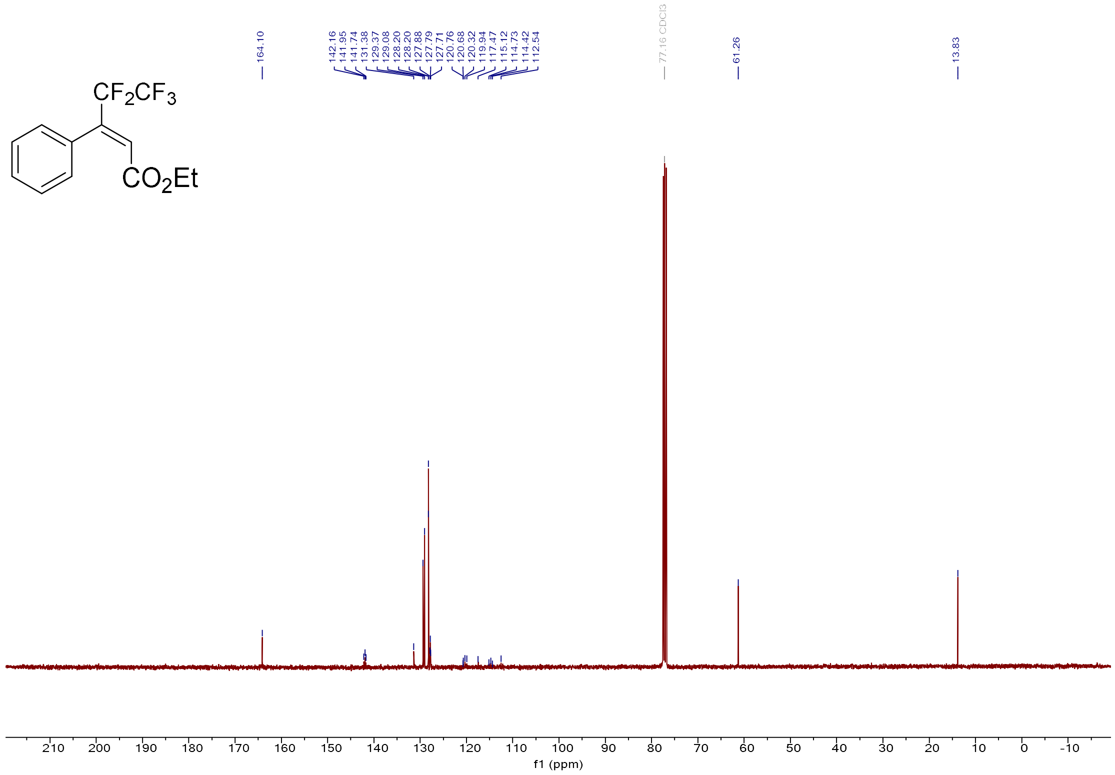
CF2CF3
CO2Et
164.10
142.16
141.95
141.74
131.38
129.37
129.08
128.20
128.20
127.88
127.79
127.71
120.76
120.68
120.32
119.94
117.47
115.12
114.73
114.42
112.54
77.16 CDCl3
61.26
13.83
f1 (ppm)

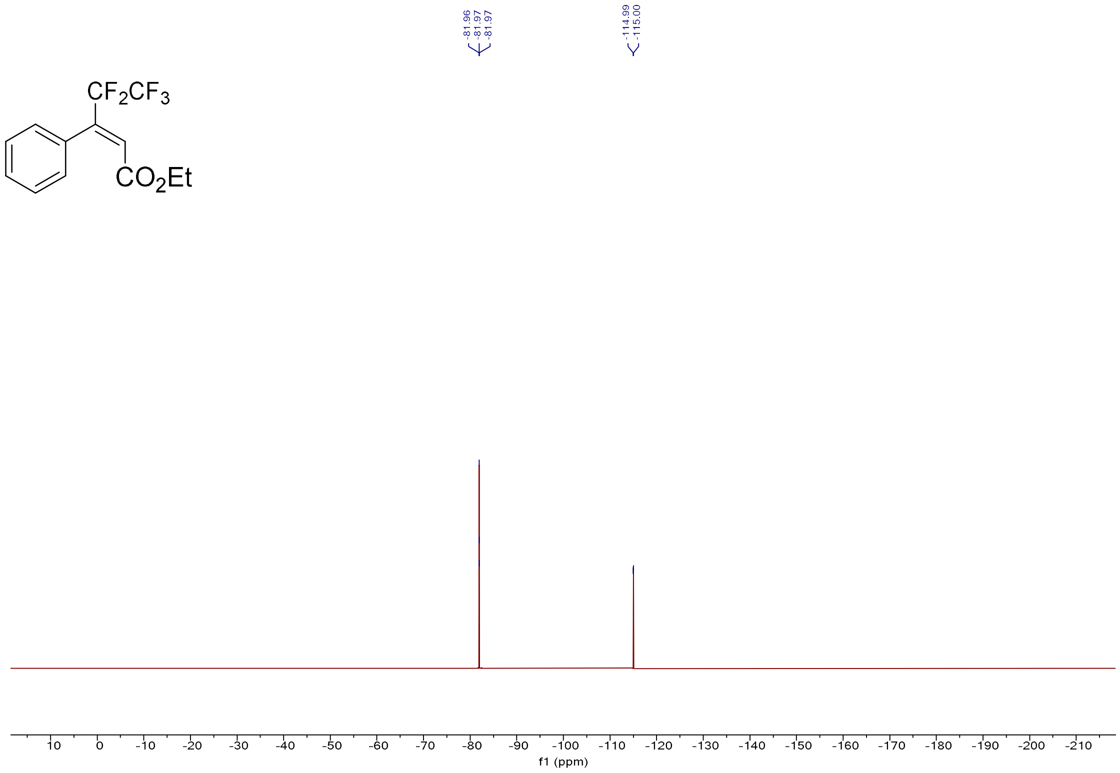
CF2CF3
CO2Et
-81.96
-81.97
-81.97
-114.99
-115.00
f1 (ppm)

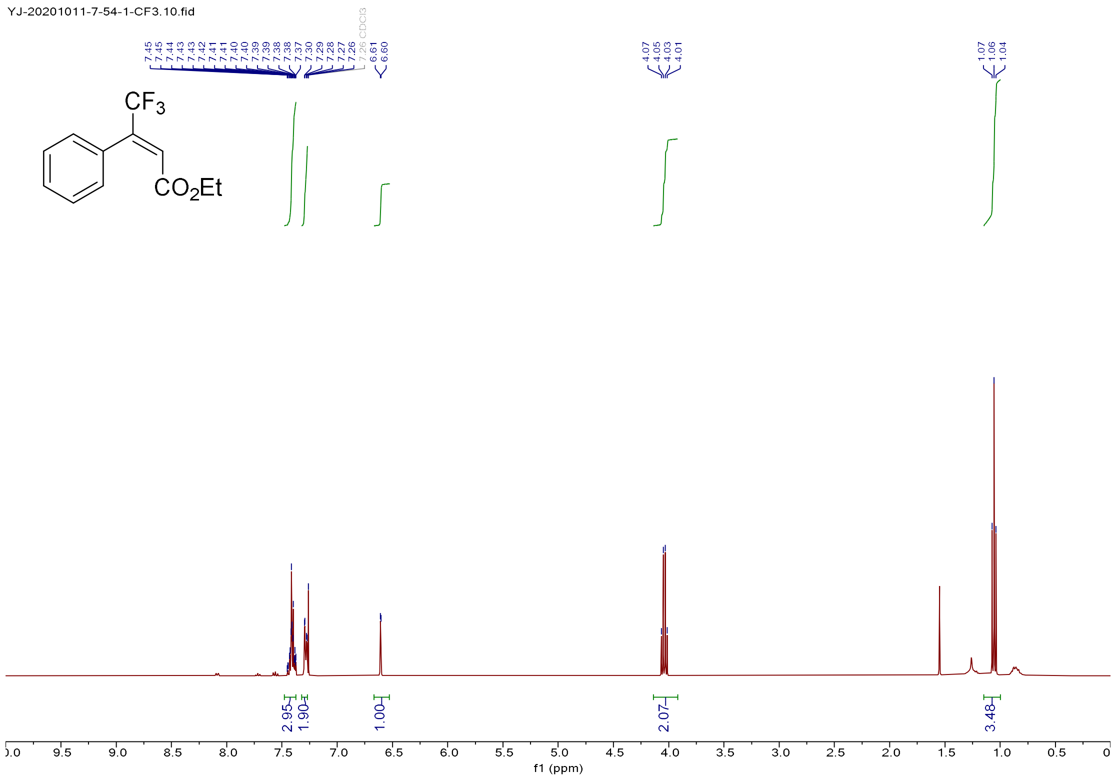
YJ-20201011-7-54-1-CF3.10.fid
7.45
7.45
7.44
7.43
7.43
7.42
7.41
7.41
7.40
7.40
7.39
7.39
7.38
7.38
7.37
7.30
7.29
7.28
7.27
7.26
7.26 CDCl3
6.61
6.60
4.07
4.05
4.03
4.01
1.07
1.06
1.04
CF3
CO2Et
2.95
1.90
1.00
2.07
3.48
f1 (ppm)

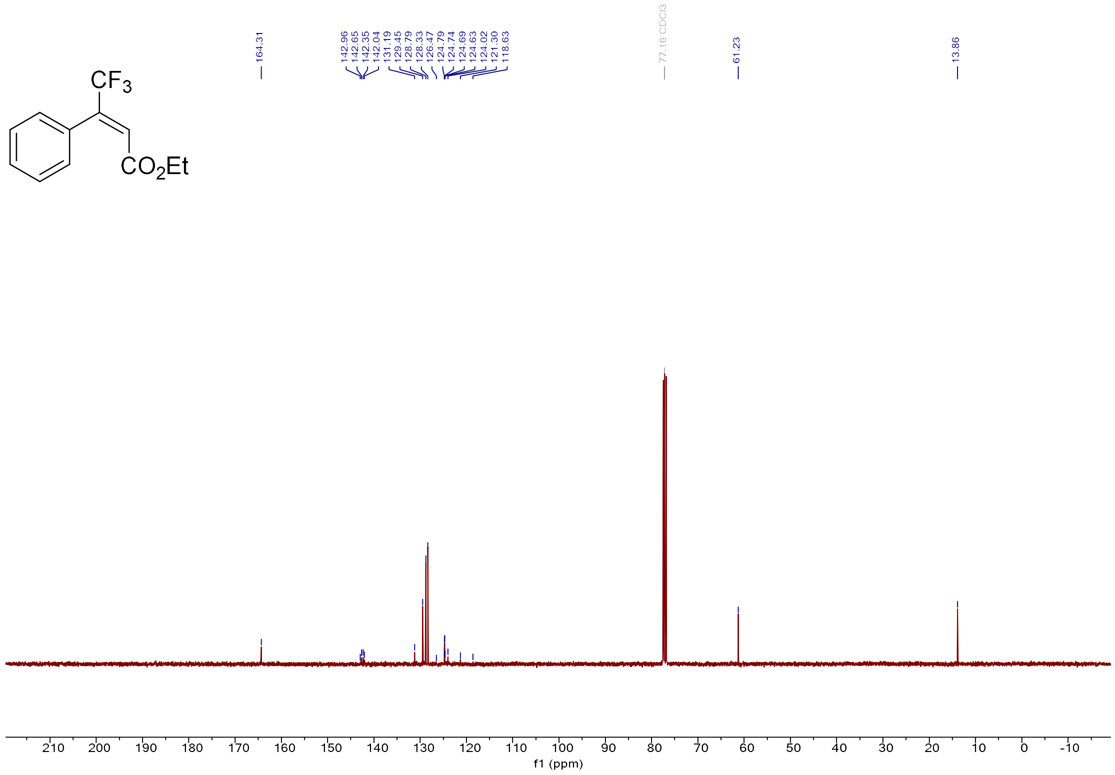
164.31
142.96
142.65
142.35
142.04
131.19
129.45
128.79
128.33
126.47
124.79
124.74
124.69
124.63
124.02
121.30
118.63
77.16 CDCl3
61.23
13.86
CF3
CO2Et
f1 (ppm)

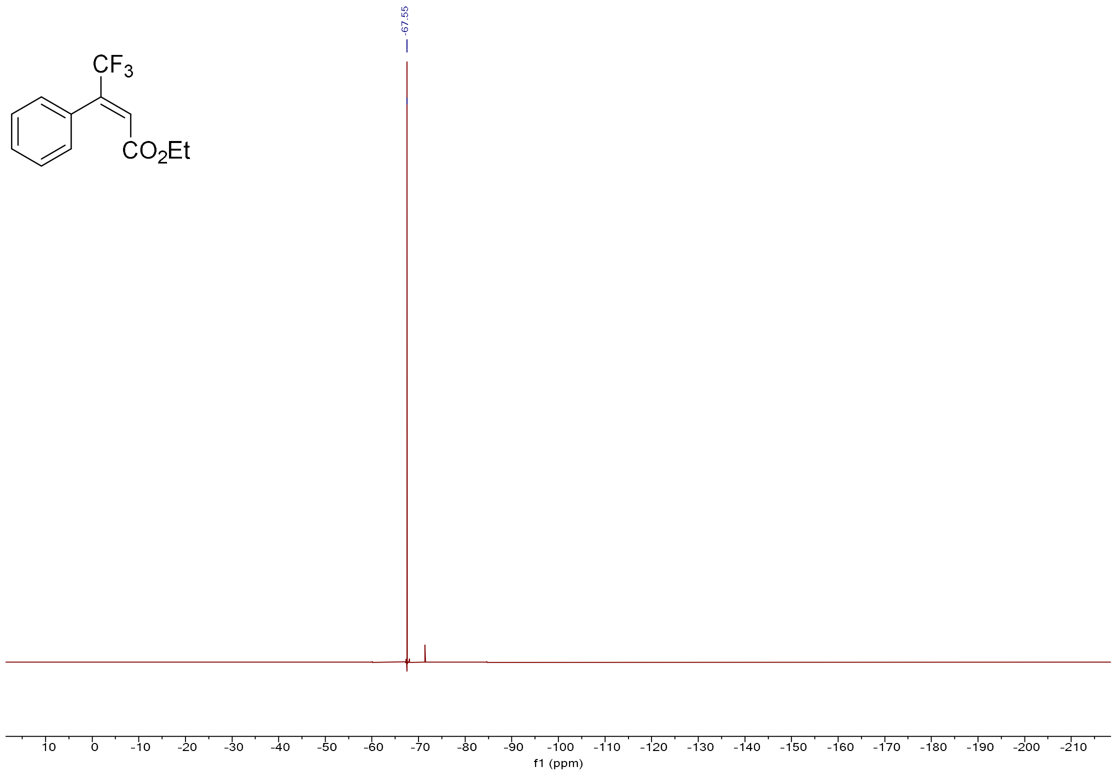
CF3
CO2Et
-67.55
f1 (ppm)

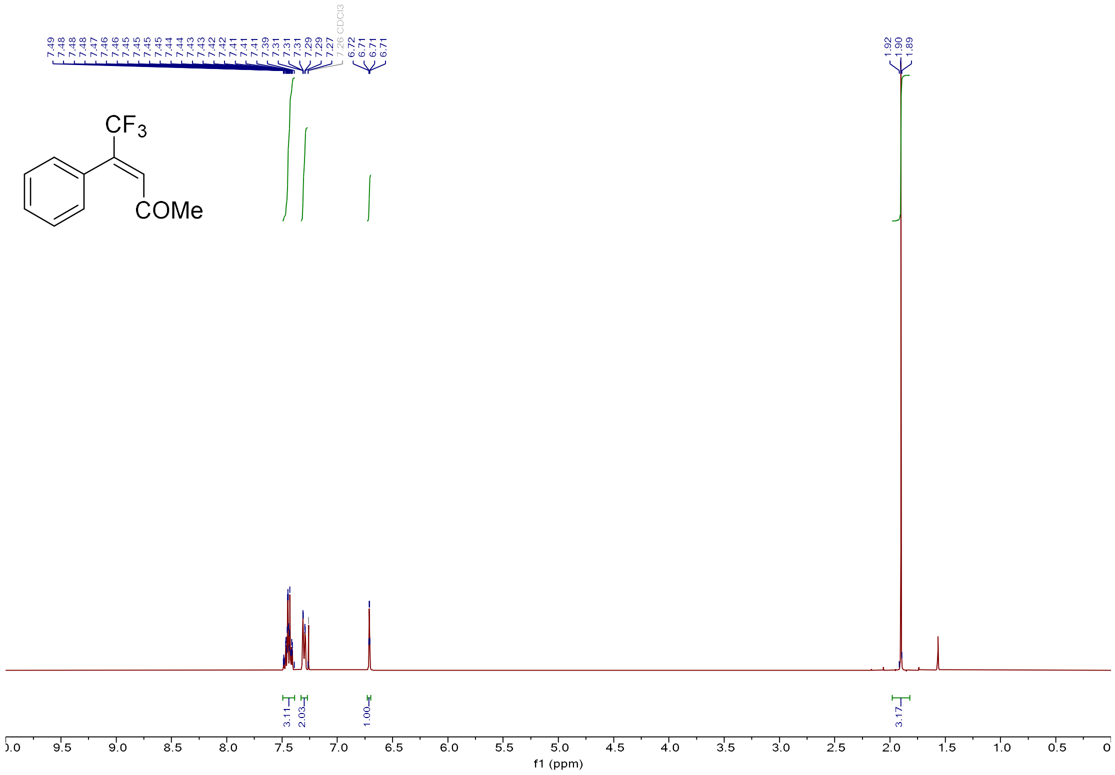
CF3
COMe
3.11
2.03
1.00
3.17
f1 (ppm)

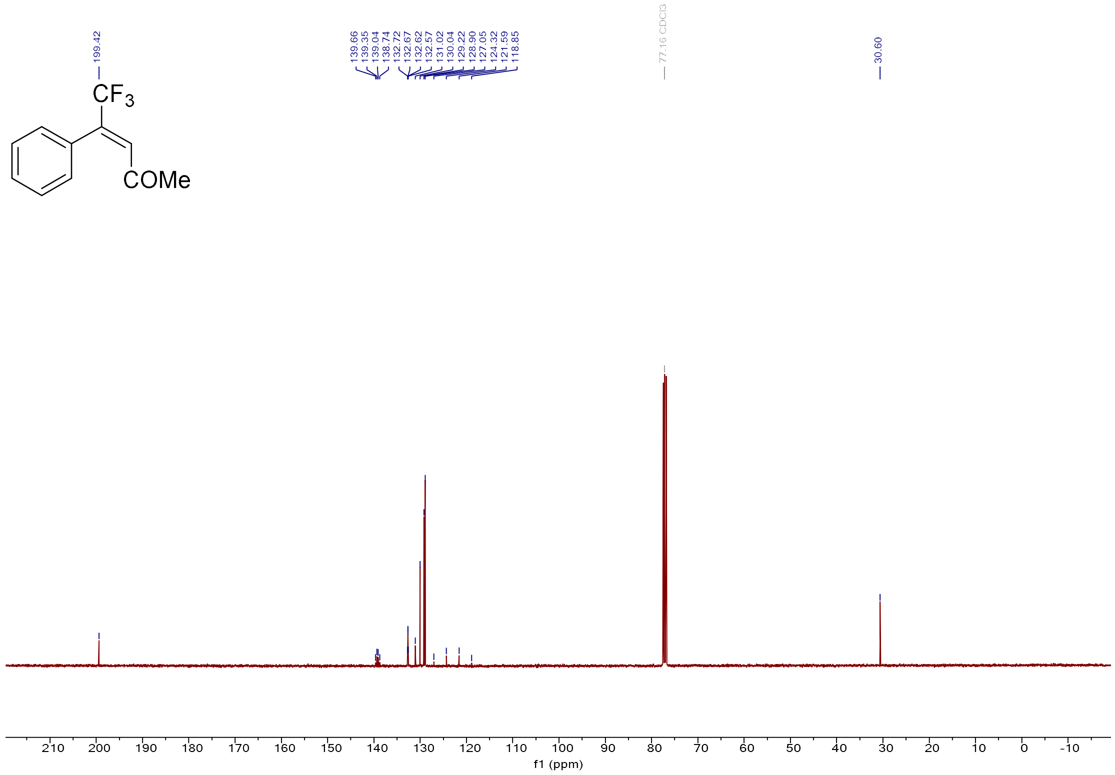

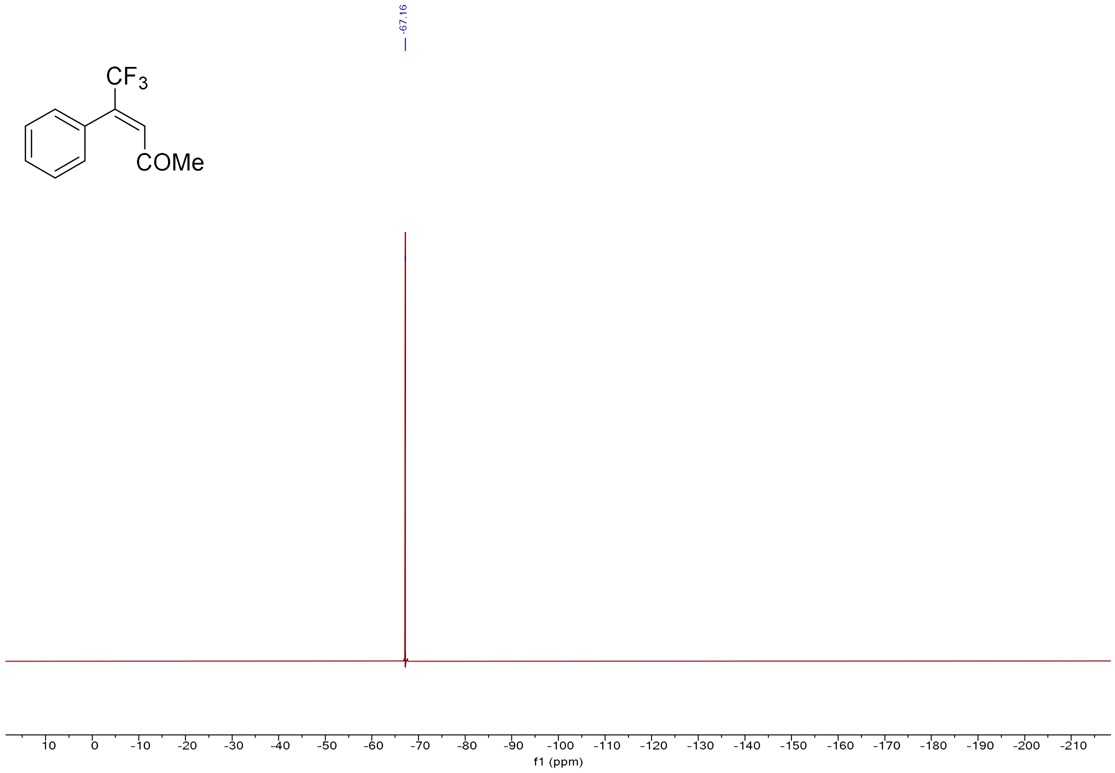

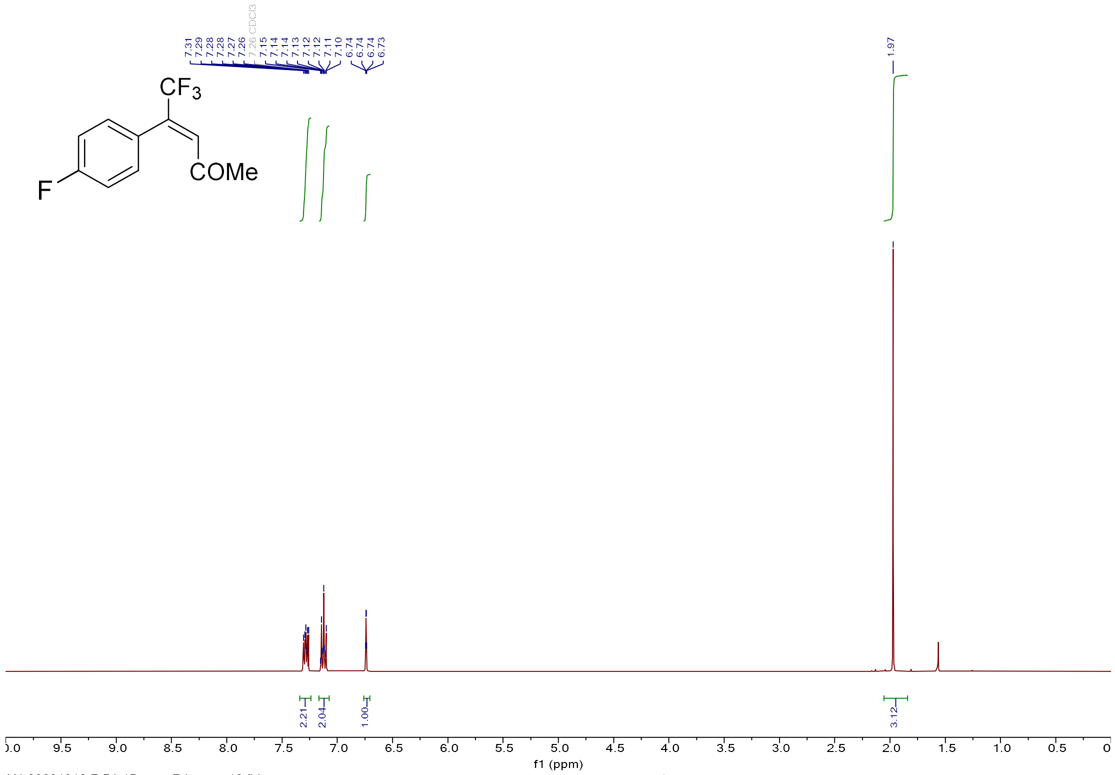

YJ-20201012-7-54-15-para-F-ketone.12.fid

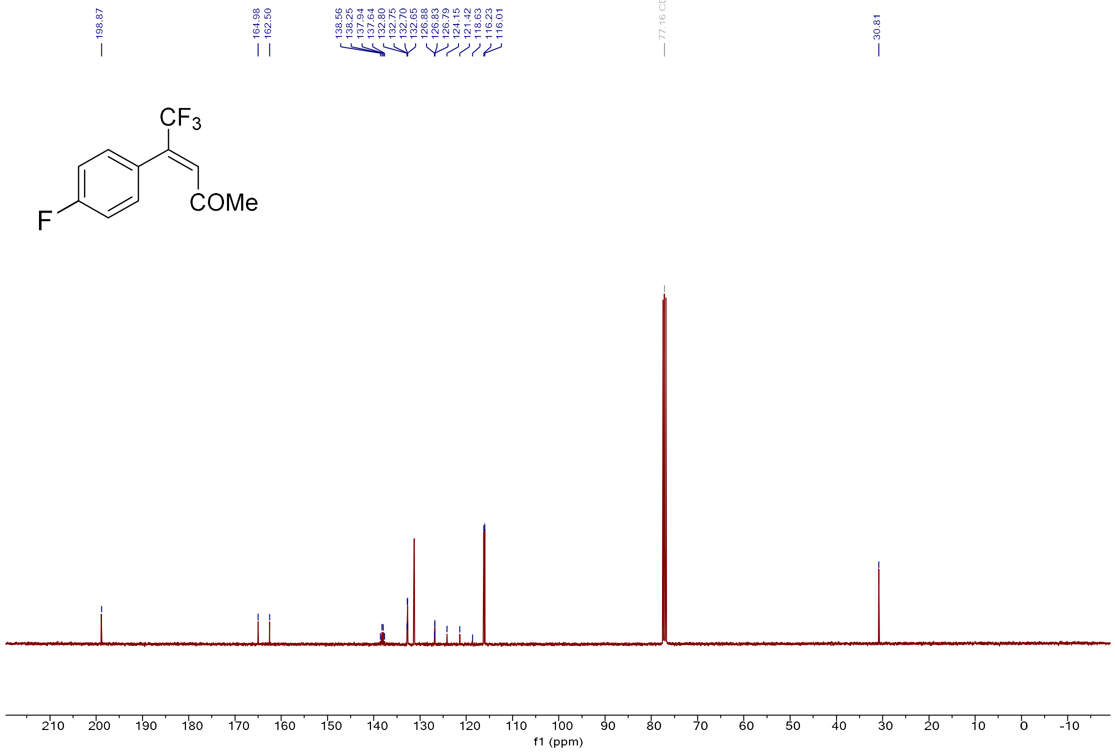

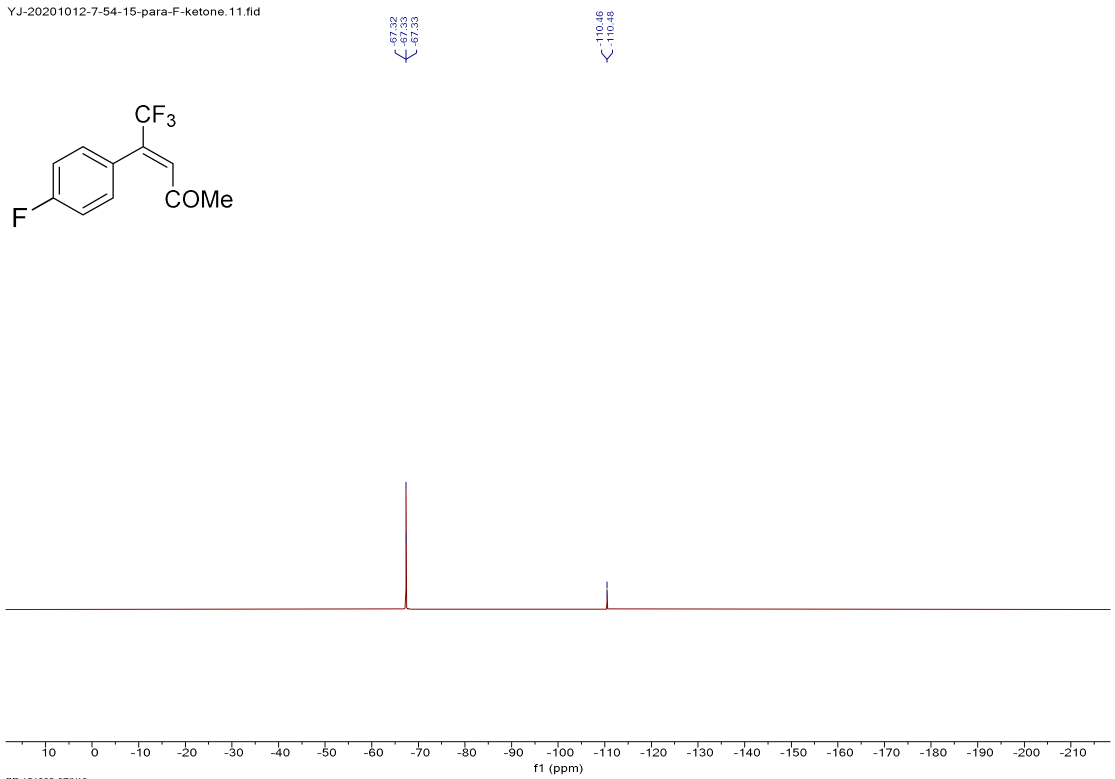
YJ-20201012-7-54-15-para-F-ketone.11.fid
-67.32
-67.33
-67.33
-110.46
-110.48
CF3
F
COMe
10 0 -10 -20 -30 -40 -50 -60 -70 -80 -90 -100 -110 -120 -130 -140 -150 -160 -170 -180 -190 -200 -210
f1 (ppm)

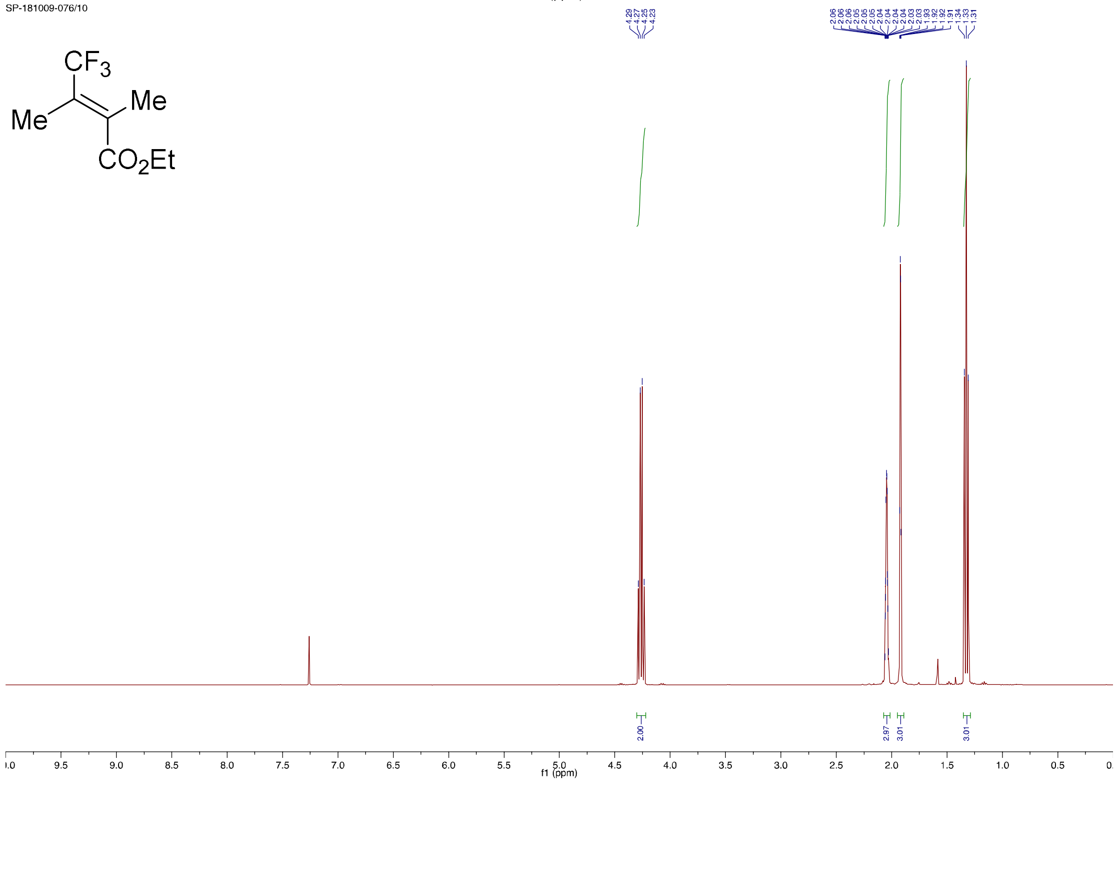
SP-181009-076/10
CF3
Me
Me
CO2Et
2.00
2.97
3.01
3.01
9.5 9.0 8.5 8.0 7.5 7.0 6.5 6.0 5.5 5.0 4.5 4.0 3.5 3.0 2.5 2.0 1.5 1.0 0.5 0
f1 (ppm)

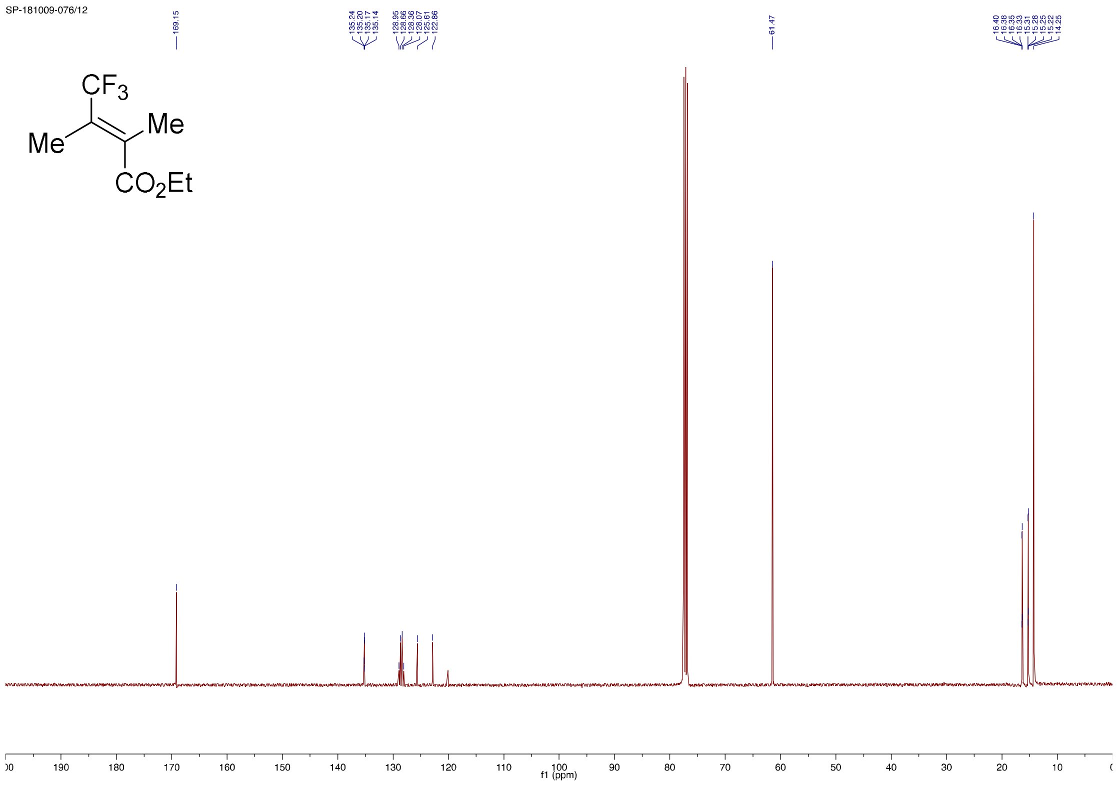
SP-181009-076/12
169.15
135.24
135.20
135.17
135.14
128.95
128.66
128.36
128.07
125.81
122.86
61.47
16.40
16.38
16.35
16.33
15.31
15.28
15.25
15.22
14.25
CF3
Me
Me
CO2Et
f1 (ppm)

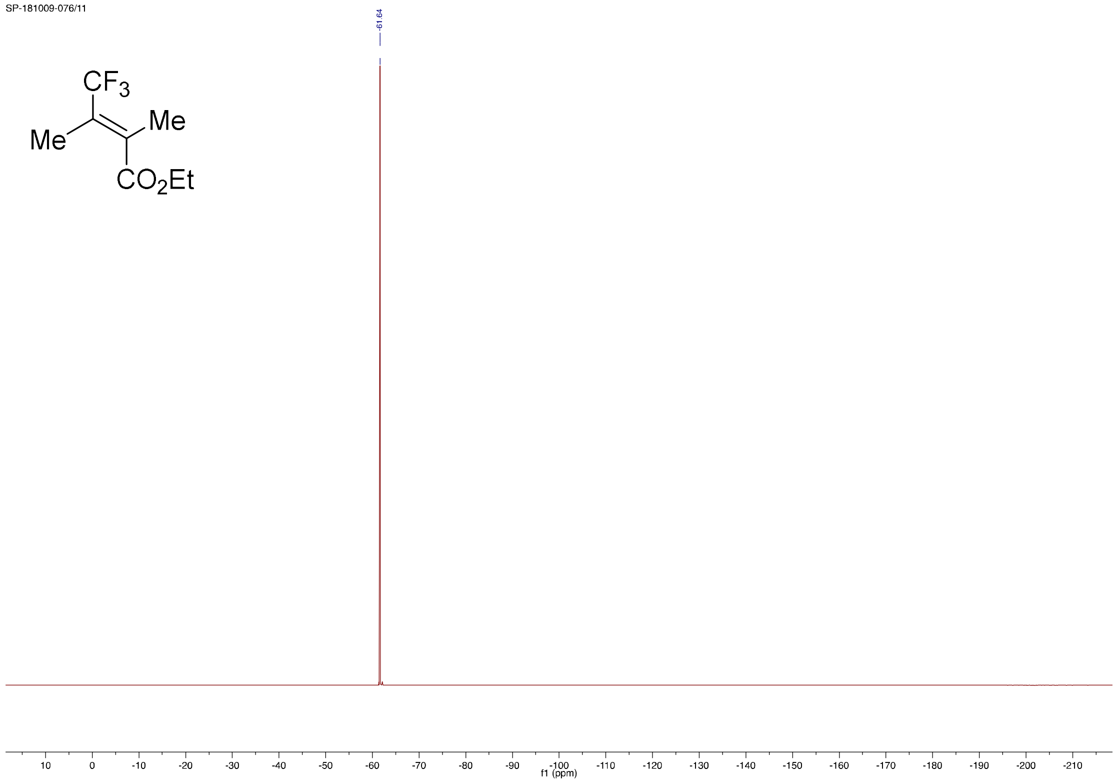
SP-181009-076/11
-61.64
CF3
Me
Me
CO2Et
f1 (ppm)

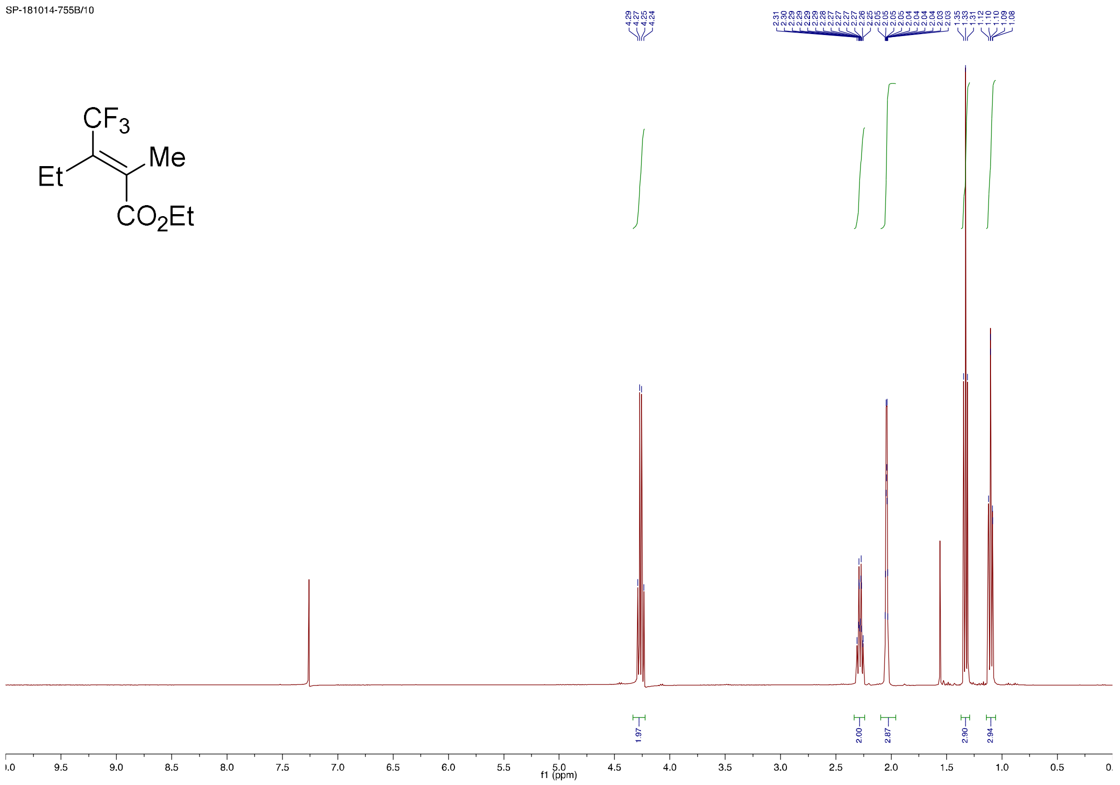
SP-181014-755B/10
CF3
Me
Et
CO2Et
f1 (ppm)

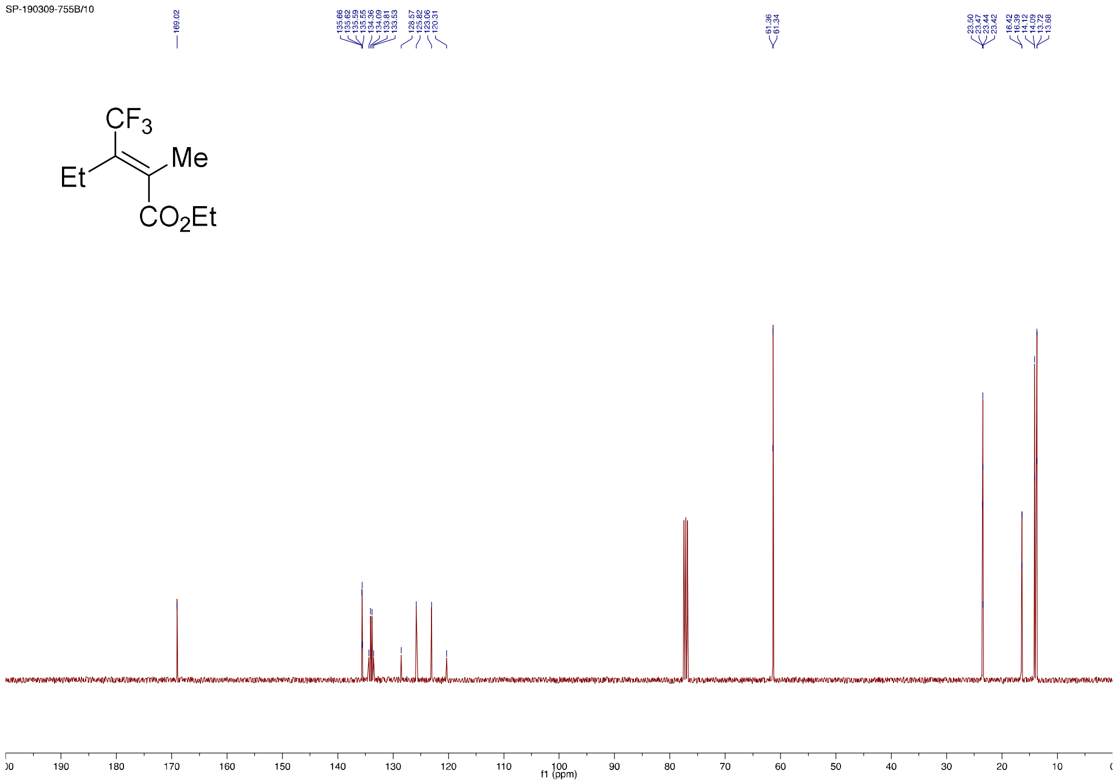
SP-190309-755B/10
CF3
Me
Et
CO2Et
f1 (ppm)

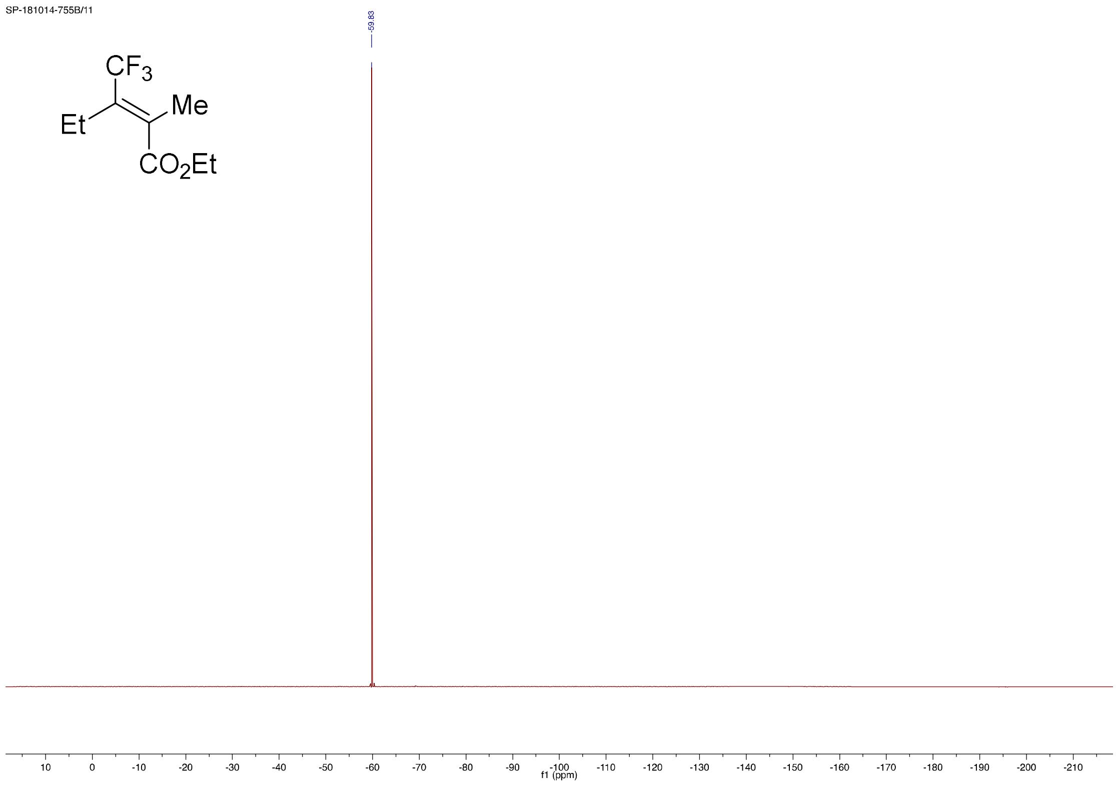
SP-181014-755B/11
-59.83
CF3
Me
Et
CO2Et
f1 (ppm)

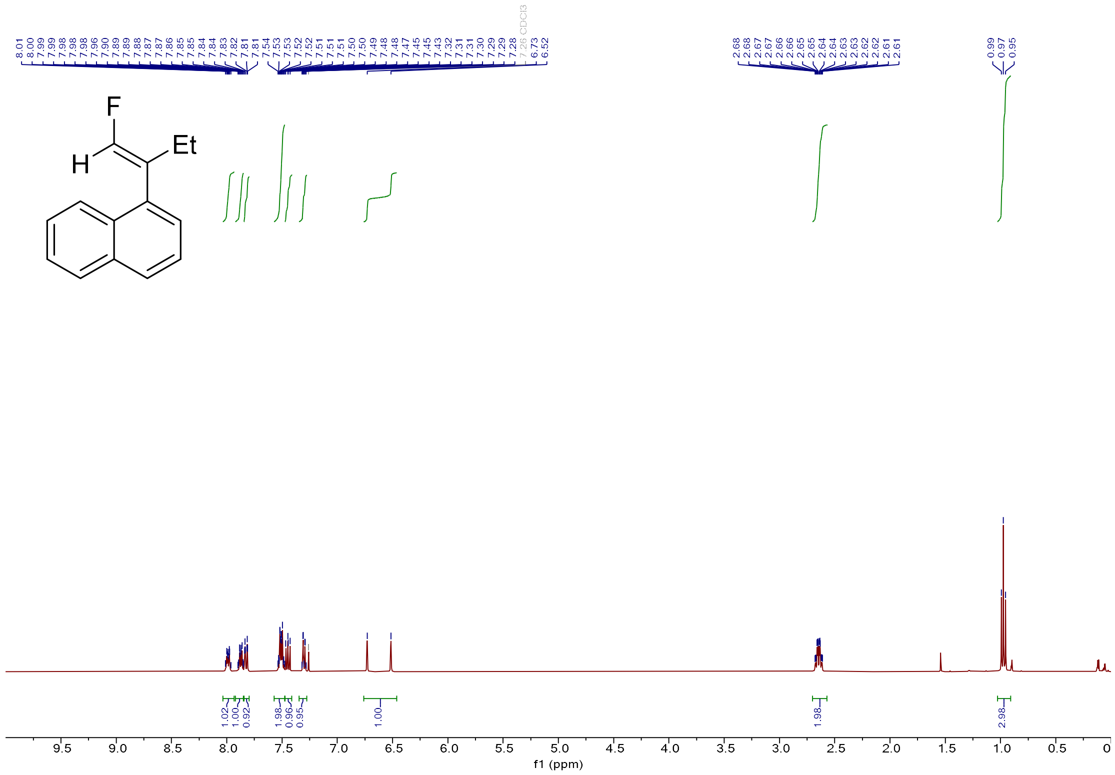
F
H
Et
7.26 CDCl3
f1 (ppm)

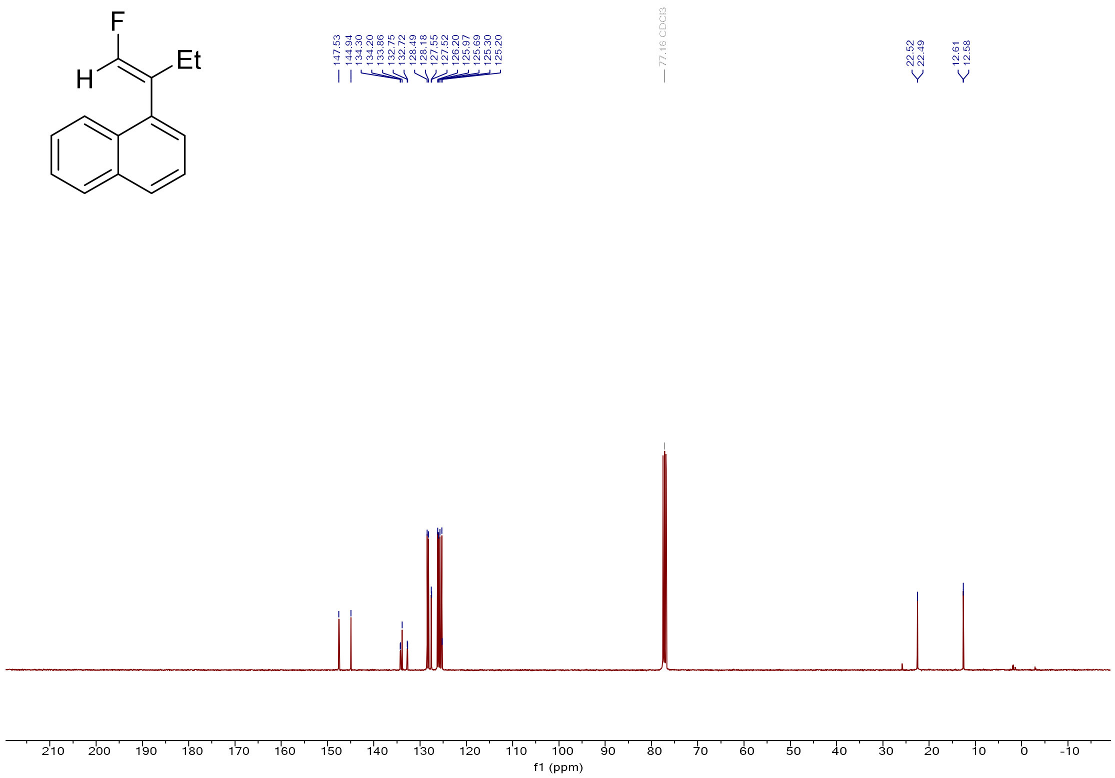

F
H
Et
147.53
144.94
134.30
134.20
133.86
132.75
132.72
128.49
128.18
127.55
127.52
126.20
125.97
125.69
125.30
125.20
77.16 CDCl3
22.52
22.49
12.61
12.58
210
200
190
180
170
160
150
140
130
120
110
100
90
80
70
60
50
40
30
20
10
0
-10
f1 (ppm)

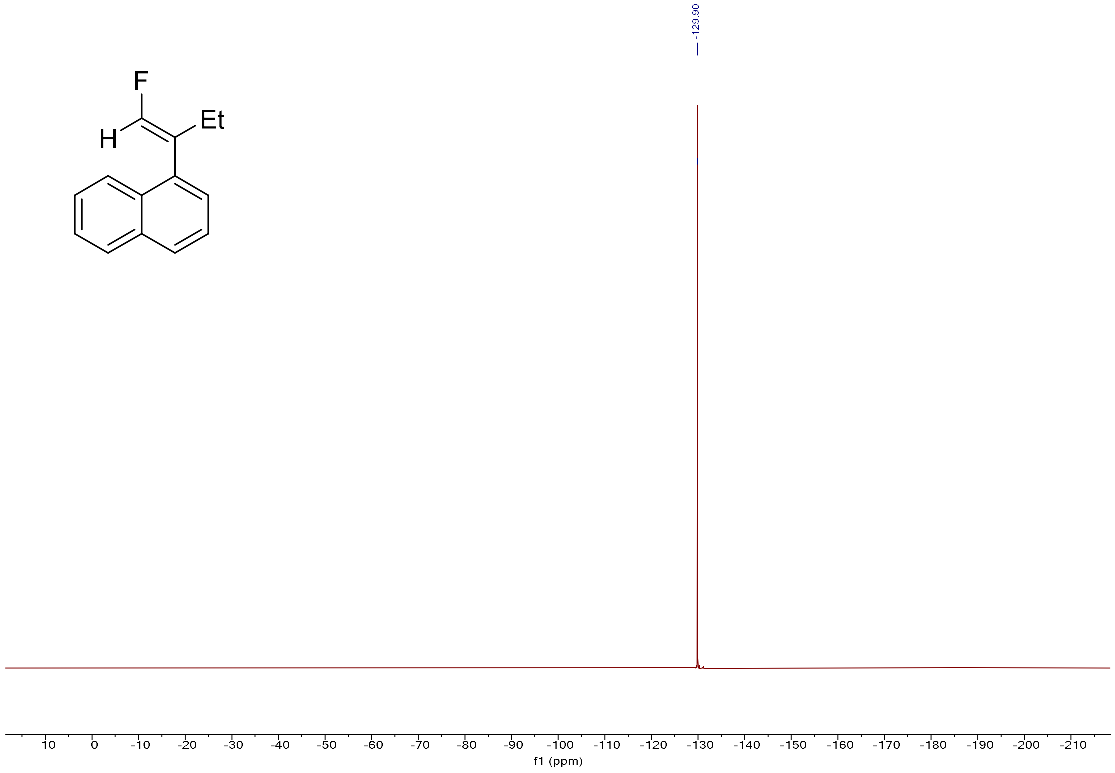

-129.90
F
H
Et
10
0
-10
-20
-30
-40
-50
-60
-70
-80
-90
-100
-110
-120
-130
-140
-150
-160
-170
-180
-190
-200
-210
f1 (ppm)

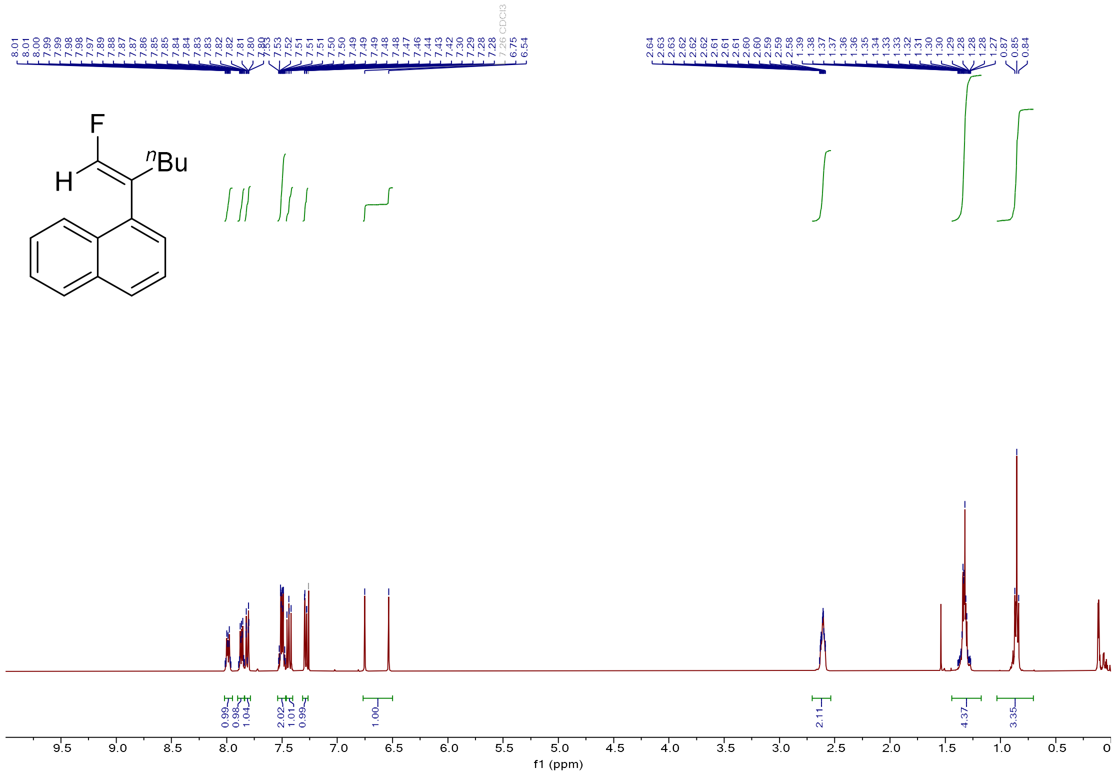

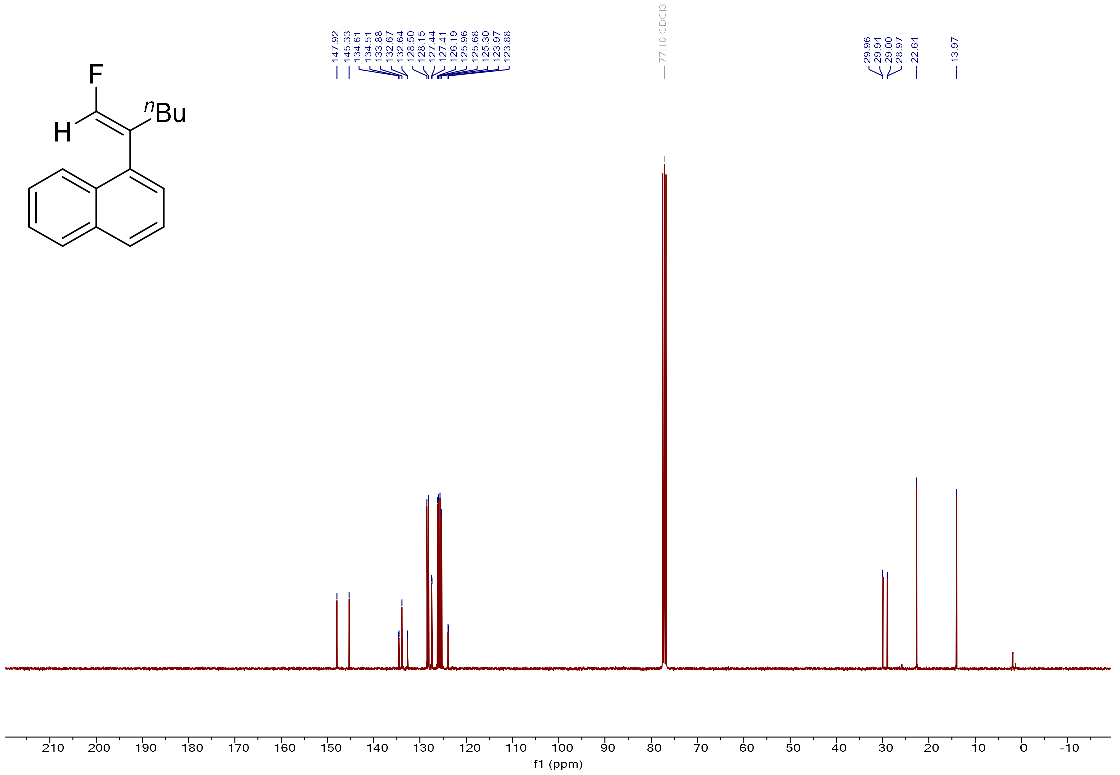

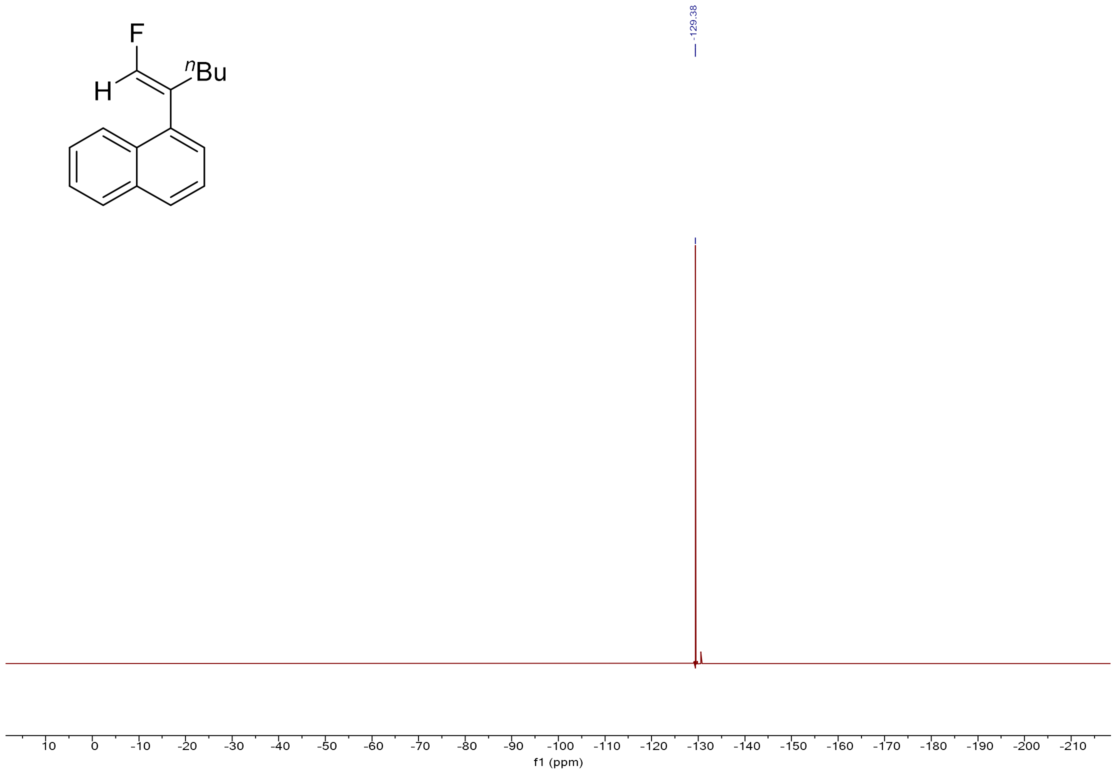
F
H
nBu
-129.38
f1 (ppm)

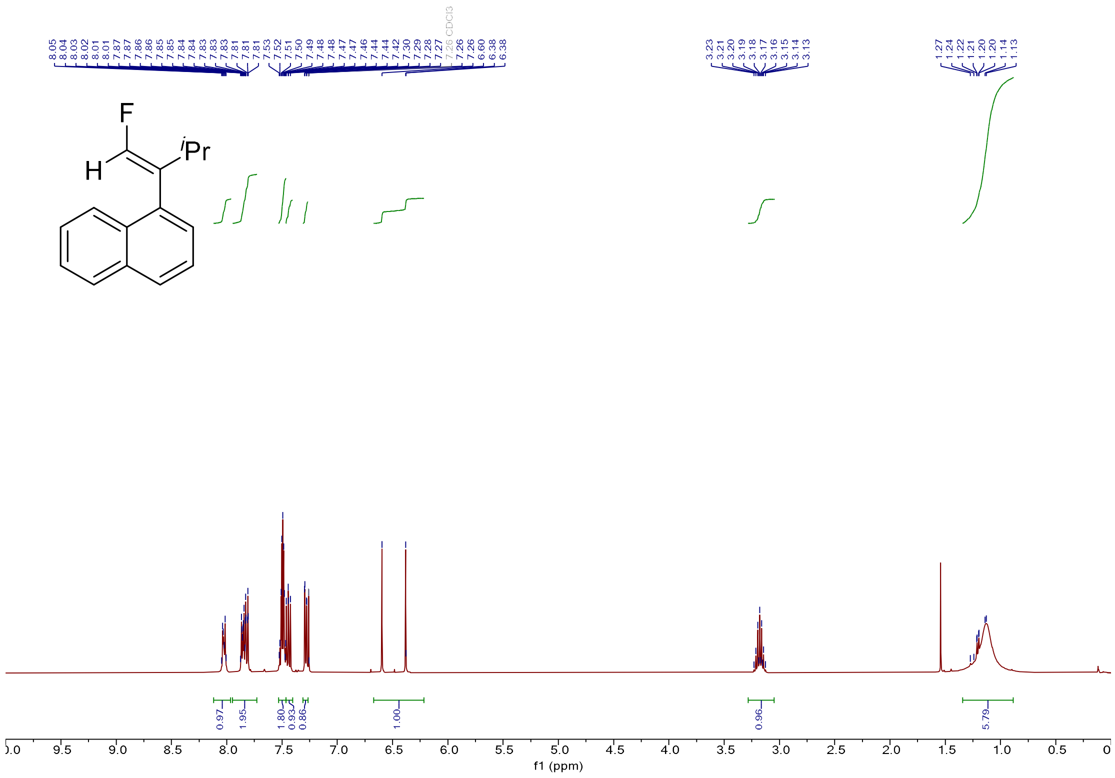
F
H
iPr
CDCl3
f1 (ppm)

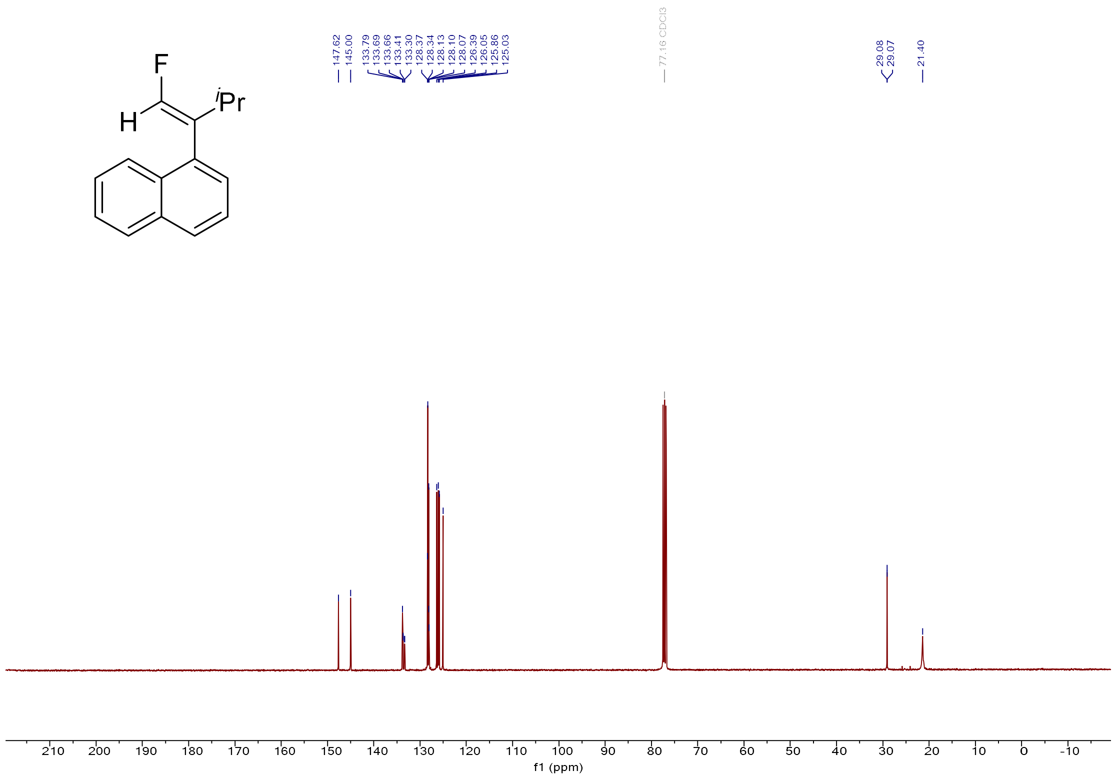
F
H
iPr
147.62
145.00
133.79
133.69
133.66
133.41
133.30
128.37
128.34
128.13
128.10
128.07
126.39
126.05
125.86
125.03
77.16 CDCl3
29.08
29.07
21.40
f1 (ppm)

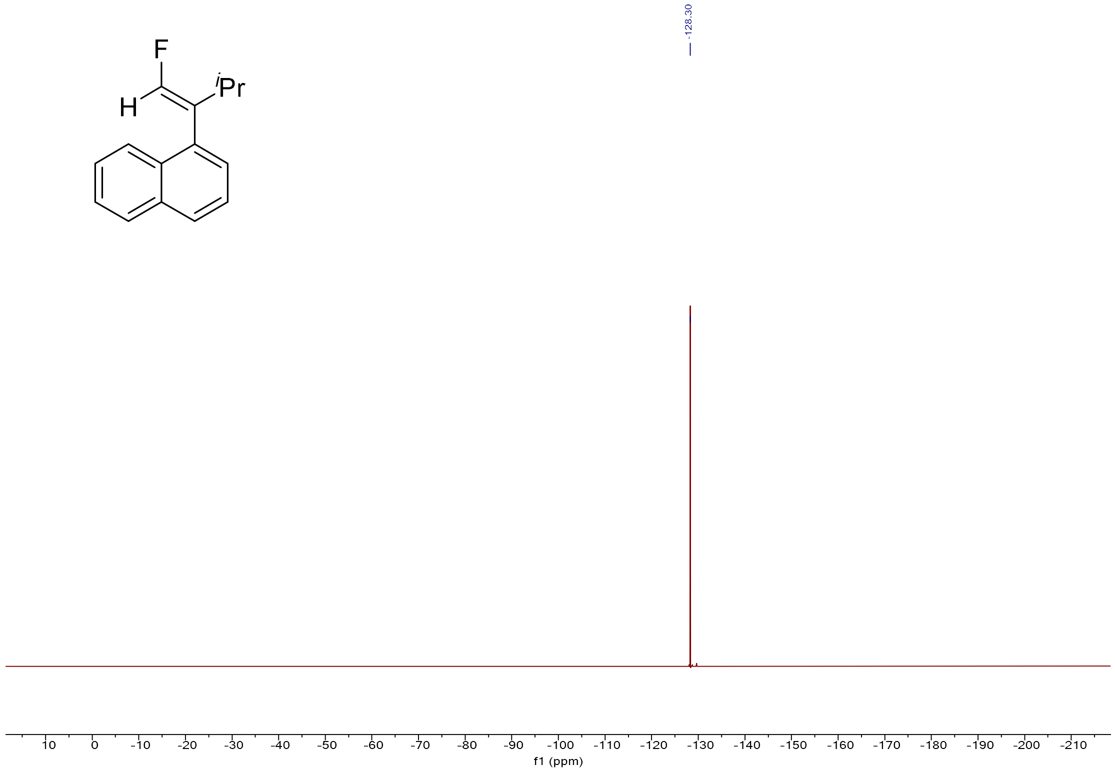
F
H
iPr
-128.30
f1 (ppm)

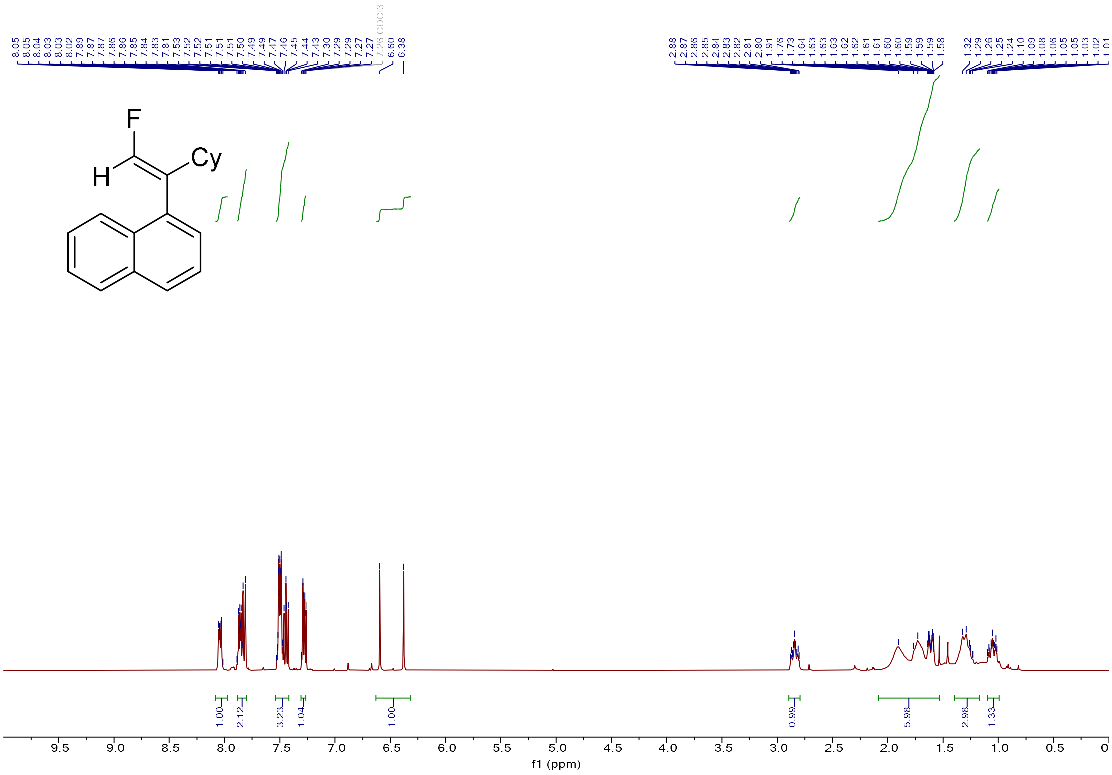
8.05 8.05 8.04 8.03 8.03 8.02 7.89 7.87 7.87 7.86 7.85 7.84 7.83 7.81 7.53 7.52 7.52 7.51 7.51 7.50 7.49 7.47 7.46 7.45 7.44 7.43 7.30 7.29 7.29 7.27 7.27 7.26 CDCl3 6.60 6.38
2.88 2.87 2.86 2.85 2.84 2.83 2.82 2.81 2.80 1.91 1.76 1.73 1.64 1.63 1.63 1.63 1.62 1.62 1.61 1.61 1.60 1.60 1.59 1.59 1.59 1.58 1.32 1.29 1.26 1.25 1.24 1.10 1.09 1.08 1.06 1.05 1.05 1.03 1.02 1.01
F
H
Cy
1.00 2.12 3.23 1.04 1.00 0.99 5.98 2.98 1.33
9.5 9.0 8.5 8.0 7.5 7.0 6.5 6.0 5.5 5.0 4.5 4.0 3.5 3.0 2.5 2.0 1.5 1.0 0.5 0
f1 (ppm)

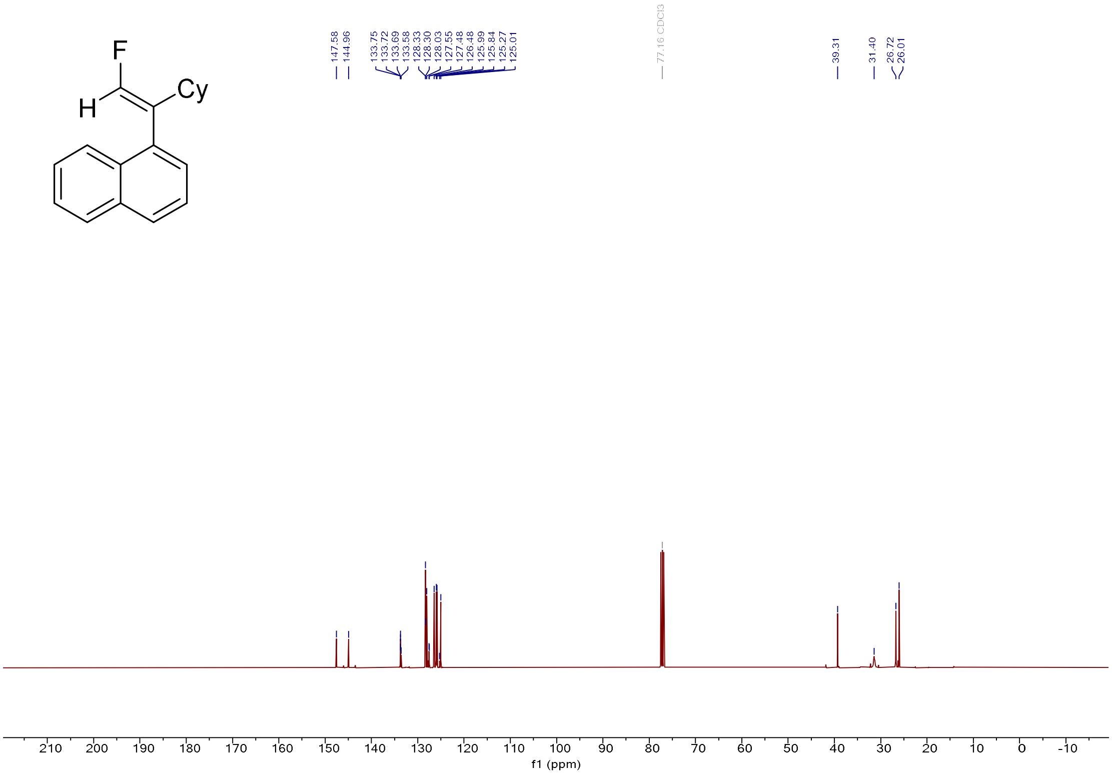
147.58 144.96 133.75 133.72 133.69 133.58 128.33 128.30 128.03 127.55 127.48 126.48 125.99 125.84 125.27 125.01
77.16 CDCl3
39.31 31.40 26.72 26.01
F
H
Cy
210 200 190 180 170 160 150 140 130 120 110 100 90 80 70 60 50 40 30 20 10 0 -10
f1 (ppm)

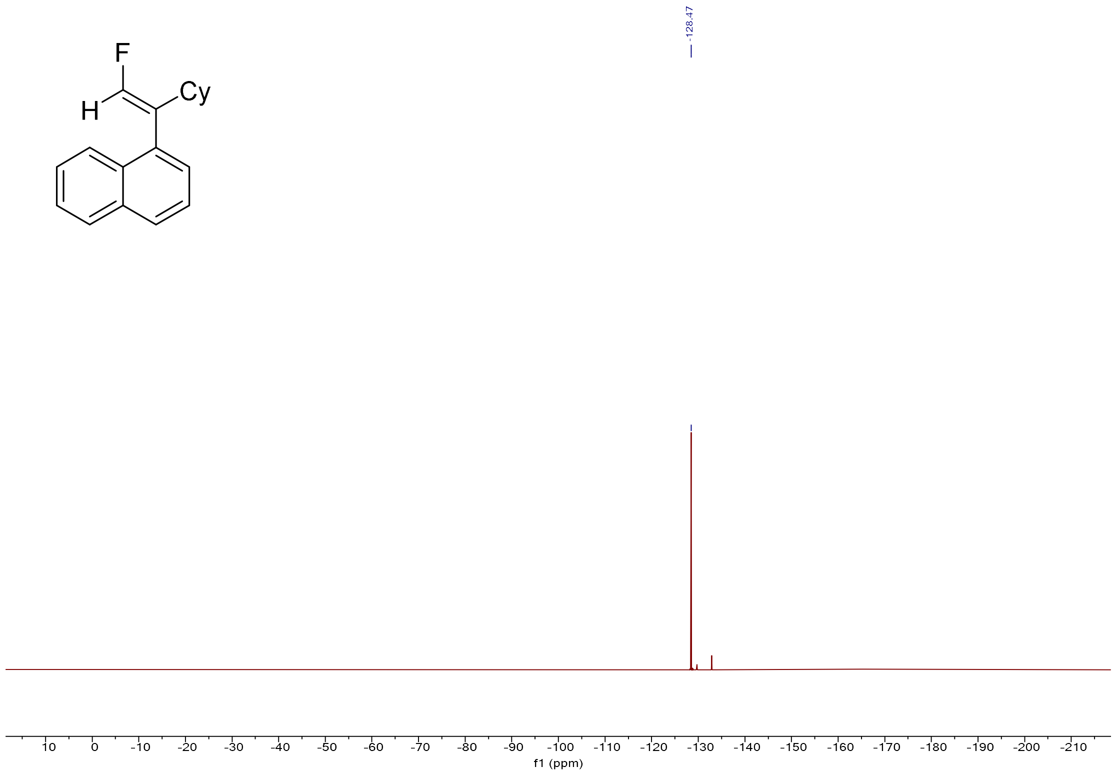
F
H
Cy
-128.47
f1 (ppm)

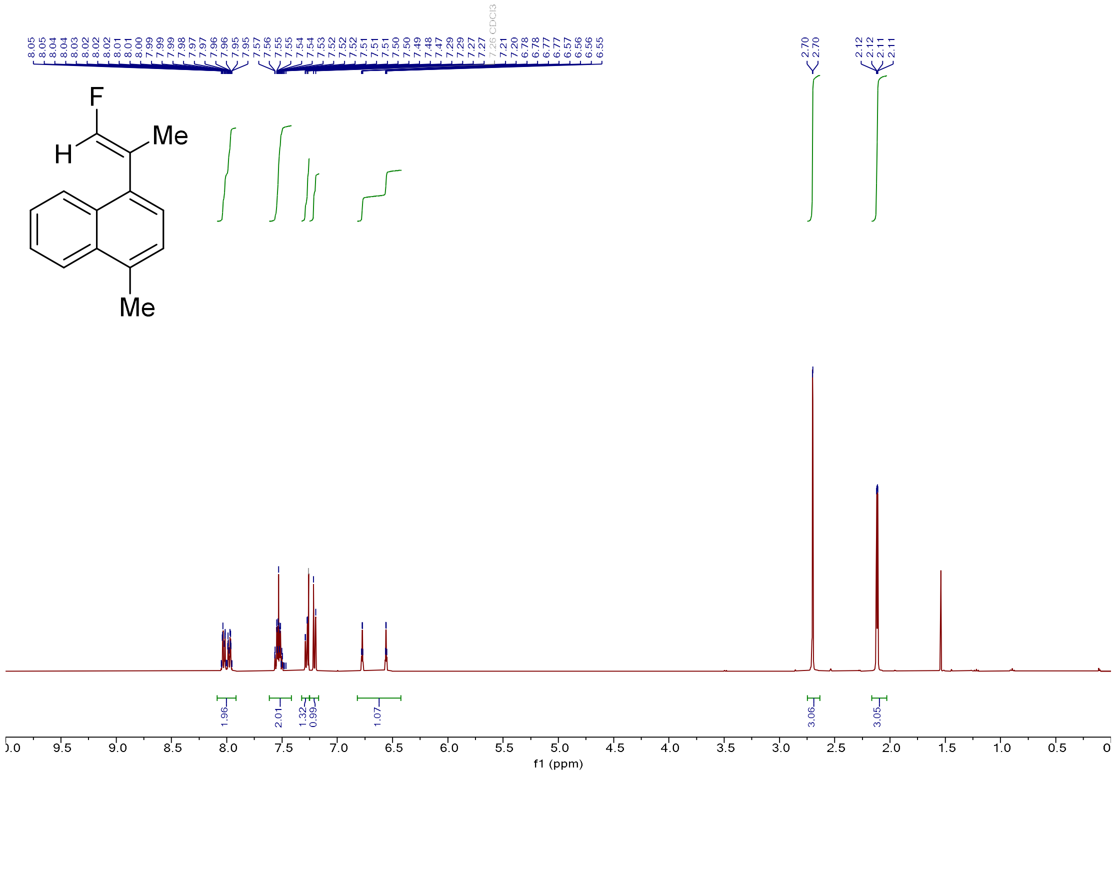
F
H
Me
Me
f1 (ppm)

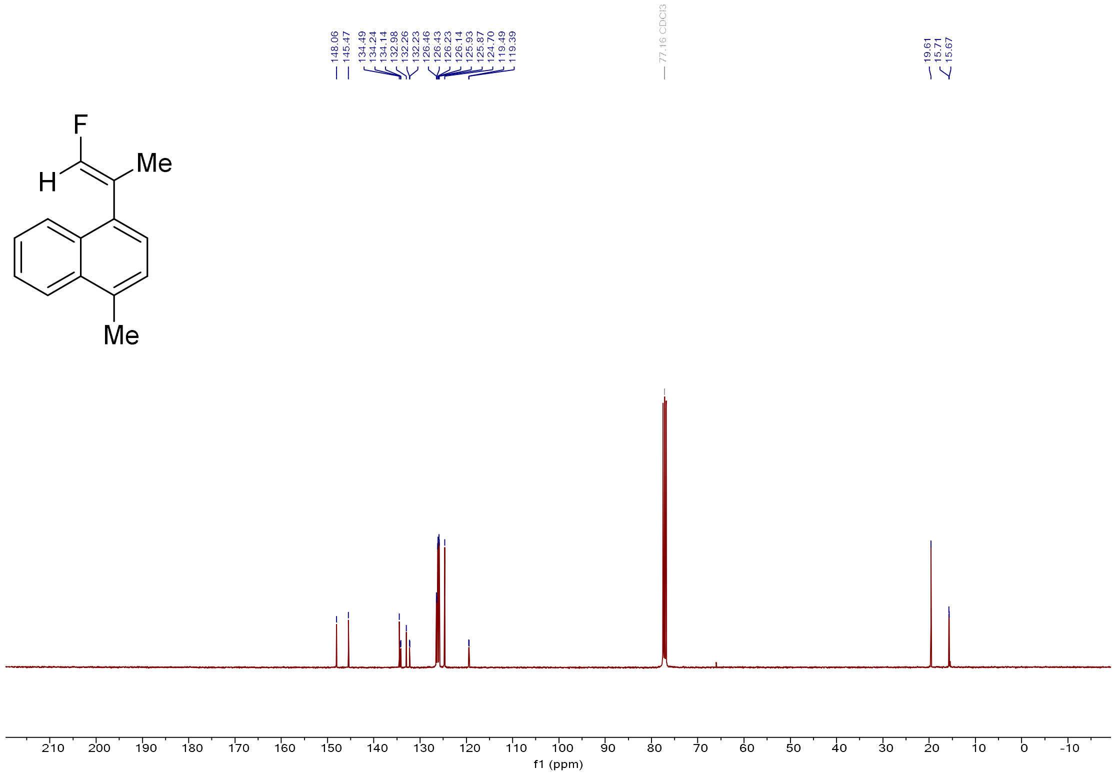
148.06
145.47
134.49
134.24
134.14
132.98
132.26
132.23
126.46
126.43
126.23
126.14
125.93
125.87
124.70
119.49
119.39
77.16 CDCl3
19.61
15.71
15.67
F
H
Me
Me
f1 (ppm)

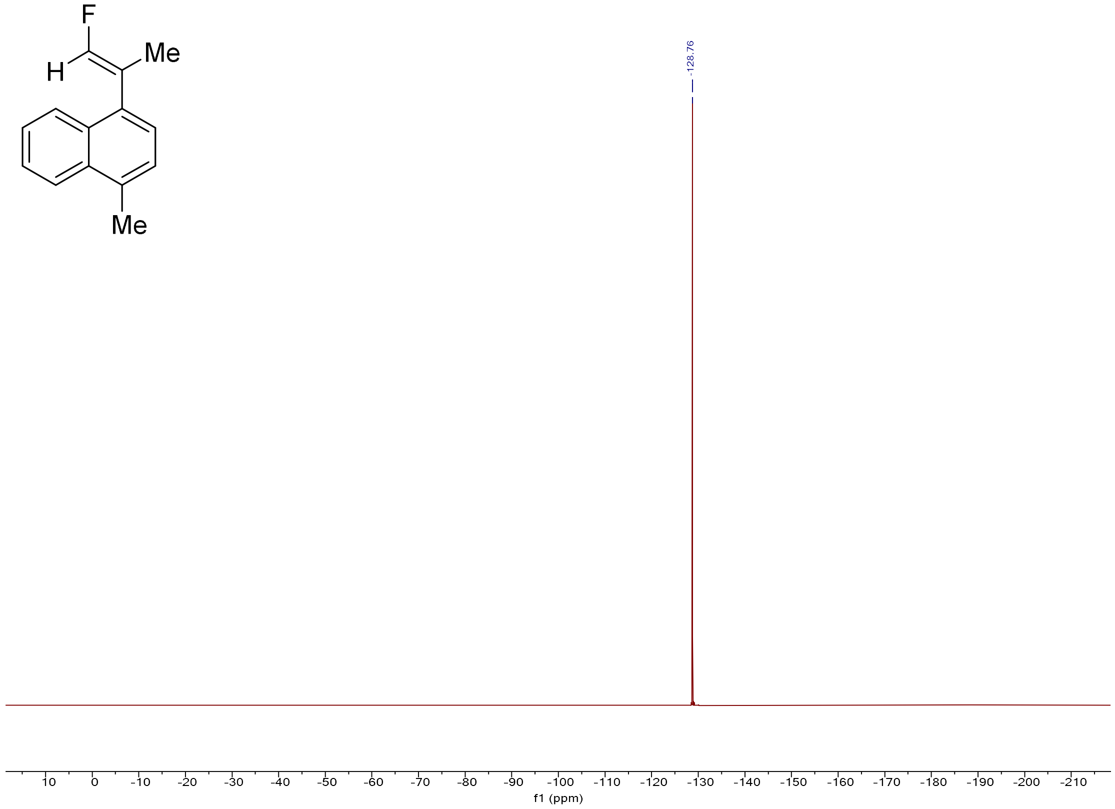
-128.76
F
H
Me
Me
f1 (ppm)

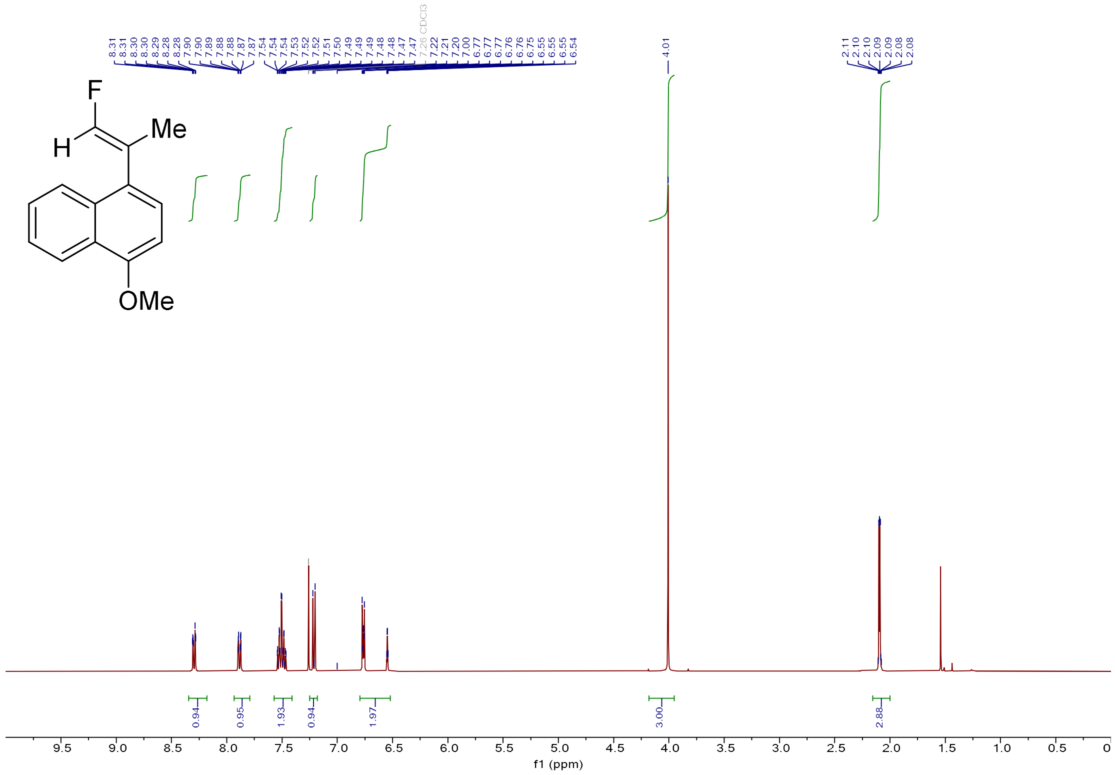

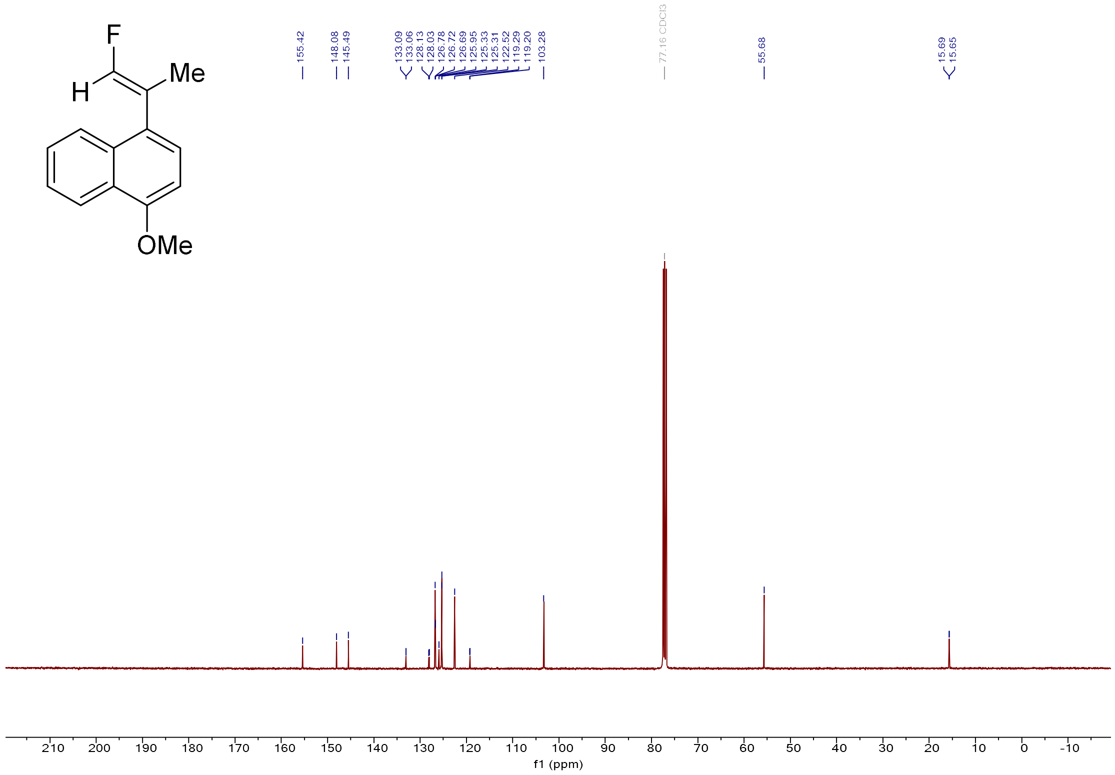

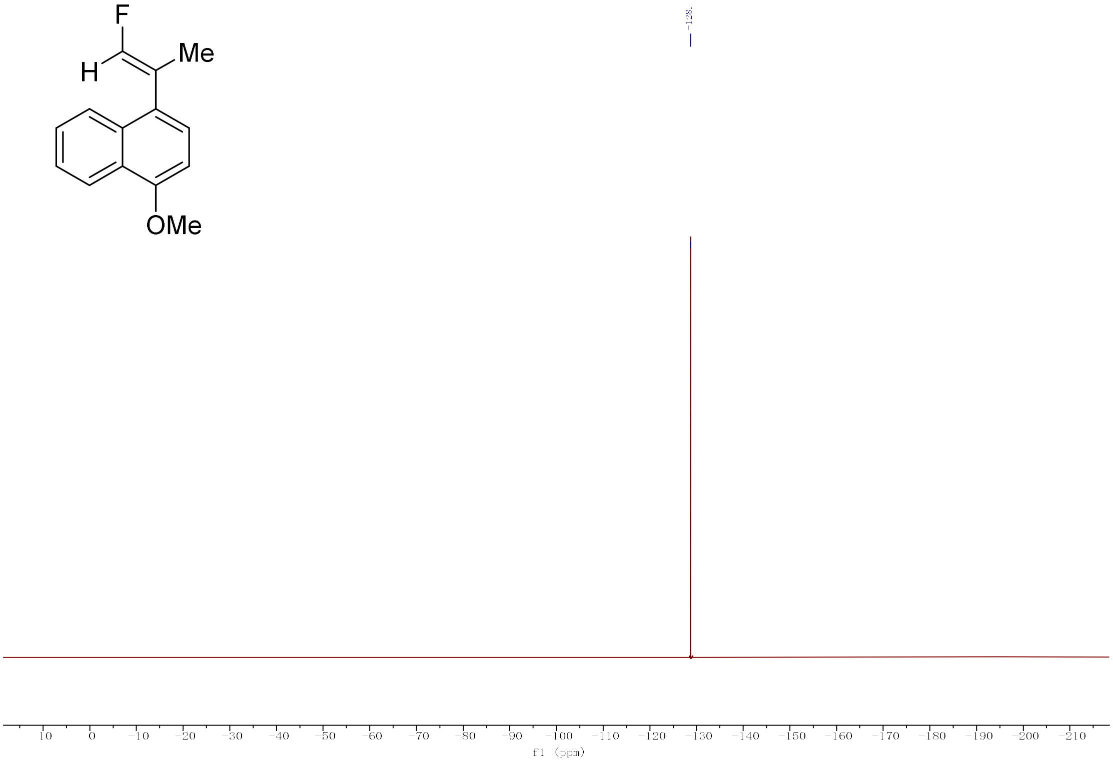
F
Me
H
OMe
-128.
10 0 -10 -20 -30 -40 -50 -60 -70 -80 -90 -100 -110 -120 -130 -140 -150 -160 -170 -180 -190 -200 -210
f1 (ppm)

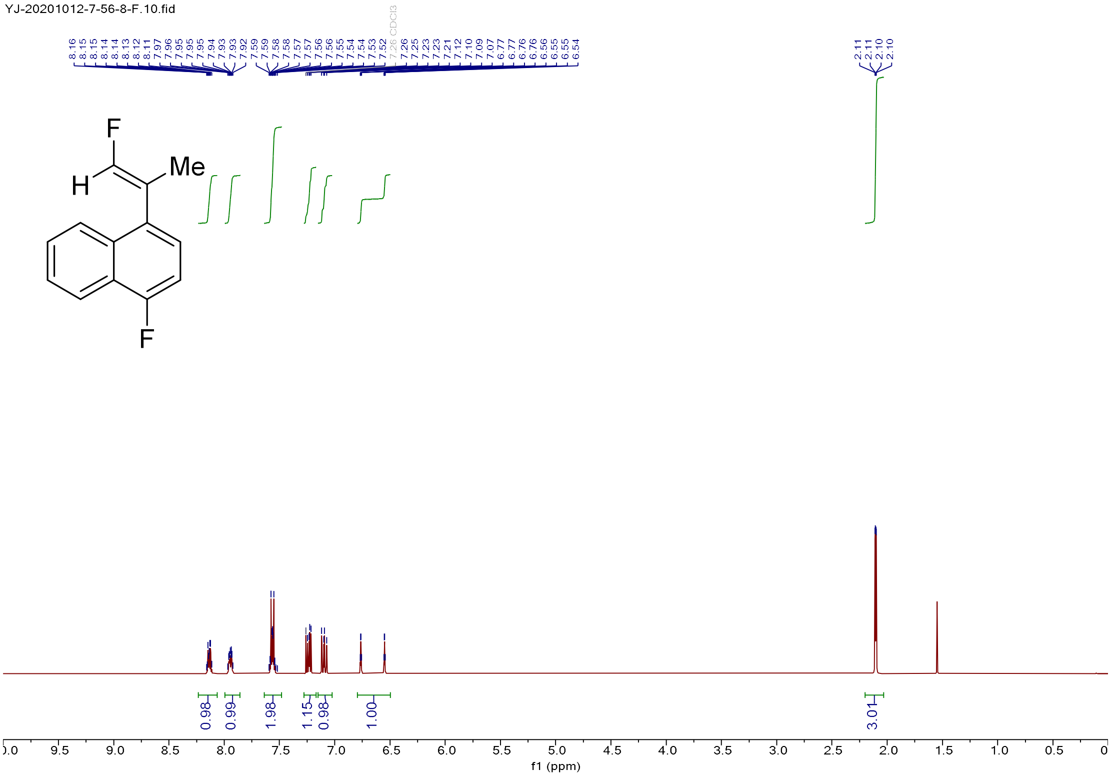
YJ-20201012-7-56-8-F.10.fid
8.16 8.15 8.15 8.14 8.14 8.13 8.12 8.11 7.97 7.96 7.95 7.95 7.95 7.94 7.93 7.93 7.92 7.59 7.59 7.58 7.58 7.57 7.57 7.56 7.56 7.55 7.54 7.54 7.53 7.52 7.26 CDCl3 7.26 7.25 7.23 7.23 7.21 7.12 7.10 7.09 7.07 6.77 6.77 6.76 6.76 6.56 6.55 6.55 6.54
2.11 2.11 2.10 2.10
F
Me
H
F
0.98 0.99 1.98 1.15 0.98 1.00 3.01
10.0 9.5 9.0 8.5 8.0 7.5 7.0 6.5 6.0 5.5 5.0 4.5 4.0 3.5 3.0 2.5 2.0 1.5 1.0 0.5 0
f1 (ppm)

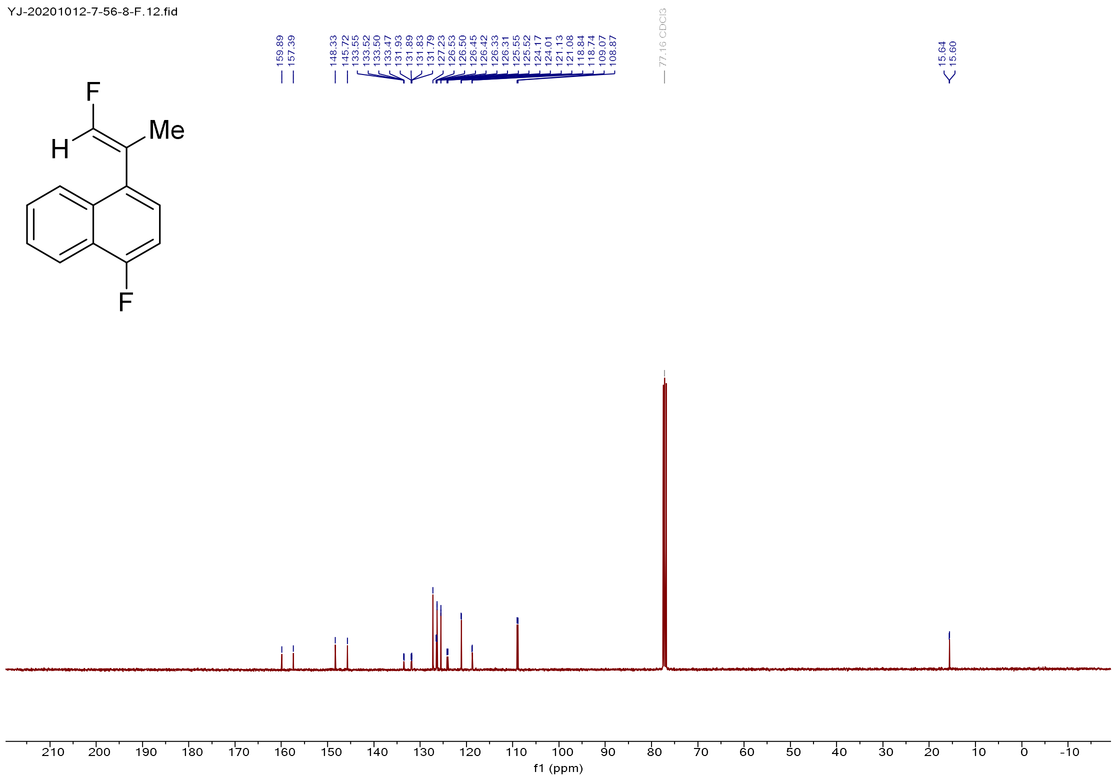
YJ-20201012-7-56-8-F.12.fid
F
H
Me
F
159.89
157.39
148.33
145.72
133.55
133.52
133.50
133.47
131.93
131.89
131.83
131.79
127.23
126.53
126.50
126.45
126.42
126.33
126.31
125.55
125.52
124.17
124.01
121.13
121.08
118.84
118.74
109.07
108.87
77.16 CDCl3
15.64
15.60
210
200
190
180
170
160
150
140
130
120
110
100
90
80
70
60
50
40
30
20
10
0
-10
f1 (ppm)

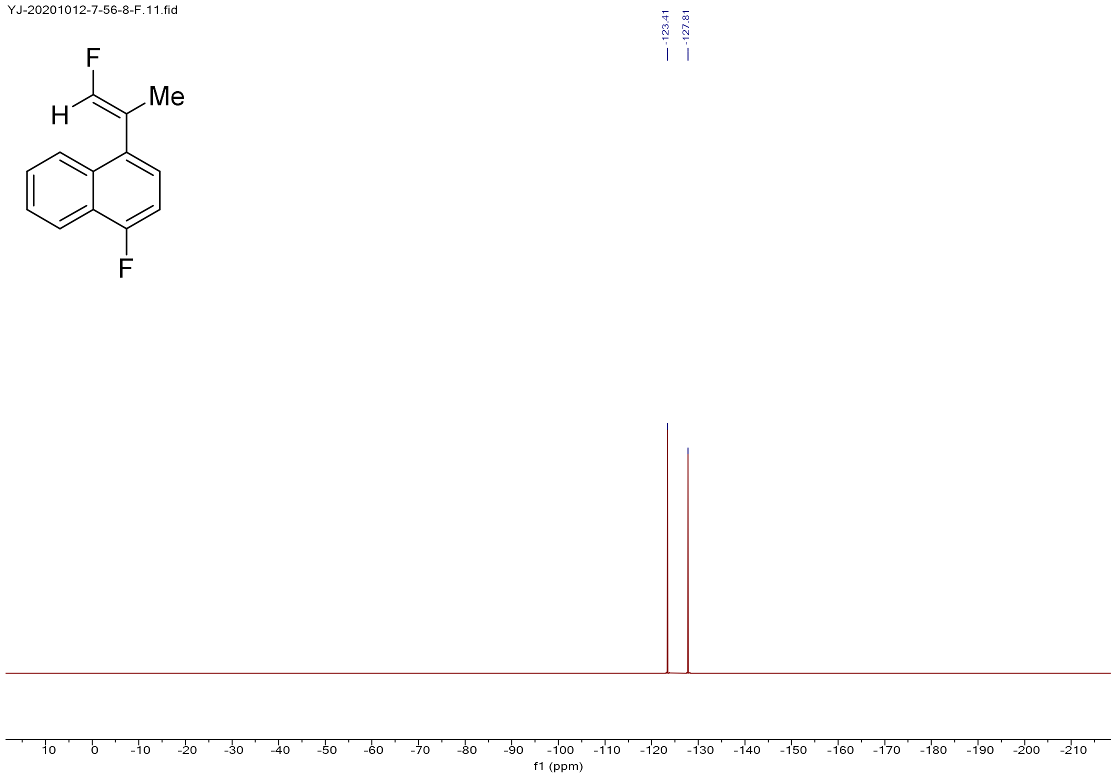
YJ-20201012-7-56-8-F.11.fid
F
H
Me
F
-123.41
-127.81
10
0
-10
-20
-30
-40
-50
-60
-70
-80
-90
-100
-110
-120
-130
-140
-150
-160
-170
-180
-190
-200
-210
f1 (ppm)

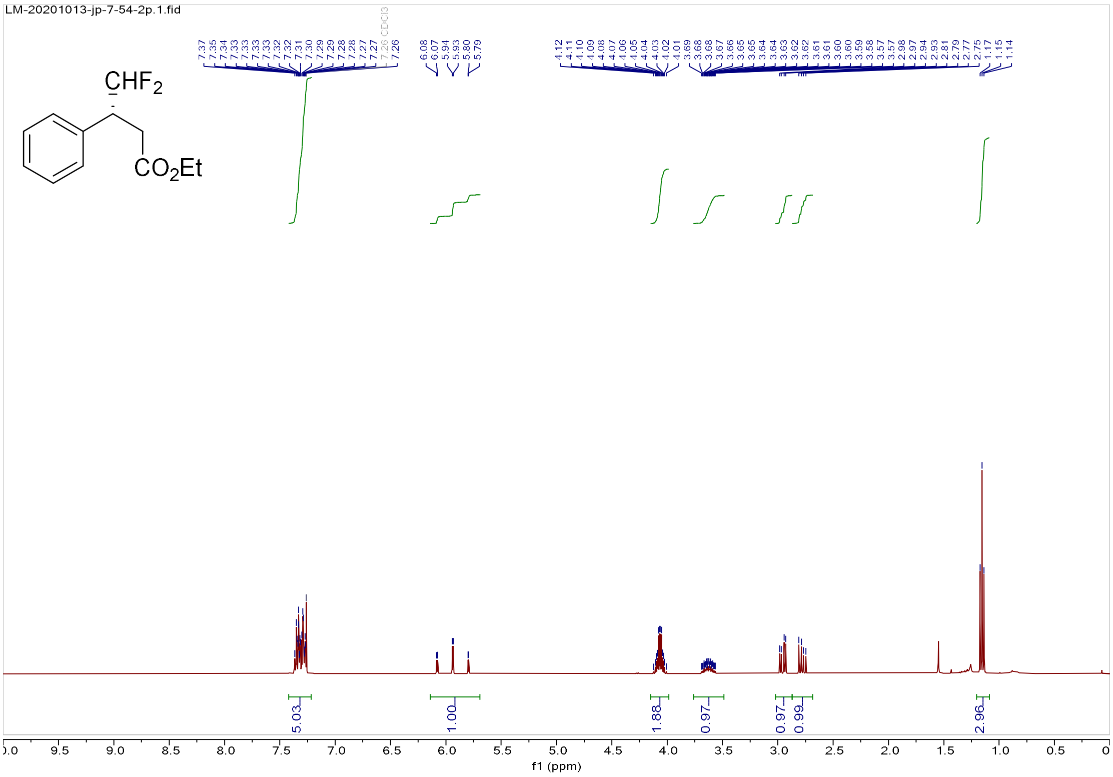
LM-20201013-jp-7-54-2p.1.fid
CHF2
CO2Et
f1 (ppm)

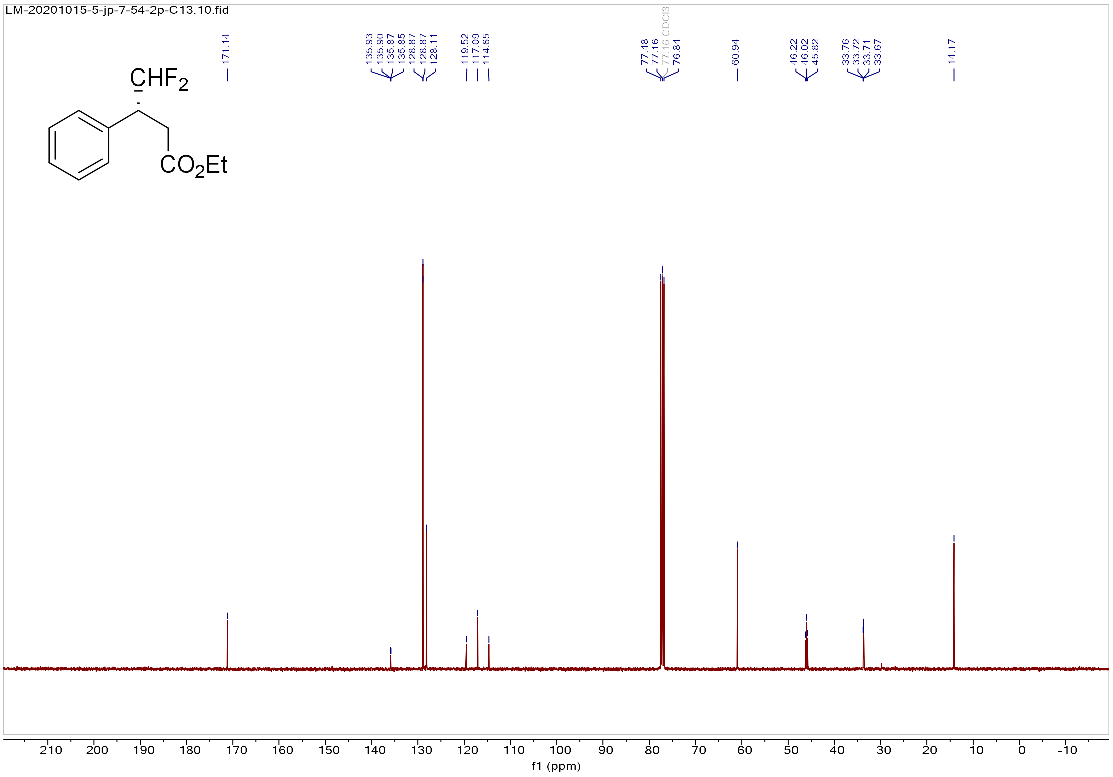
LM-20201015-5-jp-7-54-2p-C13.10.fid
CHF2
CO2Et
f1 (ppm)

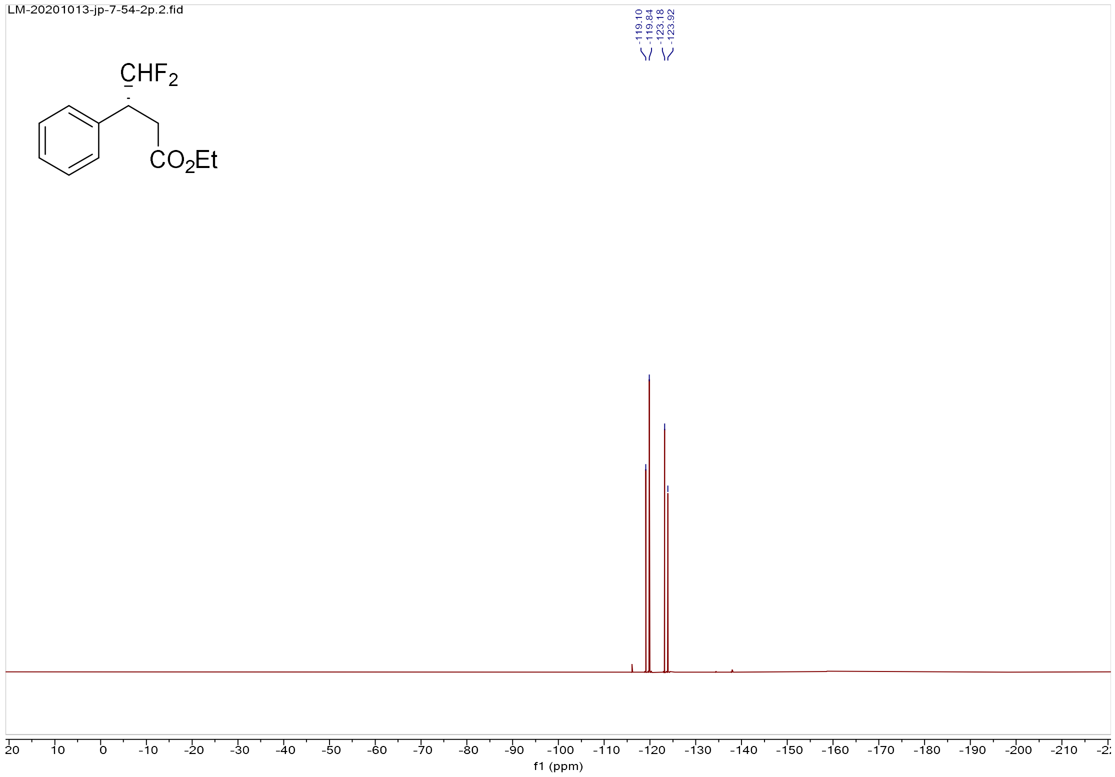
LM-20201013-jp-7-54-2p.2.fid
-119.10
-119.84
-123.18
-123.92
CHF2
CO2Et
f1 (ppm)

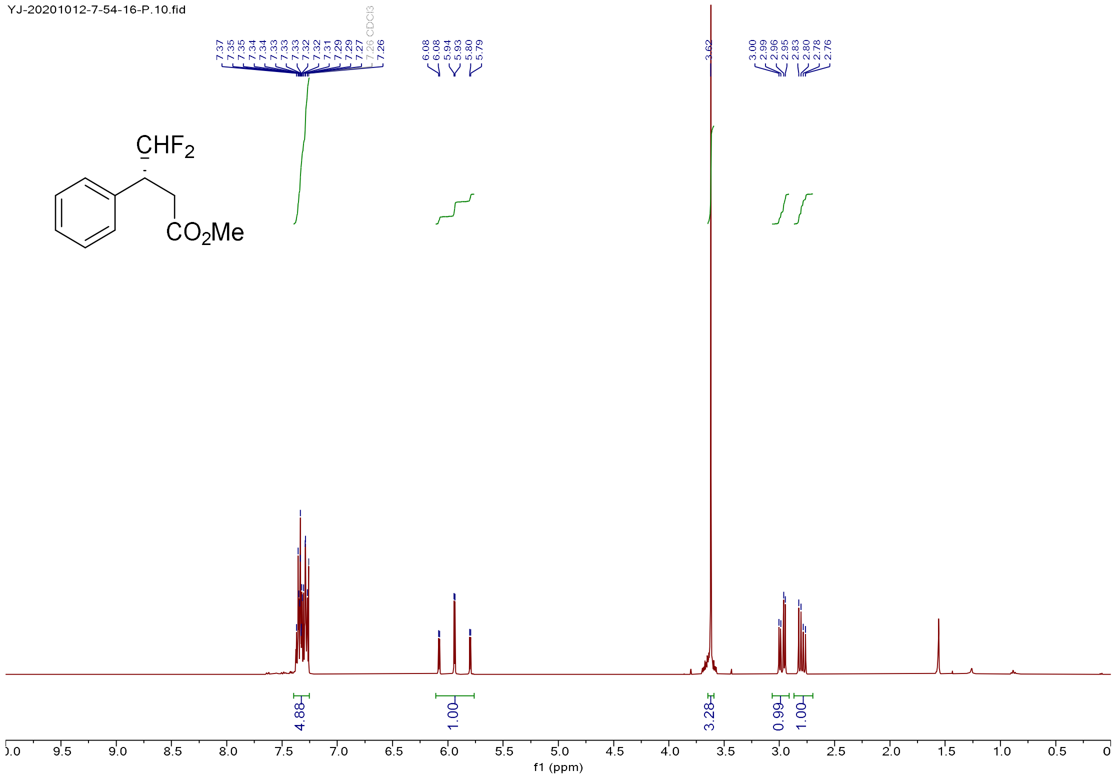
YJ-20201012-7-54-16-P.10.fid
7.37
7.35
7.35
7.34
7.34
7.33
7.33
7.33
7.32
7.32
7.31
7.29
7.29
7.27
7.26 CDCl3
7.26
6.08
6.08
5.94
5.93
5.80
5.79
3.62
3.00
2.99
2.96
2.95
2.83
2.80
2.78
2.76
CHF2
CO2Me
4.88
1.00
3.28
0.99
1.00
f1 (ppm)

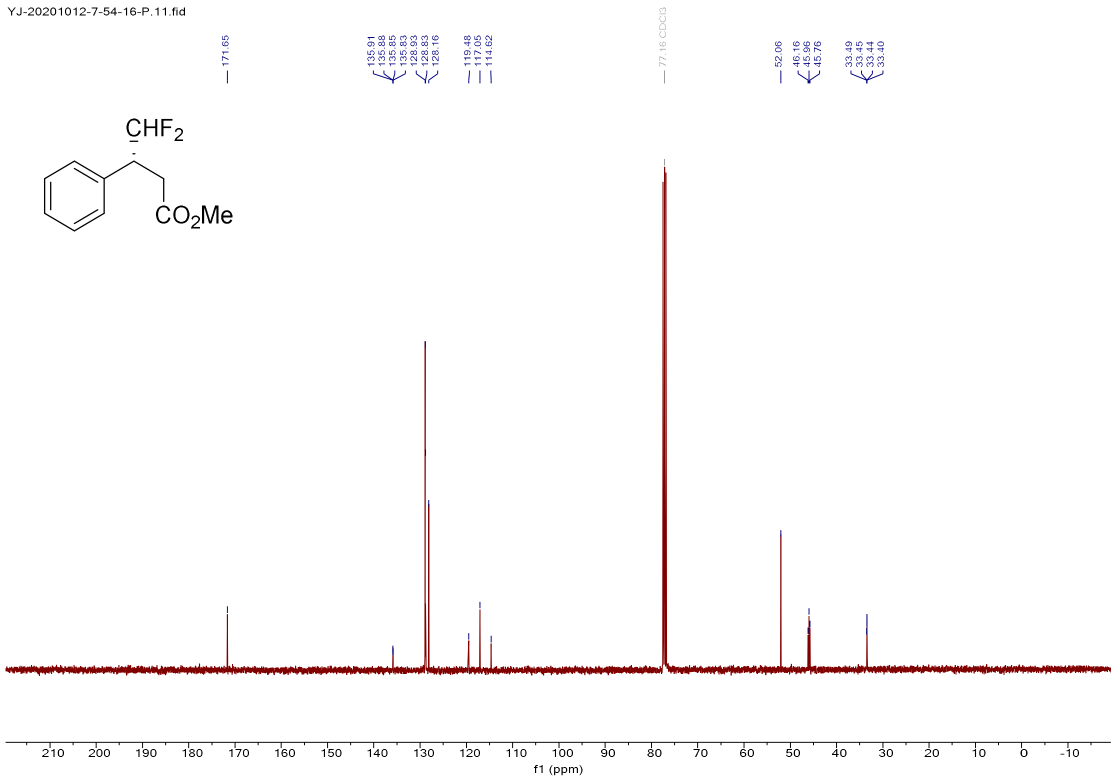
YJ-20201012-7-54-16-P.11.fid
171.65
135.91
135.88
135.85
135.83
128.93
128.83
128.16
119.48
117.05
114.62
77.16 CDCl3
52.06
46.16
45.96
45.76
33.49
33.45
33.44
33.40
CHF2
CO2Me
f1 (ppm)

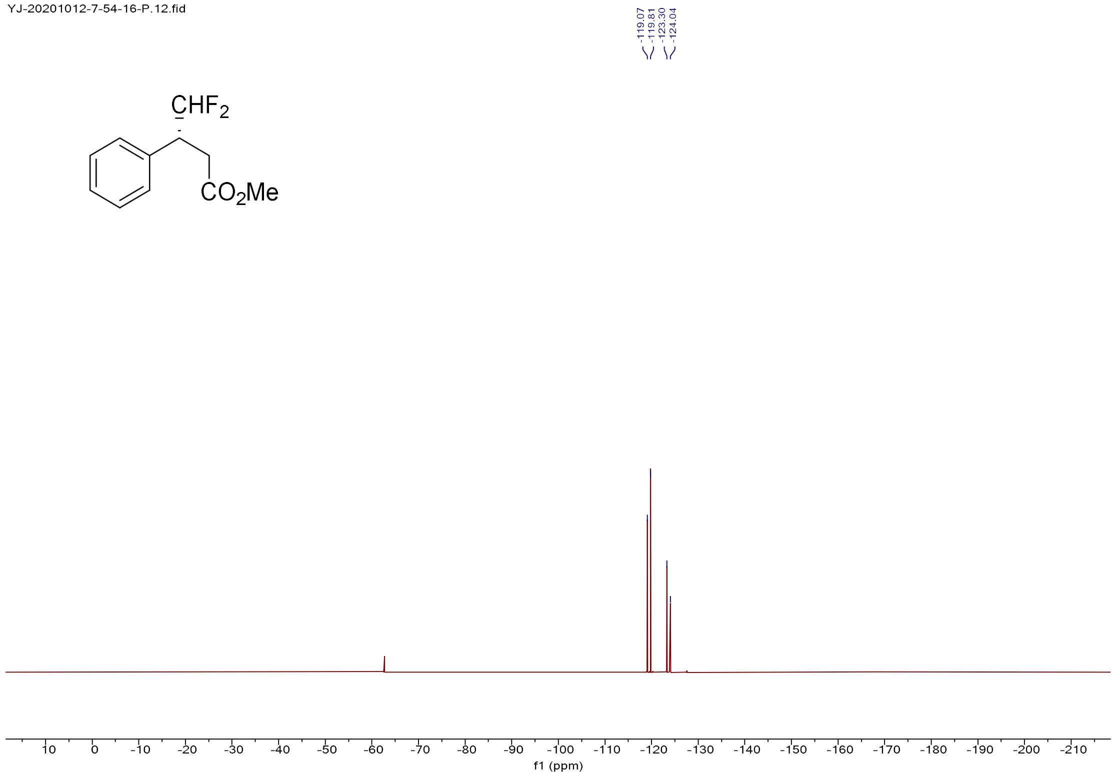
YJ-20201012-7-54-16-P.12.fid
-119.07
-119.81
-123.30
-124.04
CHF2
CO2Me
f1 (ppm)

YJ-20201012-7-54-8-P.10.fid

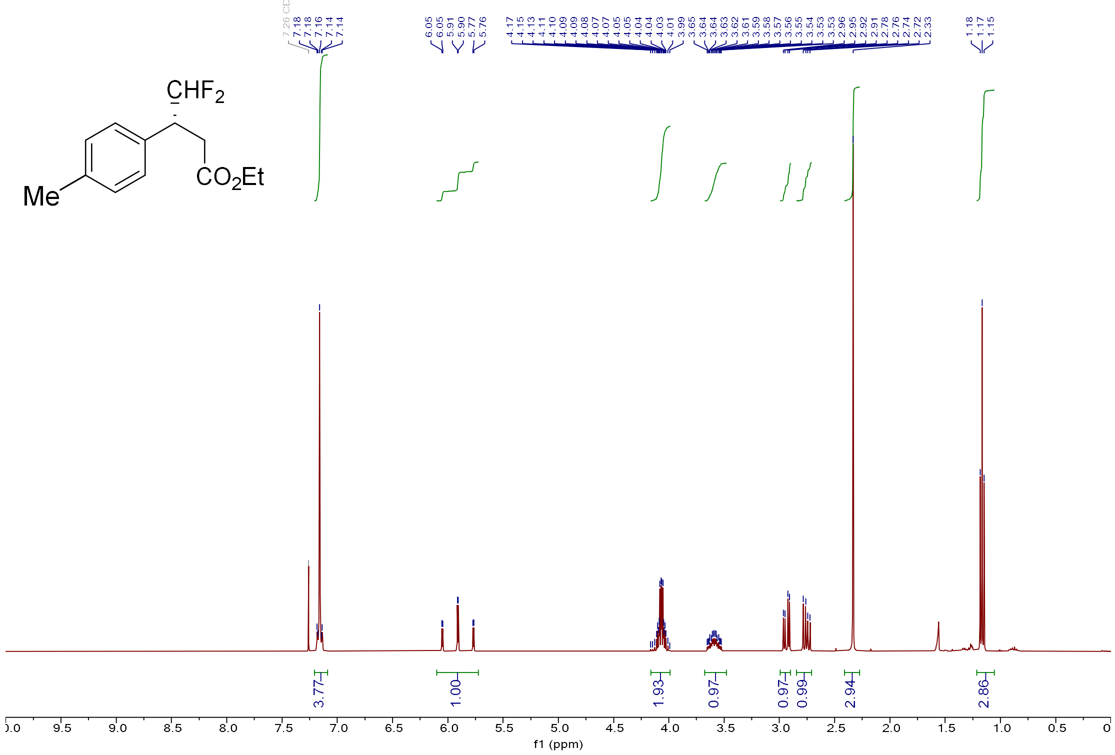

YJ-20201012-7-54-8-P.12.fid

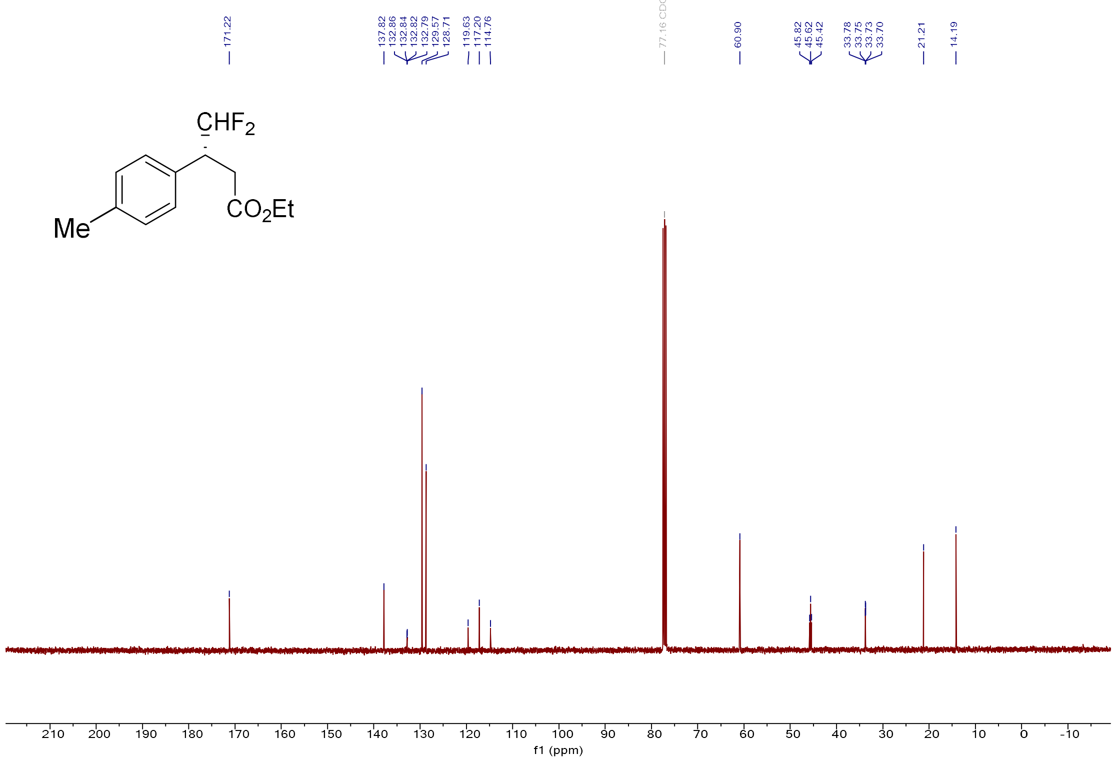

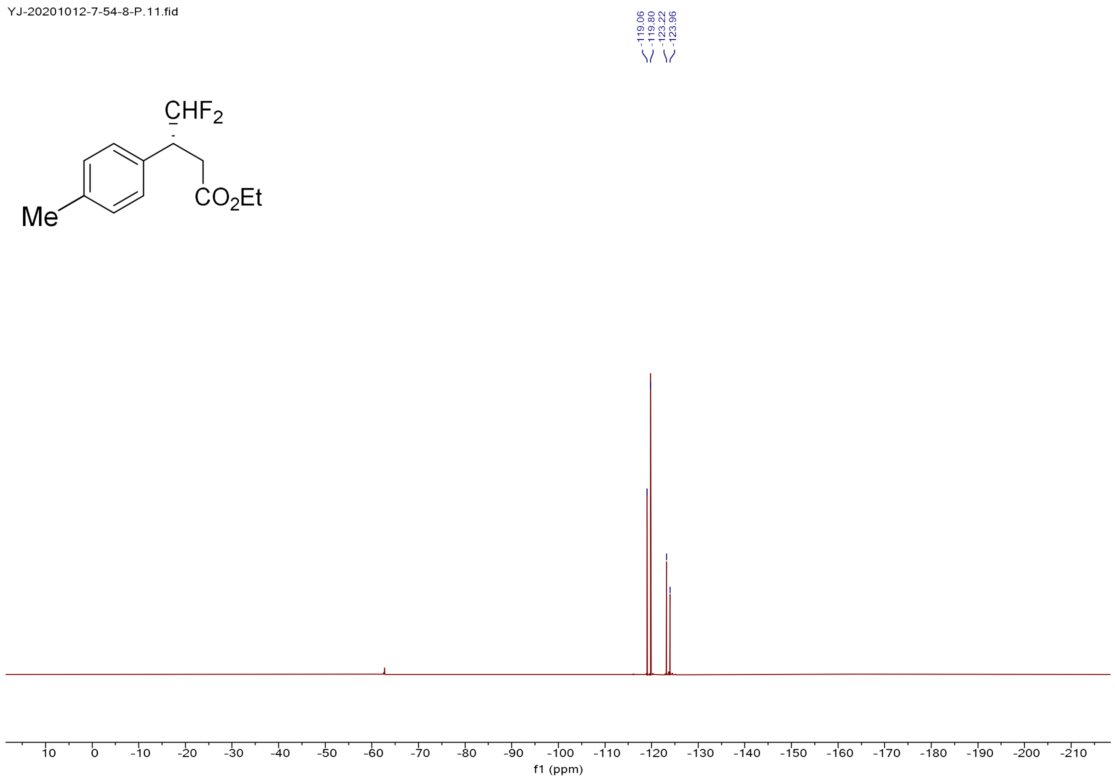
YJ-20201012-7-54-8-P.11.fid
-119.06
-119.80
-123.22
-123.96
CHF2
CO2Et
Me
f1 (ppm)

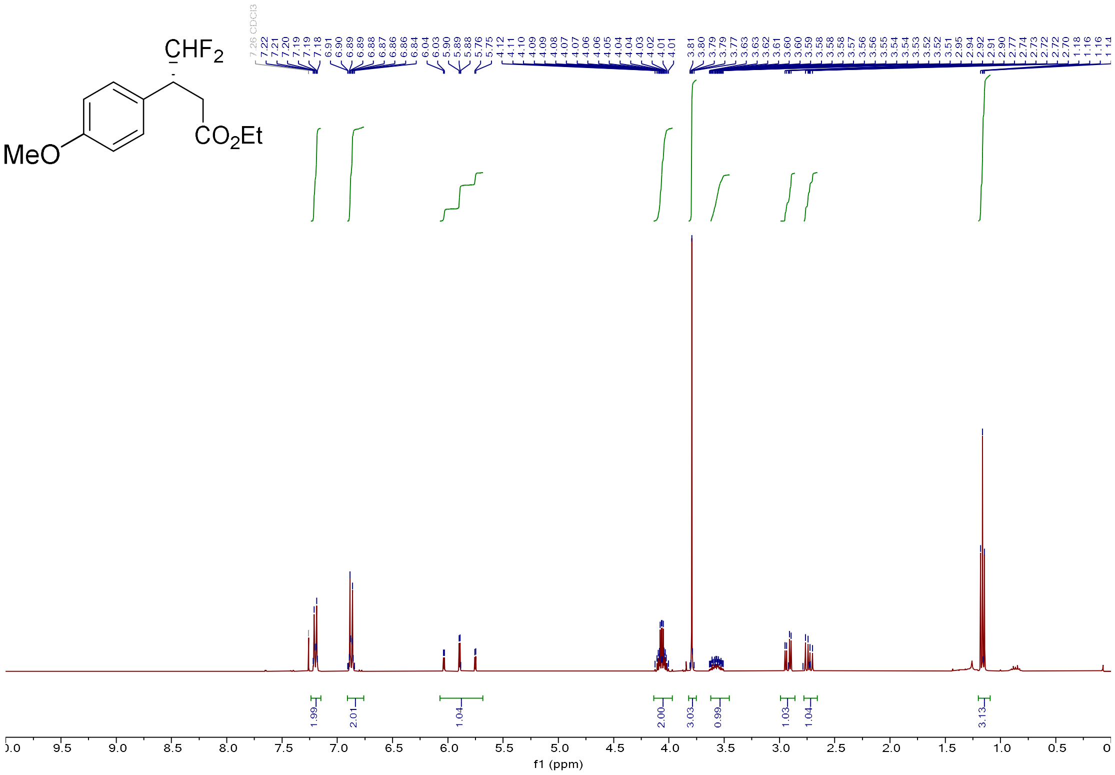
CHF2
CO2Et
MeO
7.26 CDCl3
1.99
2.01
1.04
2.00
3.03
0.99
1.03
1.04
3.13
f1 (ppm)

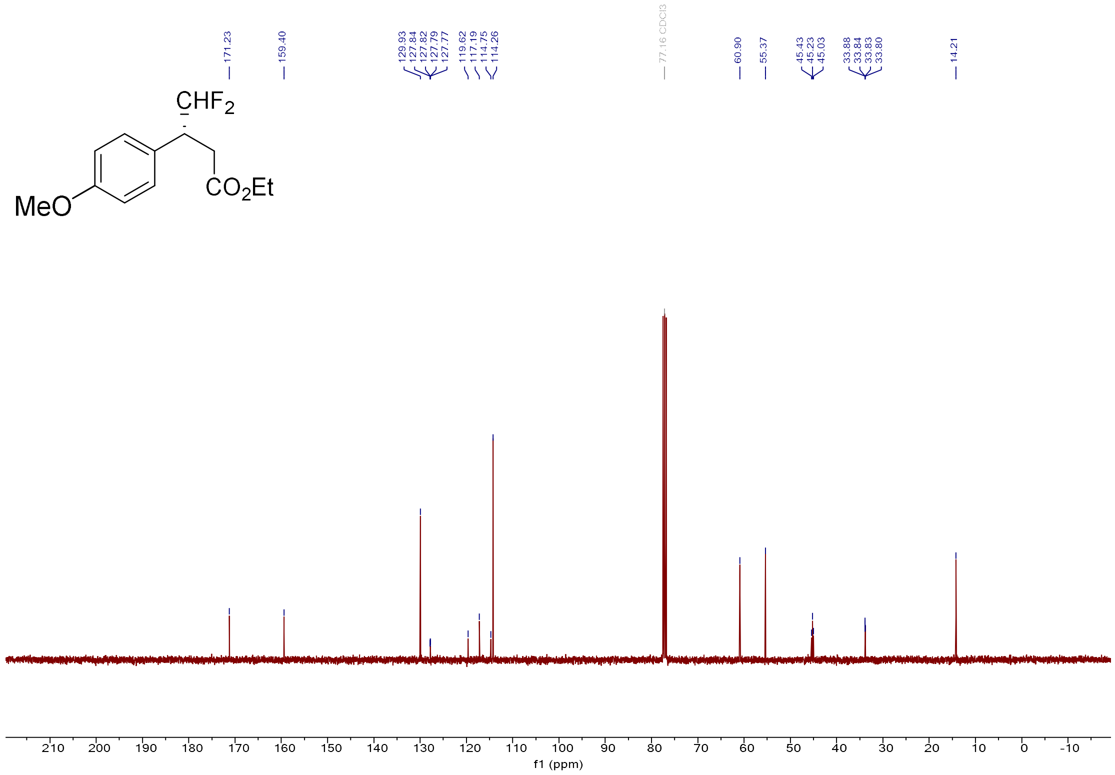

171.23
159.40
129.93
127.84
127.82
127.79
127.77
119.62
117.19
114.75
114.26
77.16 CDCl3
60.90
55.37
45.43
45.23
45.03
33.88
33.84
33.83
33.80
14.21
CHF2
CO2Et
MeO
f1 (ppm)

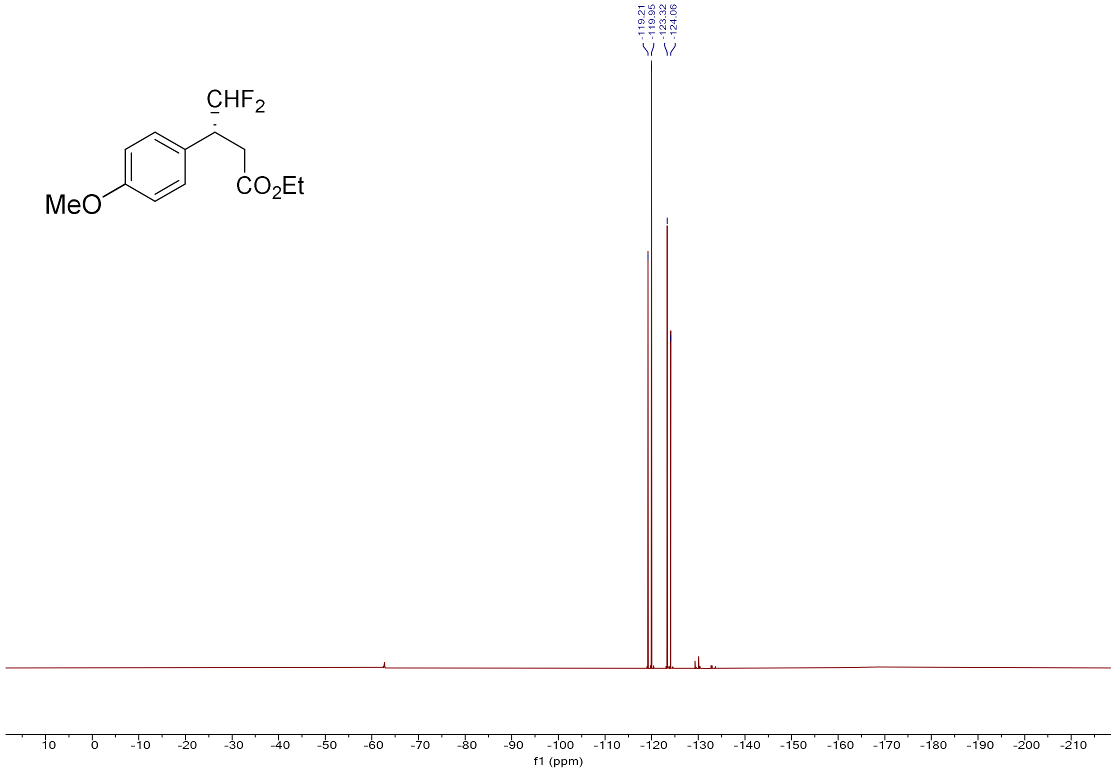

-119.21
-119.95
-123.32
-124.06
CHF2
CO2Et
MeO
f1 (ppm)

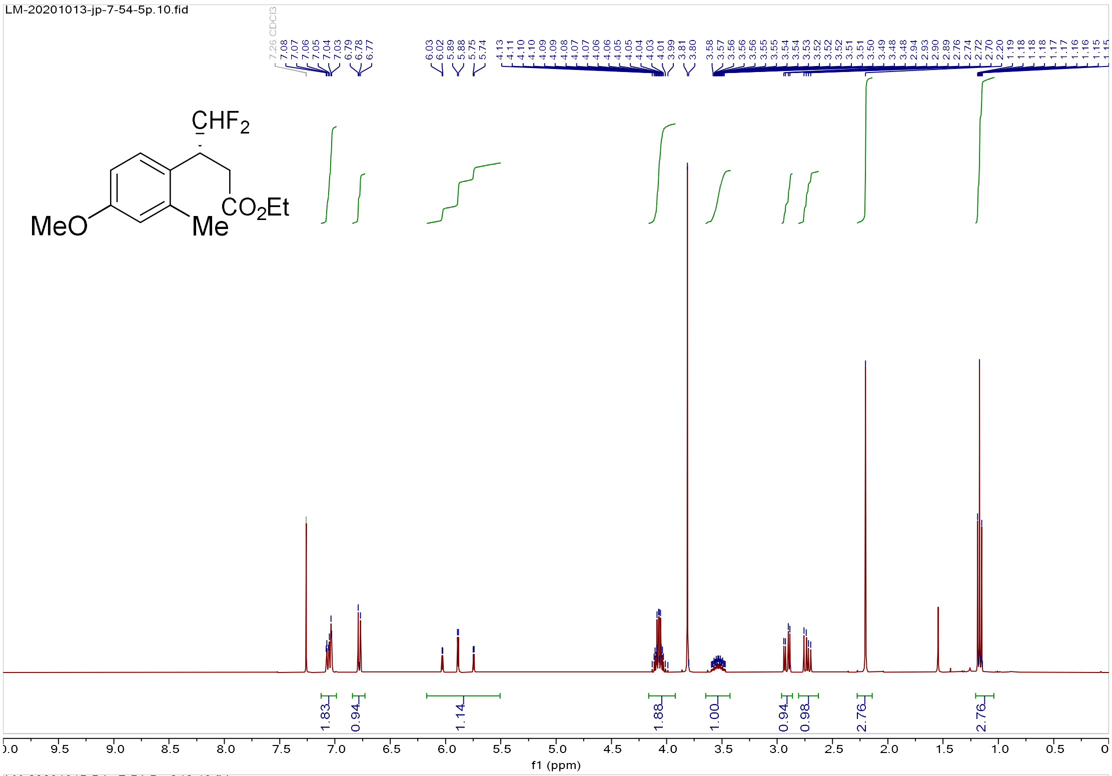
LM-20201013-jp-7-54-5p.10.fid
CHF2
MeO
Me
CO2Et
f1 (ppm)

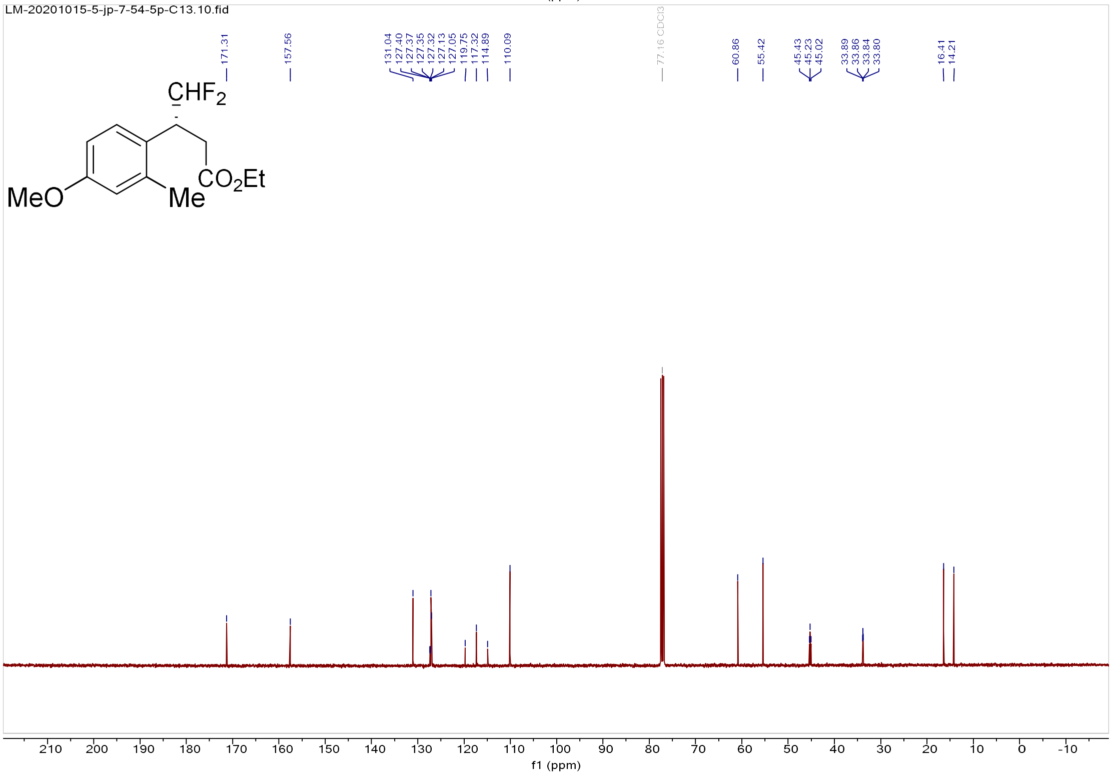
LM-20201015-5-jp-7-54-5p-C13.10.fid
171.31
157.56
131.04
127.40
127.37
127.35
127.32
127.13
127.05
119.75
117.32
114.89
110.09
77.16 CDCl3
60.86
55.42
45.43
45.23
45.02
33.89
33.86
33.84
33.80
16.41
14.21
CHF2
MeO
Me
CO2Et
f1 (ppm)

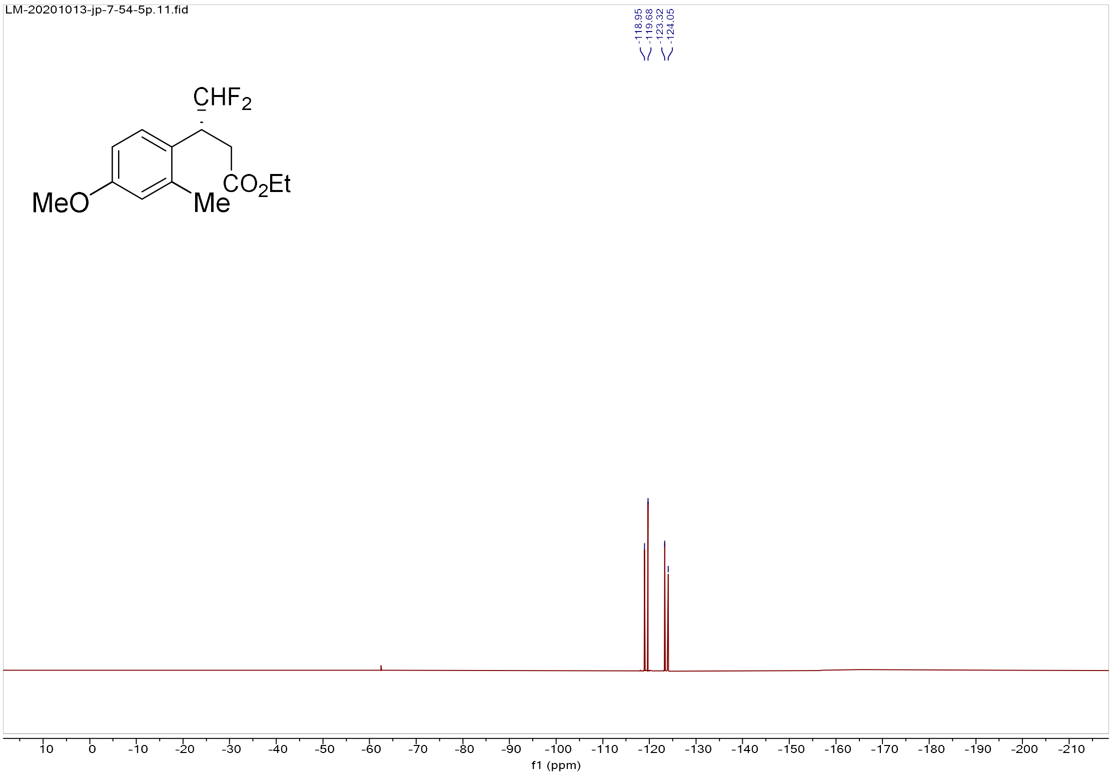
LM-20201013-jp-7-54-5p.11.fid
-118.95
-119.68
-123.32
-124.05
CHF2
MeO
Me
CO2Et
10 0 -10 -20 -30 -40 -50 -60 -70 -80 -90 -100 -110 -120 -130 -140 -150 -160 -170 -180 -190 -200 -210
f1 (ppm)

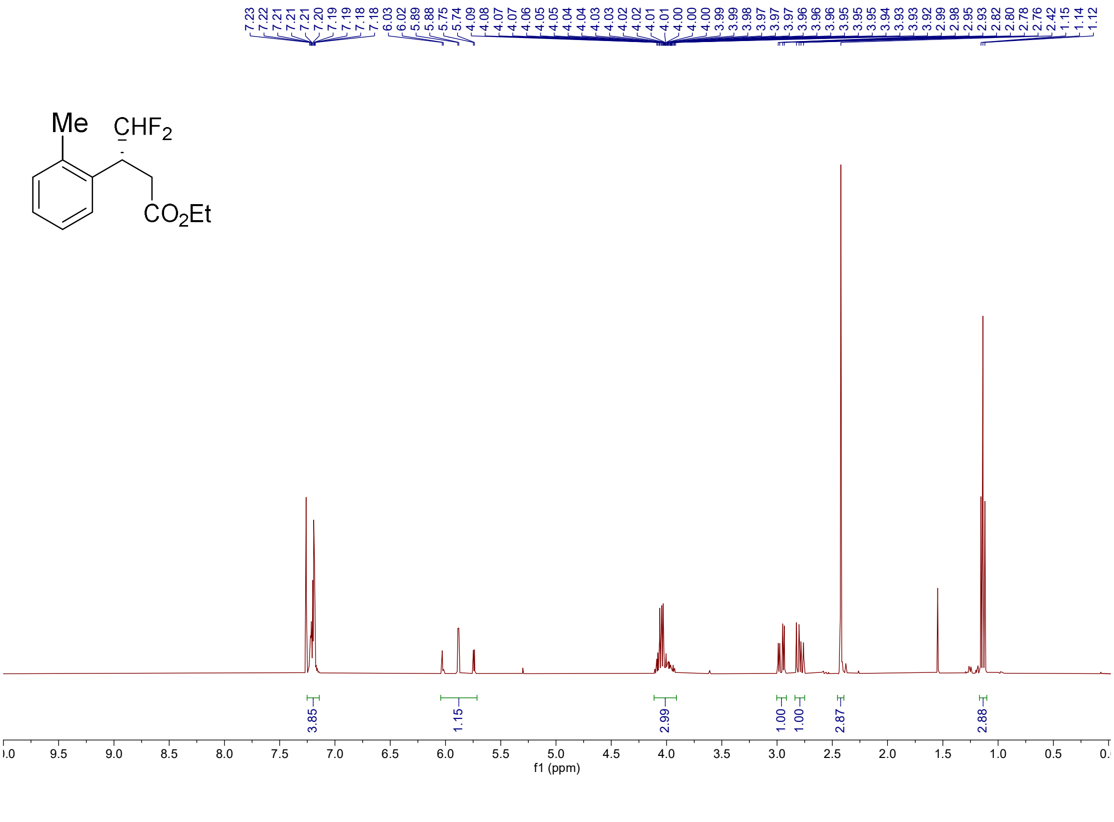
7.23 7.22 7.21 7.21 7.21 7.20 7.19 7.19 7.18 7.18 6.03 6.02 5.89 5.88 5.75 5.74 4.09 4.08 4.07 4.06 4.05 4.04 4.04 4.03 4.02 4.02 4.01 4.01 4.00 4.00 4.00 3.99 3.99 3.98 3.97 3.97 3.96 3.96 3.95 3.95 3.94 3.93 3.92 2.99 2.98 2.95 2.93 2.82 2.80 2.78 2.76 2.42 1.15 1.14 1.12
Me
CHF2
CO2Et
3.85
1.15
2.99
1.00
1.00
2.87
2.88
9.5 9.0 8.5 8.0 7.5 7.0 6.5 6.0 5.5 5.0 4.5 4.0 3.5 3.0 2.5 2.0 1.5 1.0 0.5
f1 (ppm)

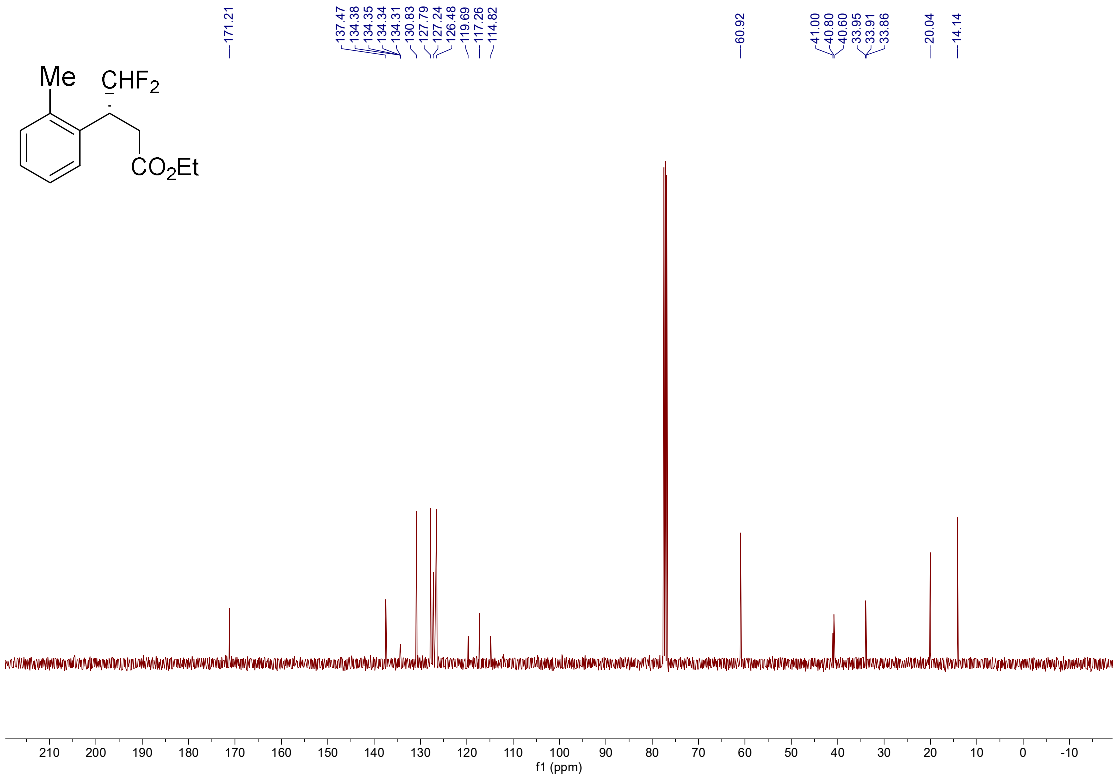

171.21
137.47
134.38
134.35
134.34
134.31
130.83
127.79
127.24
126.48
119.69
117.26
114.82
60.92
41.00
40.80
40.60
33.95
33.91
33.86
20.04
14.14
Me
CHF2
CO2Et
210 200 190 180 170 160 150 140 130 120 110 100 90 80 70 60 50 40 30 20 10 0 -10
f1 (ppm)

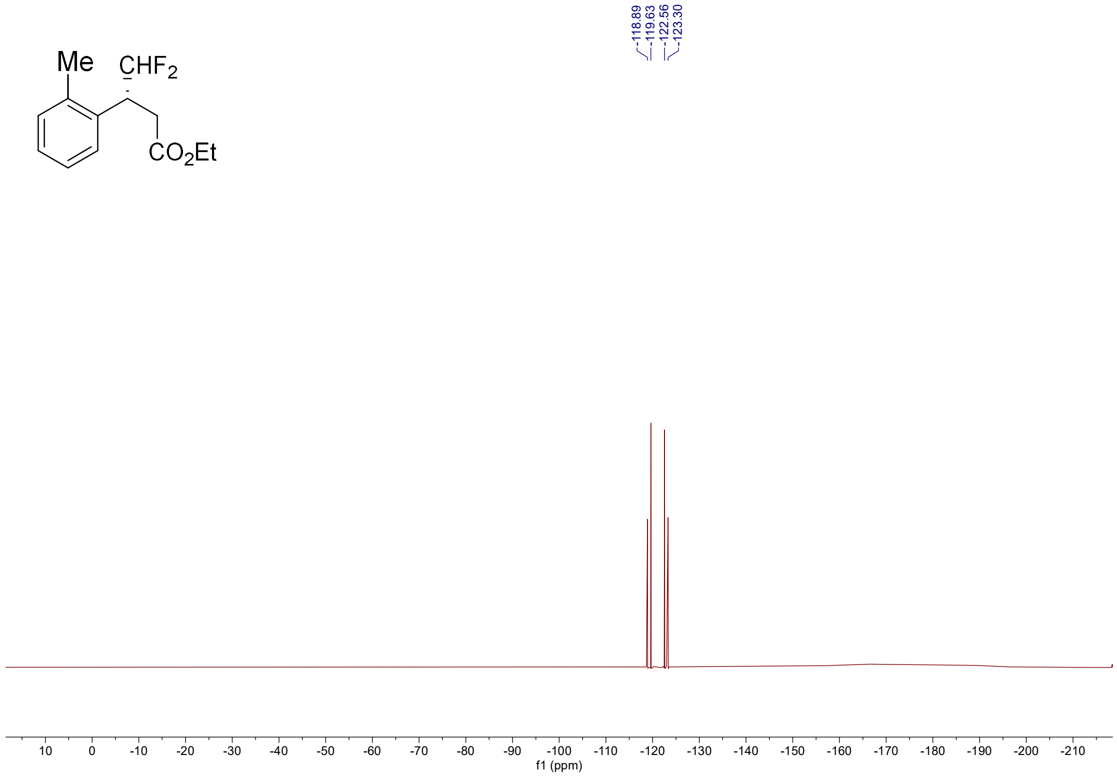

-118.89
-119.63
-122.56
-123.30
Me
CHF2
CO2Et
10 0 -10 -20 -30 -40 -50 -60 -70 -80 -90 -100 -110 -120 -130 -140 -150 -160 -170 -180 -190 -200 -210
f1 (ppm)

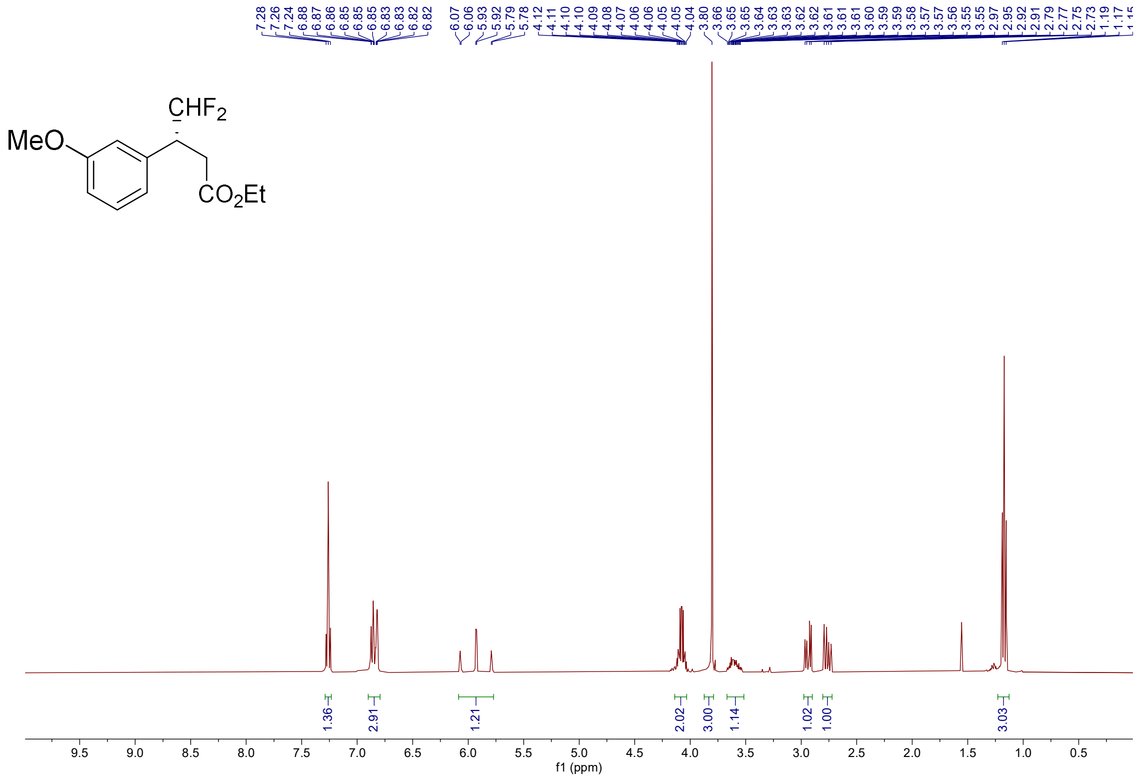

CHF2
MeO
CO2Et
7.28
7.26
7.24
6.88
6.87
6.86
6.85
6.85
6.83
6.83
6.82
6.82
6.07
6.06
5.93
5.92
5.79
5.78
1.36
2.91
1.21
2.02
3.00
1.14
1.02
1.00
3.03
f1 (ppm)

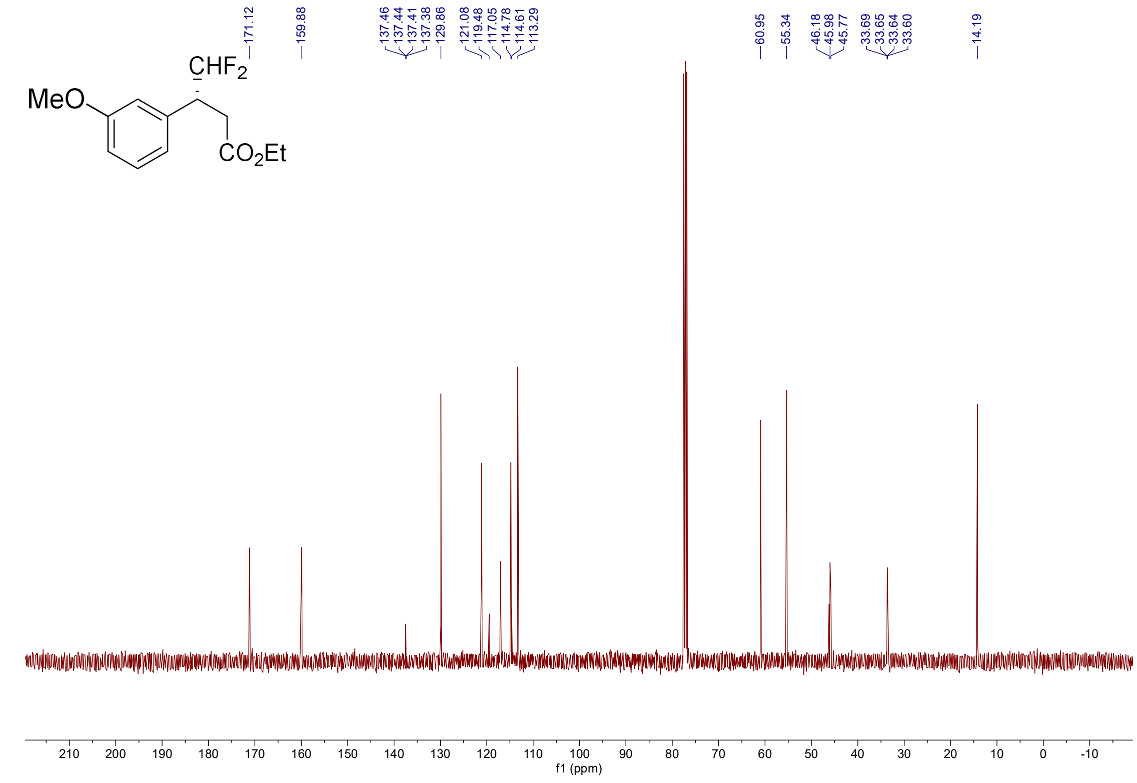

171.12
159.88
137.46
137.44
137.41
137.38
129.86
121.08
119.48
117.05
114.78
114.61
113.29
60.95
55.34
46.18
45.98
45.77
33.69
33.65
33.64
33.60
14.19
CHF2
MeO
CO2Et
f1 (ppm)

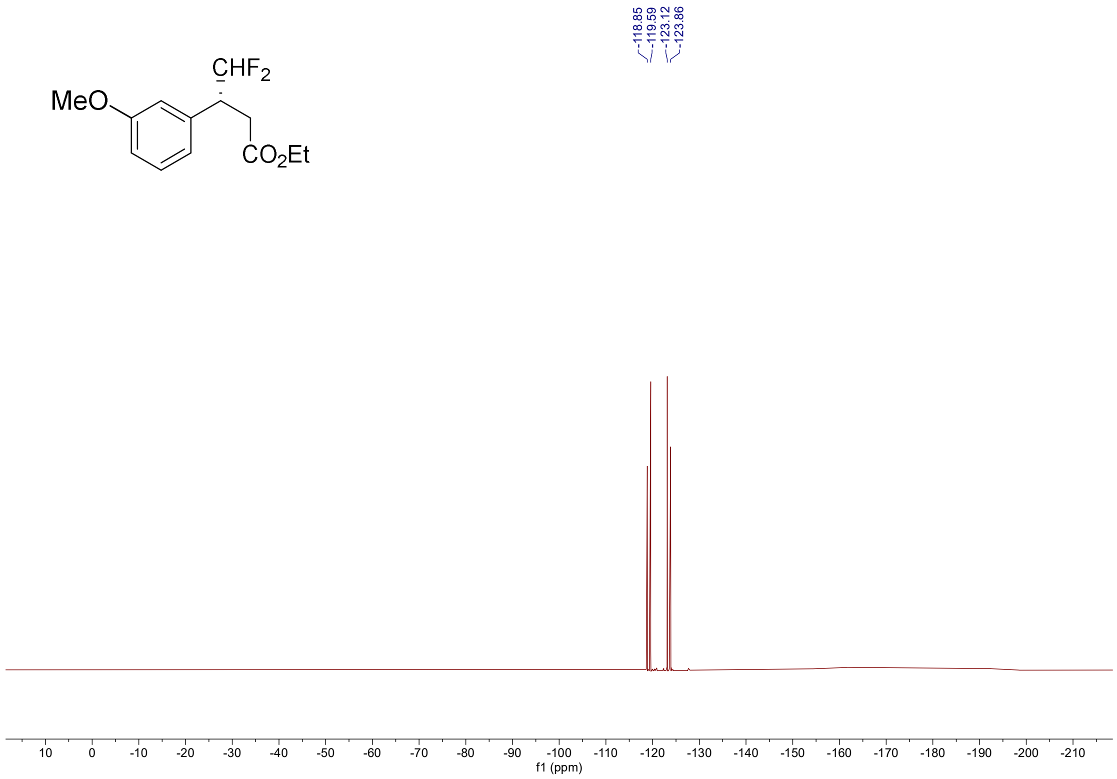

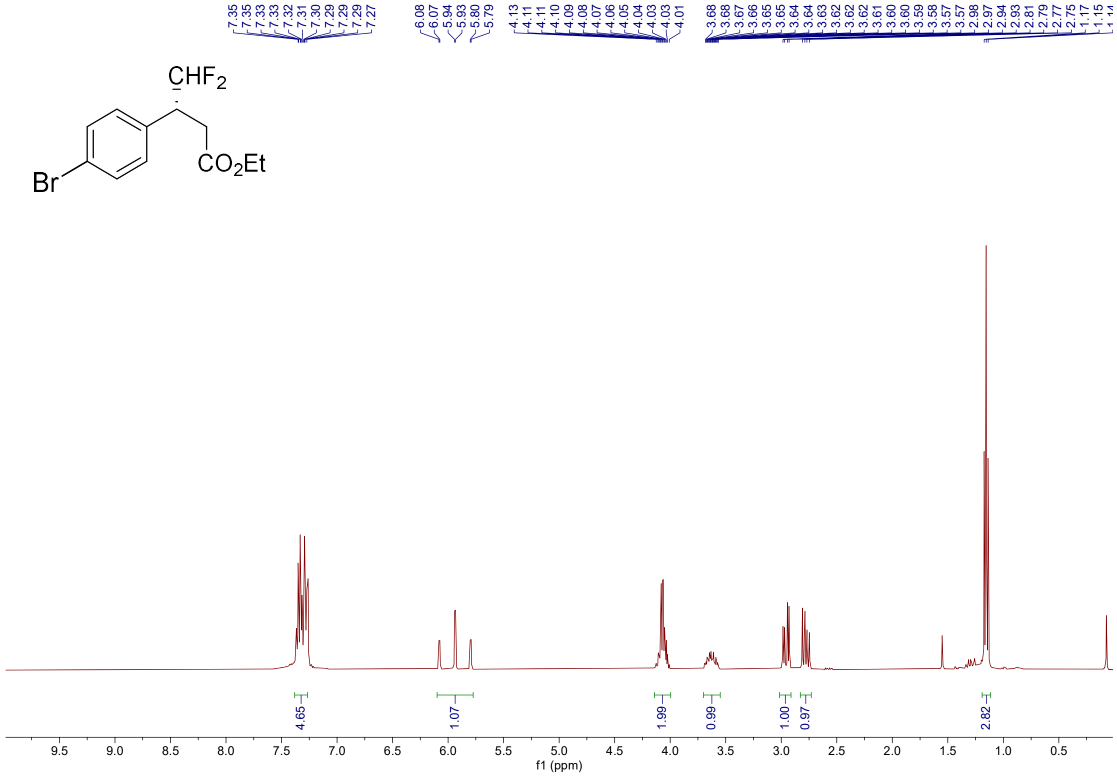

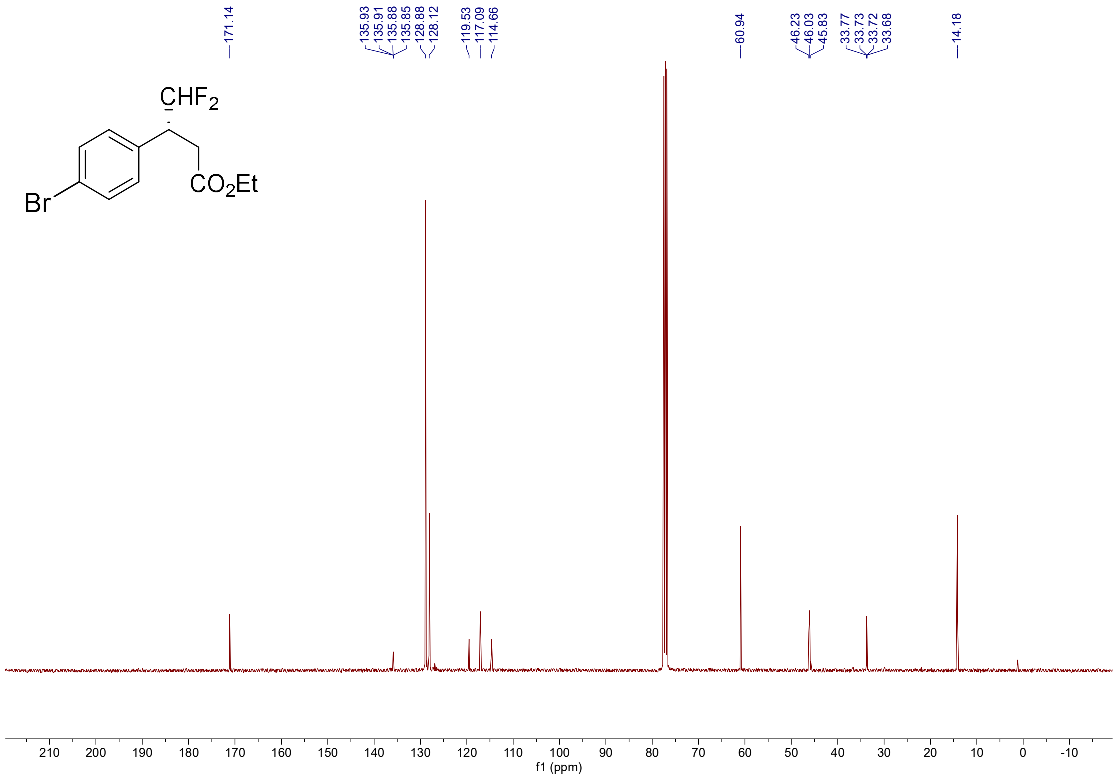
171.14
135.93
135.91
135.88
135.85
128.88
128.12
119.53
117.09
114.66
60.94
46.23
46.03
45.83
33.77
33.73
33.72
33.68
14.18
CHF2
CO2Et
Br
210 200 190 180 170 160 150 140 130 120 110 100 90 80 70 60 50 40 30 20 10 0 -10
f1 (ppm)

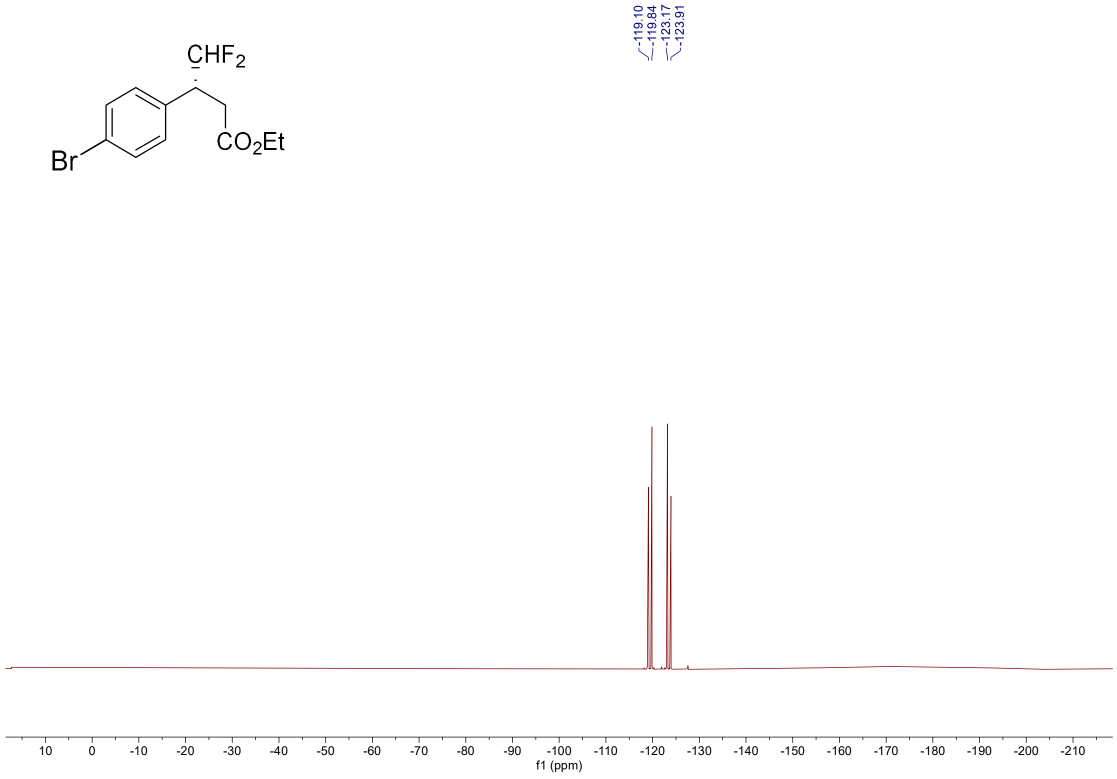
-119.10
-119.84
-123.17
-123.91
CHF2
CO2Et
Br
10 0 -10 -20 -30 -40 -50 -60 -70 -80 -90 -100 -110 -120 -130 -140 -150 -160 -170 -180 -190 -200 -210
f1 (ppm)

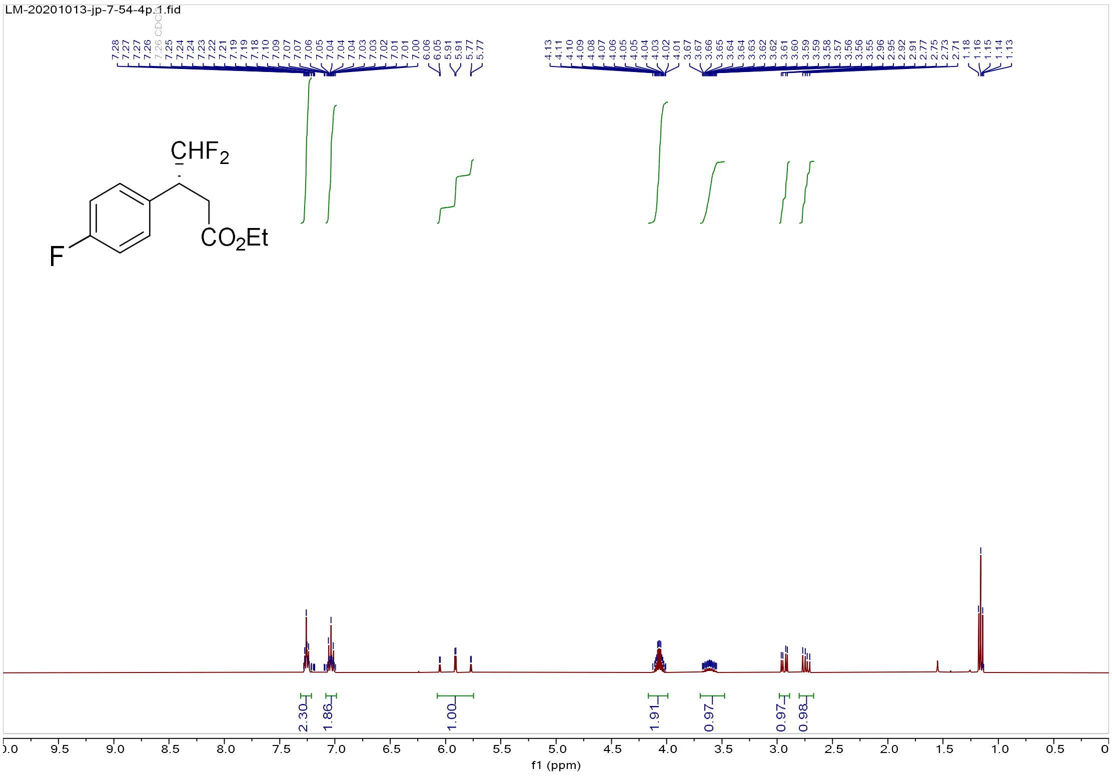
LM-20201013-jp-7-54-4p.1.fid
CHF2
F
CO2Et
f1 (ppm)

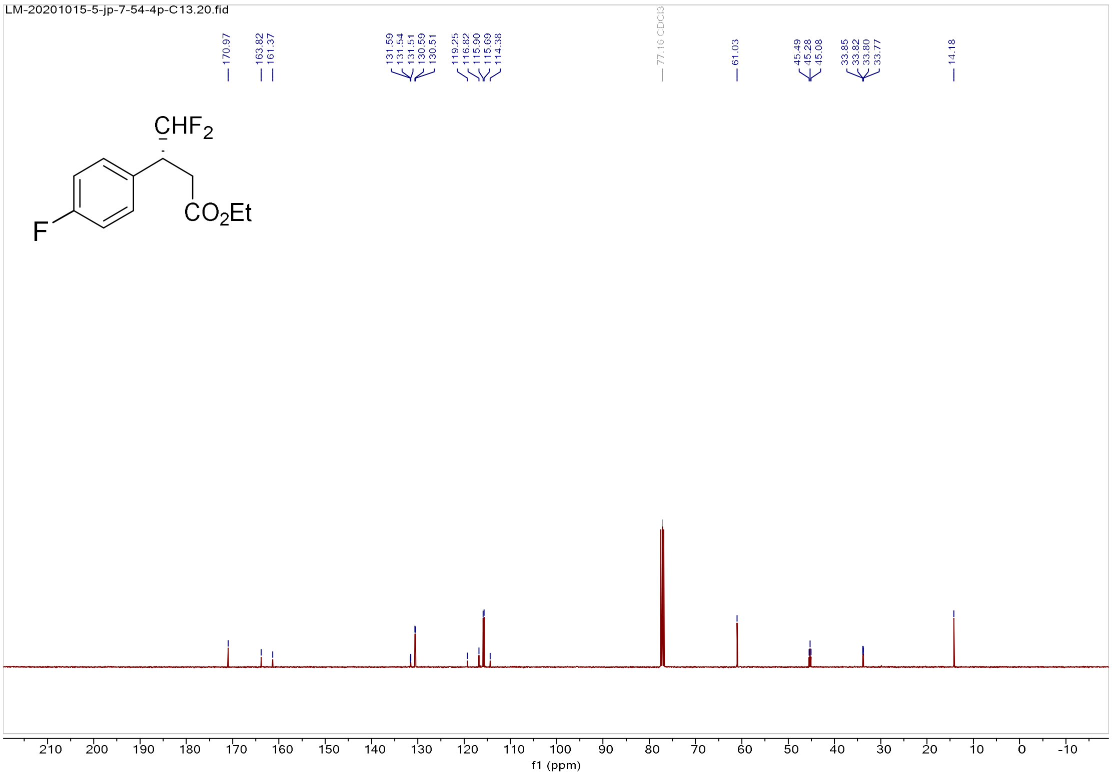
LM-20201015-5-jp-7-54-4p-C13.20.fid
170.97
163.82
161.37
131.59
131.54
131.51
130.59
130.51
119.25
116.82
115.90
115.69
114.38
77.16 CDCl3
61.03
45.49
45.28
45.08
33.85
33.82
33.80
33.77
14.18
CHF2
F
CO2Et
f1 (ppm)

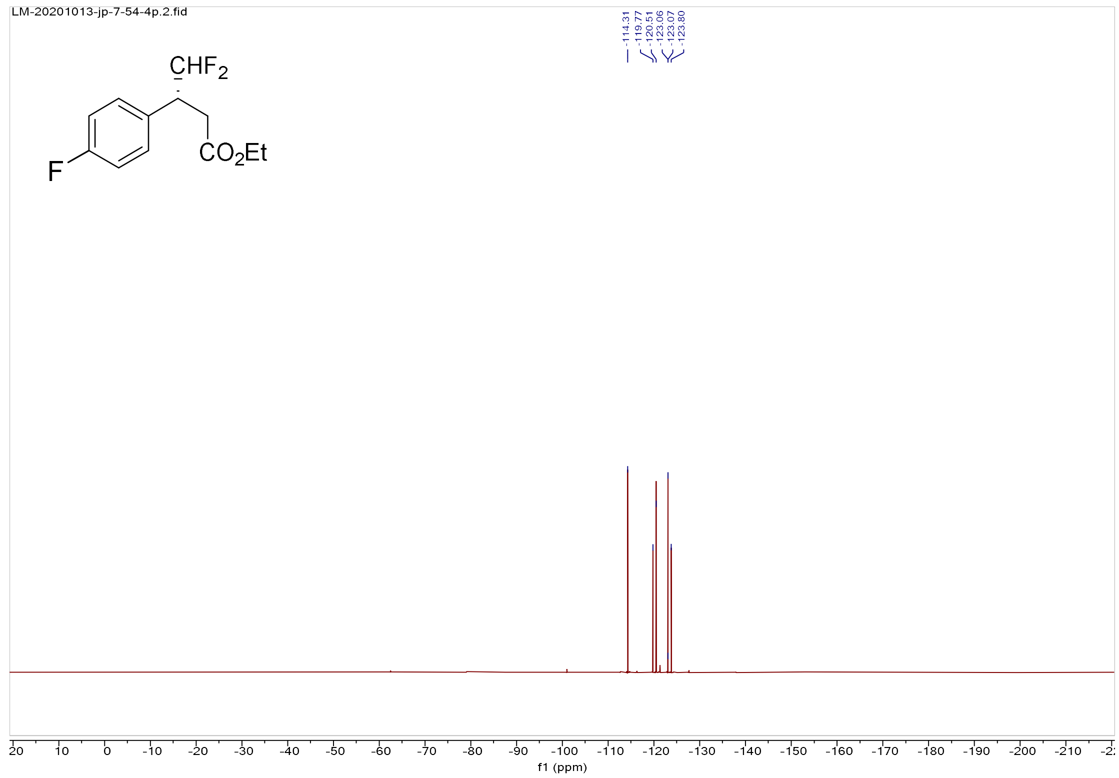
LM-20201013-jp-7-54-4p.2.fid
-114.31
-119.77
-120.51
-123.06
-123.07
-123.80
CHF2
F
CO2Et
f1 (ppm)

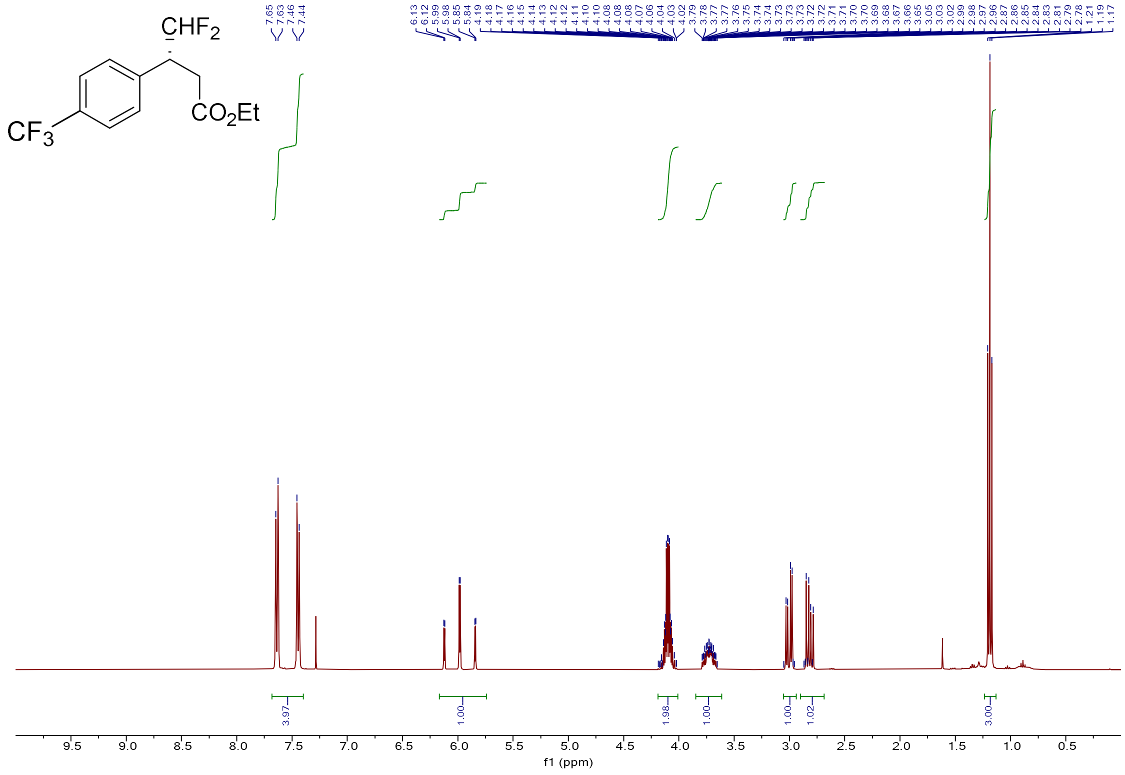
CHF2
CF3
CO2Et
3.97
1.00
1.98
1.00
1.00
1.02
3.00
f1 (ppm)

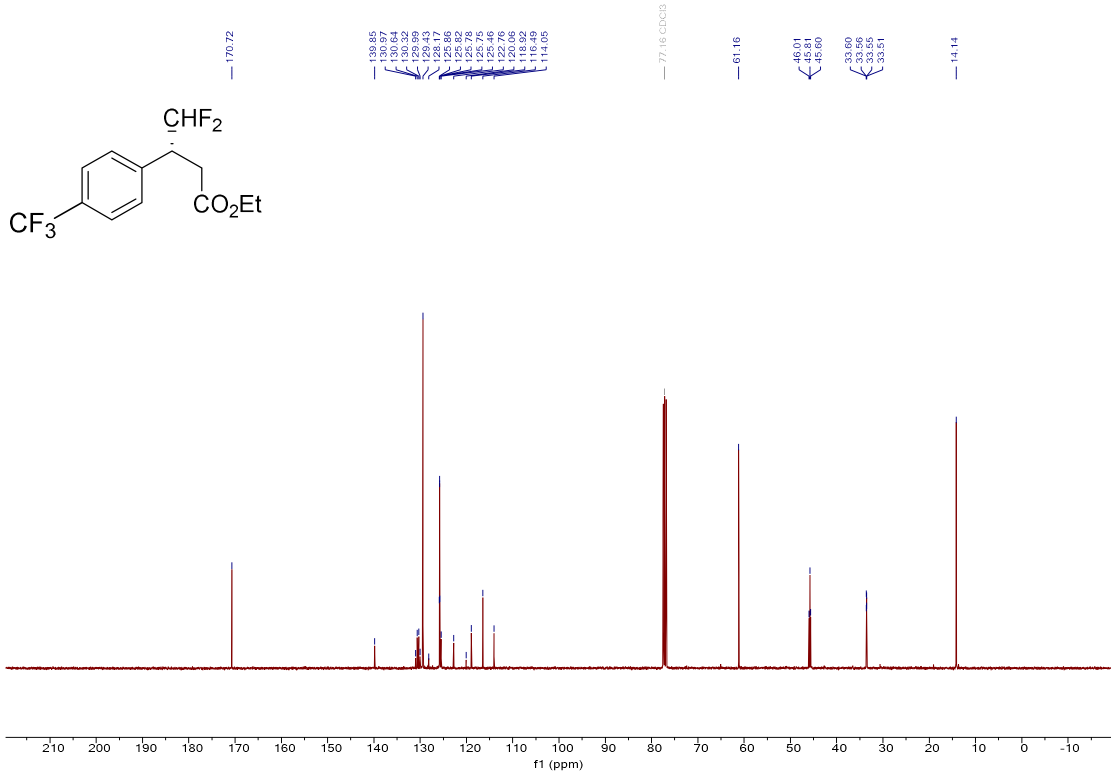
170.72
139.85
130.97
130.64
130.32
129.99
129.43
128.17
125.86
125.82
125.78
125.75
125.46
122.76
120.06
118.92
116.49
114.05
77.16 CDCl3
61.16
46.01
45.81
45.60
33.60
33.56
33.55
33.51
14.14
CHF2
CF3
CO2Et
f1 (ppm)

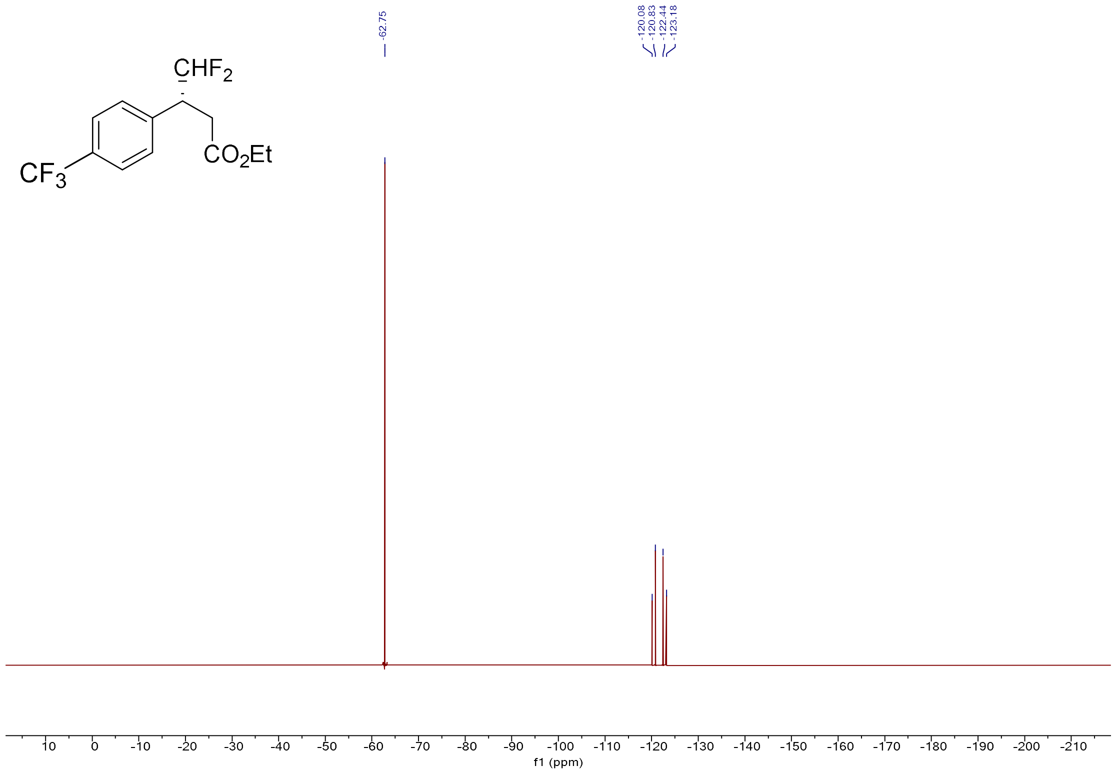
-62.75
-120.08
-120.83
-122.44
-123.18
CHF2
CF3
CO2Et
f1 (ppm)

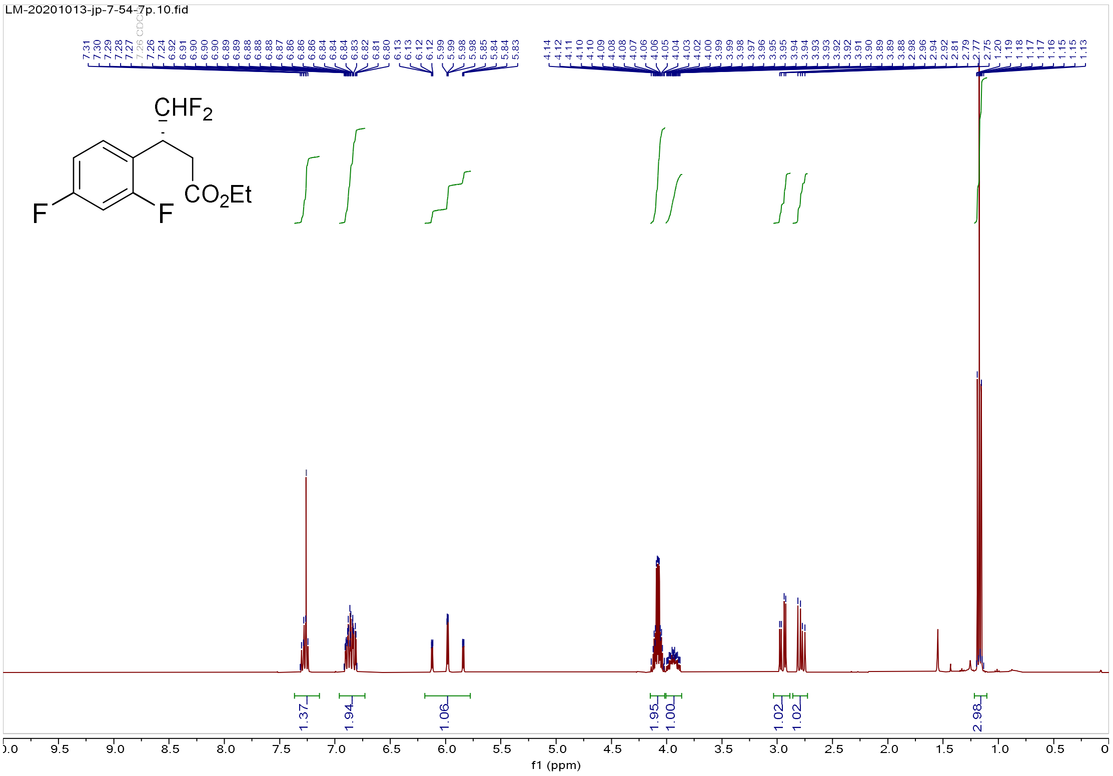

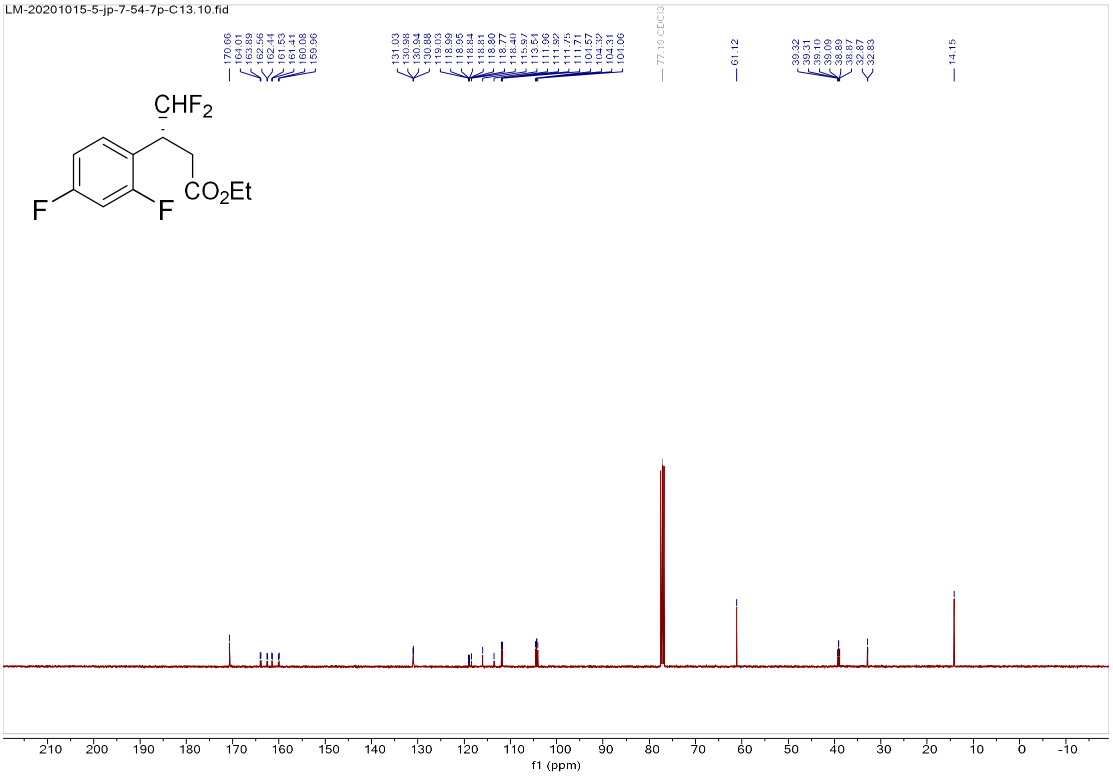

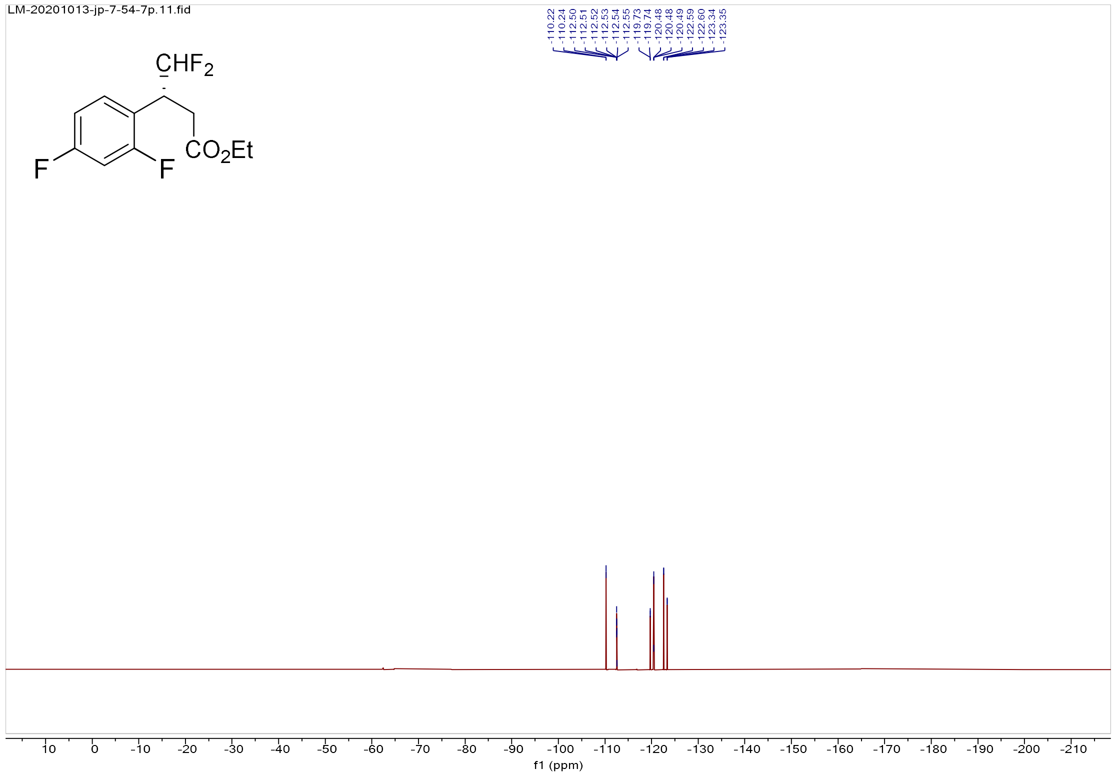
LM-20201013-jp-7-54-7p.11.fid
CHF2
CO2Et
F
F
f1 (ppm)

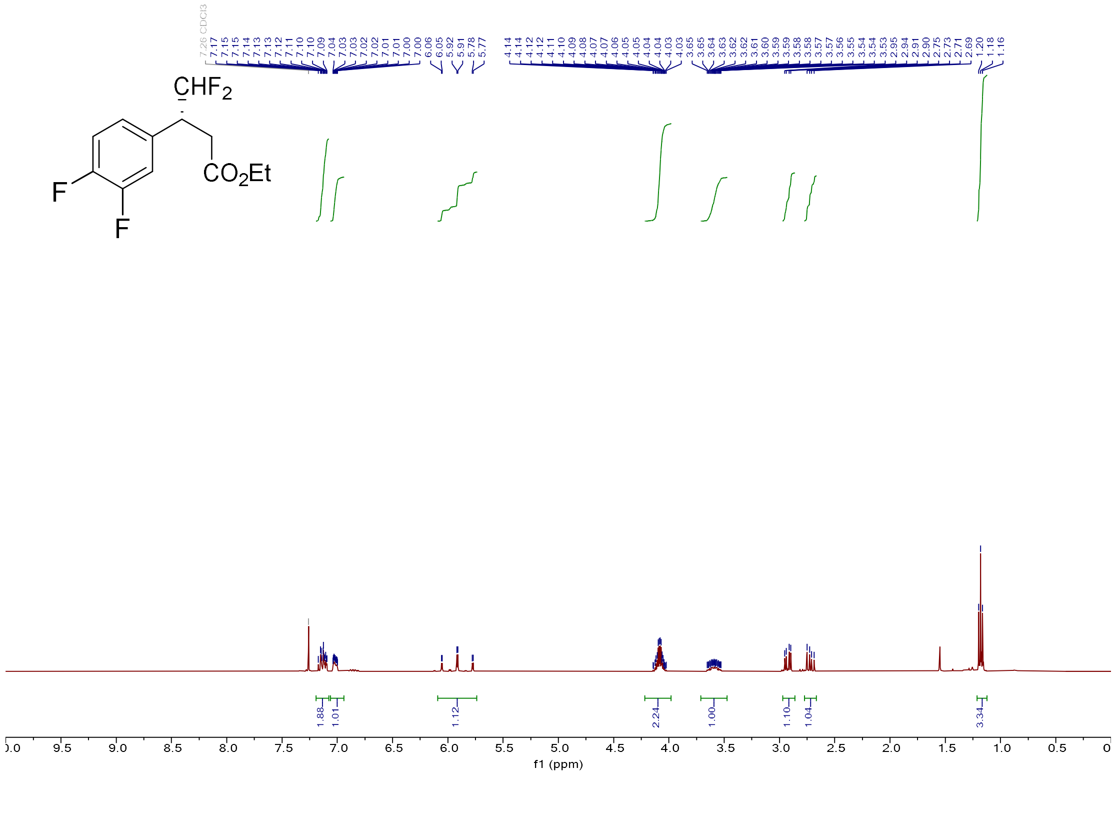
CHF2
CO2Et
F
F
f1 (ppm)

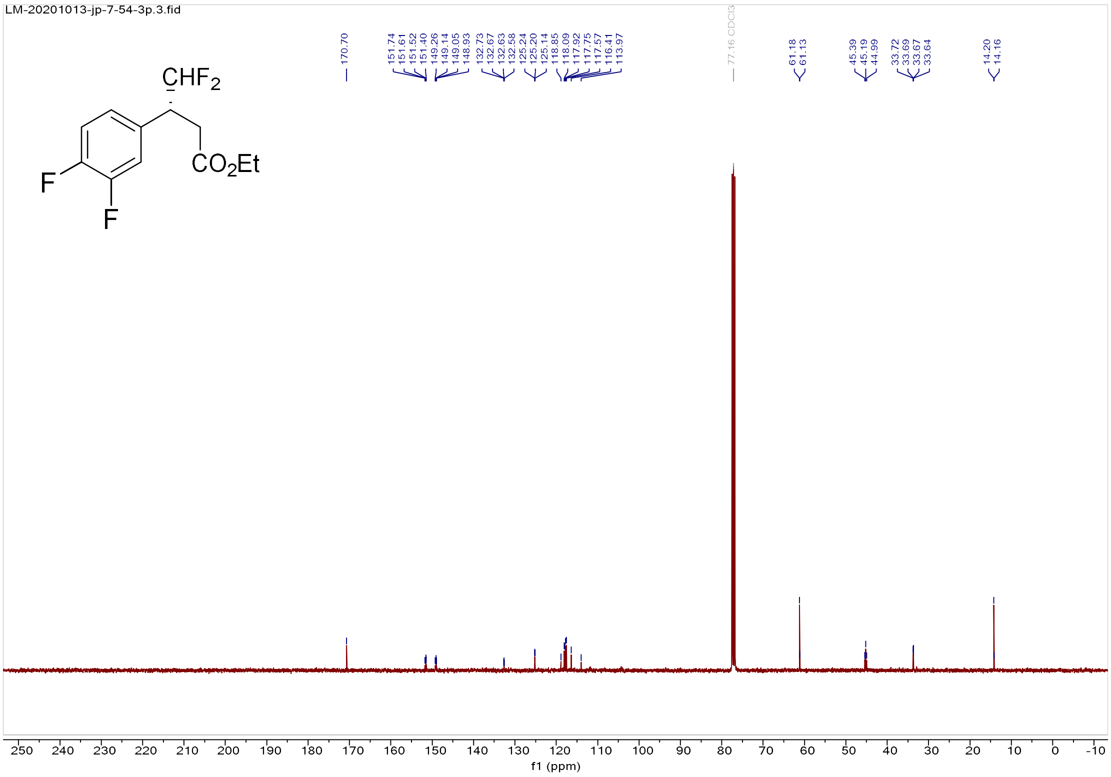
LM-20201013-jp-7-54-3p.3.fid
CHF2
CO2Et
F
F
f1 (ppm)

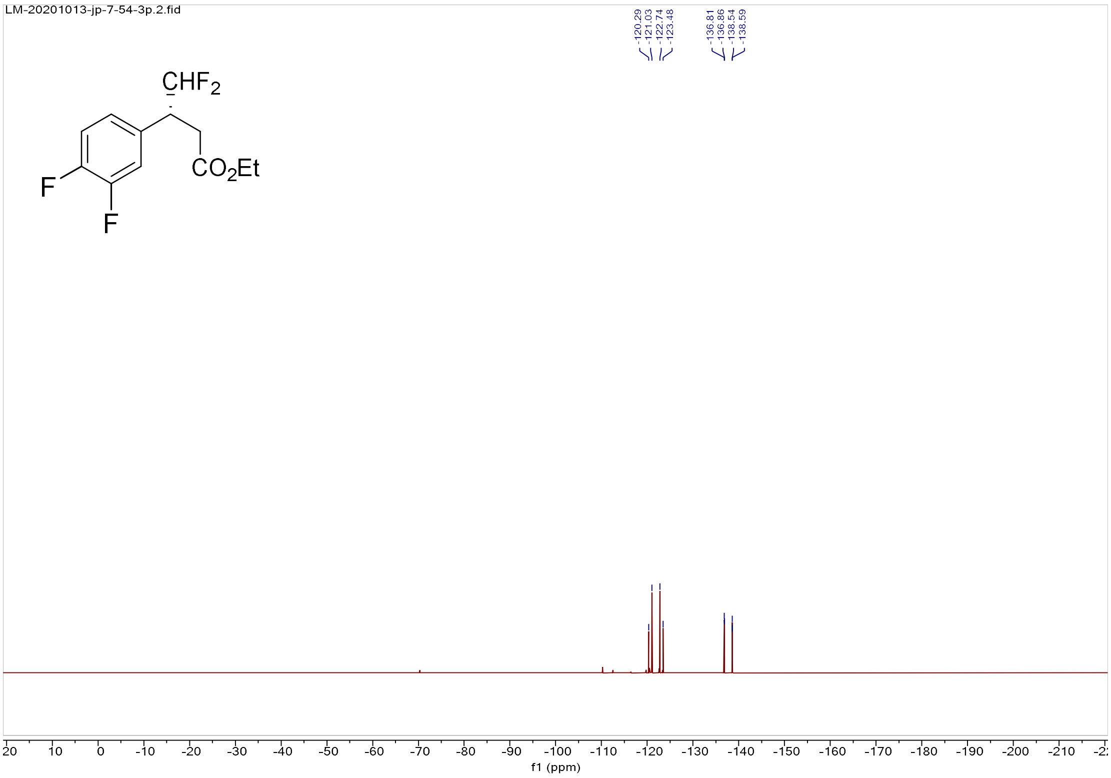
LM-20201013-jp-7-54-3p.2.fid
CHF2
CO2Et
F
F
f1 (ppm)

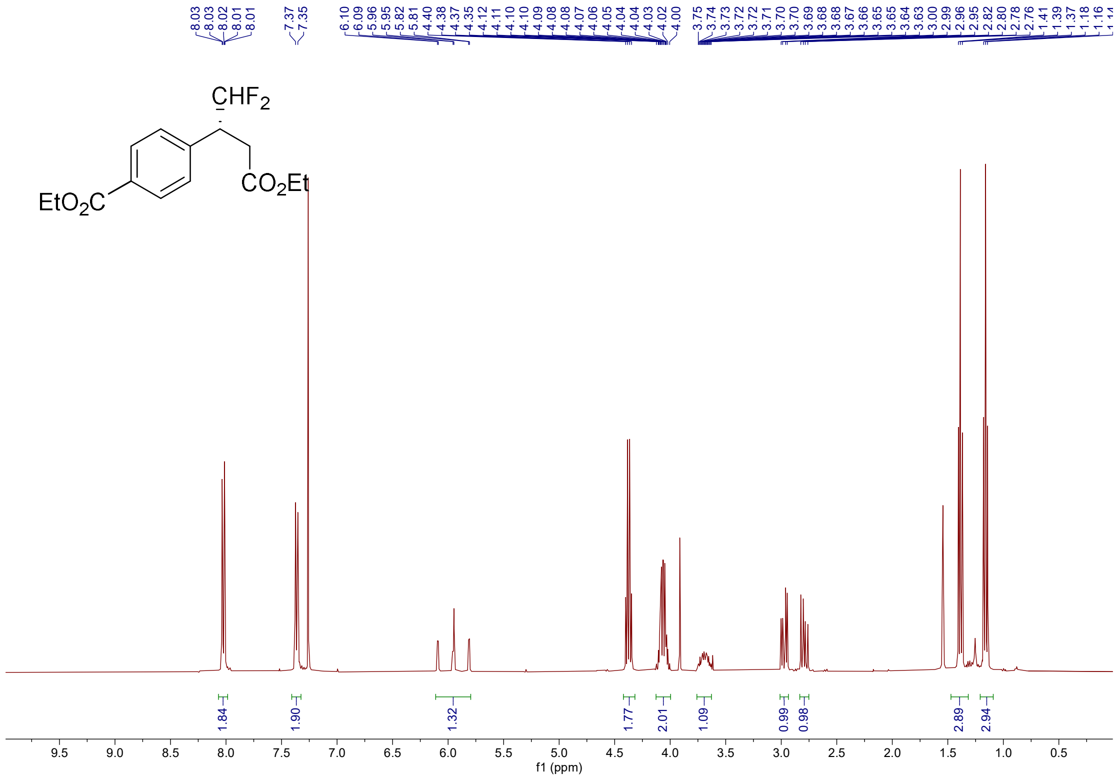

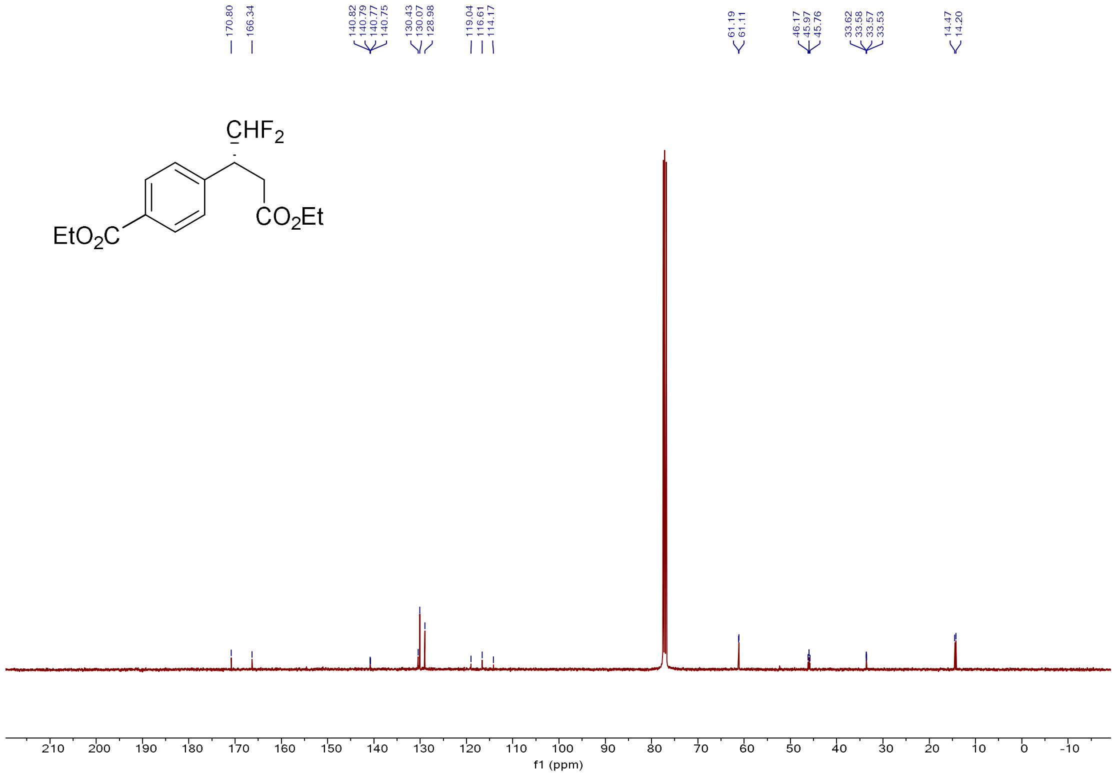

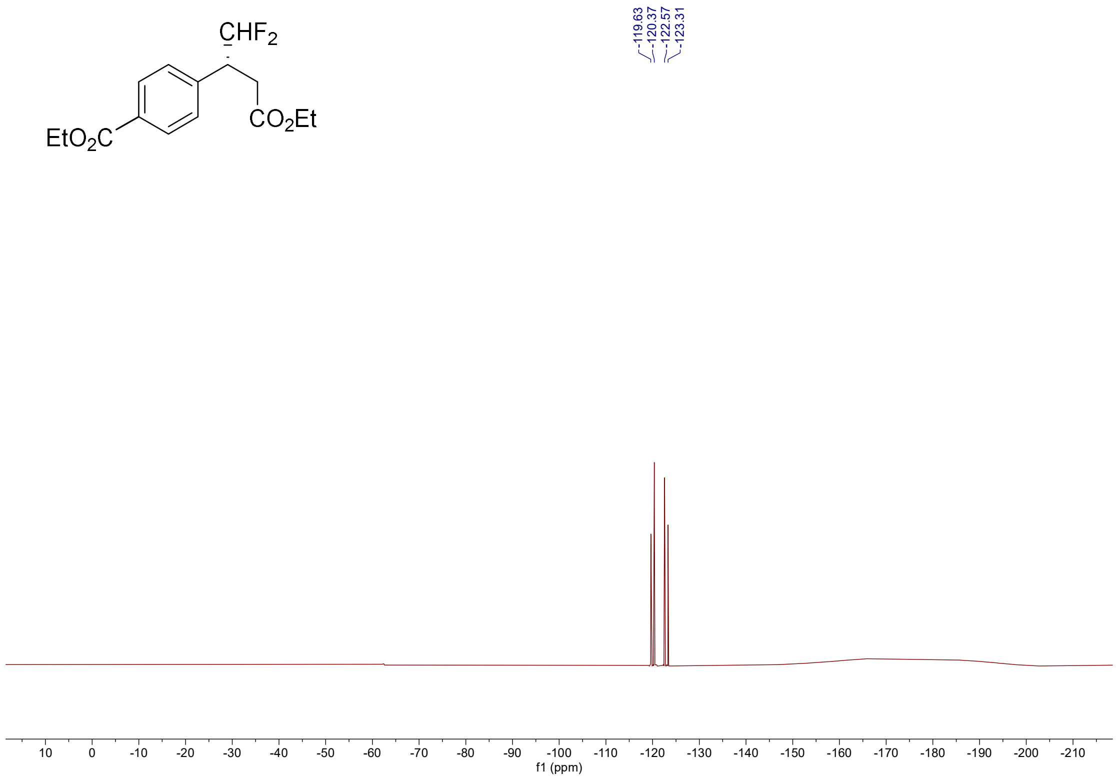

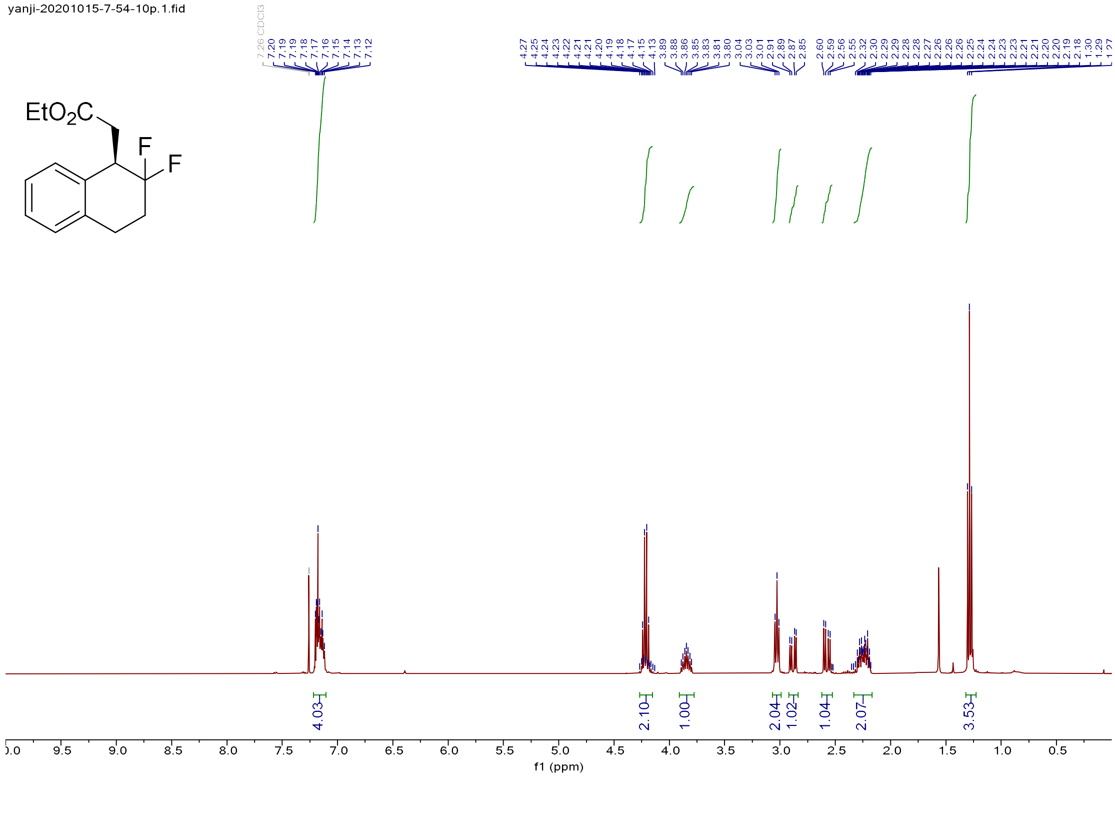

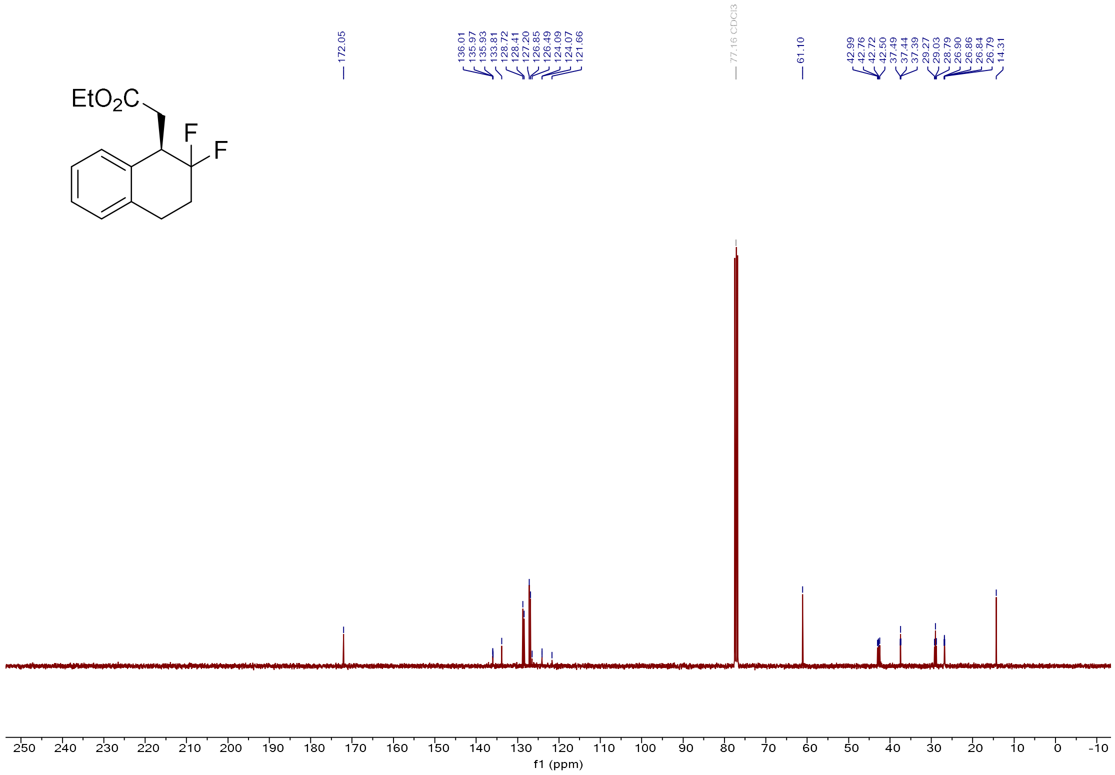
EtO2C
F
F
172.05
136.01
135.97
135.93
133.81
128.72
128.41
127.20
126.85
126.49
124.09
124.07
121.66
77.16 CDCl3
61.10
42.99
42.76
42.72
42.50
37.49
37.44
37.39
29.27
29.03
28.79
26.90
26.86
26.84
26.79
14.31
f1 (ppm)

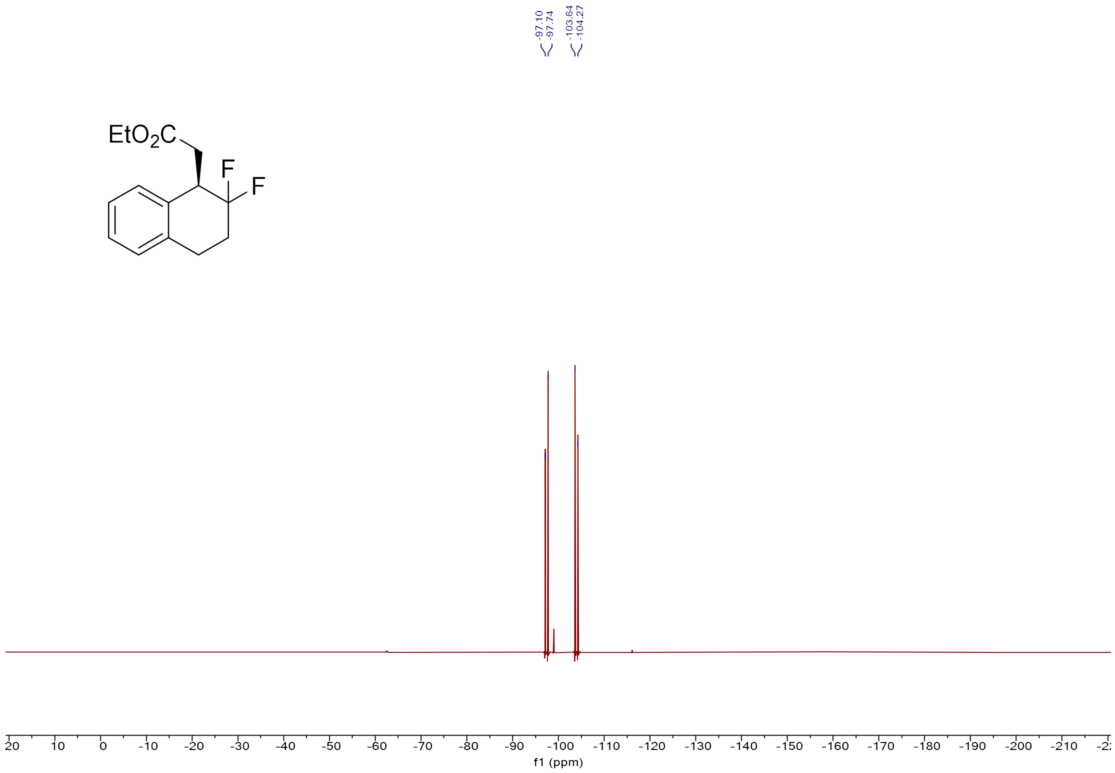
-97.10
-97.74
-103.64
-104.27
EtO2C
F
F
f1 (ppm)

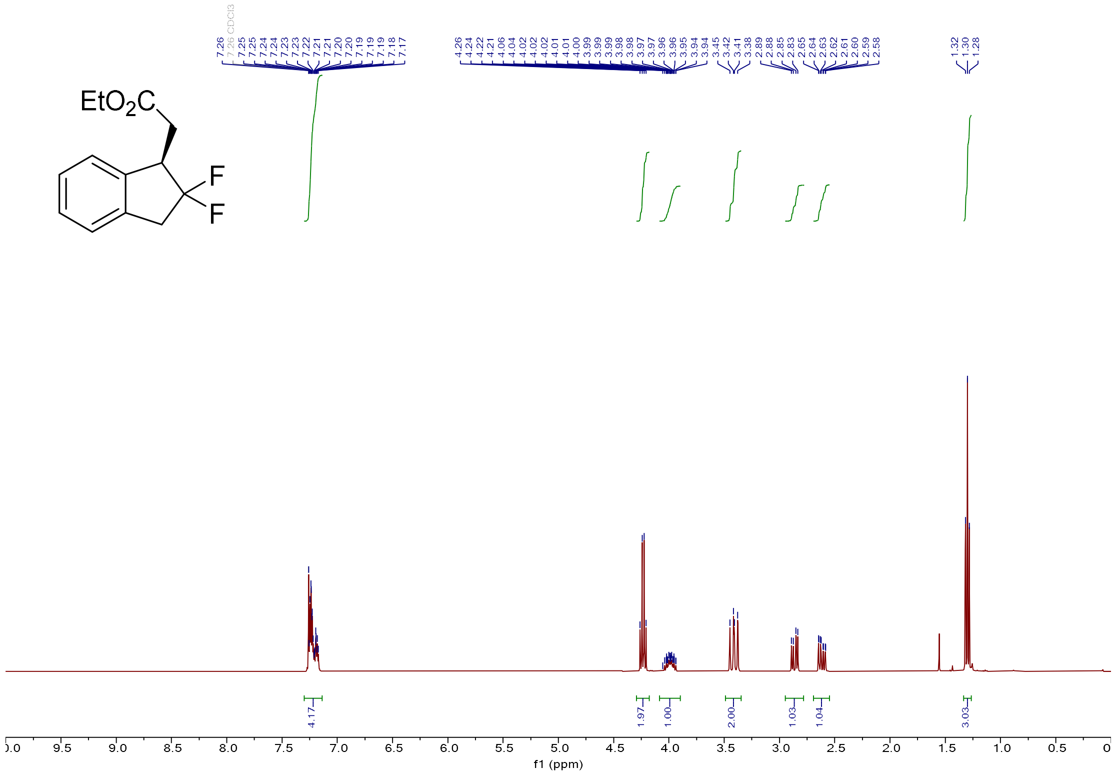

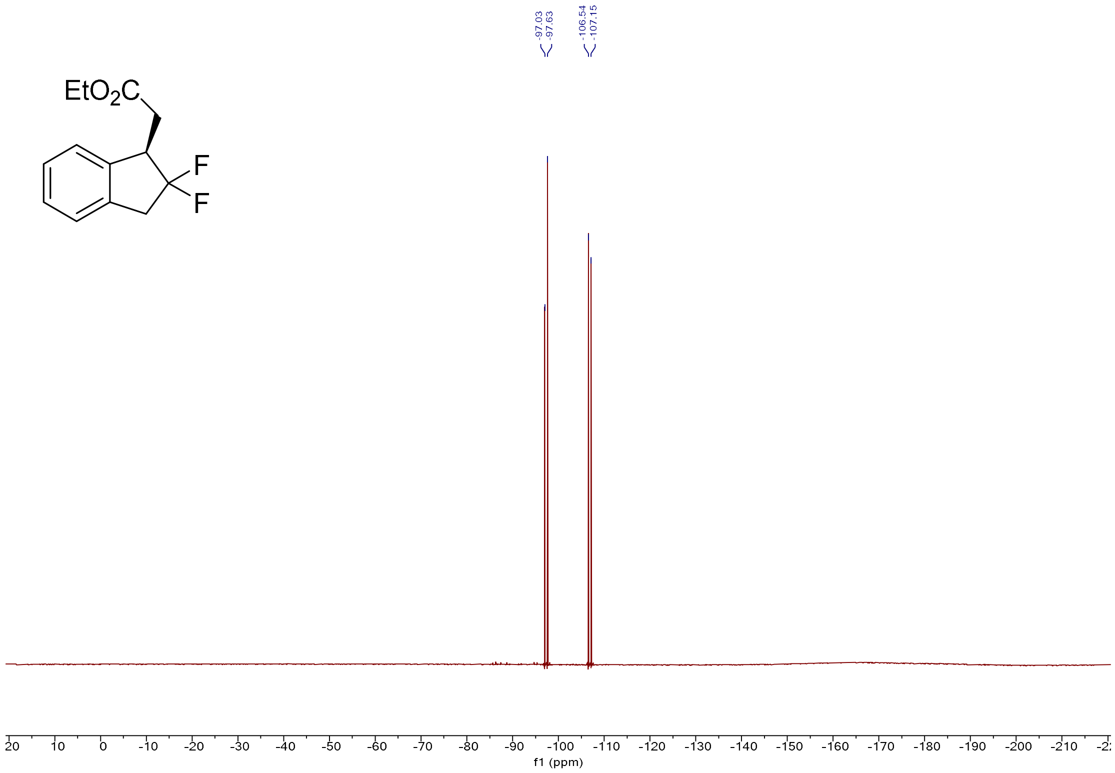

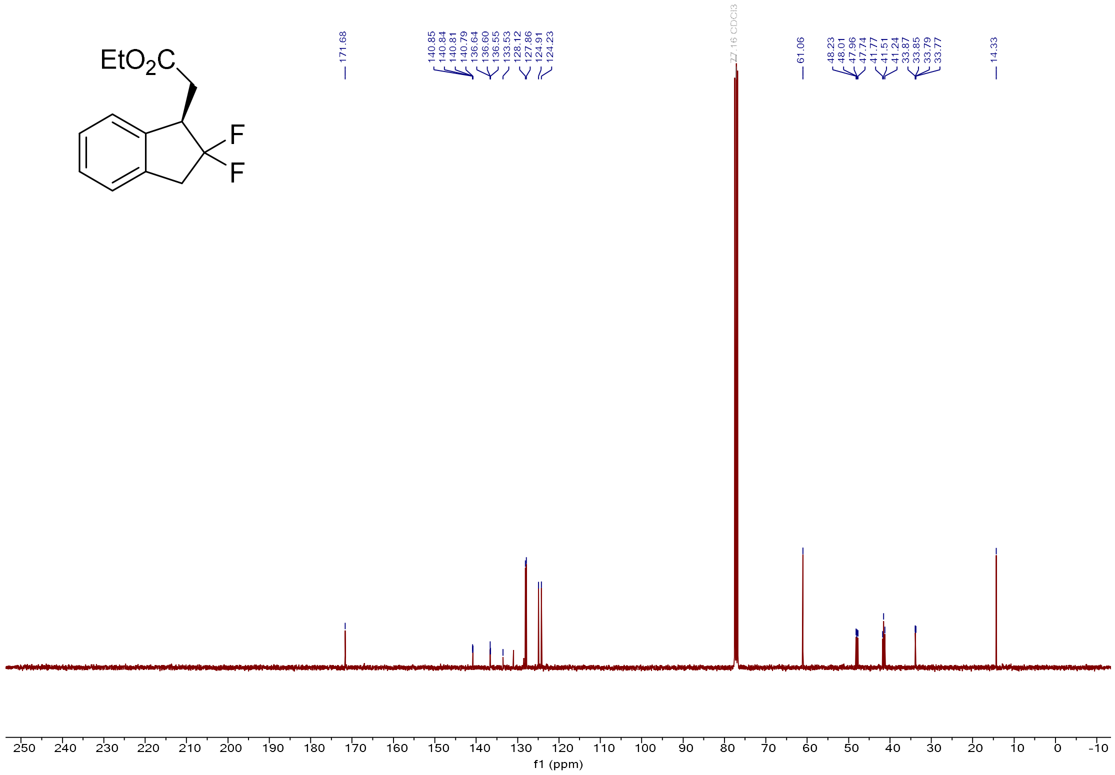
EtO2C
F
F
171.68
140.85
140.84
140.81
140.79
136.64
136.60
136.55
133.53
128.12
127.86
124.91
124.23
77.16 CDCl3
61.06
48.23
48.01
47.96
47.74
41.77
41.51
41.24
33.87
33.85
33.79
33.77
14.33
f1 (ppm)

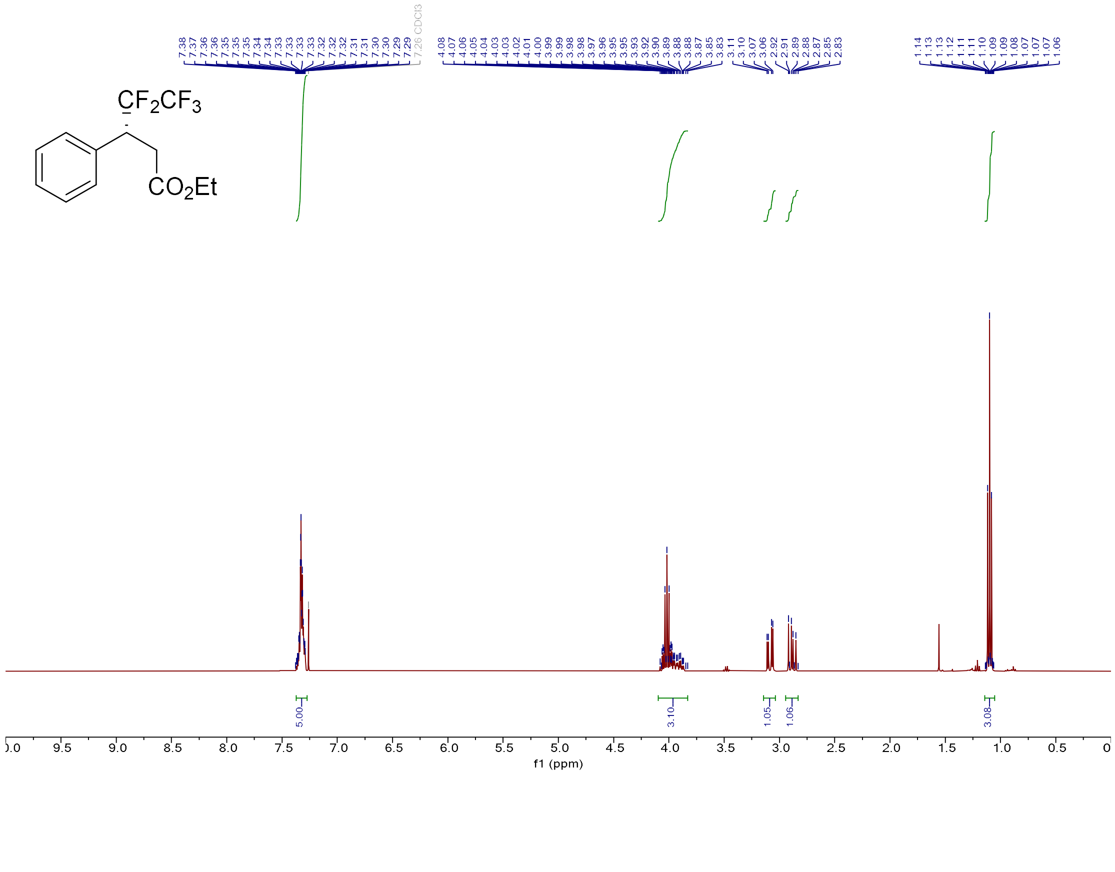
CF2CF3
CO2Et
7.26 CDCl3
5.00
3.10
1.05
1.06
3.08
f1 (ppm)

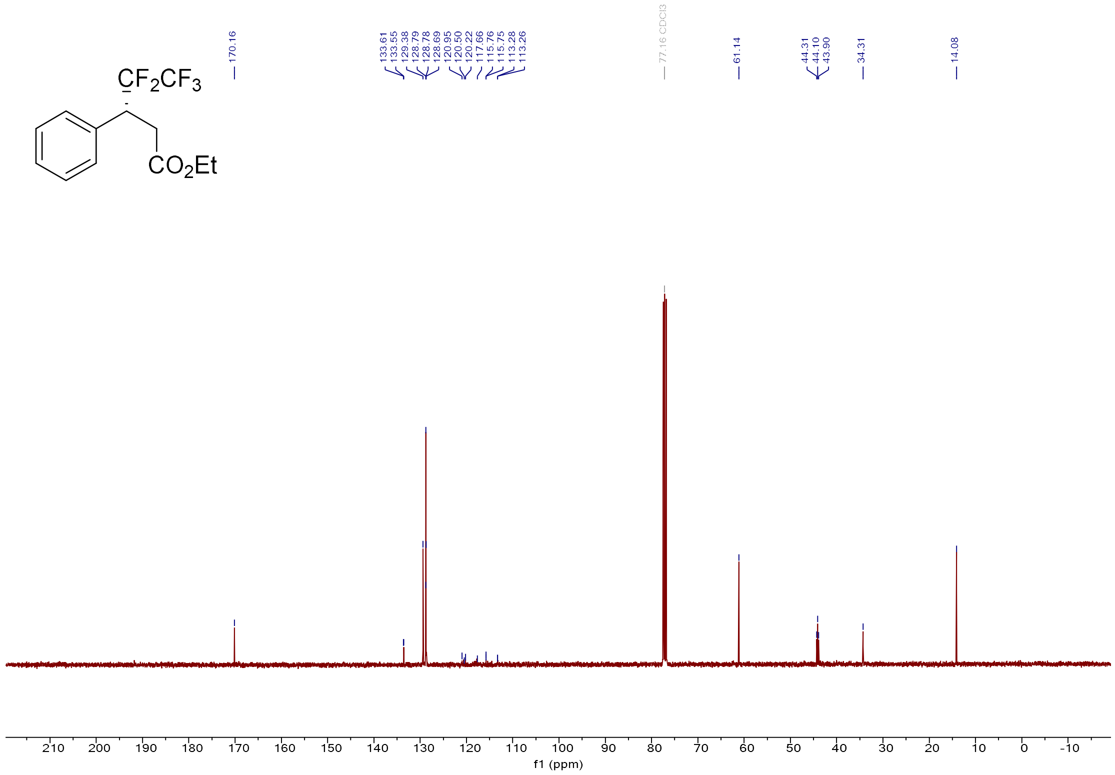
170.16
133.61
133.55
129.38
128.79
128.78
128.69
120.95
120.50
120.22
117.66
115.76
115.75
113.28
113.26
77.16 CDCl3
61.14
44.31
44.10
43.90
34.31
14.08
f1 (ppm)

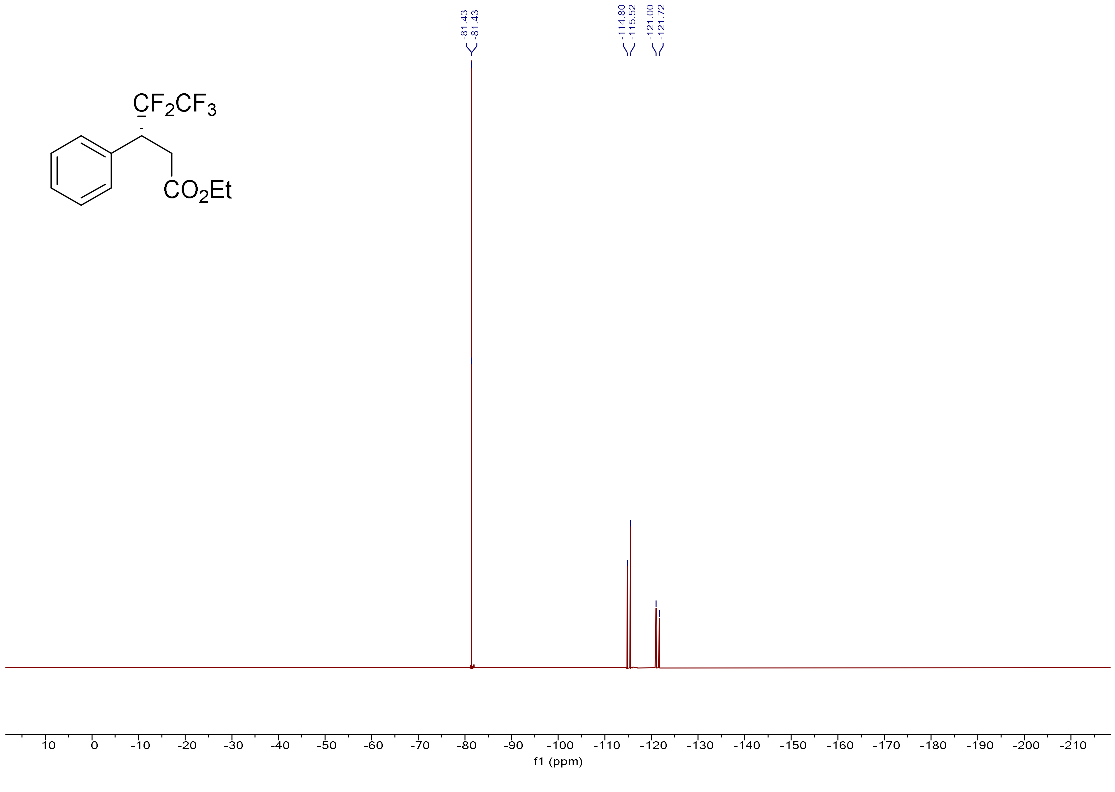
-81.43
-81.43
-114.80
-115.52
-121.00
-121.72
f1 (ppm)

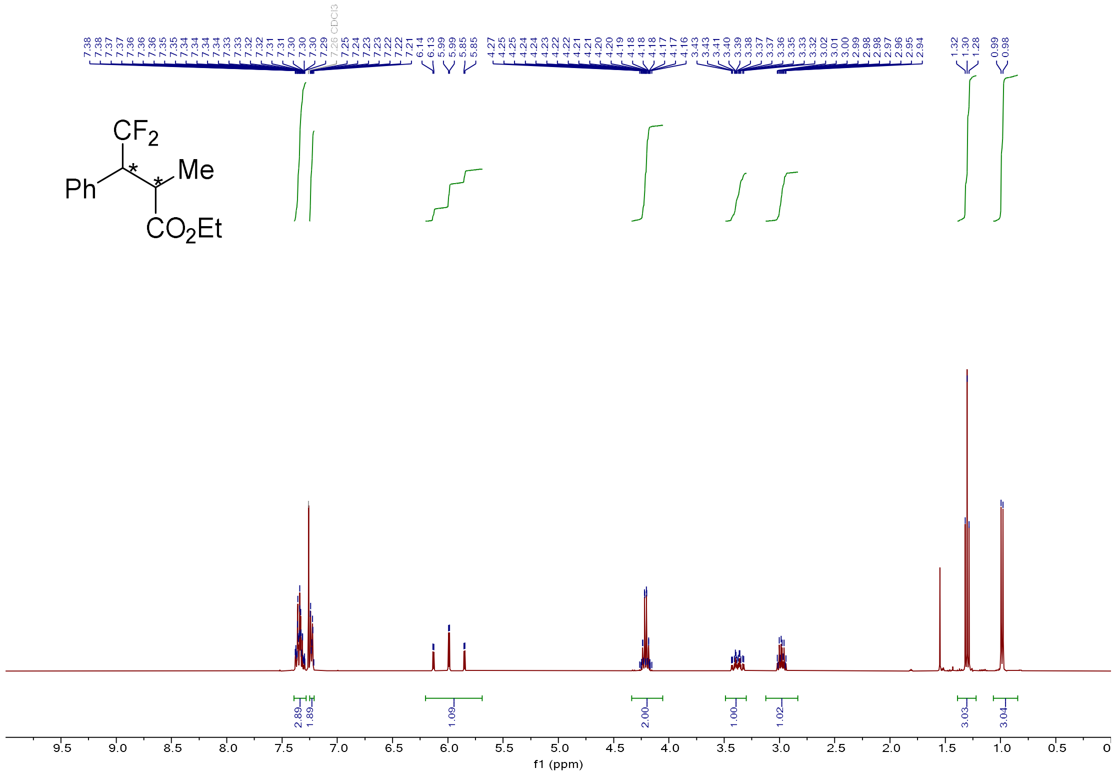

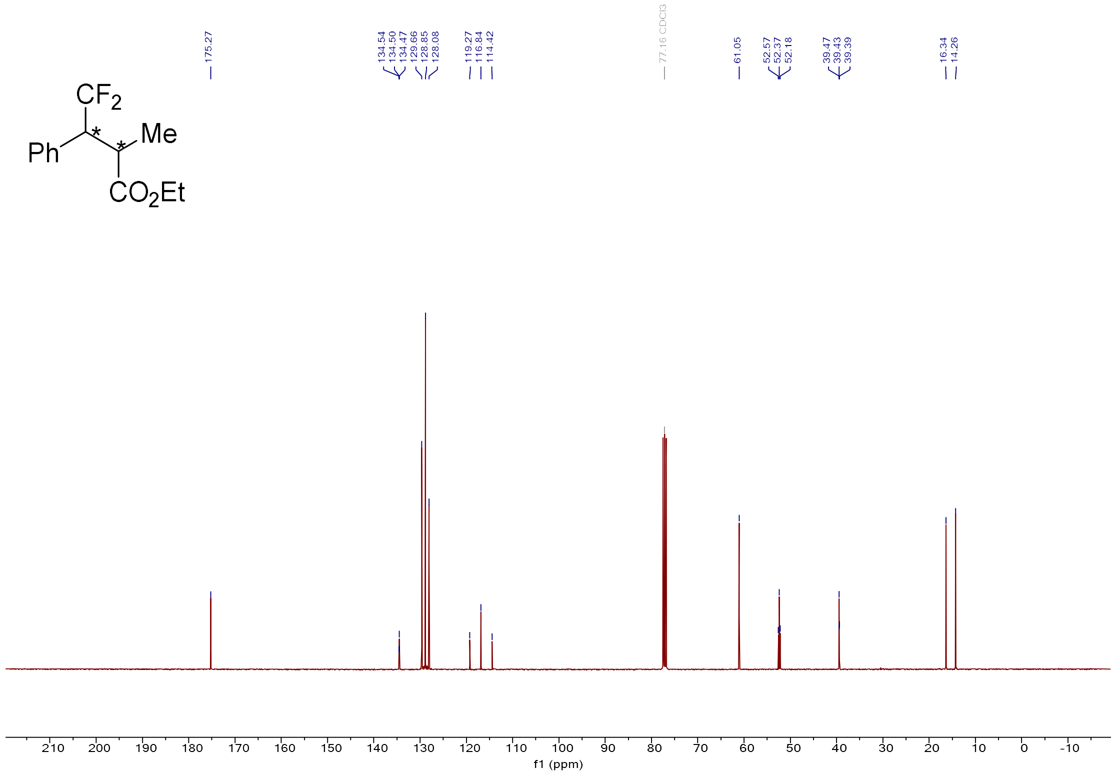

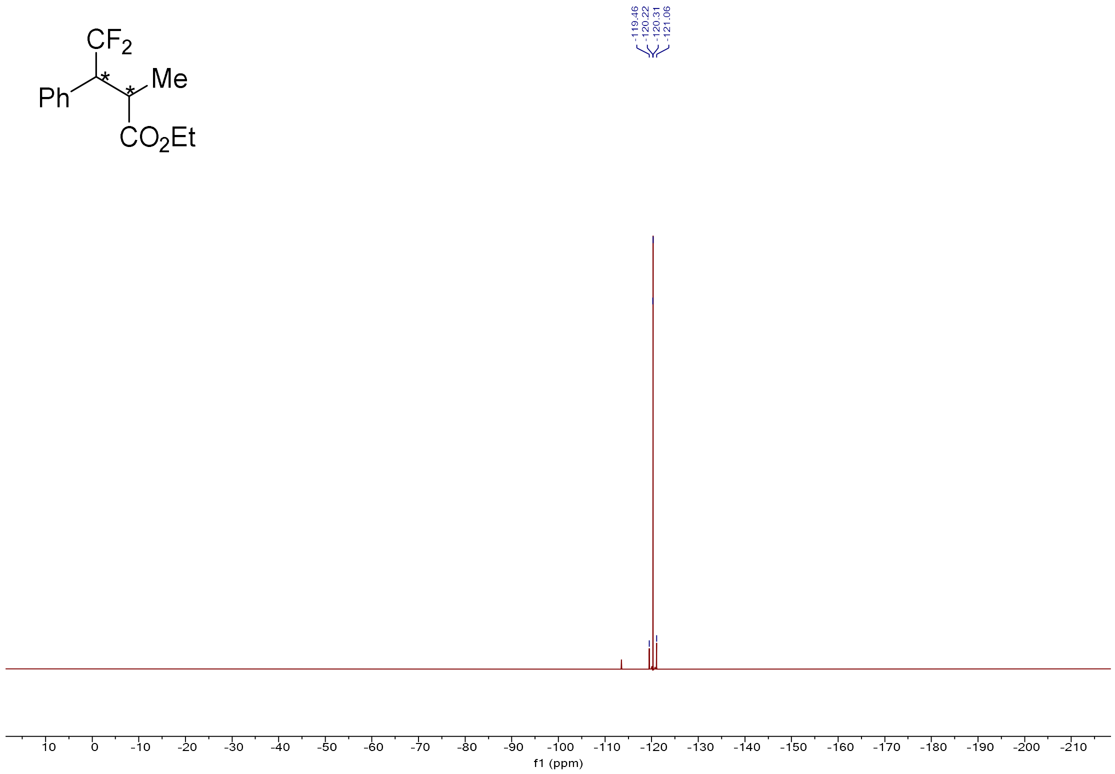
CF2
Ph
Me
CO2Et
-119.46
-120.22
-120.31
-121.06
f1 (ppm)

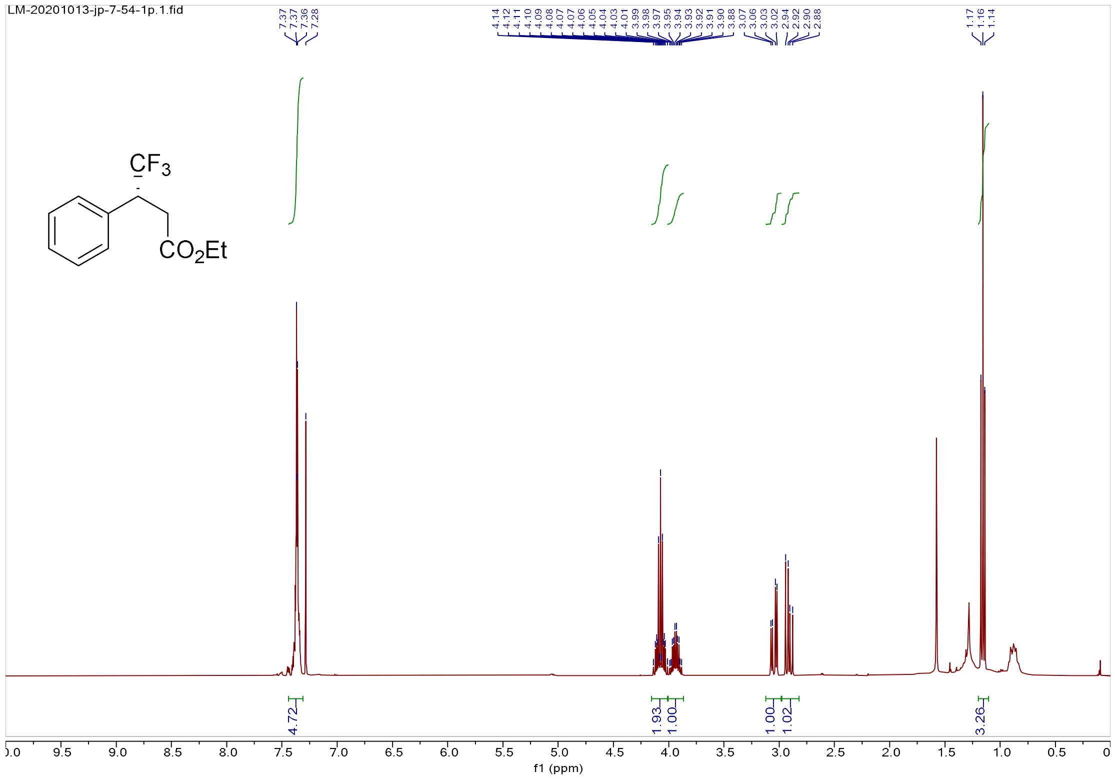
LM-20201013-jp-7-54-1p.1.fid
CF3
CO2Et
4.72
1.93
1.00
1.00
1.02
3.26
f1 (ppm)

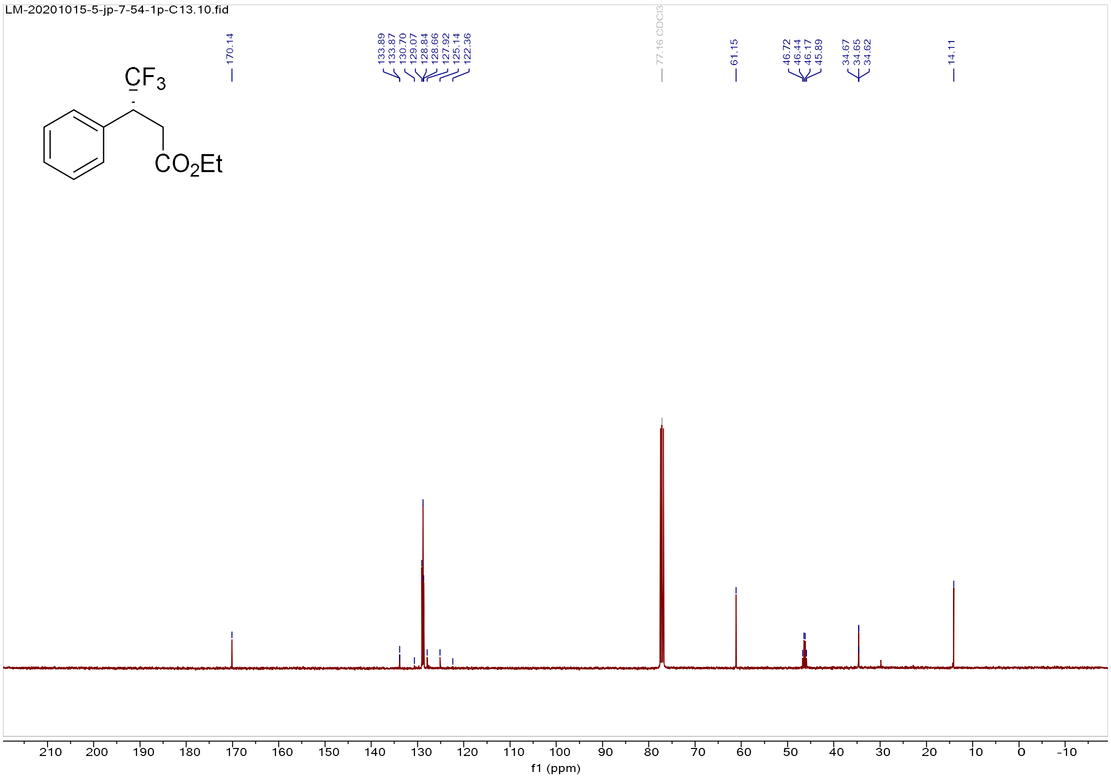
LM-20201015-5-jp-7-54-1p-C13.10.fid
170.14
133.89
133.87
130.70
129.07
128.84
128.66
127.92
125.14
122.36
77.16 CDCl3
61.15
46.72
46.44
46.17
45.89
34.67
34.65
34.62
14.11
CF3
CO2Et
f1 (ppm)

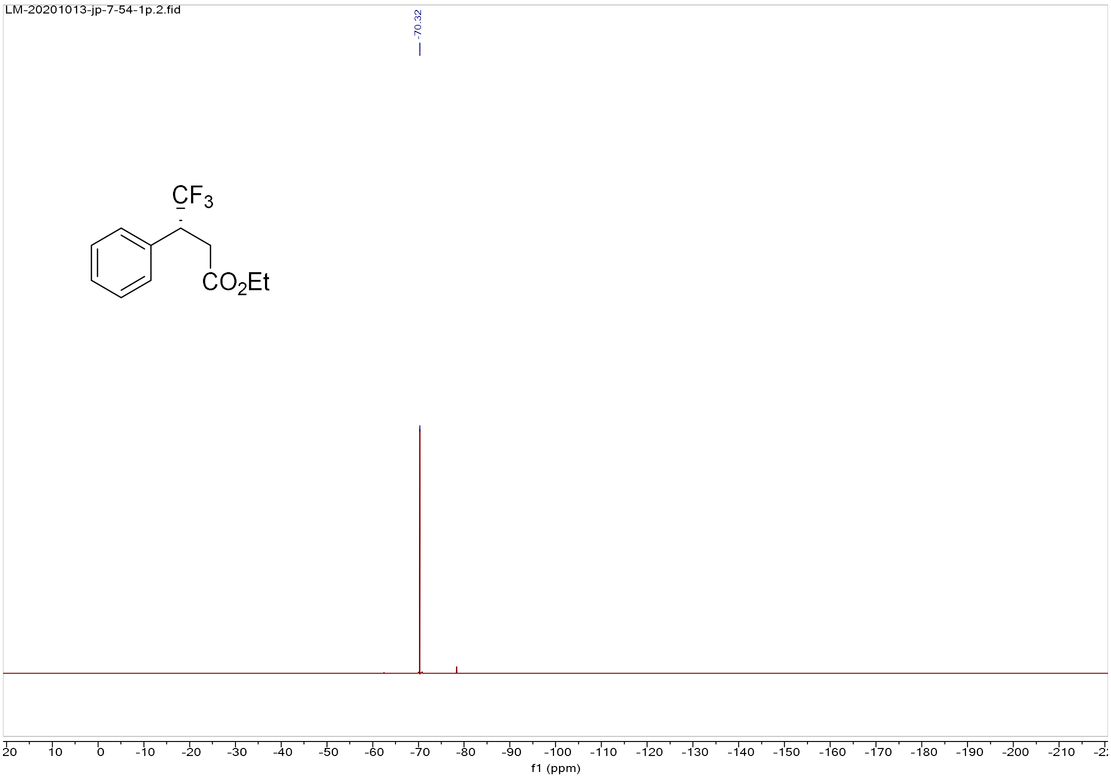
LM-20201013-jp-7-54-1p.2.fid
-70.32
CF3
CO2Et
f1 (ppm)

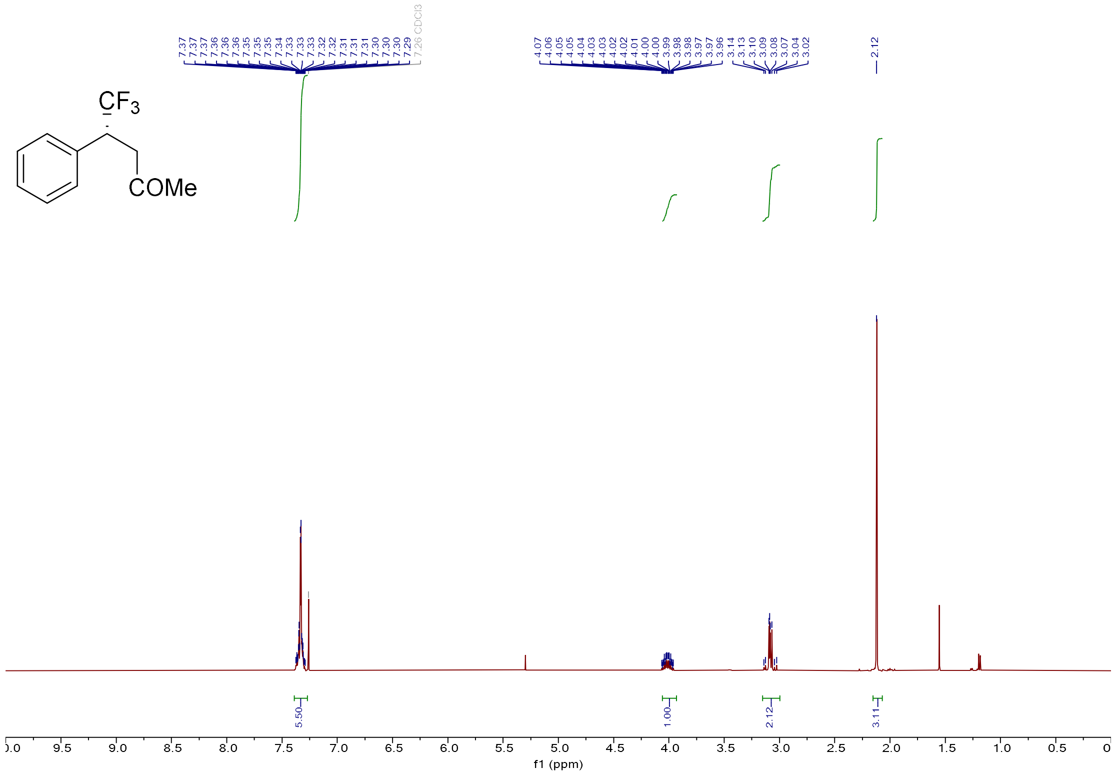

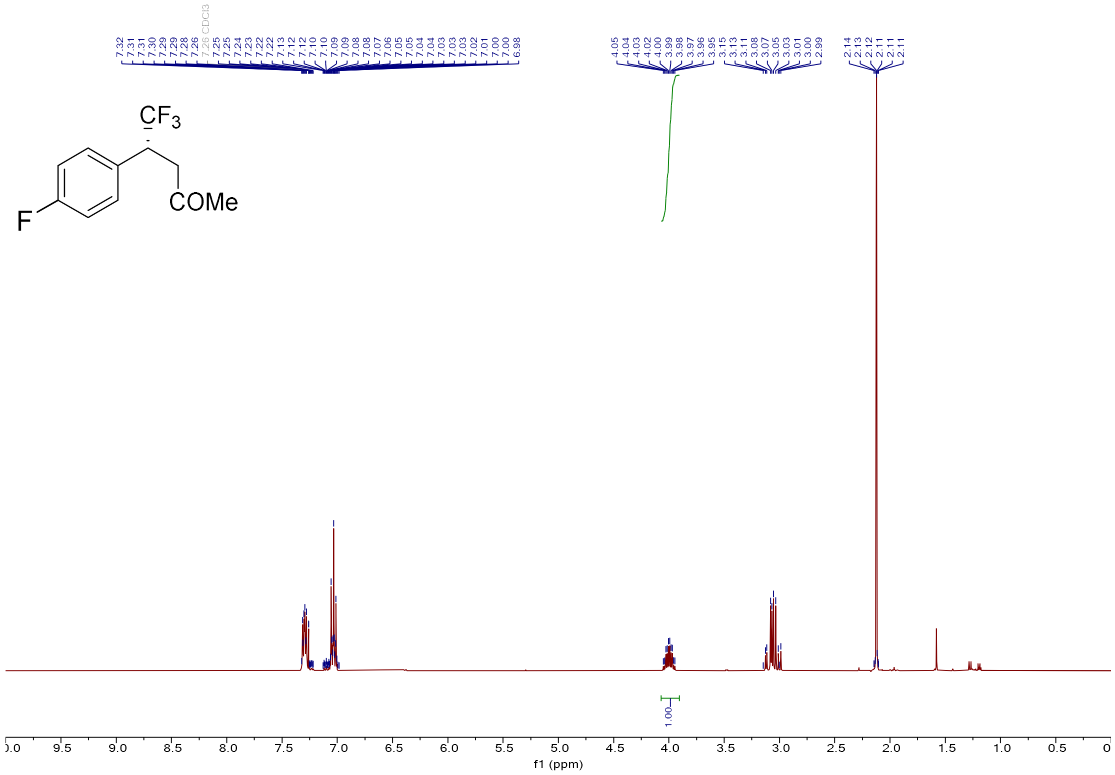

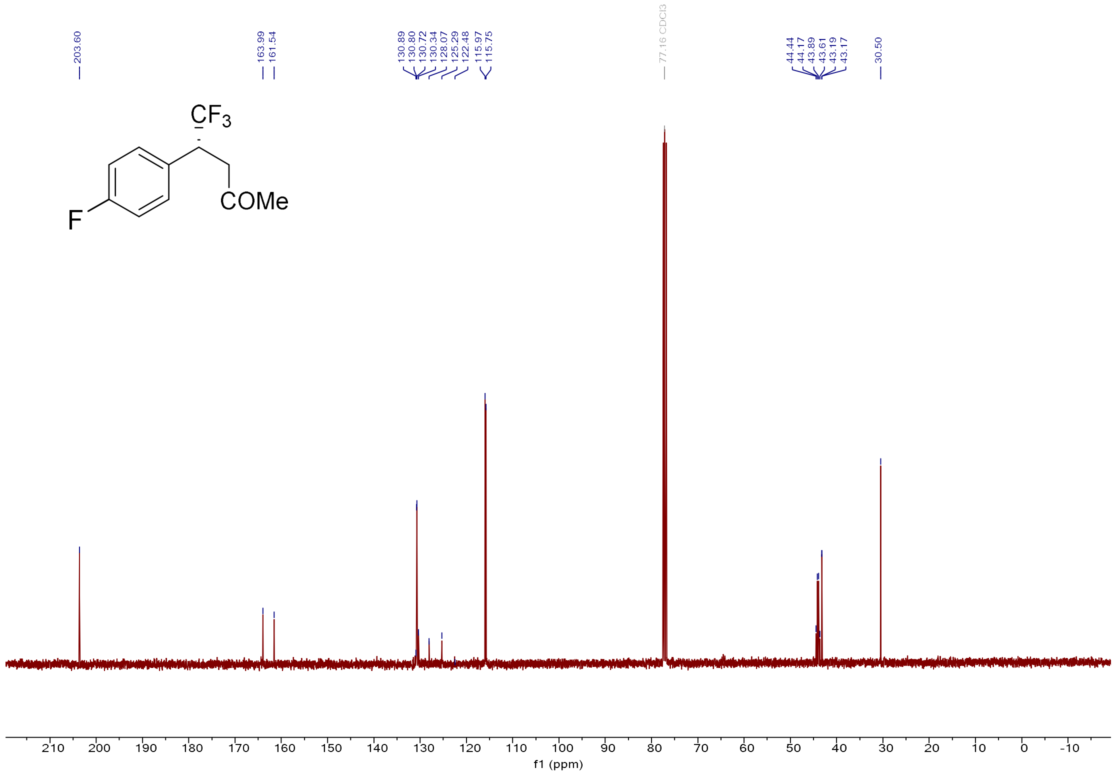
203.60
163.99
161.54
130.89
130.80
130.72
130.34
128.07
125.29
122.48
115.97
115.76
77.16 CDCl3
44.44
44.17
43.89
43.61
43.19
43.17
30.50
CF3
COMe
F
f1 (ppm)

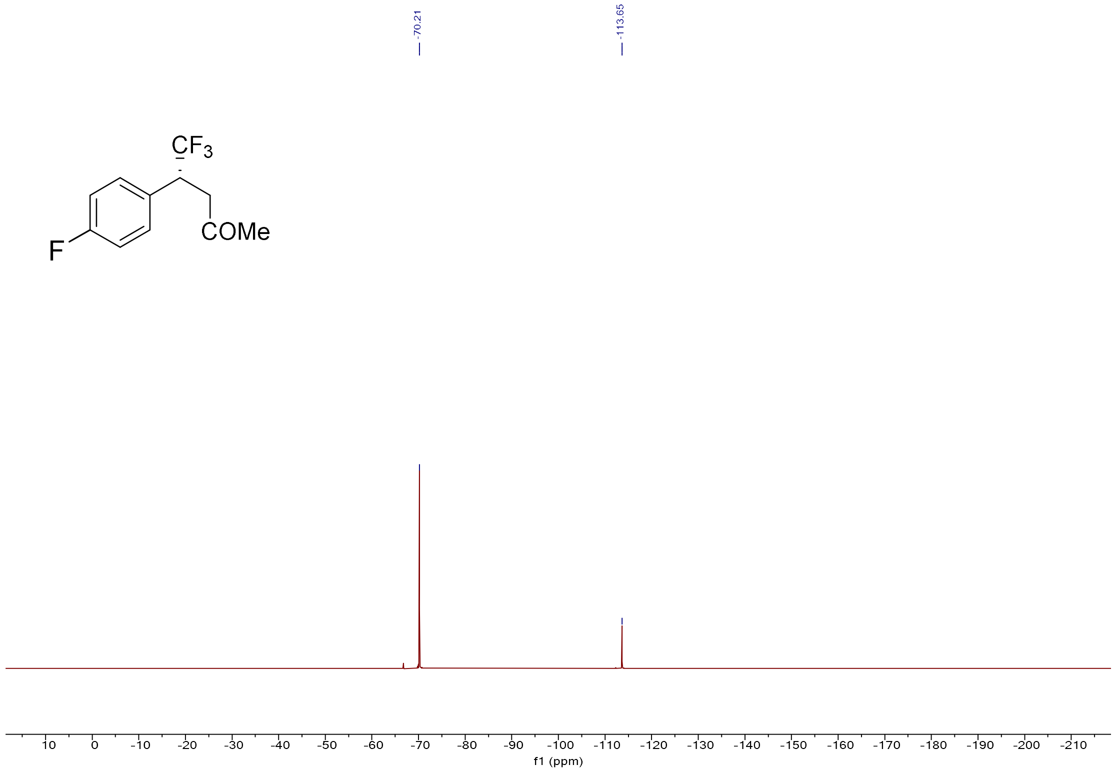
-70.21
-113.65
CF3
COMe
F
f1 (ppm)

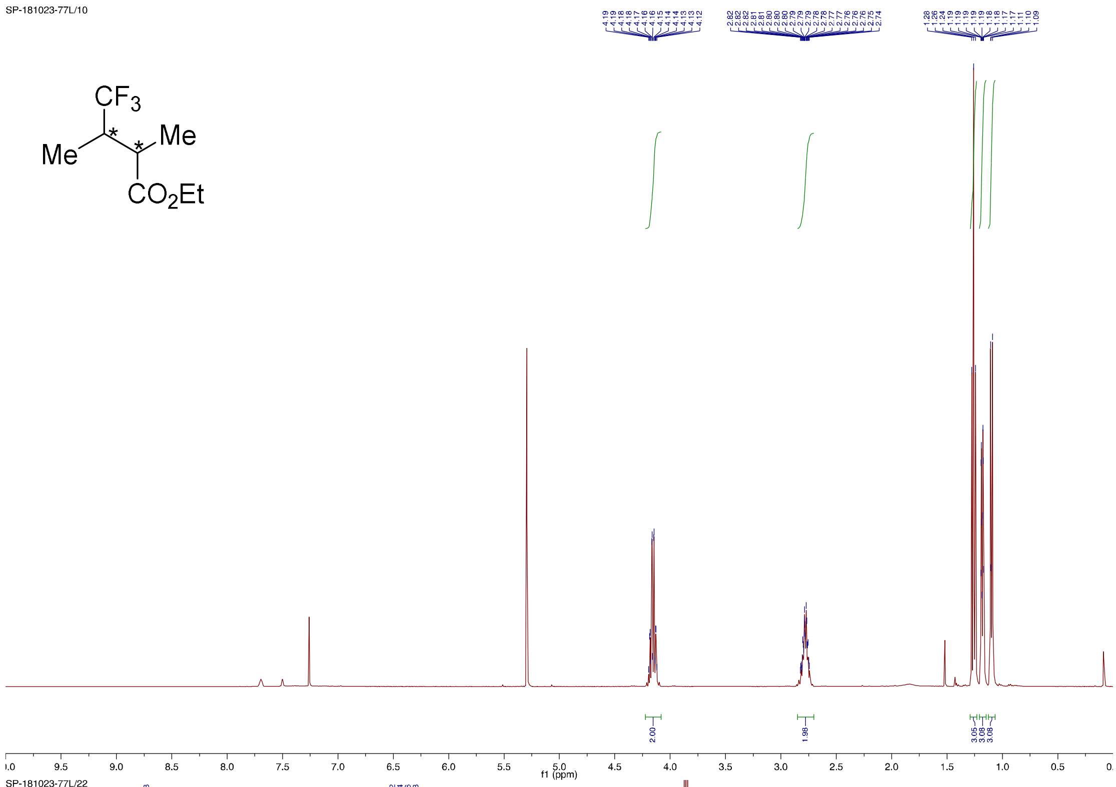
SP-181023-77L/10
CF3
Me
Me
CO2Et
2.00
1.98
3.05
3.08
3.08
f1 (ppm)

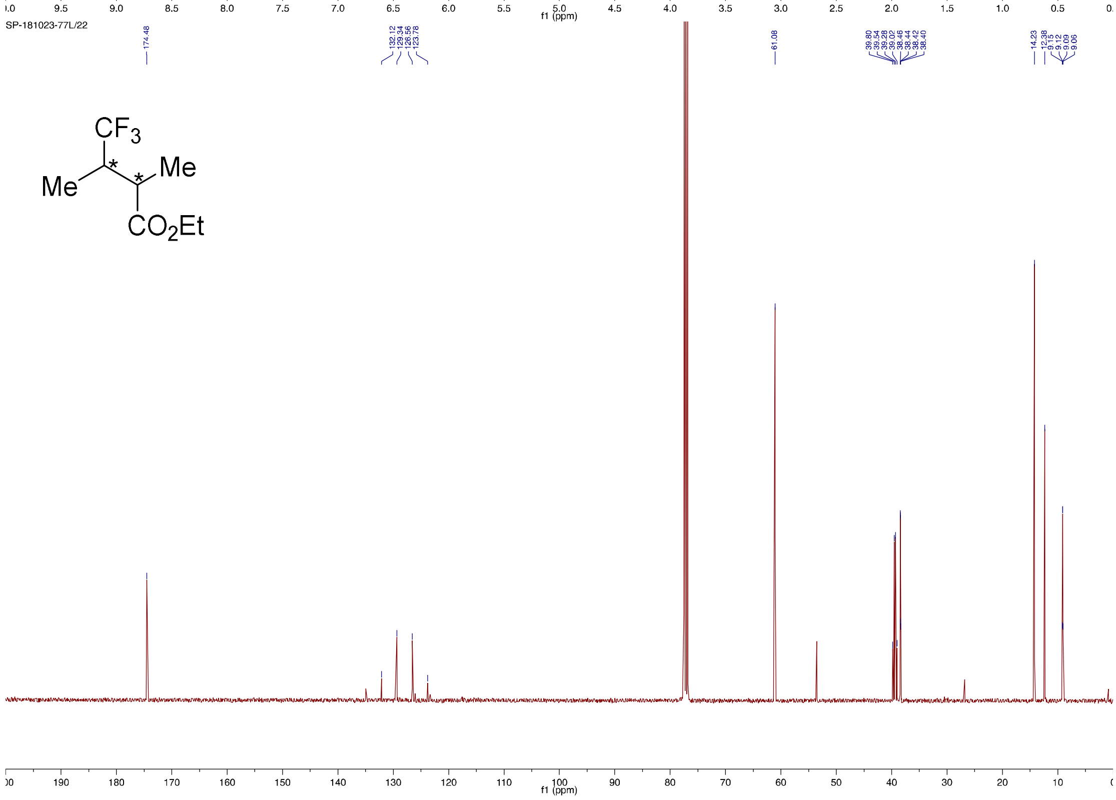
SP-181023-77L/22
174.48
132.12
129.34
126.56
123.78
61.08
14.23
12.38
CF3
Me
Me
CO2Et
f1 (ppm)

SP-181028-77L/10

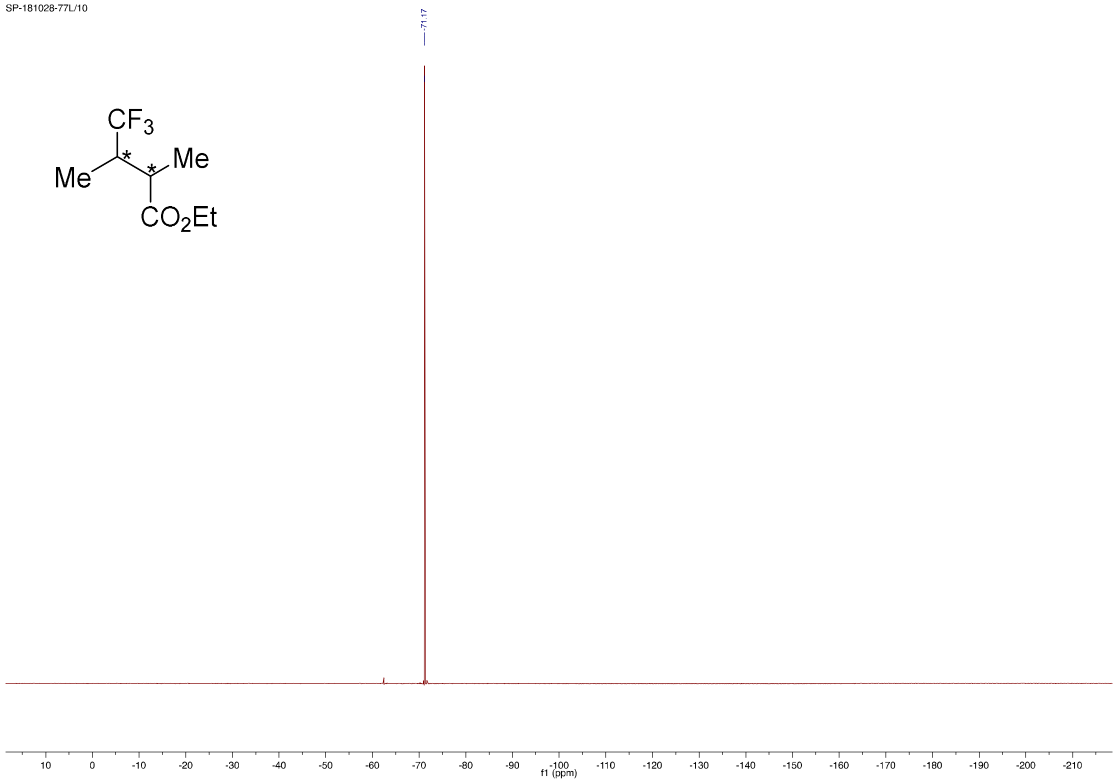

SP-181023-779L/10

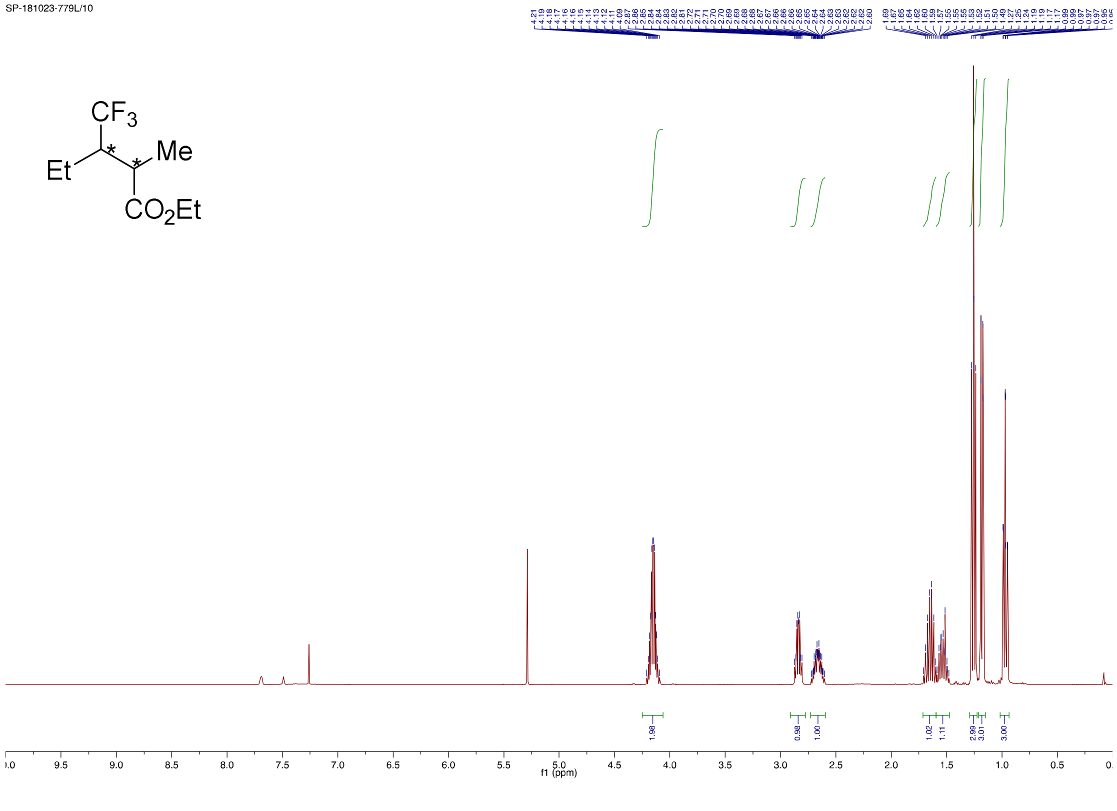

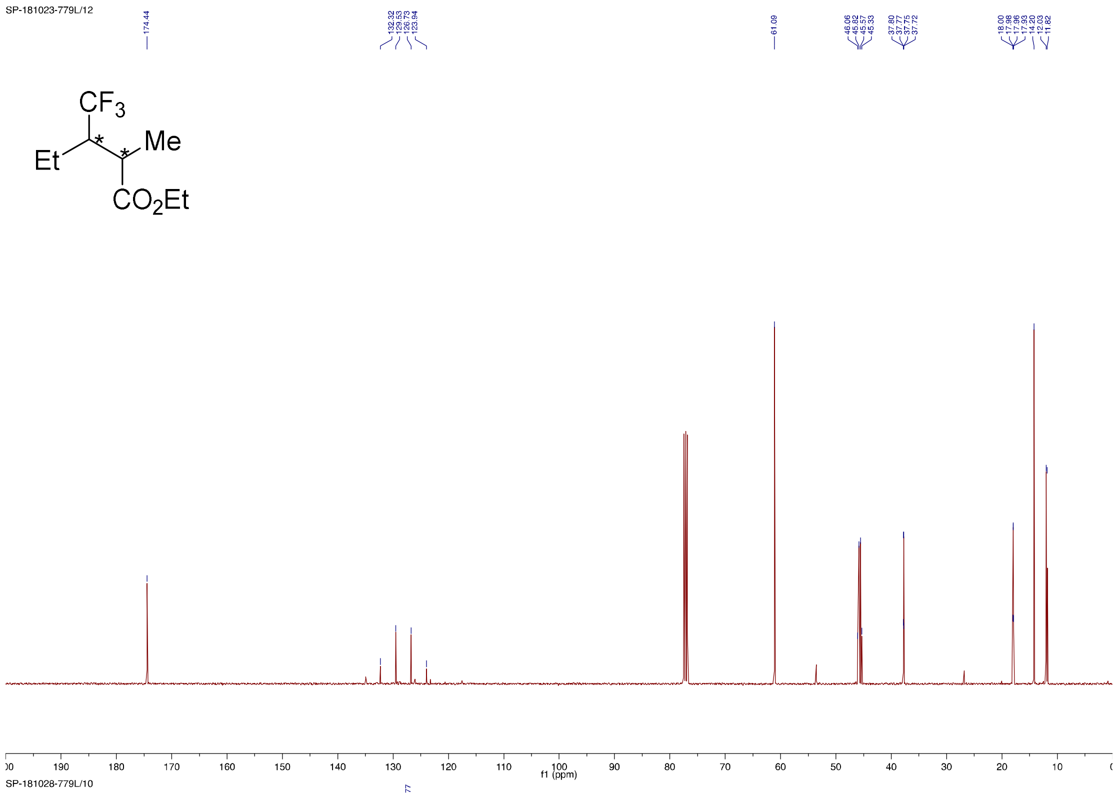
SP-181023-779L/12
CF3
Me
Et
CO2Et
174.44
132.32
129.53
126.73
123.94
61.09
46.06
45.82
45.57
45.33
37.80
37.77
37.75
37.72
18.00
17.98
17.96
17.93
14.20
12.03
11.82
f1 (ppm)

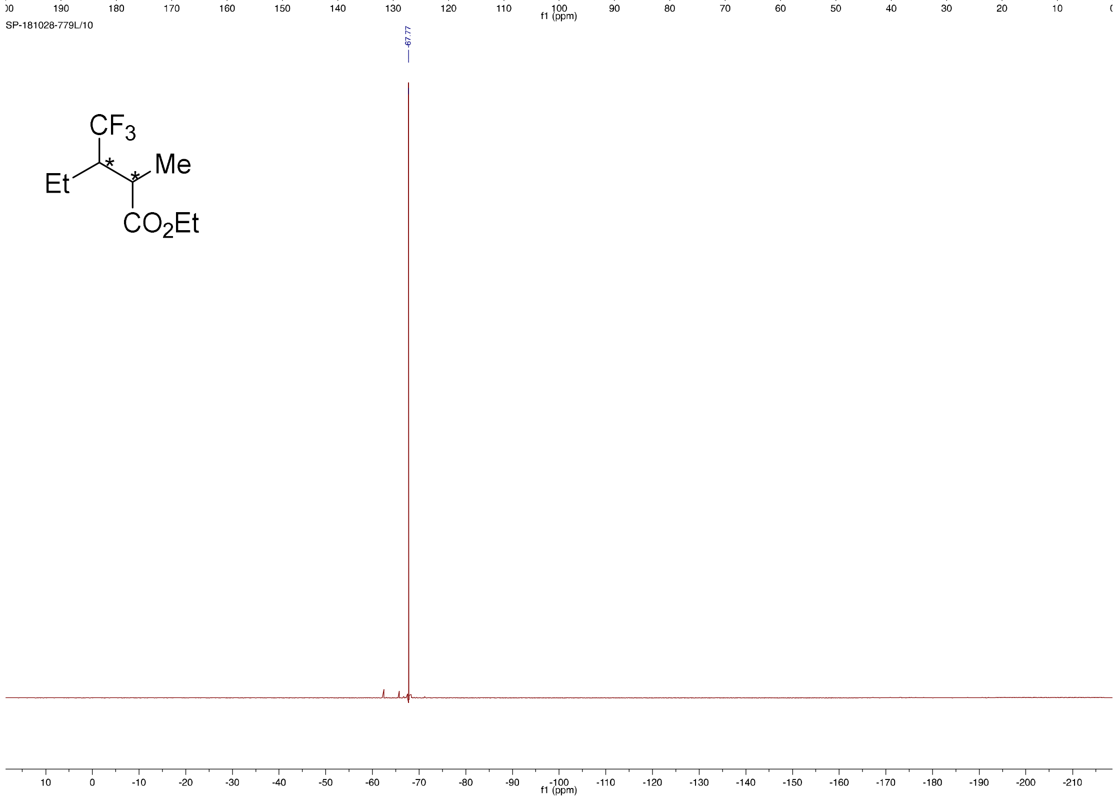
SP-181028-779L/10
-67.77
CF3
Me
Et
CO2Et
f1 (ppm)

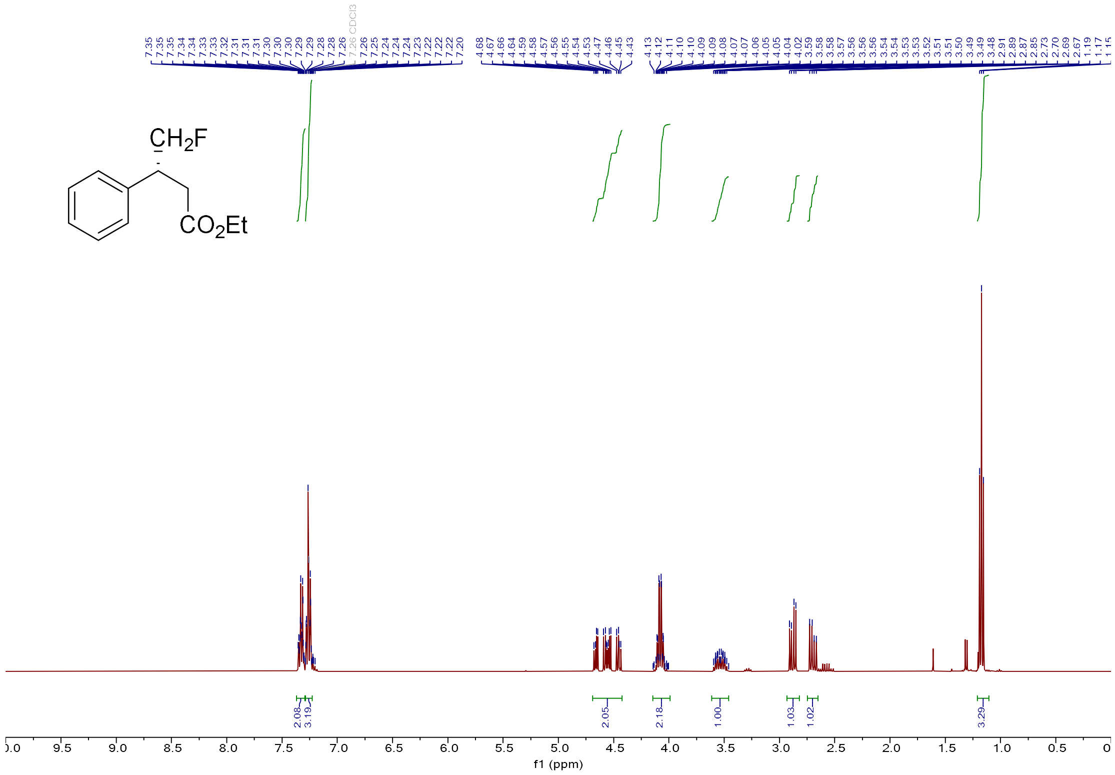

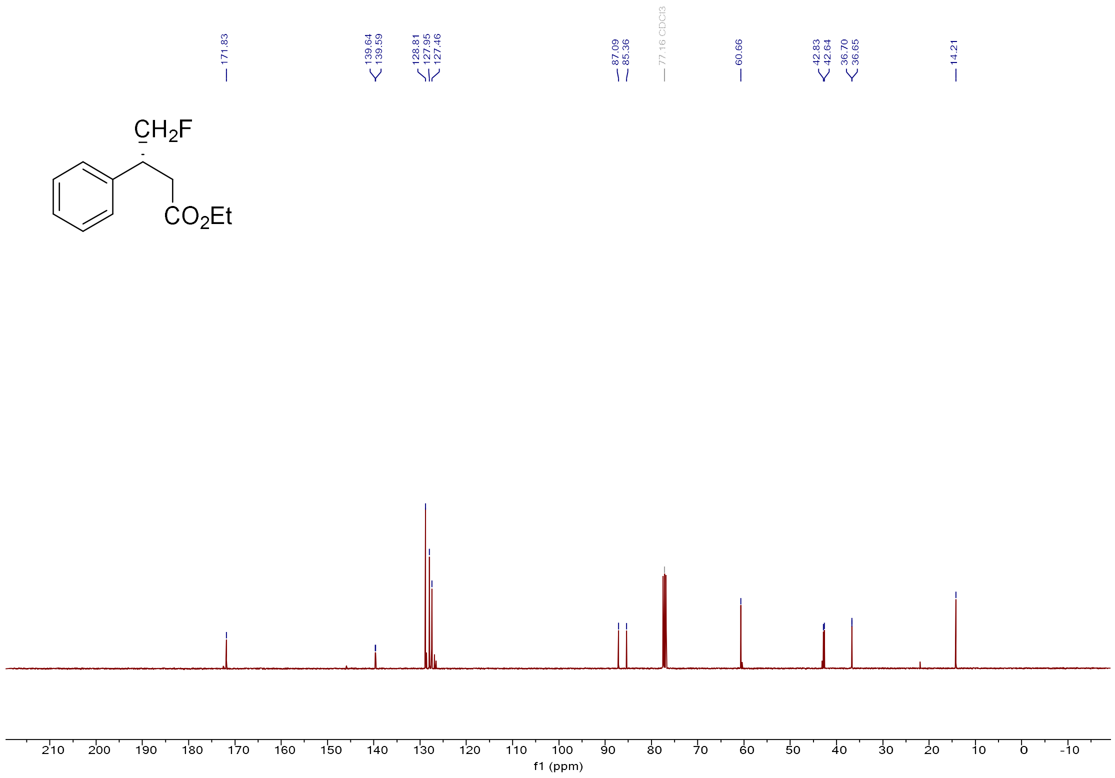

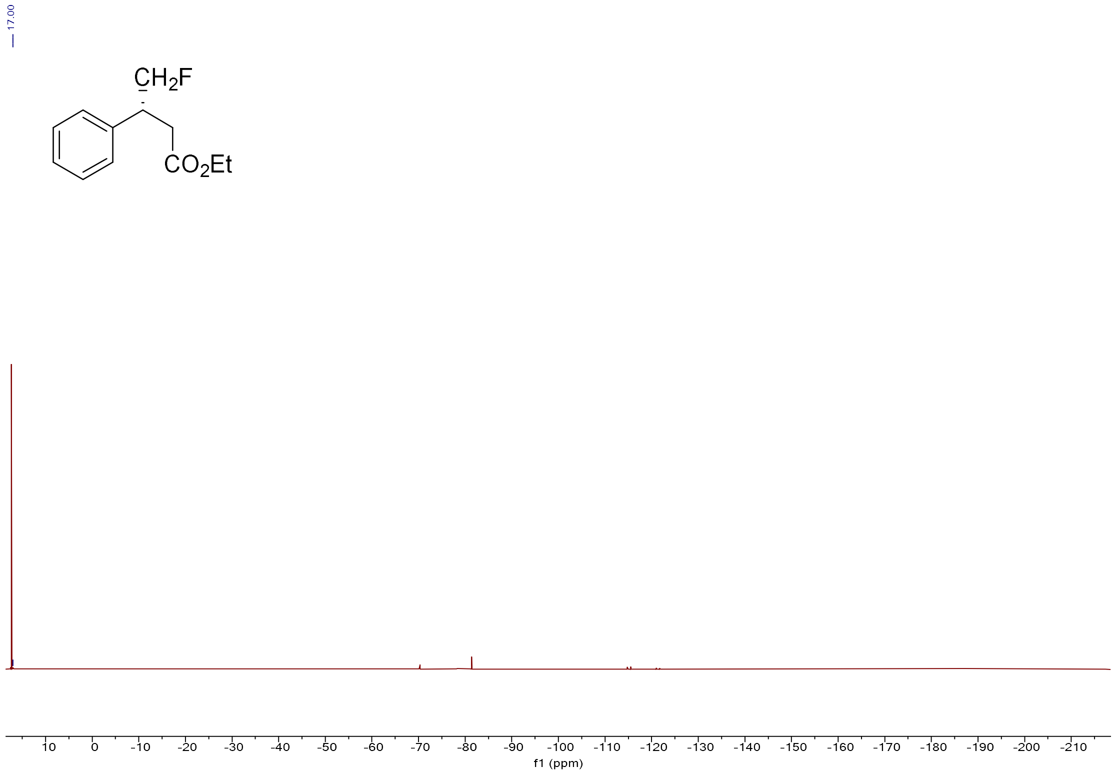

17.00
CH2F
CO2Et
f1 (ppm)

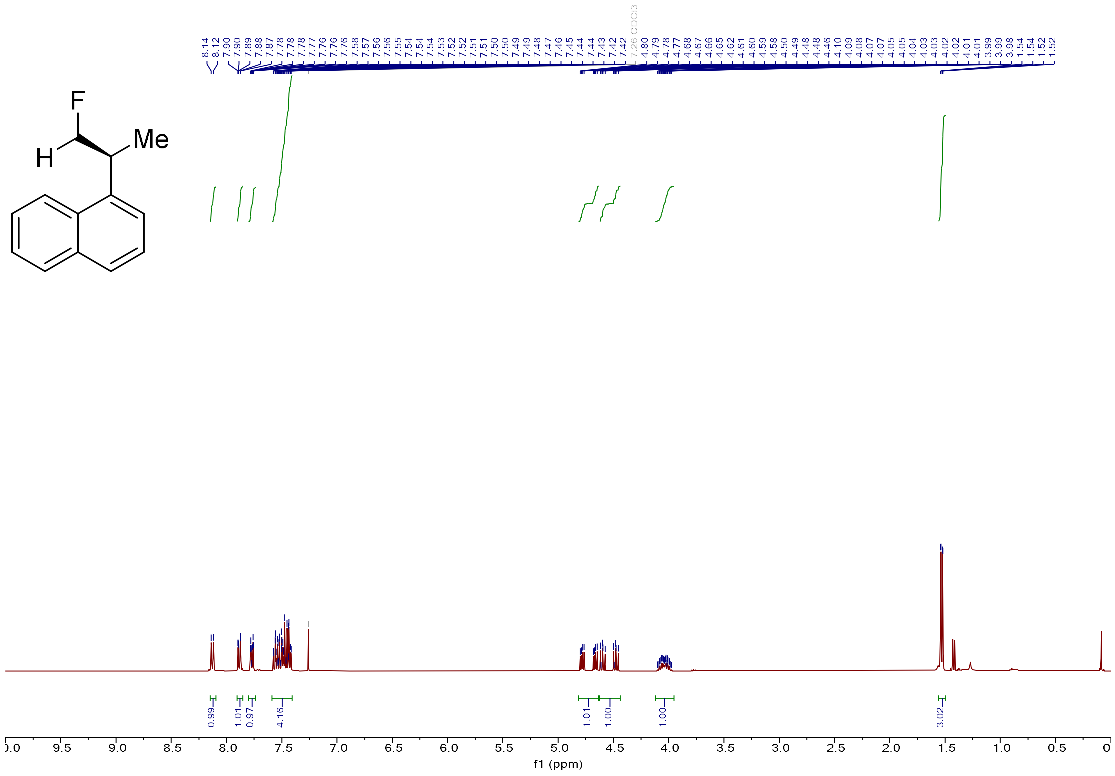

F
H
Me
CDCl3
f1 (ppm)

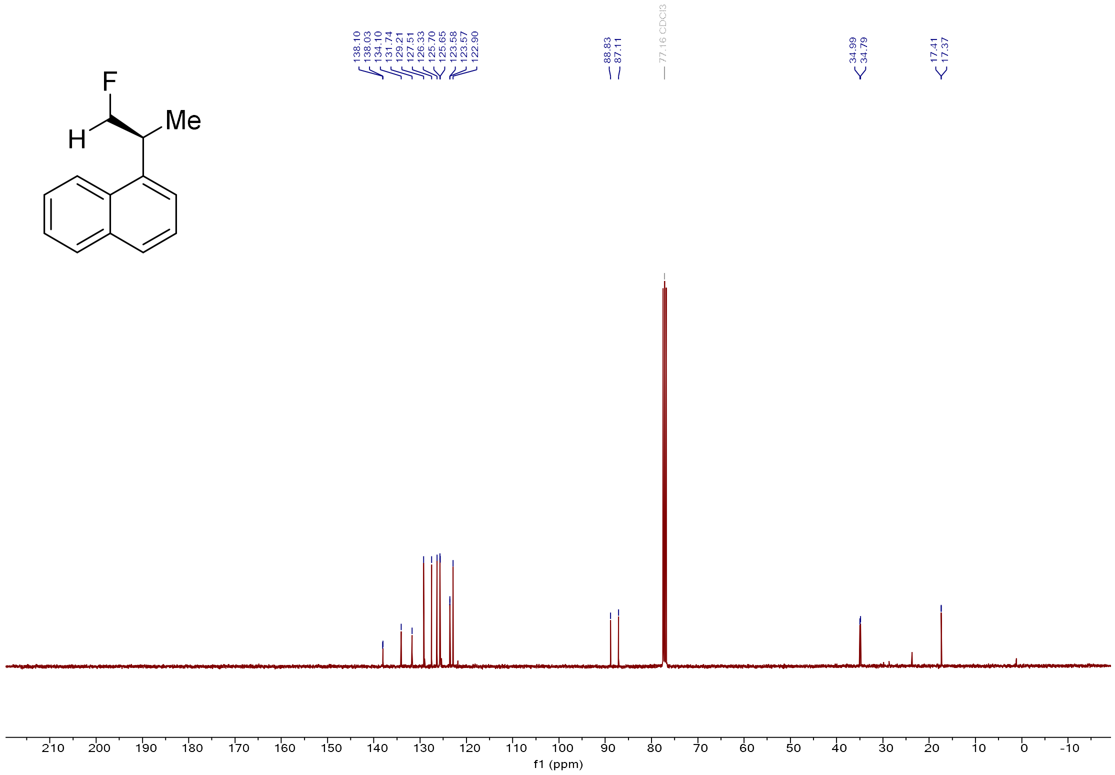
F
H
Me
138.10
138.03
134.10
131.74
129.21
127.51
126.33
125.70
125.65
123.58
123.57
122.90
88.83
87.11
77.16 CDCl3
34.99
34.79
17.41
17.37
f1 (ppm)

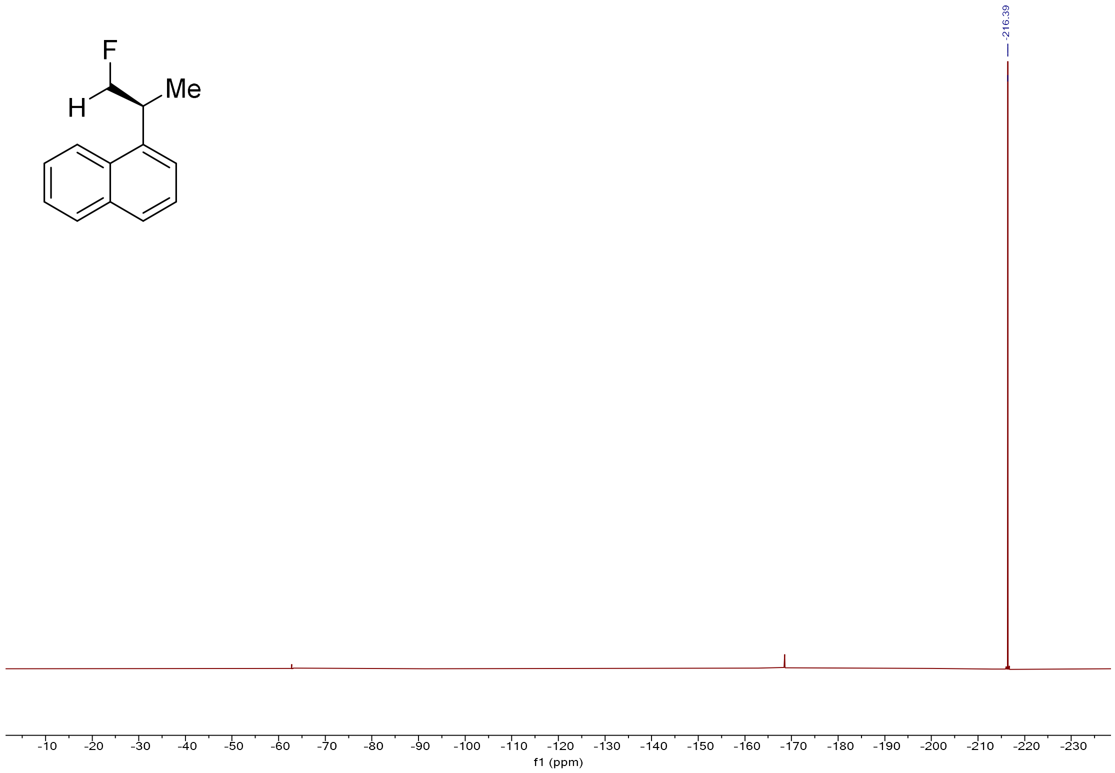
F
H
Me
-216.39
f1 (ppm)

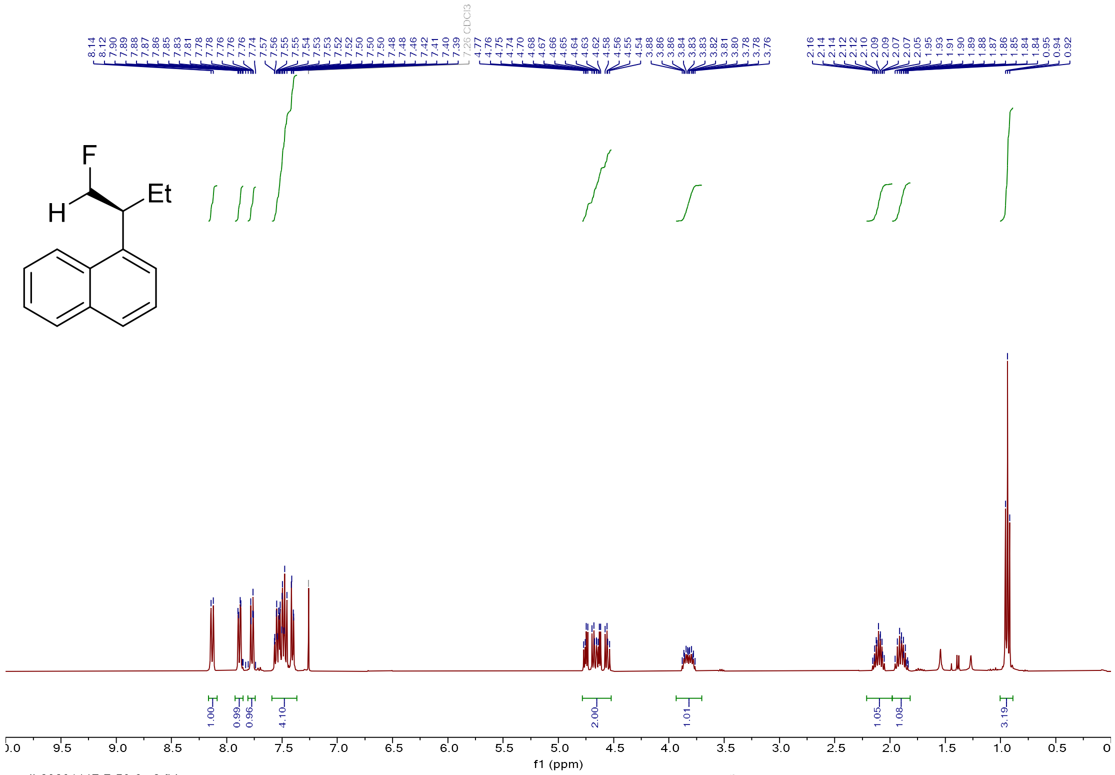

yanji-20201117-7-56-9p.3.fid

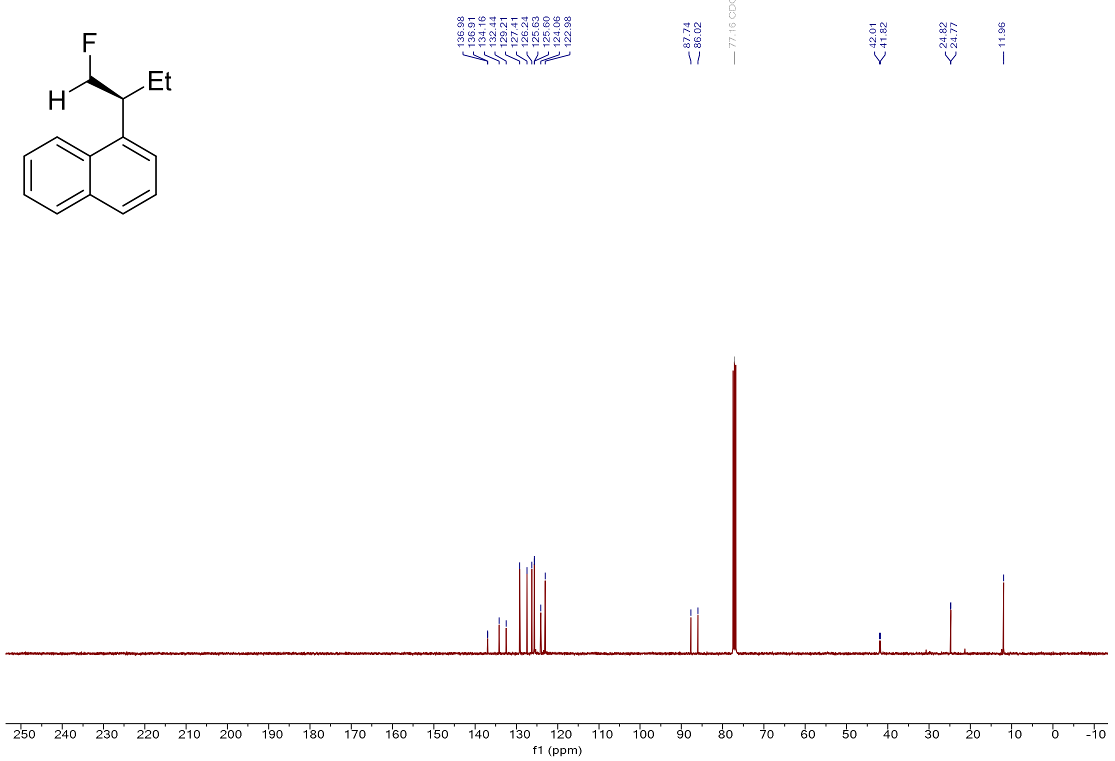

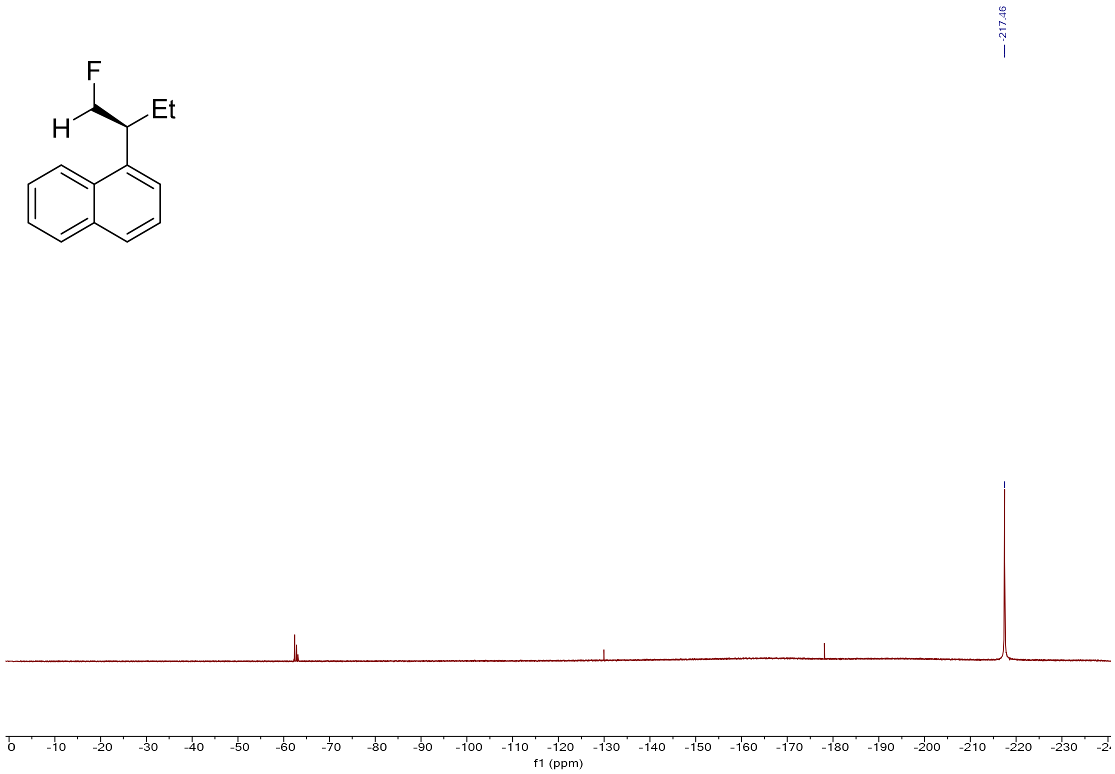

F
H
Et
-217.46
0
-10
-20
-30
-40
-50
-60
-70
-80
-90
-100
-110
-120
-130
-140
-150
-160
-170
-180
-190
-200
-210
-220
-230
f1 (ppm)

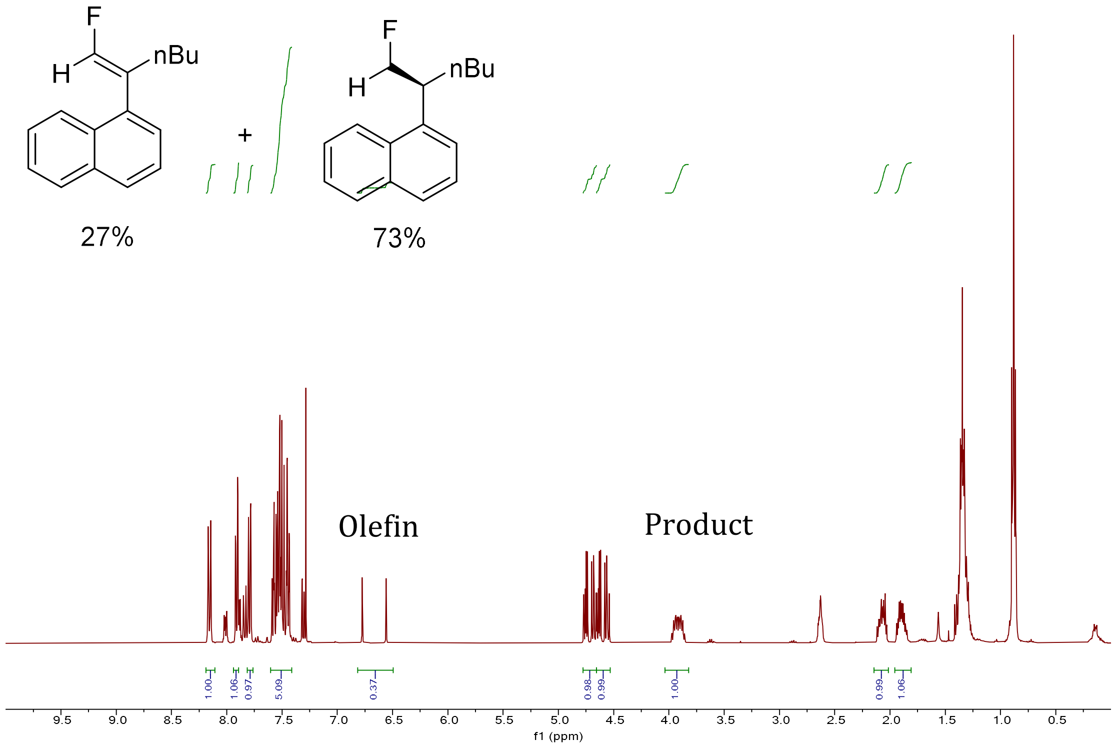

F
H
nBu
+
27%
73%
Olefin
Product
1.00
1.06
0.97
5.09
0.37
0.98
0.99
1.00
0.99
1.06
9.5
9.0
8.5
8.0
7.5
7.0
6.5
6.0
5.5
5.0
4.5
4.0
3.5
3.0
2.5
2.0
1.5
1.0
0.5
f1 (ppm)

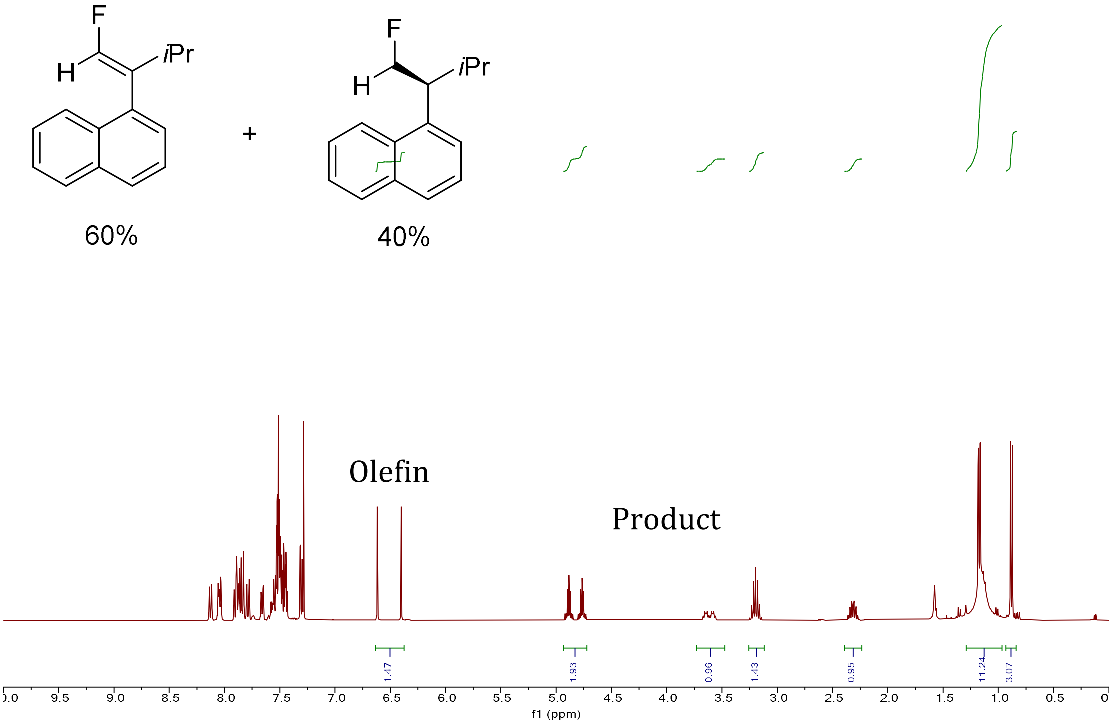
F
iPr
H
+
F
H
iPr
60%
40%
Olefin
Product
1.47
1.93
0.96
1.43
0.95
11.24
3.07
f1 (ppm)

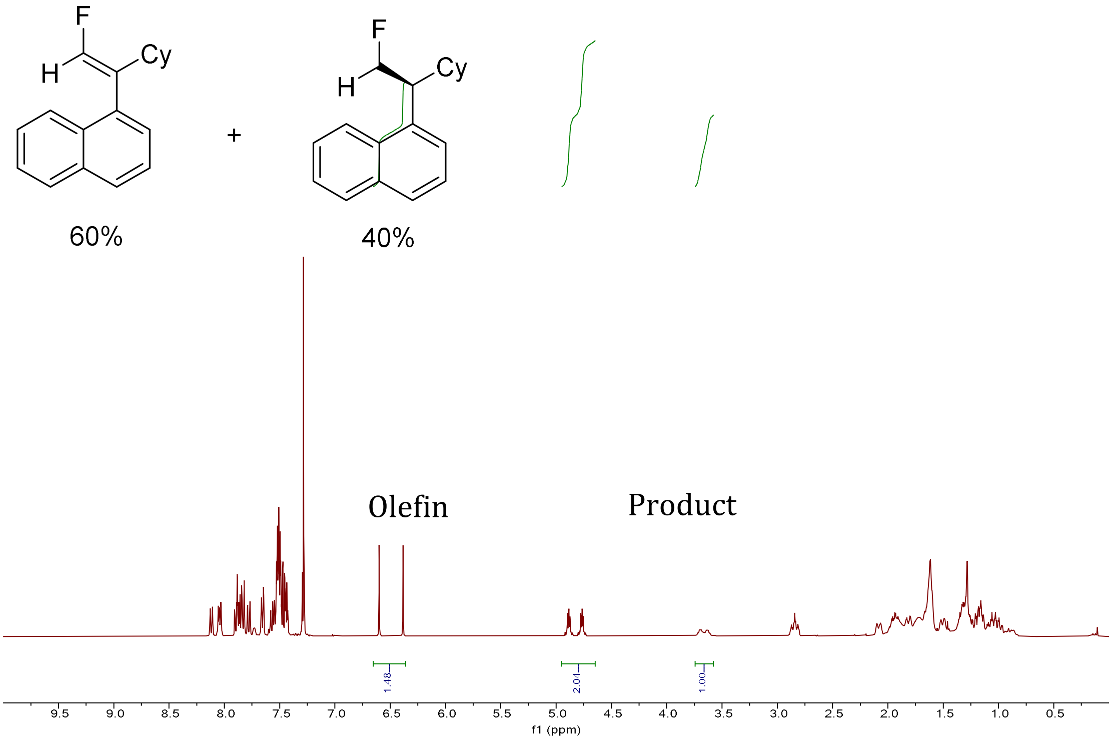
F
Cy
H
+
F
H
Cy
60%
40%
Olefin
Product
1.48
2.04
1.00
f1 (ppm)

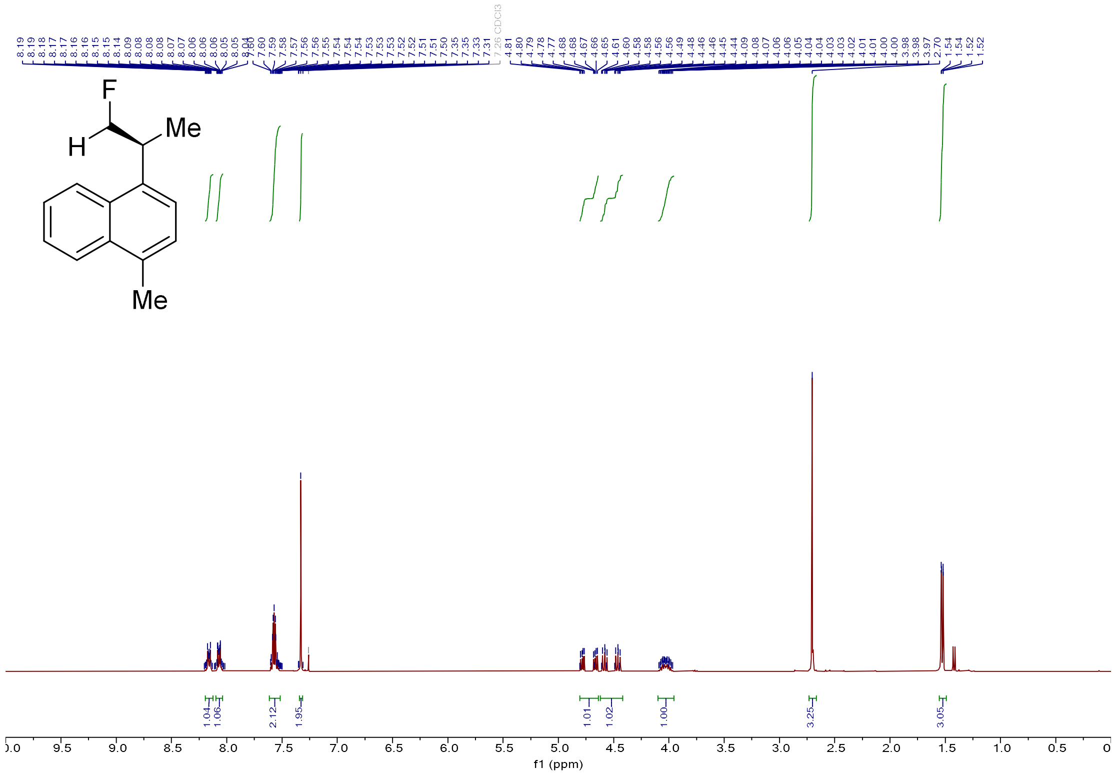
F
H
Me
Me
1.04
1.06
2.12
1.95
1.01
1.02
1.00
3.25
3.05
f1 (ppm)
7.26 CDCl3

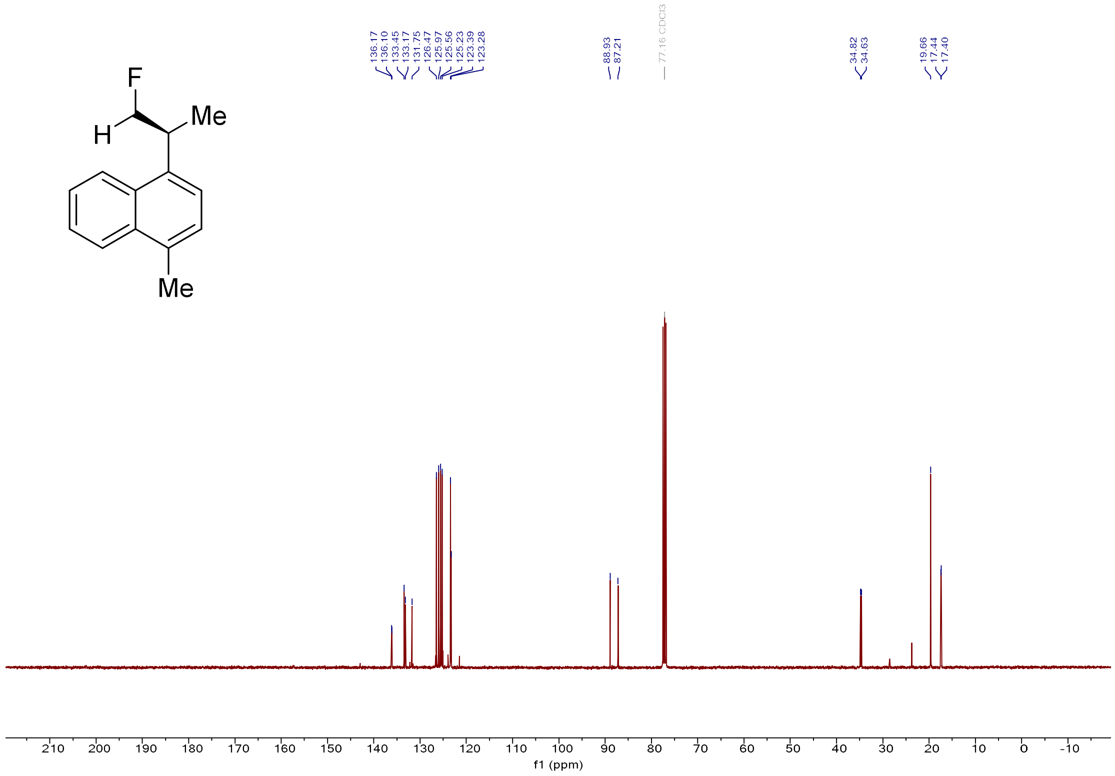
F
H
Me
Me
136.17
136.10
133.45
133.17
131.75
126.47
125.97
125.56
125.23
123.39
123.28
88.93
87.21
77.16 CDCl3
34.82
34.63
19.66
17.44
17.40
f1 (ppm)

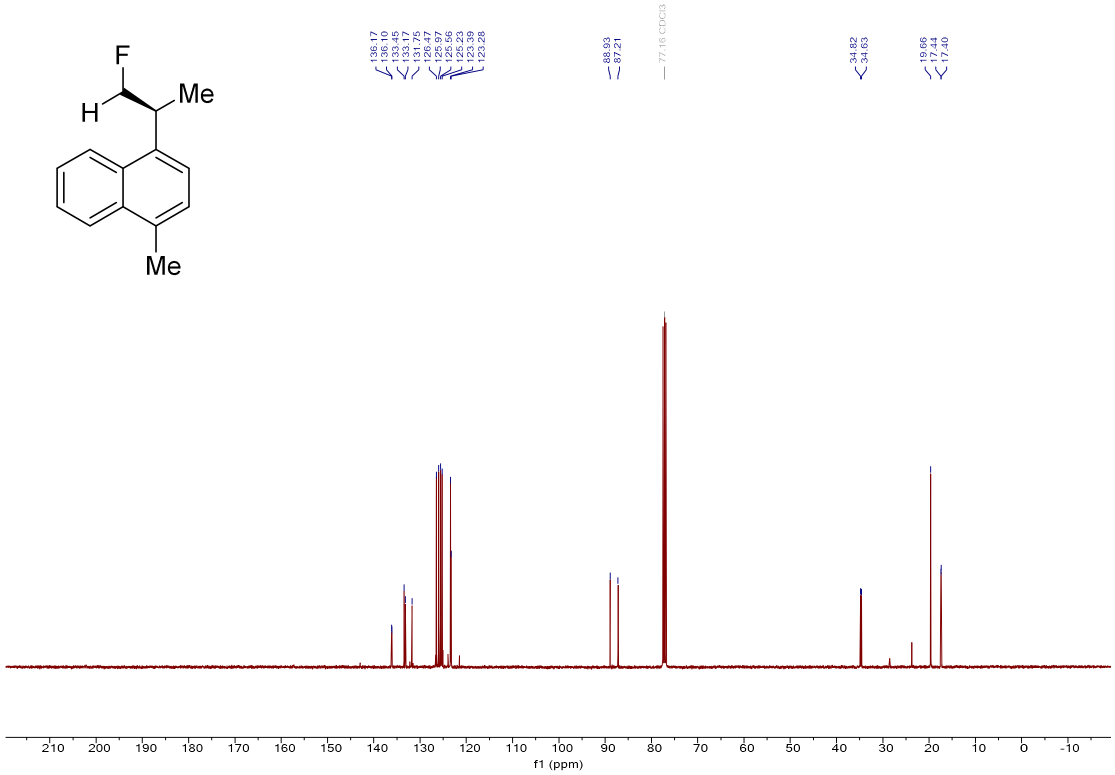
F
H
Me
Me
136.17
136.10
133.45
133.17
131.75
126.47
125.97
125.56
125.23
123.39
123.28
88.93
87.21
77.16 CDCl3
34.82
34.63
19.66
17.44
17.40
210 200 190 180 170 160 150 140 130 120 110 100 90 80 70 60 50 40 30 20 10 0 -10
f1 (ppm)

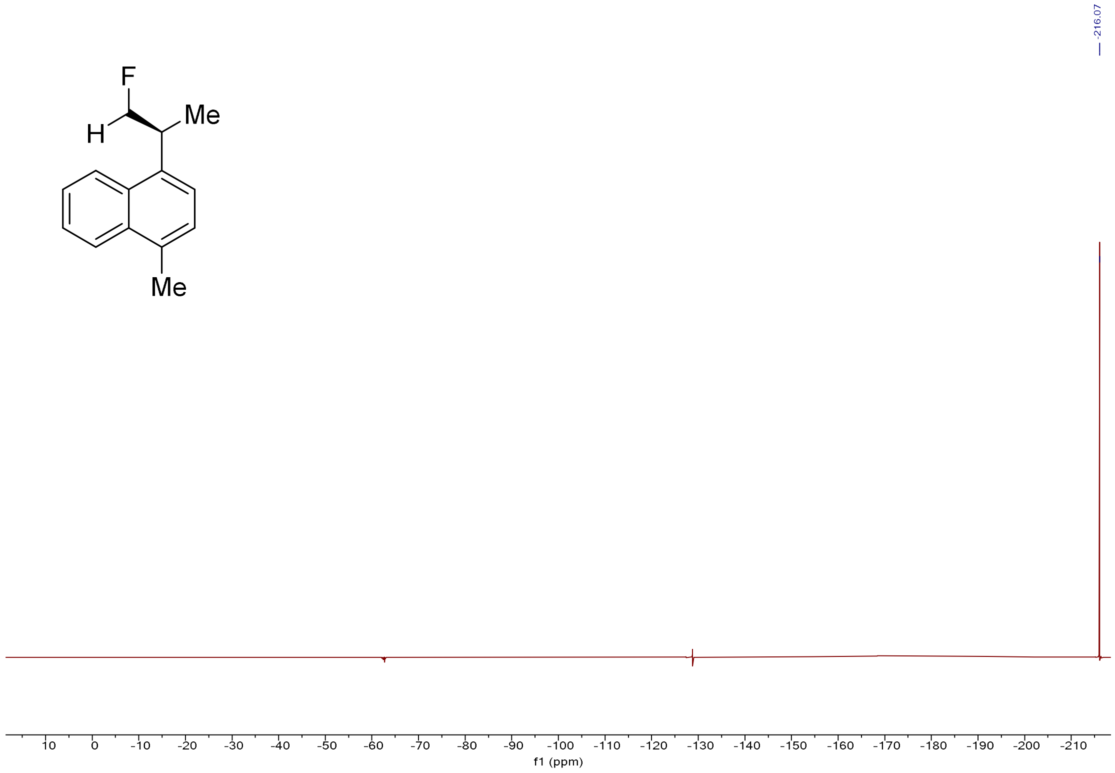
-216.07
F
H
Me
Me
10 0 -10 -20 -30 -40 -50 -60 -70 -80 -90 -100 -110 -120 -130 -140 -150 -160 -170 -180 -190 -200 -210
f1 (ppm)

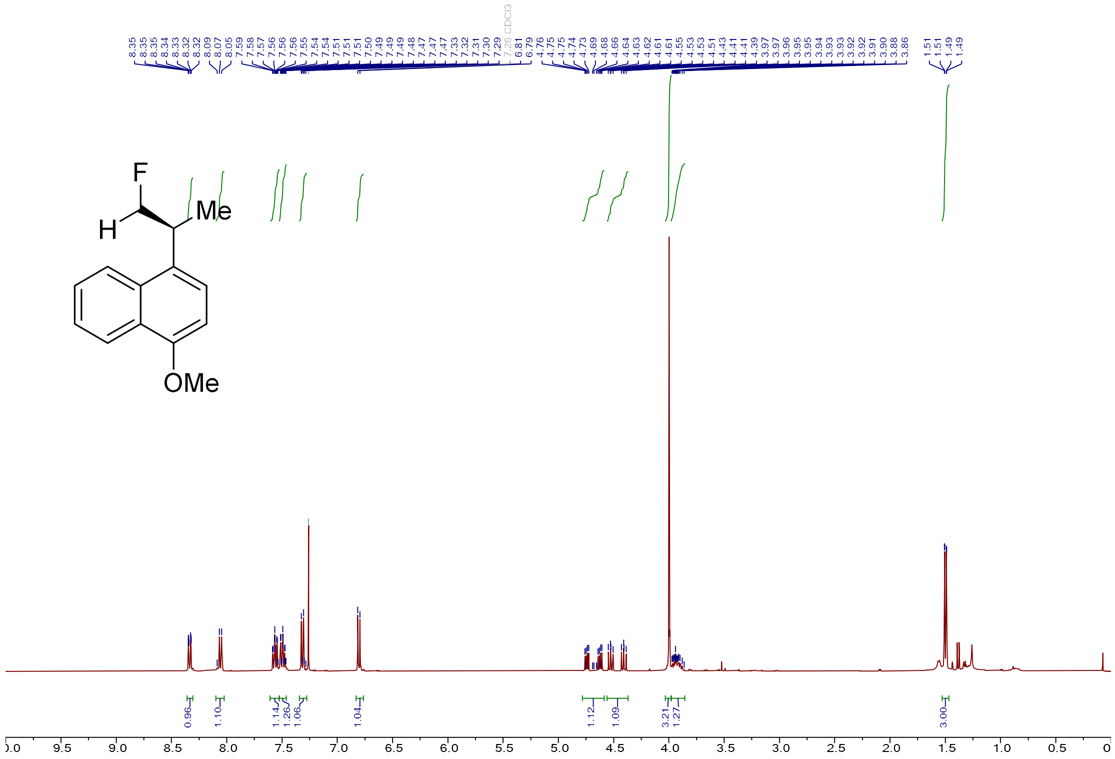

YJ-20201108-7-56-6p.12.fid

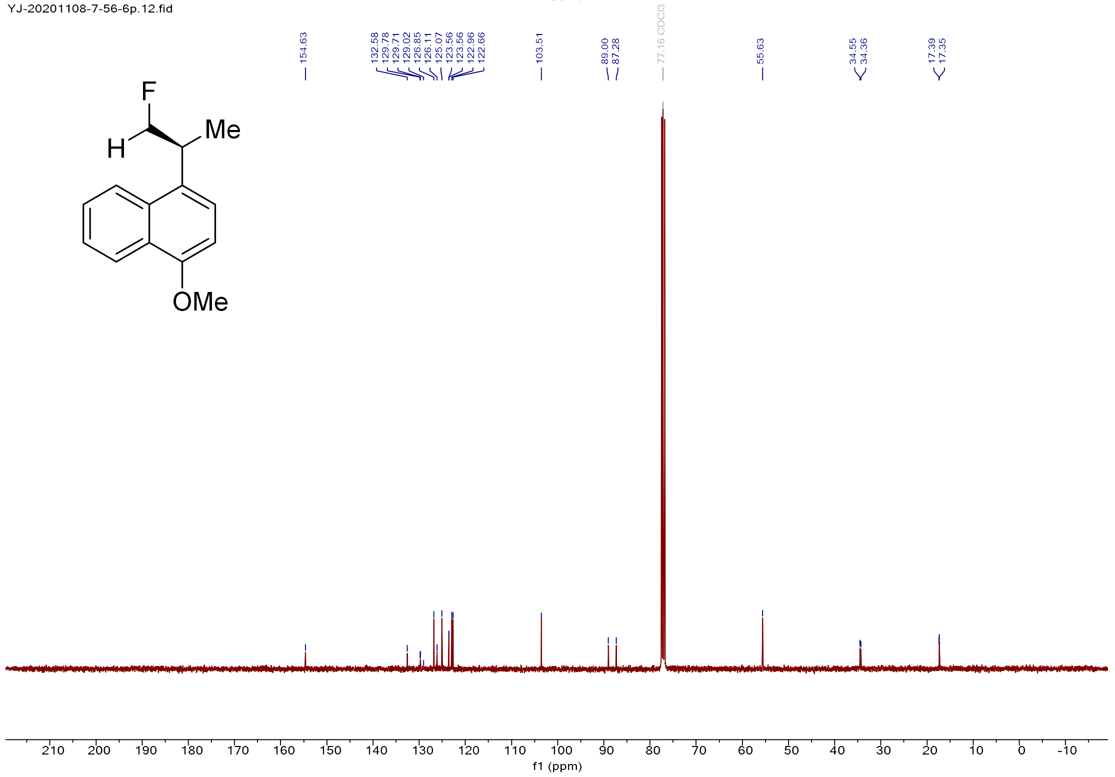

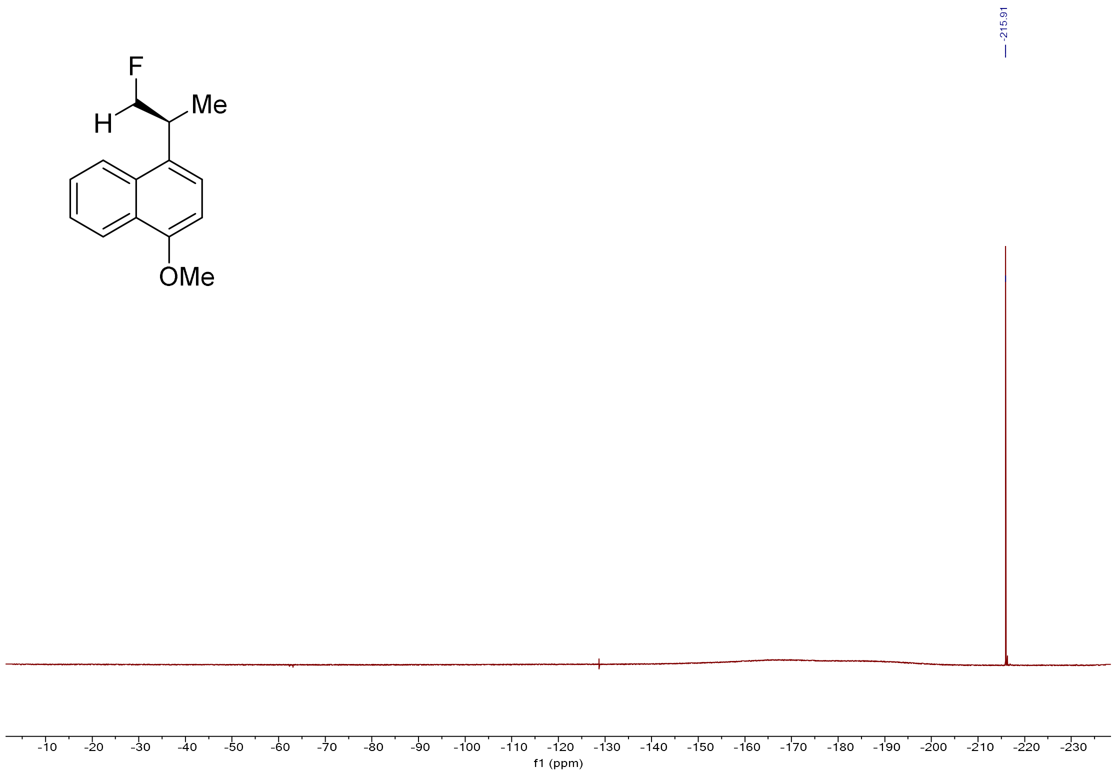

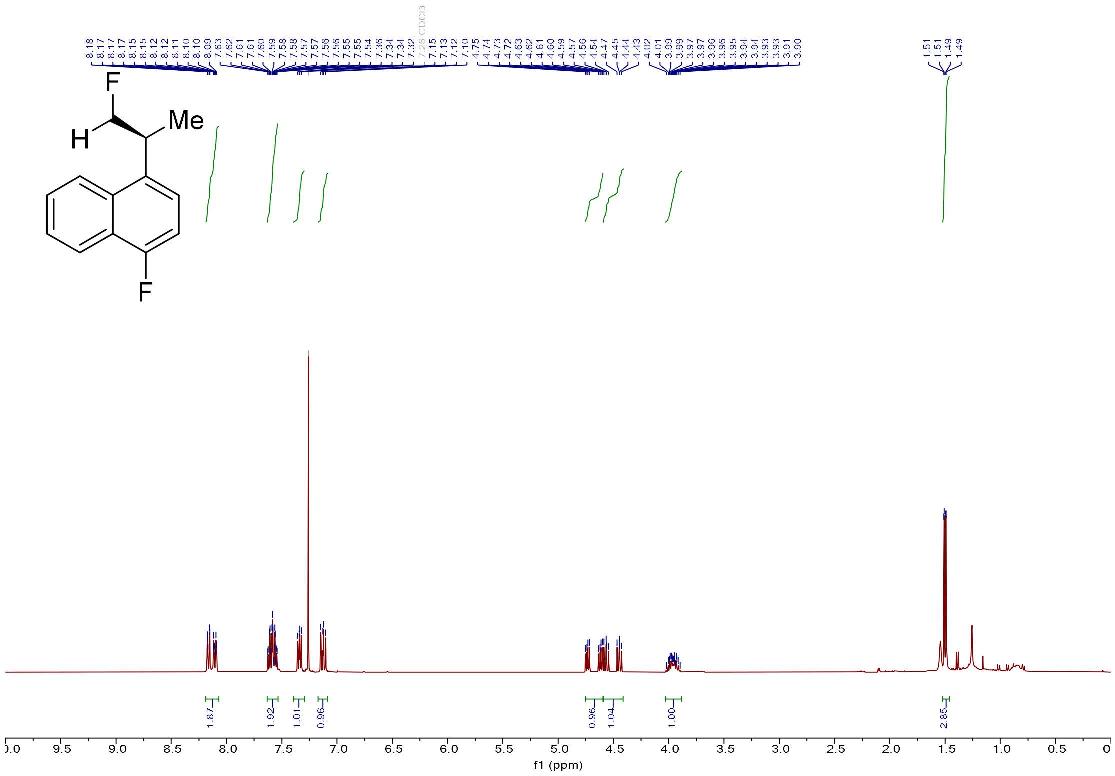

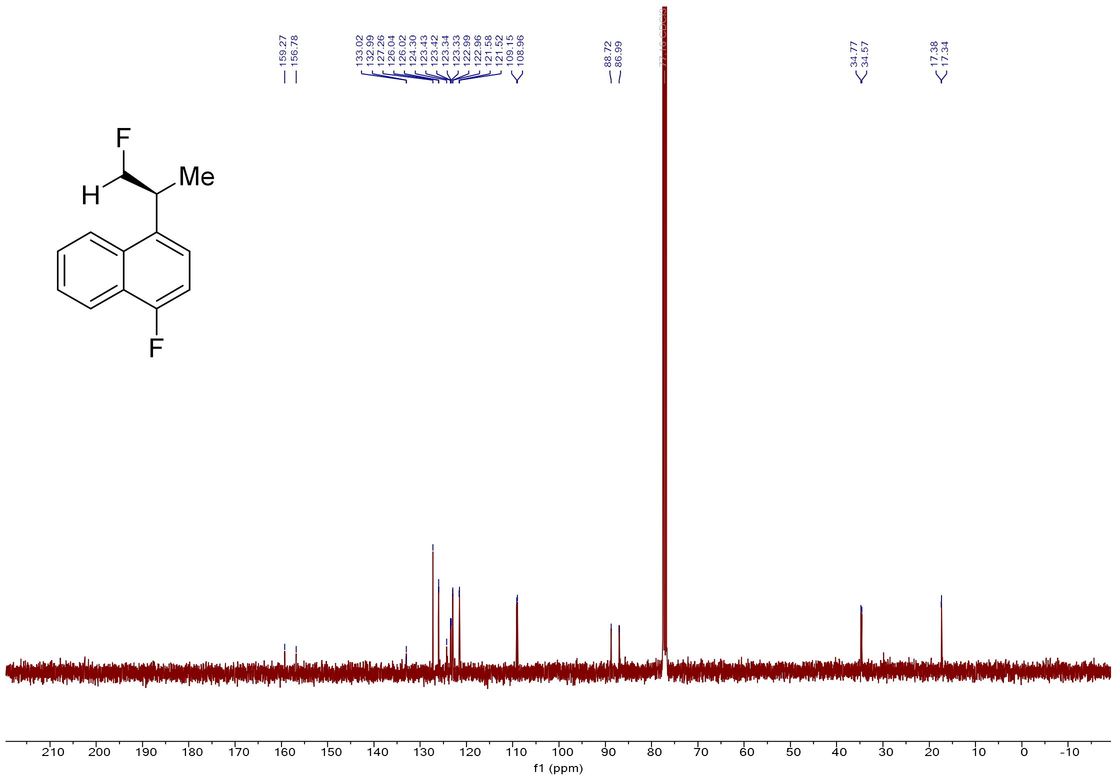

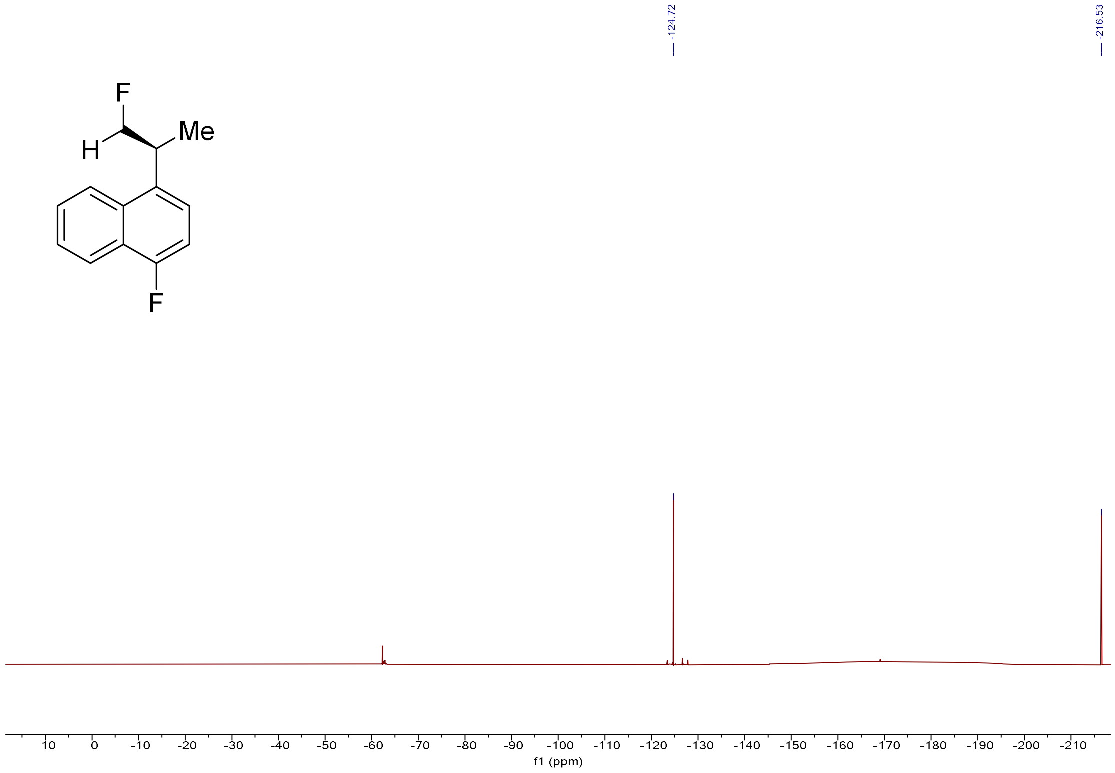

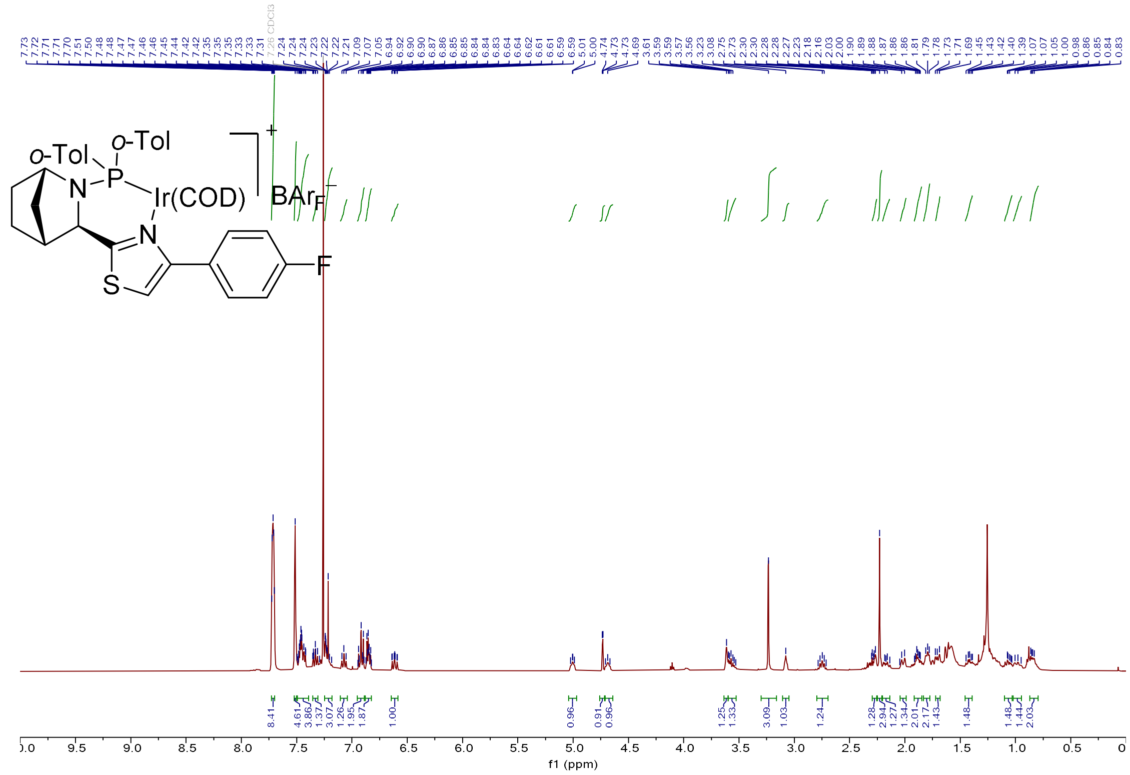
o-Tol
o-Tol
N
P
Ir(COD)
BArF−
N
S
F
f1 (ppm)

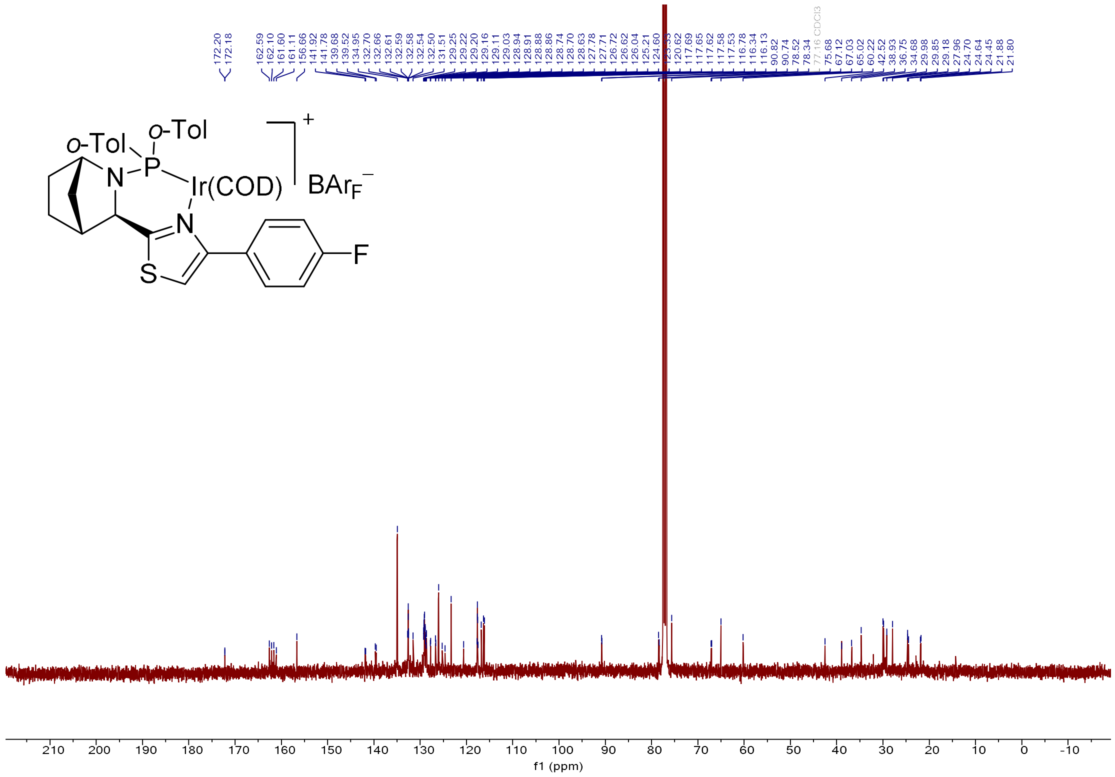
o-Tol
o-Tol
N
P
Ir(COD)
BArF−
N
S
F
f1 (ppm)

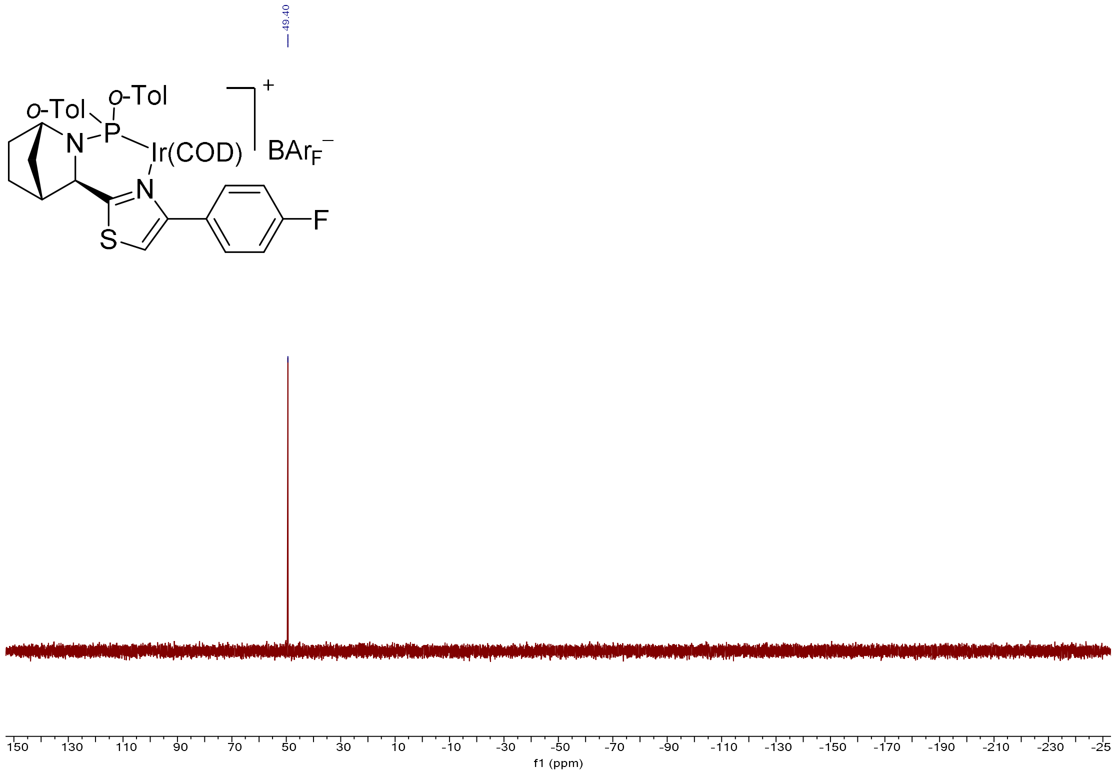
49.40
o-Tol
o-Tol
N
P
Ir(COD)
BArF
N
S
F
f1 (ppm)

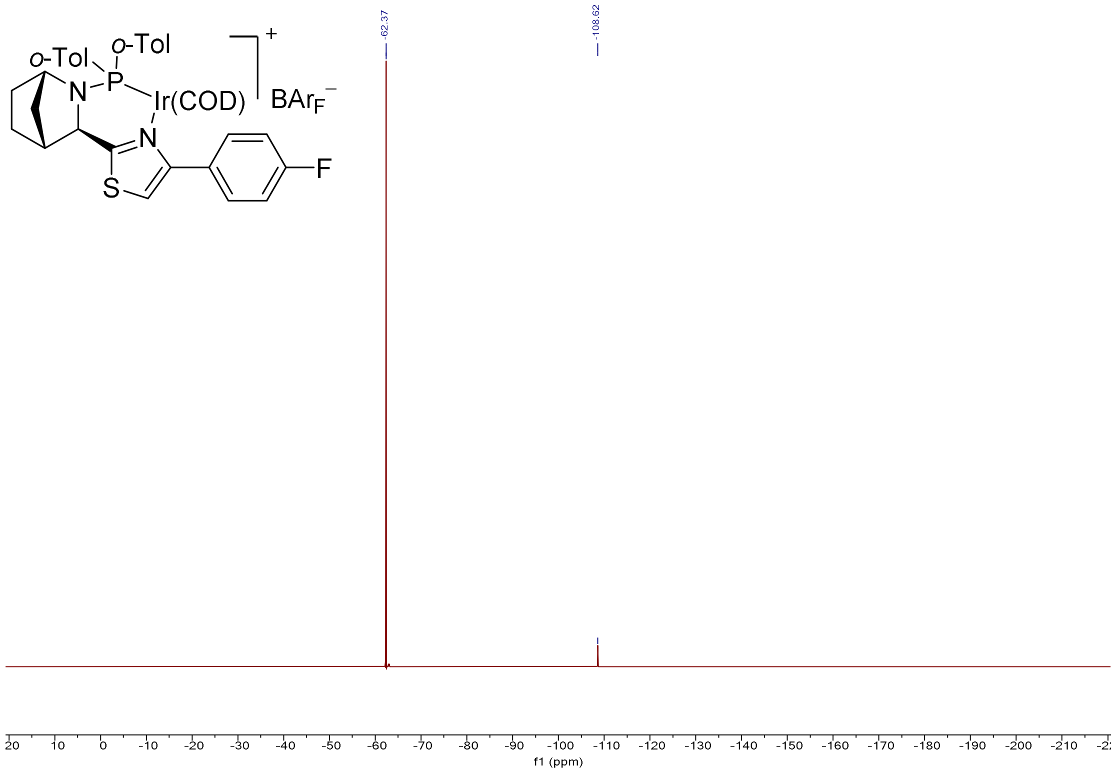
-62.37
-108.62
o-Tol
o-Tol
N
P
Ir(COD)
BArF
N
S
F
f1 (ppm)

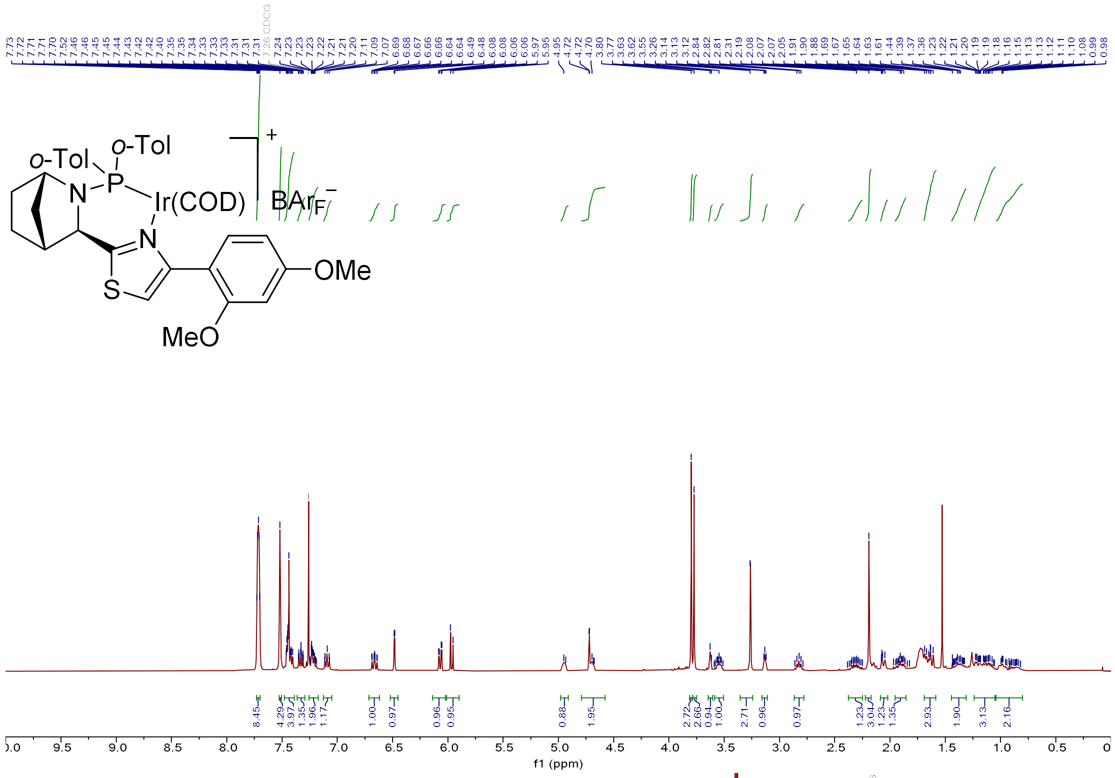

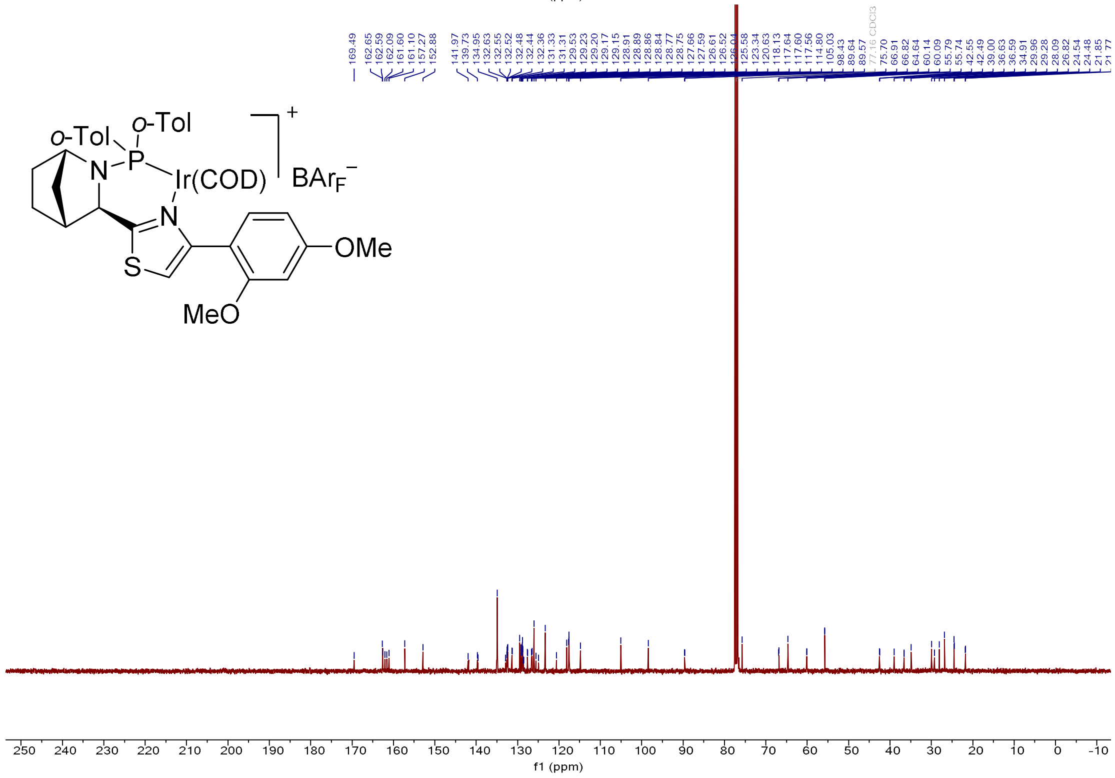

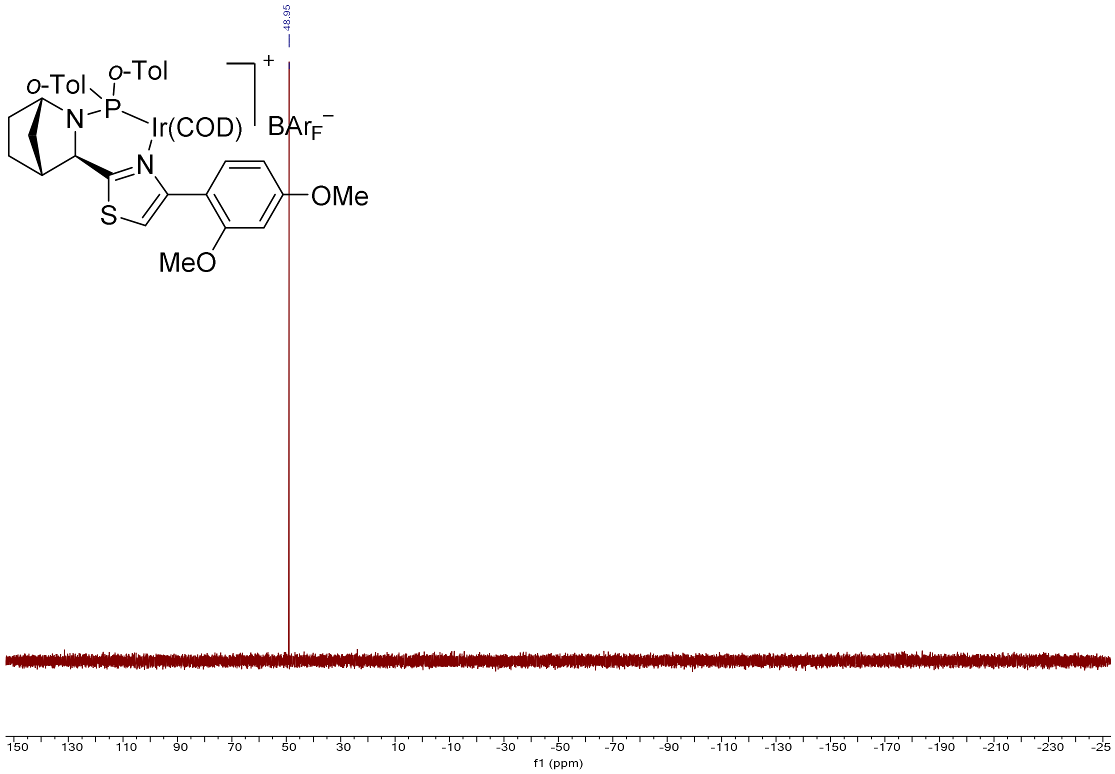
48.95
o-Tol
o-Tol
N
P
Ir(COD)
+
BArF−
N
S
OMe
MeO
150 130 110 90 70 50 30 10 -10 -30 -50 -70 -90 -110 -130 -150 -170 -190 -210 -230
f1 (ppm)

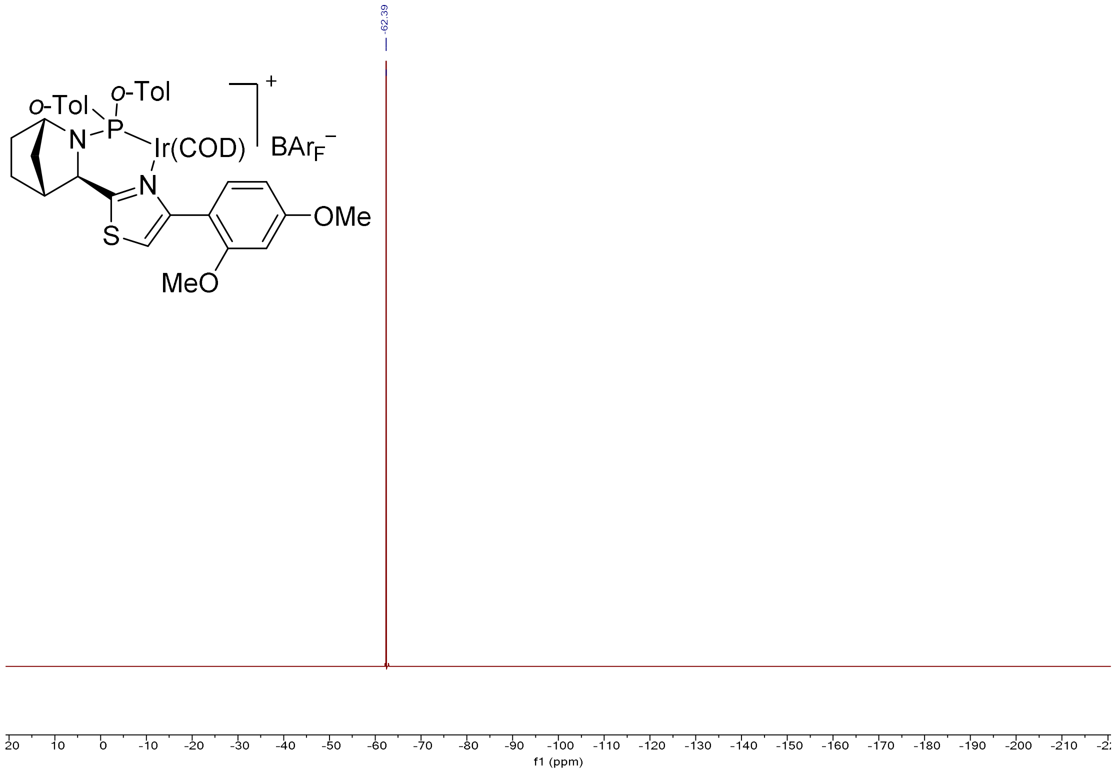
-62.39
o-Tol
o-Tol
N
P
Ir(COD)
+
BArF−
N
S
OMe
MeO
20 10 0 -10 -20 -30 -40 -50 -60 -70 -80 -90 -100 -110 -120 -130 -140 -150 -160 -170 -180 -190 -200 -210
f1 (ppm)

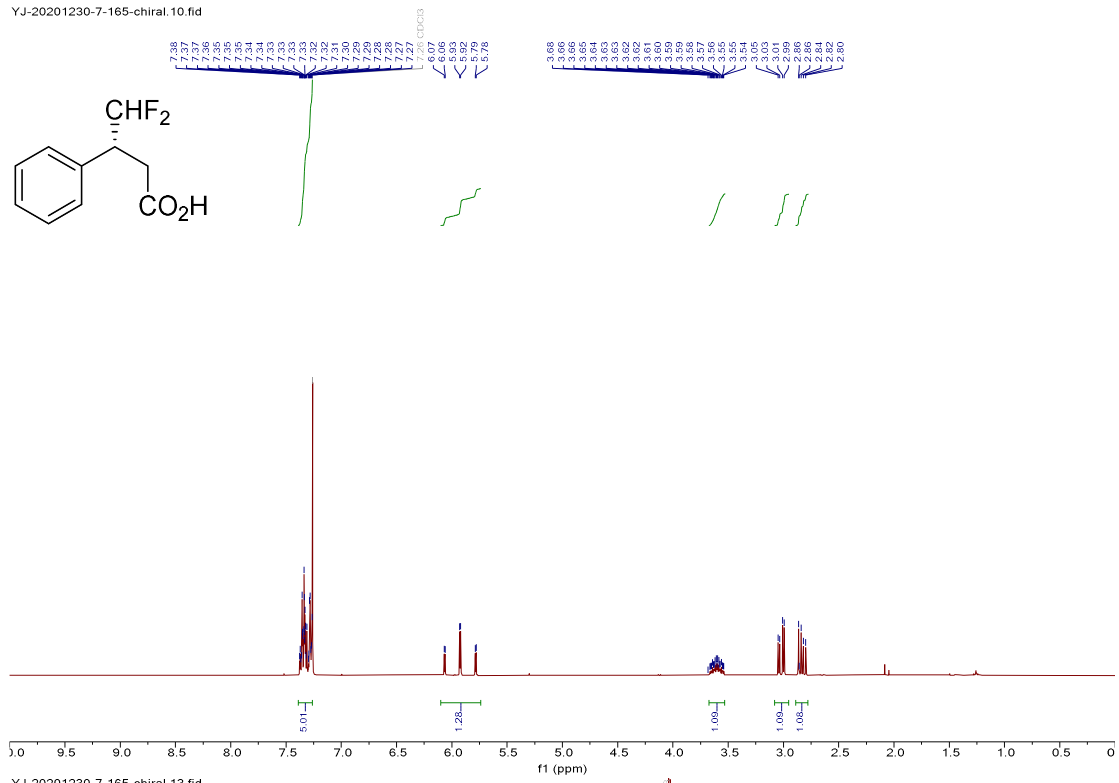
YJ-20201230-7-165-chiral.10.fid
f1 (ppm)

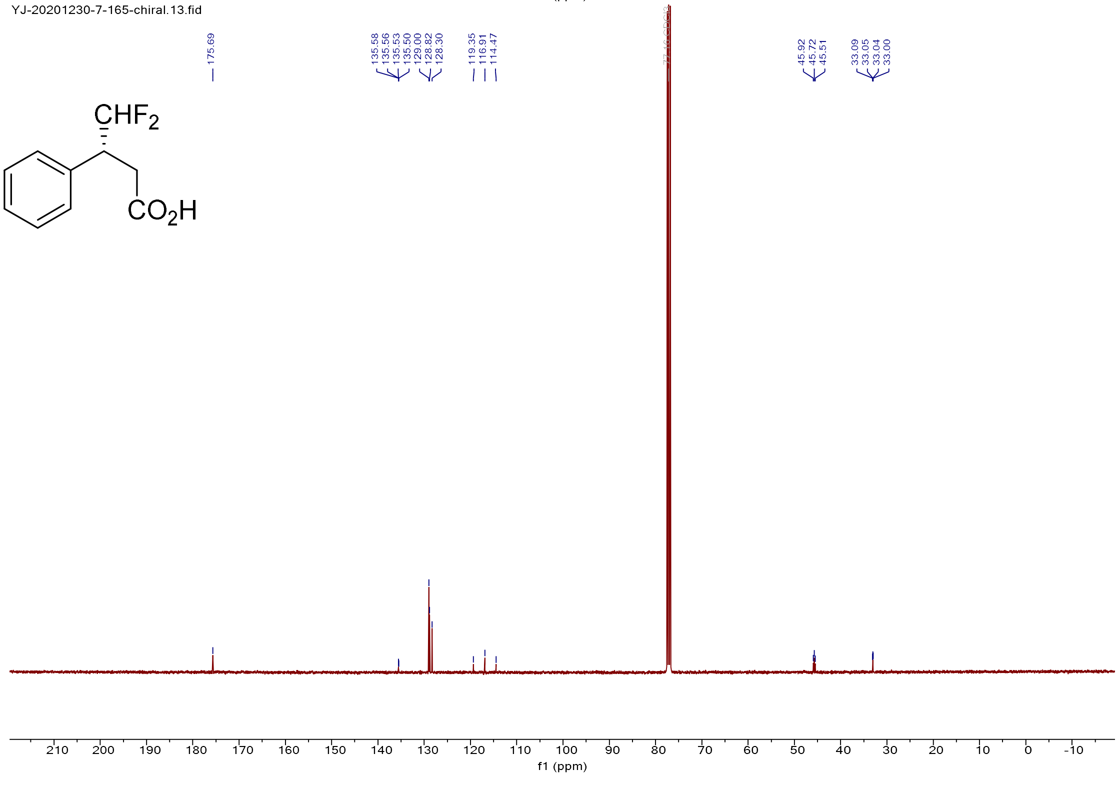
YJ-20201230-7-165-chiral.13.fid
f1 (ppm)

YJ-20201230-7-165-chiral.11.fid

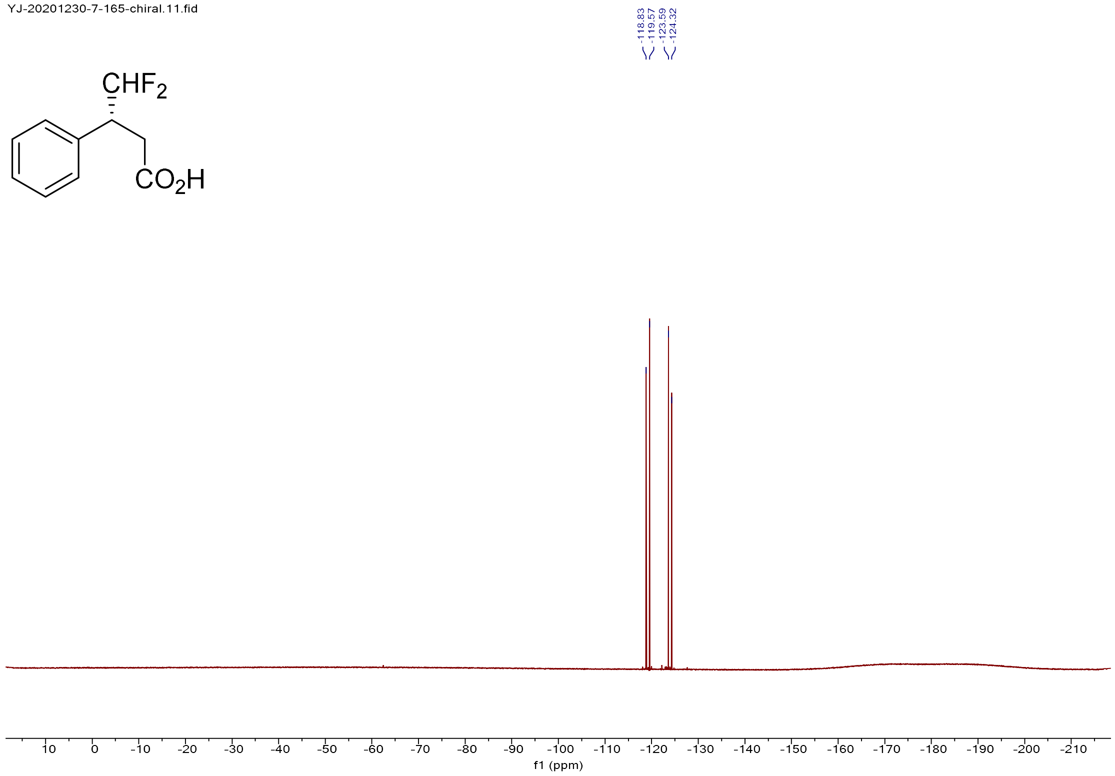

YJ-20210102-7-169-rac.10.fid

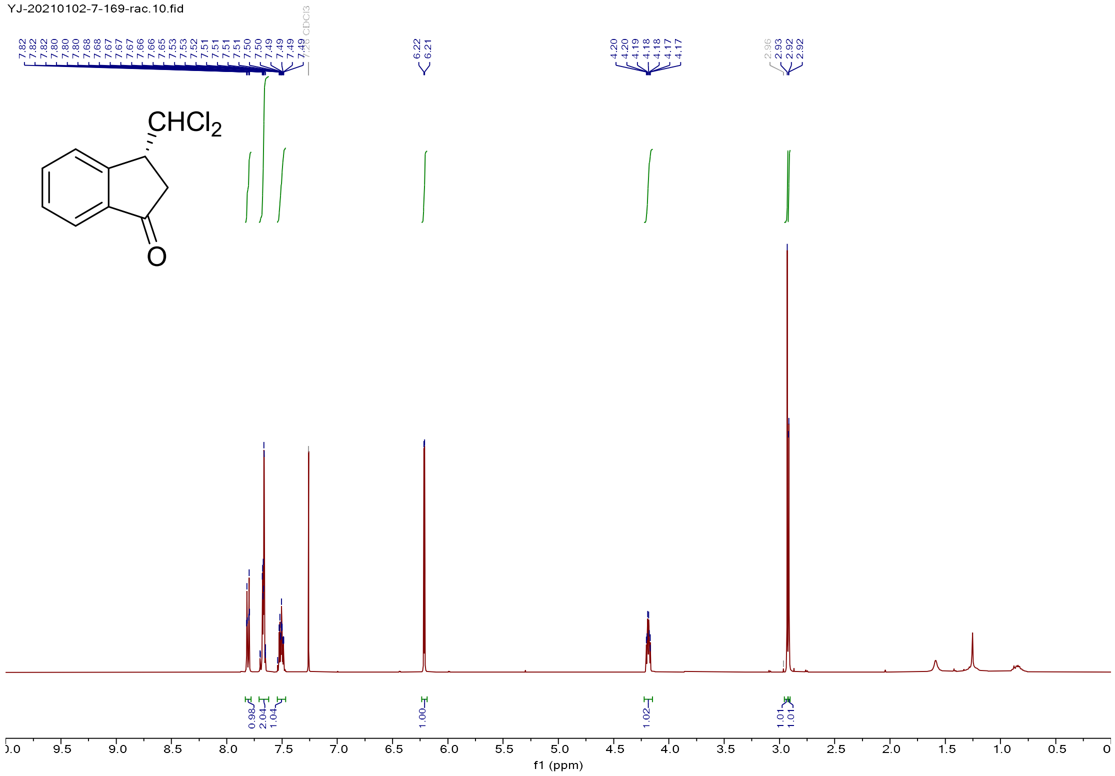

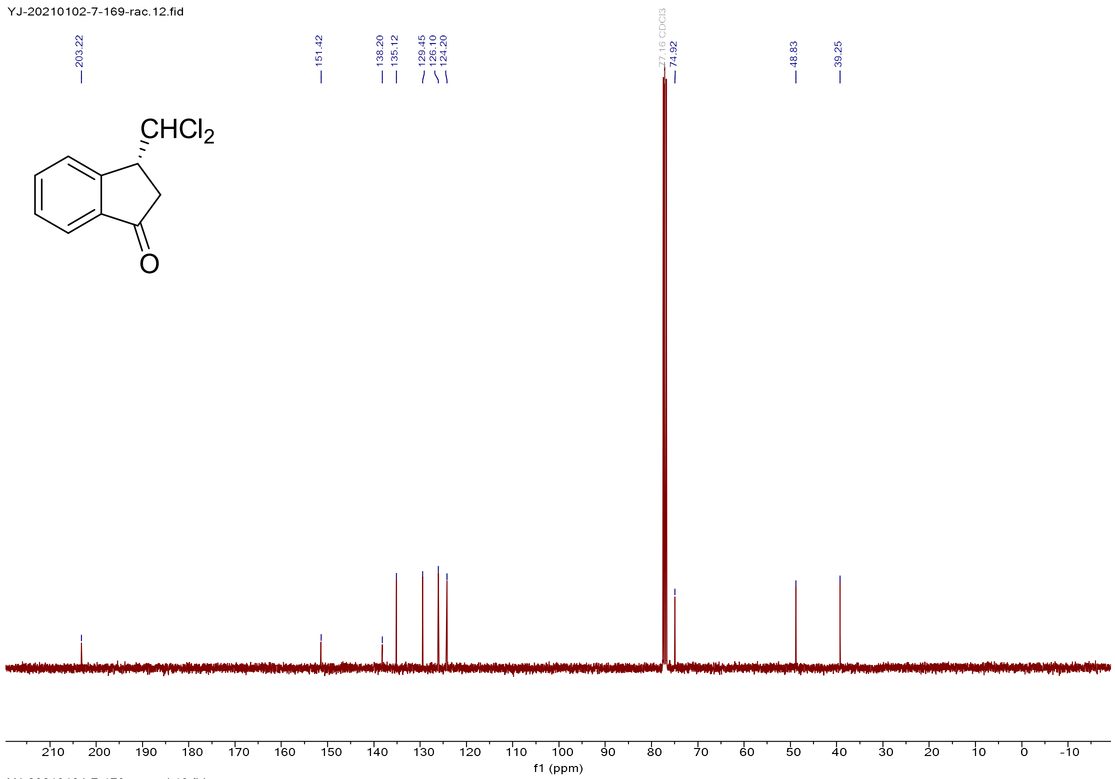
YJ-20210102-7-169-rac.12.fid
203.22
151.42
138.20
135.12
129.45
126.10
124.20
77.16 CDCl3
74.92
48.83
39.25
CHCl2
O
210 200 190 180 170 160 150 140 130 120 110 100 90 80 70 60 50 40 30 20 10 0 -10
f1 (ppm)

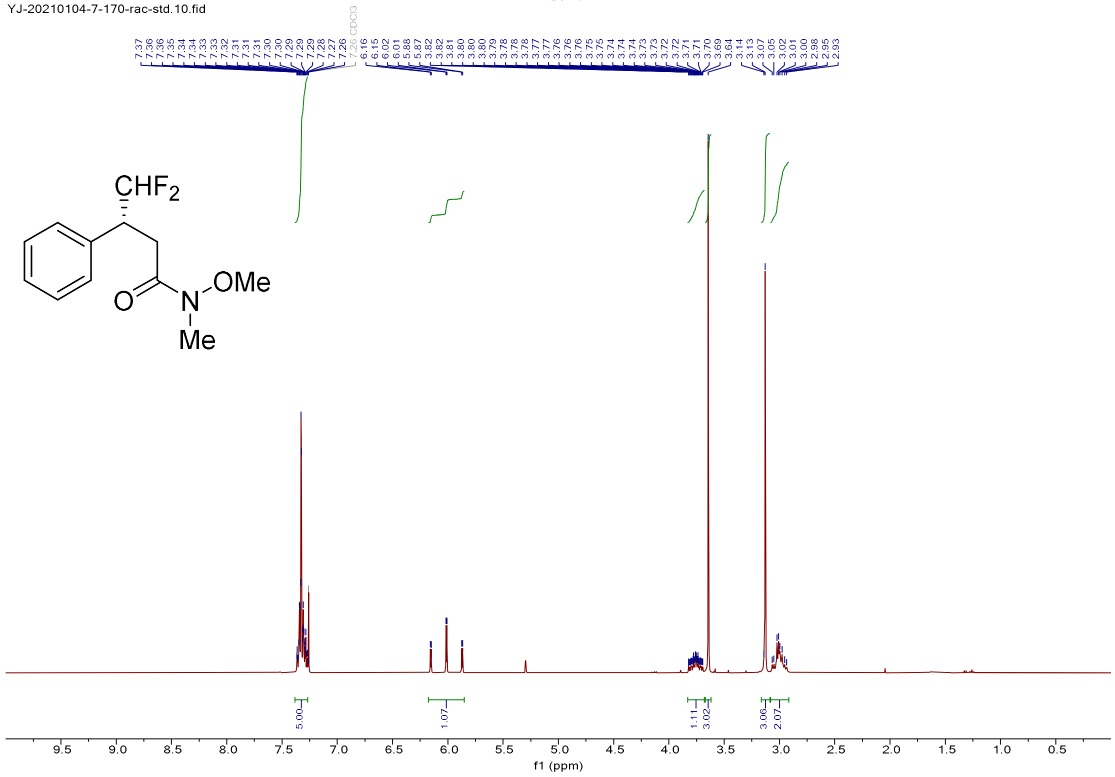
YJ-20210104-7-170-rac-std.10.fid
7.37 7.36 7.35 7.34 7.34 7.33 7.32 7.31 7.31 7.30 7.29 7.29 7.28 7.27 7.26 7.26 CDCl3
6.16 6.15 6.02 6.01 5.88 5.87 3.82 3.81 3.80 3.80 3.79 3.78 3.78 3.77 3.76 3.76 3.75 3.75 3.74 3.74 3.73 3.73 3.72 3.71 3.71 3.70 3.69 3.64 3.14 3.13 3.07 3.05 3.02 3.01 3.00 2.98 2.95 2.93
CHF2
OMe
O
N
Me
5.00
1.07
1.11
3.02
3.06
2.07
9.5 9.0 8.5 8.0 7.5 7.0 6.5 6.0 5.5 5.0 4.5 4.0 3.5 3.0 2.5 2.0 1.5 1.0 0.5
f1 (ppm)

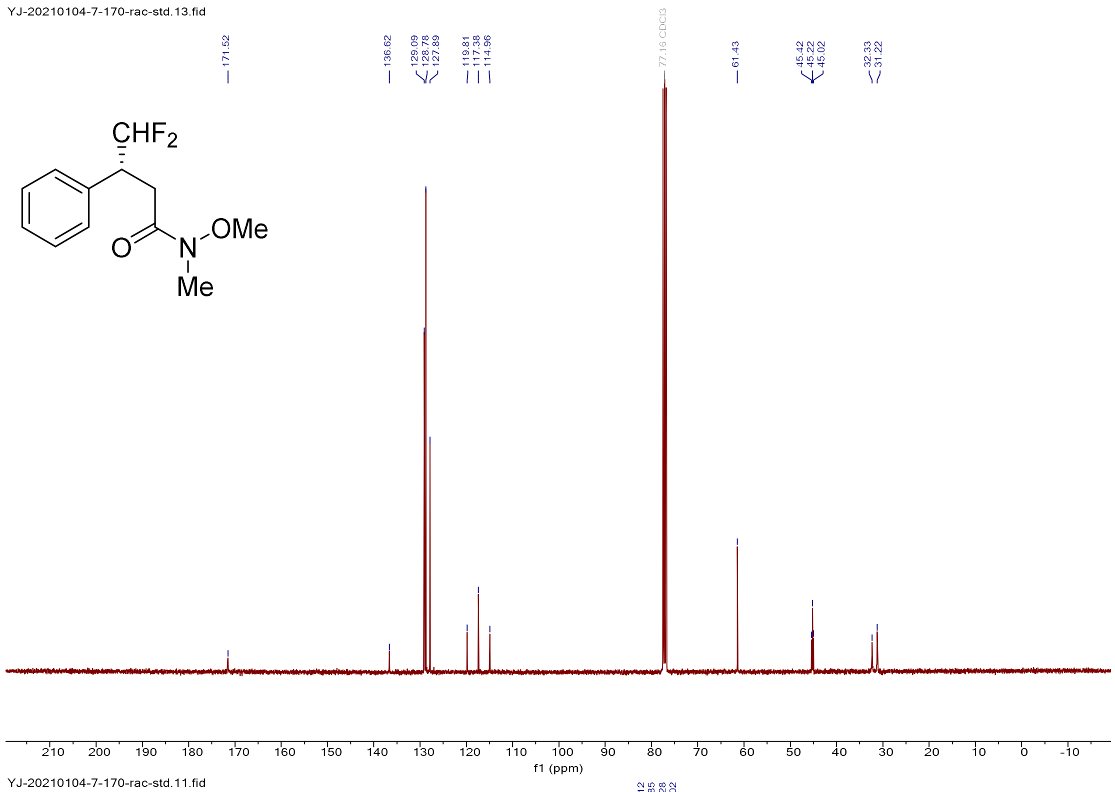
YJ-20210104-7-170-rac-std.13.fid
171.52
136.62
129.09
128.78
127.89
119.81
117.38
114.96
77.16 CDCl3
61.43
45.42
45.22
45.02
32.33
31.22
CHF2
OMe
O
N
Me
210
200
190
180
170
160
150
140
130
120
110
100
90
80
70
60
50
40
30
20
10
0
-10
f1 (ppm)

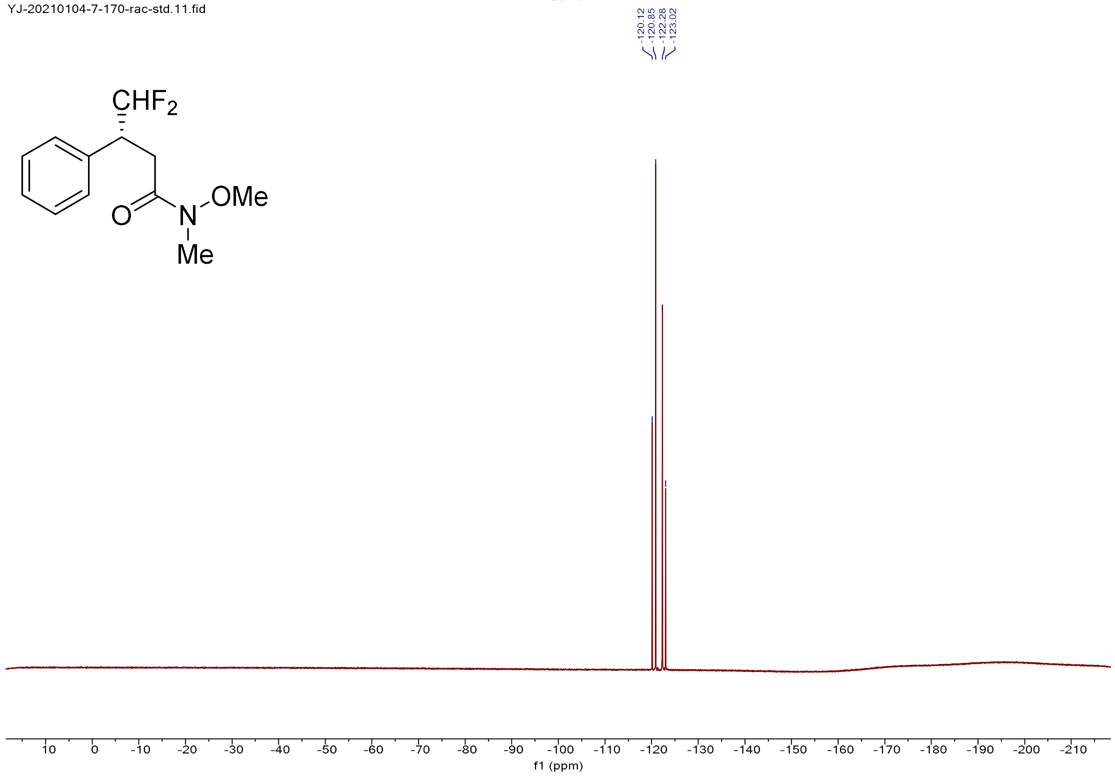
YJ-20210104-7-170-rac-std.11.fid
-120.12
-120.85
-122.28
-123.02
CHF2
OMe
O
N
Me
10
0
-10
-20
-30
-40
-50
-60
-70
-80
-90
-100
-110
-120
-130
-140
-150
-160
-170
-180
-190
-200
-210
f1 (ppm)

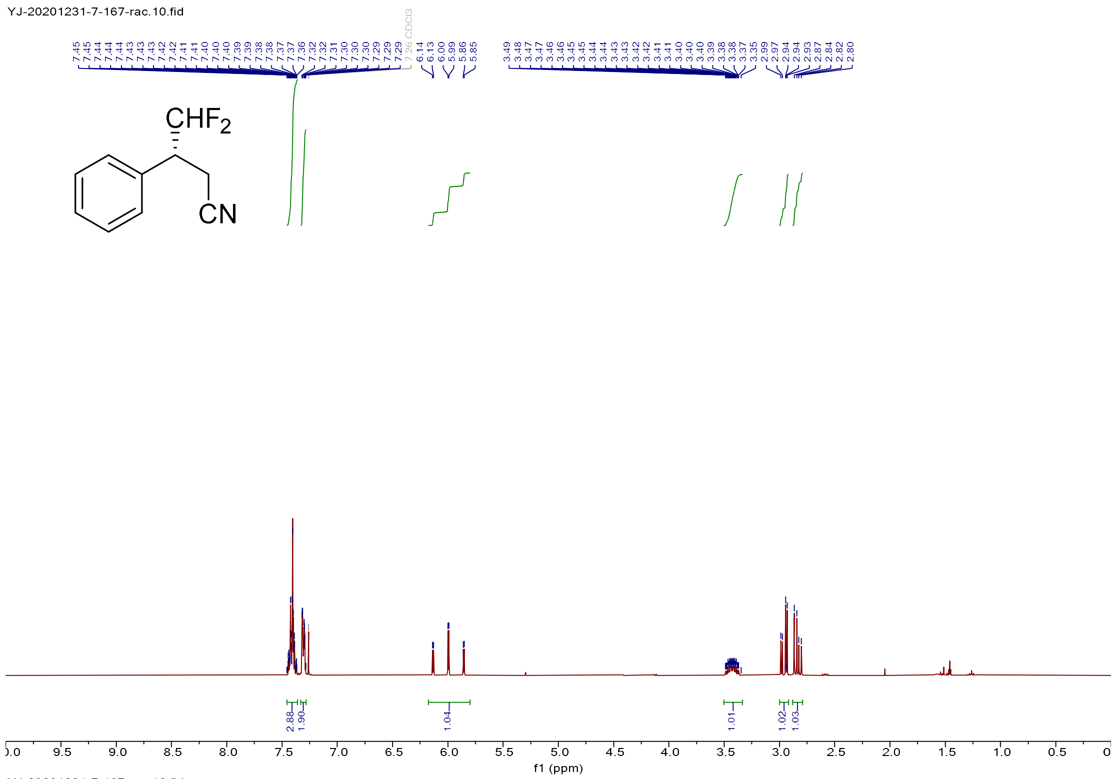

YJ-20201231-7-167-rac.10.fid
7.45 7.45 7.44 7.44 7.43 7.43 7.42 7.41 7.40 7.40 7.39 7.39 7.38 7.37 7.37 7.36 7.32 7.31 7.30 7.30 7.29 7.29
7.26 CDCl3
6.14 6.13 6.00 5.99 5.86 5.85
3.49 3.48 3.47 3.46 3.46 3.45 3.44 3.44 3.43 3.43 3.42 3.41 3.40 3.40 3.39 3.38 3.38 3.37 3.35
2.99 2.97 2.94 2.94 2.93 2.87 2.84 2.82 2.80
CHF2
CN
2.88
1.90
1.04
1.01
1.02
1.03
f1 (ppm)

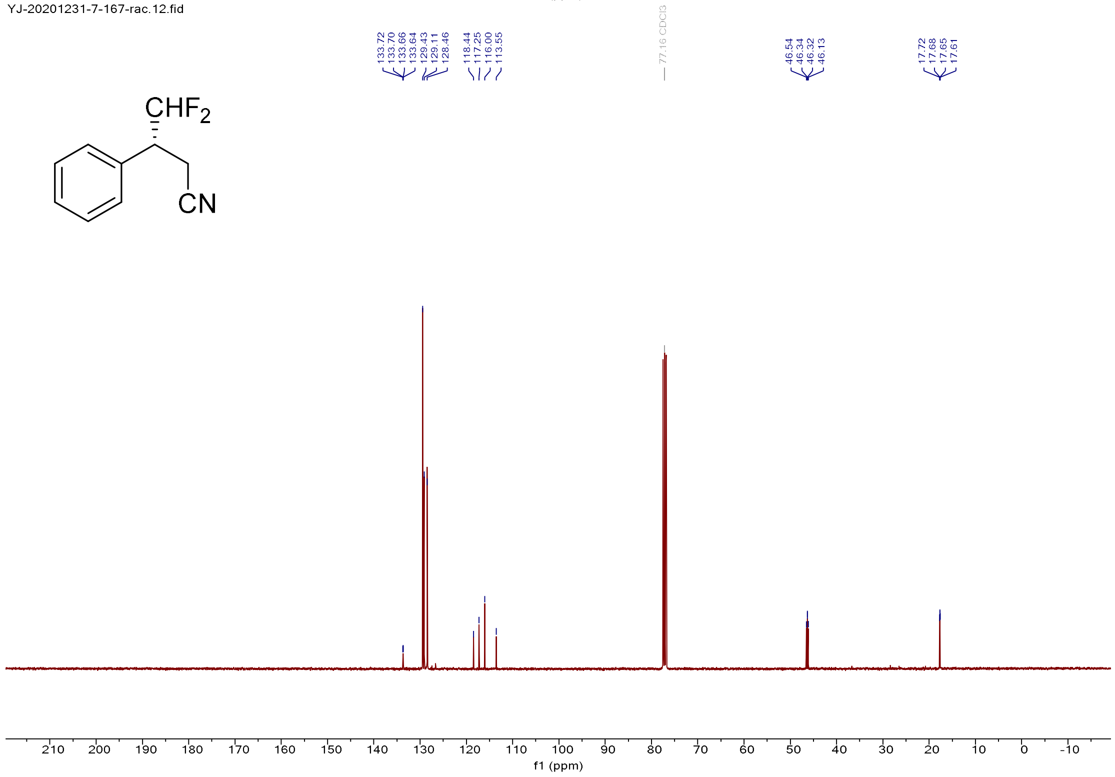

YJ-20201231-7-167-rac.12.fid
133.72 133.70 133.66 133.64 129.43 129.11 128.46
118.44 117.25 116.00 113.55
77.16 CDCl3
46.54 46.34 46.32 46.13
17.72 17.68 17.65 17.61
CHF2
CN
f1 (ppm)

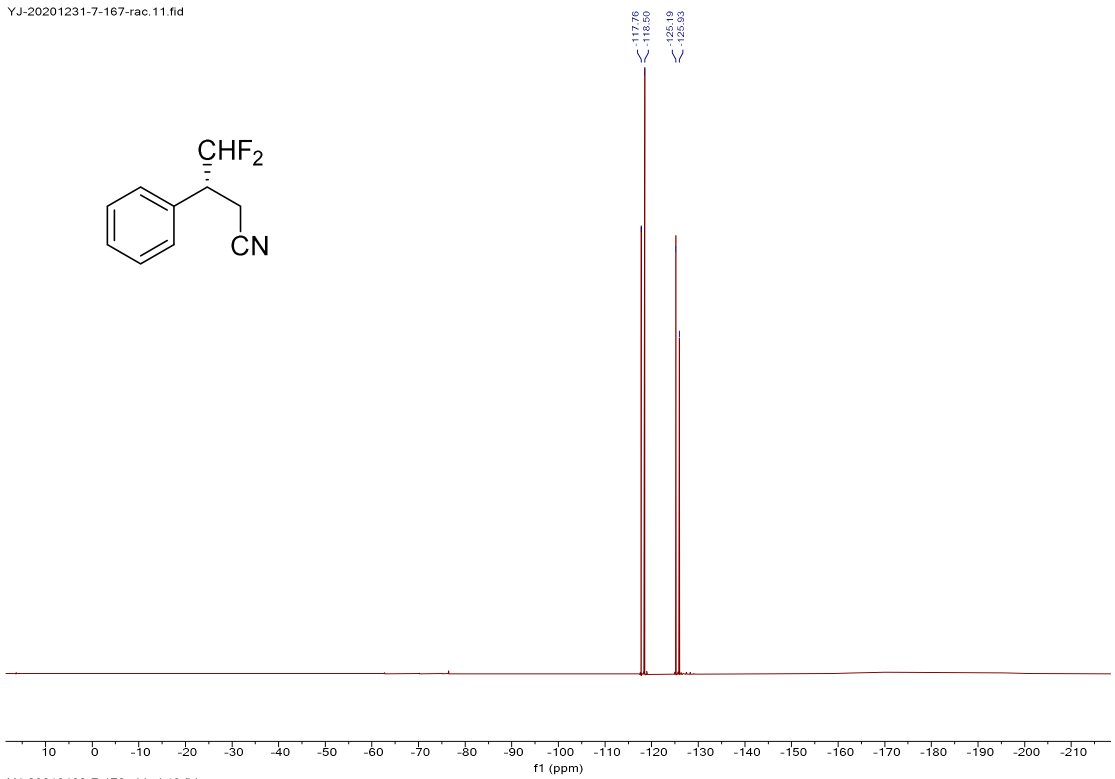
YJ-20201231-7-167-rac.11.fid
-117.76
-118.50
-125.19
-125.93
CHF2
CN
f1 (ppm)

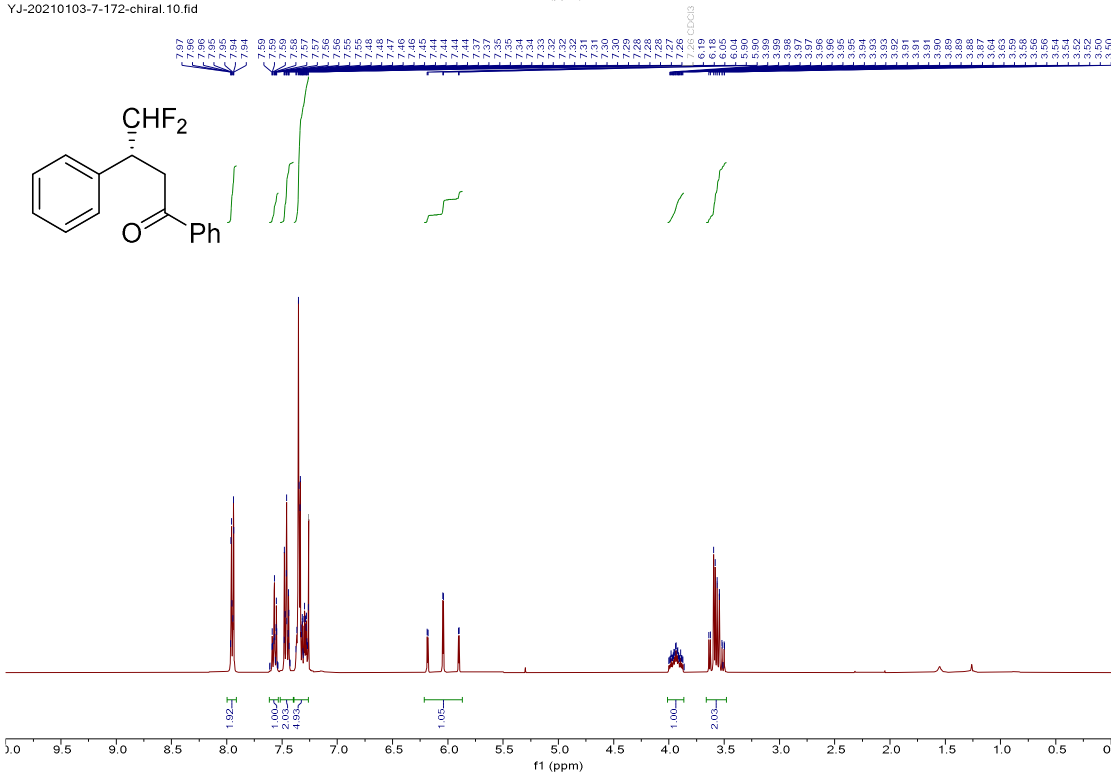
YJ-20210103-7-172-chiral.10.fid
CHF2
O
Ph
7.26 CDCl3
1.92
1.00
2.03
4.93
1.05
1.00
2.03
f1 (ppm)

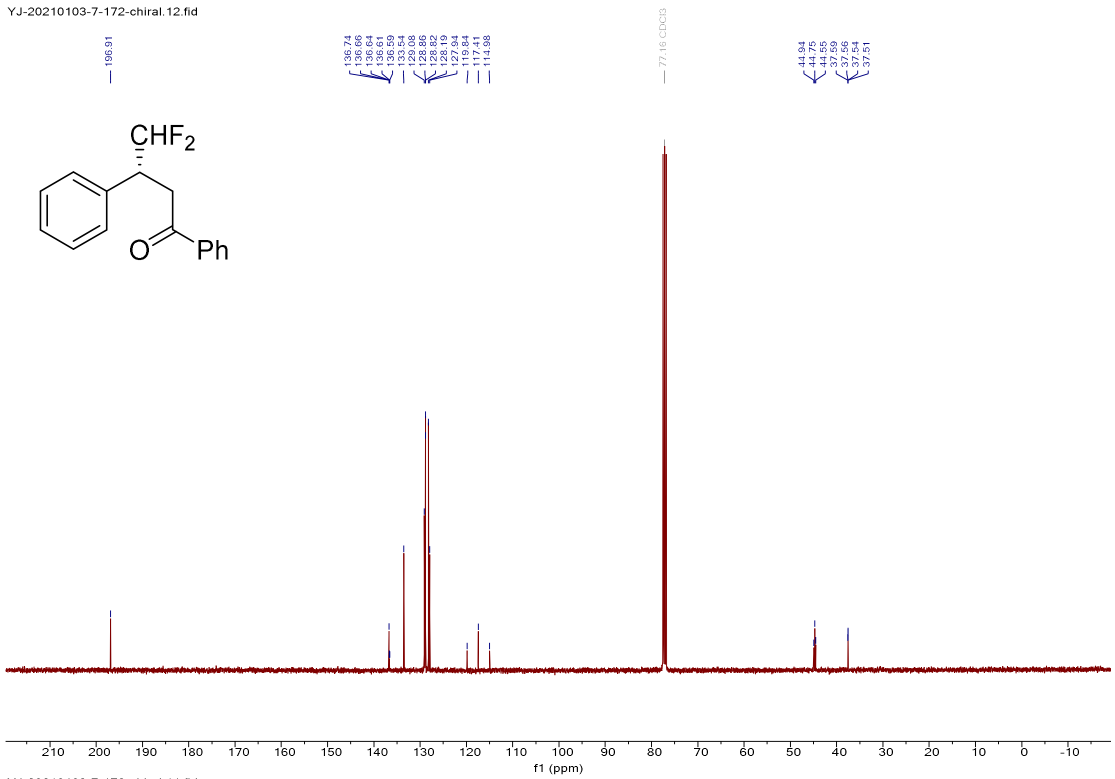
YJ-20210103-7-172-chiral.12.fid
196.91
136.74
136.66
136.64
136.61
136.59
133.54
129.08
128.86
128.82
128.19
127.94
119.84
117.41
114.98
77.16 CDCl3
44.94
44.75
44.55
37.59
37.56
37.54
37.51
CHF2
O
Ph
f1 (ppm)

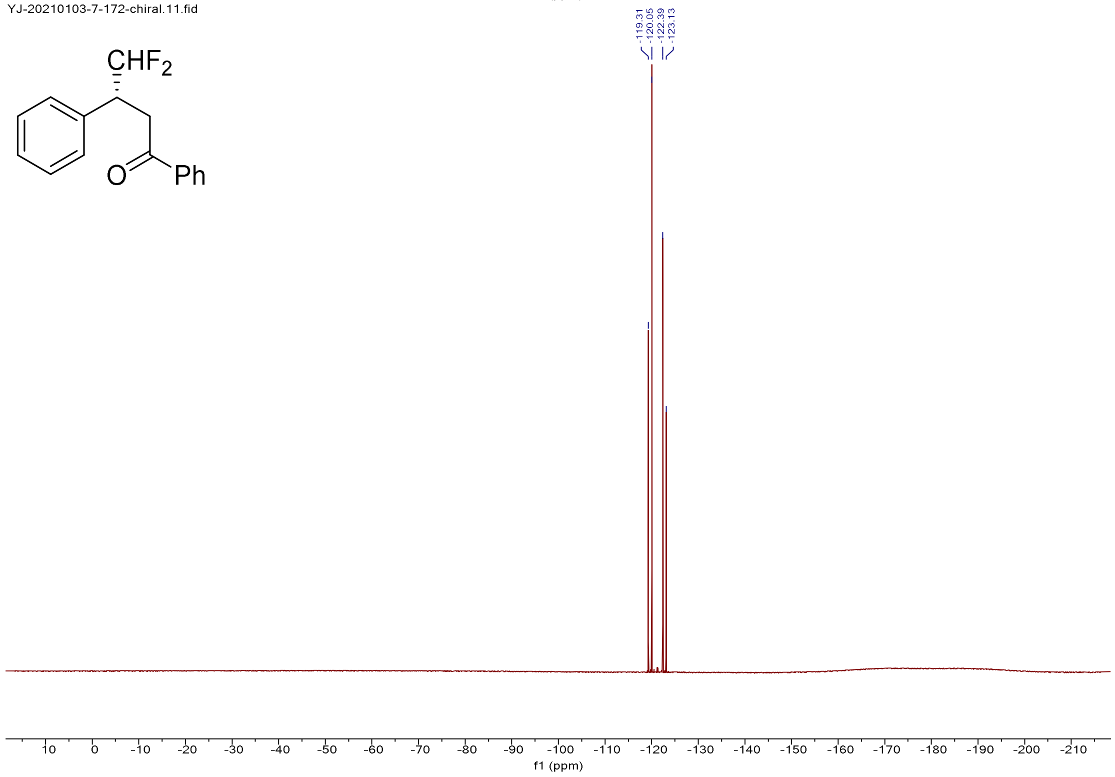
YJ-20210103-7-172-chiral.11.fid
-119.31
-120.05
-122.39
-123.13
CHF2
O
Ph
f1 (ppm)

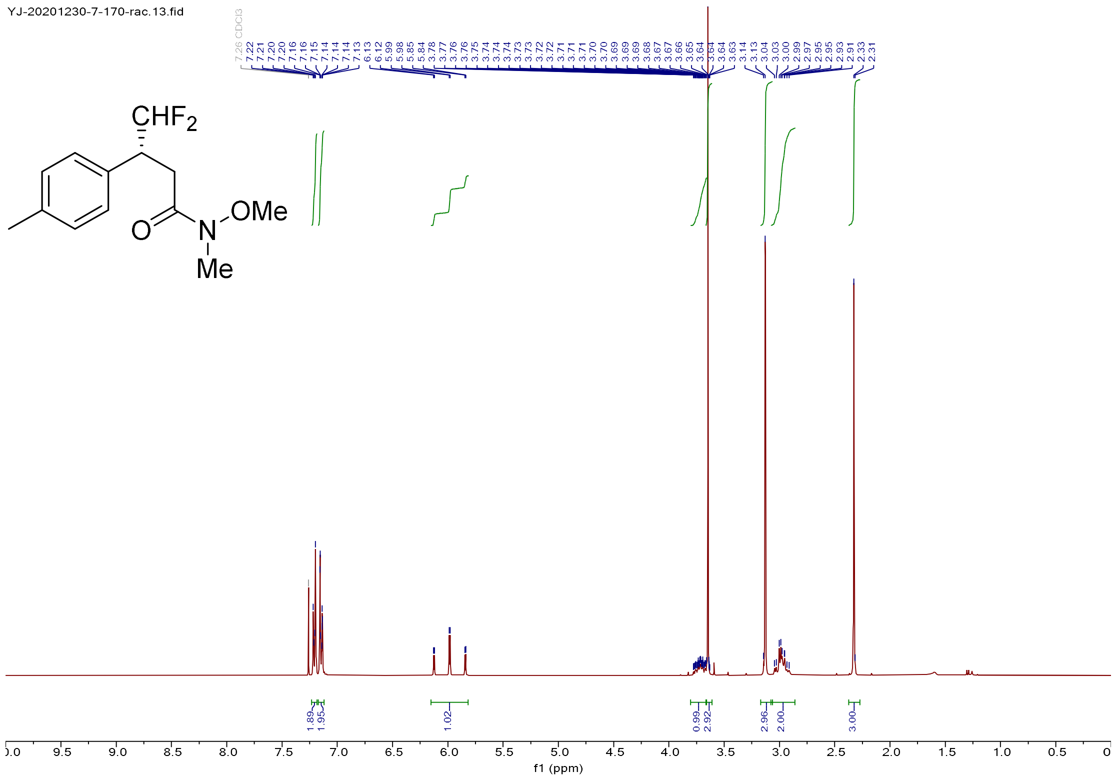
YJ-20201230-7-170-rac.13.fid
CHF2
OMe
N
O
Me
f1 (ppm)

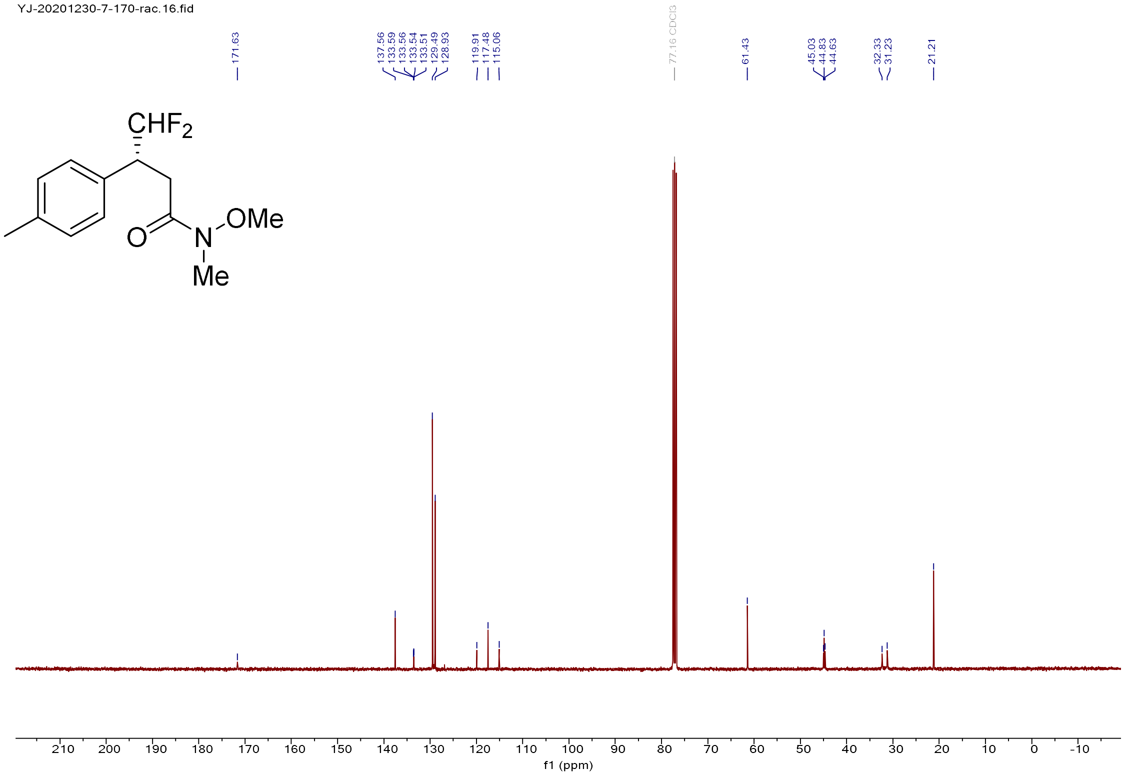
YJ-20201230-7-170-rac.16.fid
171.63
137.56
133.59
133.56
133.54
133.51
129.49
128.93
119.91
117.48
115.06
77.16 CDCl3
61.43
45.03
44.83
44.63
32.33
31.23
21.21
CHF2
OMe
N
O
Me
f1 (ppm)

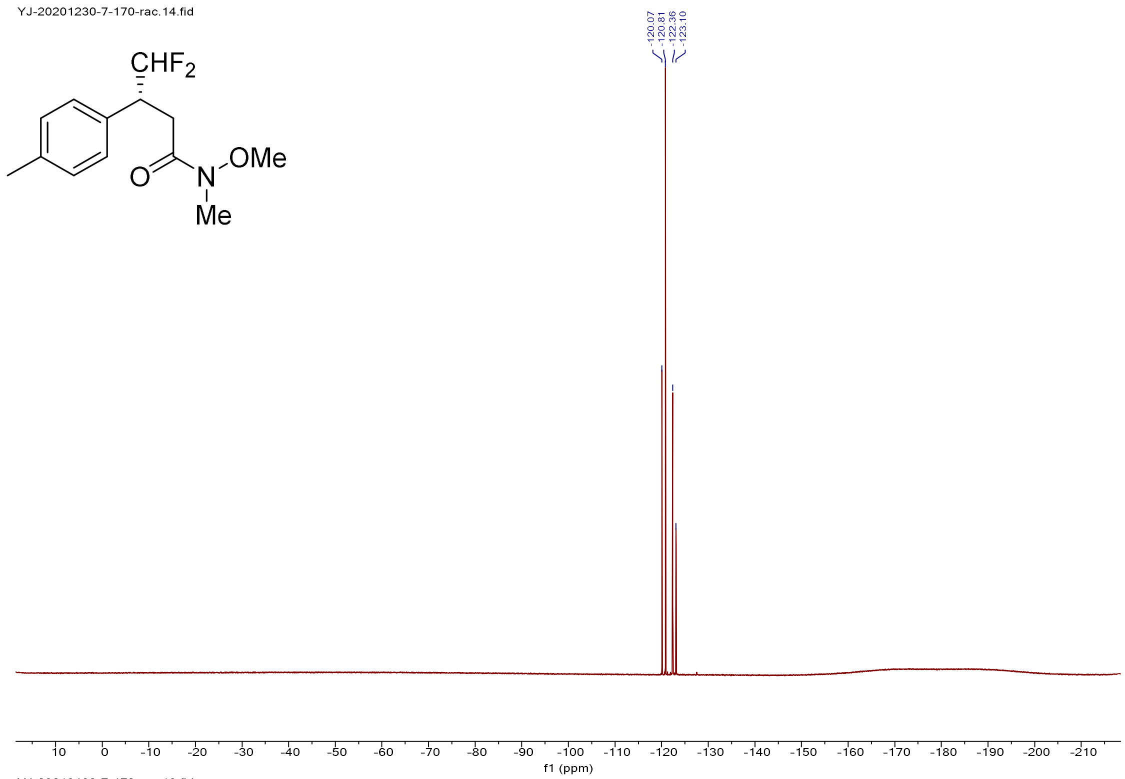

YJ-20201230-7-170-rac.14.fid
CHF2
OMe
N
O
Me
-120.07
-120.81
-122.36
-123.10
f1 (ppm)

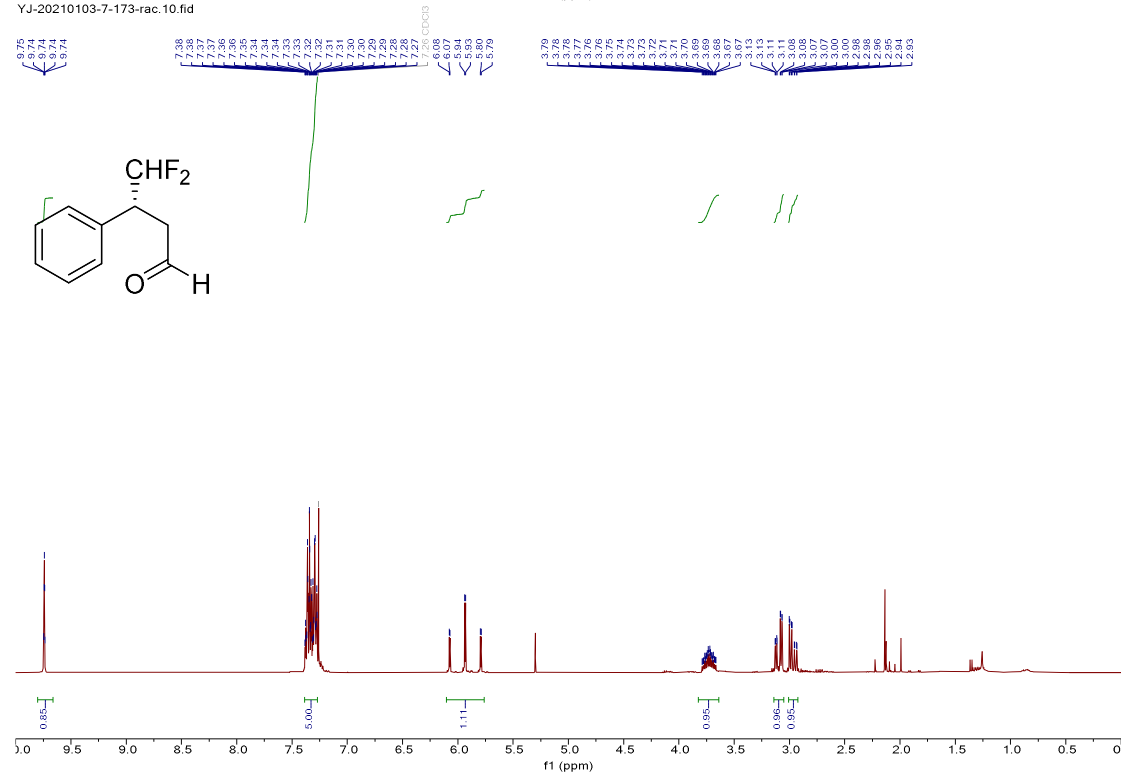

YJ-20210103-7-173-rac.10.fid
CHF2
O
H
0.85
5.00
1.11
0.95
0.96
0.95
f1 (ppm)

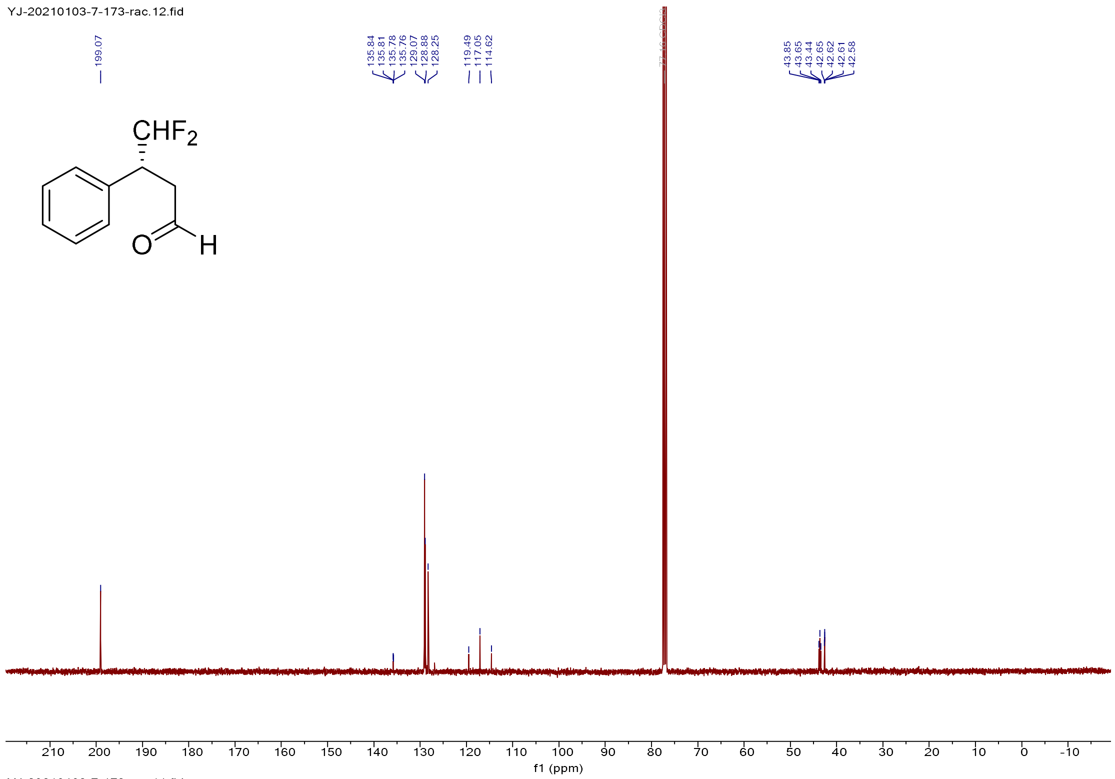
YJ-20210103-7-173-rac.12.fid
199.07
135.84
135.81
135.78
135.76
129.07
128.88
128.25
119.49
117.05
114.62
43.85
43.65
43.44
42.65
42.62
42.61
42.58
CHF2
O
H
f1 (ppm)

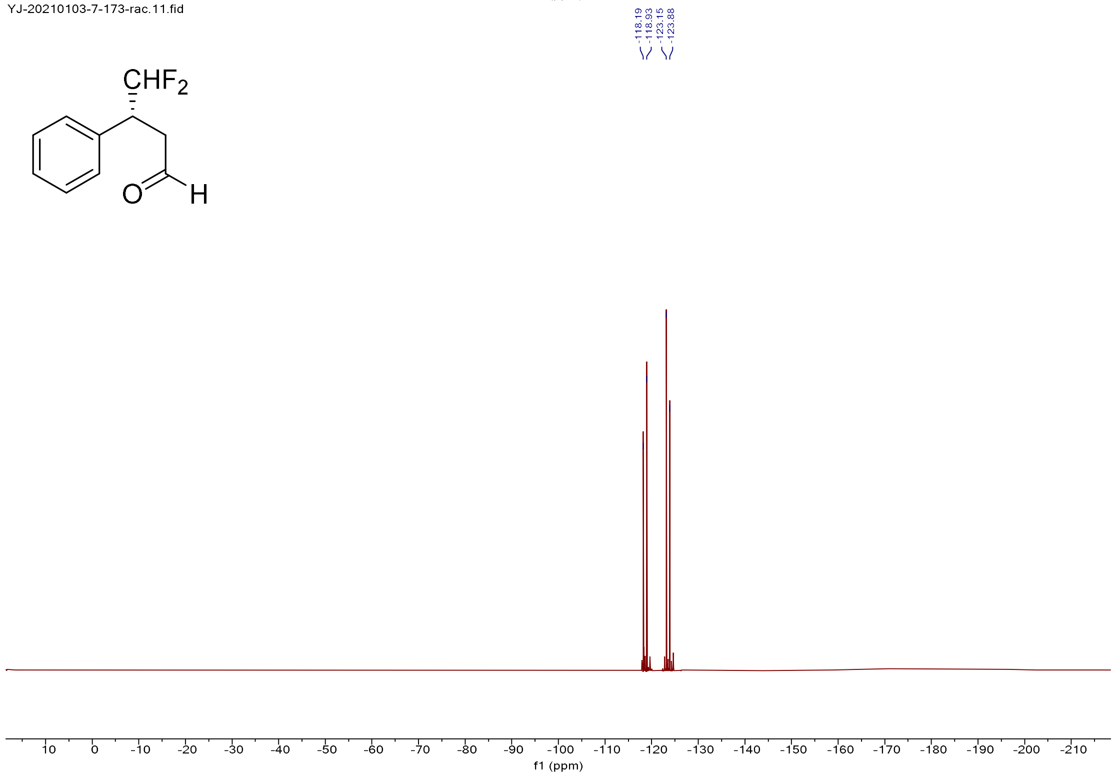
YJ-20210103-7-173-rac.11.fid
-118.19
-118.93
-123.15
-123.88
CHF2
O
H
f1 (ppm)

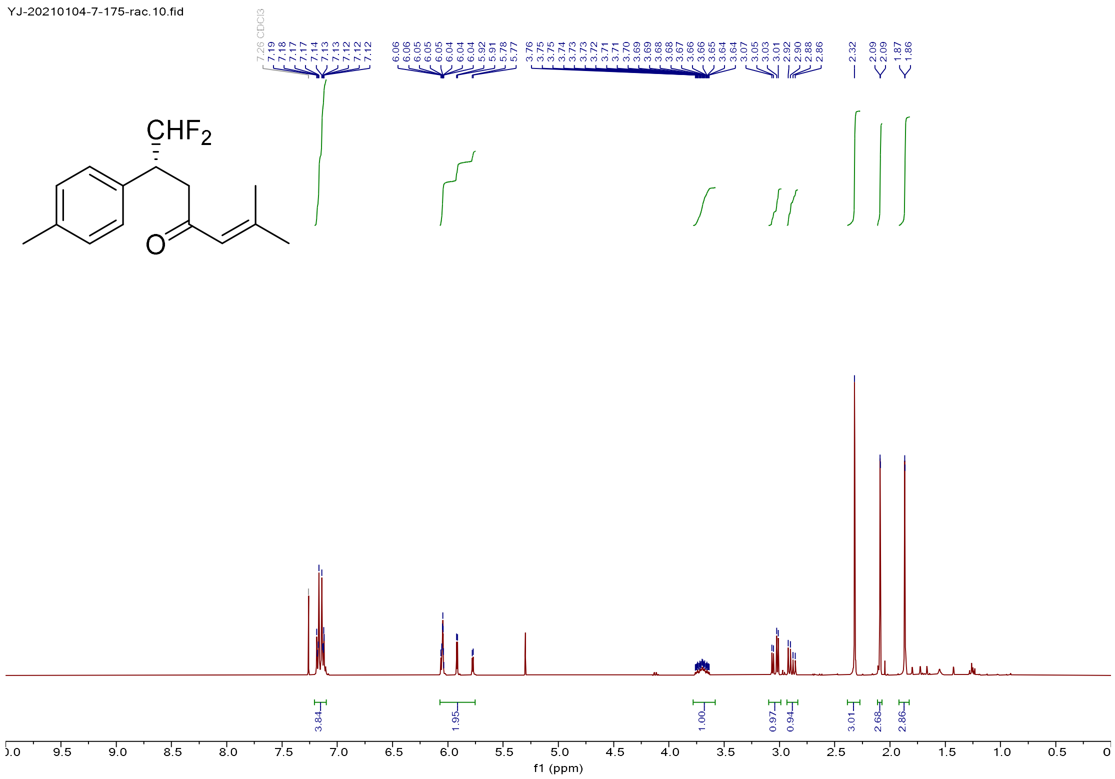
YJ-20210104-7-175-rac.10.fid
CHF2
O
7.26 CDCl3
7.19
7.18
7.17
7.17
7.14
7.13
7.13
7.12
7.12
7.12
6.06
6.06
6.05
6.05
6.05
6.04
6.04
6.04
5.92
5.91
5.78
5.77
3.76
3.75
3.75
3.74
3.73
3.72
3.71
3.71
3.70
3.69
3.69
3.68
3.68
3.67
3.66
3.66
3.65
3.64
3.64
3.07
3.05
3.03
3.01
2.92
2.90
2.88
2.86
2.32
2.09
2.09
1.87
1.86
3.84
1.95
1.00
0.97
0.94
3.01
2.68
2.86
f1 (ppm)

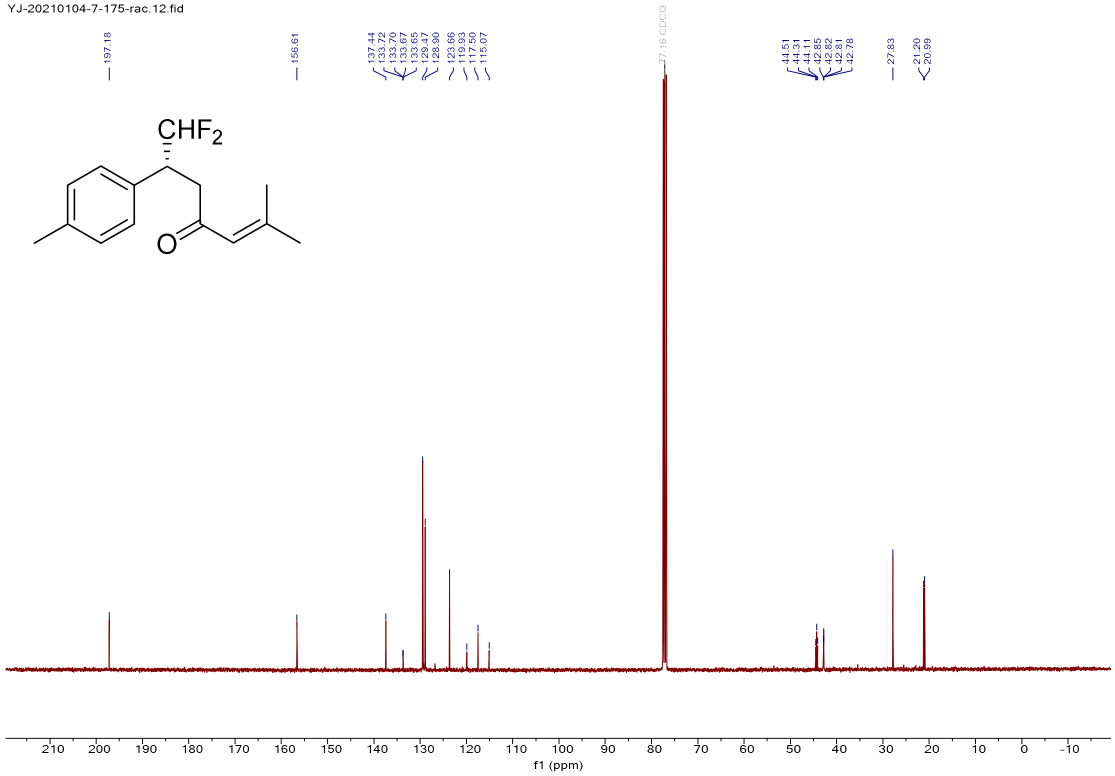
YJ-20210104-7-175-rac.12.fid
CHF2
O
197.18
156.61
137.44
133.72
133.70
133.67
133.65
129.47
128.90
123.66
119.93
117.50
115.07
77.16 CDCl3
44.51
44.31
44.11
42.85
42.82
42.81
42.78
27.83
21.20
20.99
f1 (ppm)

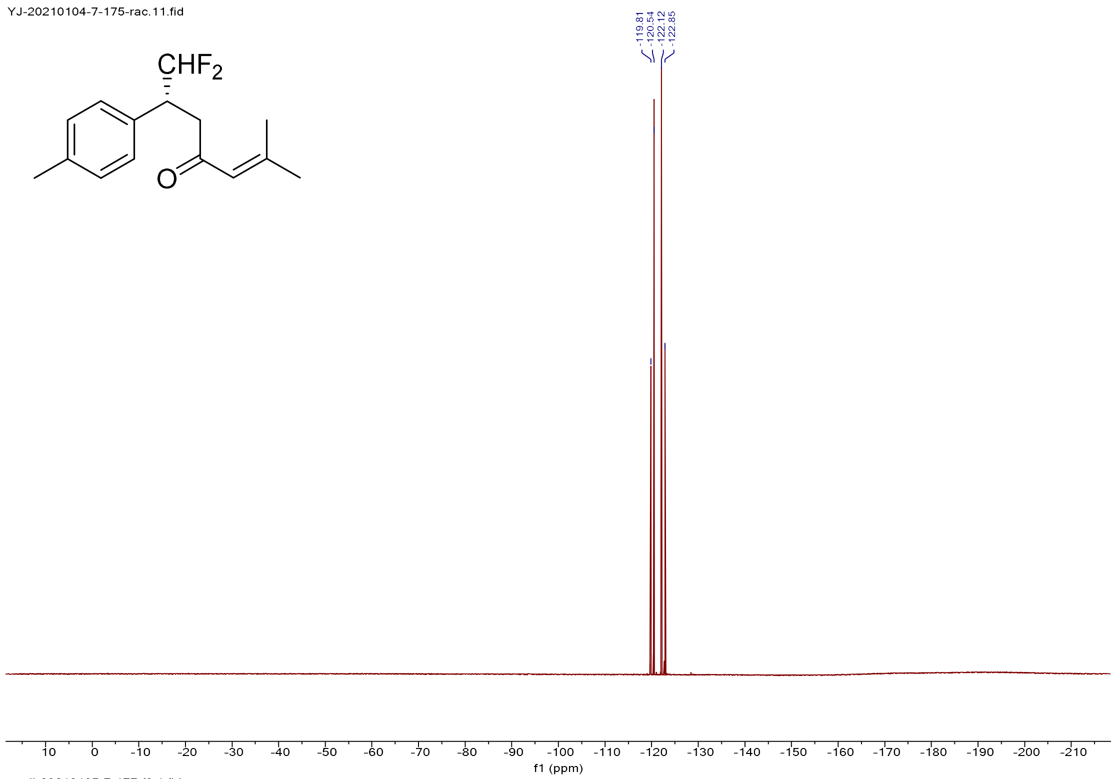
YJ-20210104-7-175-rac.11.fid
CHF2
O
-119.81
-120.54
-122.12
-122.85
10 0 -10 -20 -30 -40 -50 -60 -70 -80 -90 -100 -110 -120 -130 -140 -150 -160 -170 -180 -190 -200 -210
f1 (ppm)

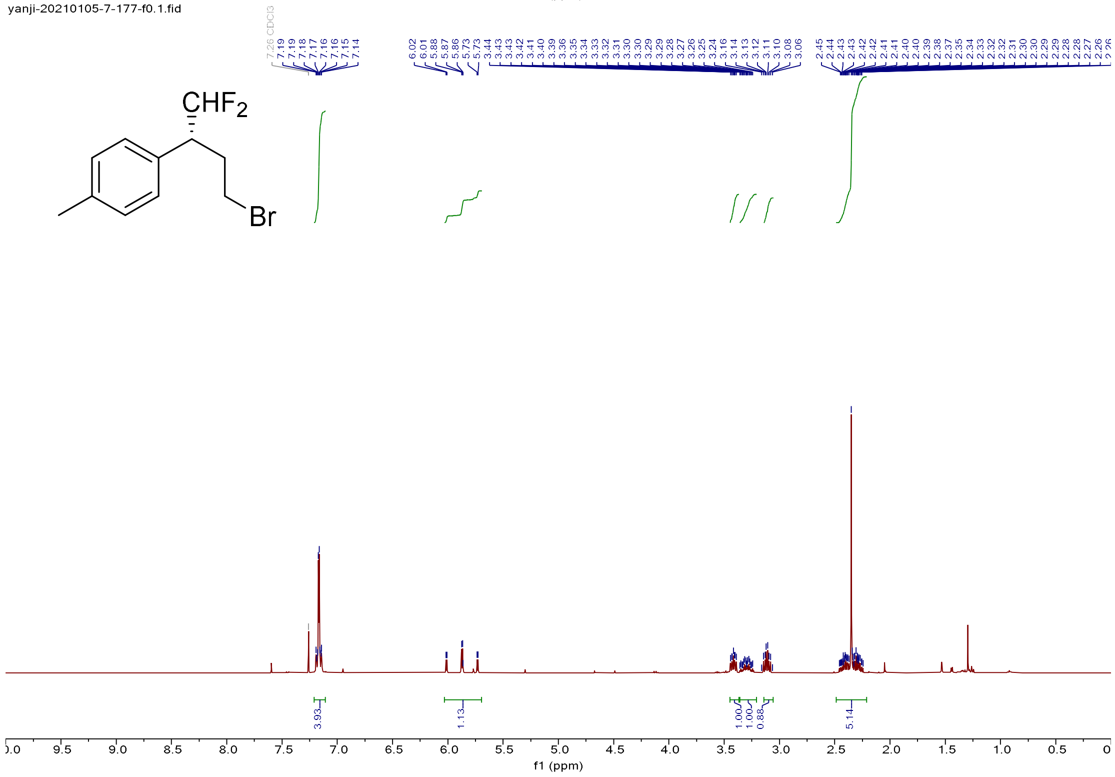
yanji-20210105-7-177-f0.1.fid
CHF2
Br
7.26 CDCl3
7.19 7.19 7.18 7.17 7.16 7.16 7.15 7.14
6.02 6.01 5.88 5.87 5.86 5.73 5.73
3.44 3.43 3.42 3.41 3.40 3.39 3.36 3.35 3.34 3.33 3.32 3.31 3.30 3.30 3.29 3.29 3.28 3.27 3.26 3.25 3.24 3.16 3.14 3.13 3.12 3.11 3.10 3.08 3.06
2.45 2.44 2.43 2.43 2.42 2.42 2.41 2.41 2.40 2.39 2.38 2.37 2.35 2.34 2.33 2.32 2.32 2.31 2.30 2.30 2.29 2.29 2.28 2.27 2.26 [illegible]
3.93
1.13
1.00
1.00
0.88
5.14
10.0 9.5 9.0 8.5 8.0 7.5 7.0 6.5 6.0 5.5 5.0 4.5 4.0 3.5 3.0 2.5 2.0 1.5 1.0 0.5 0
f1 (ppm)

yanji-20210105-7-177-f0.3.fid

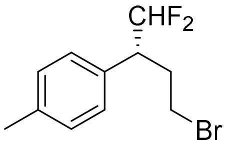

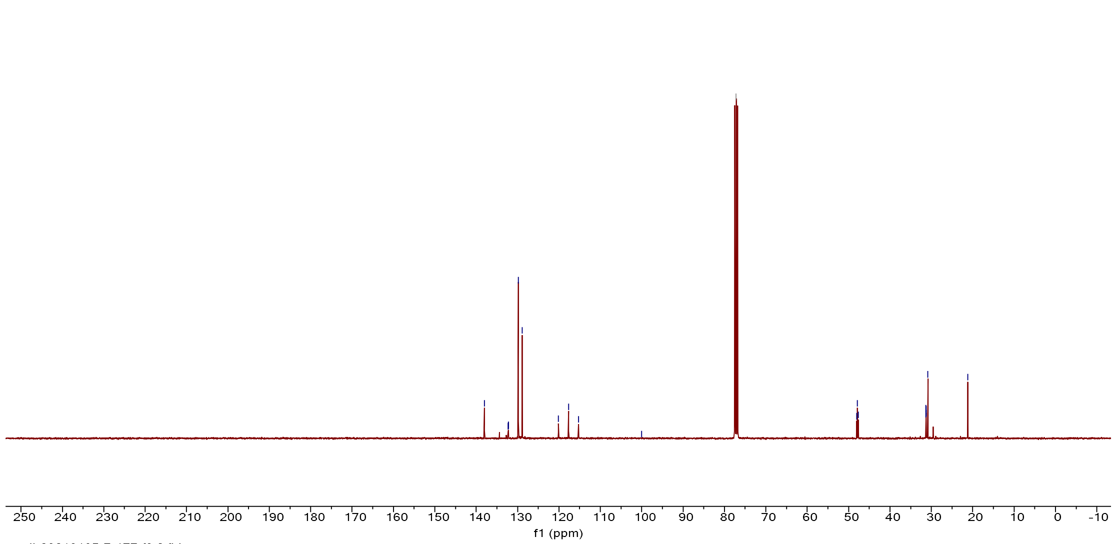

yanji-20210105-7-177-f0.2.fid

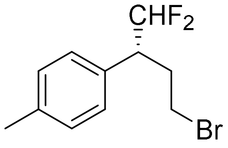

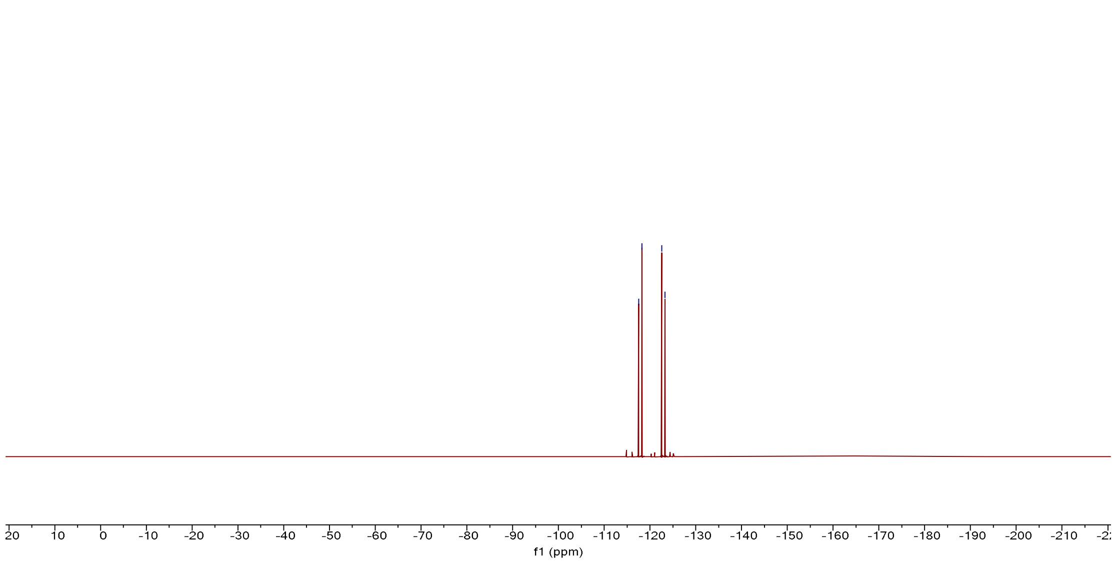

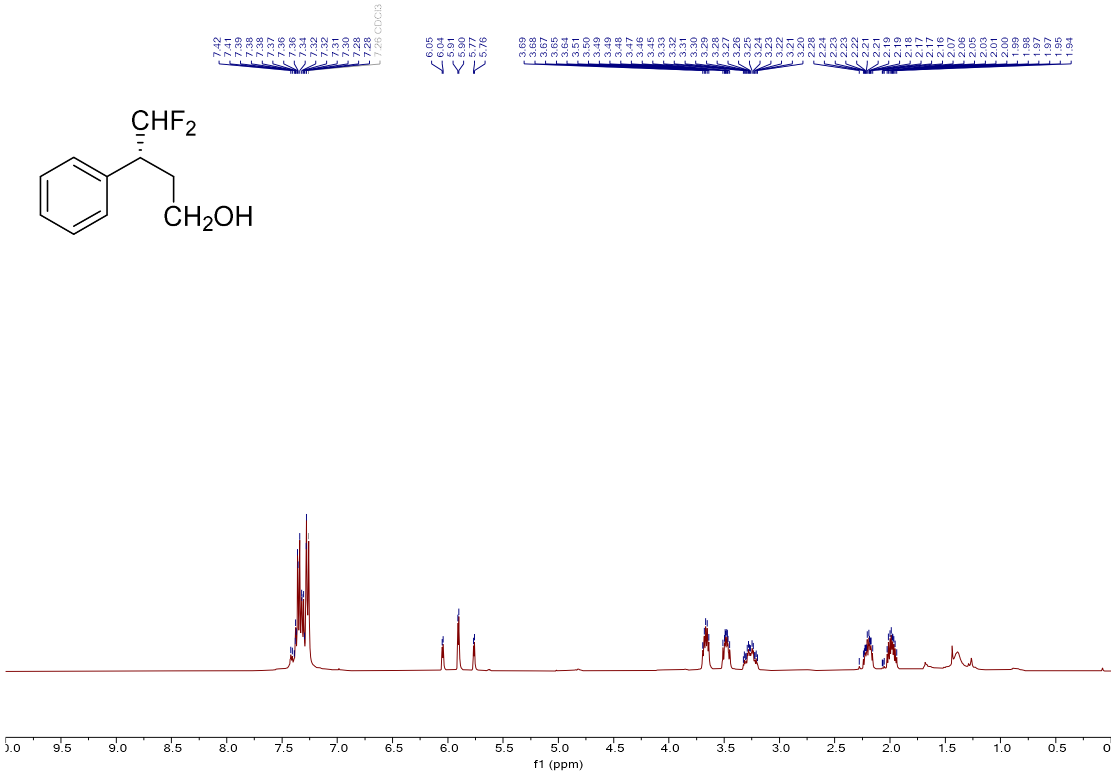
CHF2
CH2OH
7.42 7.41 7.39 7.38 7.38 7.37 7.36 7.36 7.34 7.32 7.32 7.31 7.30 7.28 7.28
7.26 CDCl3
6.05 6.04 5.91 5.90 5.77 5.76
3.69 3.68 3.67 3.65 3.64 3.51 3.50 3.49 3.49 3.48 3.47 3.46 3.45 3.33 3.32 3.31 3.30 3.29 3.28 3.27 3.26 3.25 3.24 3.23 3.22 3.21 3.20
2.28 2.24 2.23 2.23 2.22 2.21 2.21 2.19 2.19 2.18 2.17 2.17 2.16 2.07 2.06 2.05 2.03 2.01 2.00 1.99 1.98 1.97 1.95 1.94
f1 (ppm)

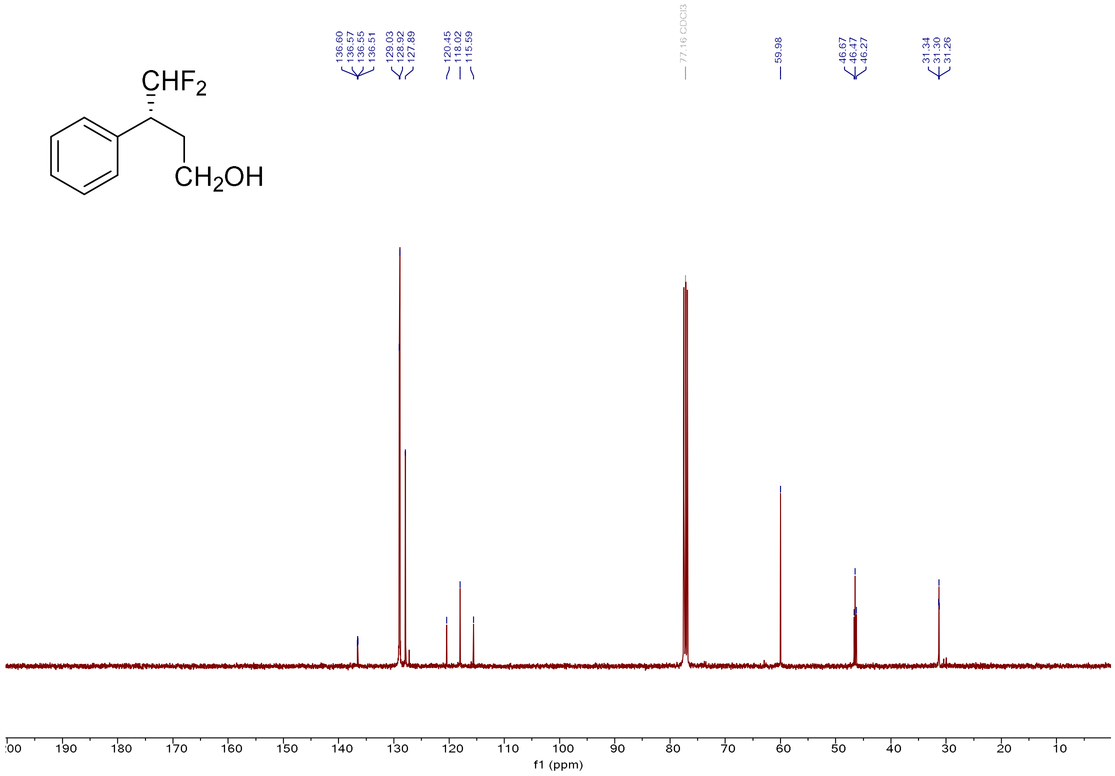
CHF2
CH2OH
136.60 136.57 136.55 136.51
129.03 128.92 127.89
120.45 118.02 115.59
77.16 CDCl3
59.98
46.67 46.47 46.27
31.34 31.30 31.26
f1 (ppm)

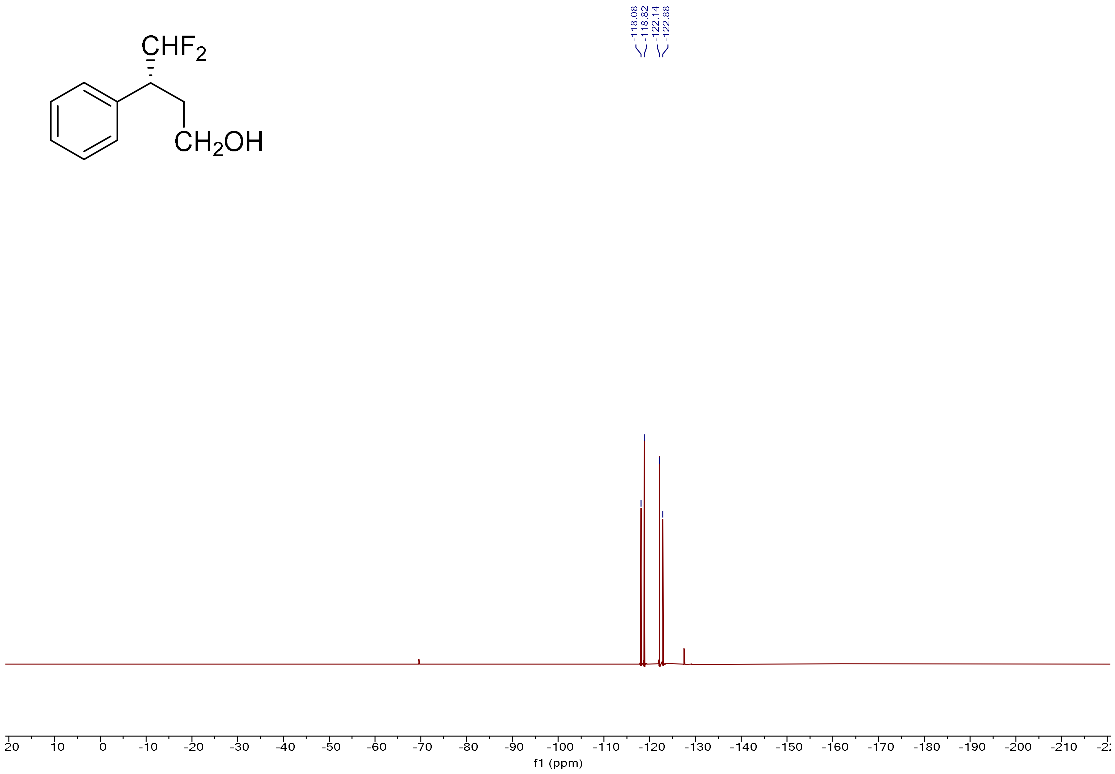
CHF2
CH2OH
f1 (ppm)

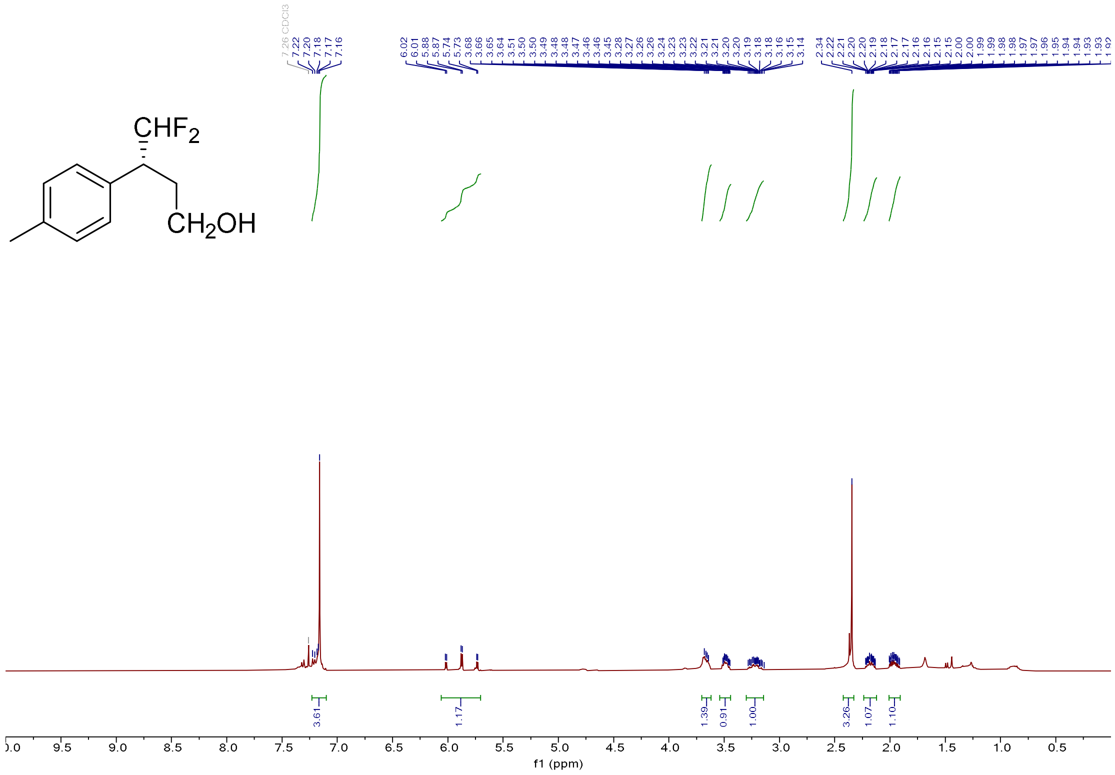
CHF2
CH2OH
f1 (ppm)

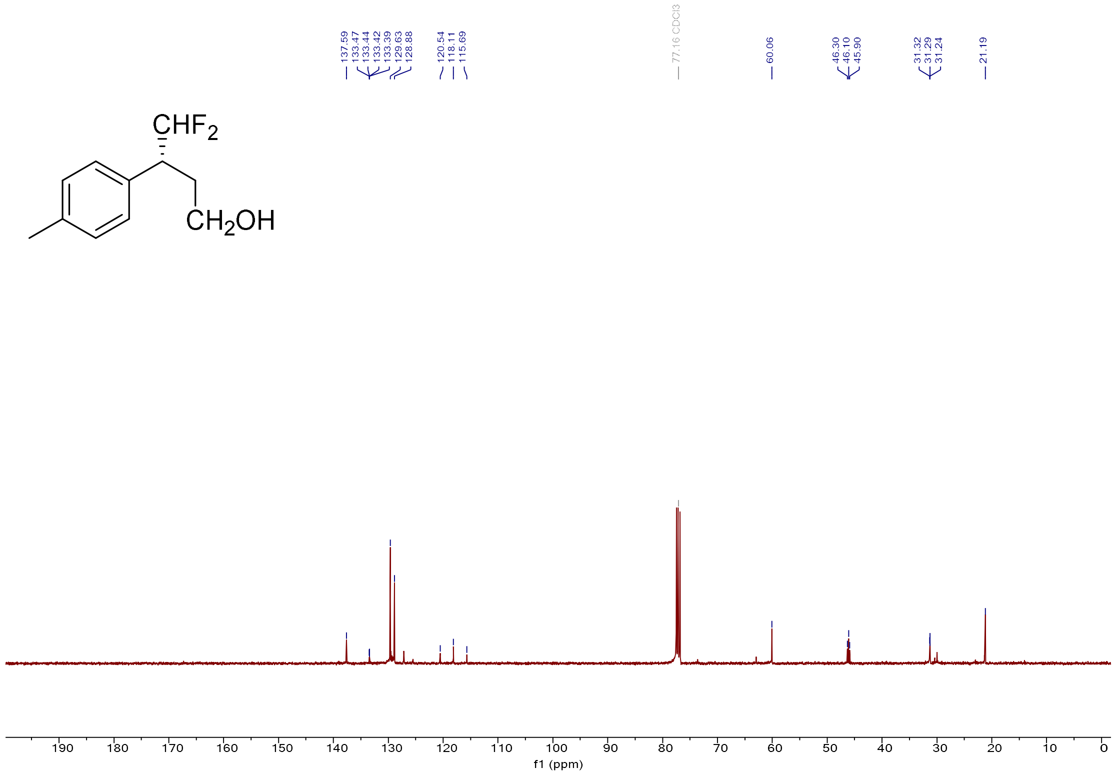
CHF2
CH2OH
137.59
133.47
133.44
133.42
133.39
129.63
128.88
120.54
118.11
115.69
77.16 CDCl3
60.06
46.30
46.10
45.90
31.32
31.29
31.24
21.19
f1 (ppm)

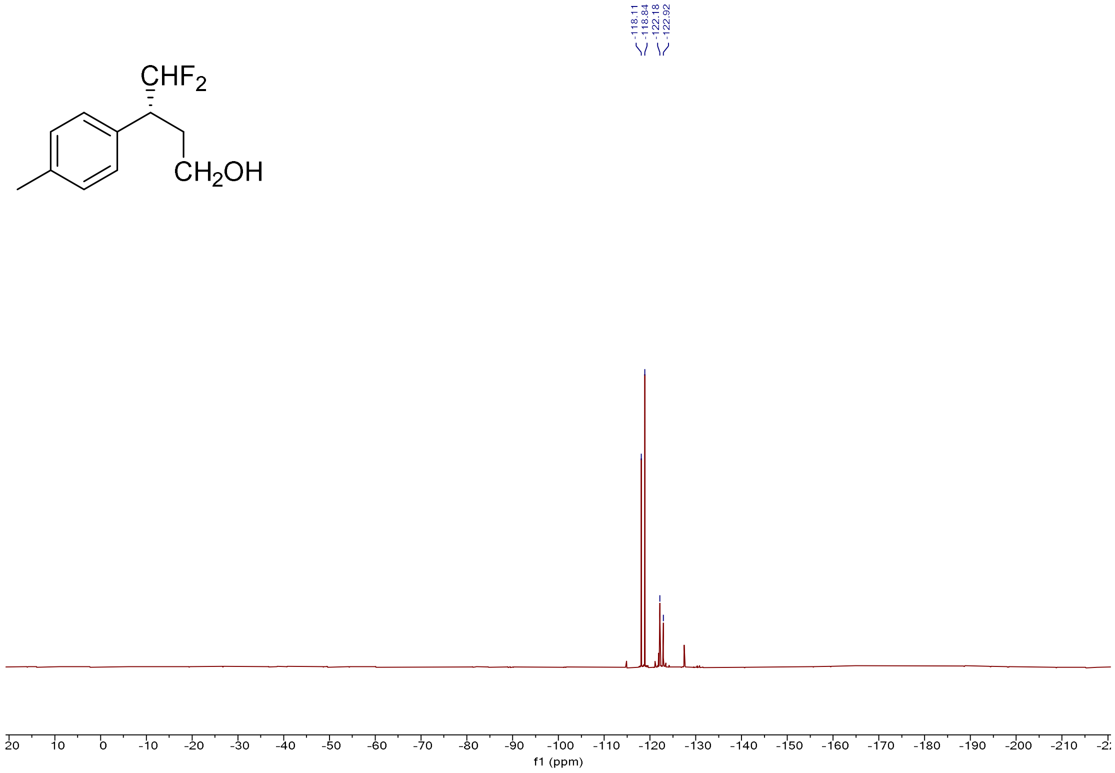
CHF2
CH2OH
-118.11
-118.84
-122.18
-122.92
f1 (ppm)

10. Chromatograms

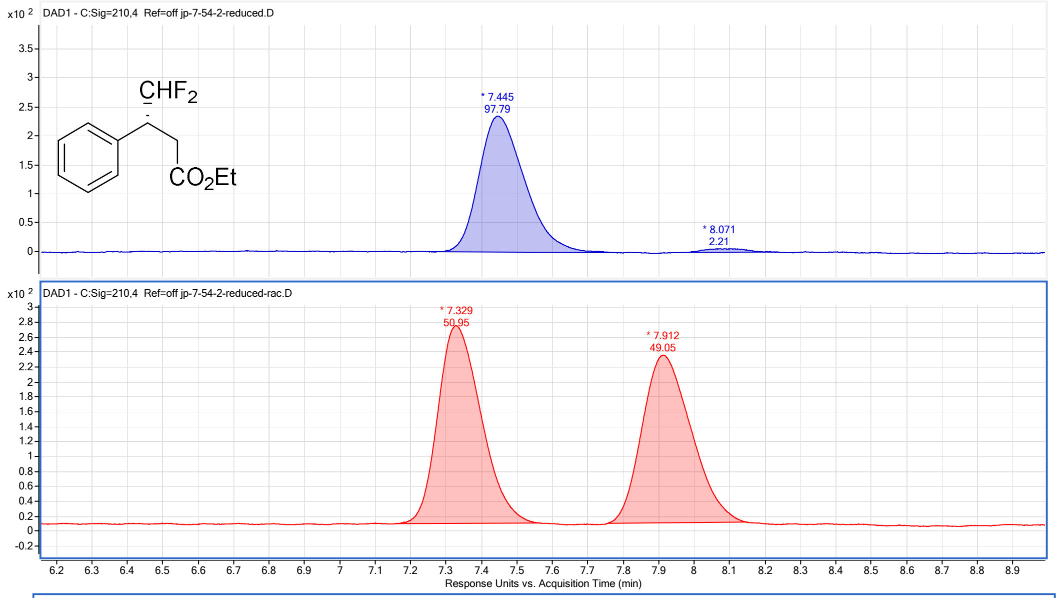

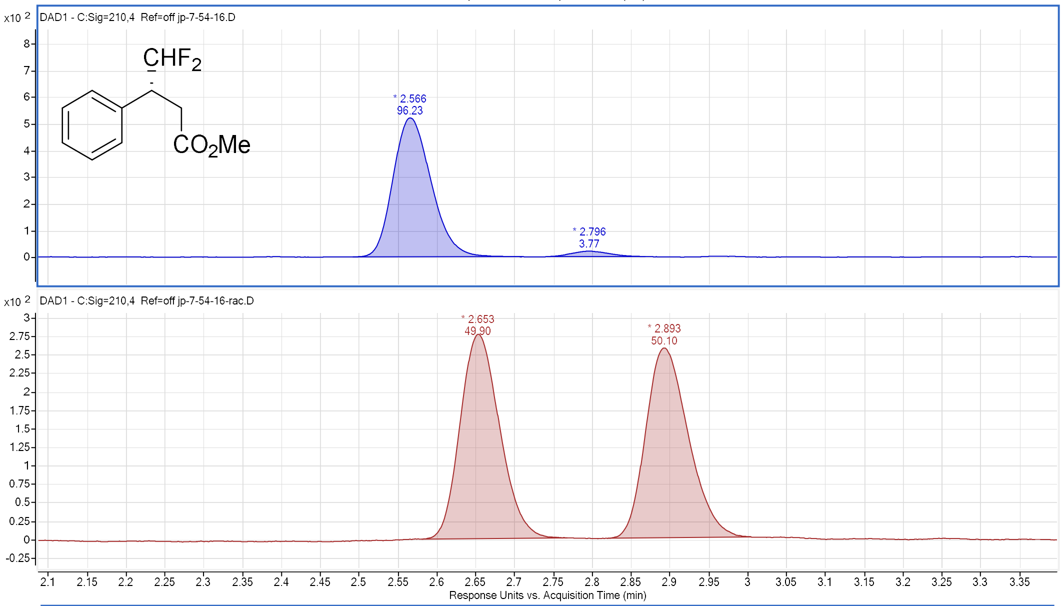

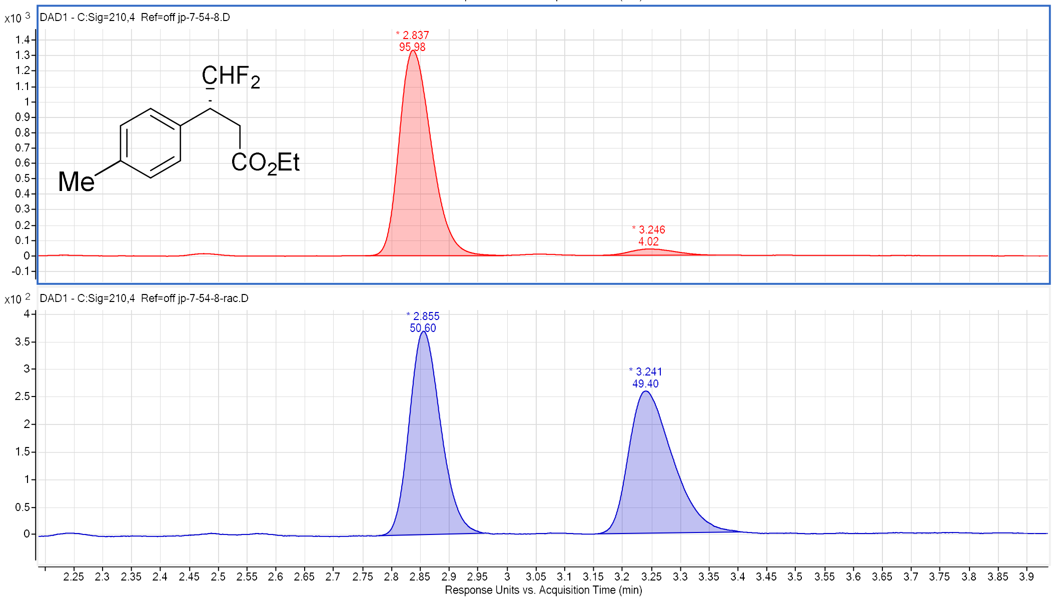

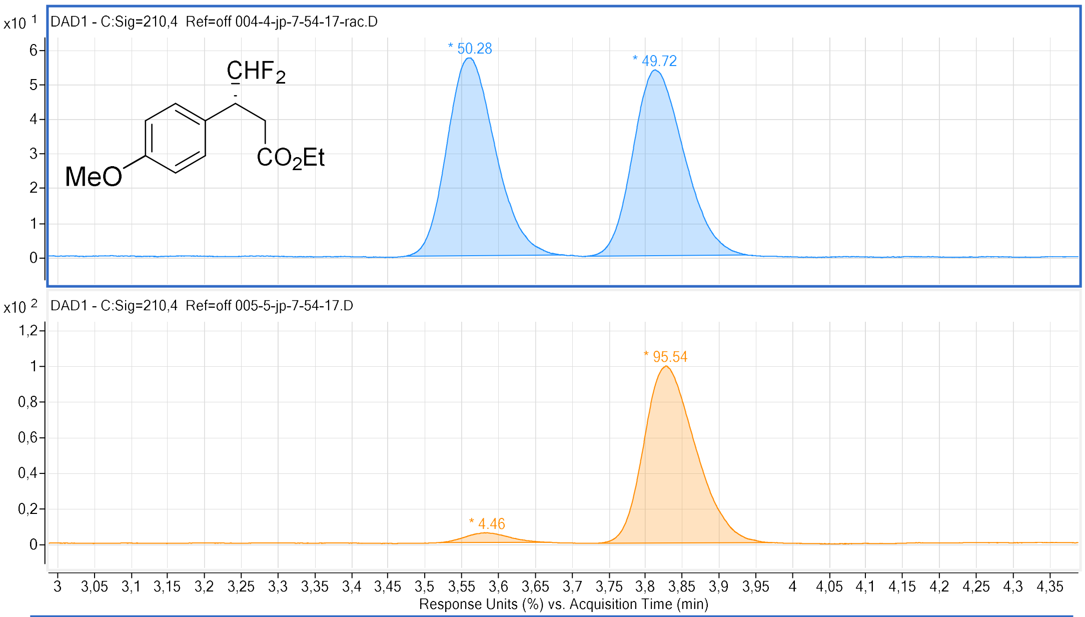

x10 1
DAD1 - C:Sig=210,4 Ref=off 004-4-jp-7-54-17-rac.D
* 50.28
* 49.72
CHF2
CO2Et
MeO
x10 2
DAD1 - C:Sig=210,4 Ref=off 005-5-jp-7-54-17.D
* 95.54
* 4.46
Response Units (%) vs. Acquisition Time (min)

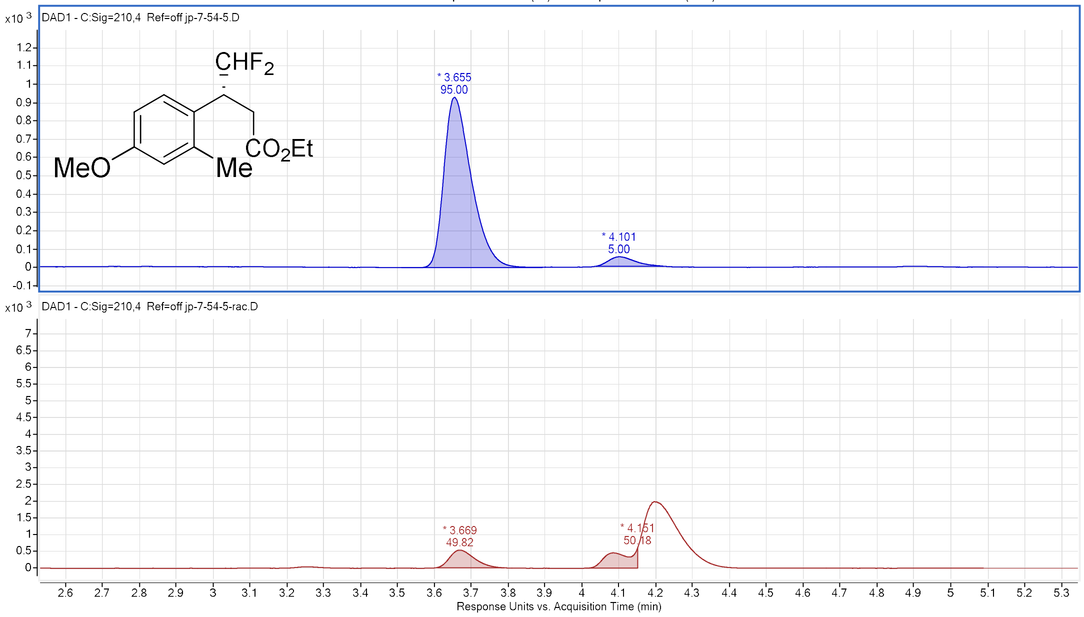

x10 3
DAD1 - C:Sig=210,4 Ref=off jp-7-54-5.D
* 3.655
95.00
* 4.101
5.00
CHF2
CO2Et
MeO
Me
x10 3
DAD1 - C:Sig=210,4 Ref=off jp-7-54-5-rac.D
* 3.669
49.82
* 4.151
50.18
Response Units vs. Acquisition Time (min)

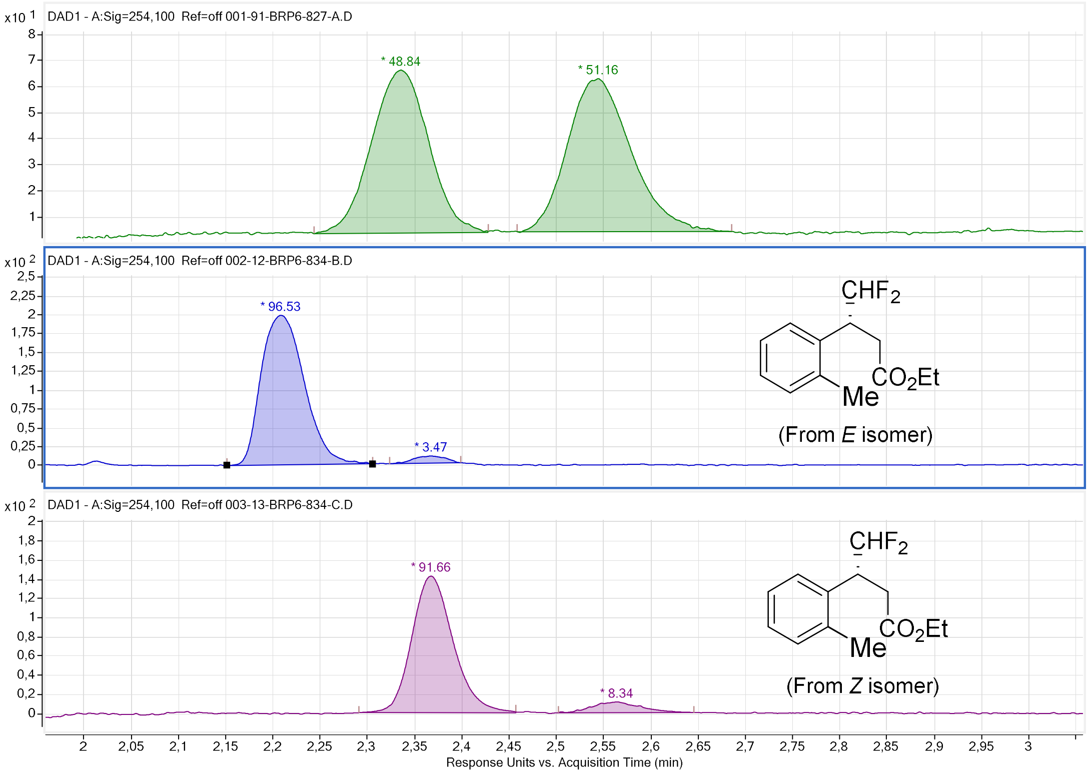
DAD1 - A:Sig=254,100 Ref=off 001-91-BRP6-827-A.D
* 48.84
* 51.16
DAD1 - A:Sig=254,100 Ref=off 002-12-BRP6-834-B.D
* 96.53
* 3.47
CHF2
CO2Et
Me
(From E isomer)
DAD1 - A:Sig=254,100 Ref=off 003-13-BRP6-834-C.D
* 91.66
* 8.34
CHF2
CO2Et
Me
(From Z isomer)
Response Units vs. Acquisition Time (min)

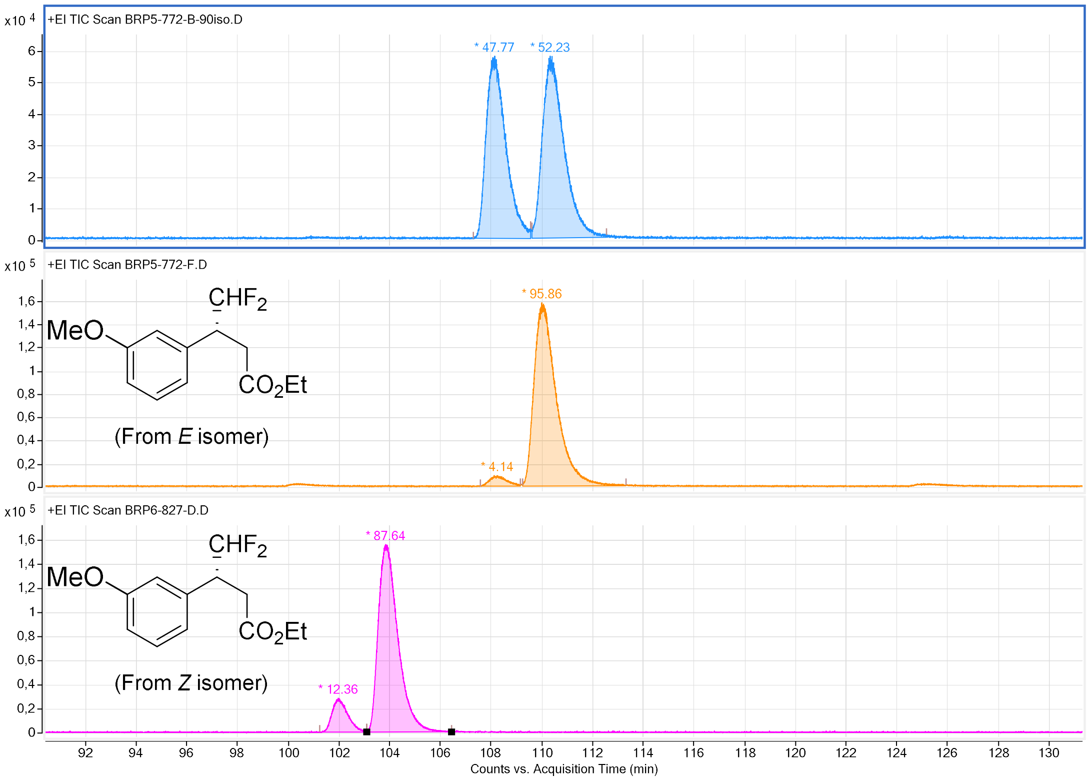
+EI TIC Scan BRP5-772-B-90iso.D
* 47.77
* 52.23
+EI TIC Scan BRP5-772-F.D
MeO
CHF2
CO2Et
(From E isomer)
* 95.86
* 4.14
+EI TIC Scan BRP6-827-D.D
MeO
CHF2
CO2Et
(From Z isomer)
* 87.64
* 12.36
Counts vs. Acquisition Time (min)

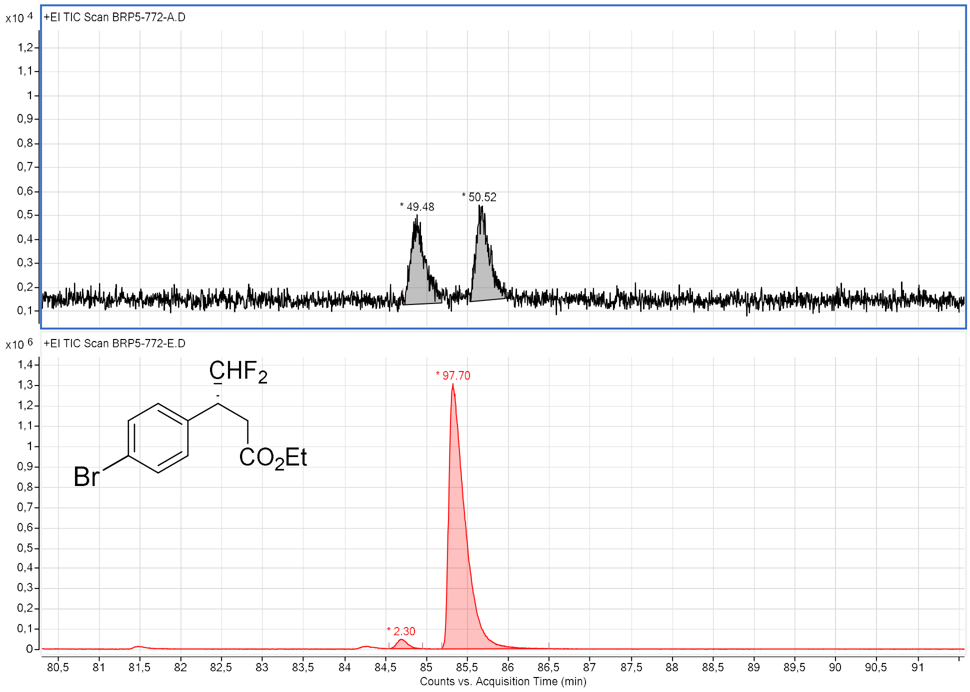
+EI TIC Scan BRP5-772-A.D
* 49.48
* 50.52
+EI TIC Scan BRP5-772-E.D
CHF2
CO2Et
Br
* 97.70
* 2.30
Counts vs. Acquisition Time (min)

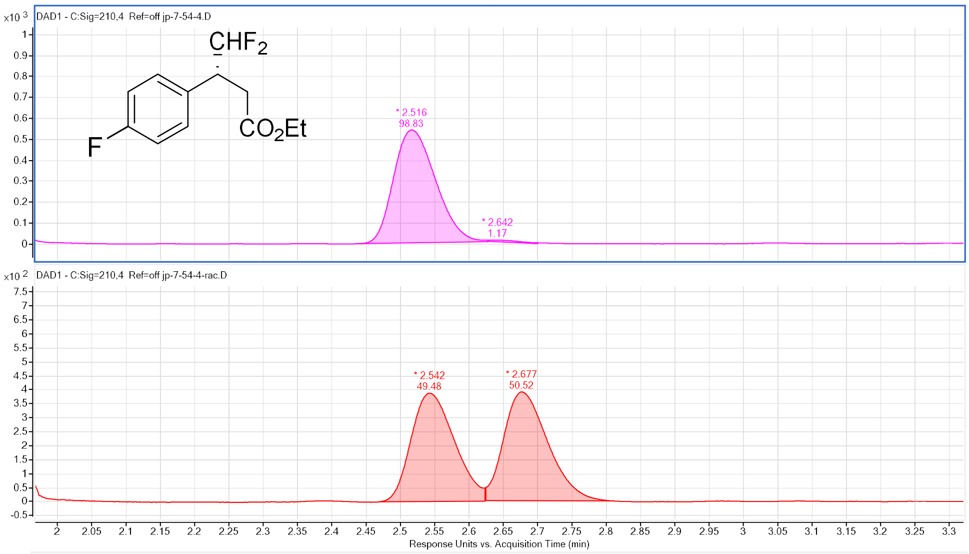
DAD1 - C:Sig=210,4 Ref=off jp-7-54-4.D
CHF2
CO2Et
F
* 2.516
98.83
* 2.642
1.17
DAD1 - C:Sig=210,4 Ref=off jp-7-54-4-rac.D
* 2.542
49.48
* 2.677
50.52
Response Units vs. Acquisition Time (min)

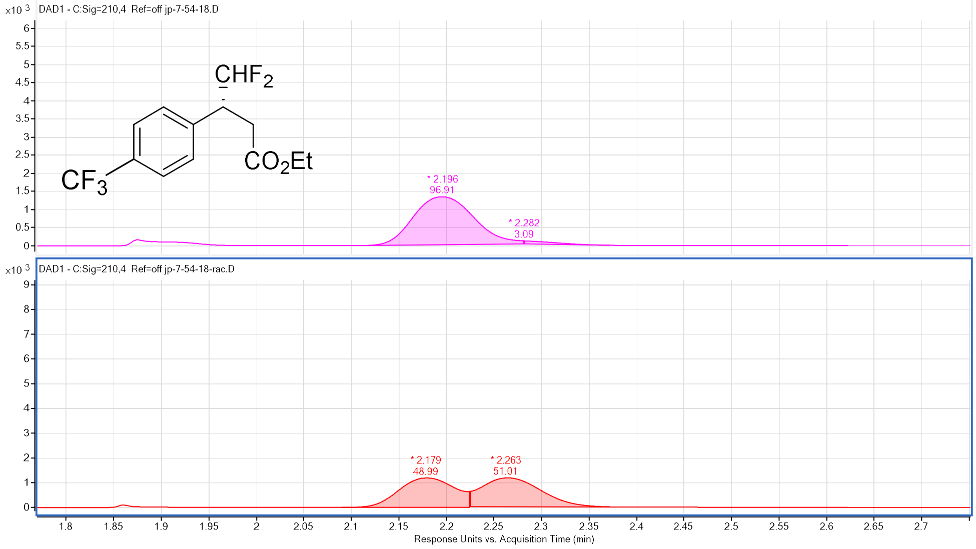
DAD1 - C:Sig=210,4 Ref=off jp-7-54-18.D
CHF2
CO2Et
CF3
* 2.196
96.91
* 2.282
3.09
DAD1 - C:Sig=210,4 Ref=off jp-7-54-18-rac.D
* 2.179
48.99
* 2.263
51.01
Response Units vs. Acquisition Time (min)

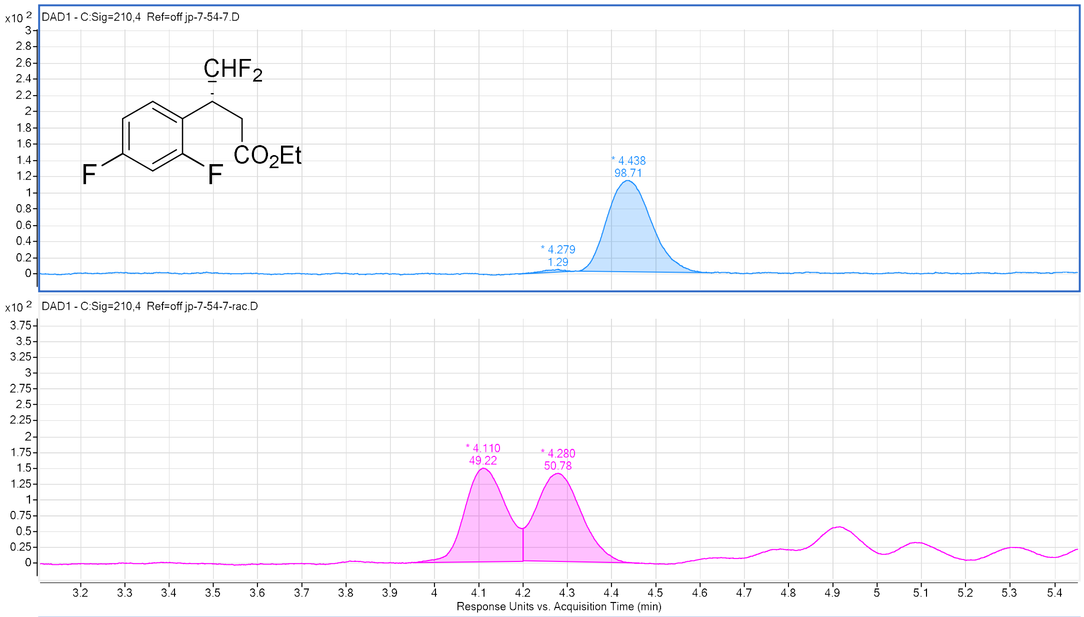
DAD1 - C:Sig=210,4 Ref=off jp-7-54-7.D
CHF2
CO2Et
F
F
* 4.438
98.71
* 4.279
1.29
DAD1 - C:Sig=210,4 Ref=off jp-7-54-7-rac.D
* 4.110
49.22
* 4.280
50.78
Response Units vs. Acquisition Time (min)

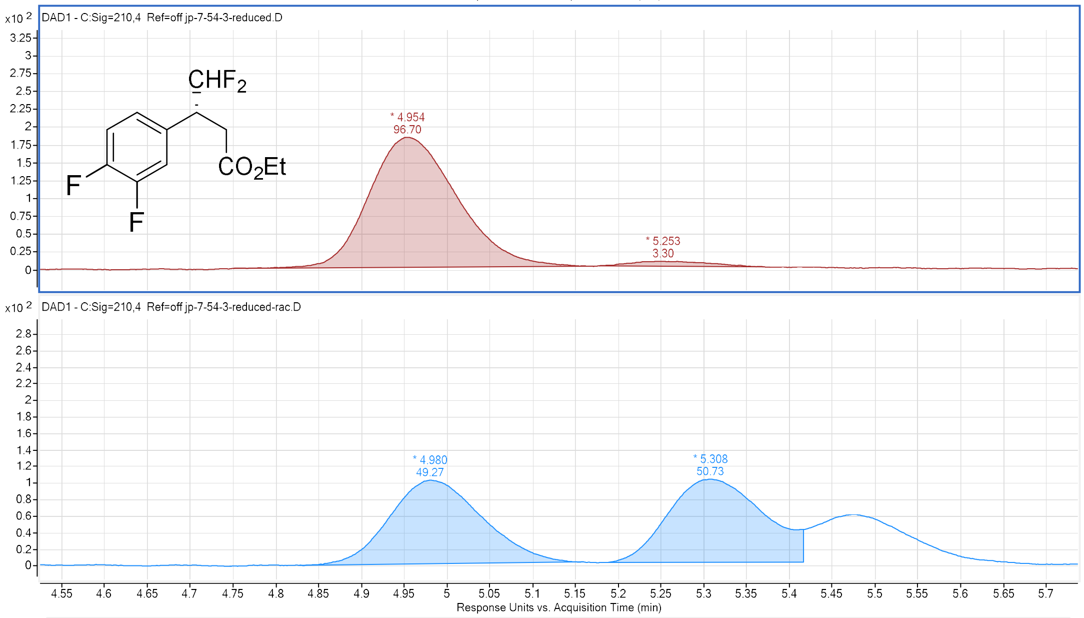
DAD1 - C:Sig=210,4 Ref=off jp-7-54-3-reduced.D
CHF2
CO2Et
F
F
* 4.954
96.70
* 5.253
3.30
DAD1 - C:Sig=210,4 Ref=off jp-7-54-3-reduced-rac.D
* 4.980
49.27
* 5.308
50.73
Response Units vs. Acquisition Time (min)

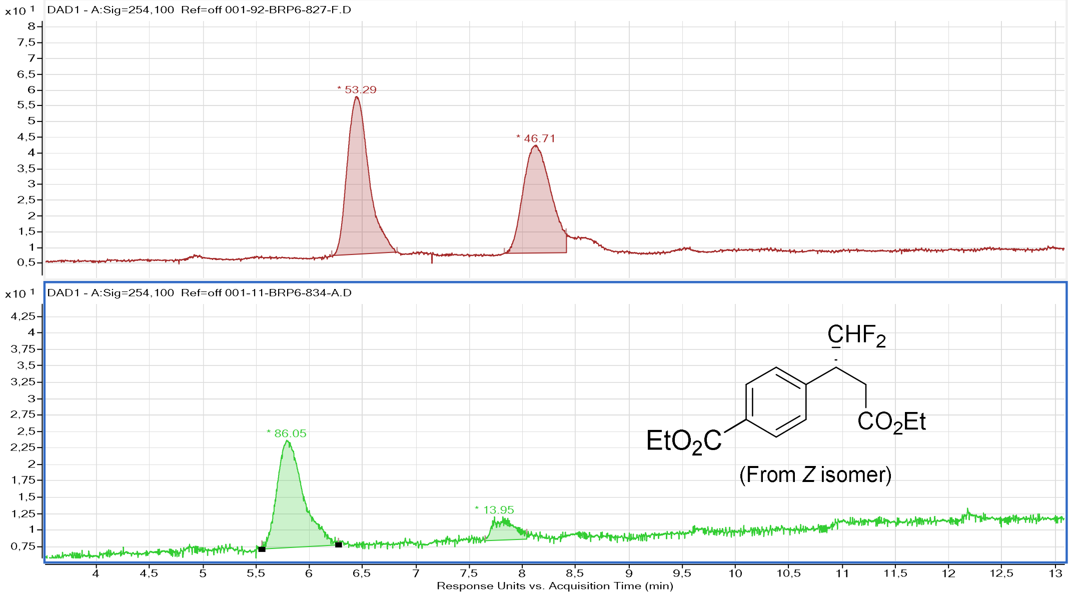
DAD1 - A:Sig=254,100 Ref=off 001-92-BRP6-827-F.D
* 53.29
* 46.71
DAD1 - A:Sig=254,100 Ref=off 001-11-BRP6-834-A.D
CHF2
EtO2C
CO2Et
(From Z isomer)
* 86.05
* 13.95
Response Units vs. Acquisition Time (min)

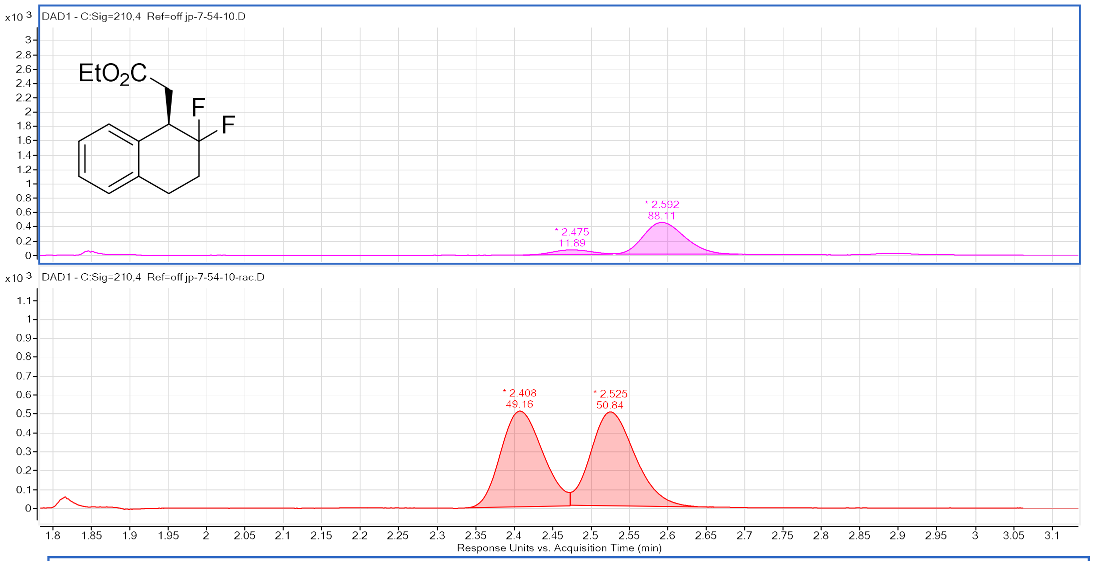
DAD1 - C:Sig=210,4 Ref=off jp-7-54-10.D
EtO2C
F
F
* 2.475
11.89
* 2.592
88.11
DAD1 - C:Sig=210,4 Ref=off jp-7-54-10-rac.D
* 2.408
49.16
* 2.525
50.84
Response Units vs. Acquisition Time (min)

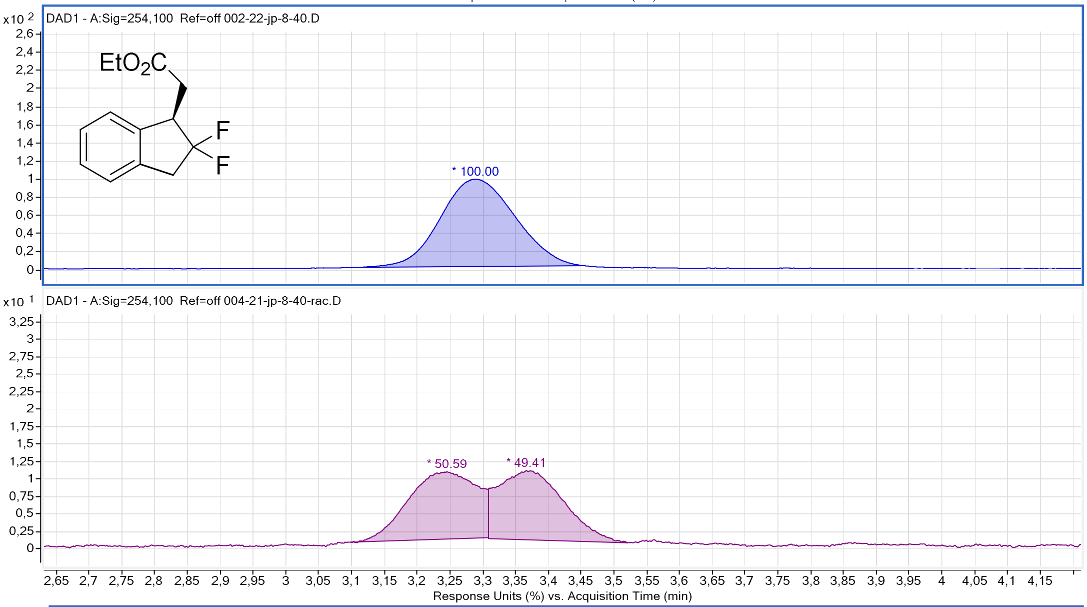
DAD1 - A:Sig=254,100 Ref=off 002-22-jp-8-40.D
EtO2C
F
F
* 100.00
DAD1 - A:Sig=254,100 Ref=off 004-21-jp-8-40-rac.D
* 50.59
* 49.41
Response Units (%) vs. Acquisition Time (min)

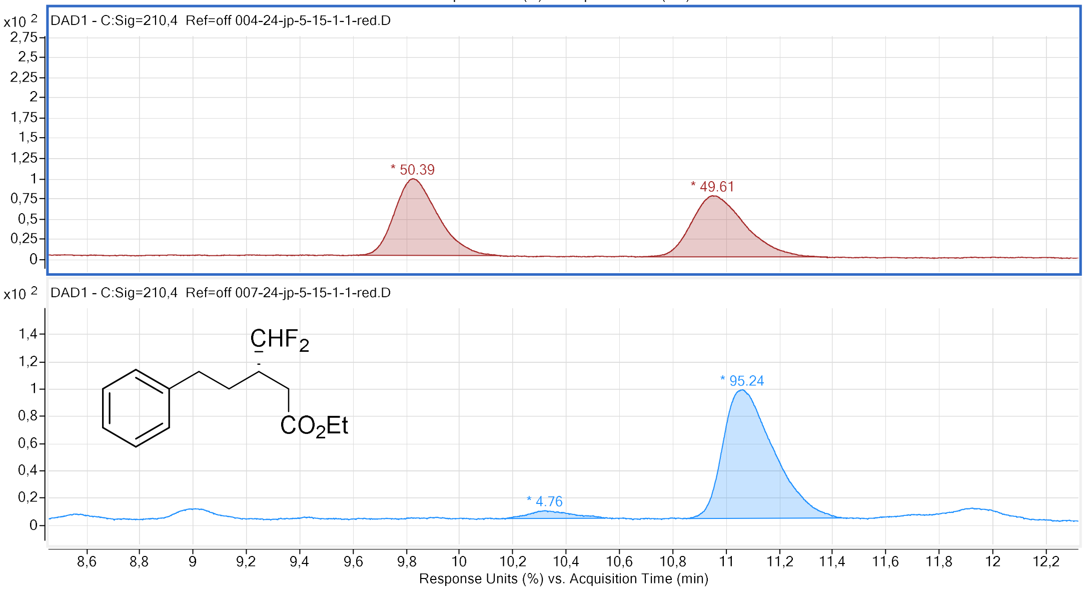
DAD1 - C:Sig=210,4 Ref=off 004-24-jp-5-15-1-1-red.D
* 50.39
* 49.61
DAD1 - C:Sig=210,4 Ref=off 007-24-jp-5-15-1-1-red.D
CHF2
CO2Et
* 4.76
* 95.24
Response Units (%) vs. Acquisition Time (min)

Chromatogram Plots

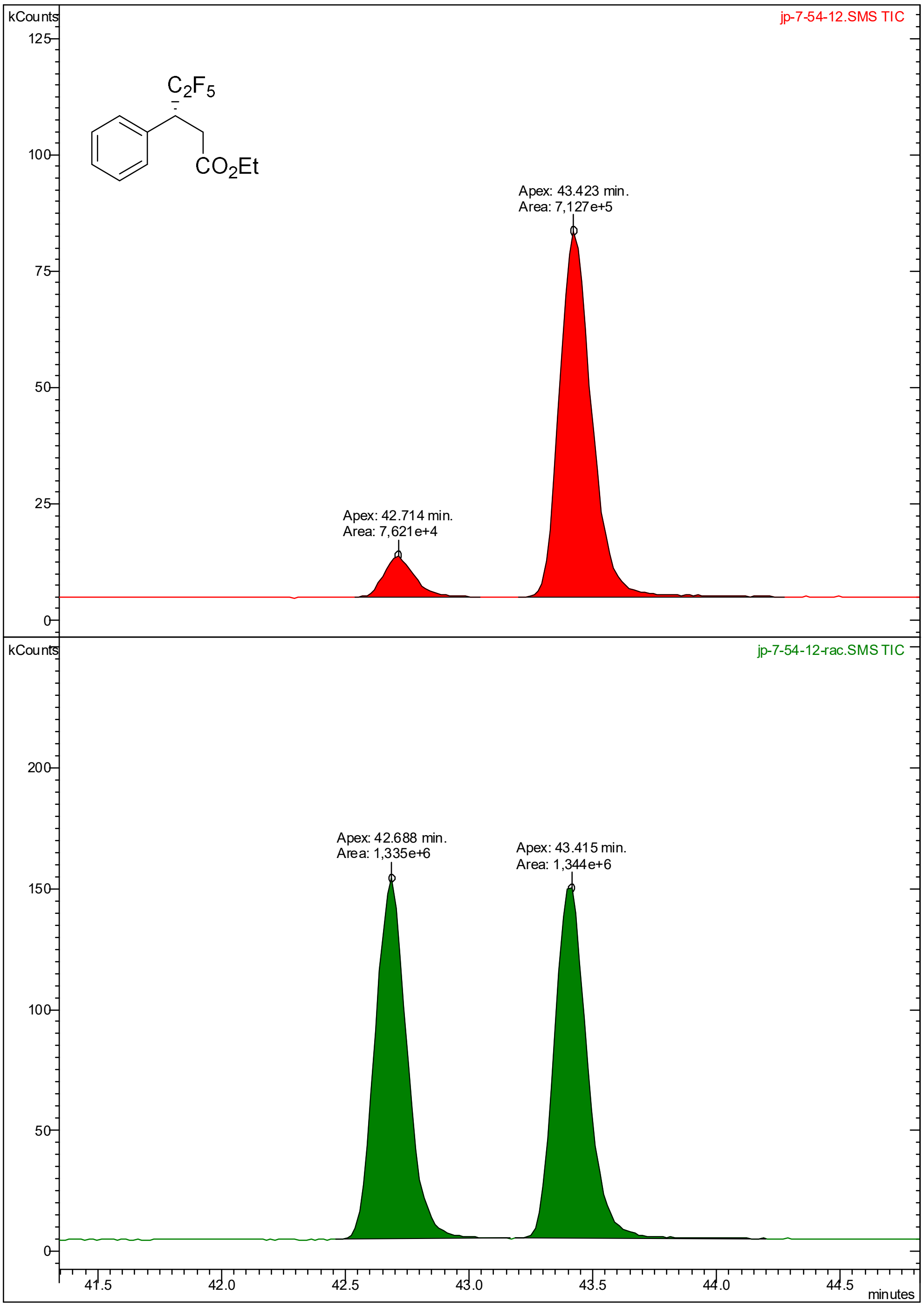

Chromatogram Plots

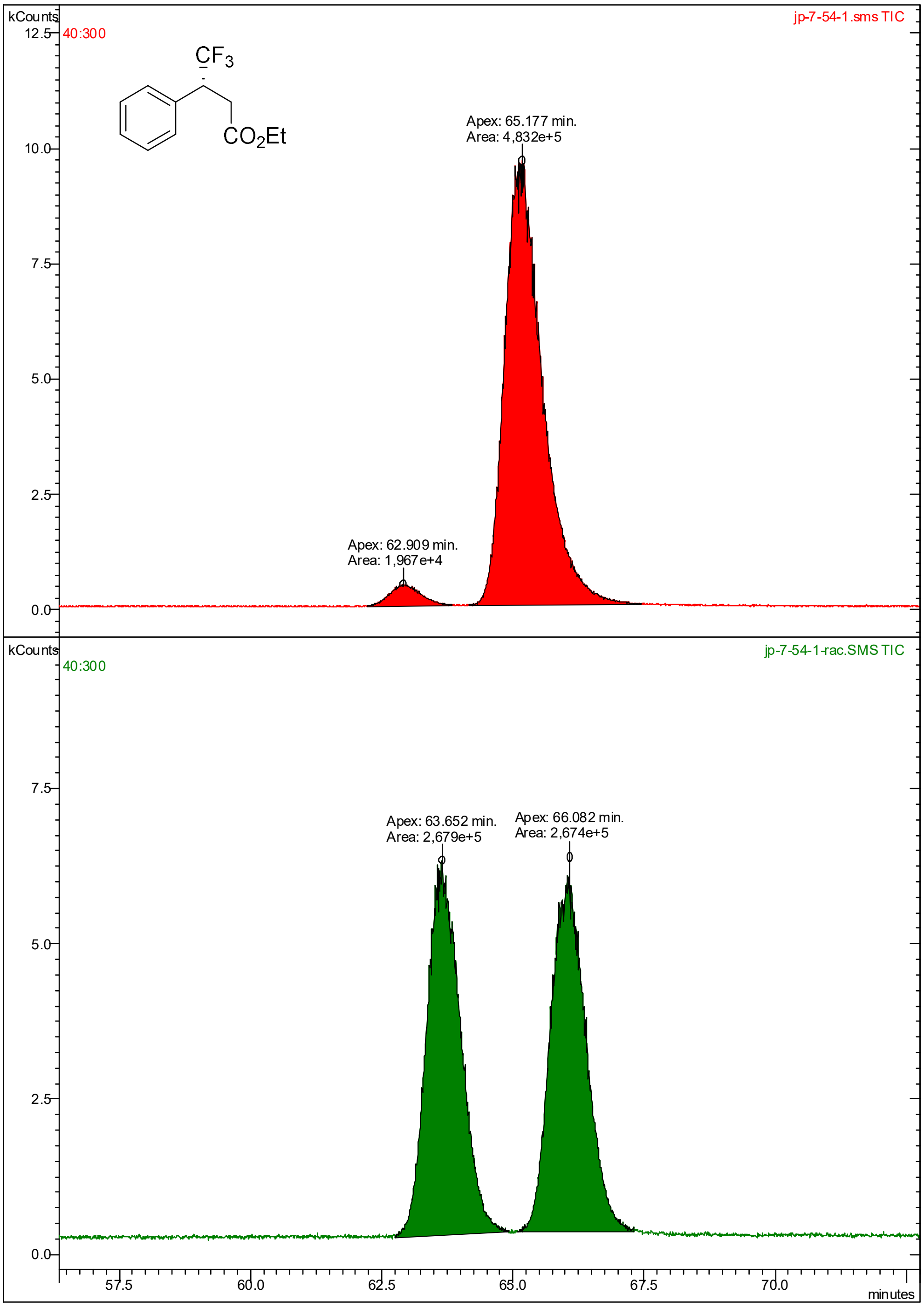

Chromatogram Plots

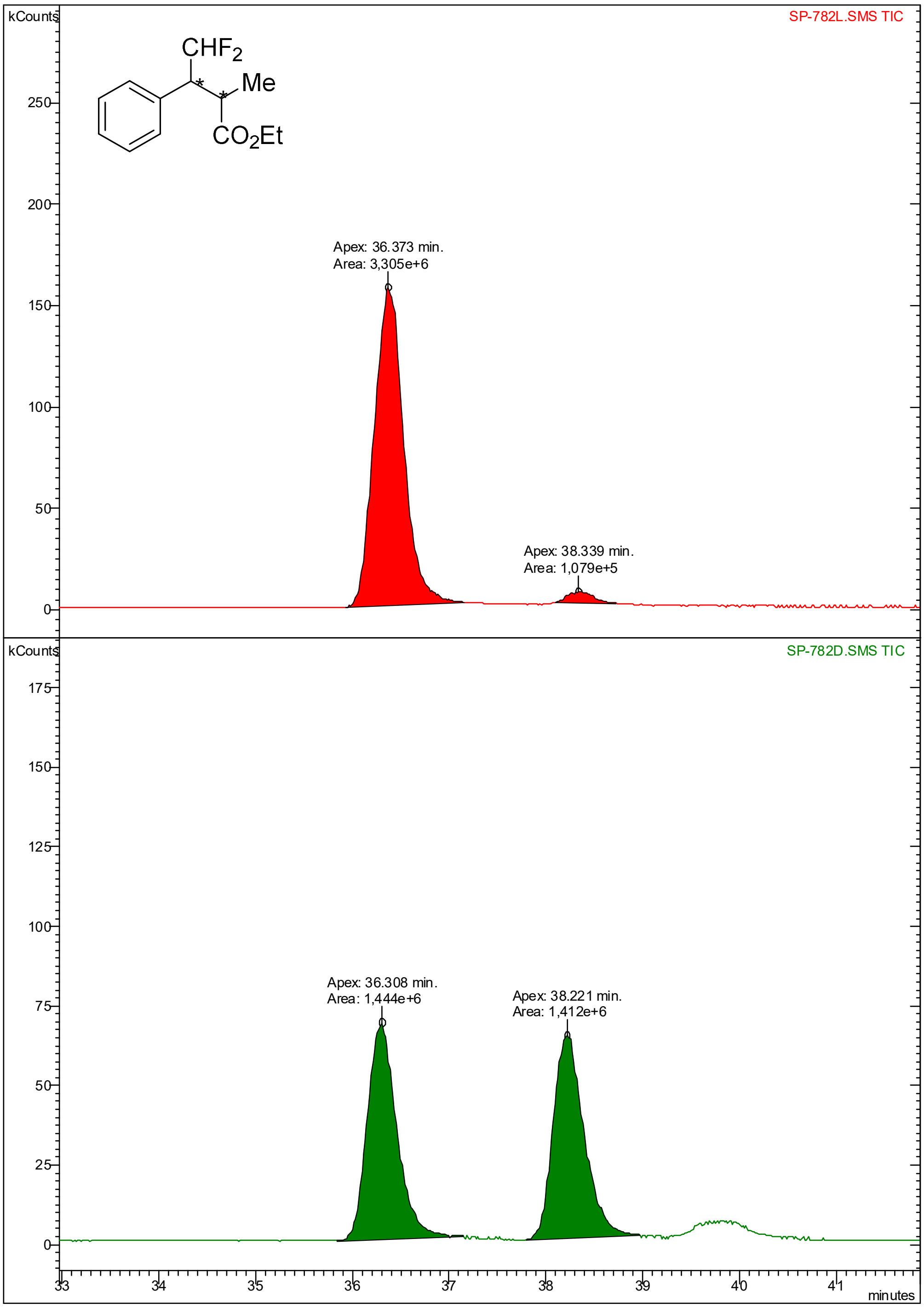

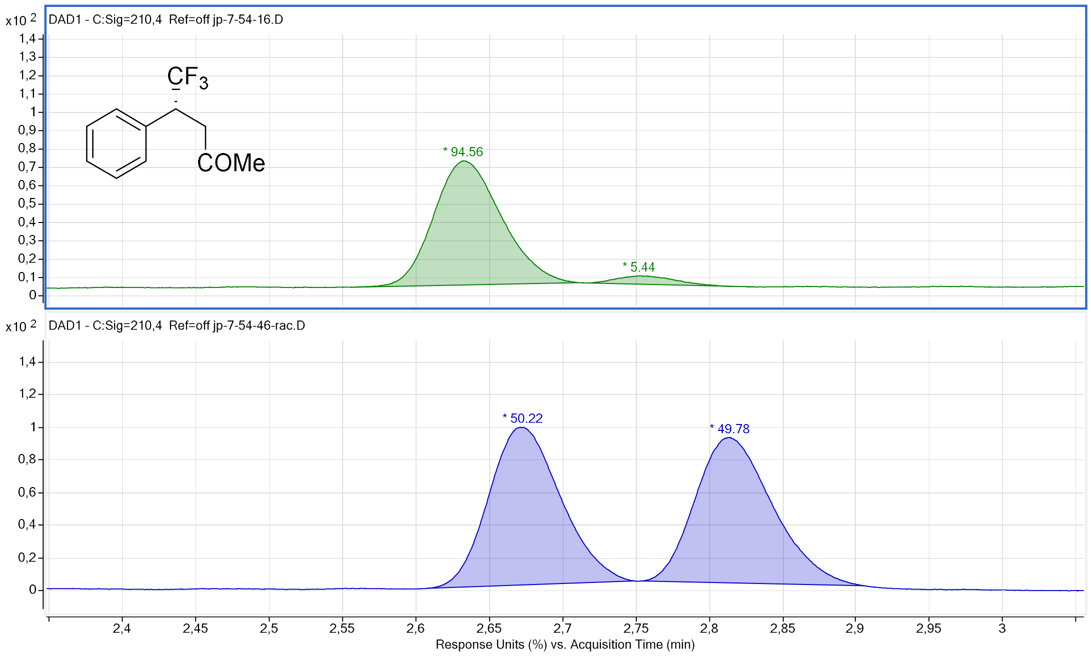
x10 2
DAD1 - C:Sig=210,4 Ref=off jp-7-54-16.D
CF3
COMe
* 94.56
* 5.44
x10 2
DAD1 - C:Sig=210,4 Ref=off jp-7-54-46-rac.D
* 50.22
* 49.78
Response Units (%) vs. Acquisition Time (min)

Chromatogram Plots

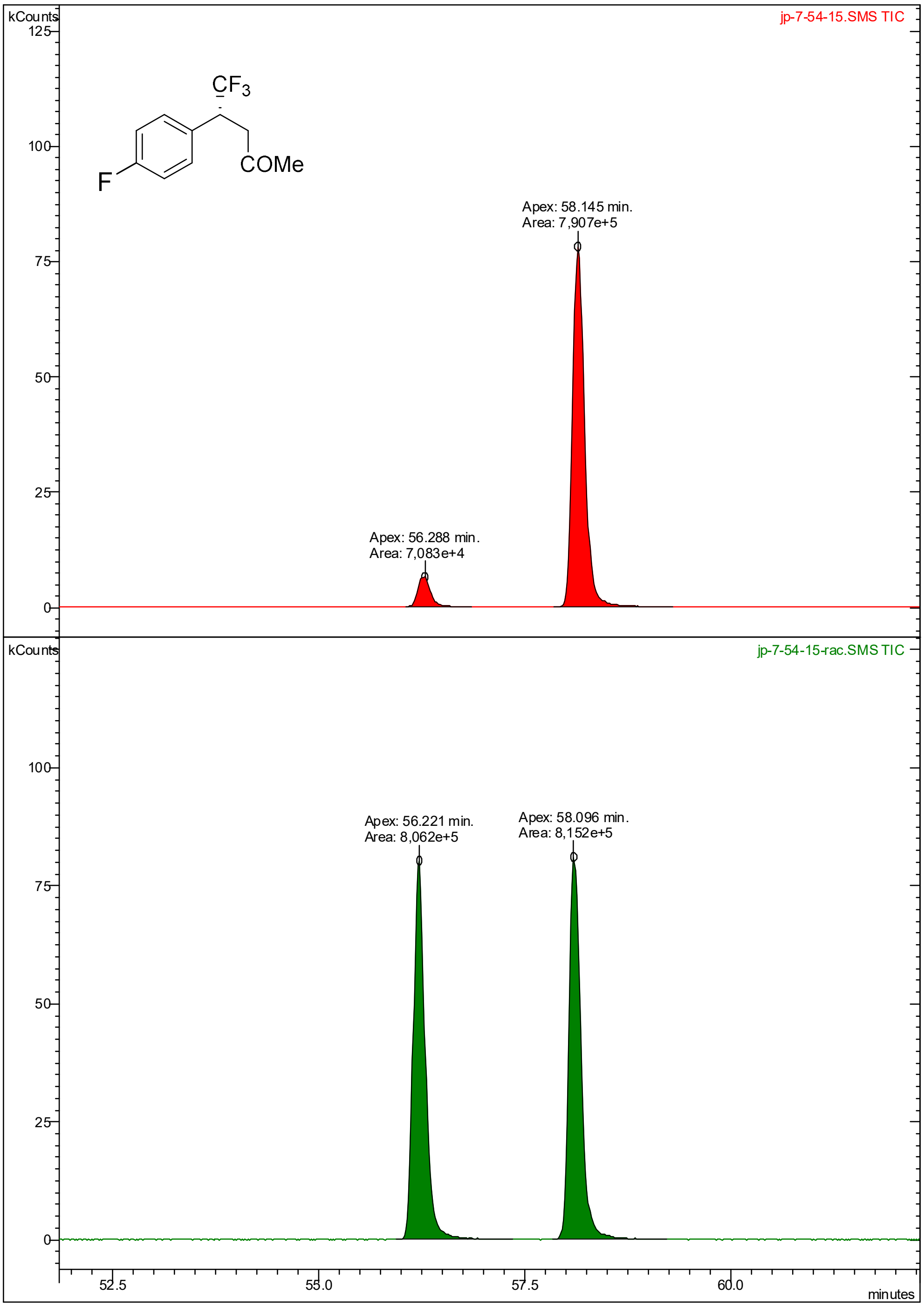

Print Date: 26 Mar 2019 09:24:37

Chromatogram Plots

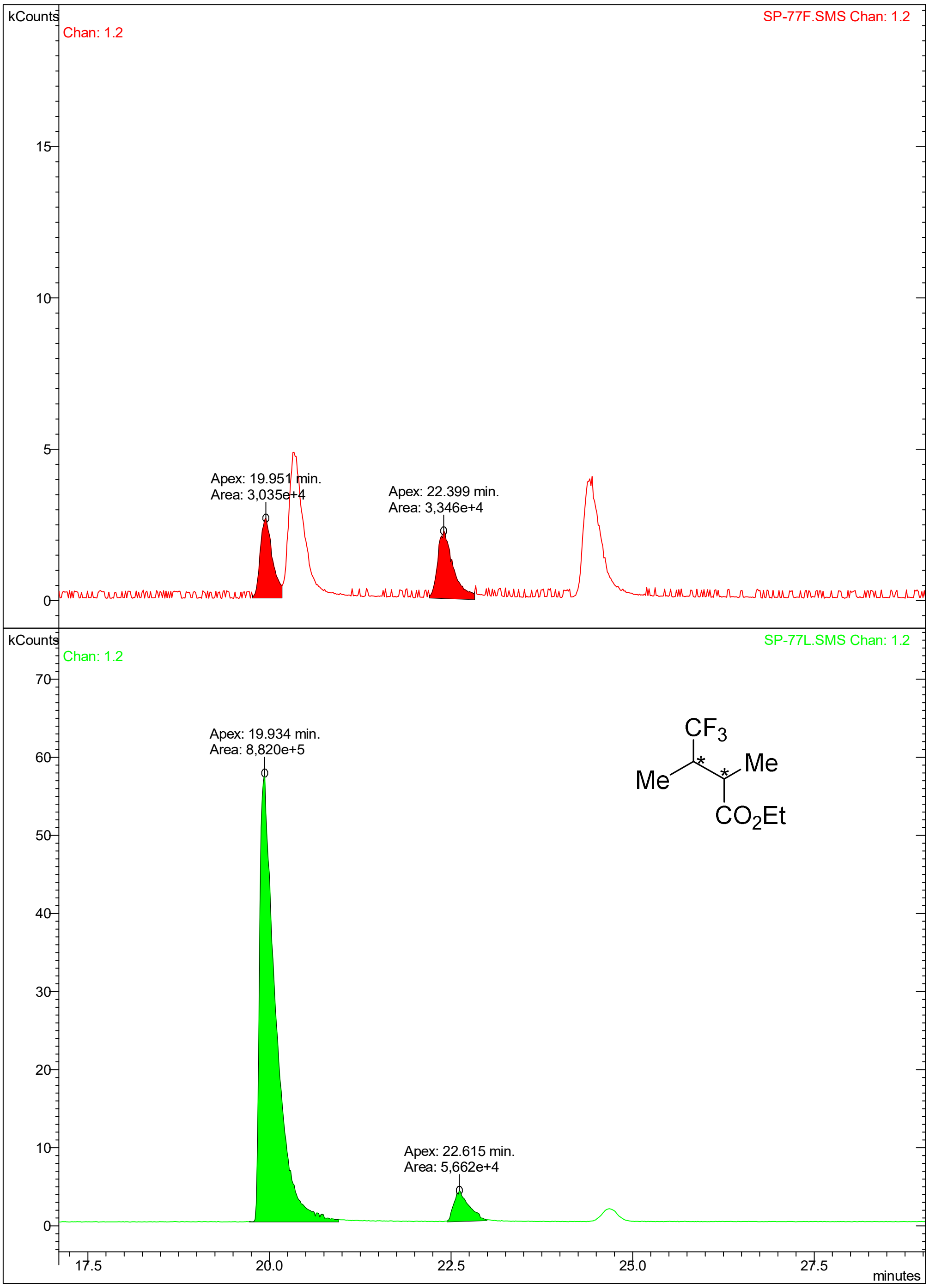

Print Date: 26 Mar 2019 09:31:38

Chromatogram Plots

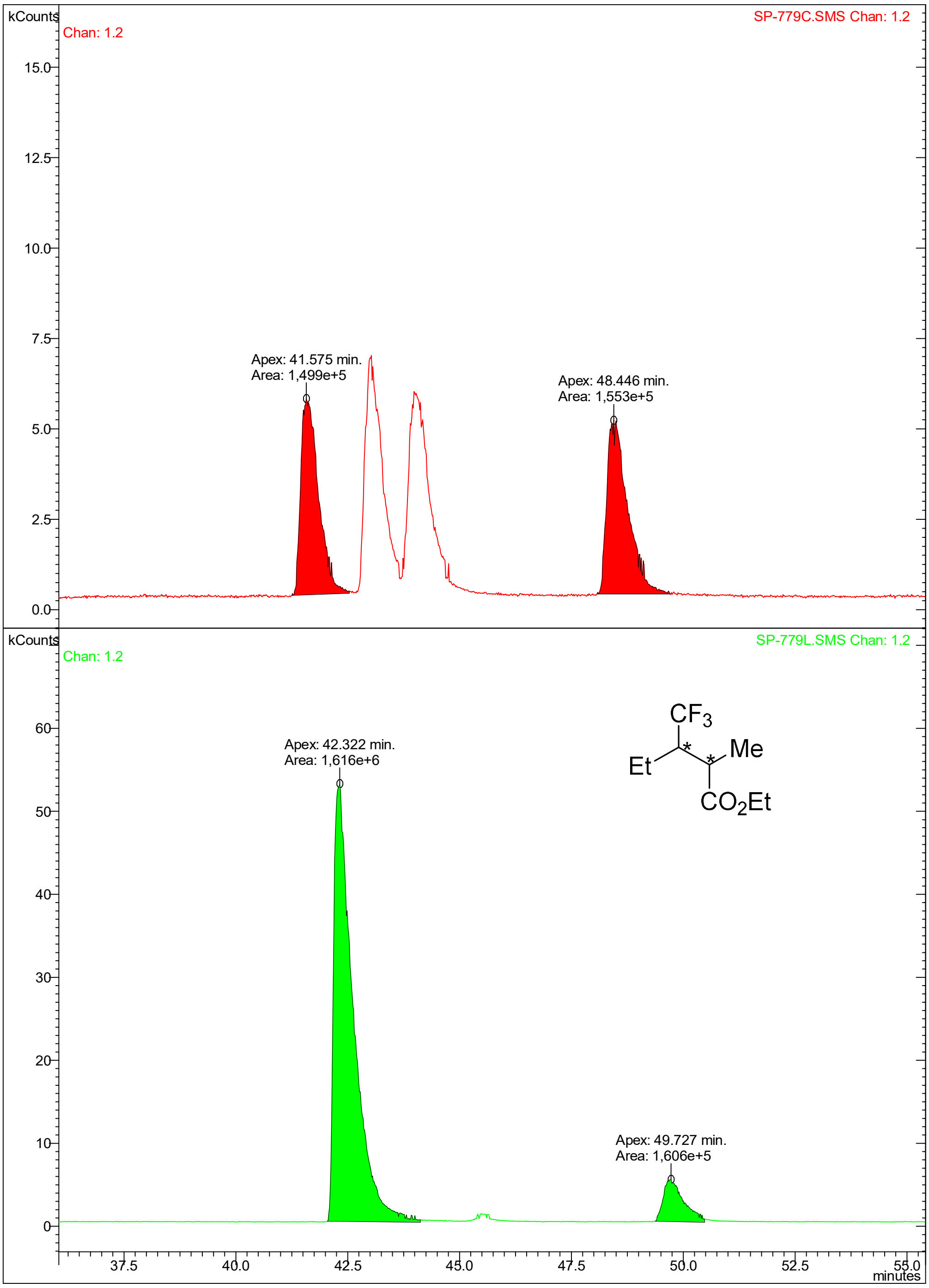

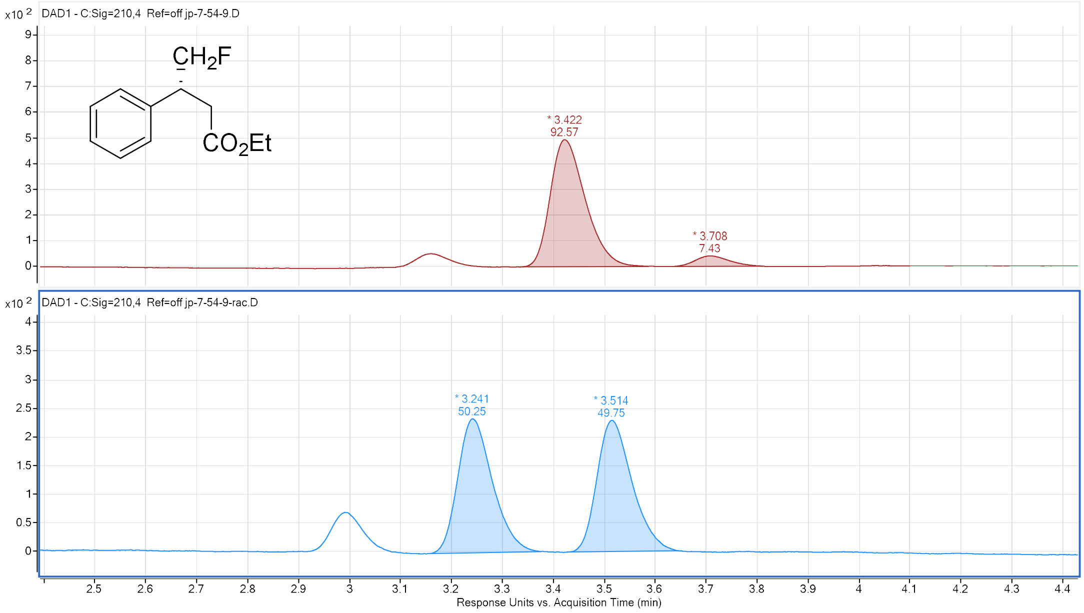
x10 2
DAD1 - C:Sig=210,4 Ref=off jp-7-54-9.D
CH2F
CO2Et
* 3.422
92.57
* 3.708
7.43
x10 2
DAD1 - C:Sig=210,4 Ref=off jp-7-54-9-rac.D
* 3.241
50.25
* 3.514
49.75
Response Units vs. Acquisition Time (min)

Chromatogram Plots

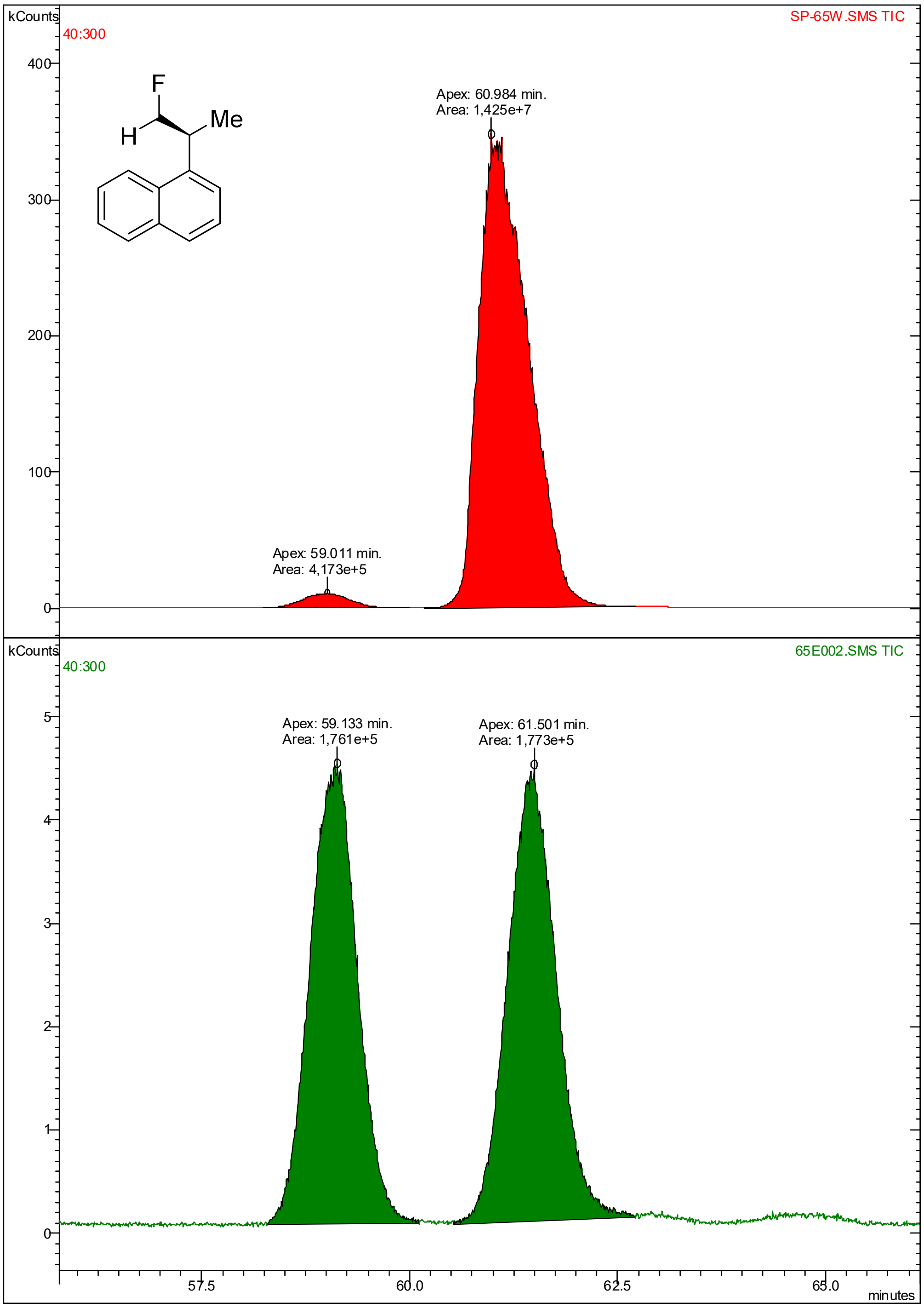

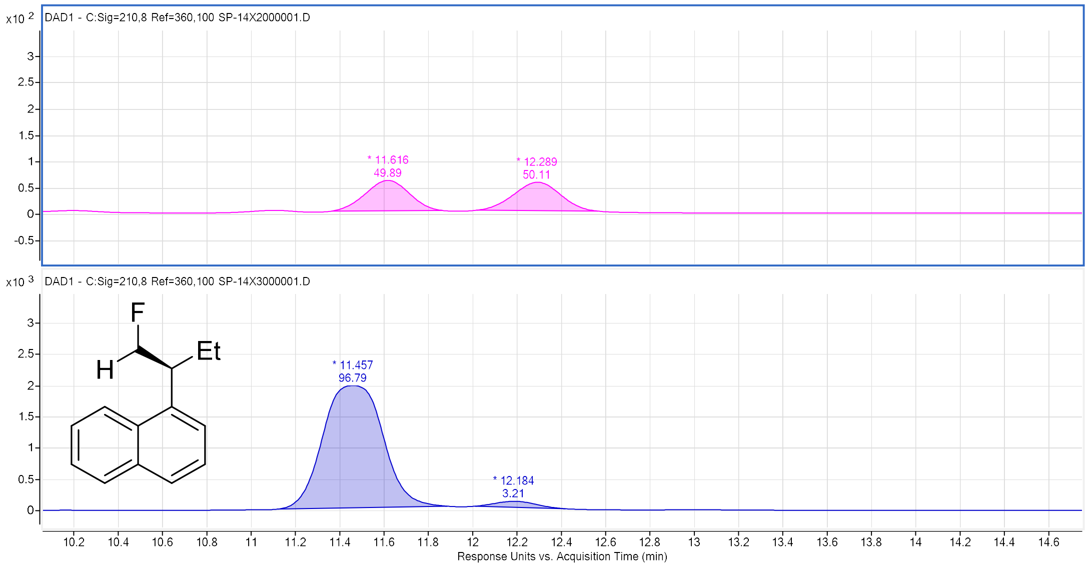
DAD1 - C:Sig=210,8 Ref=360,100 SP-14X2000001.D
* 11.616
49.89
* 12.289
50.11
DAD1 - C:Sig=210,8 Ref=360,100 SP-14X3000001.D
F
H
Et
* 11.457
96.79
* 12.184
3.21
Response Units vs. Acquisition Time (min)

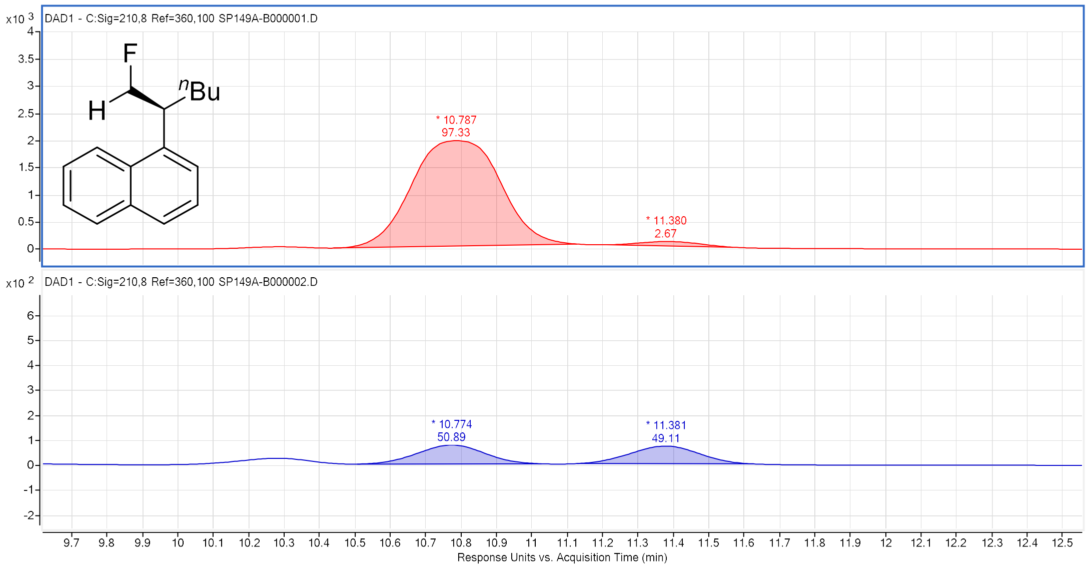
DAD1 - C:Sig=210,8 Ref=360,100 SP149A-B000001.D
F
H
nBu
* 10.787
97.33
* 11.380
2.67
DAD1 - C:Sig=210,8 Ref=360,100 SP149A-B000002.D
* 10.774
50.89
* 11.381
49.11
Response Units vs. Acquisition Time (min)

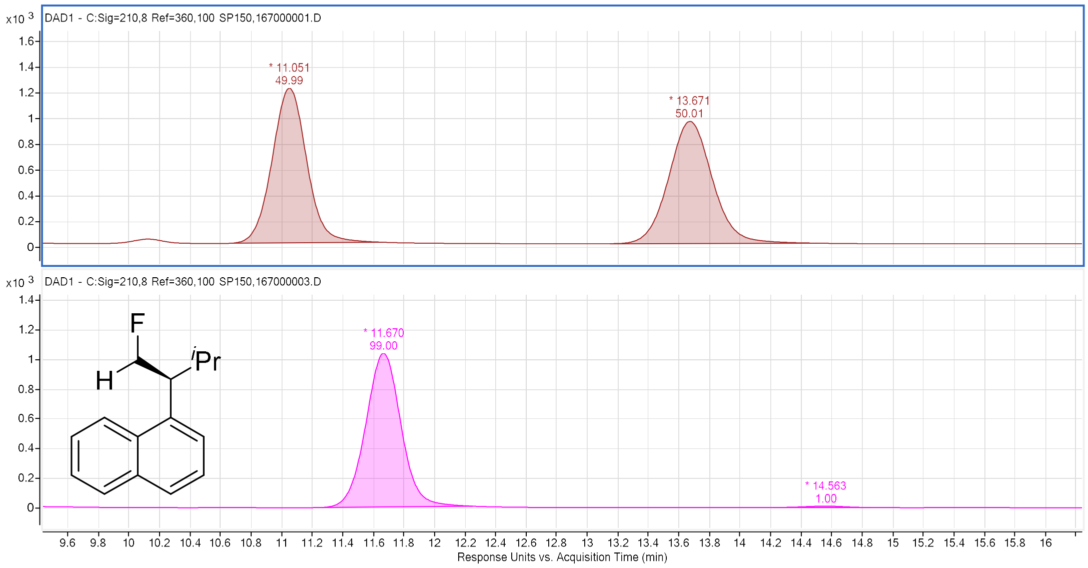
DAD1 - C:Sig=210,8 Ref=360,100 SP150,167000001.D
* 11.051
49.99
* 13.671
50.01
DAD1 - C:Sig=210,8 Ref=360,100 SP150,167000003.D
F
H
iPr
* 11.670
99.00
* 14.563
1.00
Response Units vs. Acquisition Time (min)

Chromatogram Plots

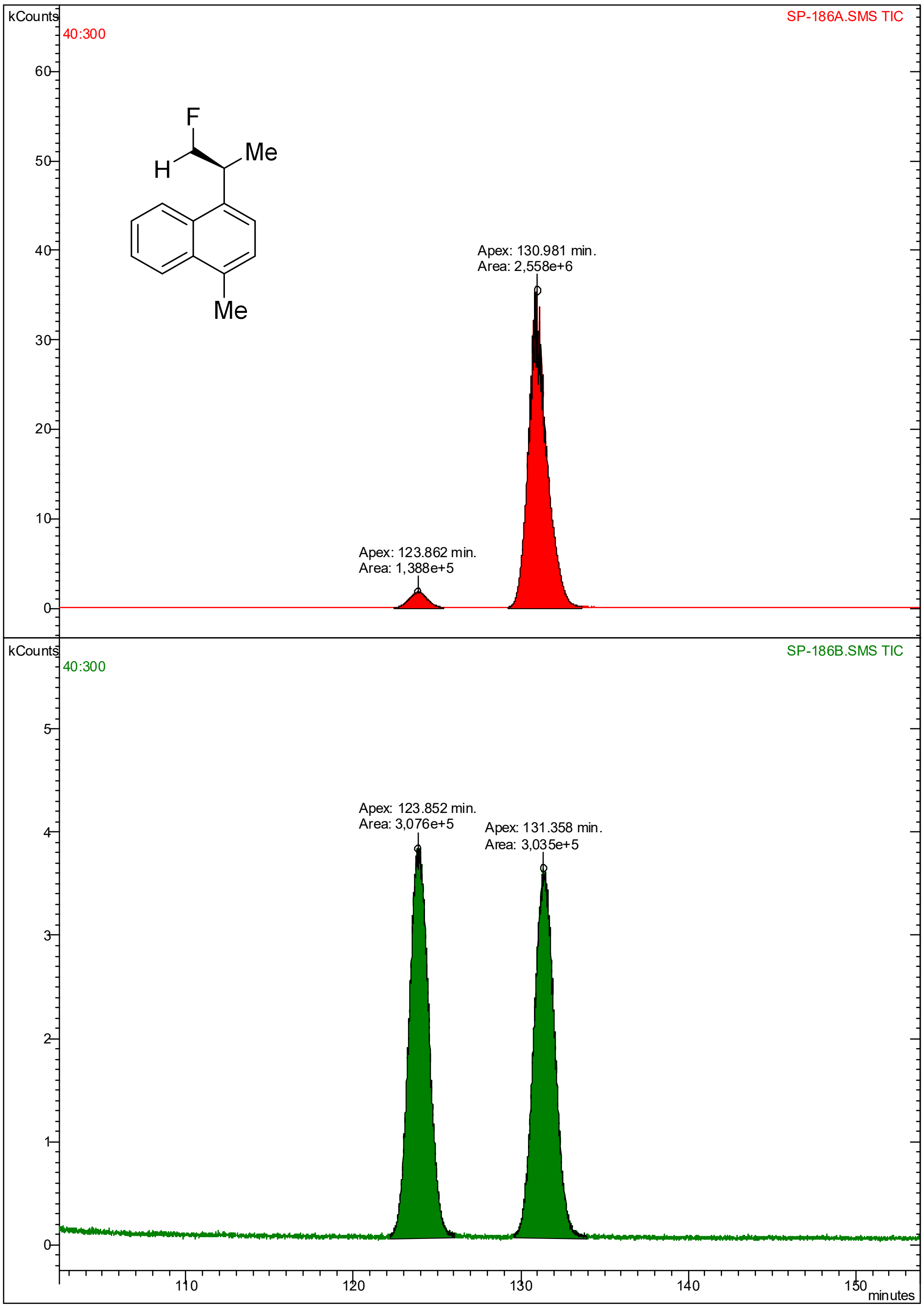

Chromatogram Plots

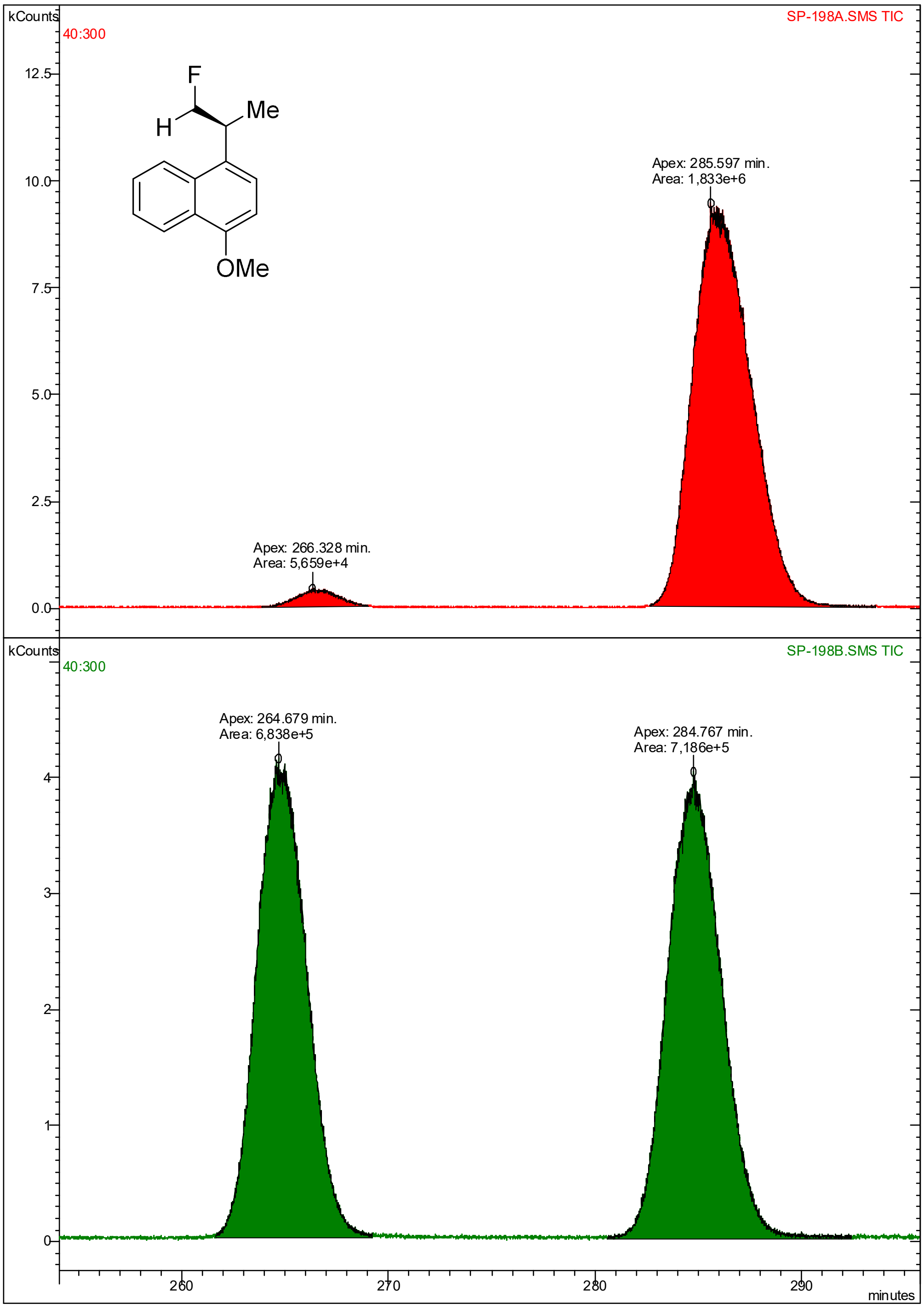

Chromatogram Plots

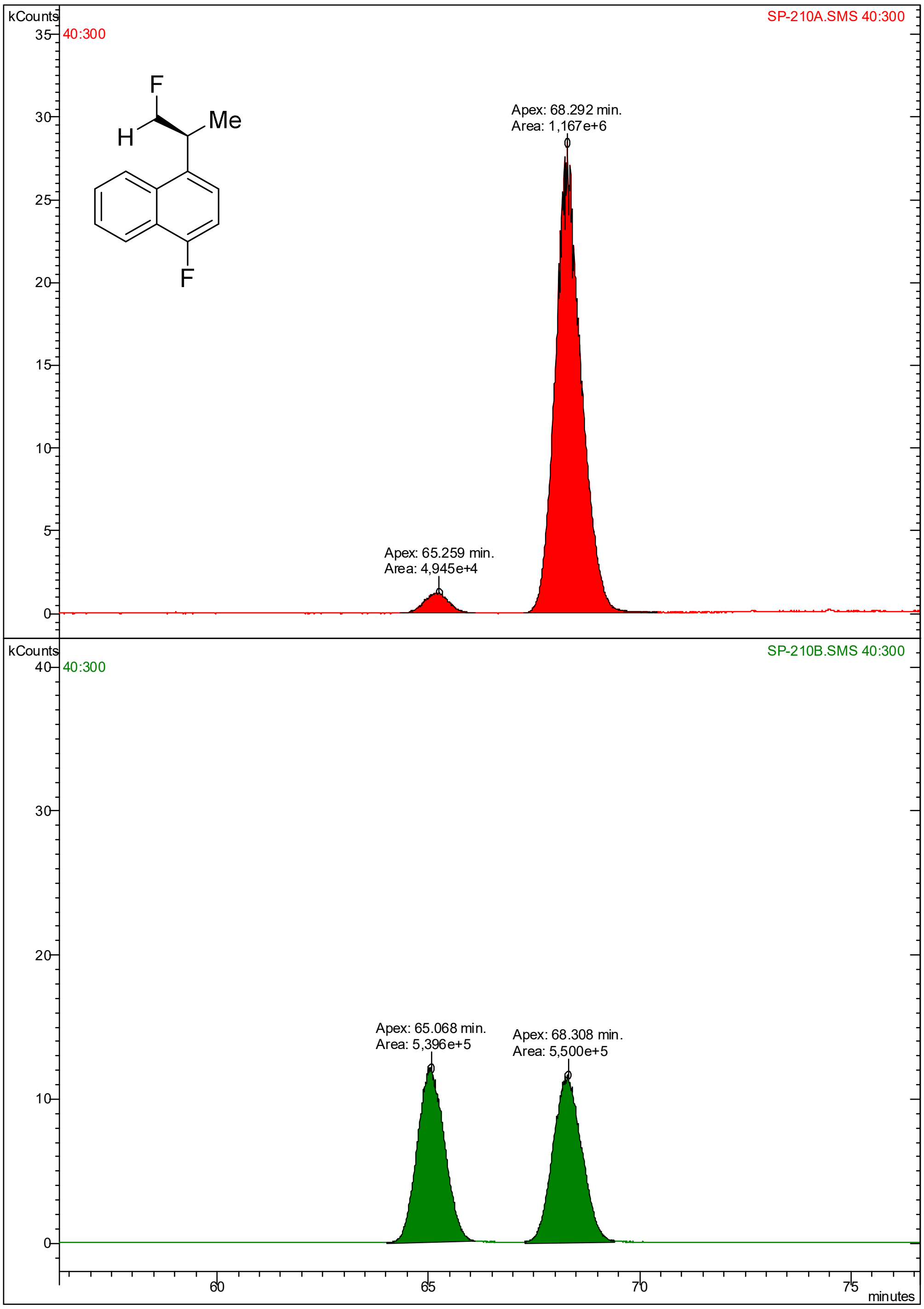

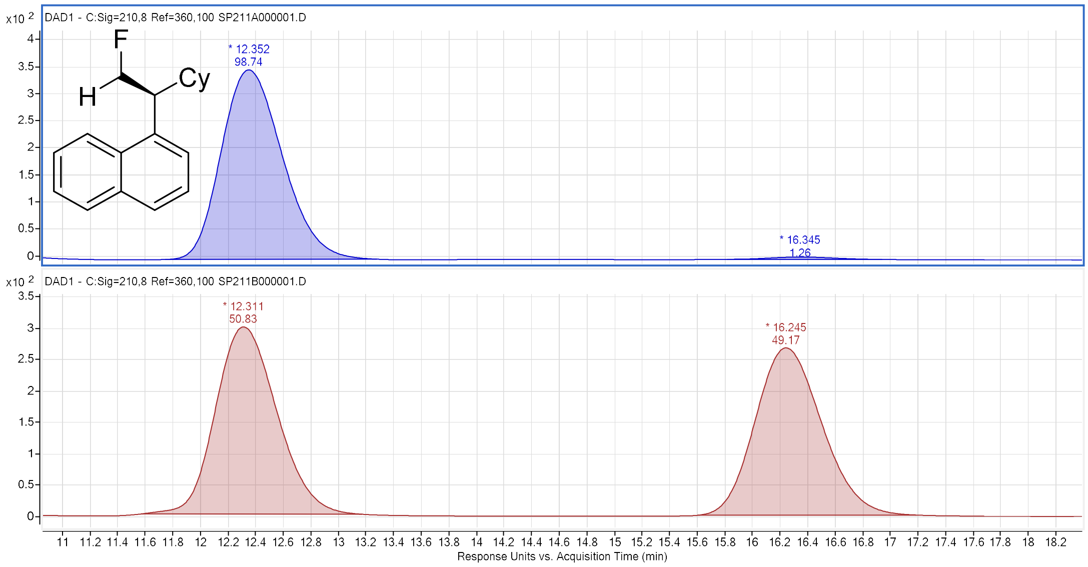
x10 2
DAD1 - C:Sig=210,8 Ref=360,100 SP211A000001.D
F
H
Cy
* 12.352
98.74
* 16.345
1.26
x10 2
DAD1 - C:Sig=210,8 Ref=360,100 SP211B000001.D
* 12.311
50.83
* 16.245
49.17
Response Units vs. Acquisition Time (min)

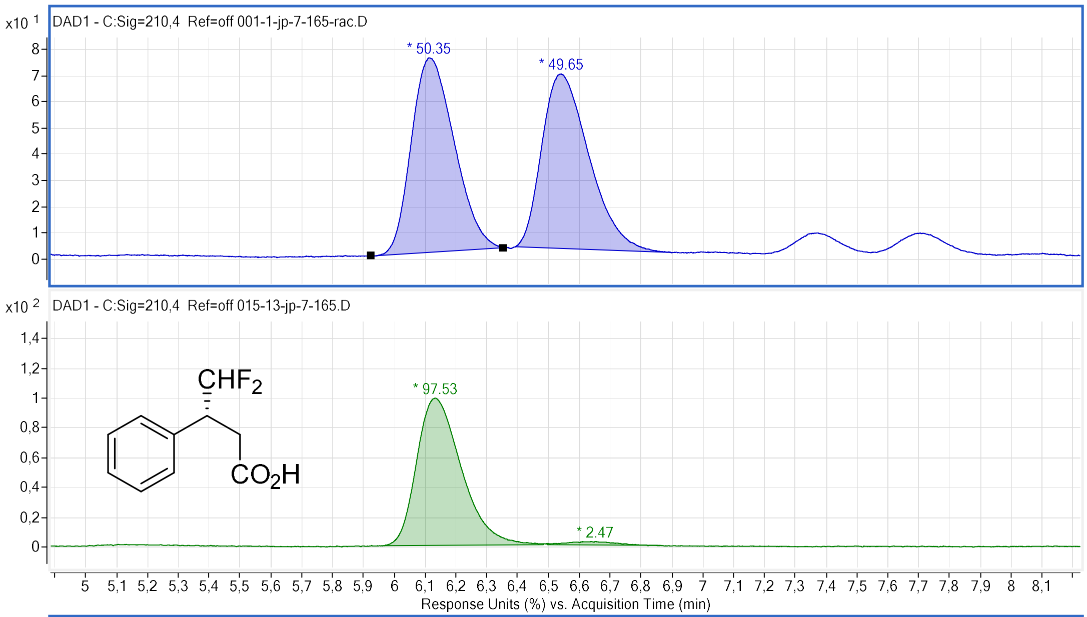
x10 1
DAD1 - C:Sig=210,4 Ref=off 001-1-jp-7-165-rac.D
* 50.35
* 49.65
x10 2
DAD1 - C:Sig=210,4 Ref=off 015-13-jp-7-165.D
CHF2
CO2H
* 97.53
* 2.47
Response Units (%) vs. Acquisition Time (min)

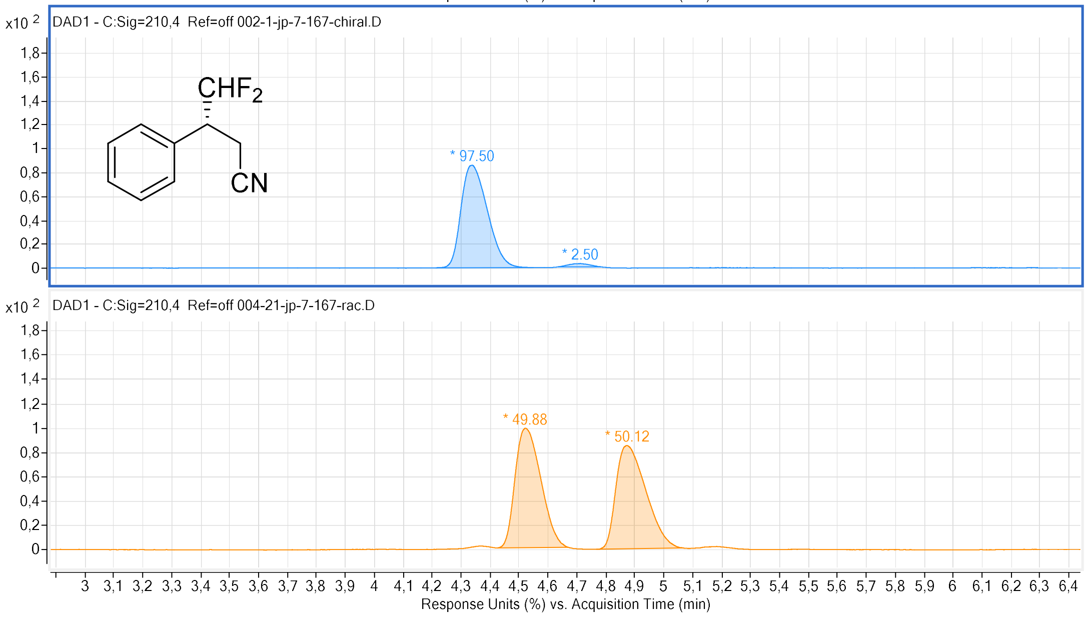
x10 2
DAD1 - C:Sig=210,4 Ref=off 002-1-jp-7-167-chiral.D
CHF2
CN
* 97.50
* 2.50
x10 2
DAD1 - C:Sig=210,4 Ref=off 004-21-jp-7-167-rac.D
* 49.88
* 50.12
Response Units (%) vs. Acquisition Time (min)

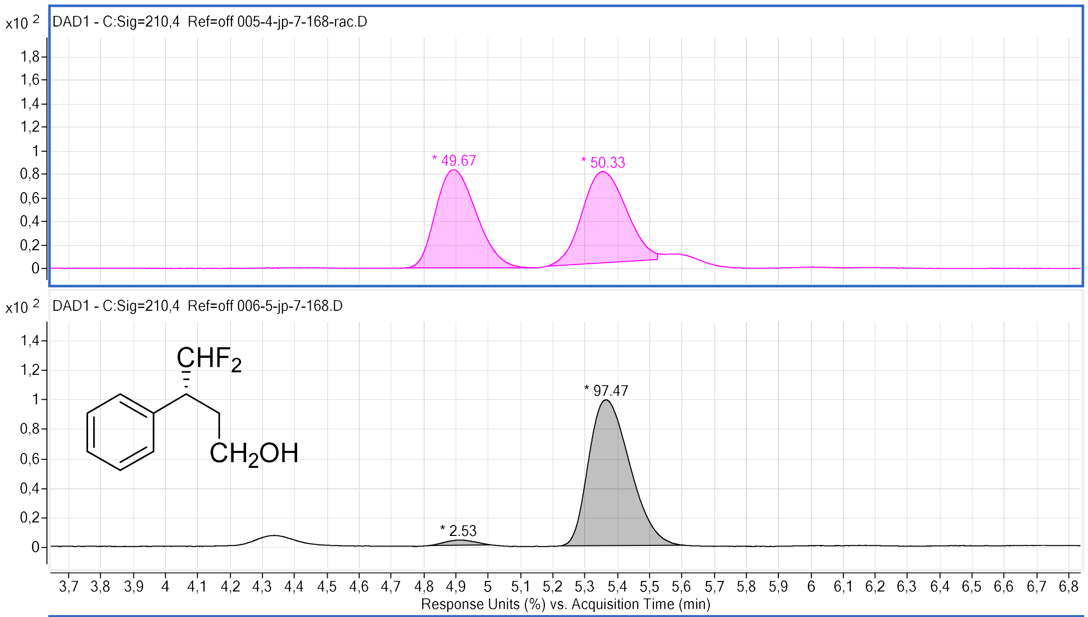
x10 2
DAD1 - C:Sig=210,4 Ref=off 005-4-jp-7-168-rac.D
* 49.67
* 50.33
x10 2
DAD1 - C:Sig=210,4 Ref=off 006-5-jp-7-168.D
CHF2
CH2OH
* 97.47
* 2.53
Response Units (%) vs. Acquisition Time (min)

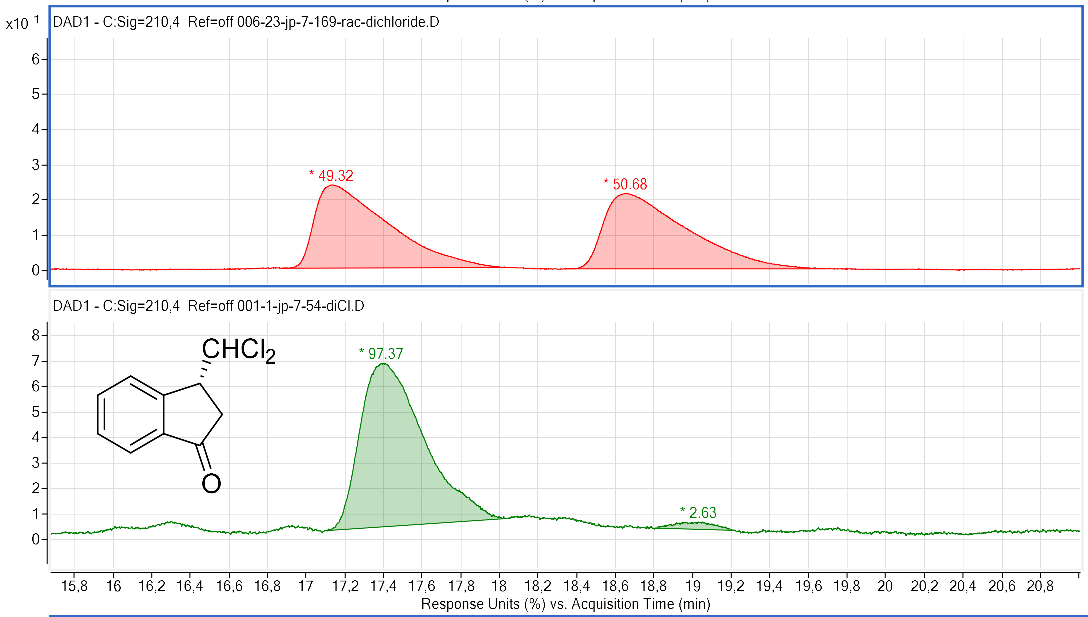
x10 1
DAD1 - C:Sig=210,4 Ref=off 006-23-jp-7-169-rac-dichloride.D
* 49.32
* 50.68
DAD1 - C:Sig=210,4 Ref=off 001-1-jp-7-54-diCl.D
CHCl2
O
* 97.37
* 2.63
Response Units (%) vs. Acquisition Time (min)

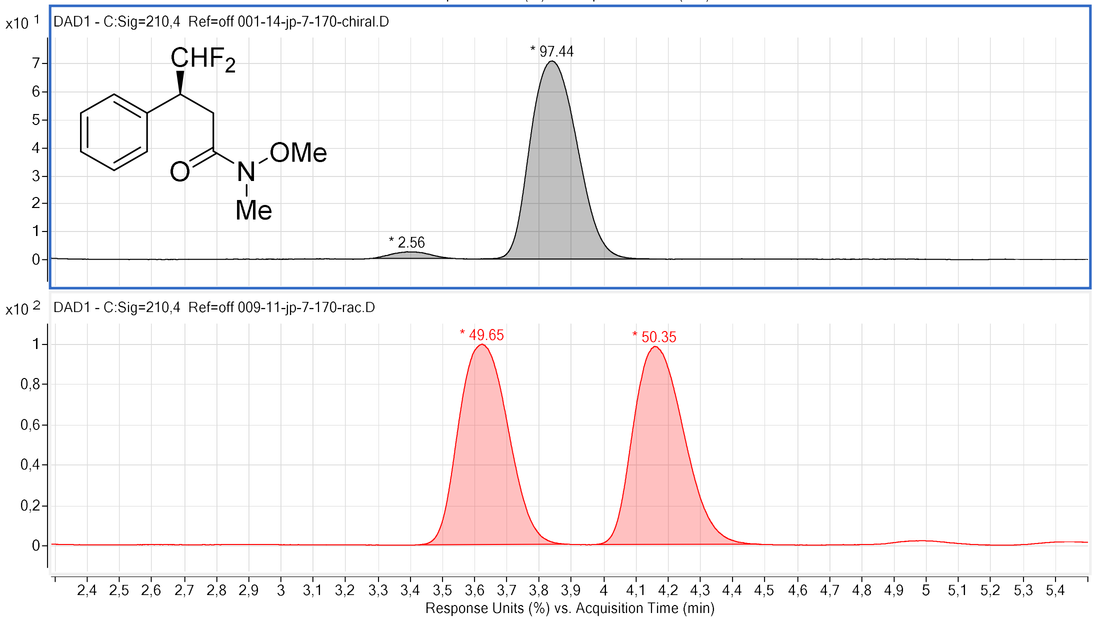
x10 1
DAD1 - C:Sig=210,4 Ref=off 001-14-jp-7-170-chiral.D
CHF2
O
N
OMe
Me
* 97.44
* 2.56
x10 2
DAD1 - C:Sig=210,4 Ref=off 009-11-jp-7-170-rac.D
* 49.65
* 50.35
Response Units (%) vs. Acquisition Time (min)

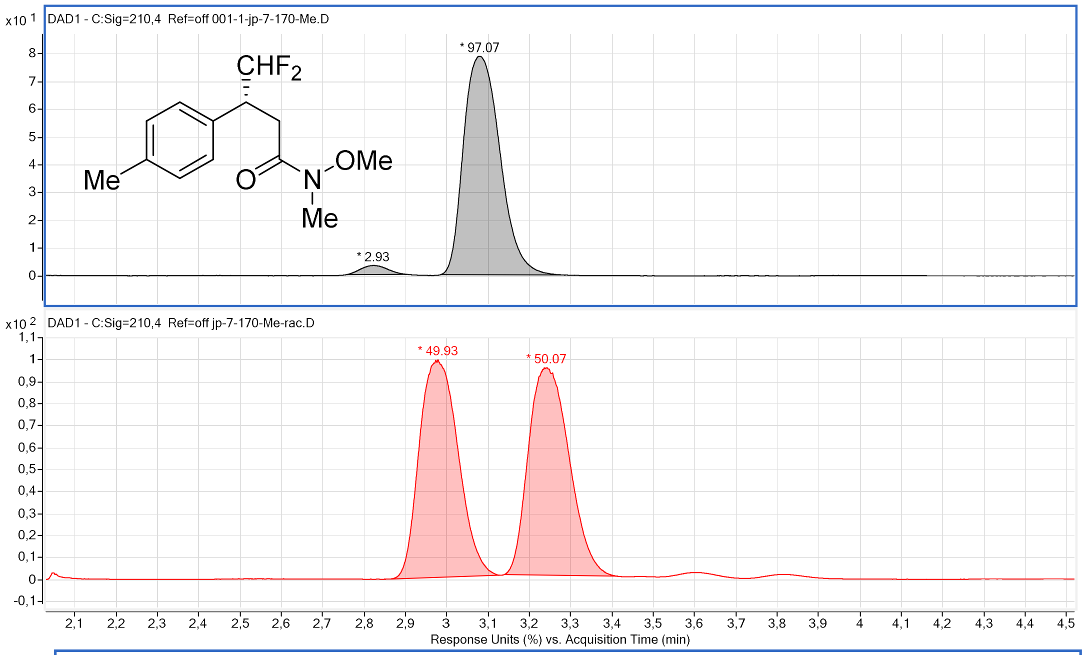
DAD1 - C:Sig=210,4 Ref=off 001-1-jp-7-170-Me.D
* 97.07
* 2.93
CHF2
Me
O
N
OMe
Me
DAD1 - C:Sig=210,4 Ref=off jp-7-170-Me-rac.D
* 49.93
* 50.07
Response Units (%) vs. Acquisition Time (min)

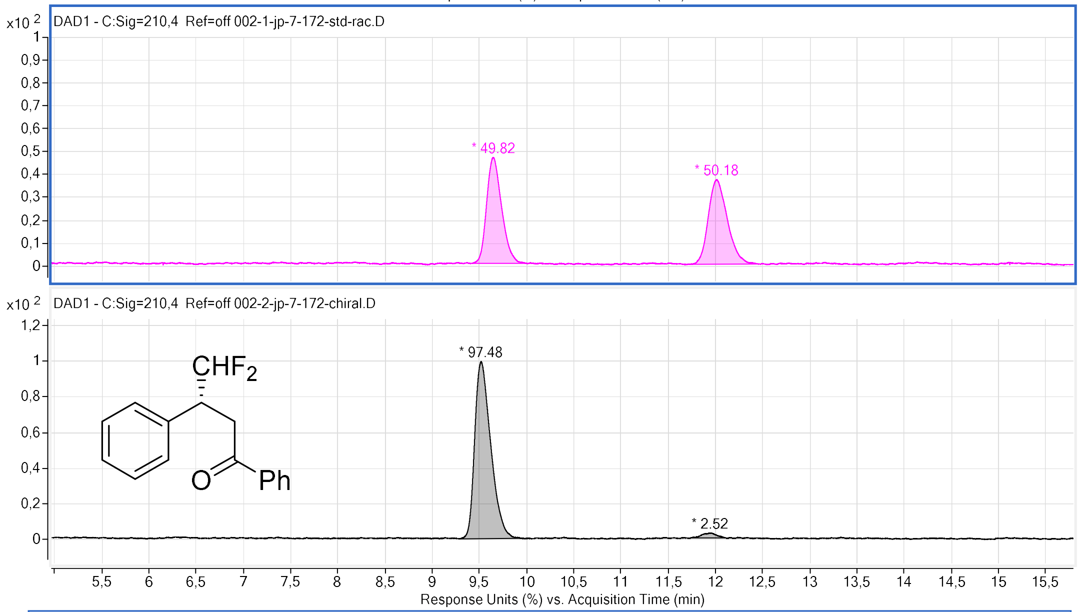
DAD1 - C:Sig=210,4 Ref=off 002-1-jp-7-172-std-rac.D
* 49.82
* 50.18
DAD1 - C:Sig=210,4 Ref=off 002-2-jp-7-172-chiral.D
* 97.48
* 2.52
CHF2
O
Ph
Response Units (%) vs. Acquisition Time (min)

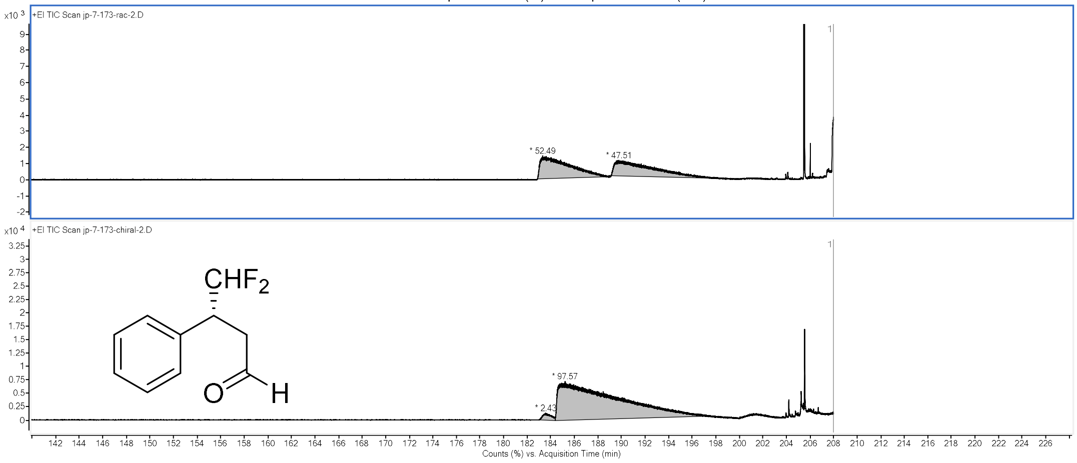
+EI TIC Scan jp-7-173-rac-2.D
* 52.49
* 47.51
+EI TIC Scan jp-7-173-chiral-2.D
* 97.57
* 2.43
CHF2
O
H
Counts (%) vs. Acquisition Time (min)

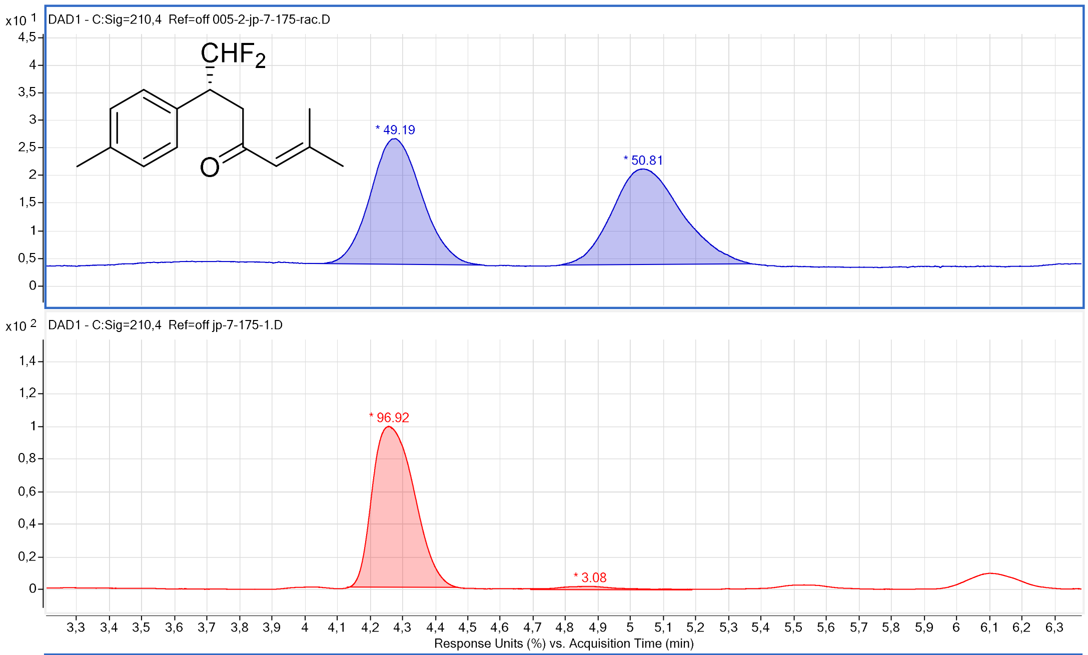

DAD1 - C:Sig=210,4 Ref=off 005-2-jp-7-175-rac.D
CHF2
O
* 49.19
* 50.81
DAD1 - C:Sig=210,4 Ref=off jp-7-175-1.D
* 96.92
* 3.08
Response Units (%) vs. Acquisition Time (min)

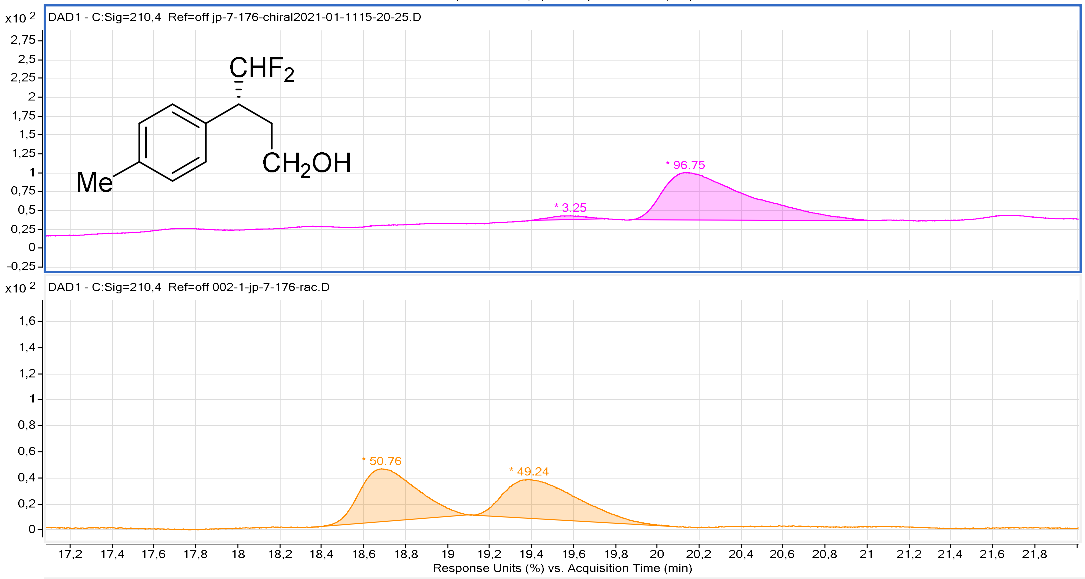

DAD1 - C:Sig=210,4 Ref=off jp-7-176-chiral2021-01-1115-20-25.D
CHF2
Me
CH2OH
* 96.75
* 3.25
DAD1 - C:Sig=210,4 Ref=off 002-1-jp-7-176-rac.D
* 50.76
* 49.24
Response Units (%) vs. Acquisition Time (min)

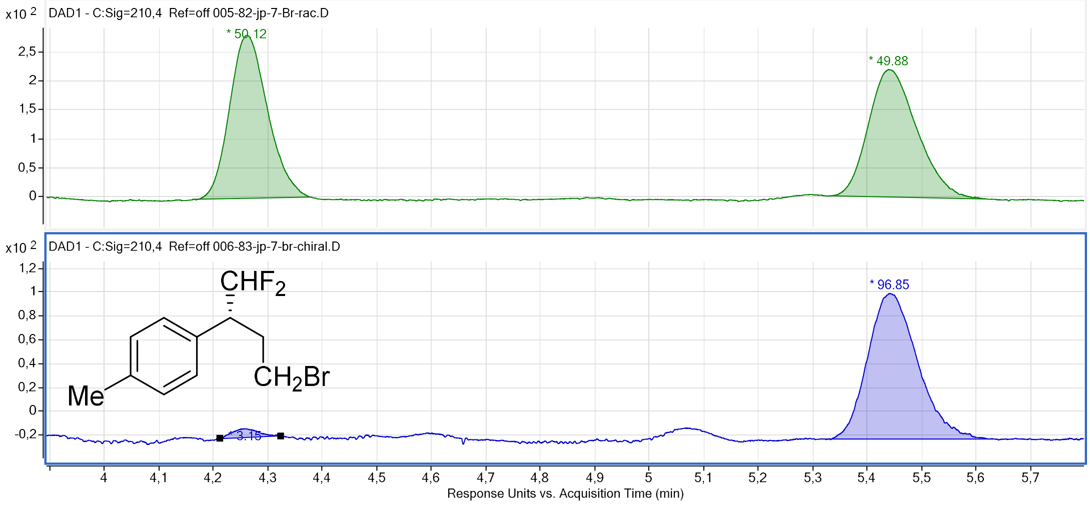

DAD1 - C:Sig=210,4 Ref=off 005-82-jp-7-Br-rac.D
* 50.12
* 49.88
DAD1 - C:Sig=210,4 Ref=off 006-83-jp-7-br-chiral.D
CHF2
Me
CH2Br
* 96.85
3.15
Response Units vs. Acquisition Time (min)

11. References

1. (a) M. Bos, W. S. Huang, T. Poisson, X. Pannecoucke, A. B. Charette and P. Jubault, *Angew. Chem.*, 2017, **129**, 13504-13508; (b) G. S. Prakash, J. Hu and G. A. Olah, *J. Fluor. Chem.*, 2001, **112**, 355-360; (c) D. J. Leng, C. M. Black and G. Pattison, *Org. Biomol. Chem.*, 2016, **14**, 1531-1535.
2. D. Grassi, H. Li and A. Alexakis, *Chem. Commun.*, 2012, **48**, 11404-11406.
3. Q. Liu, X. Shen, C. Ni and J. Hu, *Angew. Chem., Int. Ed.*, 2017, **56**, 619-623.
4. P. Poutrel, M. V. Ivanova, X. Pannecoucke, P. Jubault and T. Poisson, *Chem. Eur. J.*, 2019, **25**, 15262-15266.
